# Exosomes derived from M1 macrophages inhibit the proliferation of the A549 and H1299 lung cancer cell lines via the miRNA-let-7b-5p-GNG5 axis

**DOI:** 10.7717/peerj.14608

**Published:** 2023-01-09

**Authors:** Jingcui Peng, Sa Li, Bin Li, WenXia Hu, Cuimin Ding

**Affiliations:** 1 Department of Respiratory Medicine, The Fourth Hospital of Hebei Medical University, Shijiazhuang, China; 2 Department of Construction, The Fourth Hospital of Hebei Medical University, Shijiazhuang, China

**Keywords:** Lung cancer cells, M1 macrophages, Exosomes, MiRNA-let-7b-5p, GNG5

## Abstract

**Background:**

Almost all cells are capable of secreting exosomes (Exos) for intercellular communication and regulation. Therefore, Exos can be used as a natural therapeutic platform to regulate genes or deliver drugs to treat diseases. M1 macrophages inhibit tumor growth by releasing pro-inflammatory factors. This study explored the applicability of M1 macrophage exosomes (M1-Exos) as gene carriers and the effects on GNG5 protein, and further examined whether macrophage repolarization could inhibit tumor activity.

**Methods:**

M0 macrophages were polarized toward M1 using vitexin. Exos were obtained from M1 macrophages by ultra-centrifugation. The transwell non-contact co-culture system was used to co-culture M1 macrophages with HLF-*α* human lung epithelial cells or A549 or H1299 lung cancer cells. MTT, scratch, and transwell assays were used to detect the cell viability, migration, and invasion ability of cells in the four groups. Flow cytometry was used to detect the apoptosis rate of each group, and western blot (WB) analysis was performed to detect the change in the expression of proliferation- and apoptosis-related proteins. We screened the differentially expressed microRNAs using quantitative polymerase chain reaction technology. Luciferase reporter analysis was performed to explore the interaction between miRNA and protein. We used Xenografted A549 tumors in nude mice to study the effect of M1-Exos on tumor cell growth in vivo.

**Results:**

The results showed that, under the M1 macrophage co-culture system, lung cancer cell viability, invasion, and migration ability decreased, and the number of apoptotic cells increased, will all indicators being statistically significant (*P* < 0.05). The expression levels of PCNA, KI67, and Bcl-2 decreased significantly, but that of Bax increased (*P* < 0.05). Exosomes can have the same effect on tumor cells as M1 macrophages. Exosomes can transport miR-let-7b-5p to tumor cells, and miR-let-7b-5p can inhibit tumor cell proliferation and promote tumor cell apoptosis by regulating the GNG5 protein level.

**Conclusions:**

M1-Exos inhibit the proliferation, invasion, and metastasis of lung cancer cells through miRNA-let-7b-5p and GNG5 signaling pathways and inhibit the anti-apoptotic ability of lung cancer cells.

## Introduction

In recent years, lung cancer has become one of the most serious malignant tumor diseases in China and even the world (Wu, Wang & Zhou, 2021). In terms of the incidence of malignant tumors, lung cancer ranks first nationwide, with up to 780,000 cases diagnosed per year (Cao & Chen, 2019). The incidence of lung cancer in men and women ranks first and second among all cancers, respectively, and lung cancer carries the highest mortality rate  (Rabinel et al., 2022; Tirilomi et al., 2022). Among the types of lung cancer, non-small-cell lung cancer (NSCLC) is most common, with an incidence rate of up to 85%. According to investigations, lung adenocarcinoma has become the most common pathological type of lung cancer (Murakami et al., 2021). Smoking, second-hand smoke exposure, and drinking habits are all known factors that increase the chance of lung cancer. In China, the situation of lung cancer is severe. Invasion and metastasis are the most important common causes of death in patients with advanced lung cancer (Wang et al., 2021).

In the microenvironment where a variety of tumor cells are located, macrophages, as the main stromal cells, rely on each other and communicate with one another through non-contact signal molecules (Quail & Joyce, 2013). Macrophages in the tumor microenvironment are tumor-associated macrophages, also known as M1 macrophages. Recent studies have found that M1 macrophages can escape the body’s anti-tumor response and promote tumor blood vessel formation and proliferation, migration, and invasion (Tu et al., 2021). In the tumor microenvironment, M1 macrophages can recognize normal tissues and cancerous cells in the body and exert anti-tumor effects through immune recognition.

Exosomes (Exos), tiny membrane vesicles secreted by most cells, have lipid bilayer structures and measure mostly 40-100 nm in diameter (Théry, Zitvogel & Amigorena, 2002). Exos contain a large number of proteins, nucleic acids, and lipid components that are closely related to their source and function, but they do not contain DNA components  (Simpson et al., 2009; Ogawa et al., 2011). Various kinds of miRNAs, mRNAs, lncRNAs, tRNAs, snRNAs, snoRNAs, and circRNAs can be detected in Exos. These RNAs have the function of regulating gene expression and can be used as potential biomarkers (Jr et al., 2020). As a carrier of signal transmission between cells, Exos play a vital role in tumor growth regulation (Guo et al., 2021). As signal molecules, Exos can participate in the regulation of cell proliferation, immune response, cancer development, inflammatory response and other physiological and pathological activities  (Fortunato et al., 2019; Ingenito et al., 2019; Wei et al., 2020).

In recent years, it was reported that M1 macrophages can inhibit the proliferation, migration, and invasion of tumor cells through a variety of means, but there are few reports on the specific molecular pathway mechanism at play. Therefore, we explored the invasion of lung cancer by M1 macrophage Exos (M1-Exos). The study of molecular mechanisms related to migration ability can bring some new ideas for the clinical treatment of lung cancer metastasis and may provide new ideas for the future discovery of new clinical target sites and therapeutic drugs.

## Material and Methods

### Materials

A549 and H1299 lung cancer cells, HLF-*α* fibroblasts, and M0 macrophages were all purchased from American Type Culture Collection (Manassas, VA, USA); F12K medium and pancreatin were purchased from Gibco Laboratories (Gaithersburg, MD, USA); exosome-depleted fetal bovine serum was purchased from Gibco (A2720801; ThermoFisher Scientific, Waltham, MA, USA); cell lysate was purchased from Invitrogen Biotechnology Co., Ltd. (Carlsbad, CA, USA); leonurusine was purchased from Nanjing Jiancheng Co.; MTT kit was purchased from Ameresco; CD86, CD80, CD16, PCNA, KI67, Bax, and BCL antibodies were purchased from Abcam (Cambridge, UK); goat anti-rabbit immunoglobulin G (secondary antibody) was purchased from Beijing Zhongshan Jinqiao Biotechnology Co.; Trizol was purchased from Invitrogen; polymerase chain reaction (PCR) primers were purchased from Nanjing GenScript Biotechnology Co., Ltd.; PCR primers were all designed by the Shanghai Shenggong Co.; a FISH staining kit was purchased from Soleibao Biotech; PVDF membrane and an ECL kit were purchased from Mi1lipore (Burlington, MA, USA); a Transwell chamber was purchased from Corning, Inc., (Corning, NY, USA); Matrigel glue was purchased from Becton, Dickinson and Co. (Franklin Lakes, NJ, USA); a dual luciferase reporter gene detection kit was purchased from Biyuntian Co.; and mice were purchased from Beijing Weitong Lihua Co.

### Cell culture

A549 and H1299 lung cancer cells and normal human M0 cells were both cultured in a complete medium containing 10% exosome-depleted fetal bovine serum and 1% penicillin in RPMI 1640 in a 37 °C, 5% CO_2_ incubator. When the cell growth density reached 80%–90%, trypsin digestion was completed to collect the cells; we continued with subculturing in an appropriate proportion and selected the cells in the best period of the logarithmic growth phase for follow-up experiments.

### Polarization identification of M1 type macrophages

We adjusted the cell density of M0 cells in the logarithmic growth phase, added 50 µmol/L of vitexin, cultured the cells at 37 °C for 24 h, discarded the supernatant, washed the cells twice with sterile phosphate-buffered saline (PBS), observed the cell morphology under an inverted optical microscope, and confirmed their status and collected them. We used flow cytometry to detect whether the macrophages were polarized, including specifically to M1 macrophages; at the same time, we used the M1 macrophage markers CD86, CD16, and CD80 to confirm that the macrophages were polarized into M1 macrophages by immunofluorescence testing.

### Co-cultivation of M1 macrophages with A549 or H1299 cells

M1 macrophages no longer have the ability to divide and proliferate. We resuspended them and added them to the upper chamber of the Transwell co-culture system while also adding the resuspended A549 or H1299 cells to the lower chamber. We used RPMI 1,640 complete medium to culture cells at 37 °C without affecting the cells. At the same time, a control group was set up, involving the addition of M1 macrophages to the upper chamber and resuspended human normal cells to the lower chamber, which were cultured for a certain period of time under the same conditions. A normal cell control group, a normal cell and M1 macrophage group, a lung cancer cell control group, and a lung cancer cell and M1 macrophage group were also set up.

We centrifuged the cultured M1 macrophages at 2000 ×g for 10 min to remove cell debris, and then filtered them with a 0.22-µm filter membrane, collected the supernatant, and added it to an ultracentrifuge tube for centrifugation at 100000 ×g for 4 h. Subsequently, the pellet was collected and resuspended in PBS, and then finally centrifuged at 100000 ×g for 1 h. The resulting pellet contained Exos.

### Isolation of Exos and purification

Exos were collected from M1 macrophage medium after ultra-centrifugation. Conditioned medium was centrifuged at 2000 ×g for 30 min, then at 10000 ×g for 45 min at 4 °C to remove cells and debris. The supernatant was taken and filtered through a 0.45 µm filter membrane. The filtrate was collected, then centrifuged at 100000 ×g for 70 min at 4 °C. After removing the supernatant and resuspension in PBS, the sediments were centrifuged at 100000 ×g for 70 min at 4 °C. The supernatant was removed and the pellet was resuspended with 100 µL of PBS. The 20 µL exosome solution was observed by electron microscopy (Tecnai 12; Philips).

### Cells viability detected by MTT

Each group of cells was seeded in a 96-well plate at a cell density of 1 ×10^4^ cells/well, with 100 µL per well, and each group had 6 replicate wells. We cultured cells for 24, 48, and 72 h, respectively, then observed the cell survival status. Then, 20 µL of 0.5% MTT reagent was added to the wells, the content of which was about 5 mg/mL. After culturing to 24, 48, or 72 h, we discarded the medium and washed cells with PBS 2-3 times, and then added 200 µL of dimethyl sulfoxide solution to each well and gently shook the plate with a shaker for 7–9 min to fully dissolve the crystals for observation. A 490-nm wavelength was chosen to measure the absorbance value in the microplate reader, where we recorded and compared the survival of the cells. The experiment was replicated three times.

### Scratch detection of cell migration ability

We inoculated the A549 or H1299 cells in the log phase into a 6-well plate. When the cell density reached about 80%, we scratched the center of the plate with a sterile 10-µL pipette tip and washed the plate twice with sterile PBS. The state of the cells in the six-well plate at this time was defined as 0 h. We used an inverted microscope to record the width of the scratch. After incubating cells in a 37 °C incubator for 24 h, we observed and recorded the healing of the scratch under the same field of view, taking a photograph to record the result. Using the formula “scratch healing rate (%) = 0-h scratch width − 24-h scratch width)/0 h scratch width ×100%.” The experiment was replicated at least three times.

### Transwell assay detected the cell invasion ability of each group

First, after diluting the Matrigel with RPMI 1640 culture medium and mixing it, we took 50 µL and sealed it on the upper chamber membrane, then added the A549 or H1299 cell suspension to the upper chamber and set the tumor cells that had not been treated with the co-culture medium as the control group. In total, 3 holes were set up in each group. After culturing cells for 48 h in an incubator at 37 °C and a 5% CO_2_ volume fraction, we rinsed the cells gently with PBS, removed the cells on the membrane pores of the chamber that had not passed through the membrane, and fixed the cells passing through the lower chamber with 4%. After fixation for 20 min, we stained cells with crystal violet for 20 min, took photos with an inverted optical microscope to count the number of cells passing through the lower chamber, the total number of cells passing through the lower chamber was counted on three random optical fields for each group.

### Flow cytometry to detect cells apoptosis

Before starting the experiment, we prepared the sample cells. The test cells in each group were cultured normally to the exponential growth phase with a density of 70%. After the cells were digested with 0.25% trypsin, flow cytometry was used to observe the apoptosis of the cells in each group.

We added two mL of PBS to the prepared cells, centrifuged at 1000 rpm for 5 min, washed twice, and discarded the supernatant. We resuspended the cells in FCM staining buffer, added 100 µL per tube to the flow loading tube, added 5 µL of CD19-eFluor 450 antibody to each tube, and incubated cells for 30 min at 4 °C in the dark. Then, we washed cells 2–3 times with two mL of FCM staining buffer, centrifuged them at 1000 rpm for 5 min, and discarded the supernatant. Next, we added 100 µL of FCM staining buffer to resuspend the cells, then added 100 µL of fixation solution and mixed it with the cell suspension, incubated cells at room temperature for 20 min in the dark, centrifuged them at 1,500 rpm for 5 min, and discarded the supernatant. Finally, we added the newly permeabilization buffer and configured at 1,500 rpm to wash the cells 2–3 times, 5 min each time, and then resuspended the cells in 100 µL of FCM staining buffer, added 10 µL of BSA blocking solution to block, and incubated the cells in the dark at room temperature for 15 min. We loaded the sample on the flow cytometer as soon as possible after the operation was over. The experiments were replicated three times.

### Detection of cell proliferation markers and apoptosis markers by Western blot

We collected the co-cultured cells, lysed the cells for 20 min, and then centrifuged the cell lysate in a centrifuge at 15, 000 ×g for 30 min at 4 °C. Here, the supernatant was the protein extract, and a BCA kit was used to determine the concentration of extracted protein. Separate gel and concentrated gel were configured in polyacrylamide gel. The protein sample and loading buffer were mixed proportionally, boiled, loaded, and transferred to the membrane for measurement. Each sample had three repeated wells for testing. We blocked PVDF with 5% horse serum albumin blocking solution at 37 °C for 2 h, then rinsed with PBS three times; added PCNA, KI67, Bax, and Bcl-2 primary antibodies and let everything stand overnight at 4 °C; rinsed with PBS three times and added fluorescence anti-II, then incubated the PVDF at 37 °C for 1 h and washed it three times; added the prepared color developing solution; and stored in the dark. The ImageJ software program (U.S. National Institutes of Health, Bethesda, MD, USA) was used to analyze the gray value of protein bands. When compared to the internal reference *β*-actin, the result indicates the relative expression level of each protein. These experiments were replicated three times.

### Exos rely on the microRNA (miRNA) axis to regulate the migration and invasion of lung cancer cells

Several differential miRNAs abnormally expressed by Exos in lung cancer cells were verified through literature and quantitative PCR, and the most different miRNA, miRNA-let-7b-5p, was selected as the follow-up research target. Lentiviral vector and pcDNA3.1(+) plasmid vector were used to construct A549 or H1299 lung cancer cell overexpression and knockdown miRNA-let-7b-5p models and to set up a control group. MTT detection of cell viability, scratch detection of cell migration, Transwell detection of cell invasion ability, flow cytometry detection of cell apoptosis, and WB detection of cell proliferation markers and apoptosis marker proteins were performed in each group. These experiments were replicated three times.

### miRNA-let-7b-5p targeting to regulate the migration and invasion of lung cancer cells

A549 or H1299 lung cancer cells were transferred to a six-well plate for culture and placed in a cell incubator containing 5% CO_2_ at 37 °C for 18-24 h so that the cells reached 70%–80% of the plate bottom area during transfection. We added the transfection solution of the dual fluorescein kit to the corresponding well, 100 µL/well, gently shook the culture plate to make evenly distribute it, and placed the plate in a 37 °C, 5% CO_2_ cell incubator. After the second transfection, cell lysate was added, and the cell supernatant was collected for fluorescence detection.

We screened miRNA-let-7b-5p to target GNG5 and used lentiviral interference and overexpression technology to construct A549 cell GNG5 overexpression and interference cell models. Then MTT assay, scratch assay, Transwell assay, flow cytometry, and WB were used to detect changes in cell-related indicators. These experiments were replicated three times.

### Animal testing

Five-week-old nude BALB/c mice (Experimental Animal Center of Hebei Medical University, Shijiazhuang, China) were maintained under standard conditions at 22 °C with a 12-h light/dark cycle and free access to food and water. Mice were housed and adapted to the breeding environment for two weeks before the experiment. All animal procedures conformed to the Guide for the Care and Use of Laboratory Animals published by the US National Institutes of Health (NIH Publication, 8th Edition, 2011) and were approved by the Laboratory Animal Ethical Committee of Fourth Hebei Medical University.

Nude mice with similar body weights were randomly divided into two groups, five per group. The first group of mice was inoculated with A549 lung cancer cells in the right armpit as a control group; the second group of mice was inoculated with the same amount of A549 cells co-cultured with Exos in the right armpit. We observed the tumor formation of the mice. Four weeks later, the mice were killed by cervical dislocation, each tumor was stripped, and the organs and tissues were collected.

Each tumor was fixed, embedded, rendered transparent, dehydrated, and sectioned for hematoxylin and eosin section staining to observe the tumor tissue morphology. The section was dripped with 3% citric acid freshly diluted pepsin, rinsed with PBS, dripped with pre-hybridization and hybridization solution, and washed with SSC. We deparaffinized and hydrated the sections, then sealed them with antigen retrieval serum and incubated with primary and secondary antibodies. After color development, differentiation, dehydration, and transparency, the expression of cell proliferation markers PCNA and KI67 was observed. We treated each tissue and organ with Trizol, extracted tissue RNA, and used a reverse transcription kit to synthesize complementary DNA, with fluorescence quantitative PCR kit detection. After specific primer denaturation, annealing, and extension to detect changes in gene levels of tissue cell proliferation markers PCNA and KI67, we further verified the PCNA and KI67 protein level expression changes results by WB. The steps used were the same as those in the above WB procedure. These experiments were replicated three times.

### Statistical analysis

All statistical analyses were performed using GraphPad Prism (version 8.0; GraphPad Software, San Diego, CA). Data are presented using mean ± standard deviation values. The comparisons between two groups were analyzed using Student’s *t* test. The comparisons among multiple groups were detected by one-way analysis of variance and confirmed by Dunnett’s multiple comparisons test. *P* < 0.05 was considered to indicate a significant difference.

## Results

### Polarization and identification of M1 macrophages

**Figure 1:**
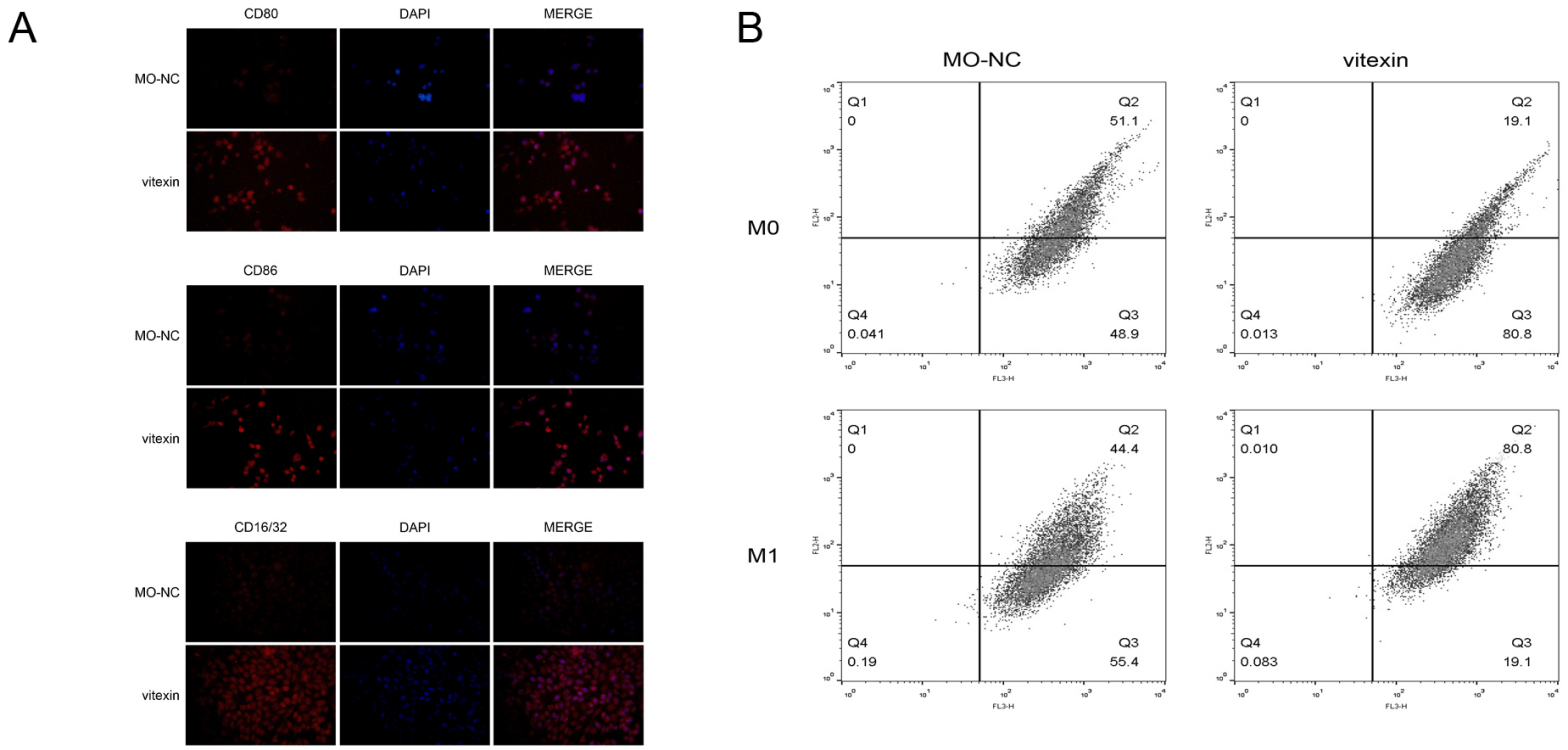
Polarization and identification of M1 macrophages. (A) Immunofluorescence test determined M1-type macrophages using M1 macrophage surface markers CD80, CD86, and CD16/32. (B) Flow cytometry detection of M0 macrophage polarization results showed that M0 macrophages were polarized into M1 macrophages under the induction of drug stimulation.

The phenotype of M1 macrophages was determined by immunofluorescence detection of special antibodies of M1 macrophage surface marker CD16 (Fig. 1A). Further, flow cytometry detection of M0 macrophage polarization results showed that M0 macrophages were polarized into M1 macrophages under the induction of drug stimulation (Fig. 1B).

### Effect of M1 macrophages on the migration and invasion of lung cancer cells

**Figure 2:**
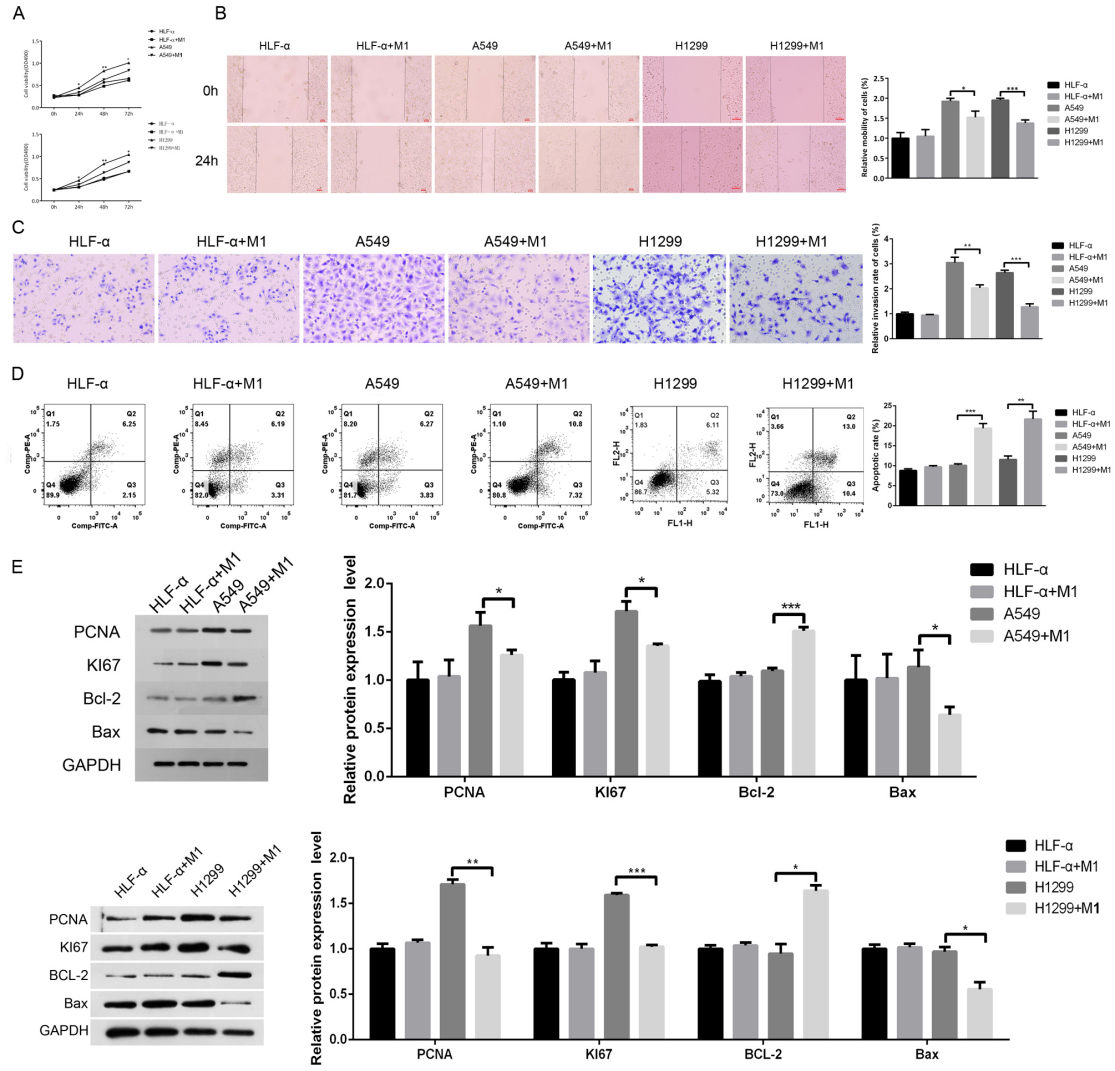
Effect of M1 macrophages on the migration and invasion of lung cancer cells. The effect of M1 macrophages on the migration and invasion of lung cancer cells. MTT (A), scratch (B), and Transwell (C) assays were used to detect the cell viability, migration, and invasion abilities of the four groups. (D) Flow cytometry was used to detect the apoptosis of each group. (E) Western blot was used to detect the proliferation-related protein of PCNA, KI67, Bax, and Bcl-2 expression. * *P* < 0.05, ** *P* < 0.01).

Compared to the Cancer group, the cell viability of the Cancer+M1 group was weakened (Fig. 2A). The scratch test showed that, compared to the Cancer group, the relative migration rate of the cells in the Cancer+M1 group decreased significantly (Fig. 2B). Transwell assay showed that the number of lung cancer cells that passed through Matrigel within 24 h was decreased significantly after co-culture with M1 macrophages (Fig. 2C); further, the number of intersecting cells was significantly reduced, with a significant difference (*P* < 0.01). Flow cytometry results showed that lung cancer cells experienced increased apoptosis after co-culture with M1 macrophages (Fig. 2D). The expression of the apoptotic protein Bcl-2 was significantly decreased, but the expression of the pro-apoptotic protein Bax was increased (Fig. 2E).

### Effect of M1 macrophage Exos on the migration and invasion of lung cancer cells

**Figure 3:**
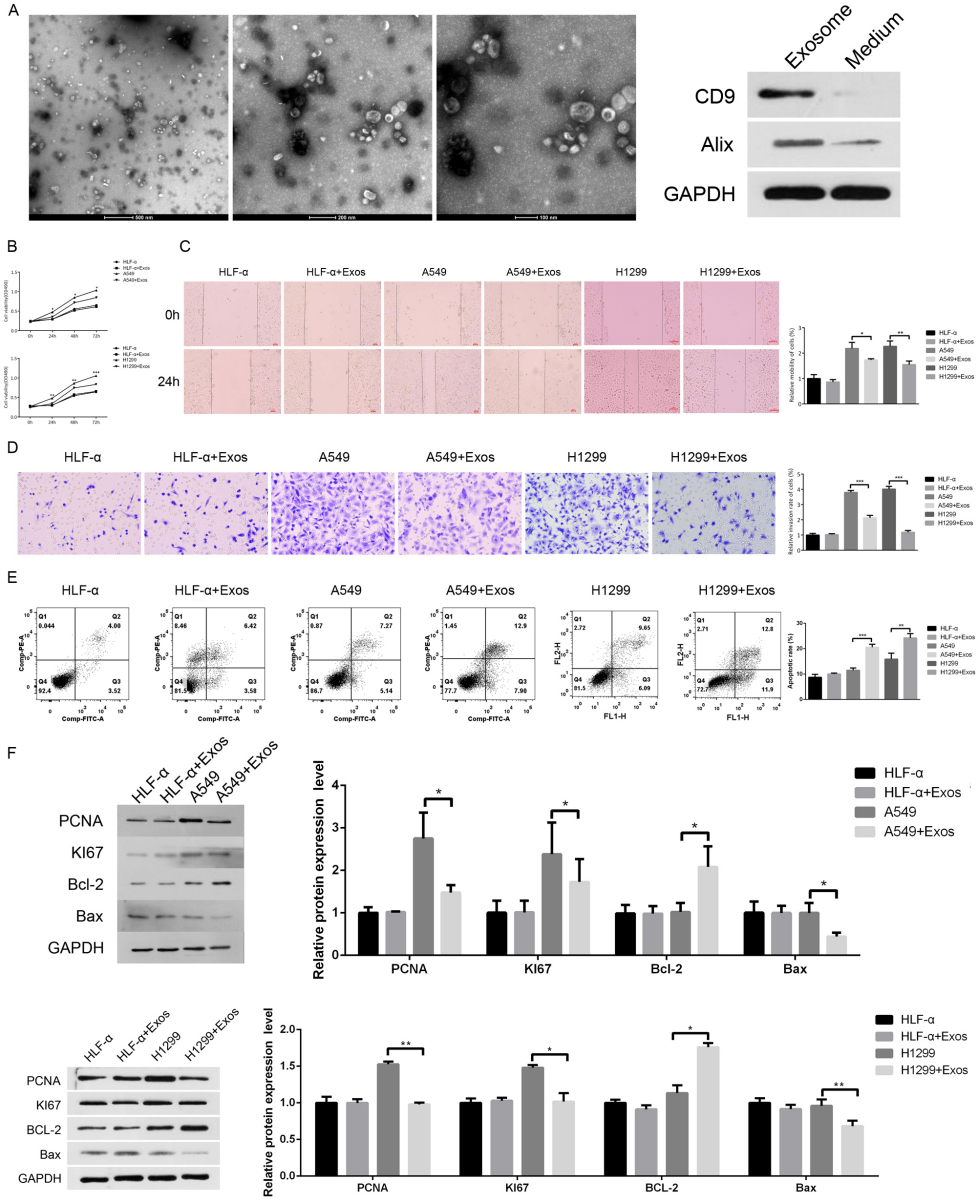
Effect of M1 macrophage Exos on the migration and invasion of lung cancer cells. (A) The typical structure of M1-Exos was clearly observed using transmission electron microscopy (left). The protein levels of exosome markers D9 and Alix were analyzed by western blot (right). The effect of M1-Exos on the migration and invasion of lung cancer cells. MTT (B), scratch (C) and Transwell (D) assays were used to detect the cell viability, migration, and invasion abilities of the four groups. (E) Flow cytometry was used to detect the apoptosis of each group. (F) Western blot was used to detect the proliferation-related protein of PCNA, KI67, Bax, and Bcl-2 expression. * *P* < 0.05, *** *P* < 0.001.

The typical structure of M1-Exos was clearly observed by electron microscopy (Fig. 3A, left). In addition, western blot was used to analyze the exosome-positive markers CD9 and Alix. (Fig. 3A, right). Compared to the A549 or H1299 group, the cell viability of the A549 or H1299-Exos group was attenuated (Fig. 3B). The scratch test showed that the relative mobility of the cells in the A549 or H1299-Exos group decreased after 48 h of co-culture compared to that in the A549 or H1299 group (the lower graph) (Fig. 3C). Transwell assay showed that the number of lung cancer cells that passed through Matrigel decreased within 24 h (Fig. 3D), and the difference was significant (*P* < 0.01). The results of flow cytometry showed that the percentage of apoptotic lung cancer cells (shown in the Q2 area) was significantly increased after adding Exos (Fig. 3E). WB protein level detection further verified that the expression levels of lung cancer cell proliferation–related proteins PCNA and KI67 and anti-apoptotic protein Bcl-2 were significantly decreased after adding Exos, but the expression of pro-apoptotic protein Bax was increased (Fig. 3F).

### Effect of miRNA-let-7b-5p on the migration and invasion of lung cancer cells

**Figure 4:**
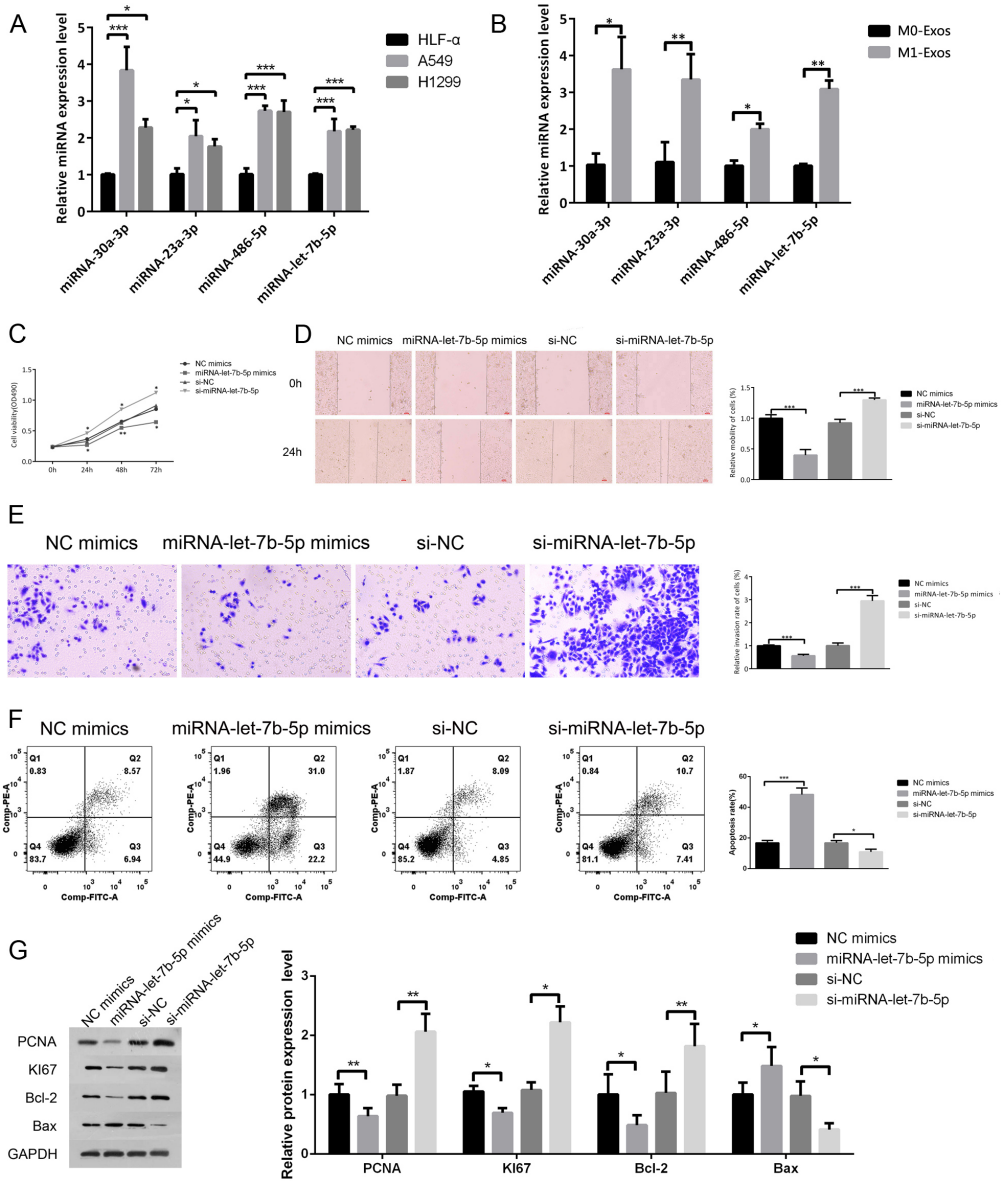
Effect of miRNA-let-7b-5p on the migration and invasion of lung cancer cells. (A) RT-PCR was used to detect expression levels of miRNA-23a-3p, miRNA-486-5p, miRNA-30a-3p, and miRNA-let-7b-5p in the HLF-A, A549 and H1299 cell groups, all of which were significantly lower in lung cancer cells. (B) Compared to M0-Exos, the corresponding four miRNAs abnormalities were found in M1-Exos with high expression. MTT (C), scratch (D), and Transwell (E) assays were used to detect the cell viability, migration, and invasion abilities of the four groups. (F) Flow cytometry was used to detect the apoptosis of each group. (G) Western blot was used to detect the proliferation-related protein of PCNA, KI67, Bax, and Bcl-2 expression. * *P* < 0.05, ** *P* < 0.01, *** *P* < 0.001.

We searched for abnormally expressed miRNAs in lung cancer by screening the literature and extracted total RNA from lung cancer cells and M1 macrophages Exos, verifying the results by qPCR. The screening of this test verified miRNA-23a-3p, miRNA-486-5p, miRNA-30a-3p and miRNA-let-7b-5p, all of which were expressed at significantly higher levels in the lung cancer cells (Fig. 4A). Compared to M0-Exos, high expression levels of the corresponding four miRNAs abnormalities were found in M1 Exos (Fig. 4B). We selected the differential gene miRNA-let-7b-5p to further explore its mechanism in lung cancer.

By constructing miRNA-let-7b-5p overexpression and silencing models of lung cancer cells, MTT was used to detect cell viability in each group at 24, 48, and 72 h. The results showed that, compared to the control group, the cell viability of the miRNA-let-7b-5p expression model was significantly increased by interference; meanwhile, the viability of the overexpression model was decreased significantly (*P* < 0.01) (Fig. 4C). The scratch test showed that the relative migration rate of cells in the overexpression group decreased significantly at 48 h, and the relative migration distance within 48 h in the interference model was significantly increased, which was statistically significant (*P* < 0.001) (Fig. 4D). Using Transwell assay to test the cell invasion ability, it was determined that, compared to the control group, the number of invaded cells in the overexpression model was halved and the invasion activity was suppressed, while, in the interference model, the number of cells that invaded the lower chamber at 24 h was increased by about 3-fold, and the invasion ability was significantly enhanced (Fig. 4E). Flow cytometric detection of cell apoptosis in each group showed that the rate of lung cancer cell apoptosis in the overexpression group was significantly increased; however, interference with its gene expression could reverse the results. In the interference model, the rate of apoptosis was reduced compared to that in the control group (Fig. 4F). Finally, the expression levels of proliferation-related proteins PCNA and KI67 and anti-apoptotic protein Bcl-2 were increased with the decrease of miRNA-let-7b-5p gene expression, while the expression of pro-apoptotic genes decreased with its silencing (Fig. 4G).

### Dual luciferase report verifies miRNA-let-7b-5p targeting genes

**Figure 5:**
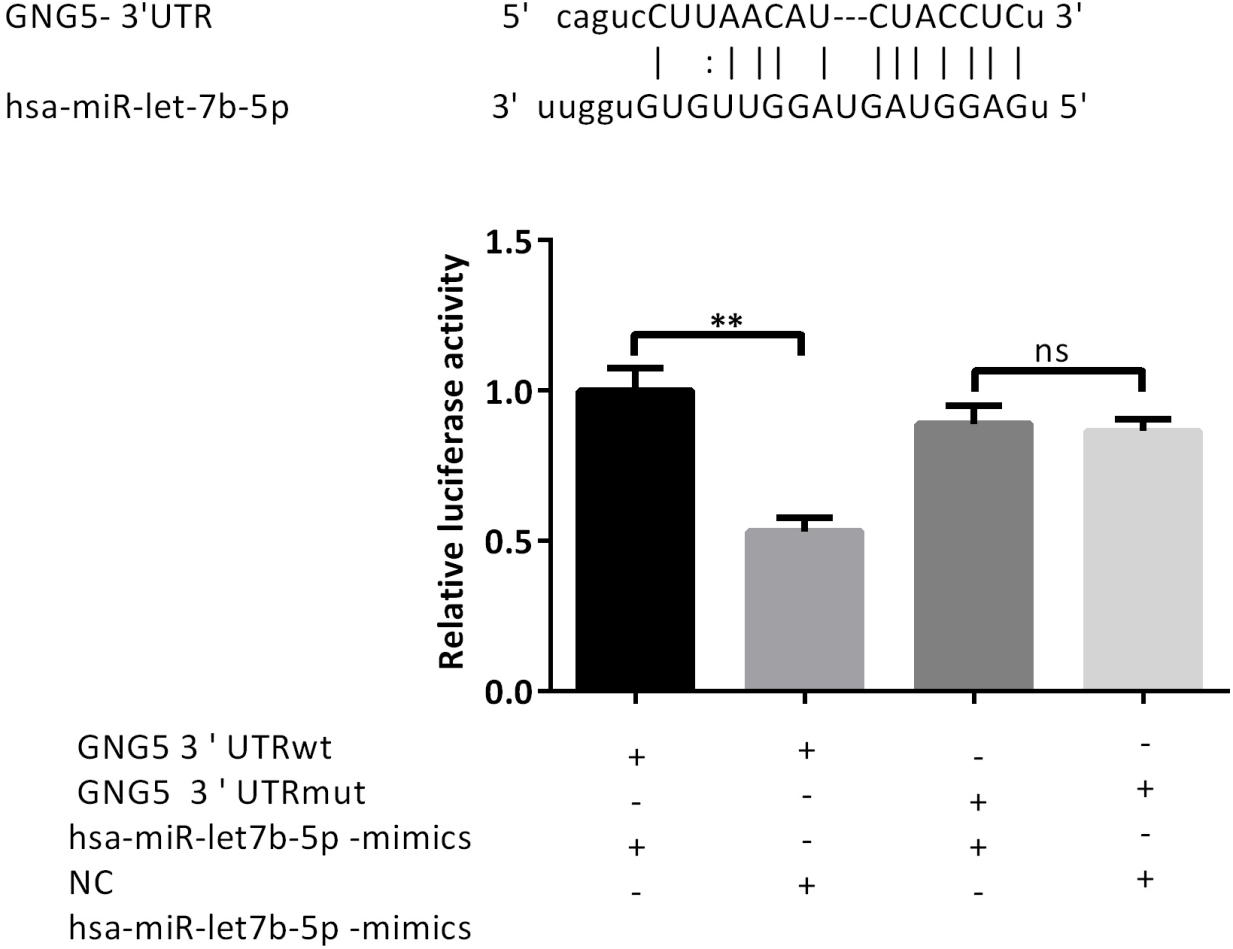
Dual luciferase report verifies miRNA-let-7b-5p targeting genes. Luciferase reporter analysis was performed to detect the bindings between miRNA-let7b-5p and GNG5 in A549 cells. ** *P* < 0.01.

To explore the targeting effect, we used the following test groups: miR-let-7b-5p mimics+GNG5-UTR-WT, mimics NC+GNG5-UTR-WT, miR-let-7b-5p mimics+GNG5-mut, and mimics NC+GNG5-UTR-mut. The results of the dual fluorescein report showed that the fluorescence intensity of the miR-let-7b-5p mimics+GNG5-UTR-WT group was decreased, while that of the miR-let-7b-5p mimics+GNG5-mut group did not change significantly (Fig. 5). They also revealed that luciferase-GNG5-3′ UTR can bind to miR-let-7b-5p, verifying that GNG5 is a target gene of miRNA-let-7b-5p.

### Target gene GNG5 axis regulates the migration and invasion of lung cancer cells

**Figure 6:**
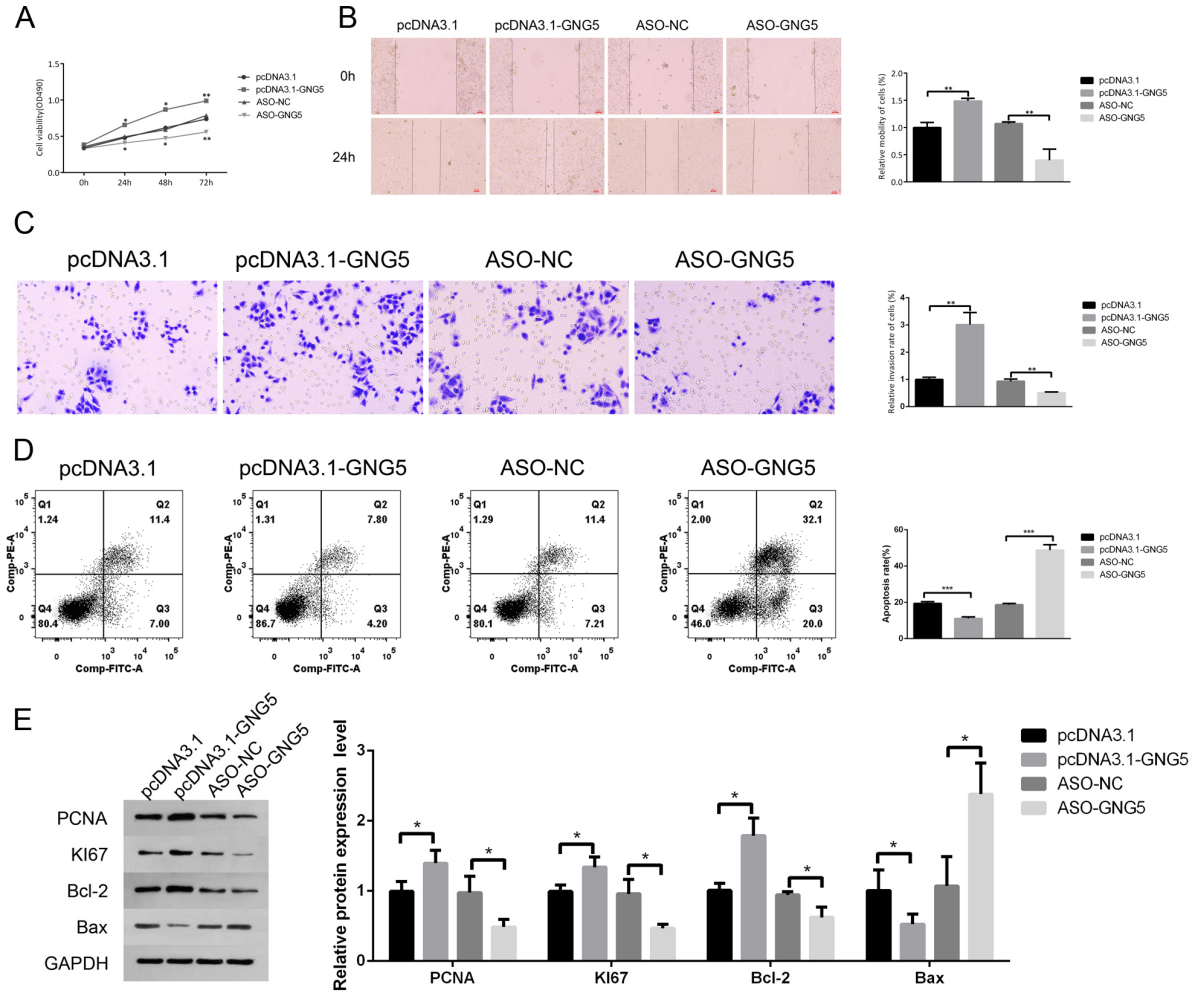
The target gene GNG5 axis regulates the migration and invasion of lung cancer cells. MTT (A), scratch (B), and Transwell (C) assays were used to detect the cell viability, migration, and invasion abilities of the four groups. (D) Flow cytometry was used to detect the apoptosis of each group. € Western blot was used to detect the proliferation-related protein of PCNA, KI67, Bax, and Bcl-2 expression. * *P* < 0.05, ** *P* < 0.01.

We constructed GNG5 overexpression and silence models and set up a control group. MTT results showed that cell viability in the overexpression model was significantly increased within 24, 48, and 72 h, while the growth of A549 cells in the silent models was inhibited (Fig. 6A). In the scratch test, the relative migration rate of the overexpression model A549 cells within 48 h was increased by about 50% relative to the control group, while the migration rate was significantly inhibited in the silent model (Fig. 6B). According to the Transwell assay, the overexpression model had the largest number of cells invading the lower chamber within 24 h, and the corresponding silent model group had the smallest number of cells in the lower chamber, with obvious differences (Fig. 6C). Flow cytometry detection of apoptosis in each group revealed that the number of A549 cells with apoptosis in the overexpression model was significantly reduced, while the apoptosis rate of A549 cells was significantly increased in the silent model (Fig. 6D). WB test results showed that, with the increase of GNG5, the protein expression levels of proliferation-related proteins PCNA and KI67 and anti-apoptotic protein Bcl-2 were also increased, but the expression of pro-apoptotic protein Bax was downregulated (Fig. 6E).

### M1-Exos inhibit tumor cell growth in vivo

**Figure 7:**
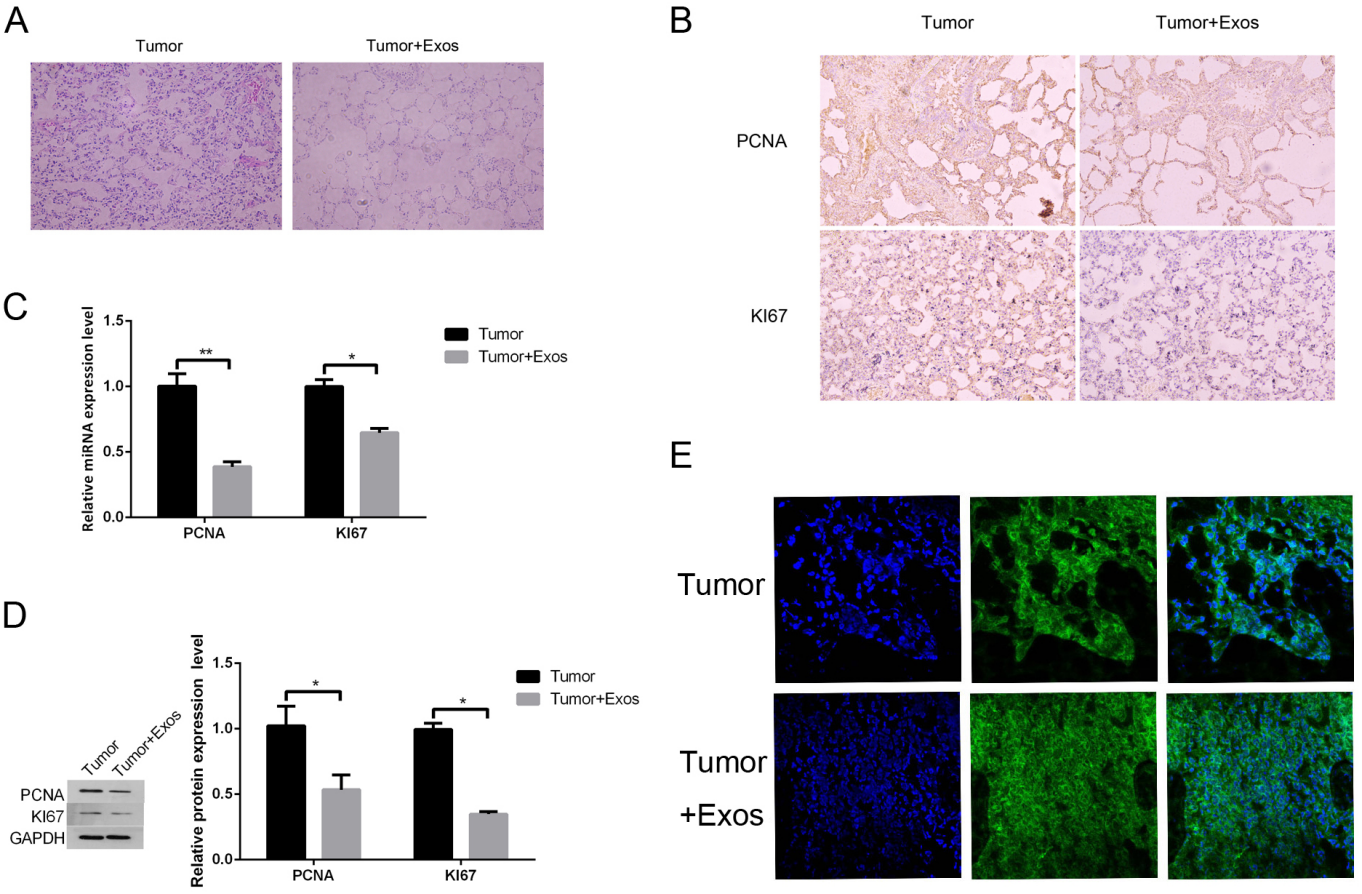
M1-Exos i nhibits tumor cell growth in vivo. (A) Observation of mouse tumors by hematoxylin and eosin staining. (B) The results of the immunohistochemical testing showed that, compared to the lung cancer group, the expression of proliferation marker proteins PCNA and KI67 in the Exos group was reduced. (C) qPCR showed that the levels of proliferation markers PCNA and KI67 genes were significantly reduced. (D) Western blot showed that the expression of PCNA and KI67 proteins in the exosomal group was inhibited. (E) The results of a FISH assay revealed the expression in tumor tissues. DAPI was used to stain nuclei (blue); green fluorescence was from the biotin fusions; right, the merged image. * *P* < 0.05, ** *P* < 0.01.

In the observation of mouse tumors by hematoxylin and eosin staining, the lung cancer+Exos group was compared to the lung cancer group, revealing that the tumor cell nuclei were smaller and the staining was lighter (Fig. 7A). Analysis showed that the Exos group had a high level of apoptosis, a large number of cells with shrinkage, and a low density of cells. In other words, Exos significantly inhibited the occurrence and development of lung cancer. Further FISH staining was conducted to detect the gene expression level of miRNA-let-7b-5p in each group of cells, and it was seen that the cells in the Exo group had strong fluorescent staining, indicating that Exos can inhibit lung cancer by upregulating the expression of miRNA-let-7b-5p (Fig. 7B). The results of immunohistochemical testing showed that, compared to the lung cancer group, the protein expression of the tissue cell proliferation markers PCNA and KI67 in the Exos group was reduced (Fig. 7C). Verification by qPCR revealed that the gene levels of proliferation markers PCNA and KI67 were significantly reduced (Fig. 7D). Finally, the results of WB verification showed that the expression of PCNA and KI67 proteins in the exosomal group was inhibited (Fig. 7E), and the results of immunohistochemistry were consistent.

## Discussion

Tumor-associated macrophages not only inhibit the cytotoxicity of T-cells but also secrete growth factors to nourish tumor growth and promote tumor metastasis. Zheng and other researchers have reported that gastric cancer tumor-related macrophage Exos reduce the prognostic effect of gastric cancer chemotherapy (Brown et al., 2009). M1 macrophages have a strong antigen presentation function; can secrete a large number of inflammatory factors, chemokines, growth factors, and matrix metalloproteinases; and participate in the anti-tumor response in a variety of ways. In the tumor microenvironment, tumor cells release Exos into the microenvironment, which carry a variety of biologically active substances and can change the biological functions of cells in the microenvironment (Zheng et al., 2017). Therefore, this experiment started with tumor-associated macrophage Exos as the entrance to explore their influence on the proliferation, invasion, and migration of lung cancer cells. An increasing number of studies are emerging that suggest the effects of Exos on tumor occurrence, development, proliferation, metastasis, and invasion, but the specific mechanisms remain to be further explored. This study used Exo-related differential genes to study the impact of lung cancer cell migration and invasion capabilities. It provided a certain reference basis for exploring the influence of TAM-derived Exos on tumor diseases, providing new ideas for the treatment of lung cancer diseases.

In this study, by polarizing M1 macrophages, extracting macrophage Exos, and co-culturing with A549 lung cancer cells, we explored the changes in A549 cell viability. MTT, scratch, and Transwell assay results support that A549 cell viability, proliferation, migration, and metastasis capacity after co-cultivation were lower than those of lung cancer cells that were not co-cultured, indicating that M1 macrophage Exos had an inhibitory effect on lung cancer cells, and it was found that the expression of proliferation proteins was reduced and the level of pro-apoptotic proteins was increased. In vivo mouse experiments verified that Exos inhibit the occurrence and development of lung cancer.

As a cancer biomarker, miRNA was also closely related to various aspects of normal physiology and pathology, such as the occurrence, proliferation, differentiation, and apoptosis of lung cancer cells. In recent years, miRNA has attracted social research attention  (Jiang et al., 2021; Zhang et al., 2022). Gou et al. (2020) found that inhibition of cardiomyocyte-derived Exo-miR-19a-3p promoted angiogenesis and improved cardiac function in mice with myocardial infarction by targeting HIF-1 *α*. Therefore, the delivery of miRNA by Exos is a new idea for the treatment of diseases. In this study, in the study of the effect of tumor-associated macrophage Exos on lung cancer function, we further explored the differential expression of miRNA and miRNA-let-7b-5p gene in lung cancer and examined the effect on lung cancer function. Based on the impact of GNG5 on lung cancer, we developed an in-depth understanding of the mechanism of Exos on lung cancer invasion and metastasis. This study provides a new theoretical basis for the clinical treatment of lung cancer. M1 macrophage Exos are expected to be used in the clinical treatment of lung cancer.

##  Supplemental Information

10.7717/peerj.14608/supp-1 Supplemental Information 1 Uncropped Gels/Blots

10.7717/peerj.14608/supp-2 Supplemental Information 2 Uncropped Micrographs Fig. 1 CD80

10.7717/peerj.14608/supp-3 Supplemental Information 3 Uncropped Micrographs Fig. 1 CD86

10.7717/peerj.14608/supp-4 Supplemental Information 4 Uncropped Micrographs Fig. 1 CD1632

10.7717/peerj.14608/supp-5 Supplemental Information 5 Uncropped Micrographs Fig. 2

10.7717/peerj.14608/supp-6 Supplemental Information 6 Uncropped Micrographs Fig. 3

10.7717/peerj.14608/supp-7 Supplemental Information 7 Uncropped Micrographs Figs. 4–2

10.7717/peerj.14608/supp-8 Supplemental Information 8 Uncropped Micrographs Fig. 6

10.7717/peerj.14608/supp-9 Supplemental Information 9 Uncropped Micrographs Fig. 7

10.7717/peerj.14608/supp-10 Supplemental Information 10 ARRIVE 2.0 Checklist

10.7717/peerj.14608/supp-11 Supplemental Information 11 Original data

## Abbreviations

Exos: exosomes
M1-Exos: M1 macrophage exosomes
NSCLC: non-small-cell lung cancer

## Additional Information and Declarations

### Data Availability

The following information was supplied regarding data availability: The raw data are available in the Supplemental Files.
