# Supplementary figures and images for: Exosomes derived from M1 macrophages inhibit the proliferation of the A549 and H1299 lung cancer cell lines via the miRNA-let-7b-5p-GNG5 axis (part 1 of 4)
Source: PeerJ. 2023 Jan 9;11:e14608. doi: 10.7717/peerj.14608 (PMC9835688; doi:10.7717/peerj.14608)

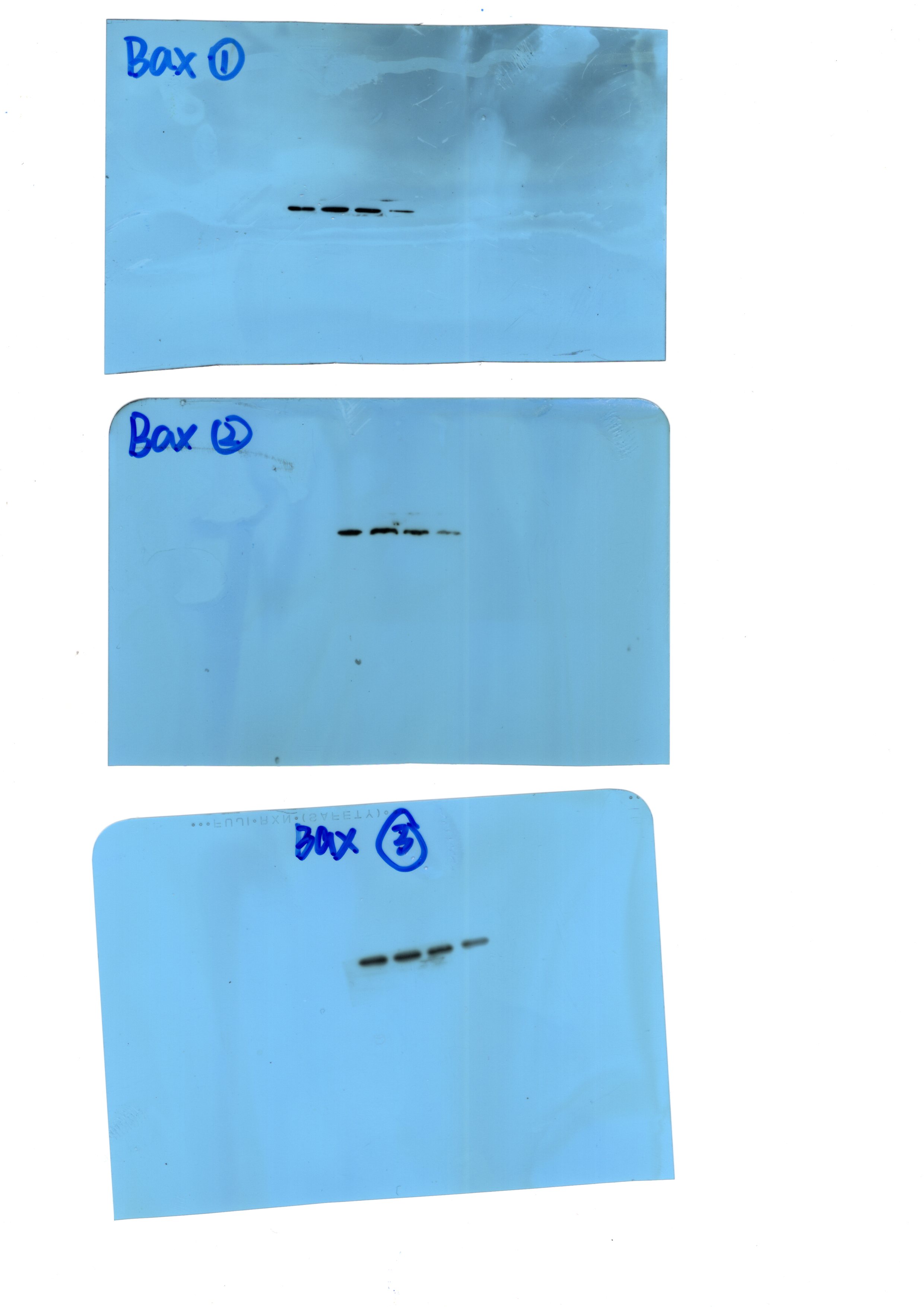

Supplement: Supplemental Information 1 [file peerj-11-14608-s001.zip › Western/Figure 4 WB/BAX orignal.jpg]

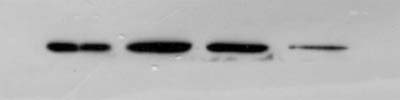

Supplement: Supplemental Information 1 [file peerj-11-14608-s001.zip › Western/Figure 4 WB/Baxú¿1ú⌐.jpg]

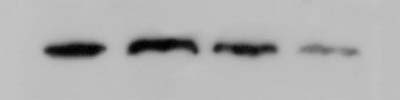

Supplement: Supplemental Information 1 [file peerj-11-14608-s001.zip › Western/Figure 4 WB/Baxú¿2ú⌐.jpg]

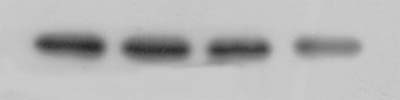

Supplement: Supplemental Information 1 [file peerj-11-14608-s001.zip › Western/Figure 4 WB/Baxú¿3ú⌐.jpg]

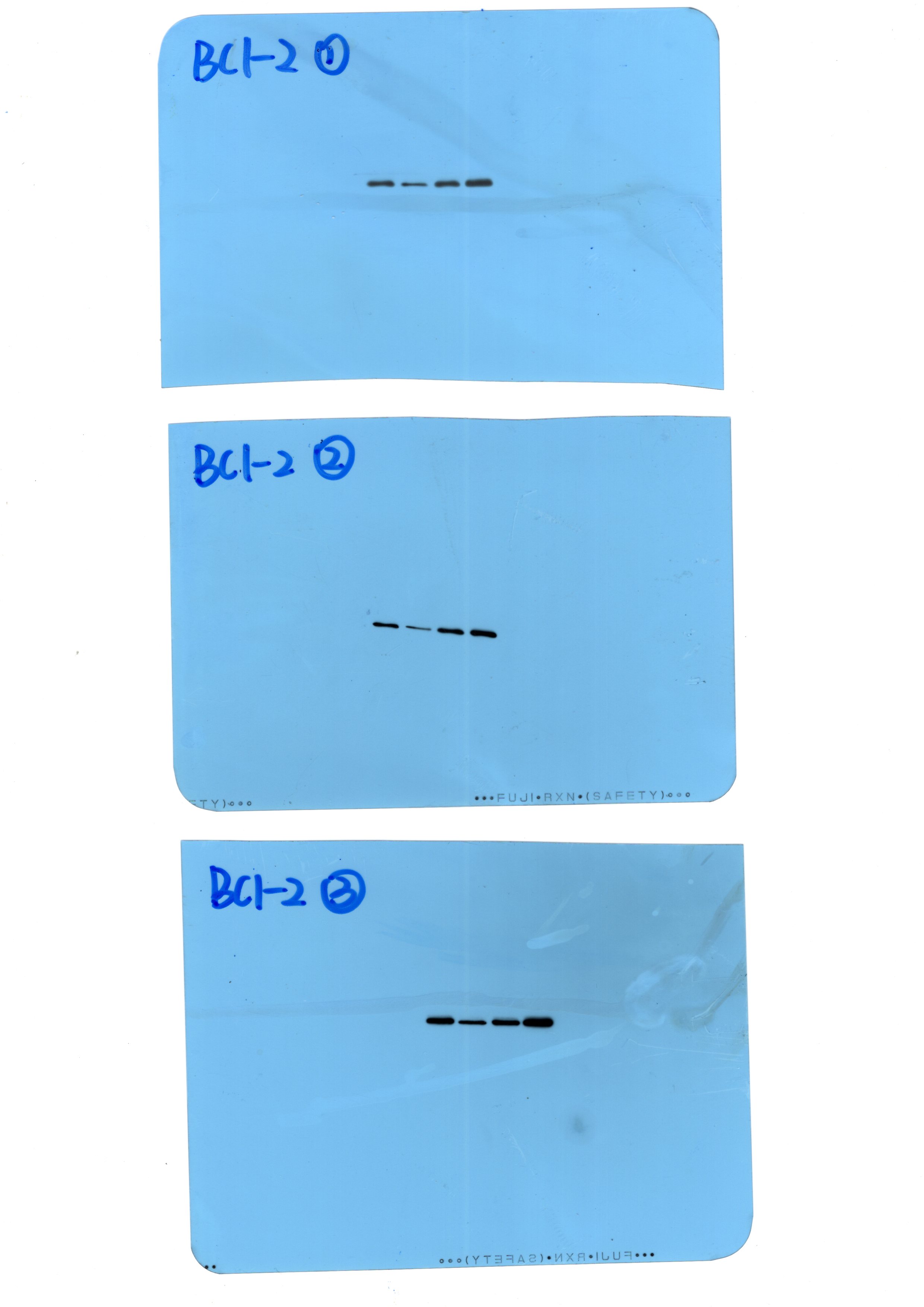

Supplement: Supplemental Information 1 [file peerj-11-14608-s001.zip › Western/Figure 4 WB/BCL-2 orignal.jpg]

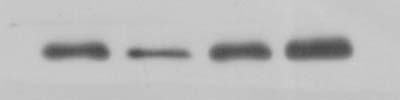

Supplement: Supplemental Information 1 [file peerj-11-14608-s001.zip › Western/Figure 4 WB/Bcl-2ú¿1ú⌐.jpg]

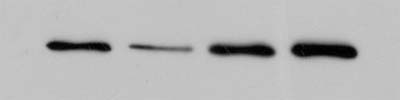

Supplement: Supplemental Information 1 [file peerj-11-14608-s001.zip › Western/Figure 4 WB/Bcl-2ú¿2ú⌐.jpg]

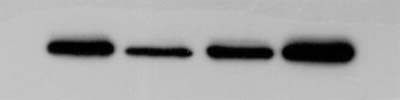

Supplement: Supplemental Information 1 [file peerj-11-14608-s001.zip › Western/Figure 4 WB/Bcl-2ú¿3ú⌐.jpg]

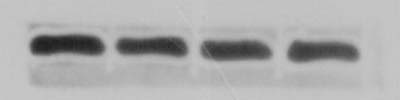

Supplement: Supplemental Information 1 [file peerj-11-14608-s001.zip › Western/Figure 4 WB/GADPH(1).jpg]

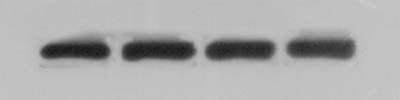

Supplement: Supplemental Information 1 [file peerj-11-14608-s001.zip › Western/Figure 4 WB/GADPH(2).jpg]

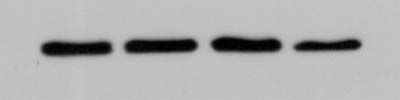

Supplement: Supplemental Information 1 [file peerj-11-14608-s001.zip › Western/Figure 4 WB/GADPH(3).jpg]

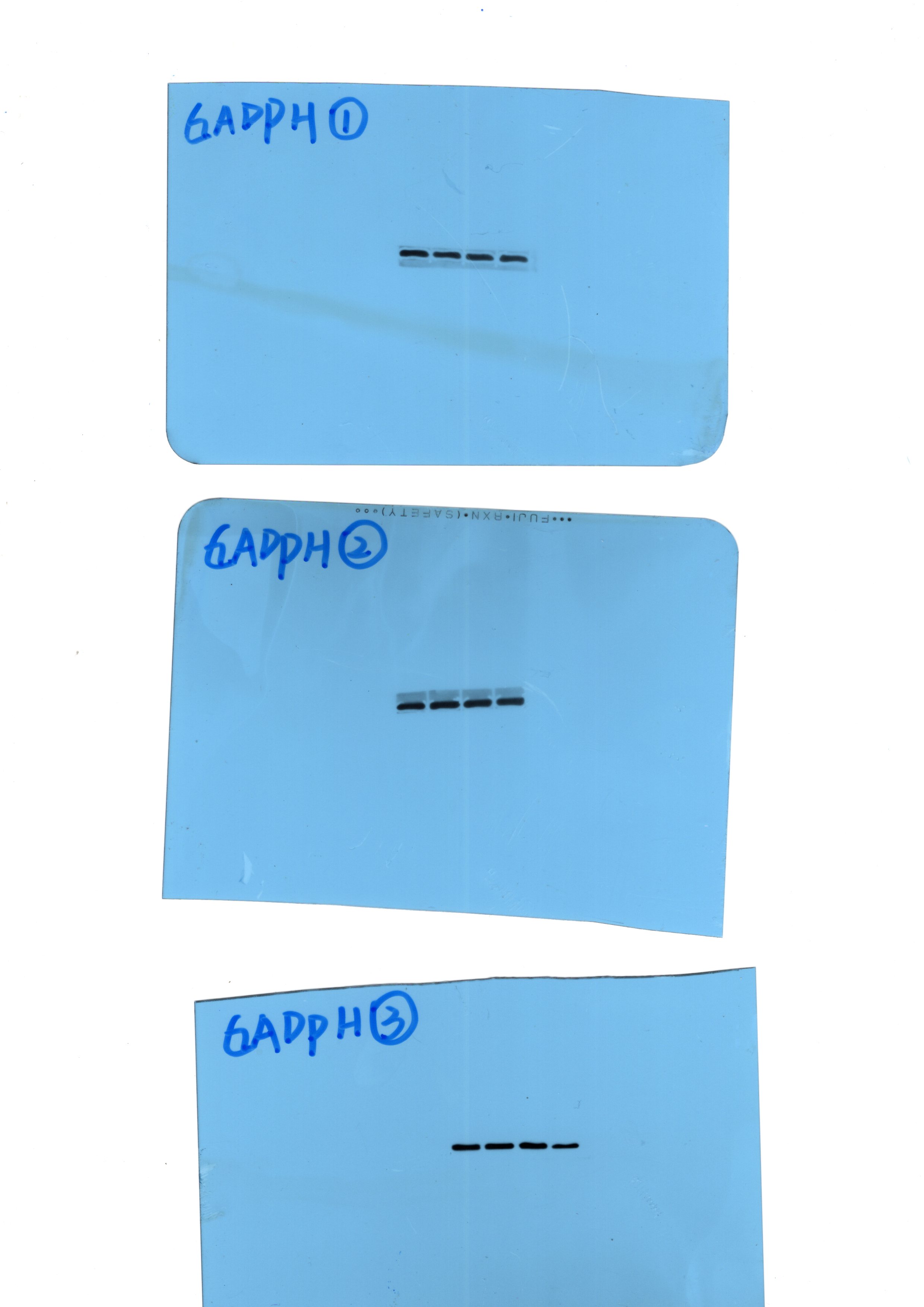

Supplement: Supplemental Information 1 [file peerj-11-14608-s001.zip › Western/Figure 4 WB/GAPDH orignal.jpg]

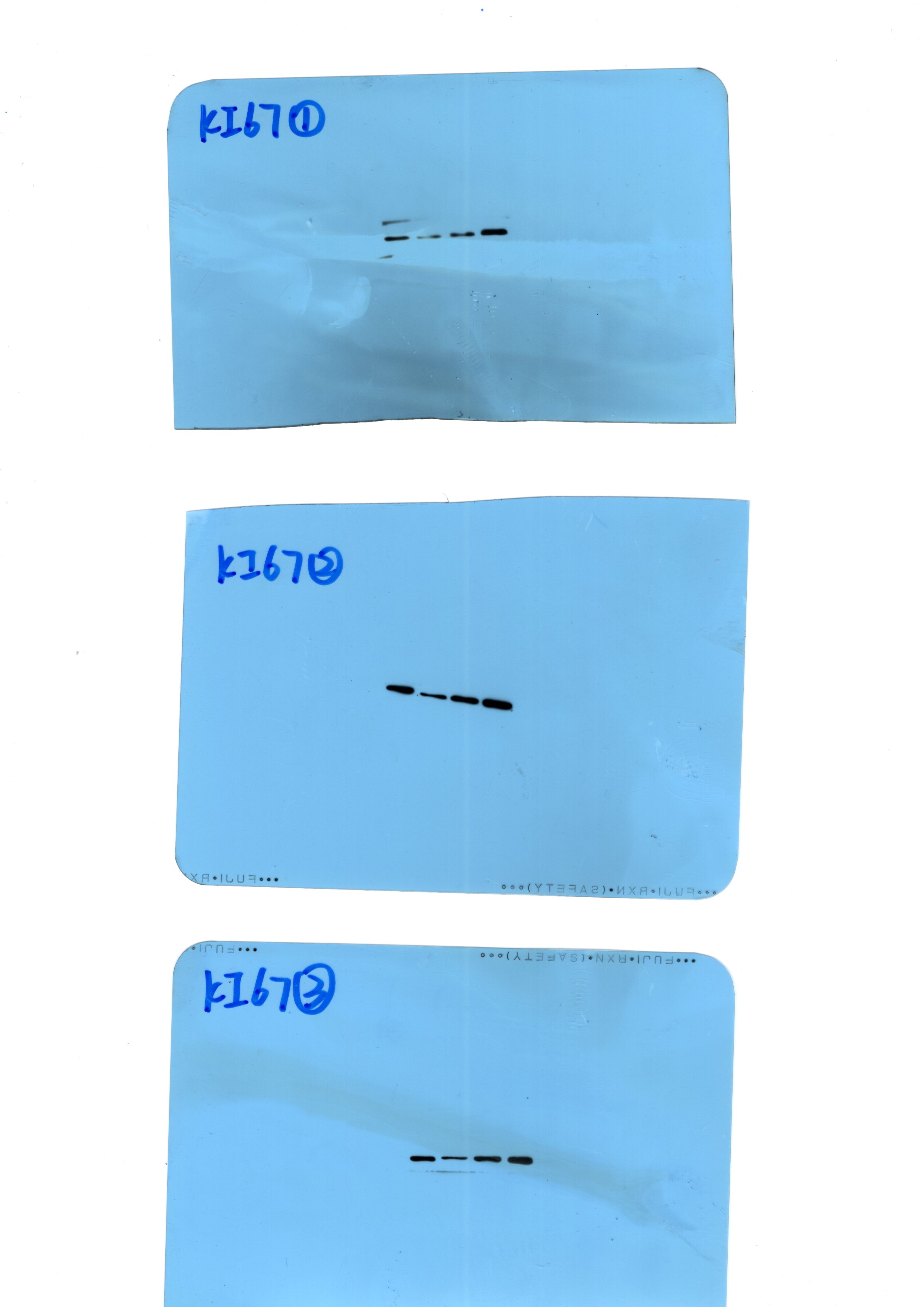

Supplement: Supplemental Information 1 [file peerj-11-14608-s001.zip › Western/Figure 4 WB/KI67 orignal.jpg]

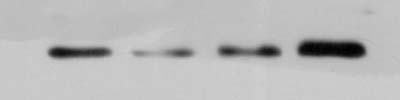

Supplement: Supplemental Information 1 [file peerj-11-14608-s001.zip › Western/Figure 4 WB/KI67(1).jpg]

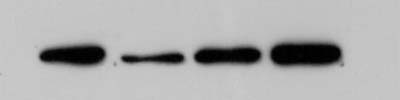

Supplement: Supplemental Information 1 [file peerj-11-14608-s001.zip › Western/Figure 4 WB/KI67(2).jpg]

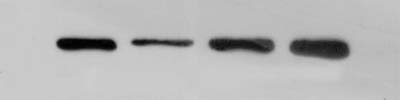

Supplement: Supplemental Information 1 [file peerj-11-14608-s001.zip › Western/Figure 4 WB/KI67(3).jpg]

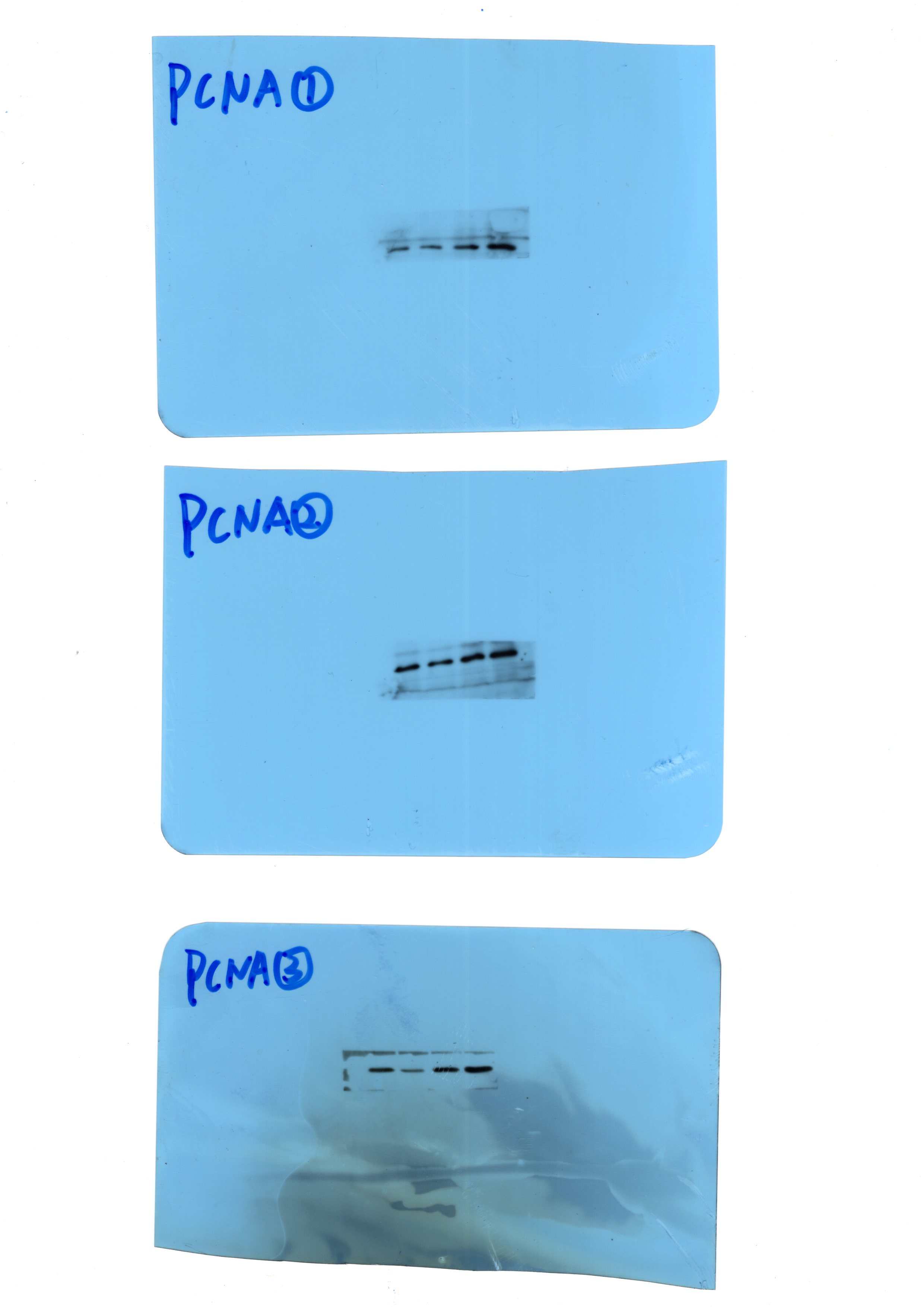

Supplement: Supplemental Information 1 [file peerj-11-14608-s001.zip › Western/Figure 4 WB/PCNA orignal.jpg]

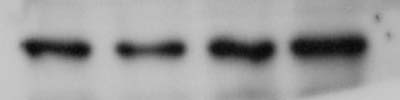

Supplement: Supplemental Information 1 [file peerj-11-14608-s001.zip › Western/Figure 4 WB/PCNA(1).jpg]

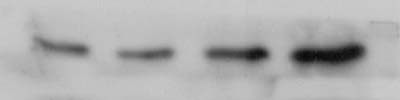

Supplement: Supplemental Information 1 [file peerj-11-14608-s001.zip › Western/Figure 4 WB/PCNA(2).jpg]

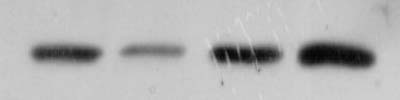

Supplement: Supplemental Information 1 [file peerj-11-14608-s001.zip › Western/Figure 4 WB/PCNA(3).jpg]

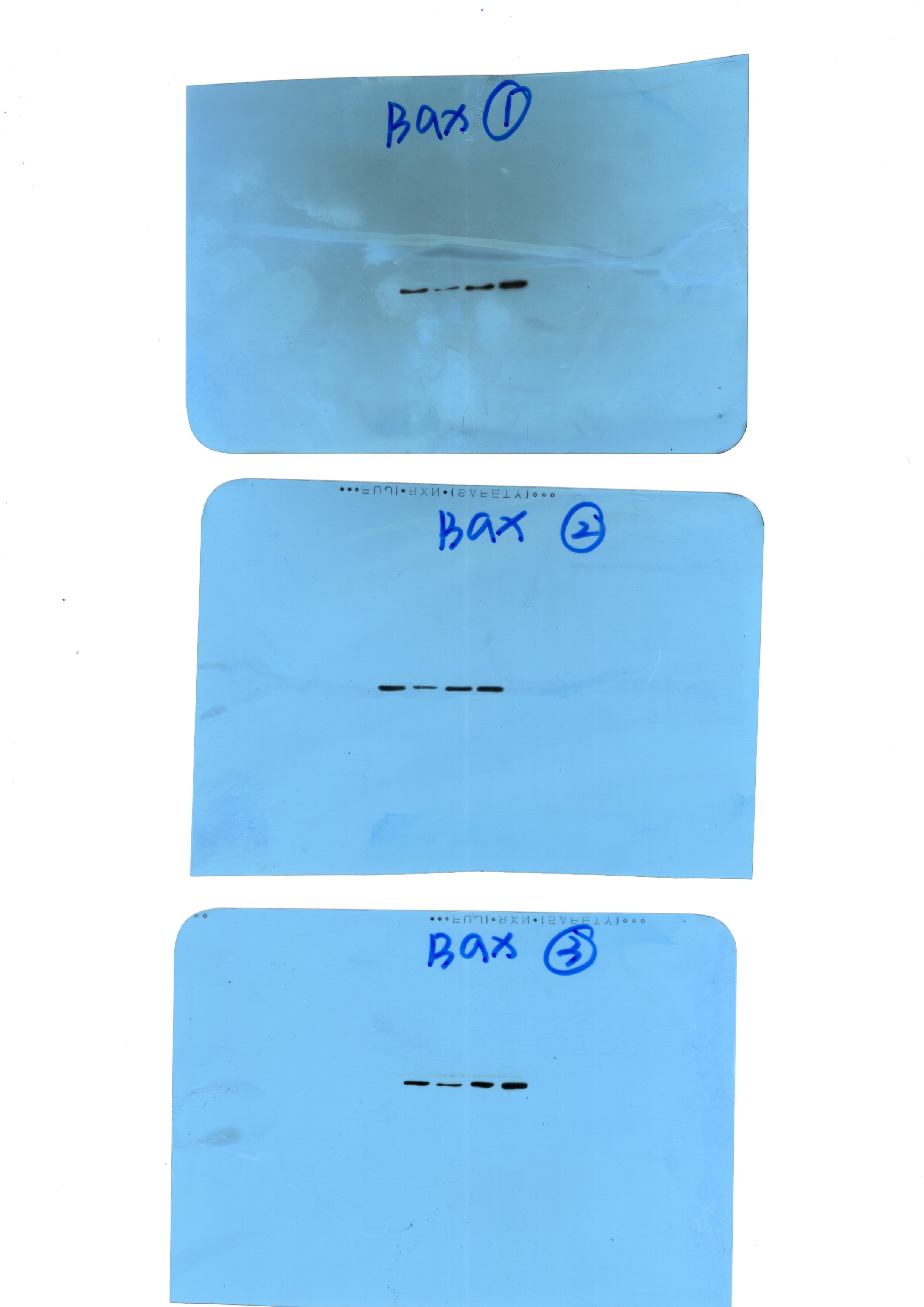

Supplement: Supplemental Information 1 [file peerj-11-14608-s001.zip › Western/Figure 6 WB/BAX orignal.jpg]

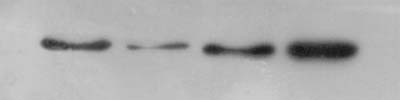

Supplement: Supplemental Information 1 [file peerj-11-14608-s001.zip › Western/Figure 6 WB/Bax(1).jpg]

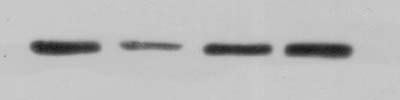

Supplement: Supplemental Information 1 [file peerj-11-14608-s001.zip › Western/Figure 6 WB/Bax(2).jpg]

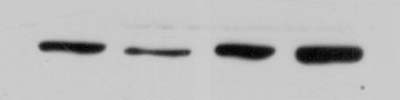

Supplement: Supplemental Information 1 [file peerj-11-14608-s001.zip › Western/Figure 6 WB/Bax(3).jpg]

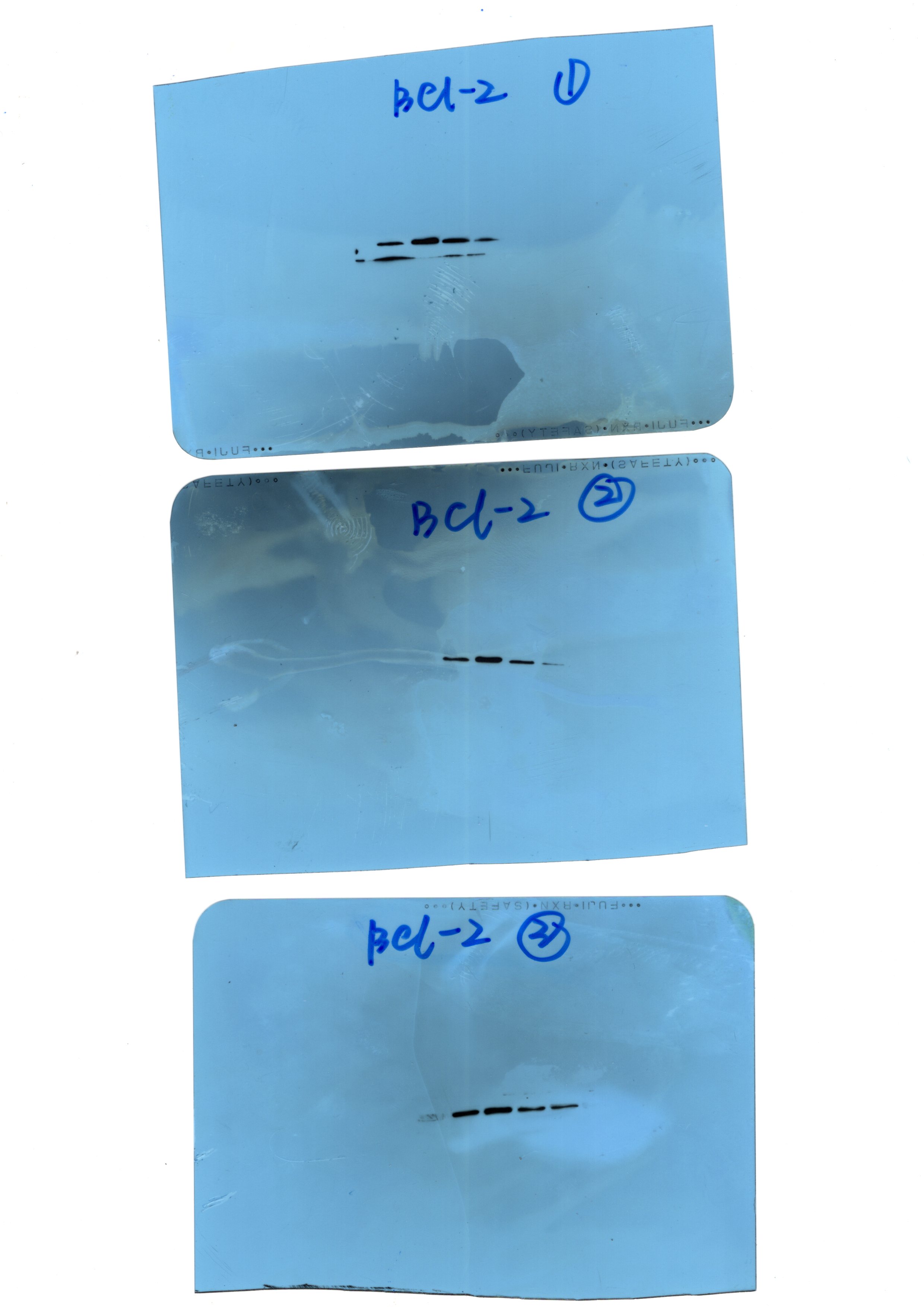

Supplement: Supplemental Information 1 [file peerj-11-14608-s001.zip › Western/Figure 6 WB/bcl-2 orignal.jpg]

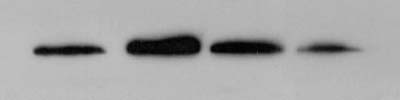

Supplement: Supplemental Information 1 [file peerj-11-14608-s001.zip › Western/Figure 6 WB/Bcl-2ú¿1ú⌐.jpg]

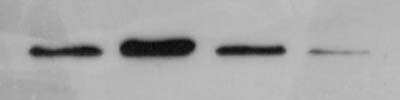

Supplement: Supplemental Information 1 [file peerj-11-14608-s001.zip › Western/Figure 6 WB/Bcl-2ú¿2ú⌐.jpg]

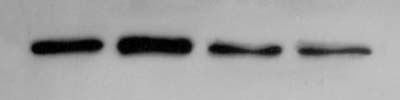

Supplement: Supplemental Information 1 [file peerj-11-14608-s001.zip › Western/Figure 6 WB/Bcl-2ú¿3ú⌐.jpg]

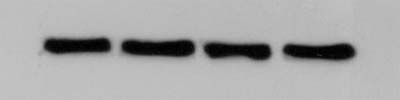

Supplement: Supplemental Information 1 [file peerj-11-14608-s001.zip › Western/Figure 6 WB/GADPH(1).jpg]

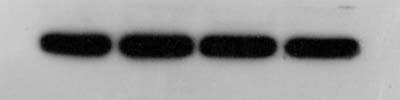

Supplement: Supplemental Information 1 [file peerj-11-14608-s001.zip › Western/Figure 6 WB/GADPH(2).jpg]

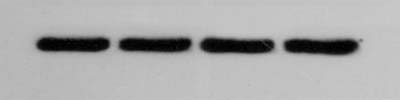

Supplement: Supplemental Information 1 [file peerj-11-14608-s001.zip › Western/Figure 6 WB/GADPH(3).jpg]

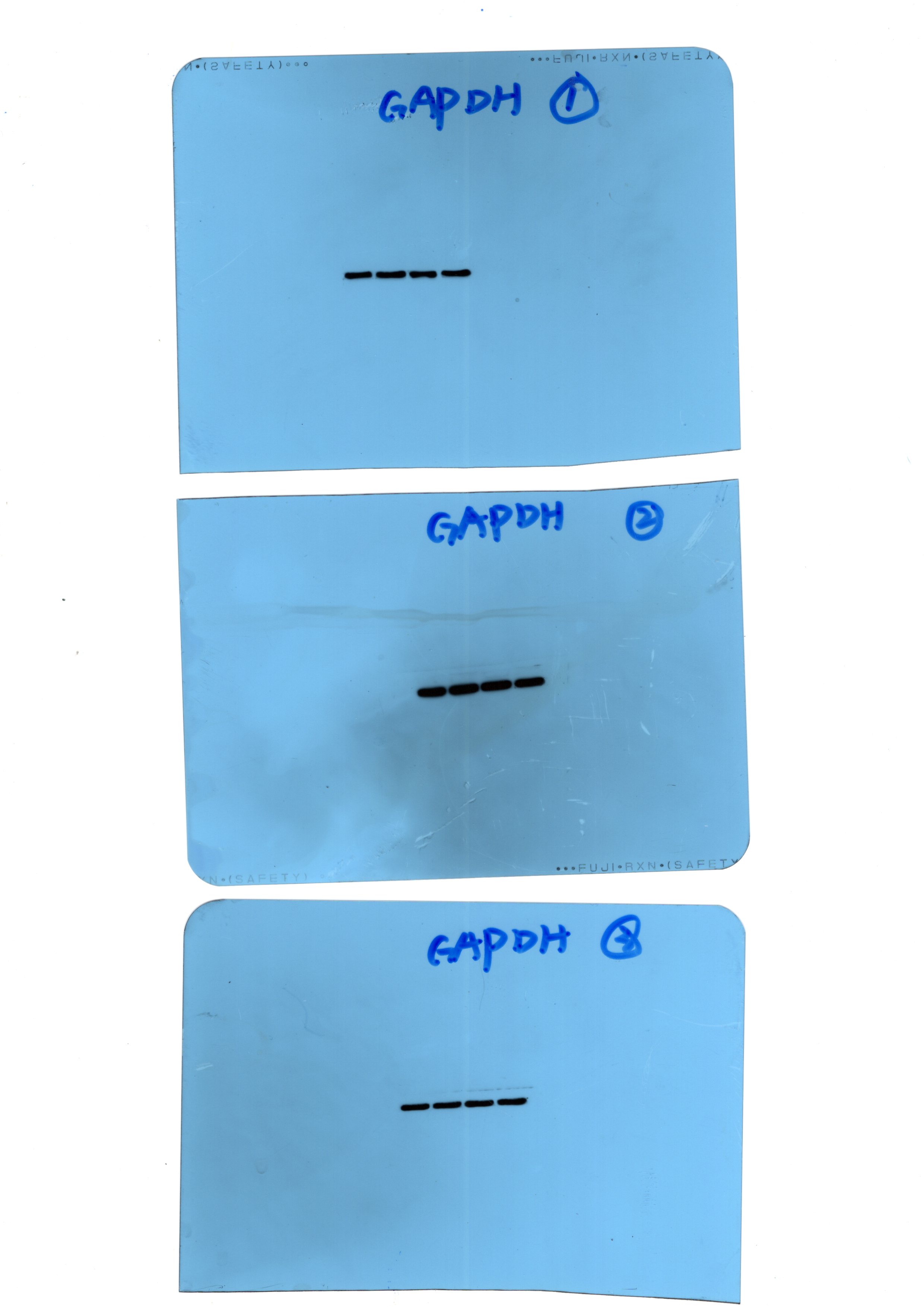

Supplement: Supplemental Information 1 [file peerj-11-14608-s001.zip › Western/Figure 6 WB/GAPDH orignal.jpg]

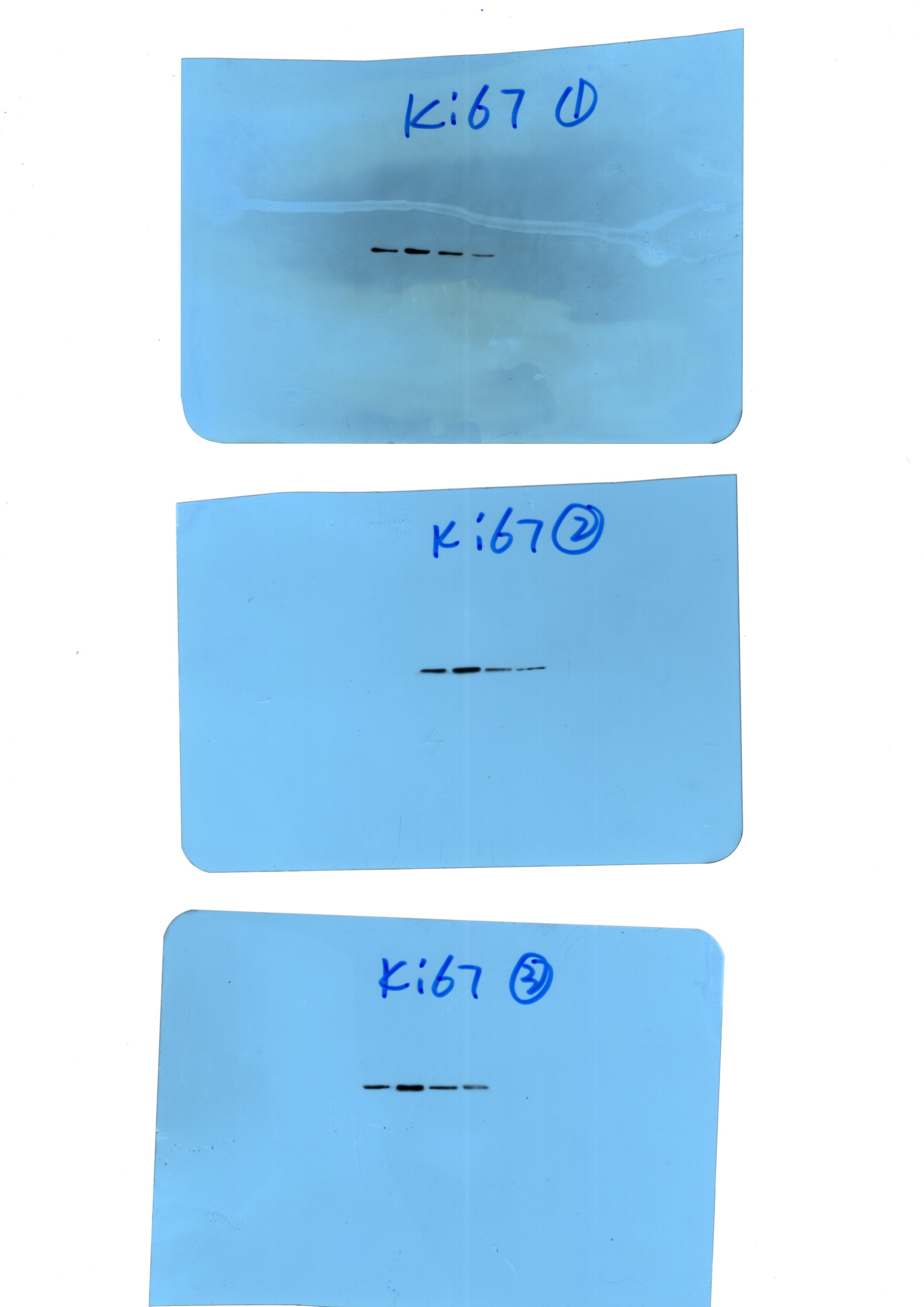

Supplement: Supplemental Information 1 [file peerj-11-14608-s001.zip › Western/Figure 6 WB/KI67 orignal.jpg]

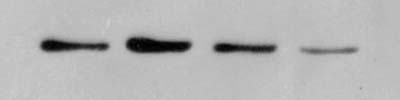

Supplement: Supplemental Information 1 [file peerj-11-14608-s001.zip › Western/Figure 6 WB/KI67(1).jpg]

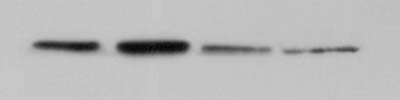

Supplement: Supplemental Information 1 [file peerj-11-14608-s001.zip › Western/Figure 6 WB/KI67(2).jpg]

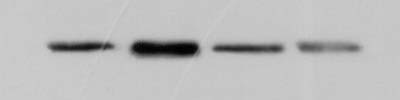

Supplement: Supplemental Information 1 [file peerj-11-14608-s001.zip › Western/Figure 6 WB/KI67(3).jpg]

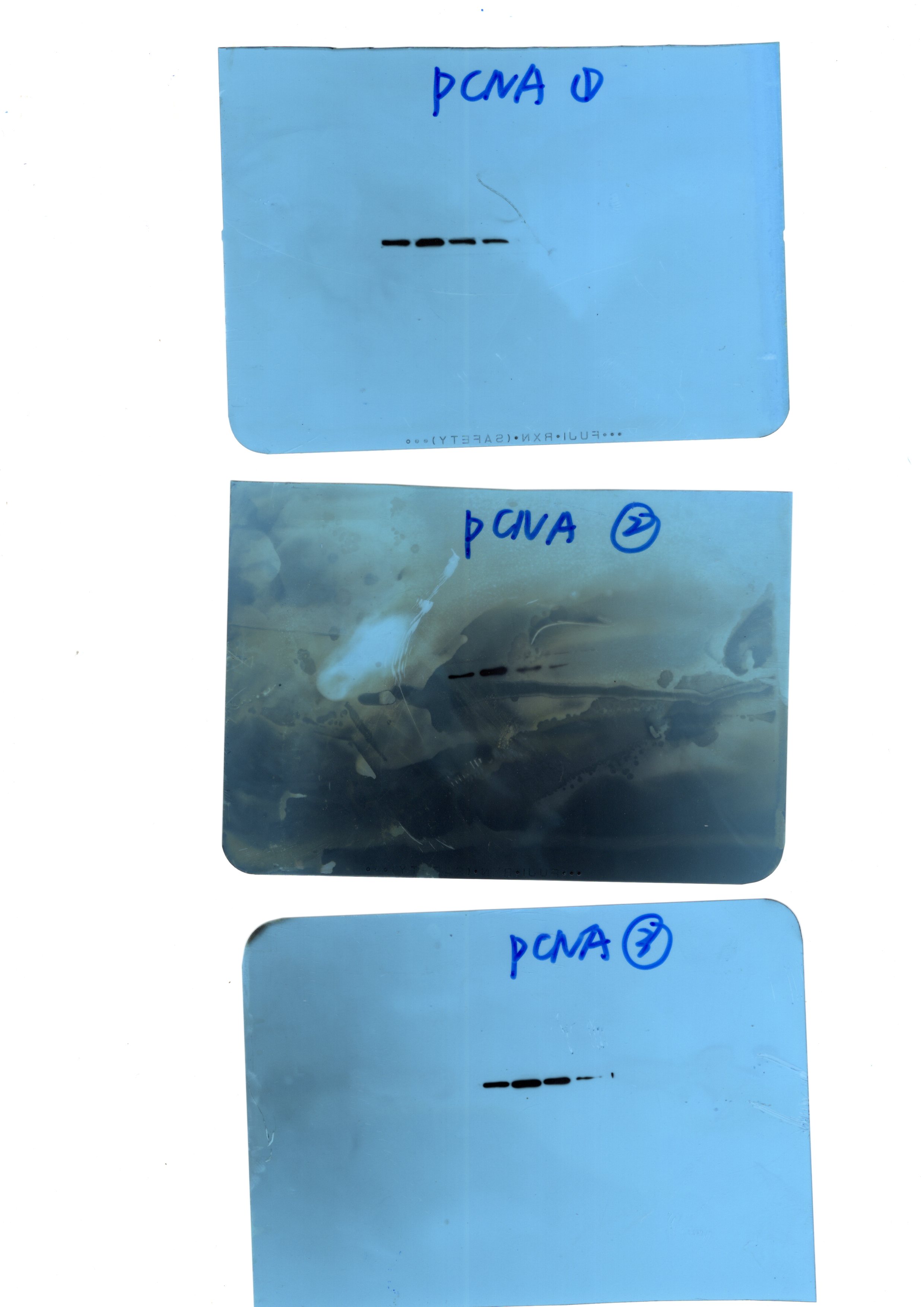

Supplement: Supplemental Information 1 [file peerj-11-14608-s001.zip › Western/Figure 6 WB/PCNA orignal.jpg]

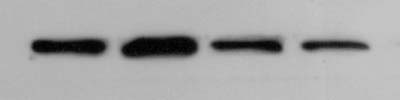

Supplement: Supplemental Information 1 [file peerj-11-14608-s001.zip › Western/Figure 6 WB/PCNA(1).jpg]

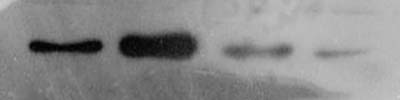

Supplement: Supplemental Information 1 [file peerj-11-14608-s001.zip › Western/Figure 6 WB/PCNA(2).jpg]

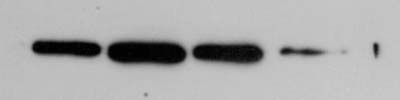

Supplement: Supplemental Information 1 [file peerj-11-14608-s001.zip › Western/Figure 6 WB/PCNA(3).jpg]

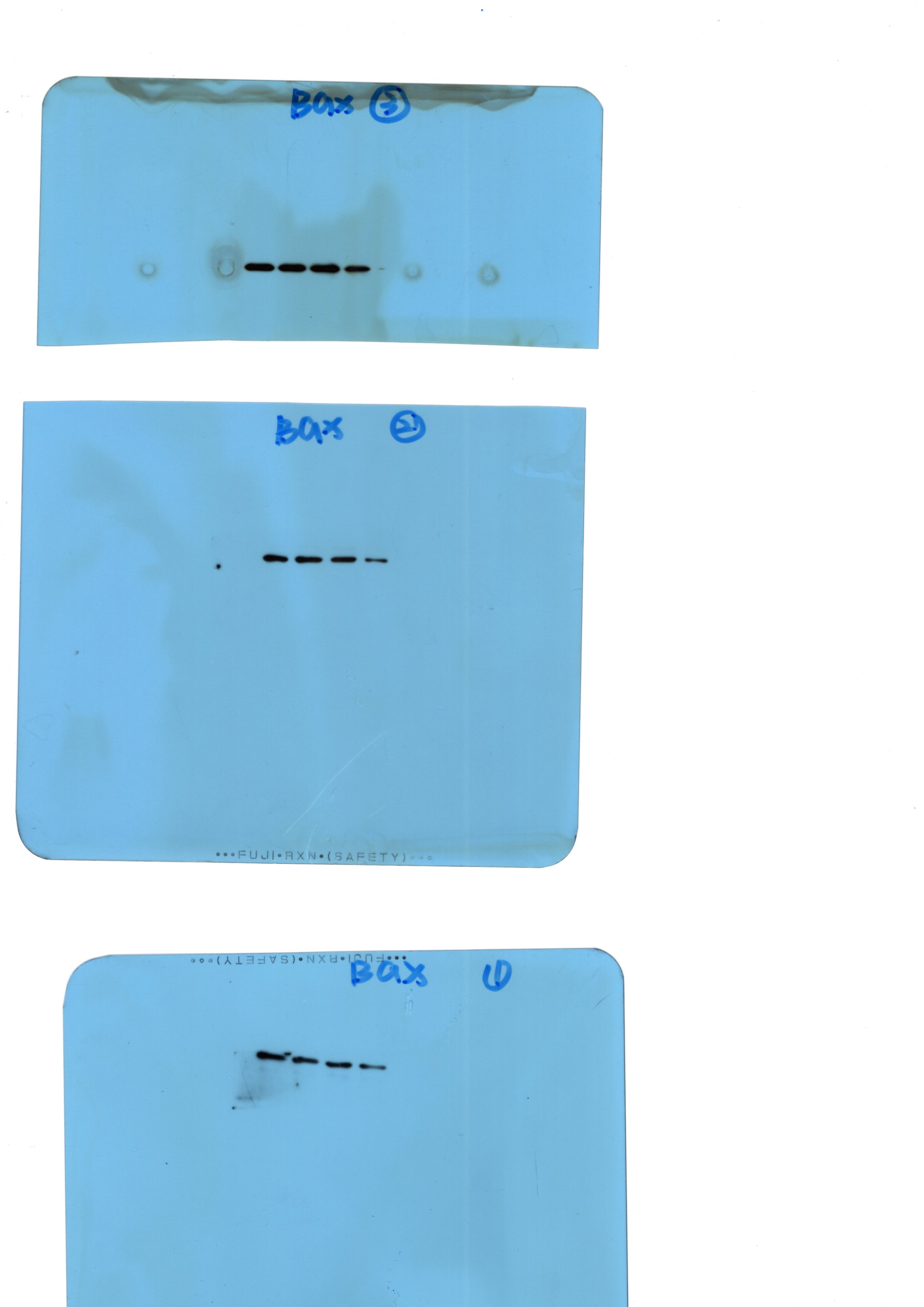

Supplement: Supplemental Information 1 [file peerj-11-14608-s001.zip › Western/Figure2 WB/BAX orignal.jpg]

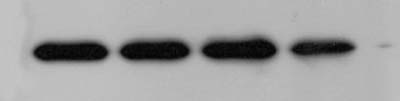

Supplement: Supplemental Information 1 [file peerj-11-14608-s001.zip › Western/Figure2 WB/Baxú¿1ú⌐.jpg]

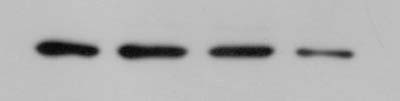

Supplement: Supplemental Information 1 [file peerj-11-14608-s001.zip › Western/Figure2 WB/Baxú¿2ú⌐.jpg]

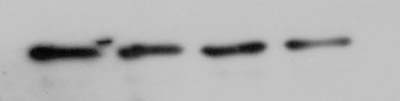

Supplement: Supplemental Information 1 [file peerj-11-14608-s001.zip › Western/Figure2 WB/Baxú¿3ú⌐.jpg]

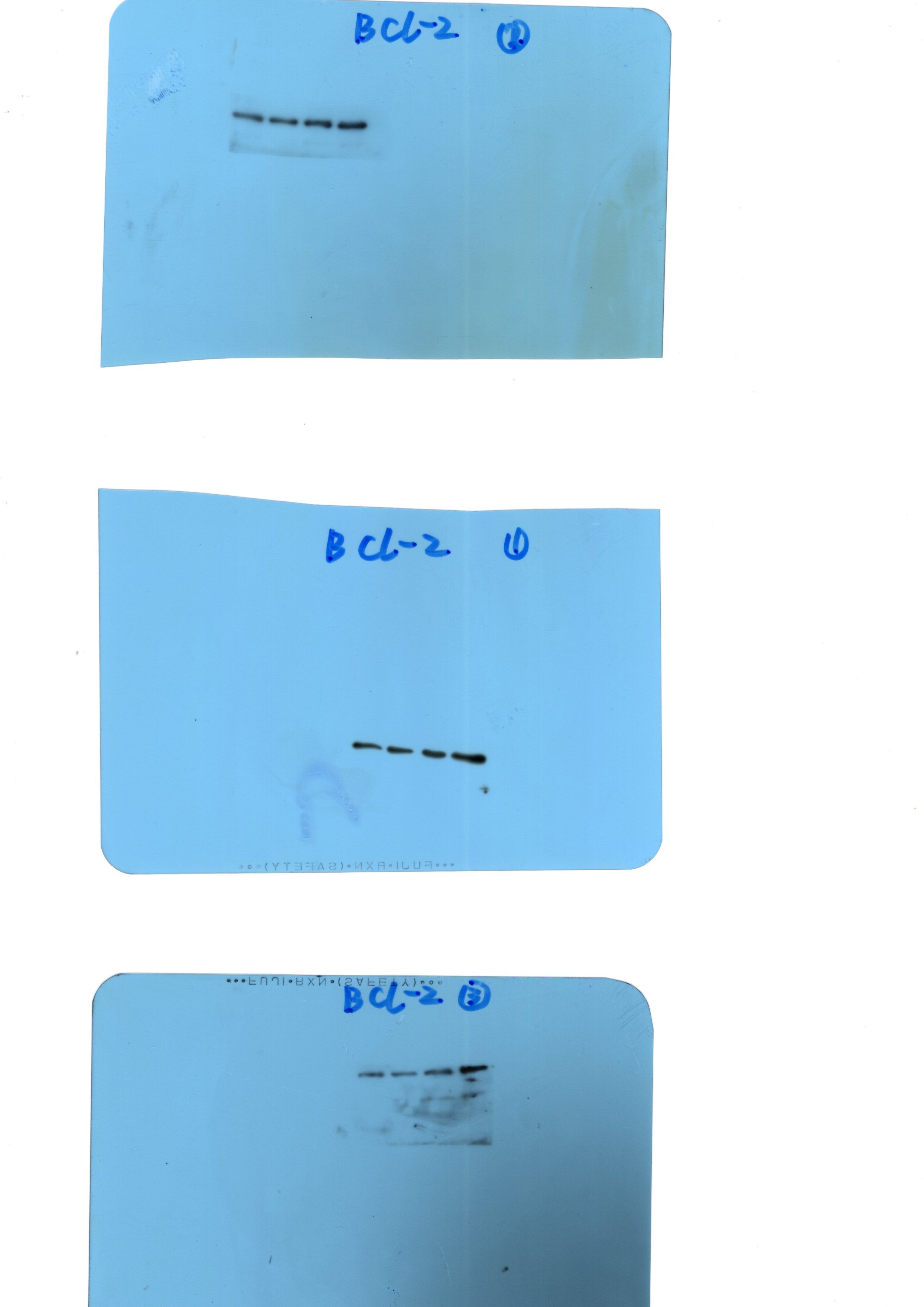

Supplement: Supplemental Information 1 [file peerj-11-14608-s001.zip › Western/Figure2 WB/BCL-2 orignal.jpg]

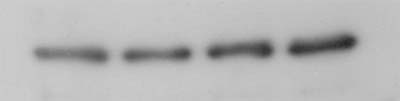

Supplement: Supplemental Information 1 [file peerj-11-14608-s001.zip › Western/Figure2 WB/BCL-2(1).jpg]

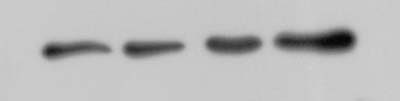

Supplement: Supplemental Information 1 [file peerj-11-14608-s001.zip › Western/Figure2 WB/BCL-2(2).jpg]

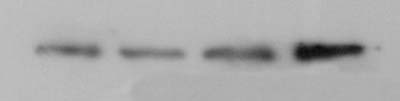

Supplement: Supplemental Information 1 [file peerj-11-14608-s001.zip › Western/Figure2 WB/BCL-2(3).jpg]

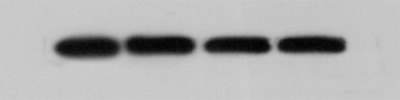

Supplement: Supplemental Information 1 [file peerj-11-14608-s001.zip › Western/Figure2 WB/GADPH(1).jpg]

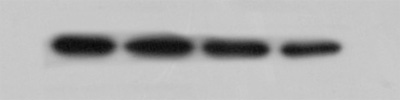

Supplement: Supplemental Information 1 [file peerj-11-14608-s001.zip › Western/Figure2 WB/GADPH(2).jpg]

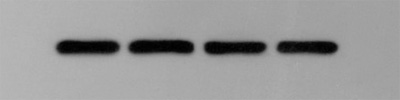

Supplement: Supplemental Information 1 [file peerj-11-14608-s001.zip › Western/Figure2 WB/GADPH(3).jpg]

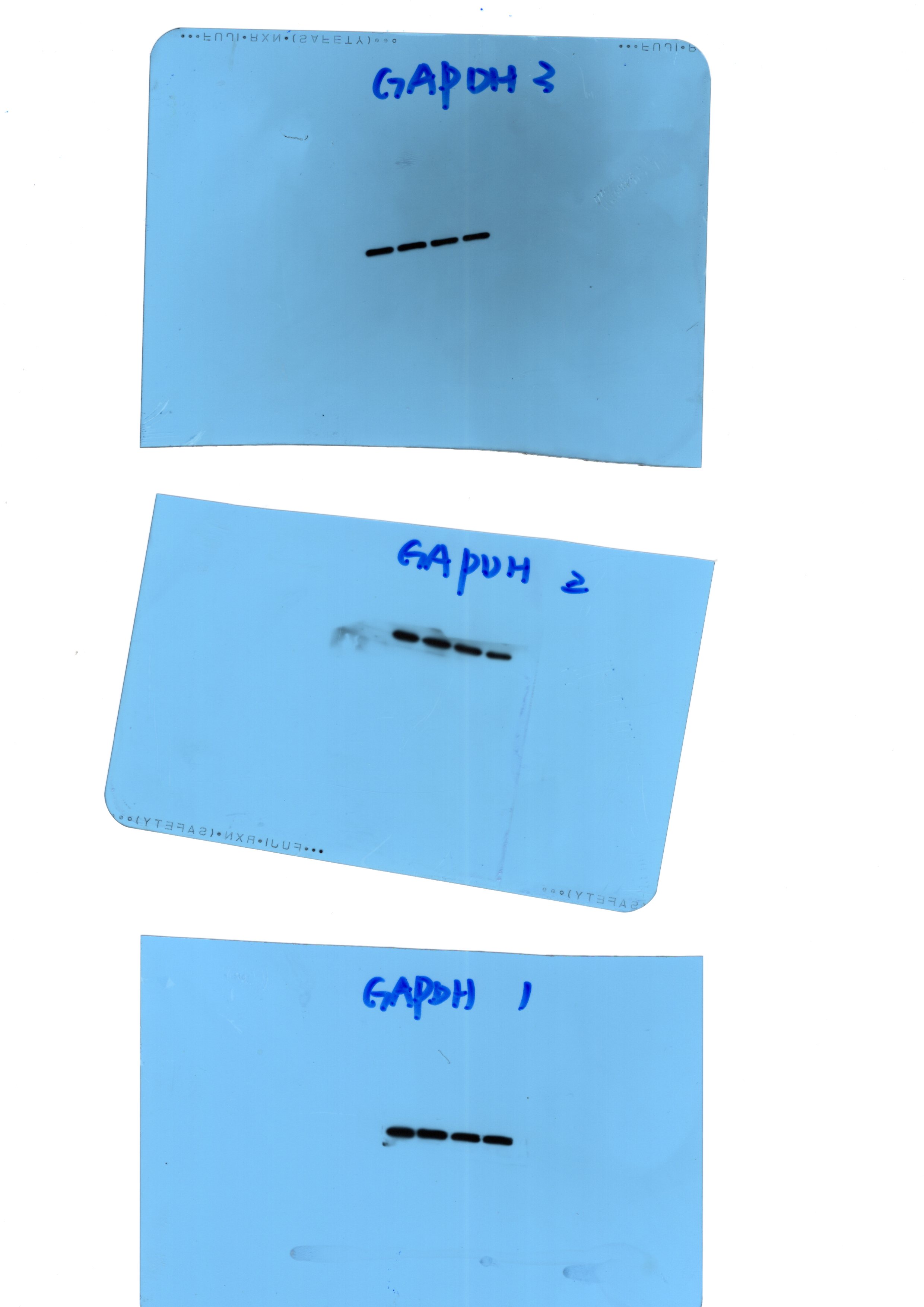

Supplement: Supplemental Information 1 [file peerj-11-14608-s001.zip › Western/Figure2 WB/GAPDH orignal.jpg]

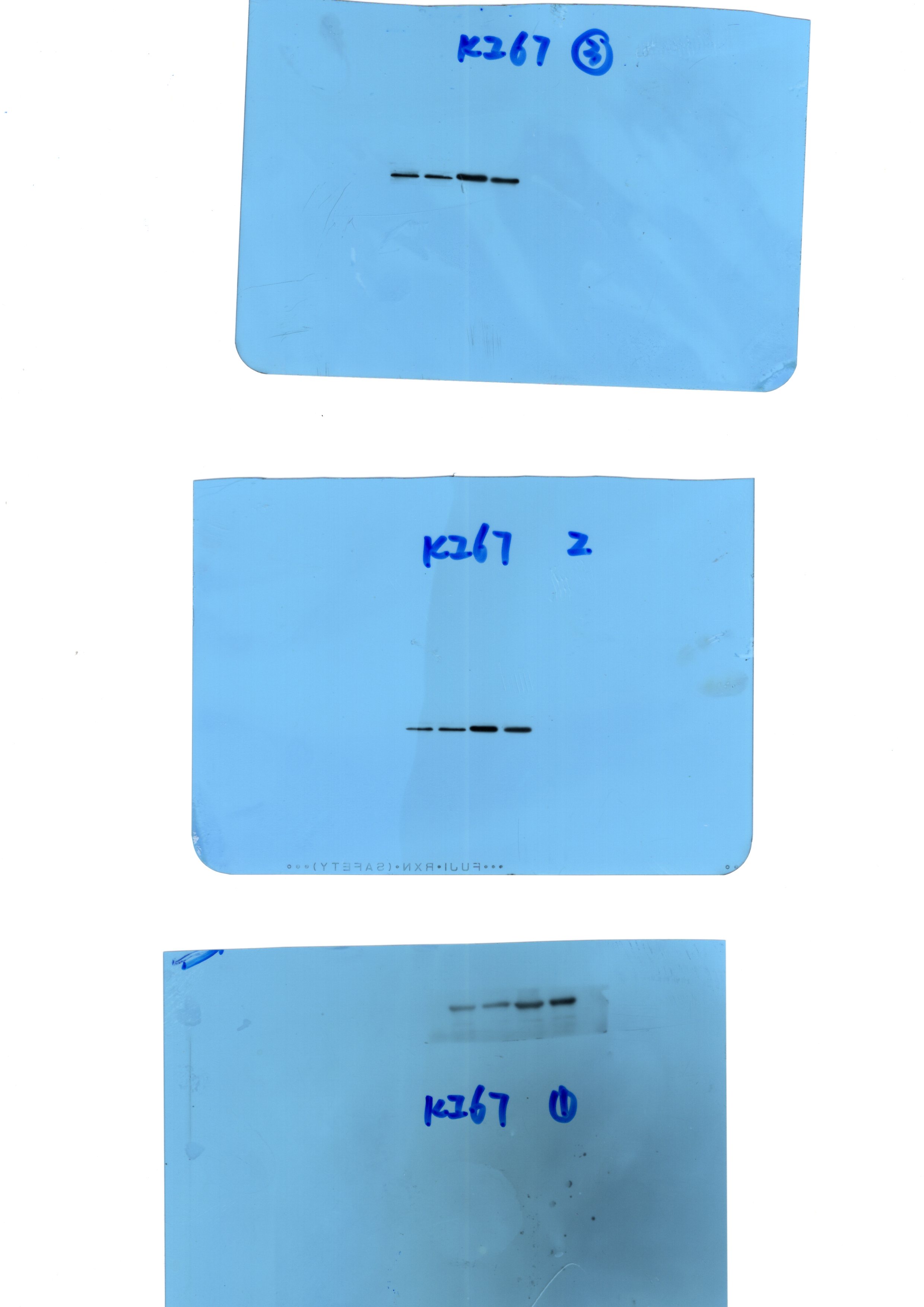

Supplement: Supplemental Information 1 [file peerj-11-14608-s001.zip › Western/Figure2 WB/KI167 orignal.jpg]

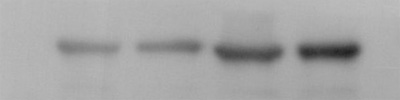

Supplement: Supplemental Information 1 [file peerj-11-14608-s001.zip › Western/Figure2 WB/KI67(1).jpg]

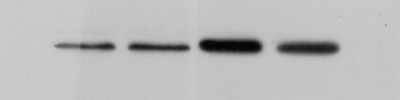

Supplement: Supplemental Information 1 [file peerj-11-14608-s001.zip › Western/Figure2 WB/KI67(2).jpg]

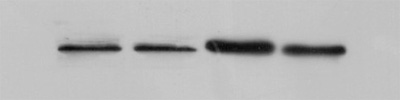

Supplement: Supplemental Information 1 [file peerj-11-14608-s001.zip › Western/Figure2 WB/KI67(3).jpg]

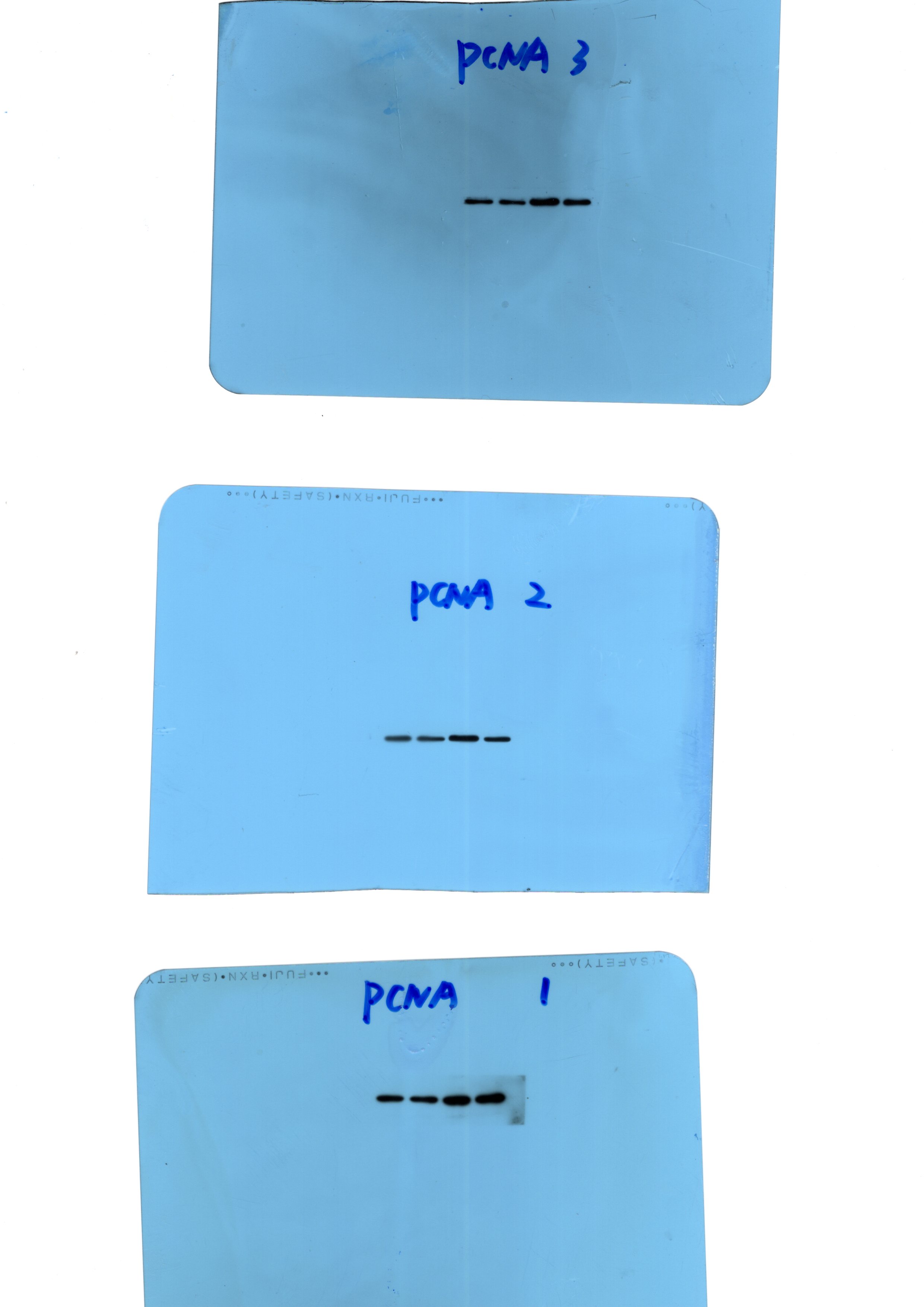

Supplement: Supplemental Information 1 [file peerj-11-14608-s001.zip › Western/Figure2 WB/PCN orignal.jpg]

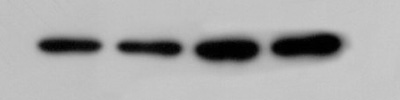

Supplement: Supplemental Information 1 [file peerj-11-14608-s001.zip › Western/Figure2 WB/PCNA(1).jpg]

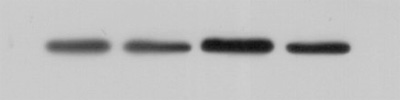

Supplement: Supplemental Information 1 [file peerj-11-14608-s001.zip › Western/Figure2 WB/PCNA(2).jpg]

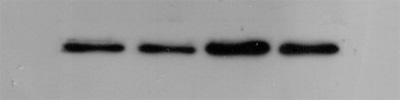

Supplement: Supplemental Information 1 [file peerj-11-14608-s001.zip › Western/Figure2 WB/PCNA(3).jpg]

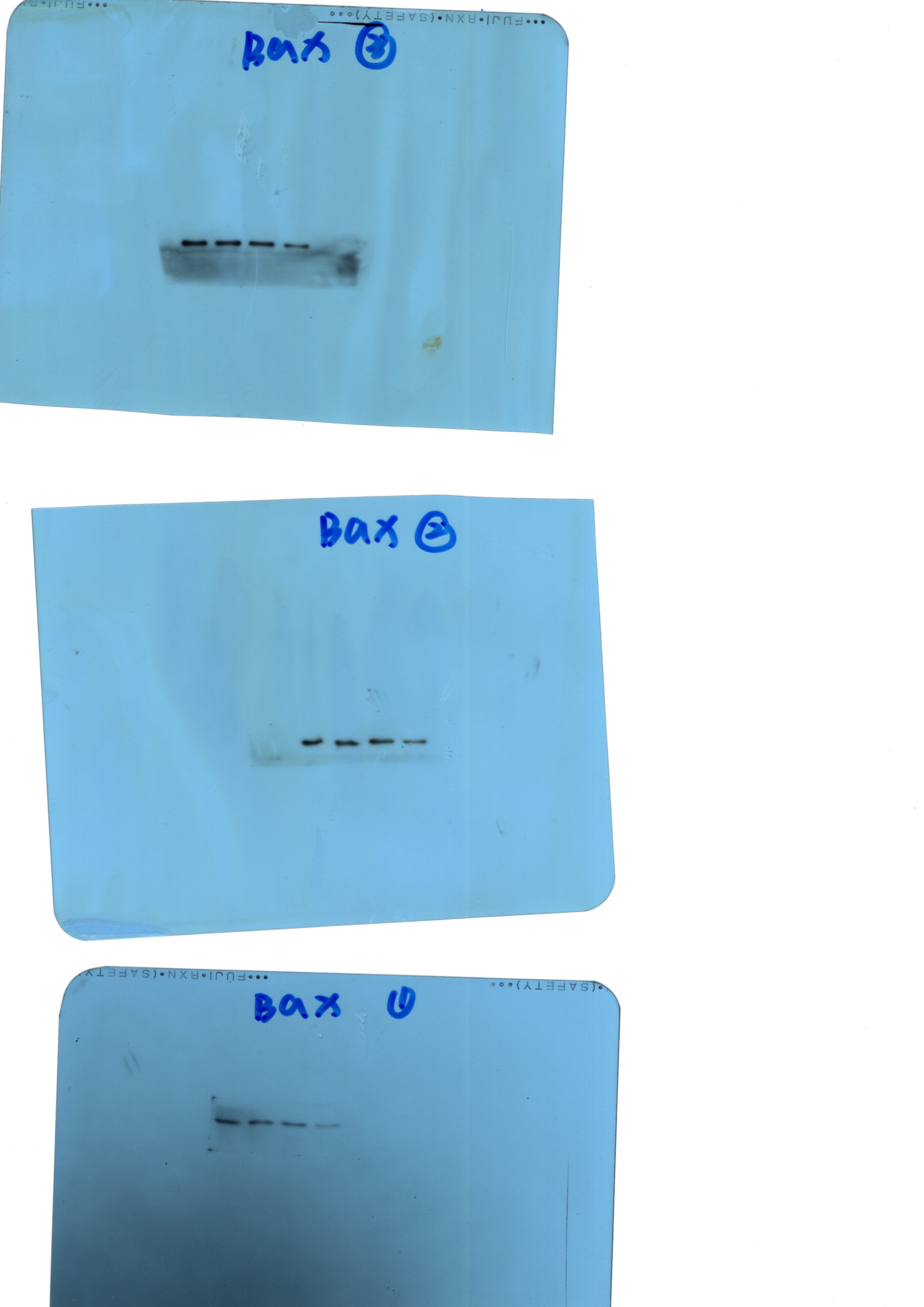

Supplement: Supplemental Information 1 [file peerj-11-14608-s001.zip › Western/Figure3 WB/BAX orignal.jpg]

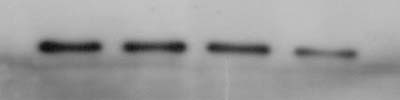

Supplement: Supplemental Information 1 [file peerj-11-14608-s001.zip › Western/Figure3 WB/Bax(1).jpg]

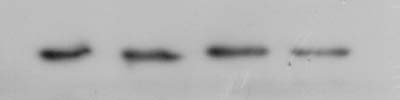

Supplement: Supplemental Information 1 [file peerj-11-14608-s001.zip › Western/Figure3 WB/Bax(2).jpg]

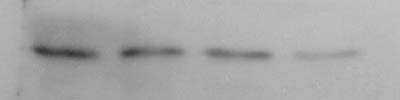

Supplement: Supplemental Information 1 [file peerj-11-14608-s001.zip › Western/Figure3 WB/Bax(3).jpg]

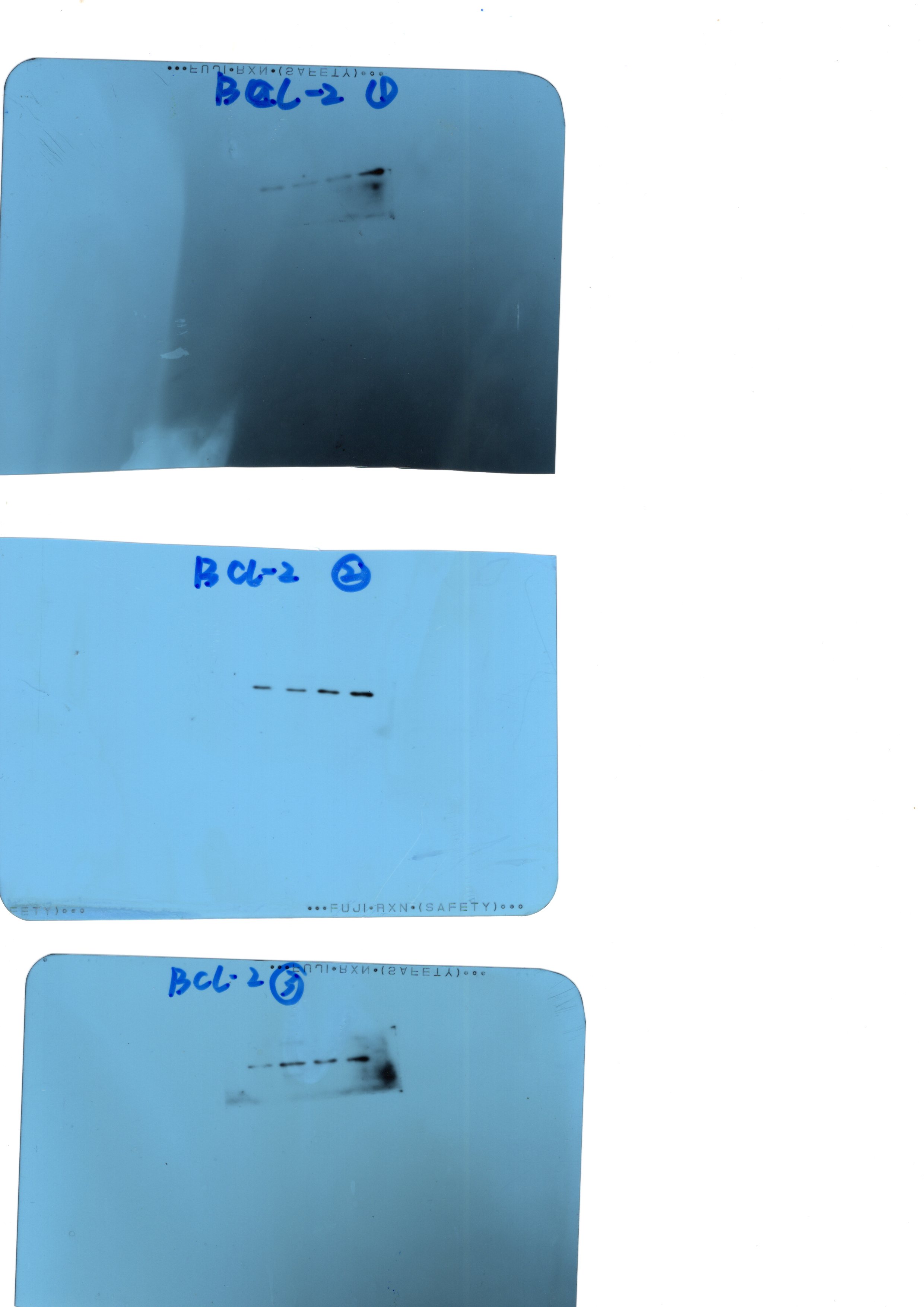

Supplement: Supplemental Information 1 [file peerj-11-14608-s001.zip › Western/Figure3 WB/BCL-2 orignal.jpg]

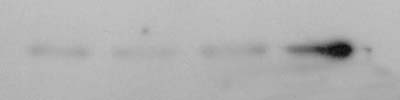

Supplement: Supplemental Information 1 [file peerj-11-14608-s001.zip › Western/Figure3 WB/BCL-2(1).jpg]

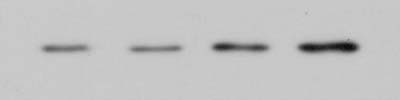

Supplement: Supplemental Information 1 [file peerj-11-14608-s001.zip › Western/Figure3 WB/BCL-2(2).jpg]

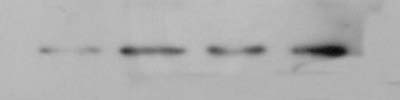

Supplement: Supplemental Information 1 [file peerj-11-14608-s001.zip › Western/Figure3 WB/BCL-2(3).jpg]

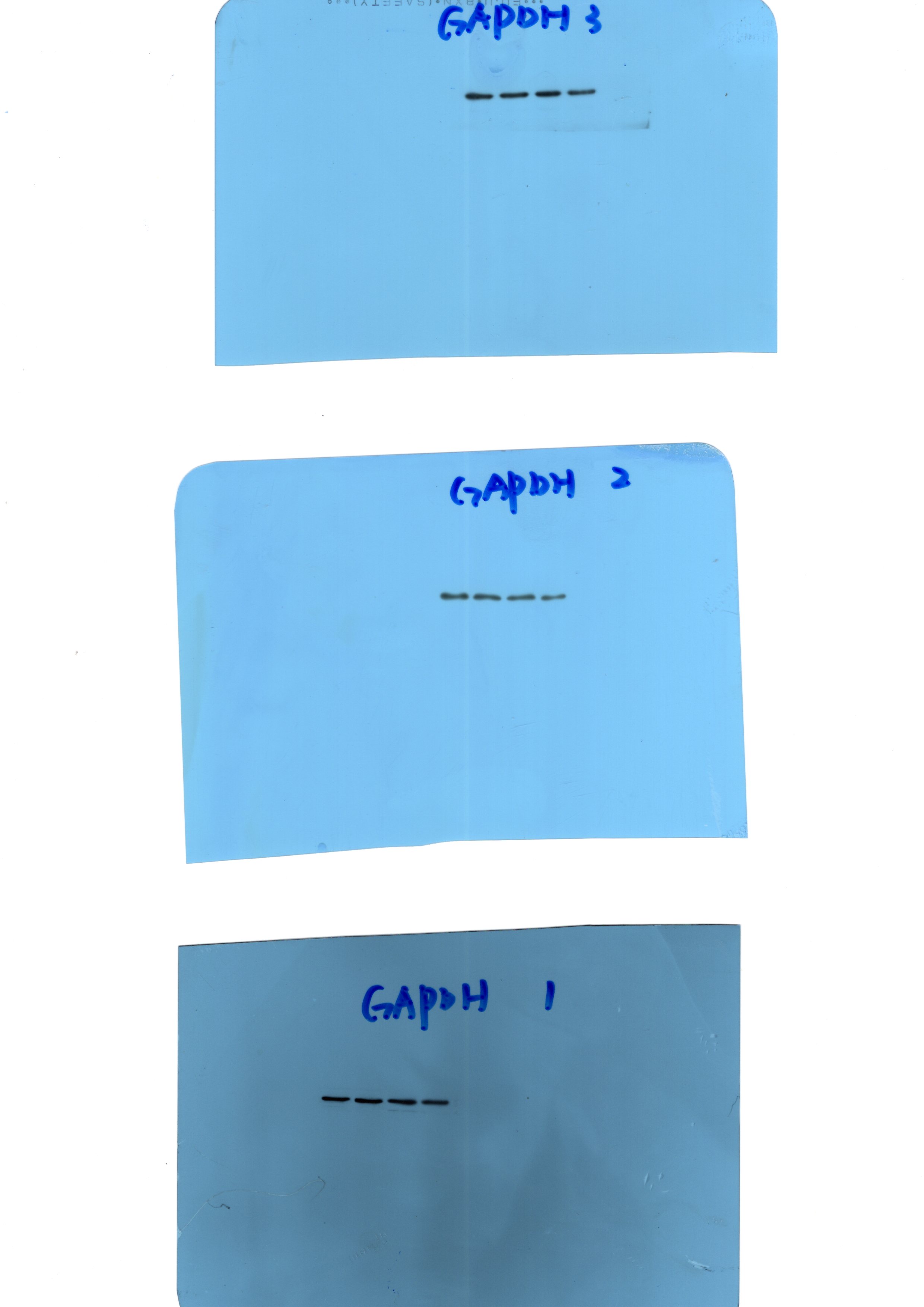

Supplement: Supplemental Information 1 [file peerj-11-14608-s001.zip › Western/Figure3 WB/GAPDH orignal.jpg]

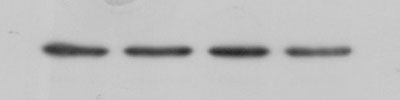

Supplement: Supplemental Information 1 [file peerj-11-14608-s001.zip › Western/Figure3 WB/GAPDH(1).jpg]

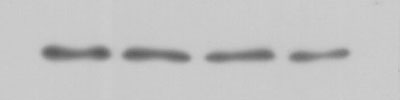

Supplement: Supplemental Information 1 [file peerj-11-14608-s001.zip › Western/Figure3 WB/GAPDH(2).jpg]

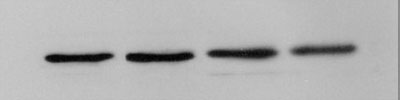

Supplement: Supplemental Information 1 [file peerj-11-14608-s001.zip › Western/Figure3 WB/GAPDH(3).jpg]

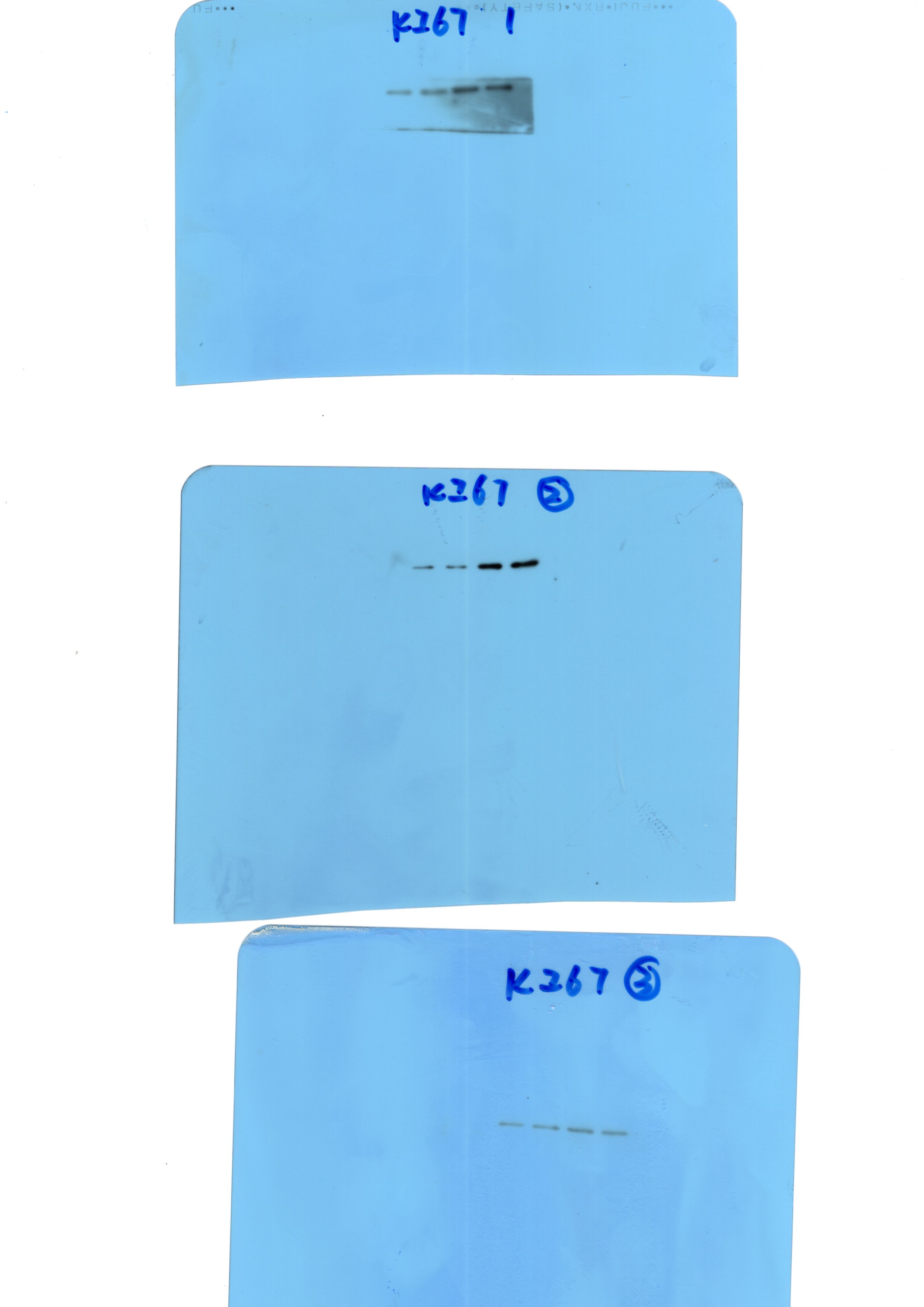

Supplement: Supplemental Information 1 [file peerj-11-14608-s001.zip › Western/Figure3 WB/KI67 orignal.jpg]

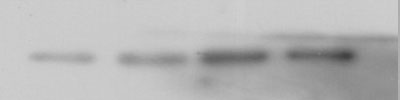

Supplement: Supplemental Information 1 [file peerj-11-14608-s001.zip › Western/Figure3 WB/KI67(1).jpg]

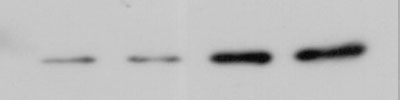

Supplement: Supplemental Information 1 [file peerj-11-14608-s001.zip › Western/Figure3 WB/KI67(2).jpg]

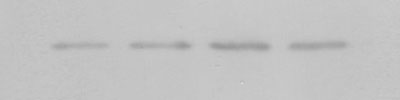

Supplement: Supplemental Information 1 [file peerj-11-14608-s001.zip › Western/Figure3 WB/KI67(3).jpg]

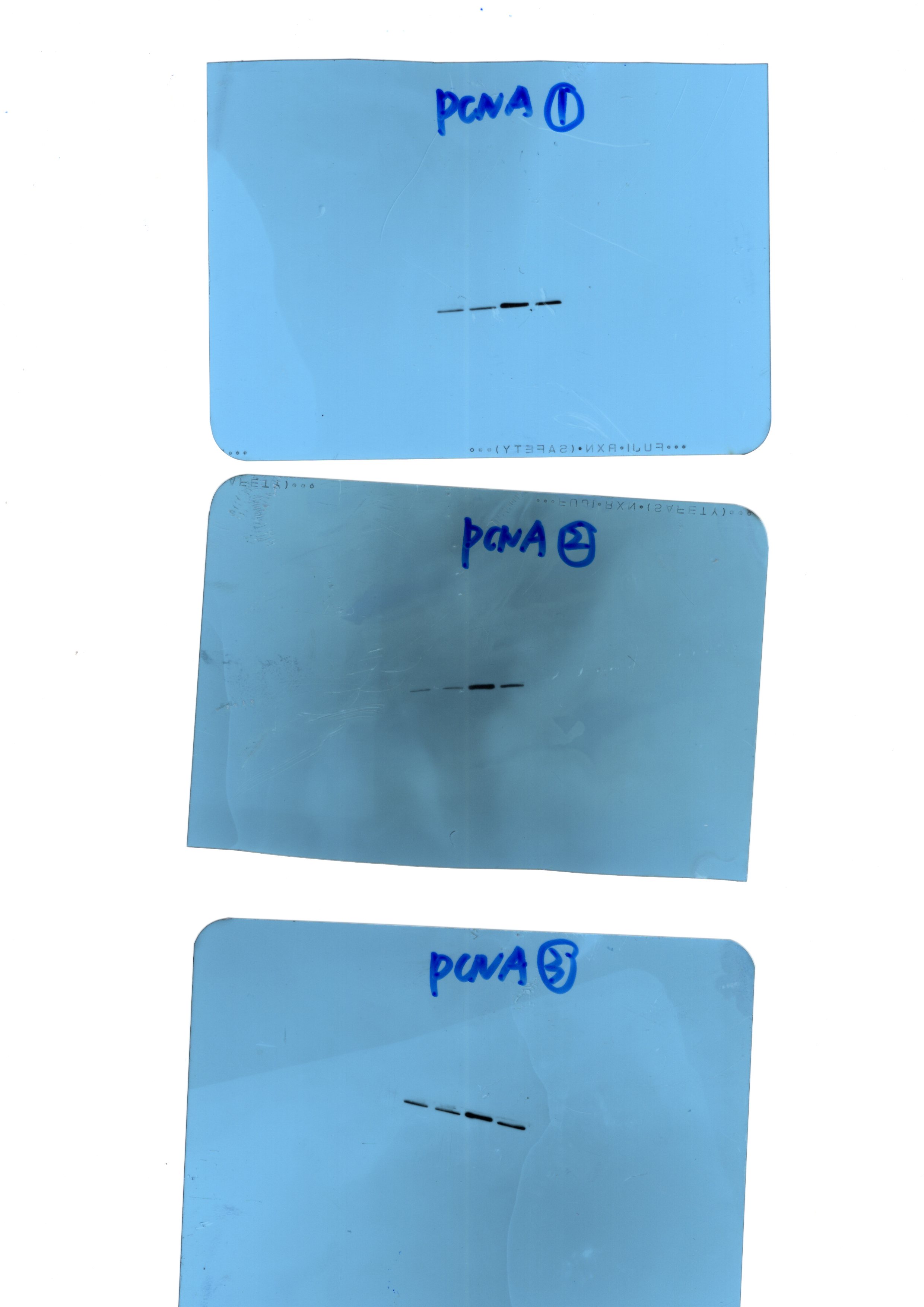

Supplement: Supplemental Information 1 [file peerj-11-14608-s001.zip › Western/Figure3 WB/PCNA orignal.jpg]

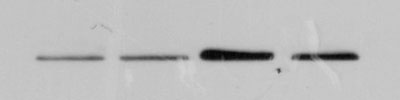

Supplement: Supplemental Information 1 [file peerj-11-14608-s001.zip › Western/Figure3 WB/PCNA(1).jpg]

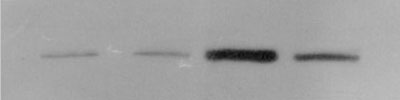

Supplement: Supplemental Information 1 [file peerj-11-14608-s001.zip › Western/Figure3 WB/PCNA(2).jpg]

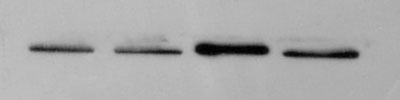

Supplement: Supplemental Information 1 [file peerj-11-14608-s001.zip › Western/Figure3 WB/PCNA(3).jpg]

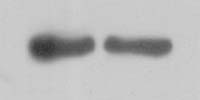

Supplement: Supplemental Information 1 [file peerj-11-14608-s001.zip › Western/Figure7 WB/GADPH(1).jpg]

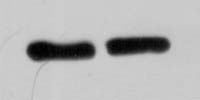

Supplement: Supplemental Information 1 [file peerj-11-14608-s001.zip › Western/Figure7 WB/GADPH(2).jpg]

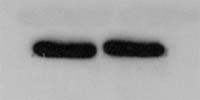

Supplement: Supplemental Information 1 [file peerj-11-14608-s001.zip › Western/Figure7 WB/GADPH(3).jpg]

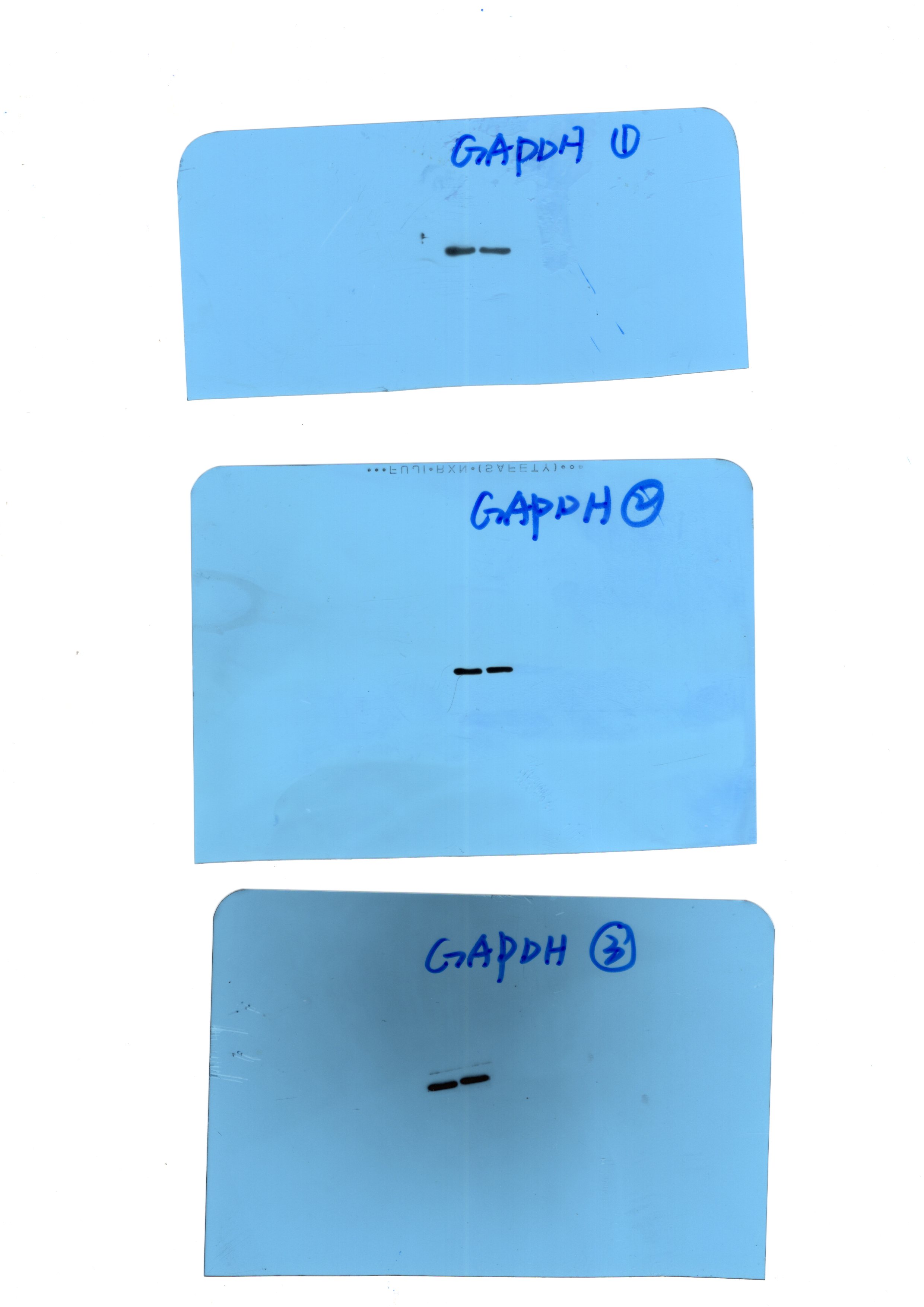

Supplement: Supplemental Information 1 [file peerj-11-14608-s001.zip › Western/Figure7 WB/GAPDH orignal.jpg]

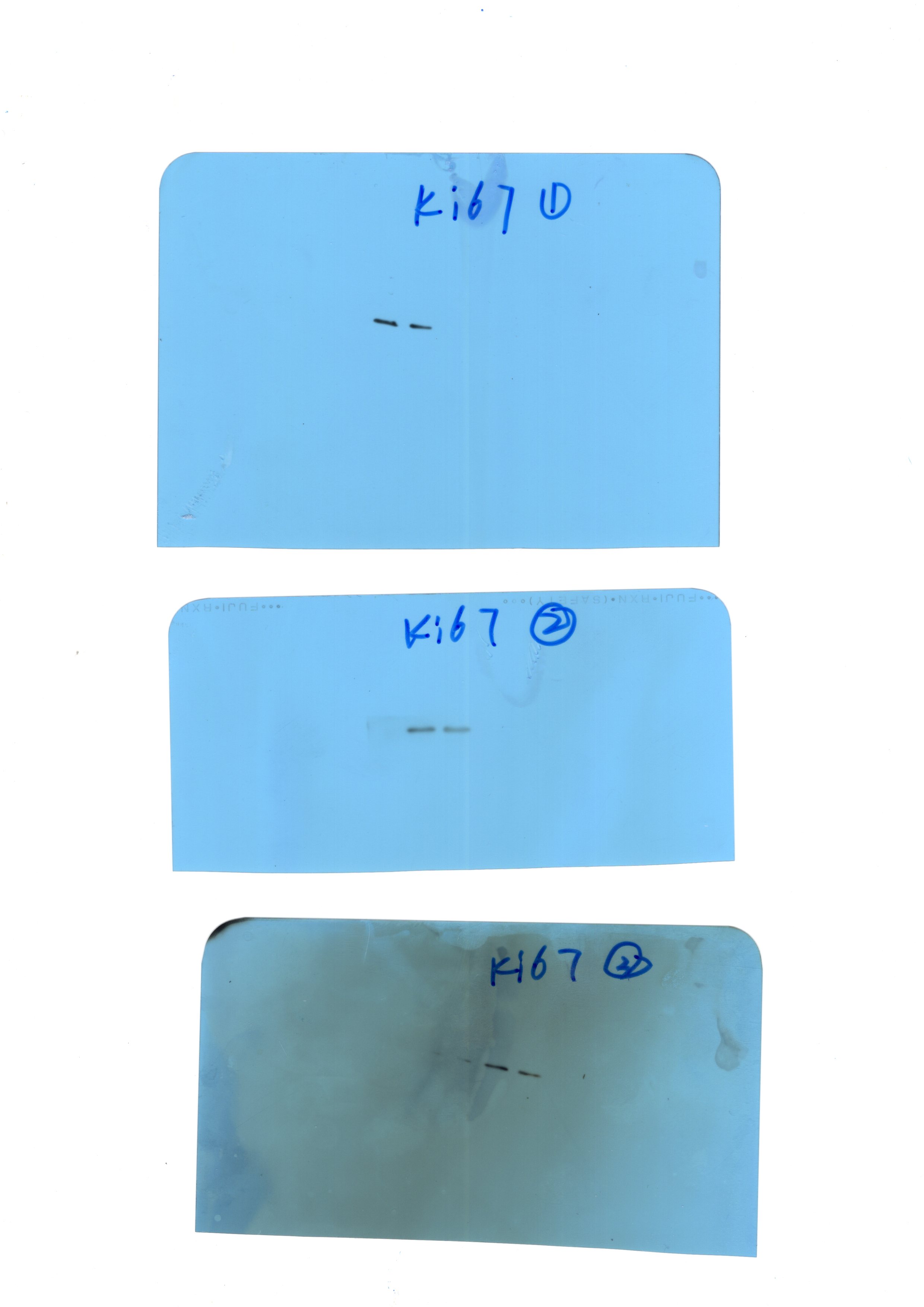

Supplement: Supplemental Information 1 [file peerj-11-14608-s001.zip › Western/Figure7 WB/KI67 orignal.jpg]

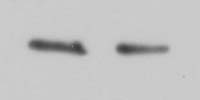

Supplement: Supplemental Information 1 [file peerj-11-14608-s001.zip › Western/Figure7 WB/KI67(1).jpg]

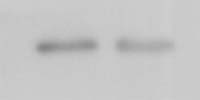

Supplement: Supplemental Information 1 [file peerj-11-14608-s001.zip › Western/Figure7 WB/KI67(2).jpg]

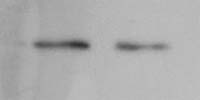

Supplement: Supplemental Information 1 [file peerj-11-14608-s001.zip › Western/Figure7 WB/KI67(3).jpg]

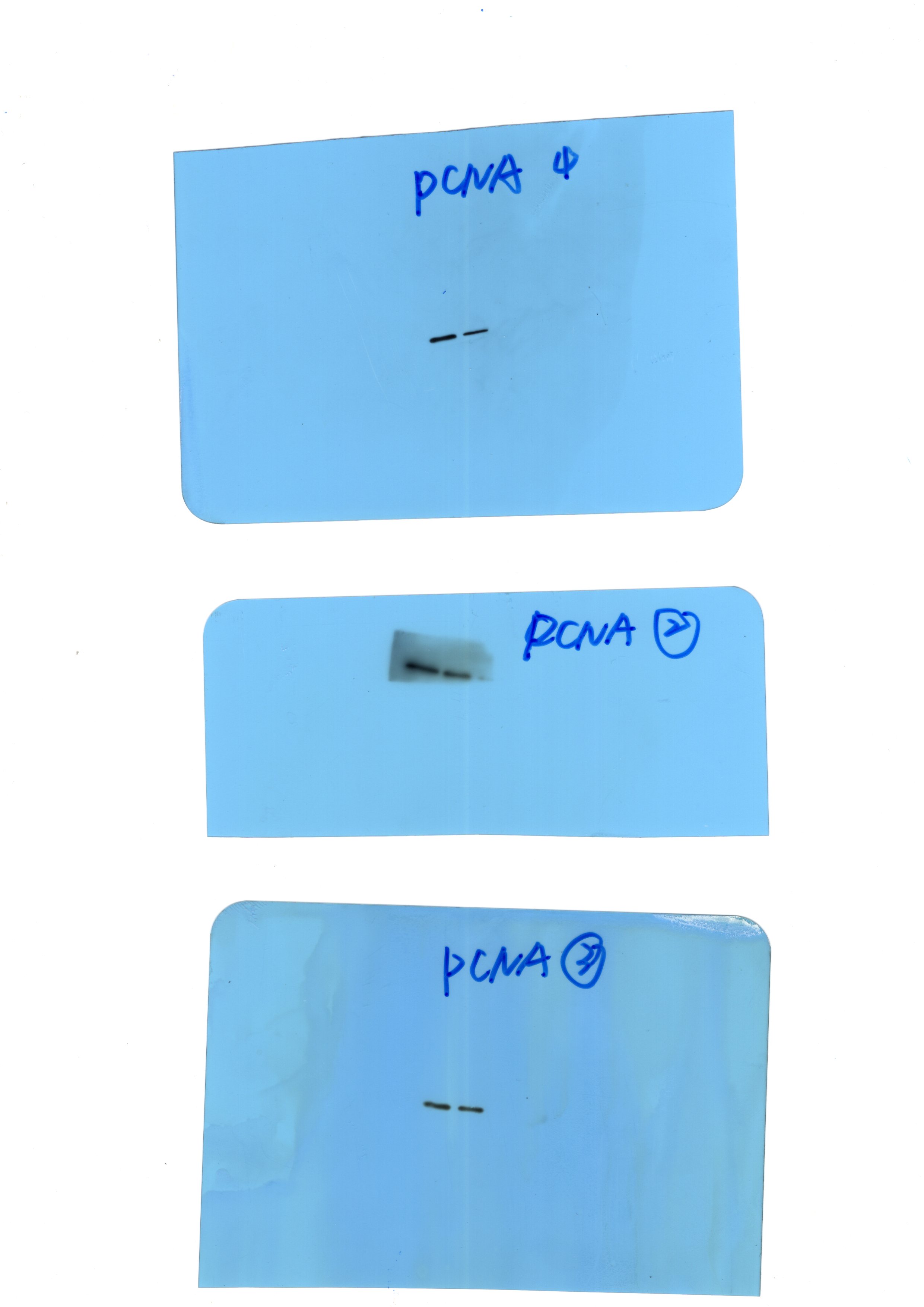

Supplement: Supplemental Information 1 [file peerj-11-14608-s001.zip › Western/Figure7 WB/PCNA orignal.jpg]

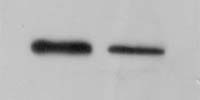

Supplement: Supplemental Information 1 [file peerj-11-14608-s001.zip › Western/Figure7 WB/PCNA(1).jpg]

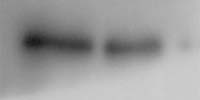

Supplement: Supplemental Information 1 [file peerj-11-14608-s001.zip › Western/Figure7 WB/PCNA(2).jpg]

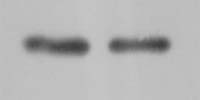

Supplement: Supplemental Information 1 [file peerj-11-14608-s001.zip › Western/Figure7 WB/PCNA(3).jpg]

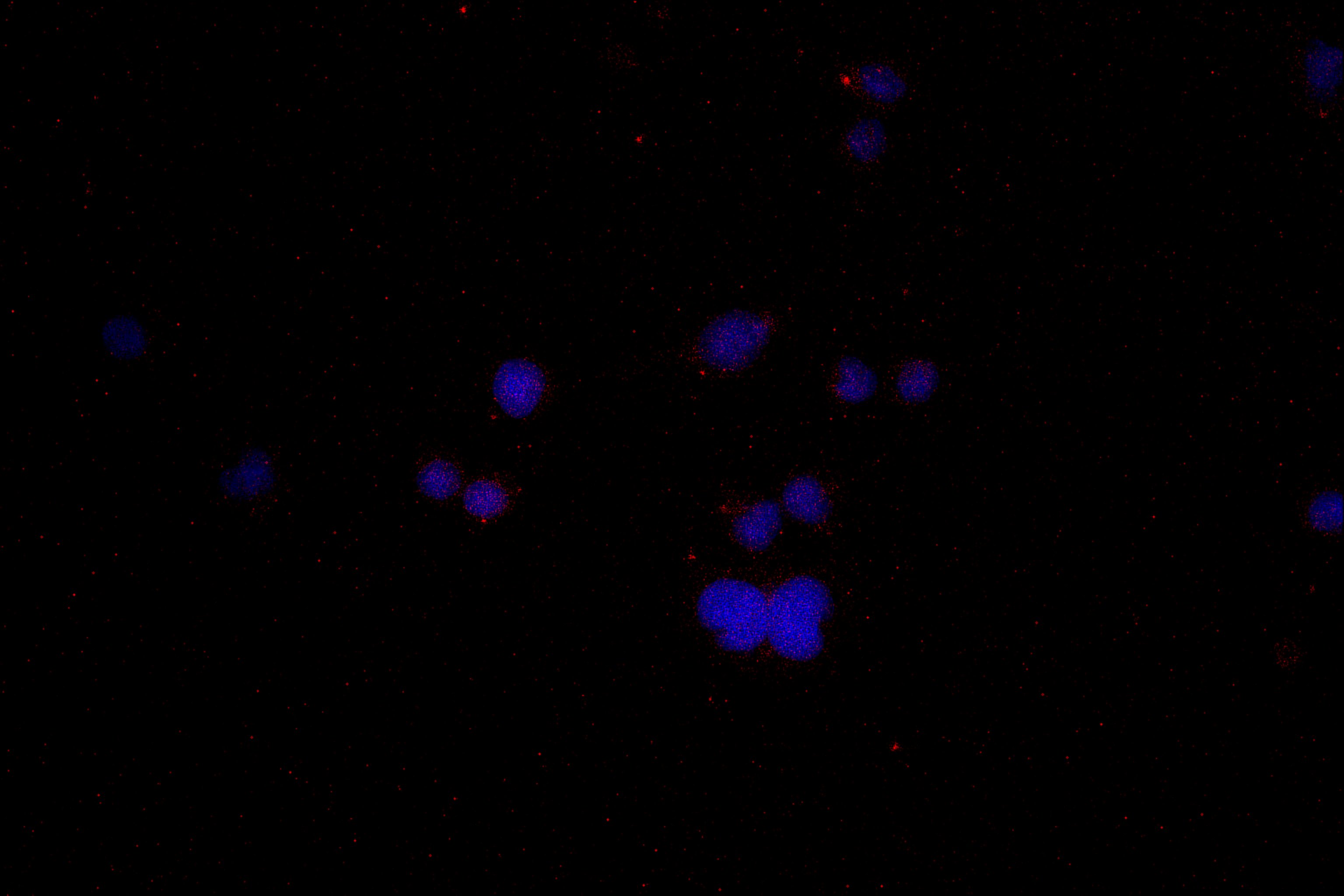

Supplement: Supplemental Information 2 [file peerj-11-14608-s002.zip › micrograph Figure1 CD80/MO-NC组/1-1-1.jpg]

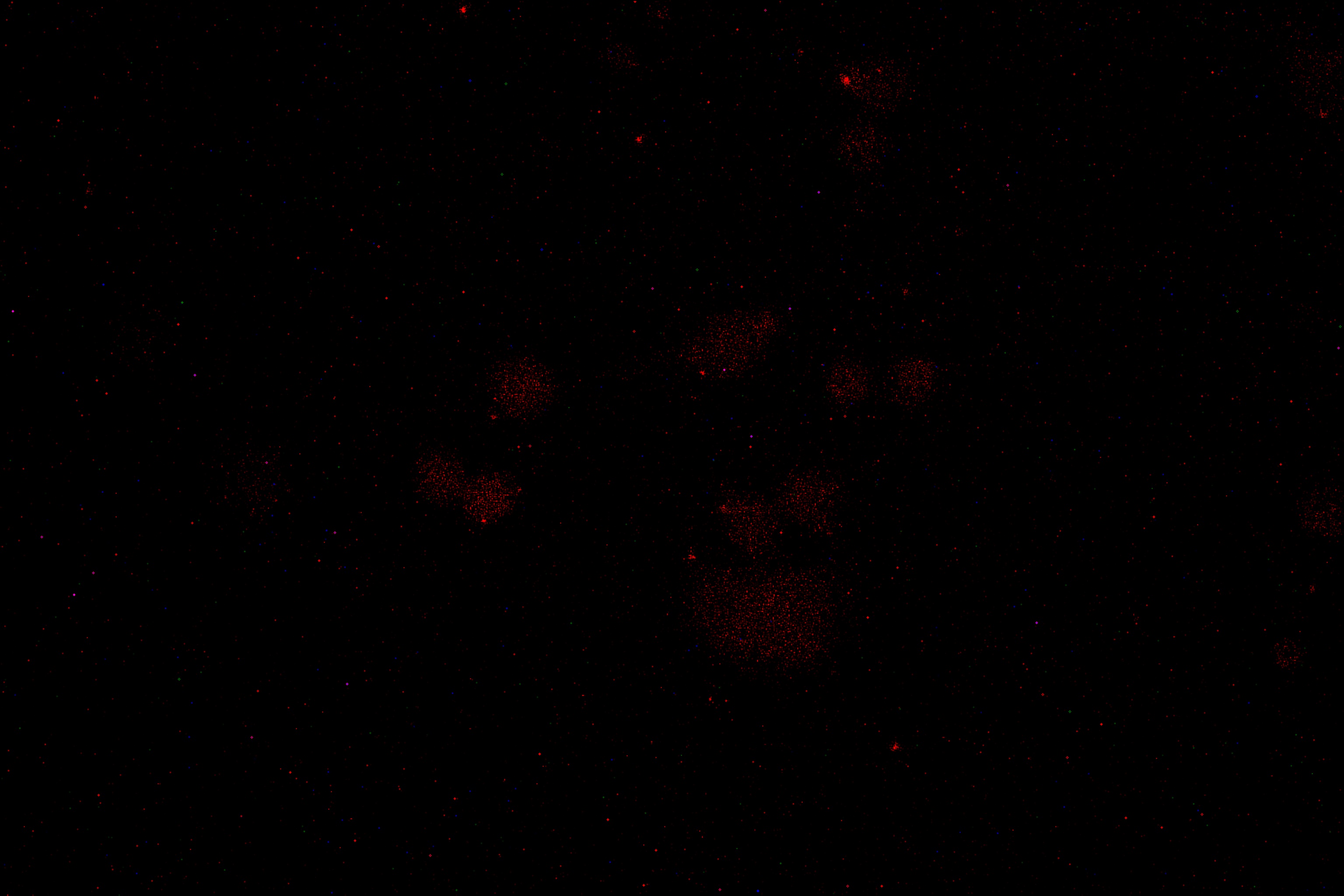

Supplement: Supplemental Information 2 [file peerj-11-14608-s002.zip › micrograph Figure1 CD80/MO-NC组/1-1.jpg]

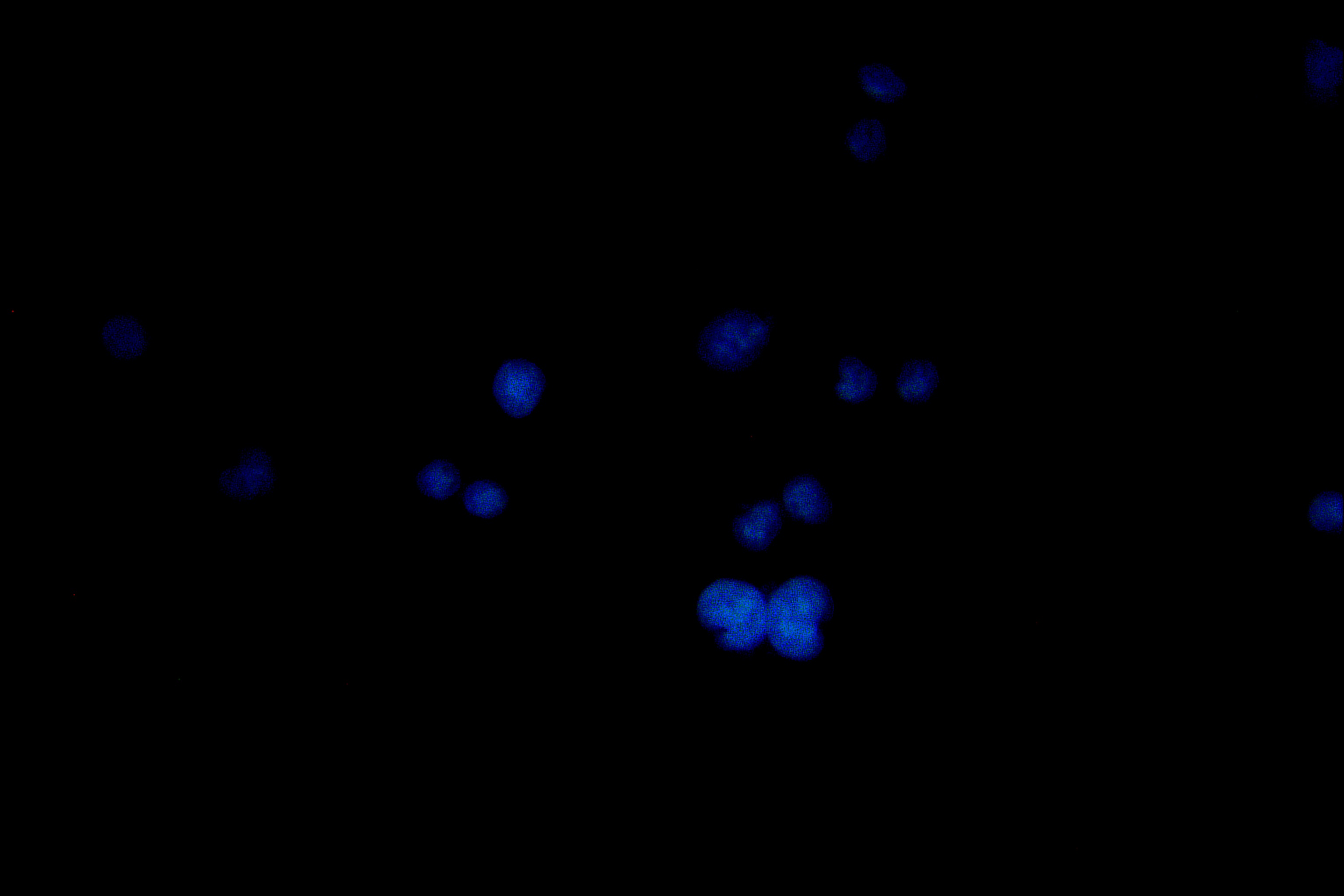

Supplement: Supplemental Information 2 [file peerj-11-14608-s002.zip › micrograph Figure1 CD80/MO-NC组/1.jpg]

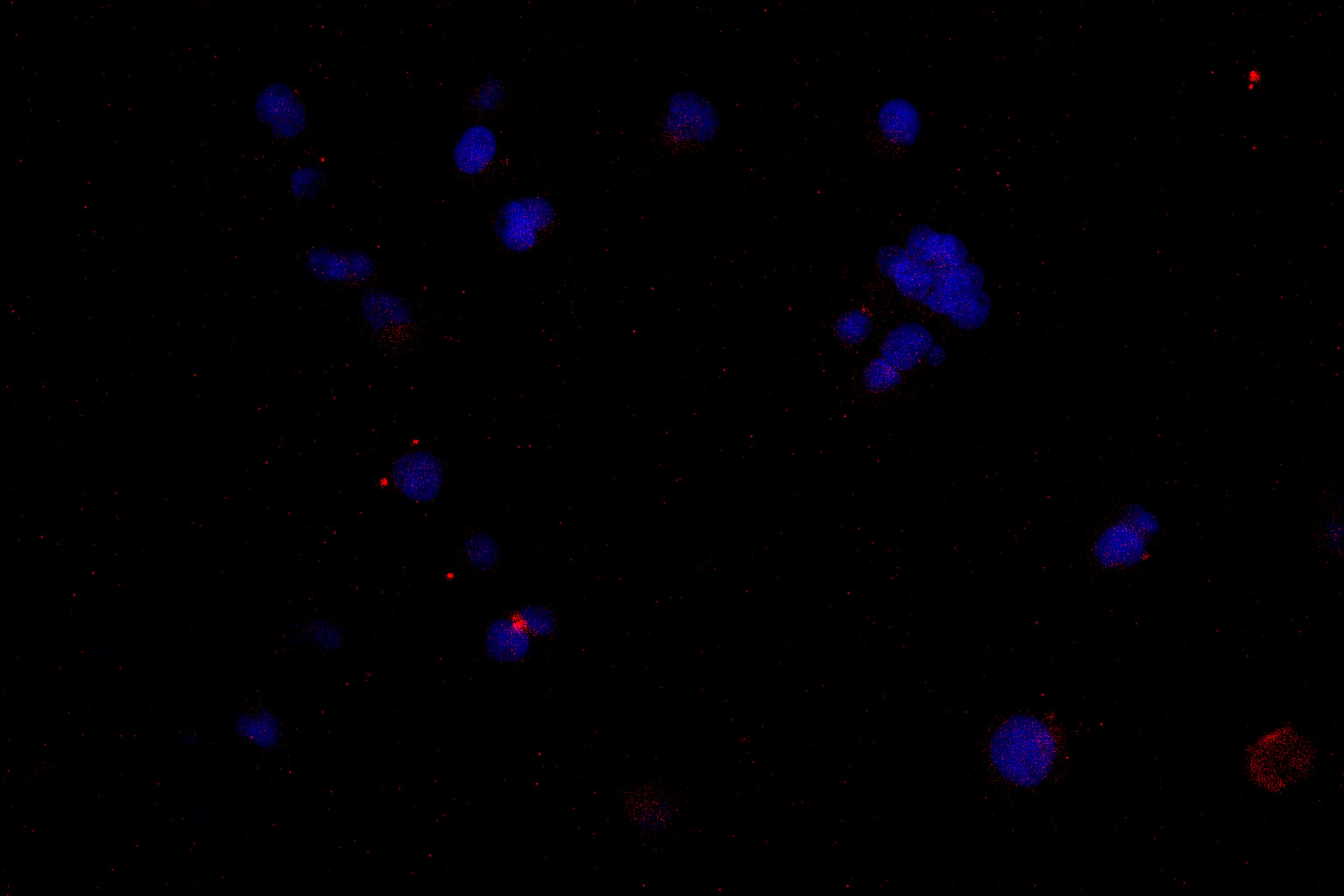

Supplement: Supplemental Information 2 [file peerj-11-14608-s002.zip › micrograph Figure1 CD80/MO-NC组/2-2-2.jpg]

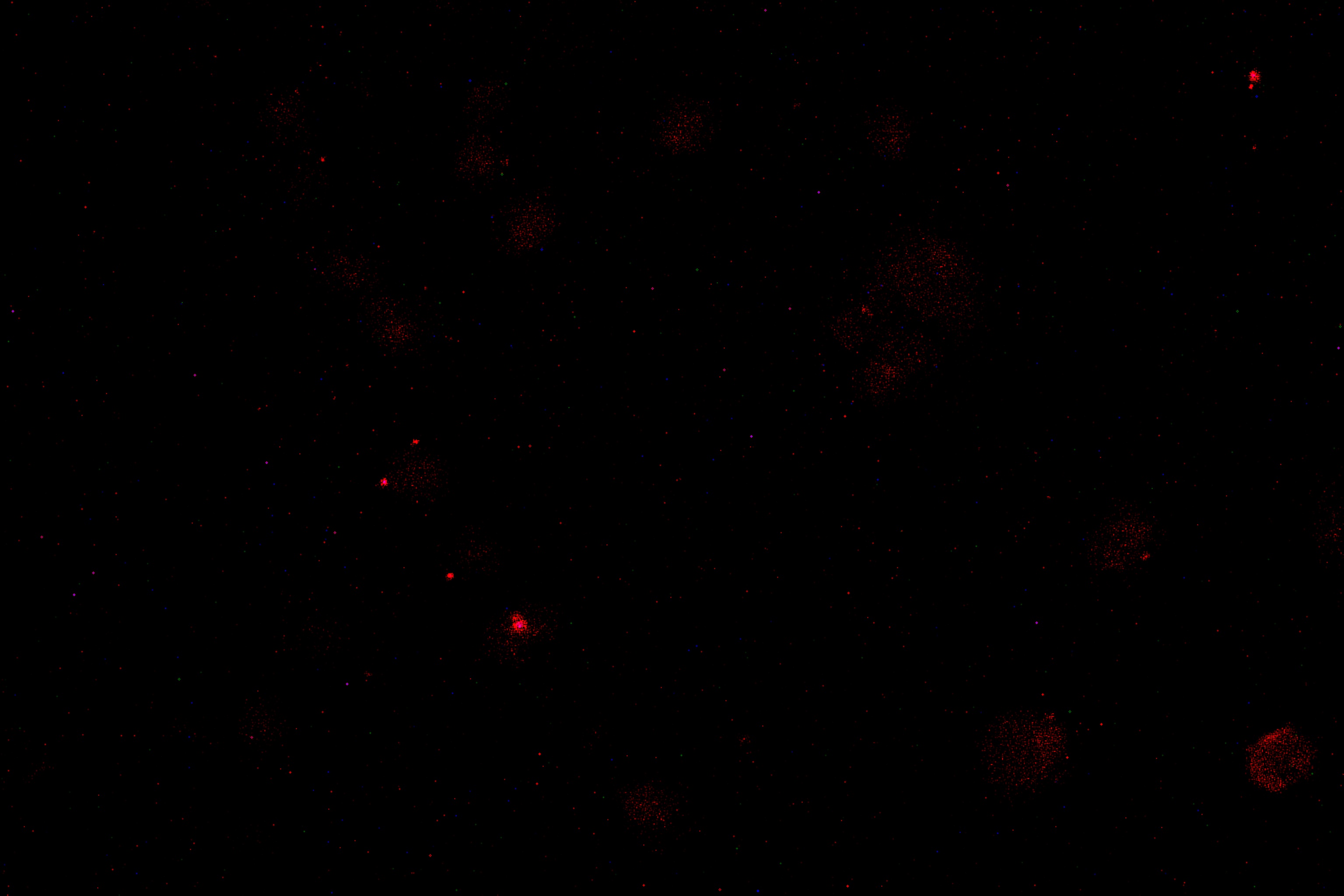

Supplement: Supplemental Information 2 [file peerj-11-14608-s002.zip › micrograph Figure1 CD80/MO-NC组/2-2.jpg]

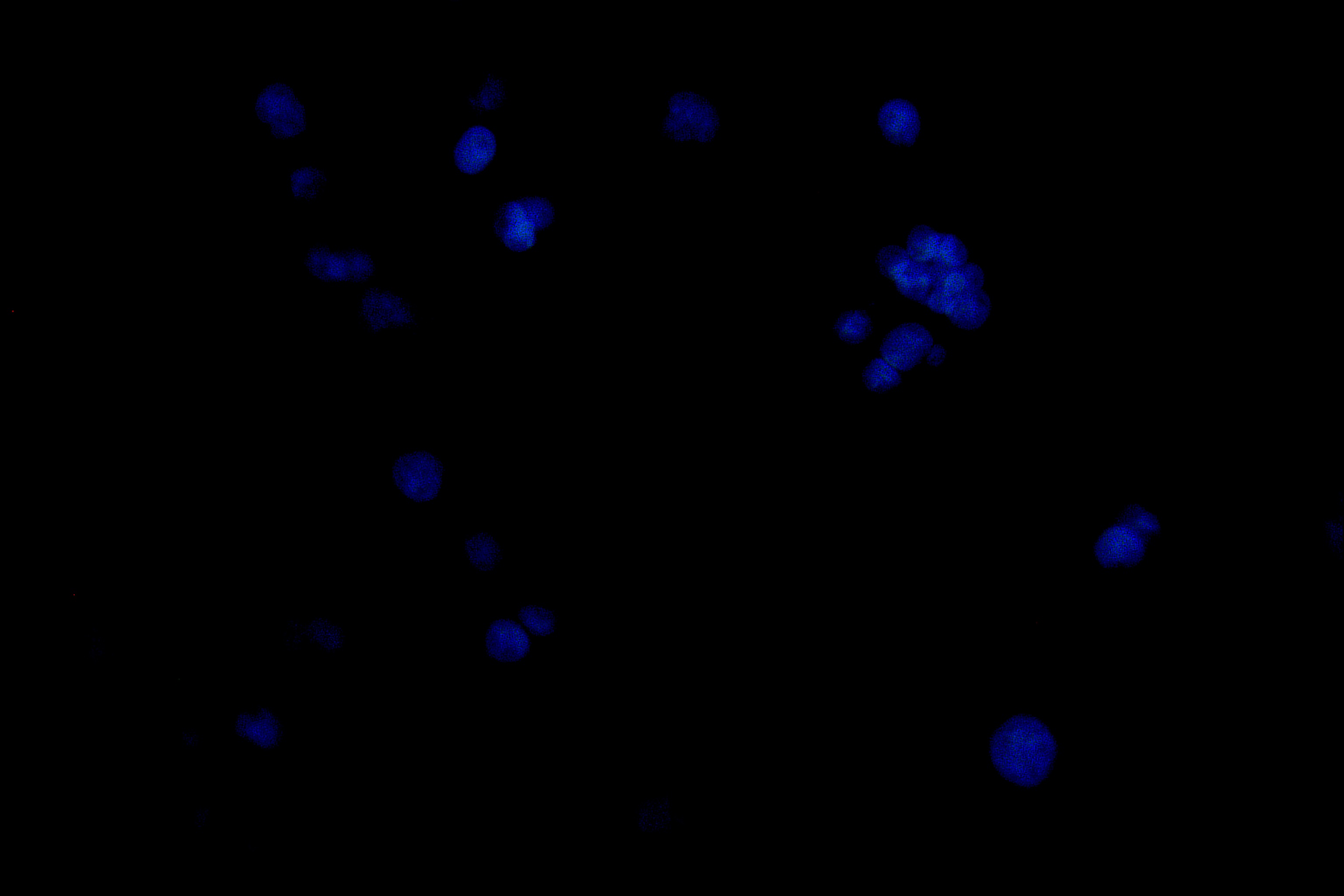

Supplement: Supplemental Information 2 [file peerj-11-14608-s002.zip › micrograph Figure1 CD80/MO-NC组/2.jpg]

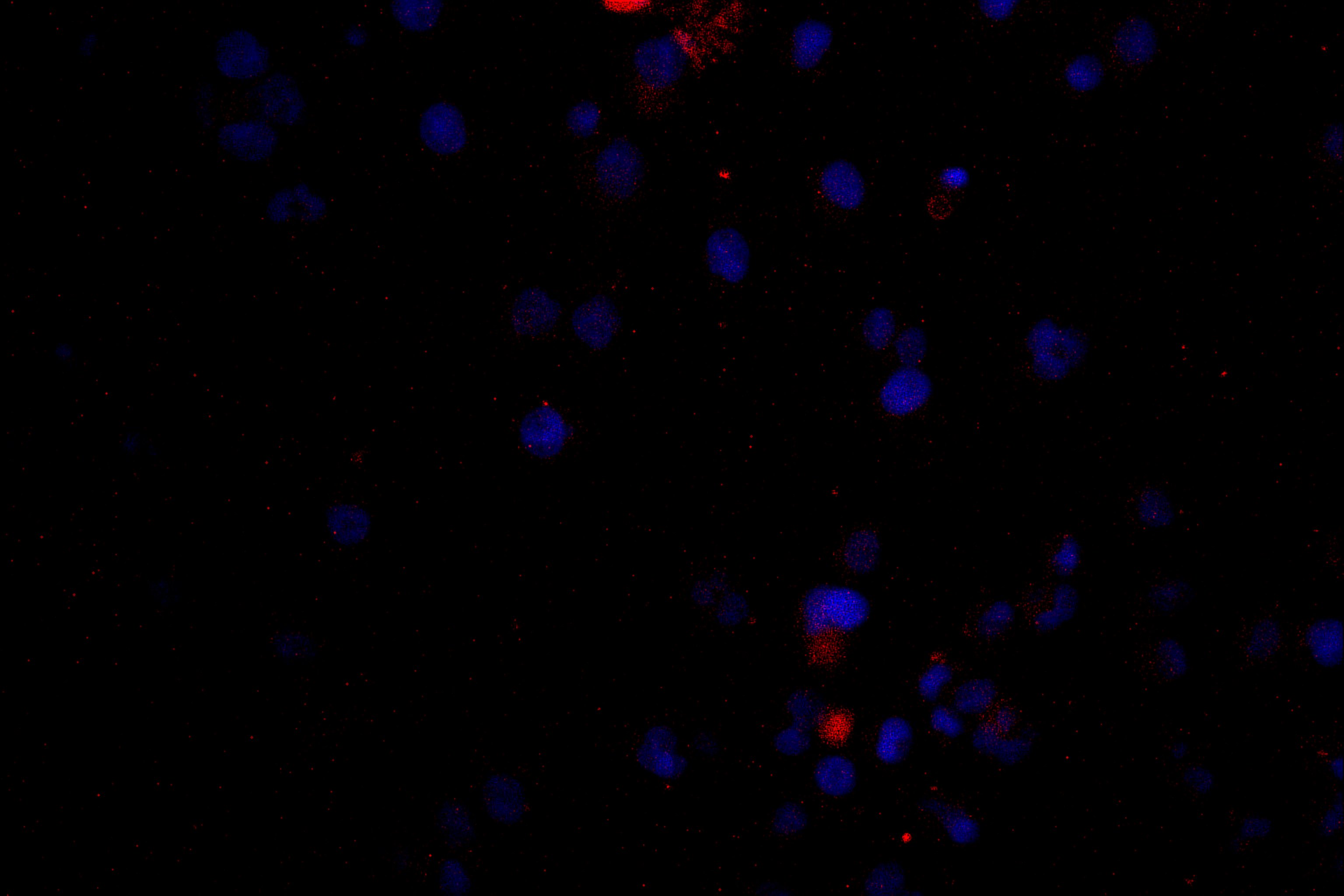

Supplement: Supplemental Information 2 [file peerj-11-14608-s002.zip › micrograph Figure1 CD80/MO-NC组/3-3-3.jpg]

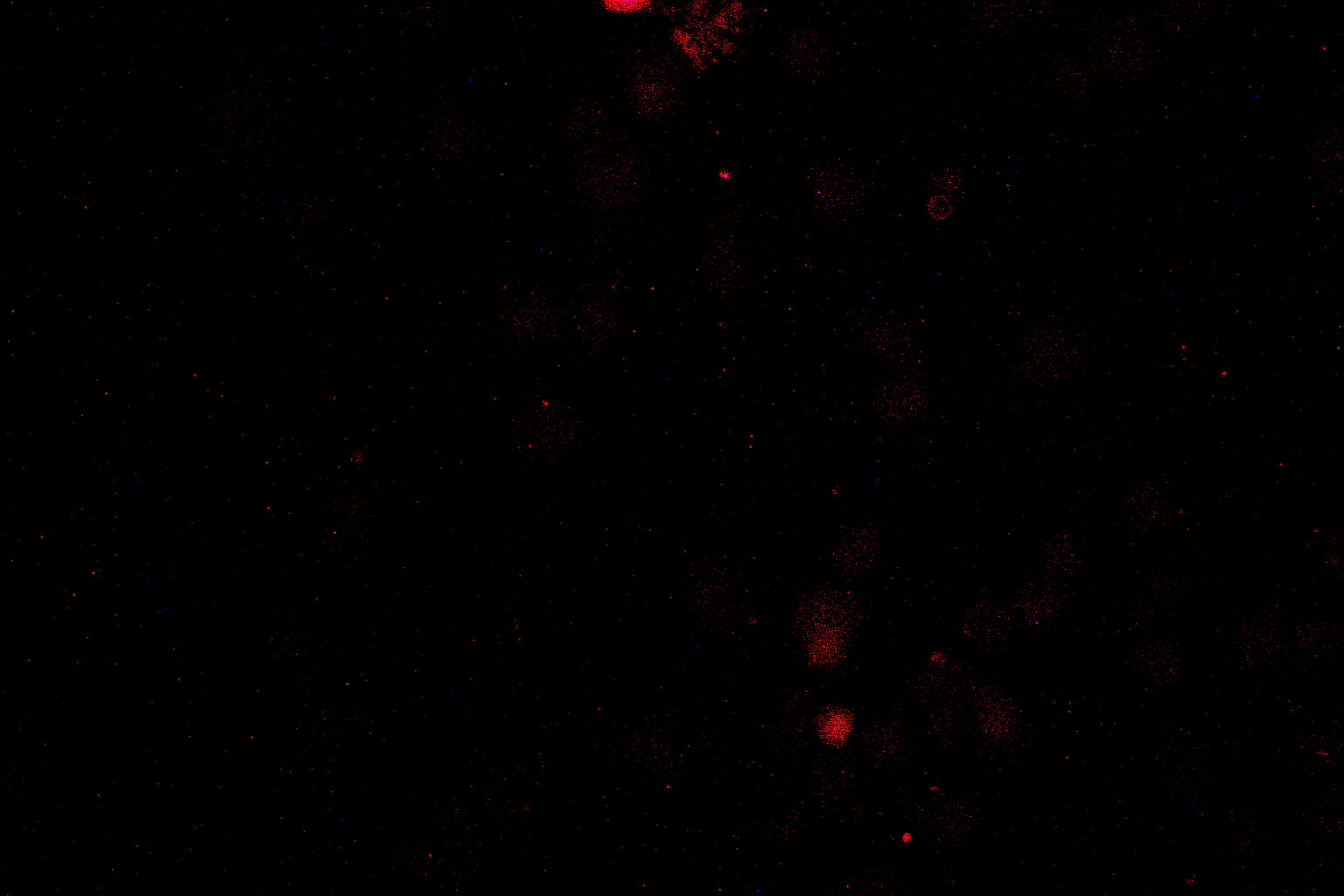

Supplement: Supplemental Information 2 [file peerj-11-14608-s002.zip › micrograph Figure1 CD80/MO-NC组/3-3.jpg]
