# Supplementary figures and images for: Exosomes derived from M1 macrophages inhibit the proliferation of the A549 and H1299 lung cancer cell lines via the miRNA-let-7b-5p-GNG5 axis (part 2 of 4)
Source: PeerJ. 2023 Jan 9;11:e14608. doi: 10.7717/peerj.14608 (PMC9835688; doi:10.7717/peerj.14608)

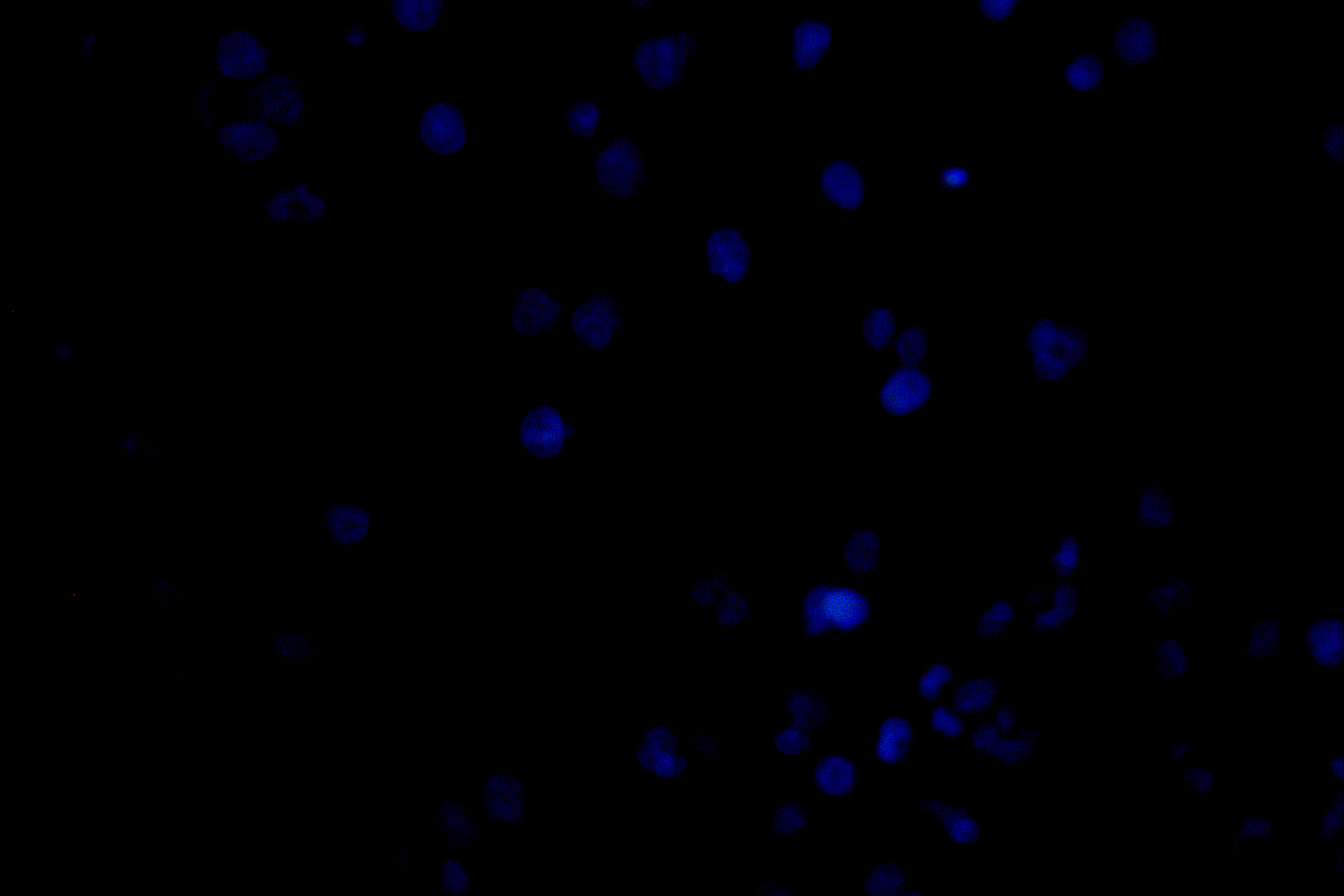

Supplement: Supplemental Information 2 [file peerj-11-14608-s002.zip › micrograph Figure1 CD80/MO-NC组/3.jpg]

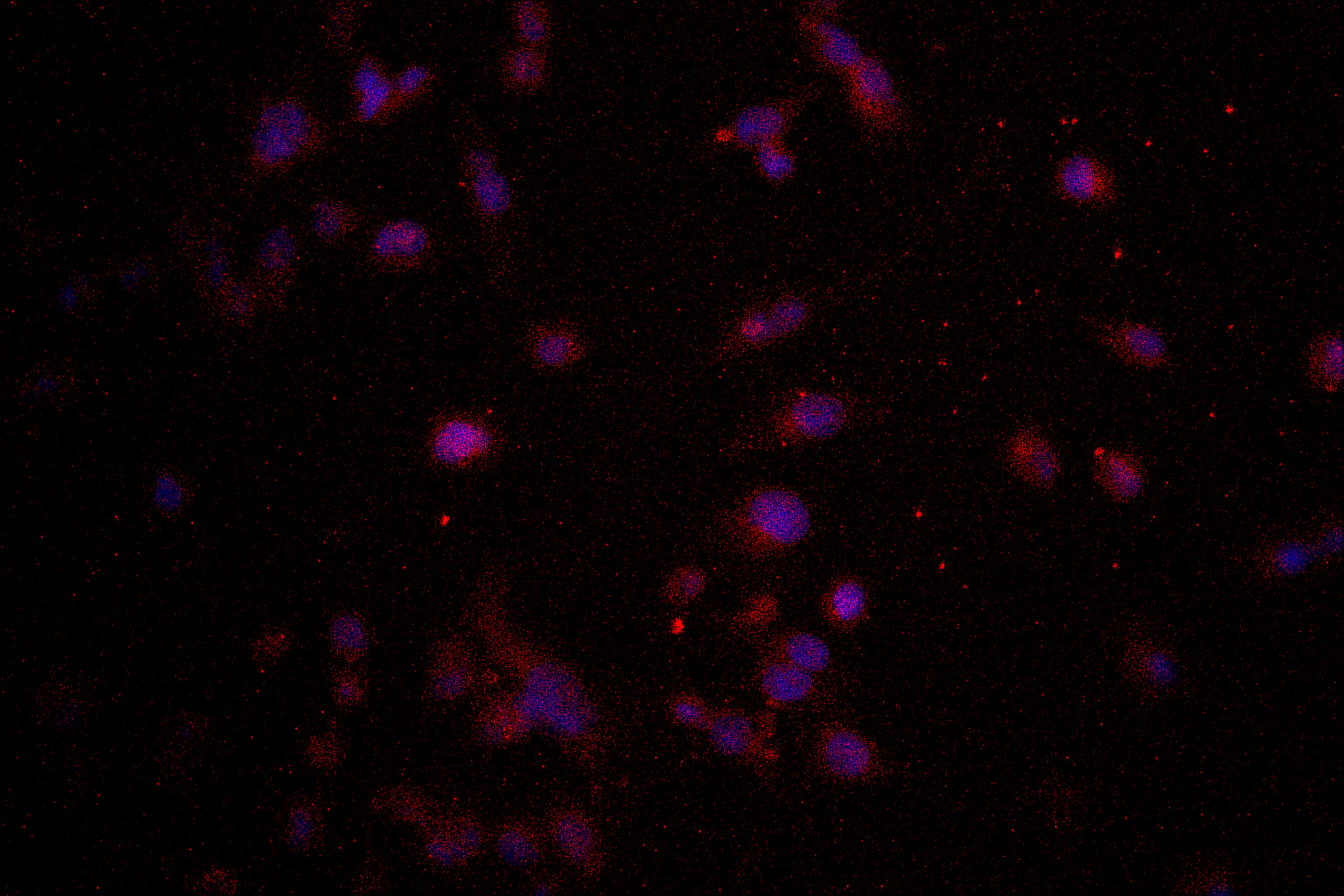

Supplement: Supplemental Information 2 [file peerj-11-14608-s002.zip › micrograph Figure1 CD80/MO-vitexin/1-1-1.jpg]

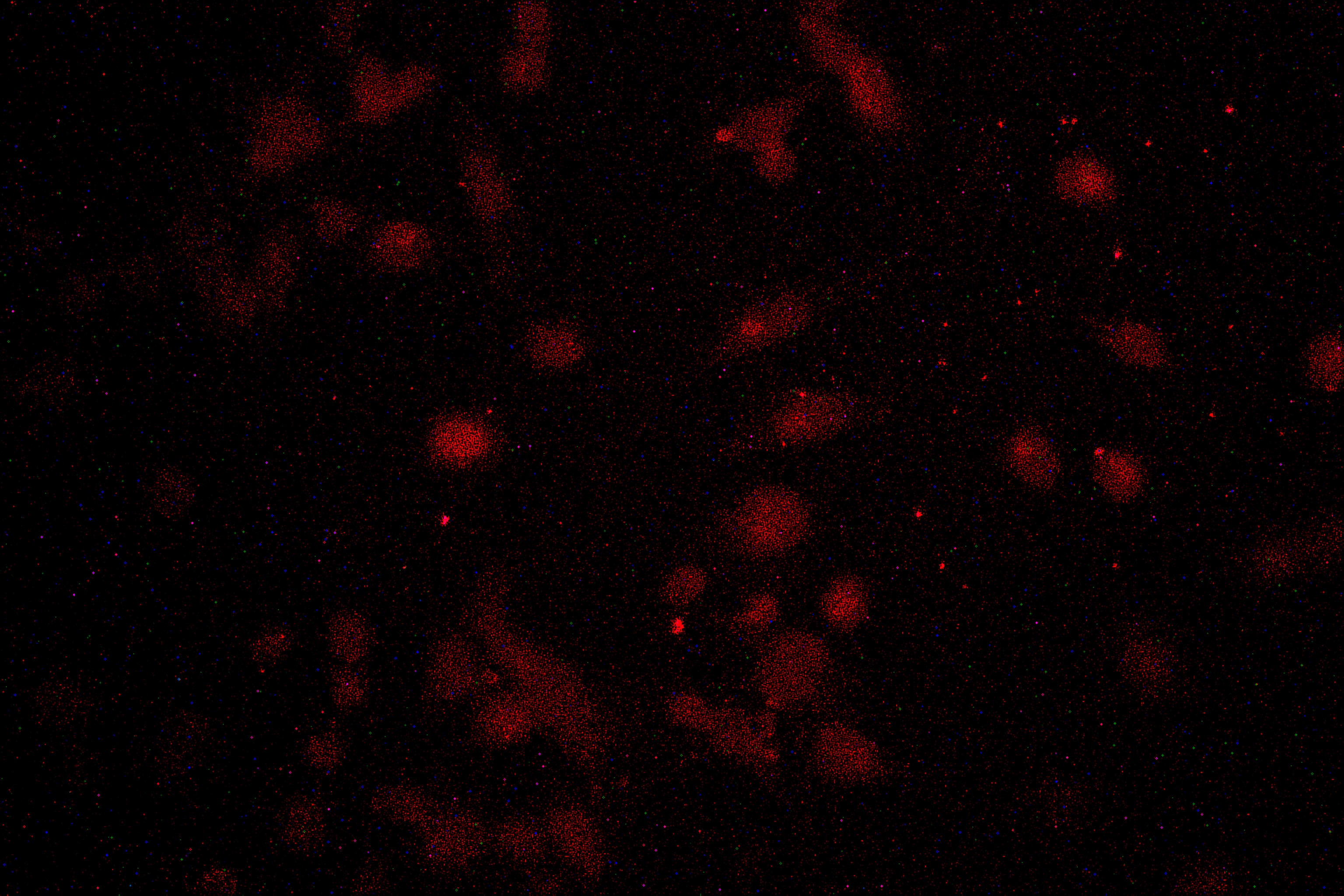

Supplement: Supplemental Information 2 [file peerj-11-14608-s002.zip › micrograph Figure1 CD80/MO-vitexin/1-1.jpg]

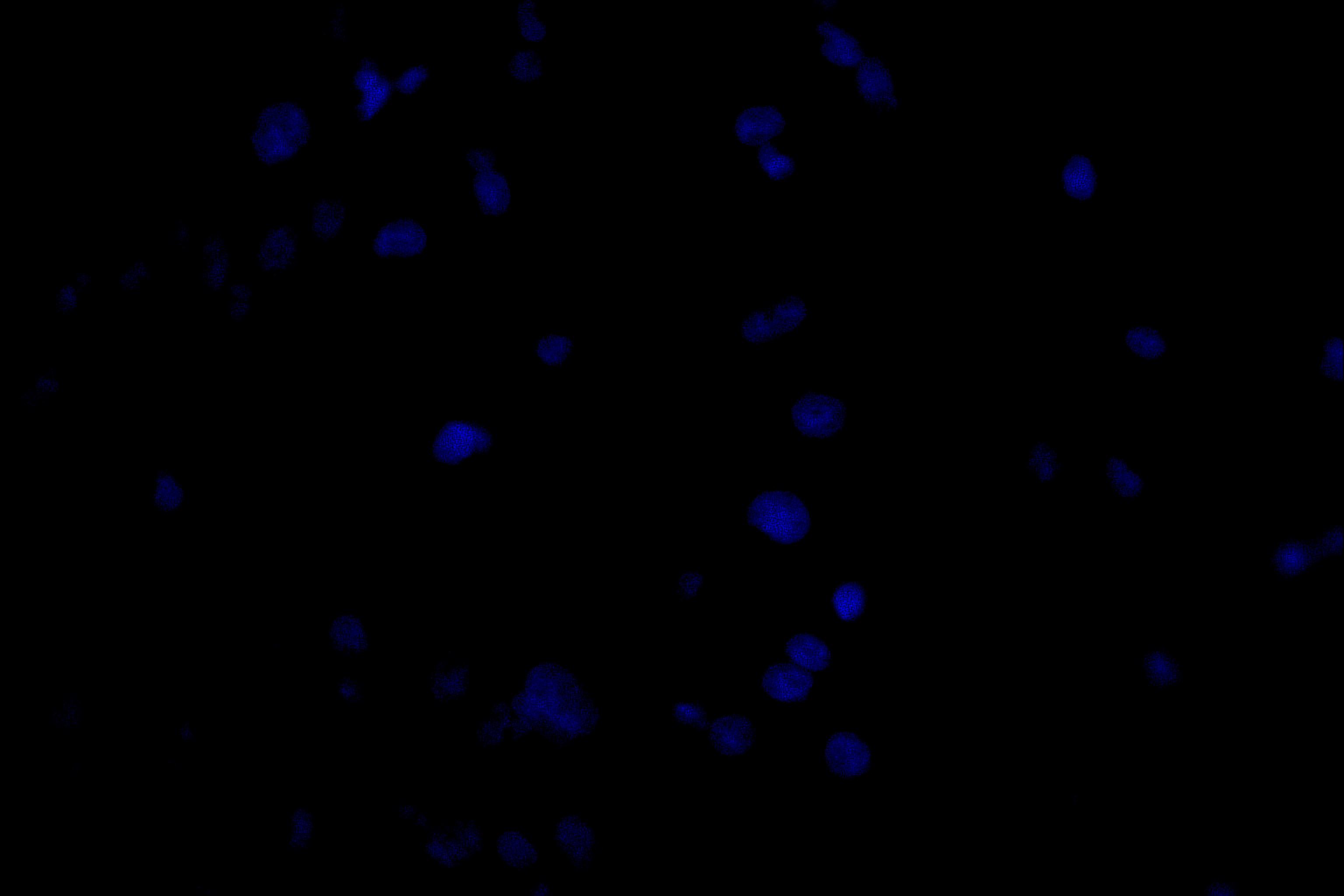

Supplement: Supplemental Information 2 [file peerj-11-14608-s002.zip › micrograph Figure1 CD80/MO-vitexin/1.jpg]

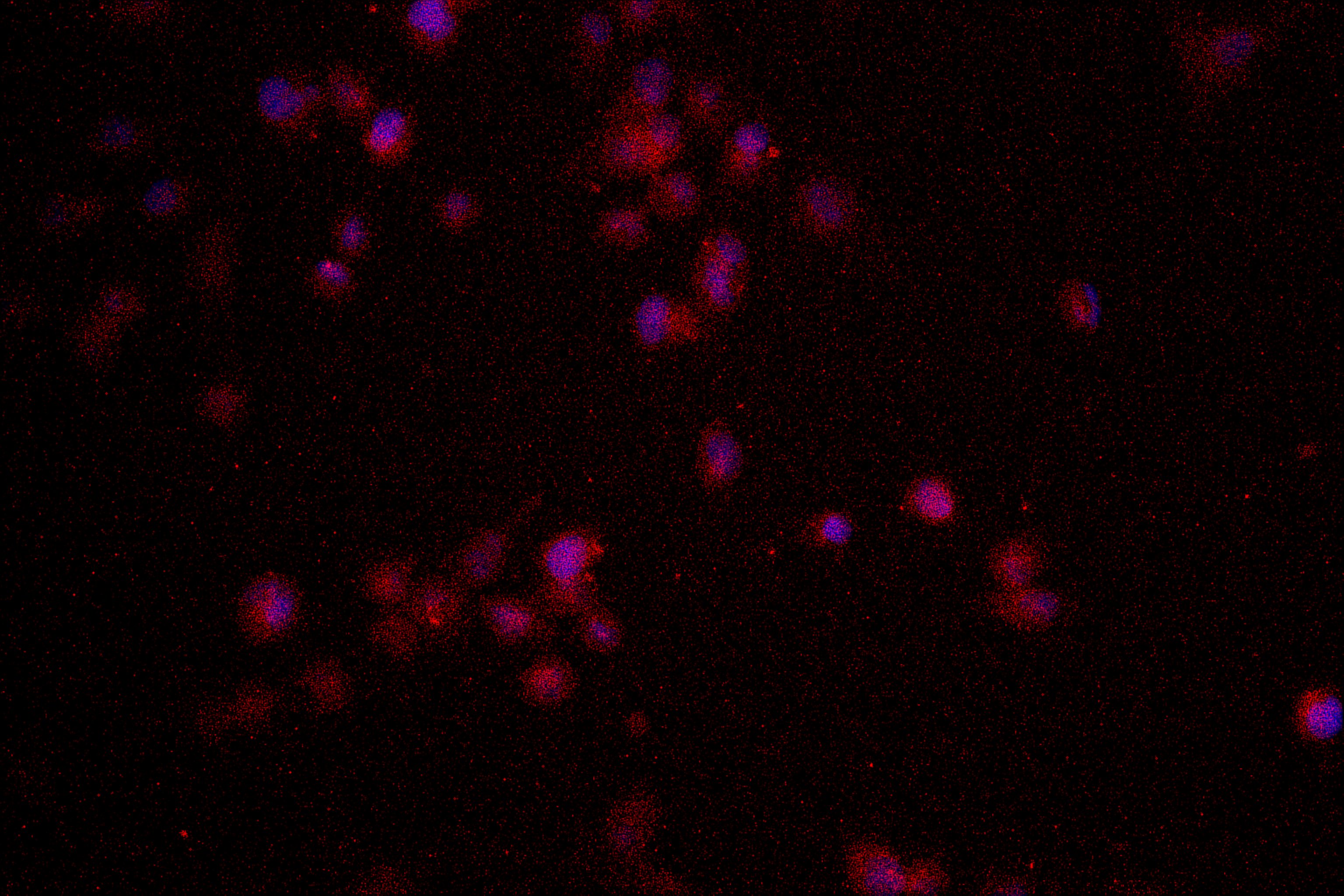

Supplement: Supplemental Information 2 [file peerj-11-14608-s002.zip › micrograph Figure1 CD80/MO-vitexin/2-2-2.jpg]

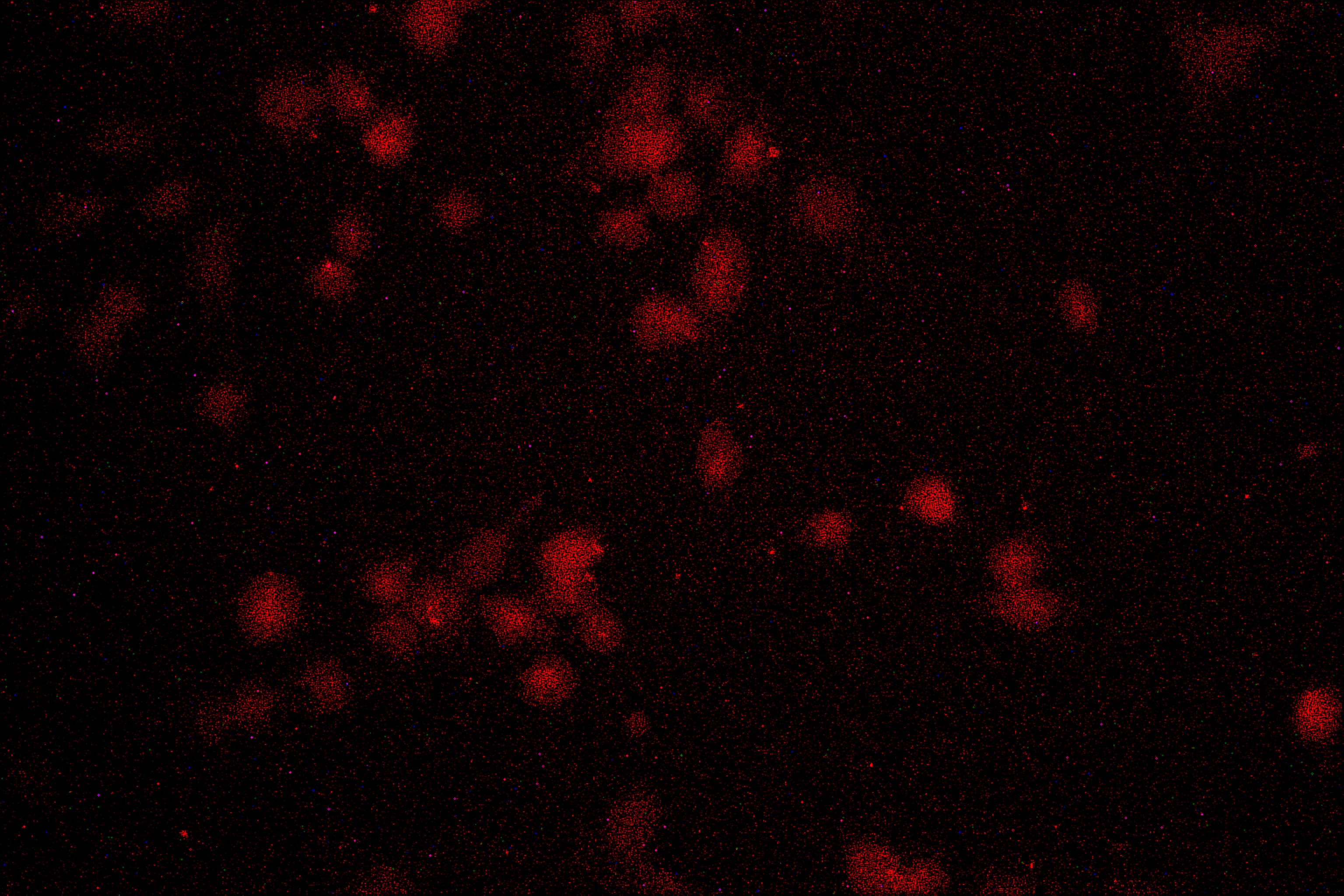

Supplement: Supplemental Information 2 [file peerj-11-14608-s002.zip › micrograph Figure1 CD80/MO-vitexin/2-2.jpg]

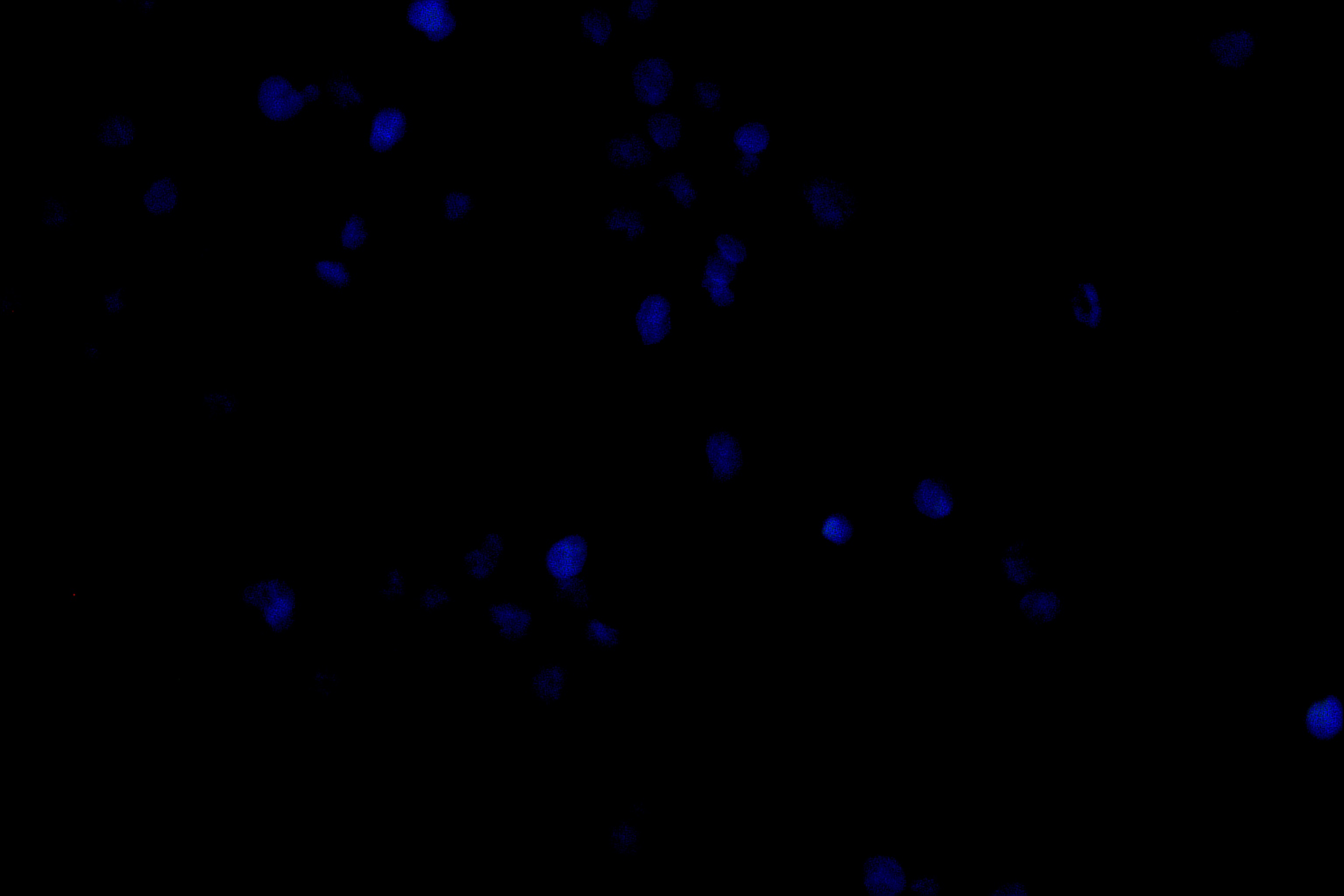

Supplement: Supplemental Information 2 [file peerj-11-14608-s002.zip › micrograph Figure1 CD80/MO-vitexin/2.jpg]

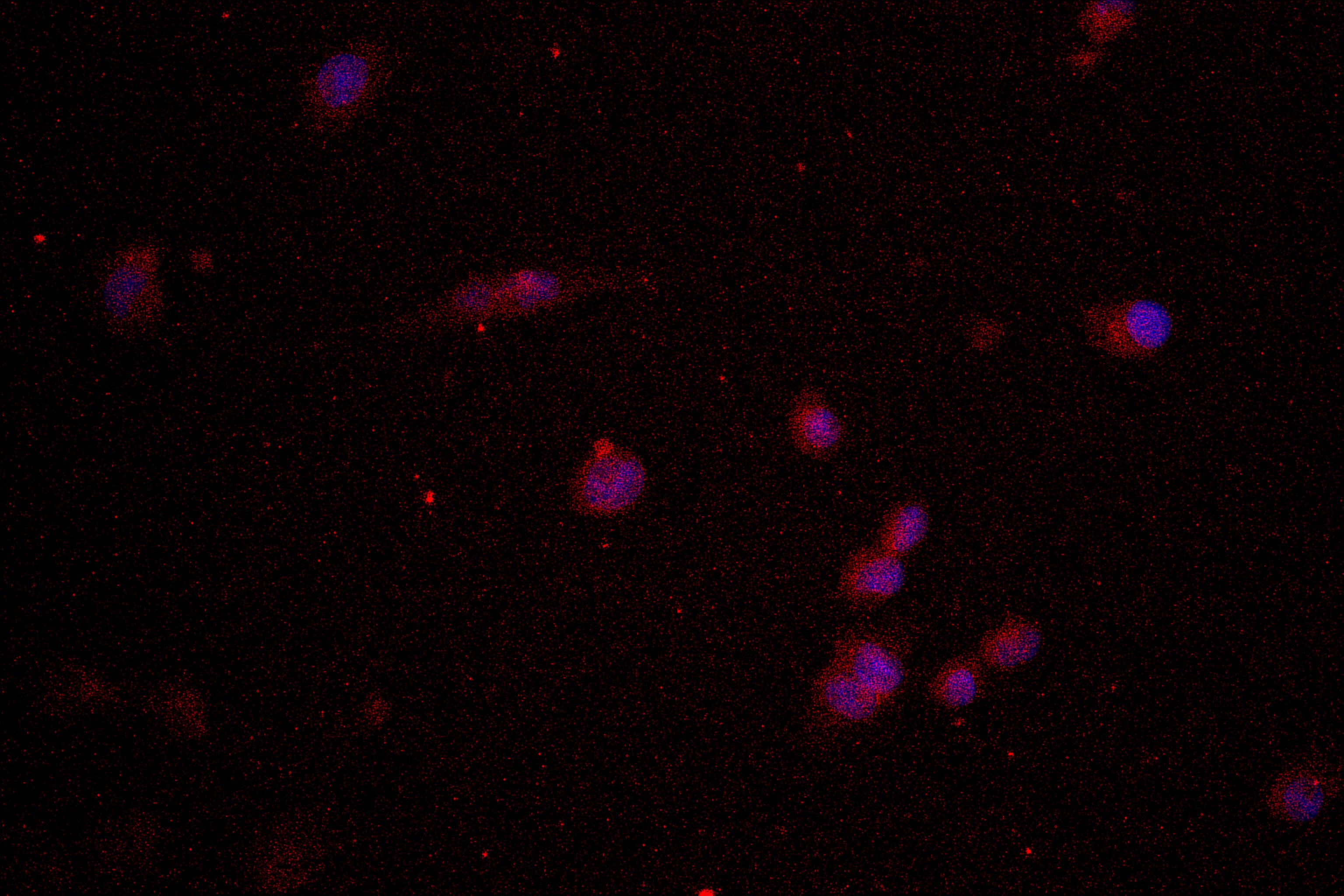

Supplement: Supplemental Information 2 [file peerj-11-14608-s002.zip › micrograph Figure1 CD80/MO-vitexin/3-3-3.jpg]

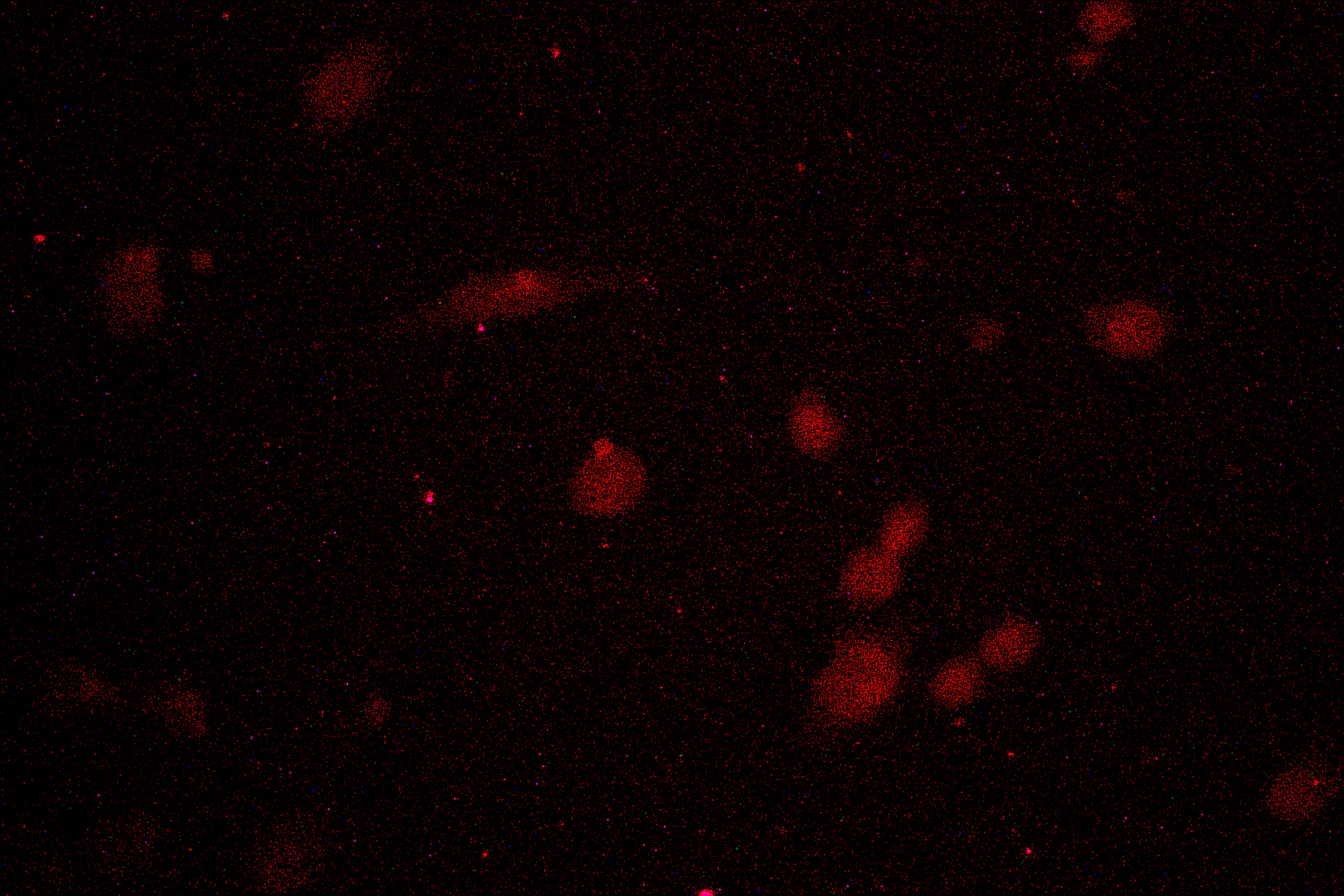

Supplement: Supplemental Information 2 [file peerj-11-14608-s002.zip › micrograph Figure1 CD80/MO-vitexin/3-3.jpg]

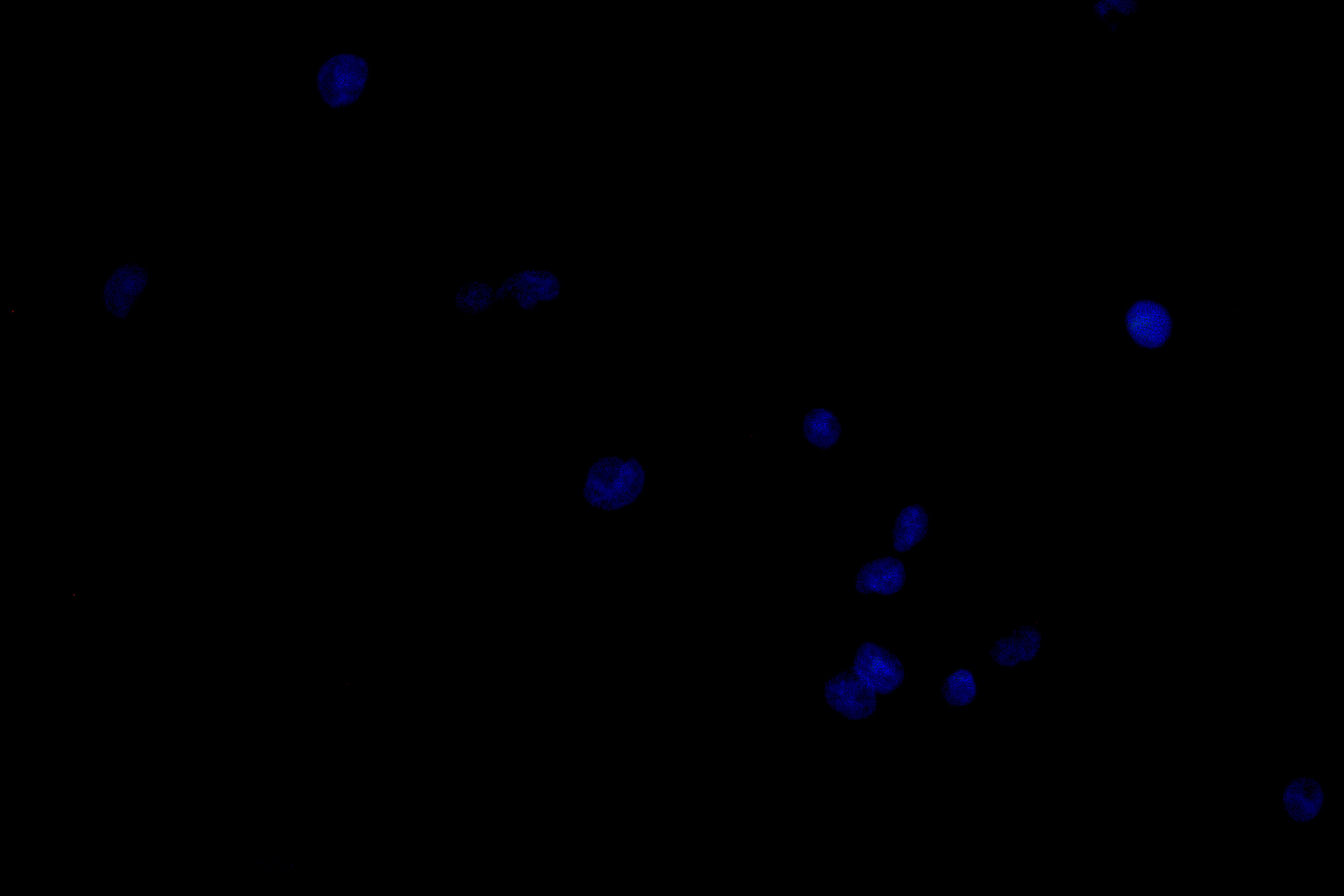

Supplement: Supplemental Information 2 [file peerj-11-14608-s002.zip › micrograph Figure1 CD80/MO-vitexin/3.jpg]

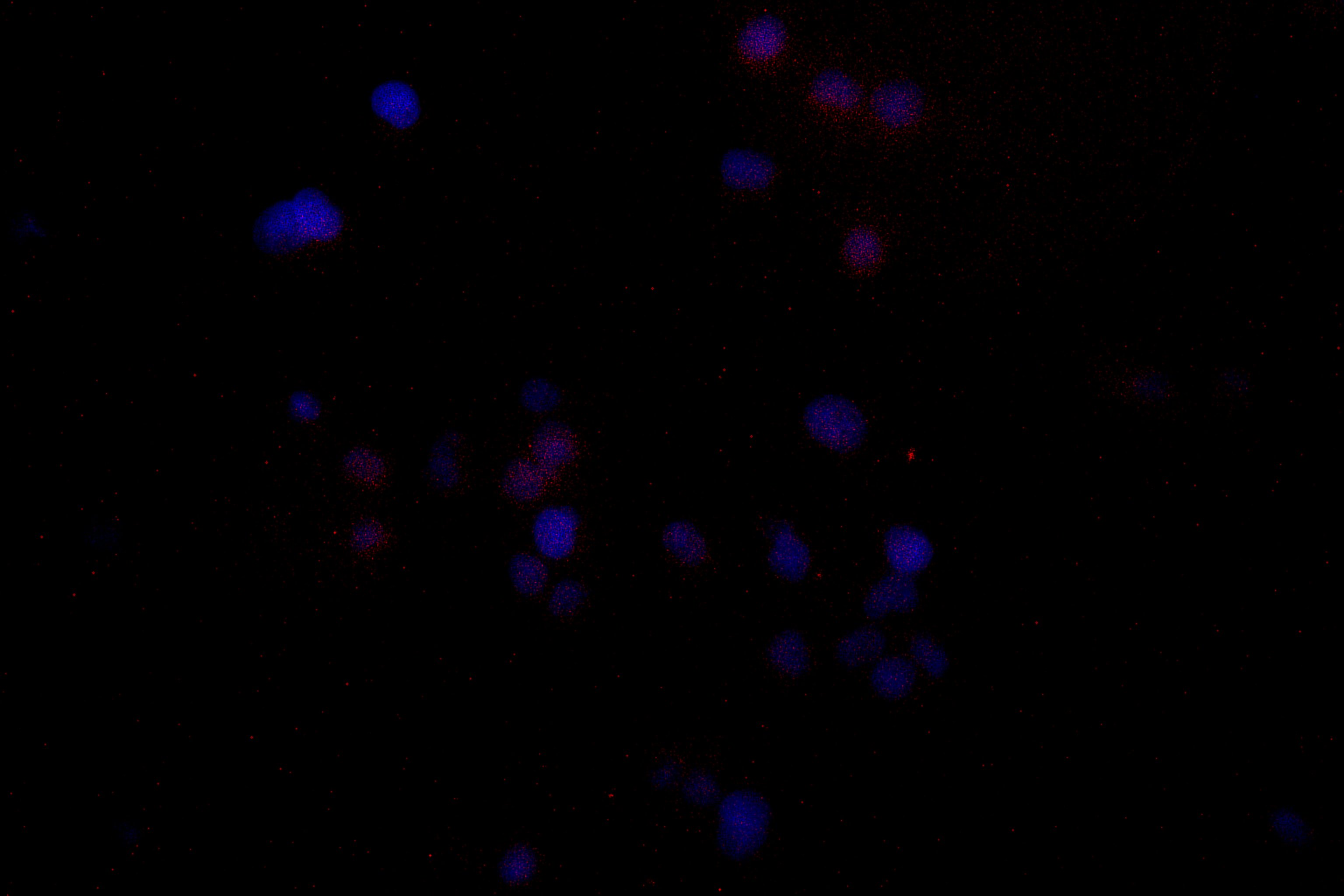

Supplement: Supplemental Information 3 [file peerj-11-14608-s003.zip › micrograph Figure1 CD86/MO-NC组/1-1-1.jpg]

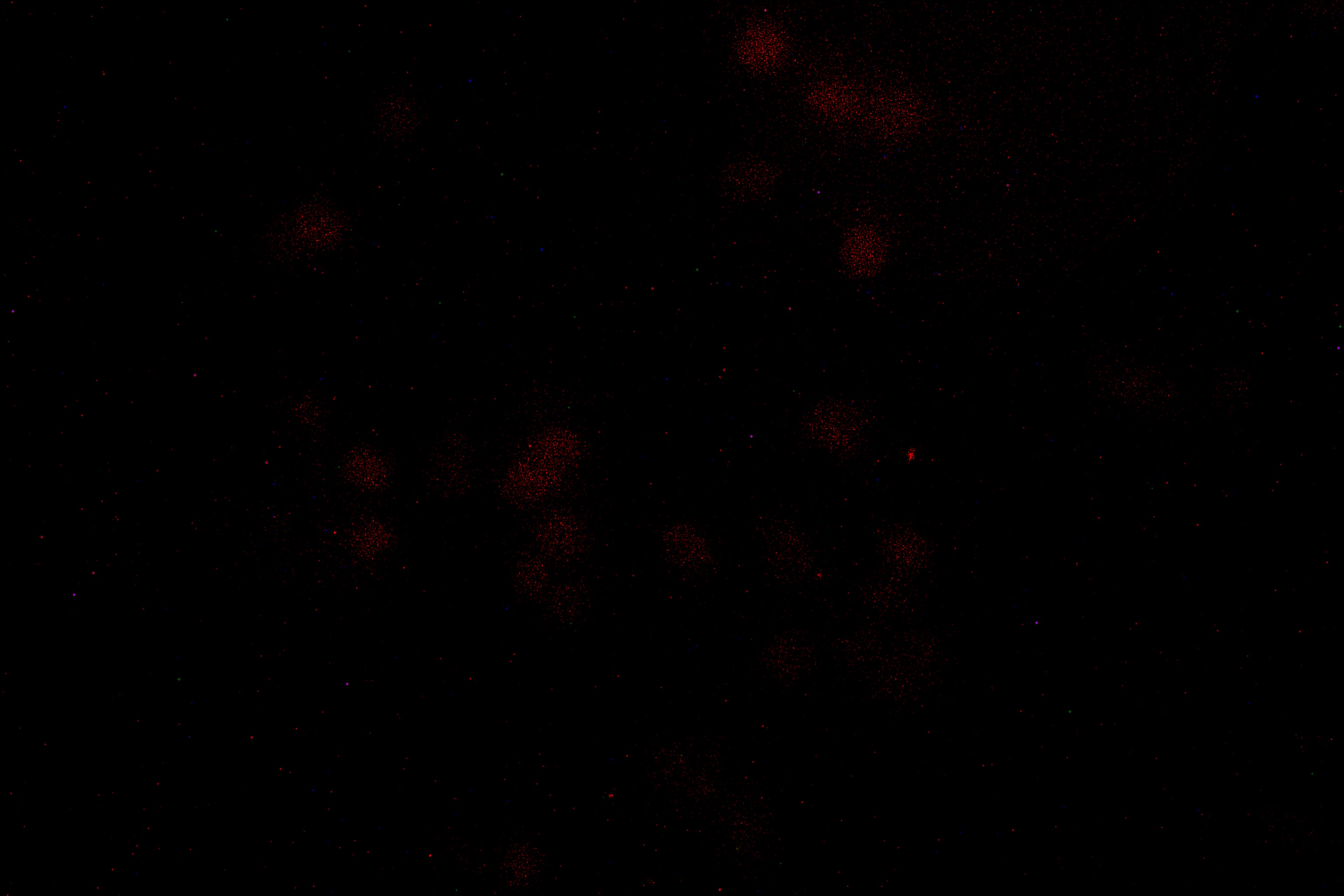

Supplement: Supplemental Information 3 [file peerj-11-14608-s003.zip › micrograph Figure1 CD86/MO-NC组/1-1.jpg]

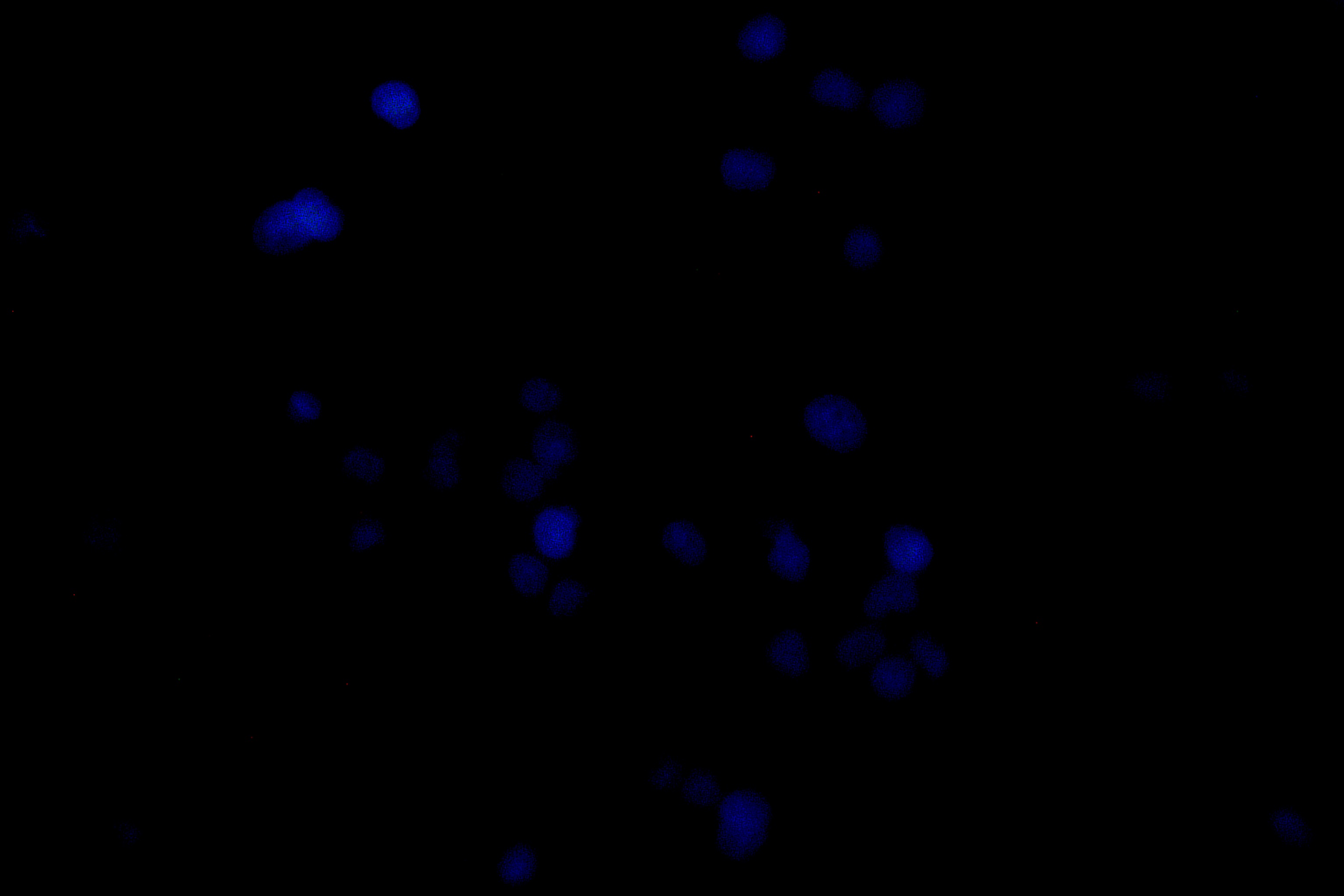

Supplement: Supplemental Information 3 [file peerj-11-14608-s003.zip › micrograph Figure1 CD86/MO-NC组/1.jpg]

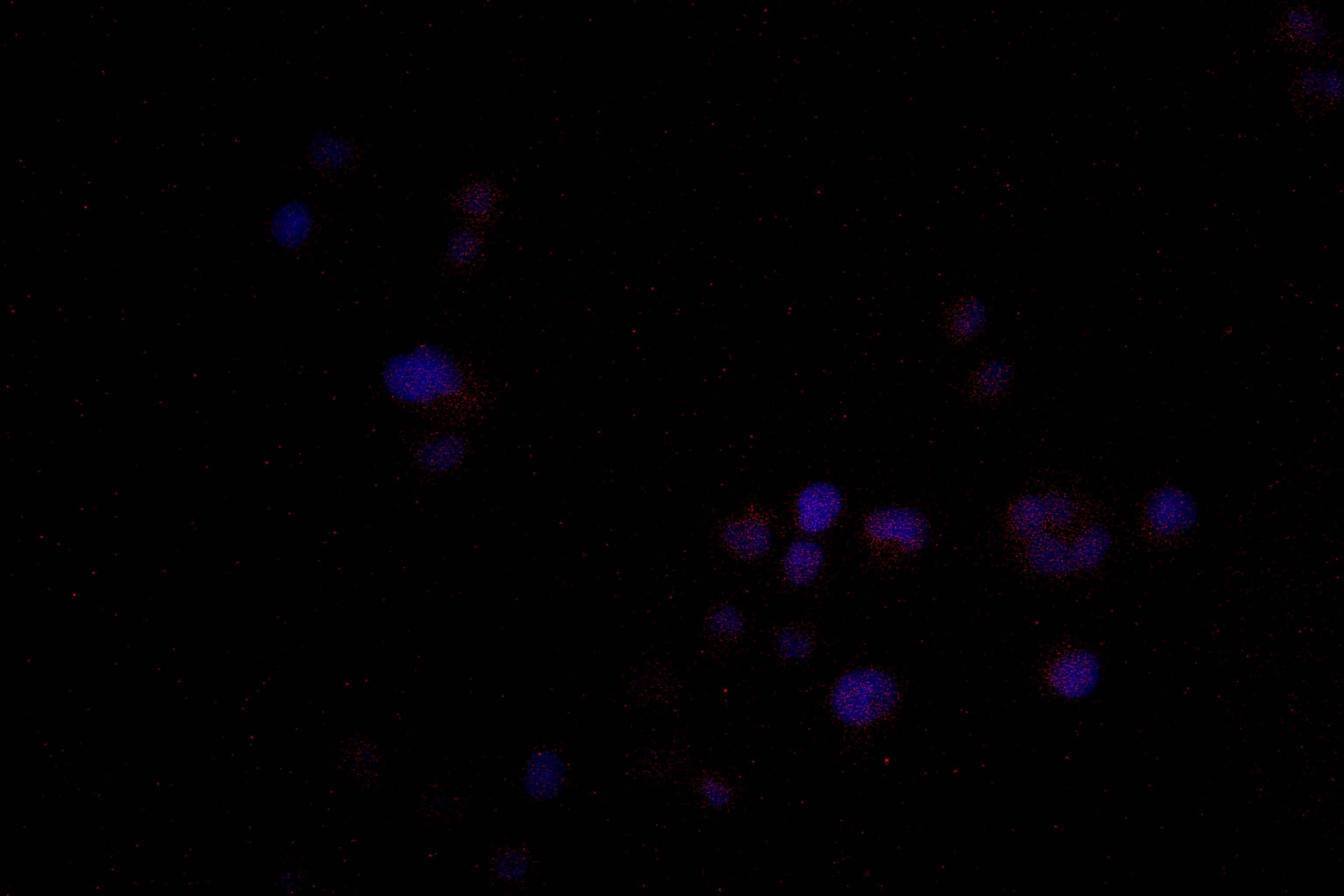

Supplement: Supplemental Information 3 [file peerj-11-14608-s003.zip › micrograph Figure1 CD86/MO-NC组/2-2-2.jpg]

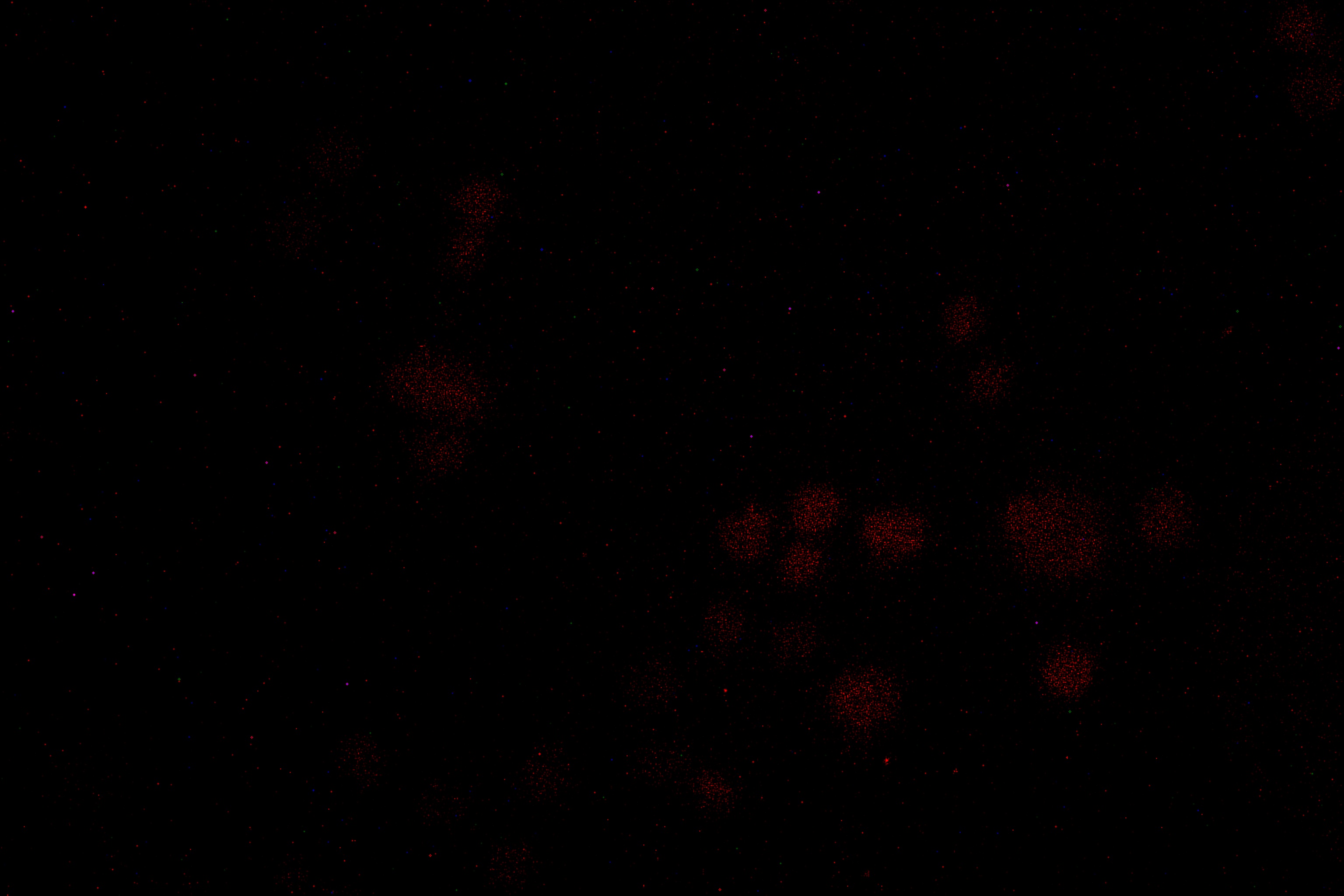

Supplement: Supplemental Information 3 [file peerj-11-14608-s003.zip › micrograph Figure1 CD86/MO-NC组/2-2.jpg]

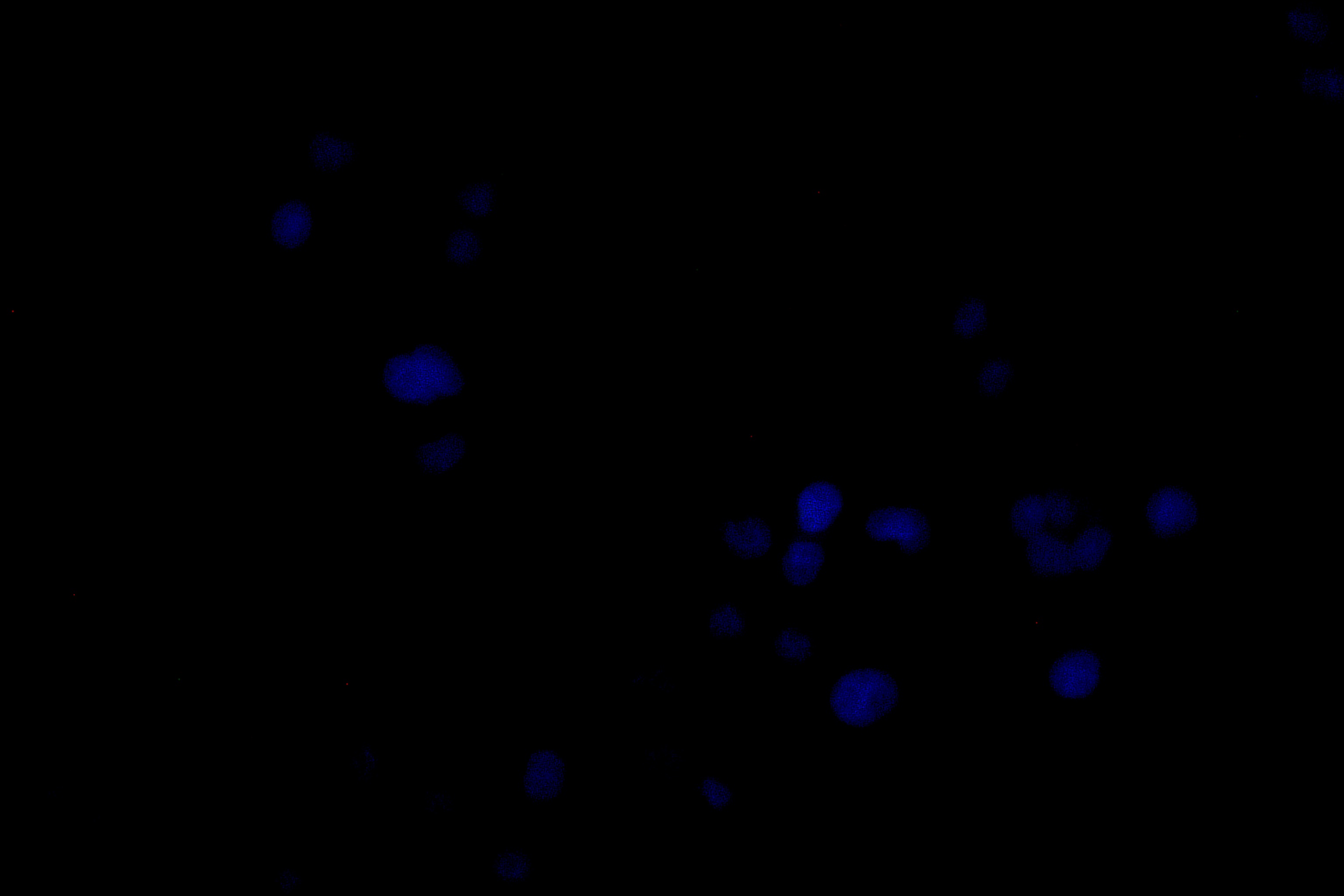

Supplement: Supplemental Information 3 [file peerj-11-14608-s003.zip › micrograph Figure1 CD86/MO-NC组/2.jpg]

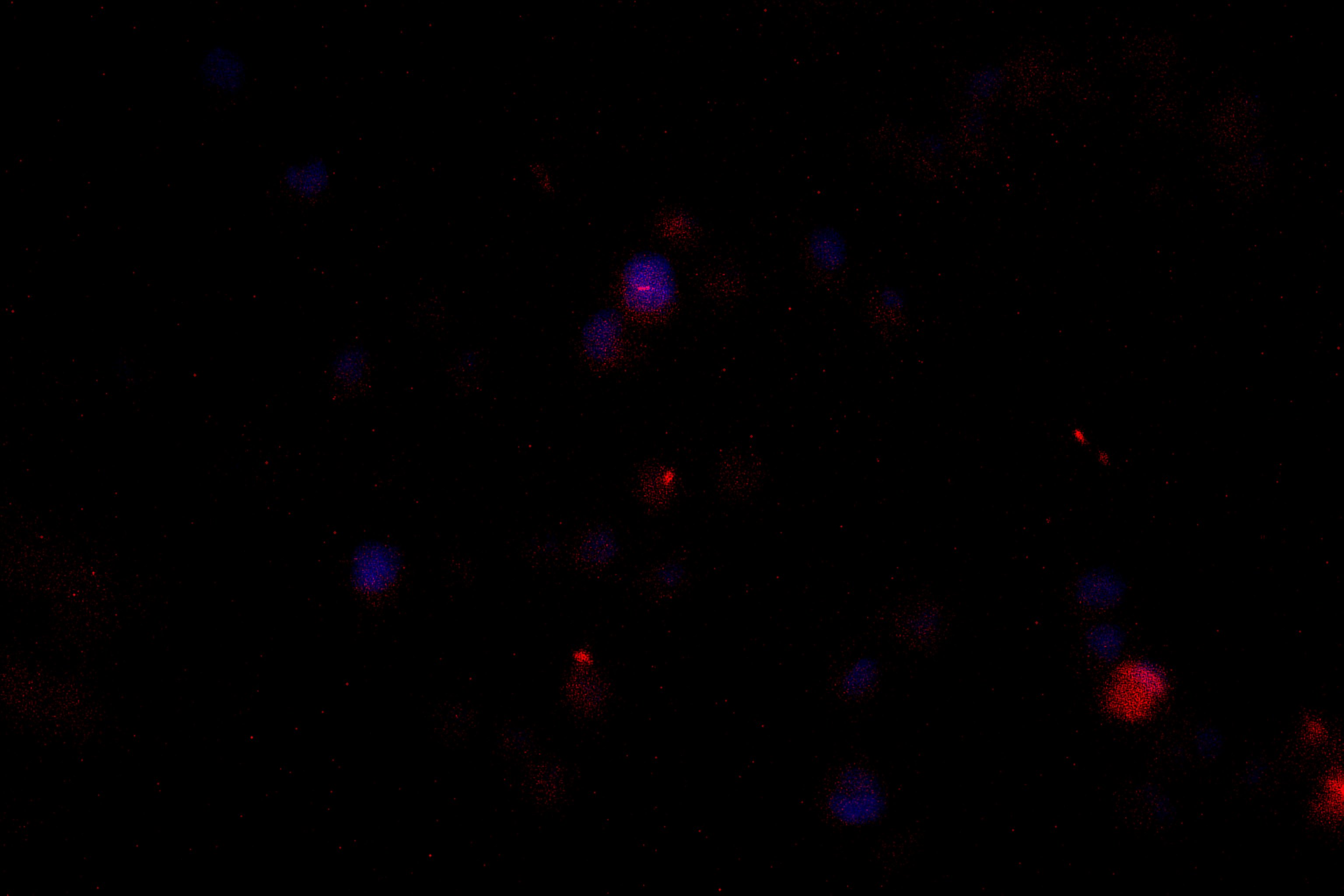

Supplement: Supplemental Information 3 [file peerj-11-14608-s003.zip › micrograph Figure1 CD86/MO-NC组/3-3-3.jpg]

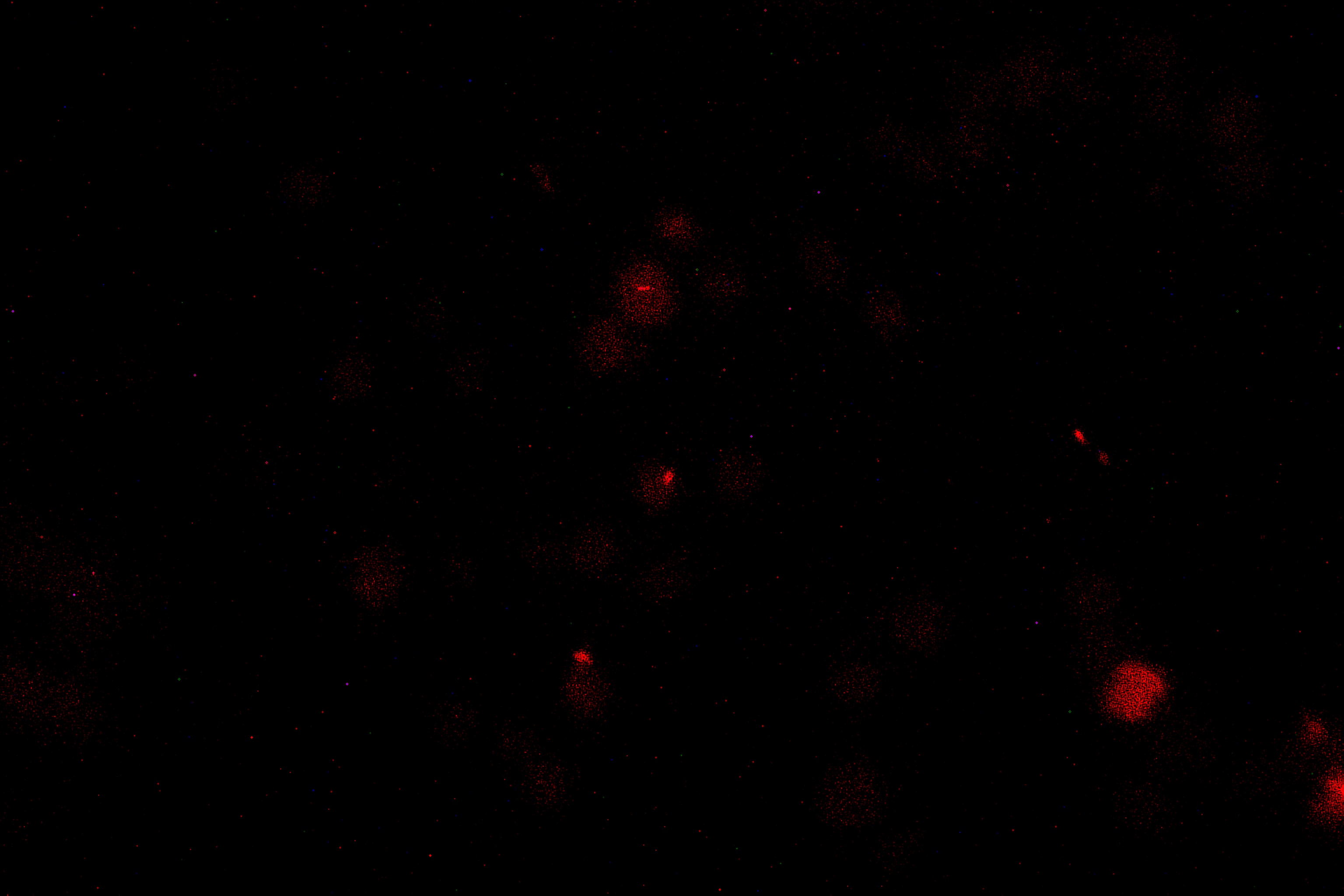

Supplement: Supplemental Information 3 [file peerj-11-14608-s003.zip › micrograph Figure1 CD86/MO-NC组/3-3.jpg]

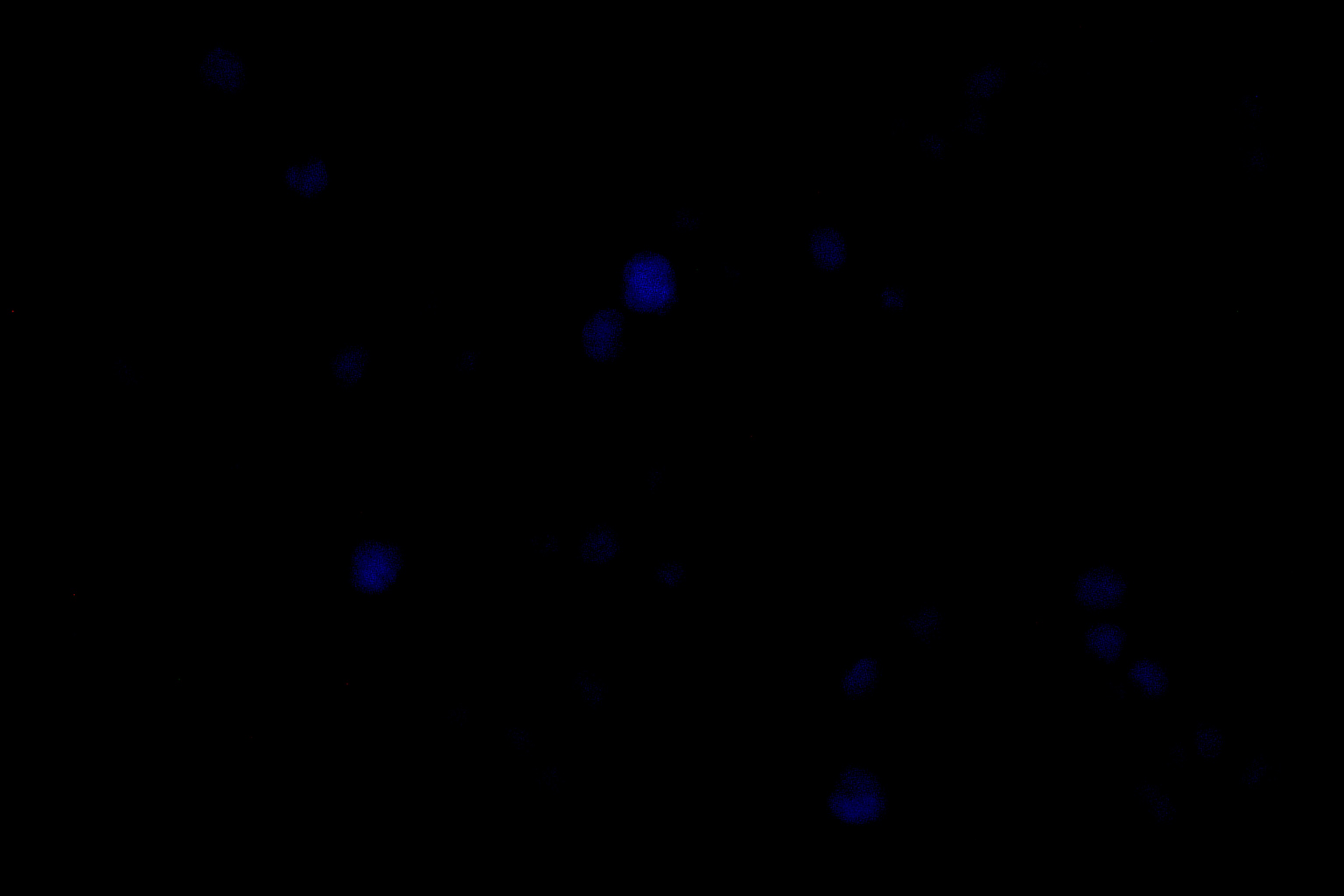

Supplement: Supplemental Information 3 [file peerj-11-14608-s003.zip › micrograph Figure1 CD86/MO-NC组/3.jpg]

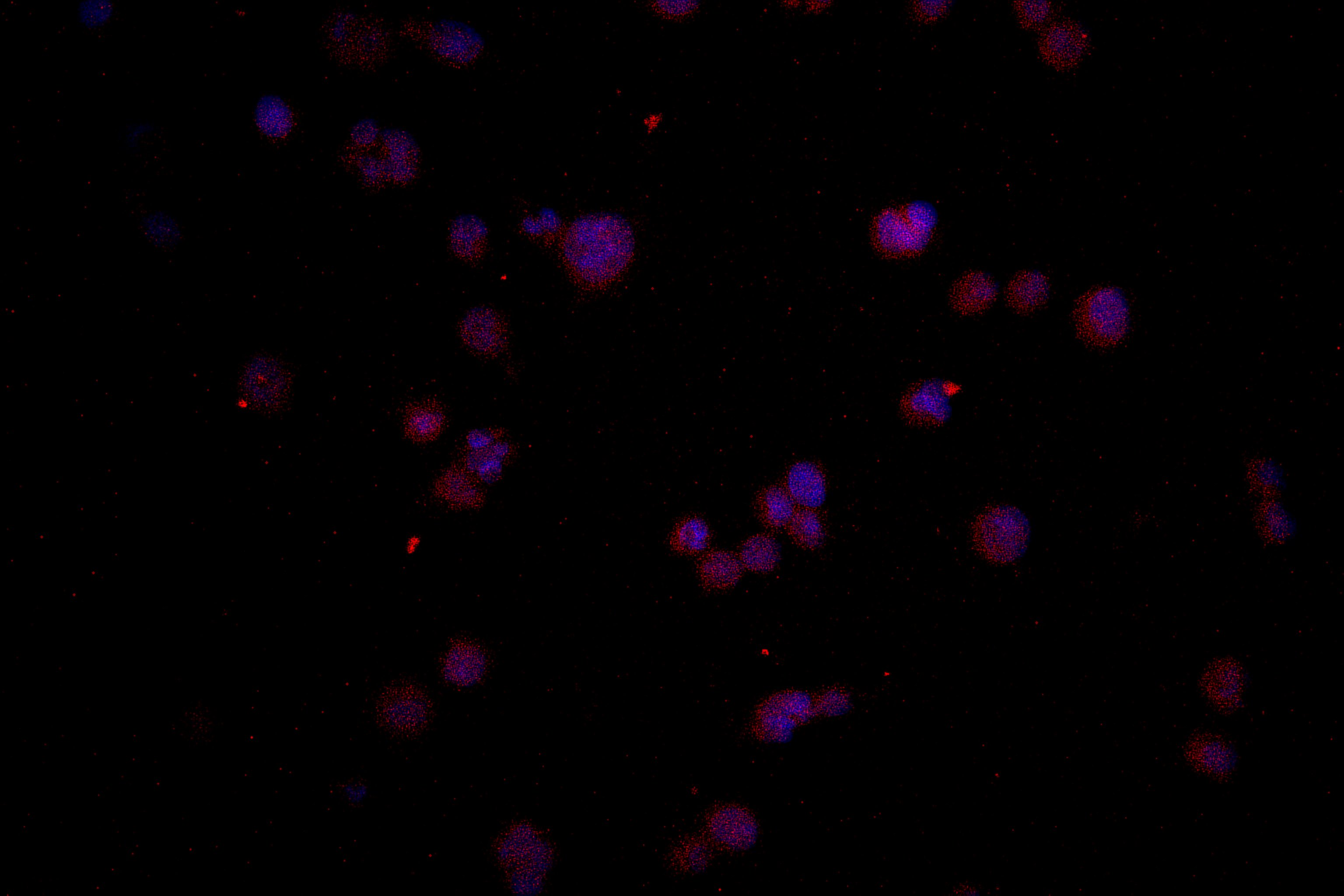

Supplement: Supplemental Information 3 [file peerj-11-14608-s003.zip › micrograph Figure1 CD86/MO-vitexin/1-1-1.jpg]

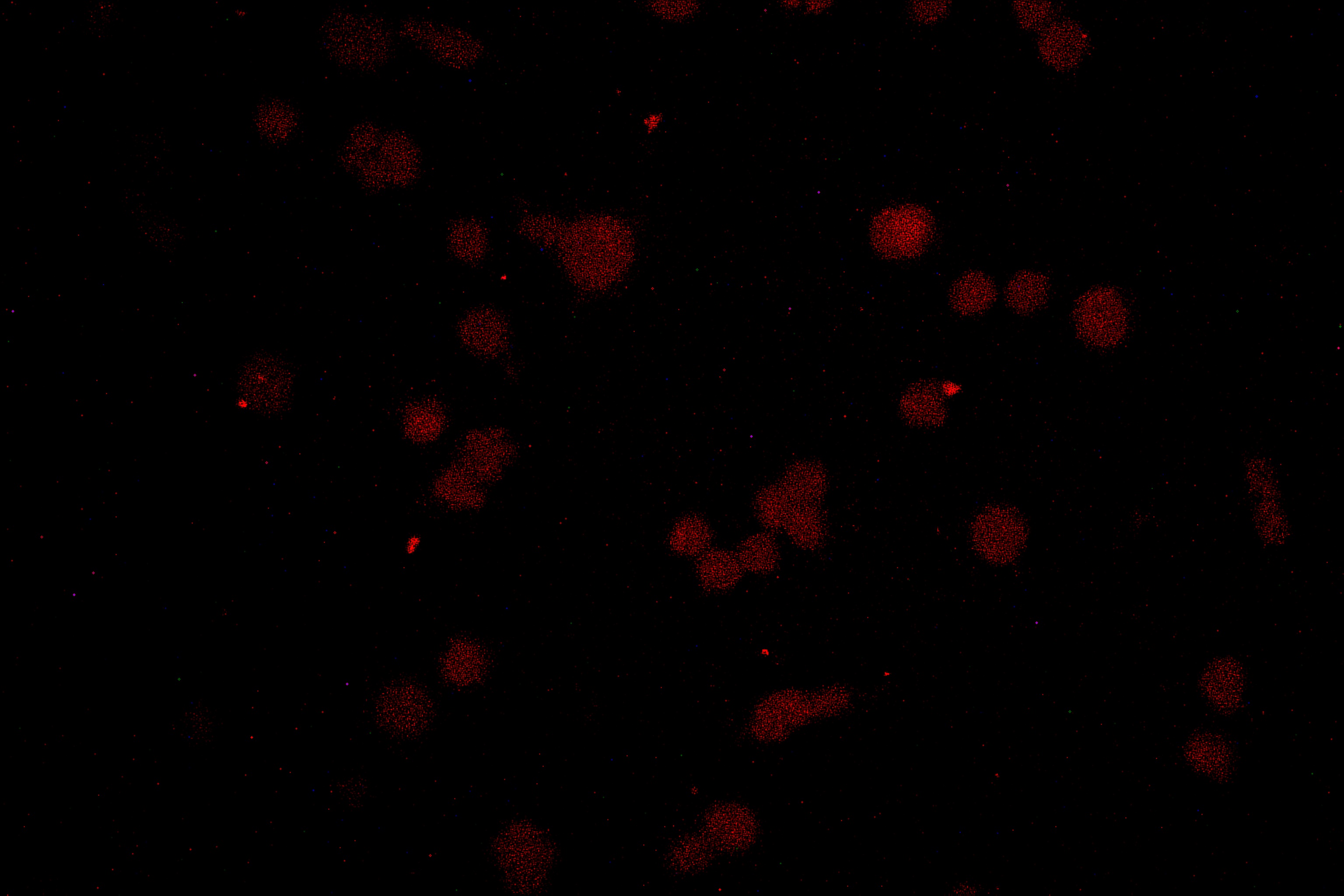

Supplement: Supplemental Information 3 [file peerj-11-14608-s003.zip › micrograph Figure1 CD86/MO-vitexin/1-1.jpg]

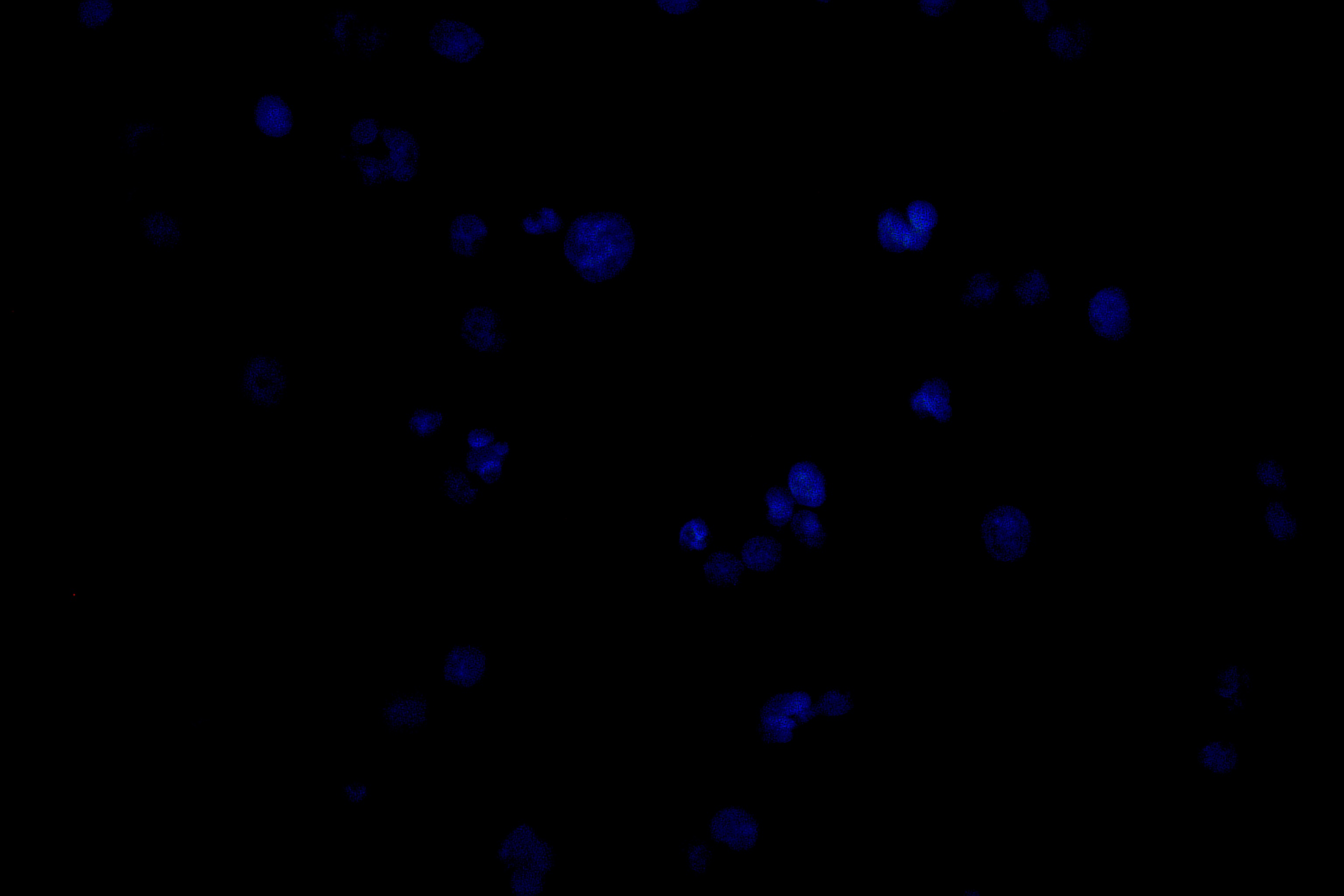

Supplement: Supplemental Information 3 [file peerj-11-14608-s003.zip › micrograph Figure1 CD86/MO-vitexin/1.jpg]

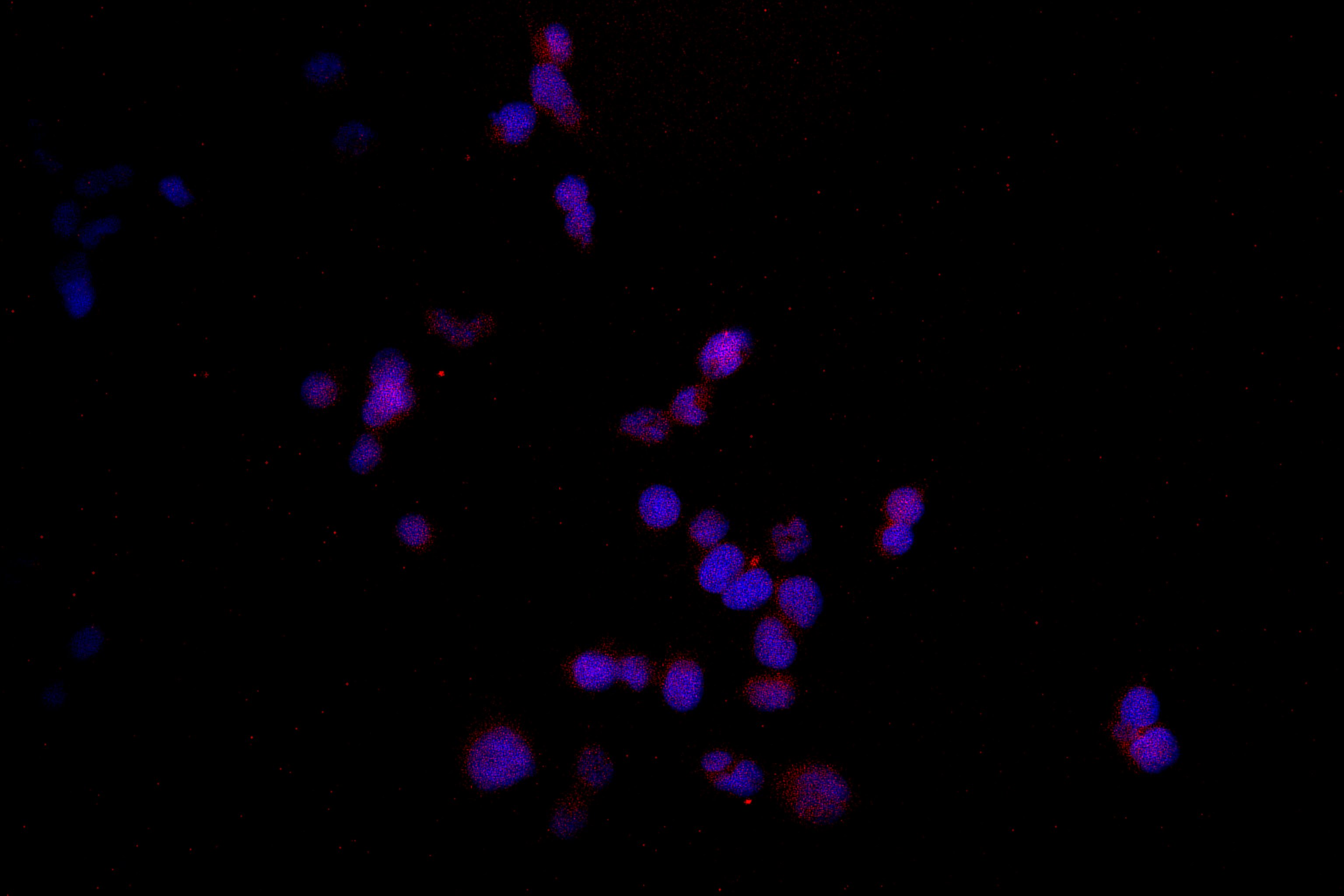

Supplement: Supplemental Information 3 [file peerj-11-14608-s003.zip › micrograph Figure1 CD86/MO-vitexin/2-2-2.jpg]

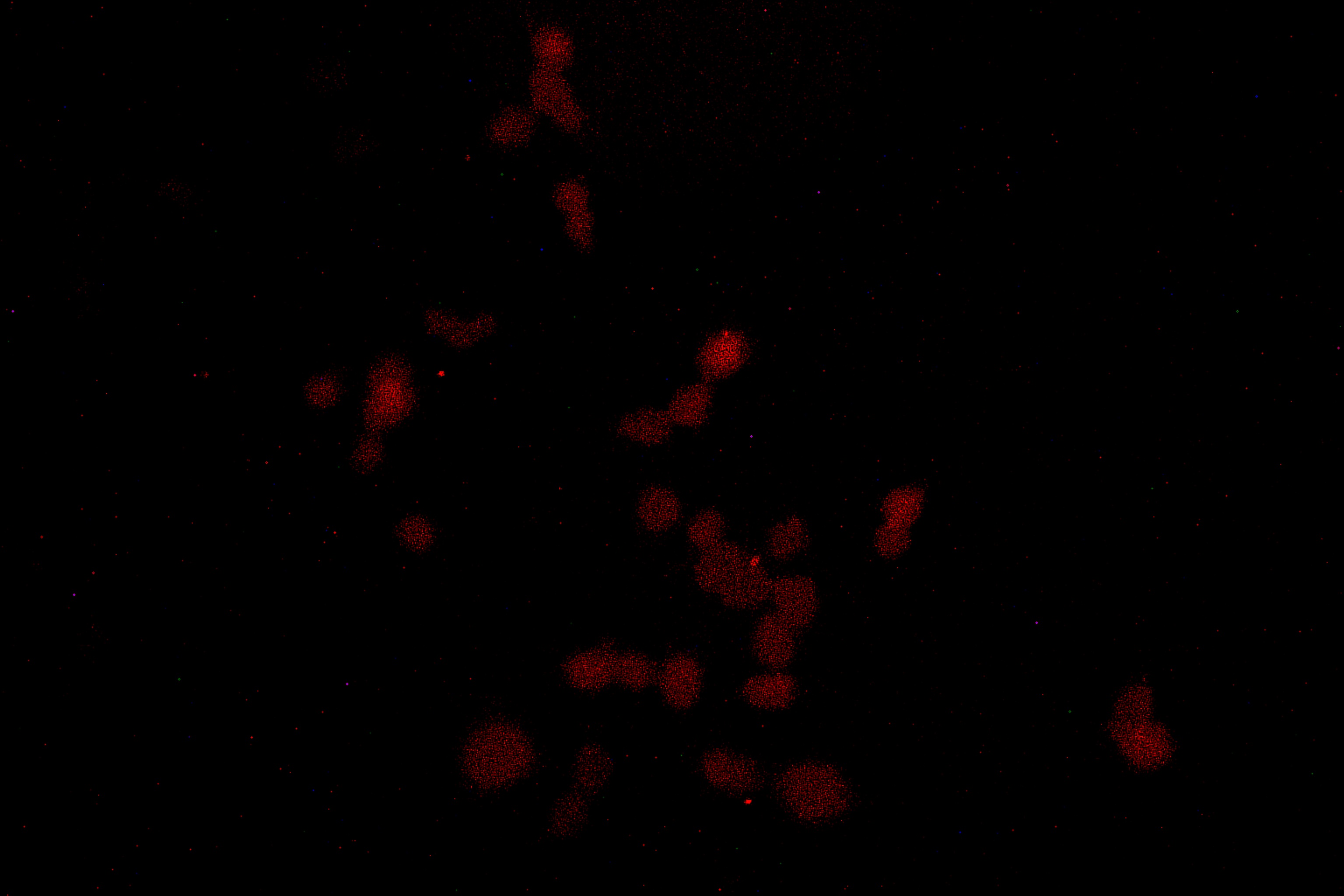

Supplement: Supplemental Information 3 [file peerj-11-14608-s003.zip › micrograph Figure1 CD86/MO-vitexin/2-2.jpg]

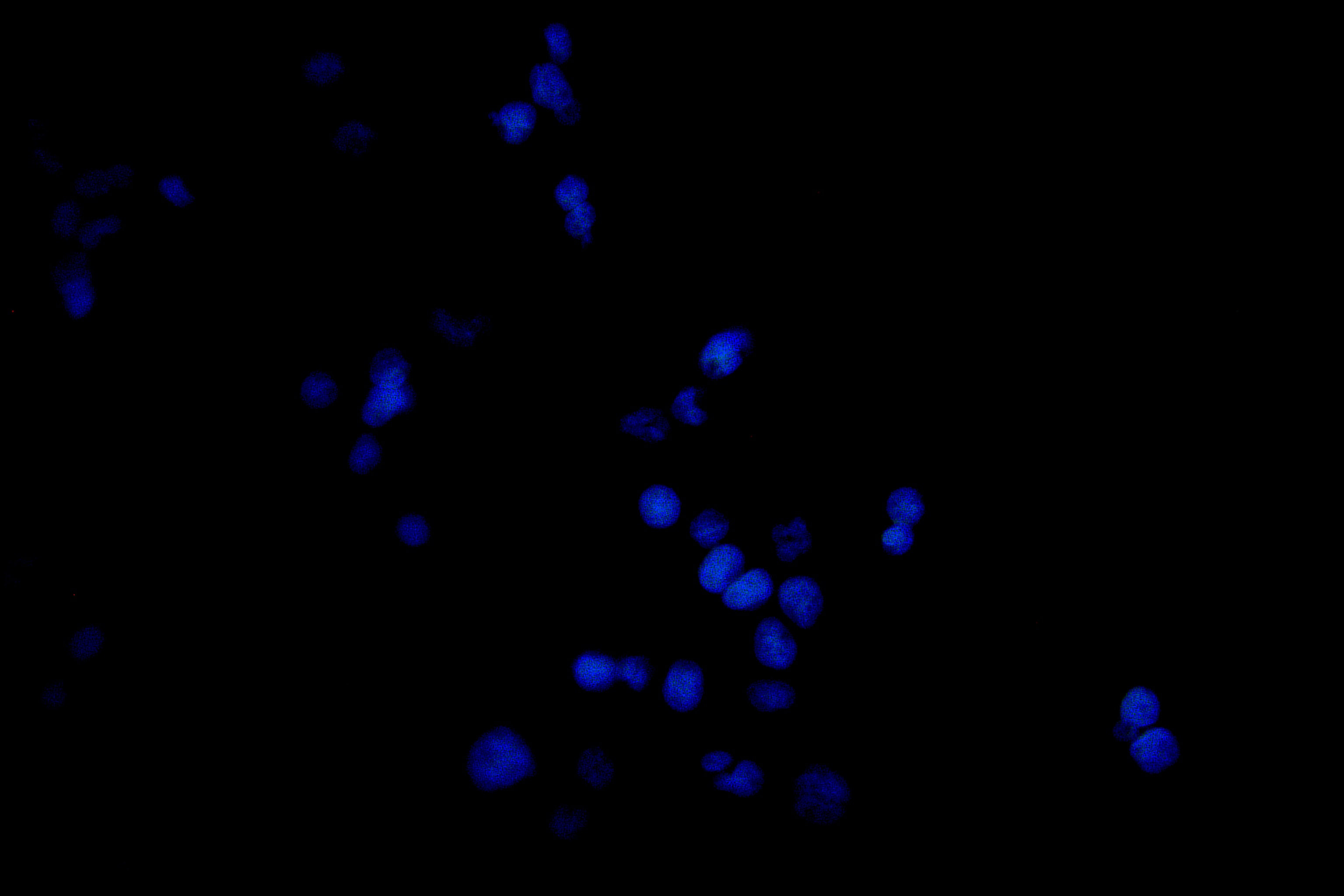

Supplement: Supplemental Information 3 [file peerj-11-14608-s003.zip › micrograph Figure1 CD86/MO-vitexin/2.jpg]

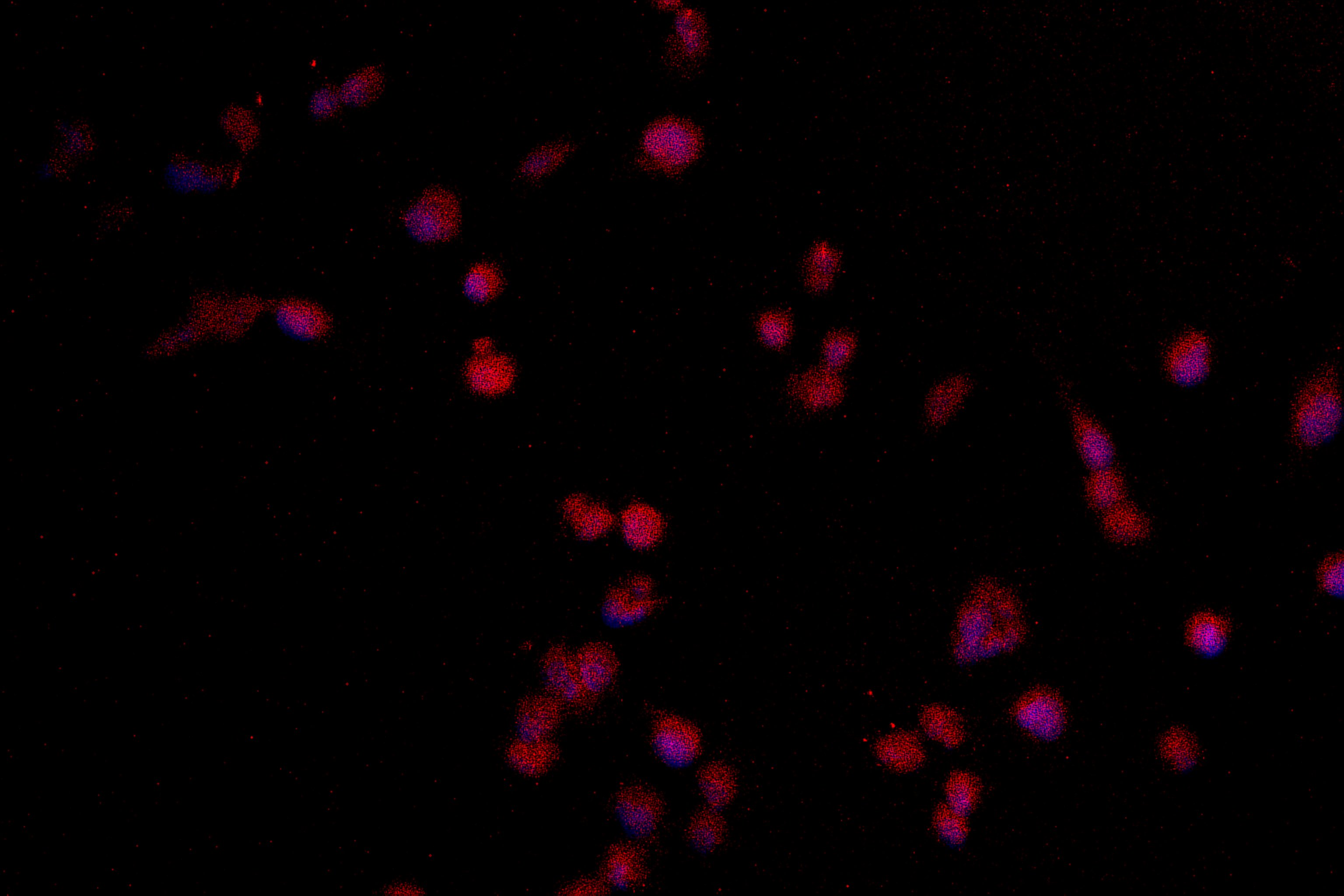

Supplement: Supplemental Information 3 [file peerj-11-14608-s003.zip › micrograph Figure1 CD86/MO-vitexin/3-3-3.jpg]

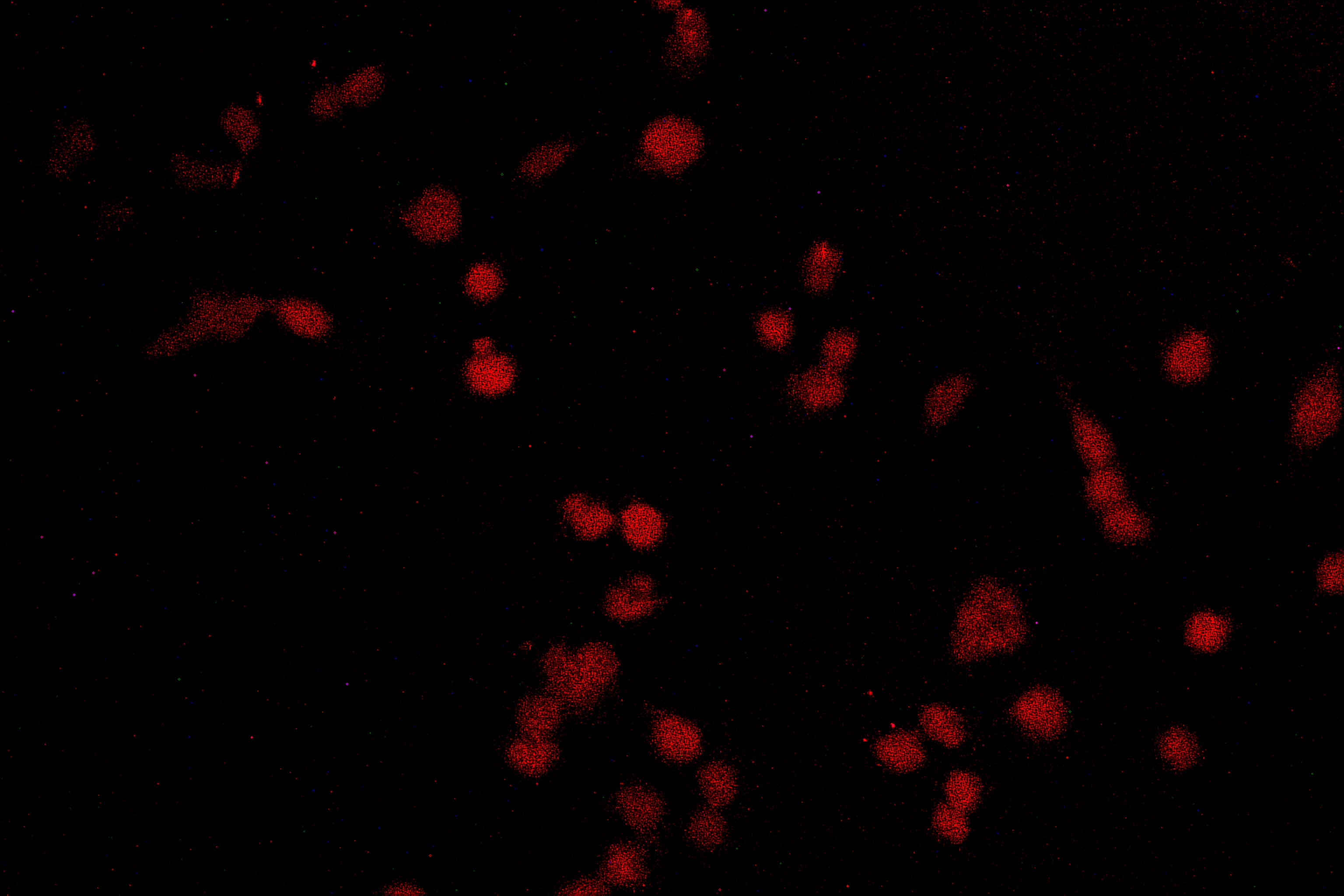

Supplement: Supplemental Information 3 [file peerj-11-14608-s003.zip › micrograph Figure1 CD86/MO-vitexin/3-3.jpg]

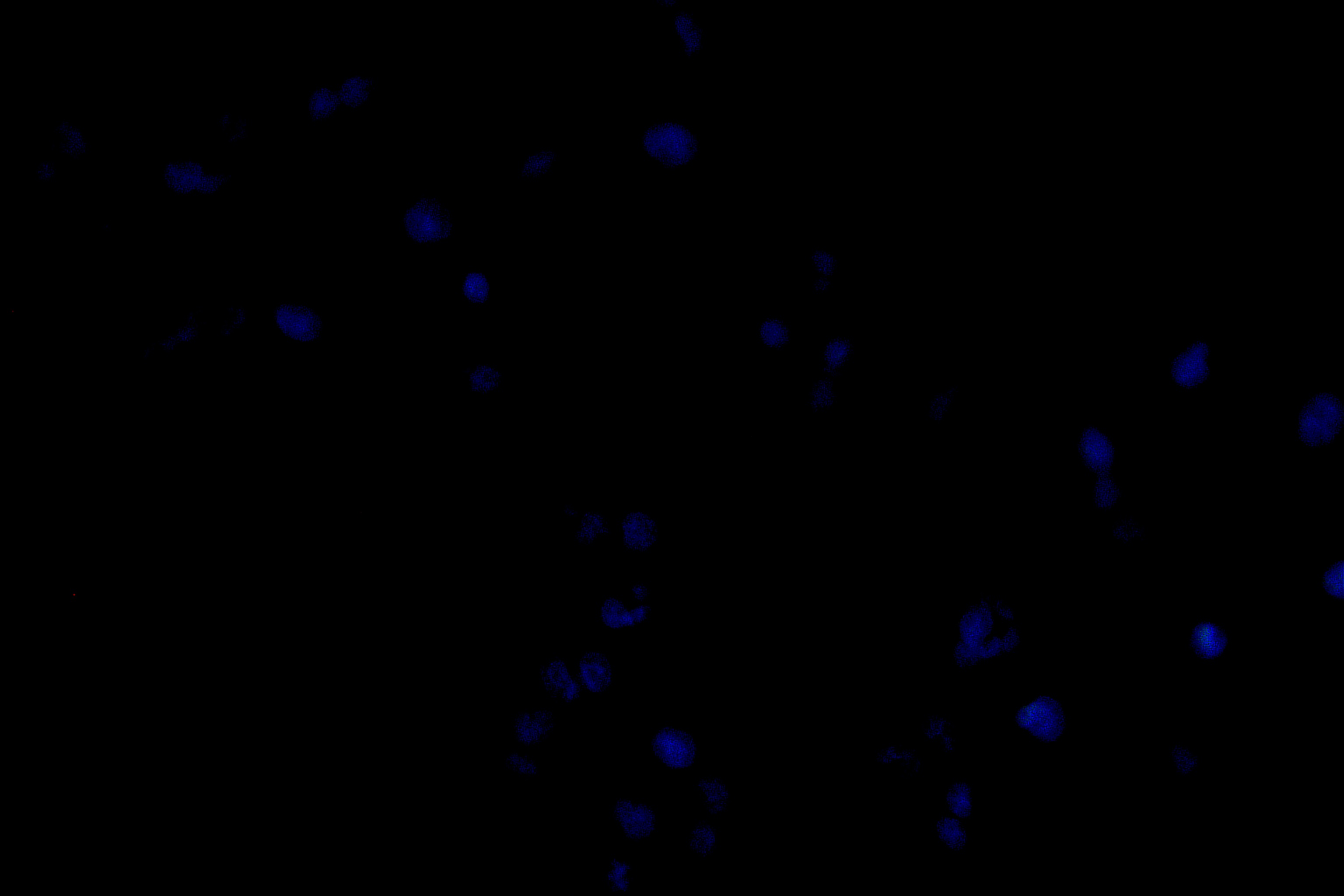

Supplement: Supplemental Information 3 [file peerj-11-14608-s003.zip › micrograph Figure1 CD86/MO-vitexin/3.jpg]

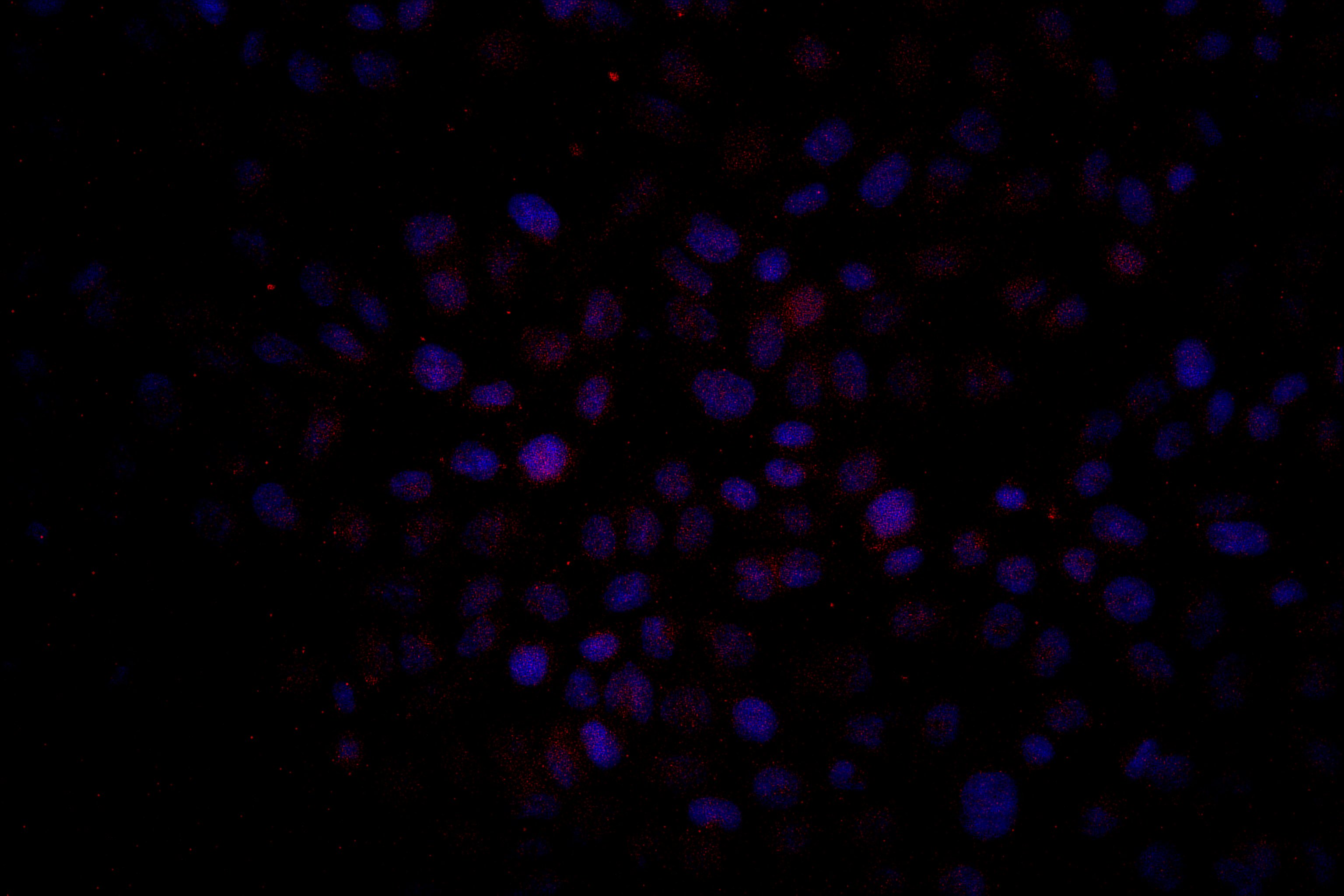

Supplement: Supplemental Information 4 [file peerj-11-14608-s004.zip › micrograph Figure1 CD1632/MO-NC/1-1-1.jpg]

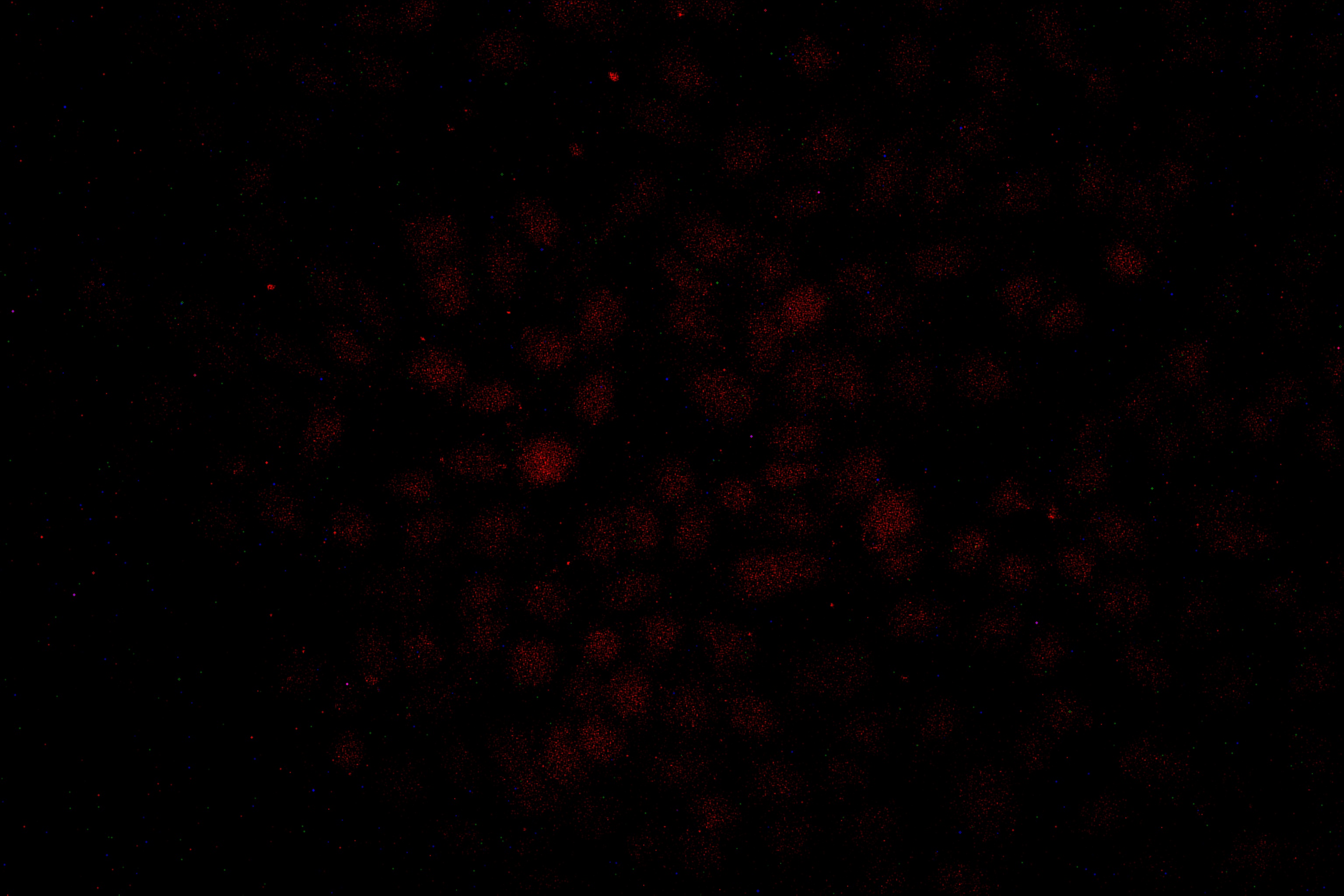

Supplement: Supplemental Information 4 [file peerj-11-14608-s004.zip › micrograph Figure1 CD1632/MO-NC/1-1.jpg]

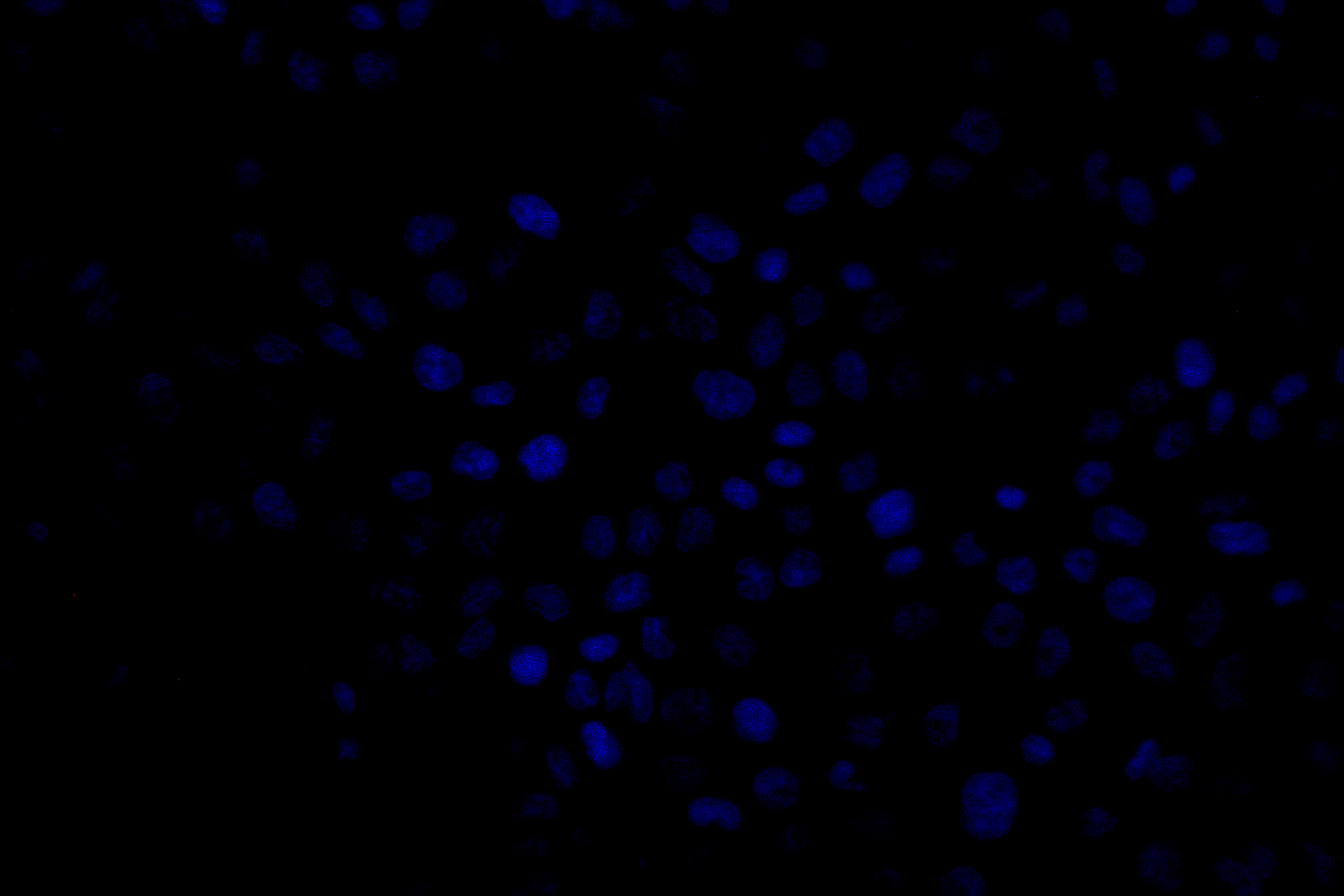

Supplement: Supplemental Information 4 [file peerj-11-14608-s004.zip › micrograph Figure1 CD1632/MO-NC/1.jpg]

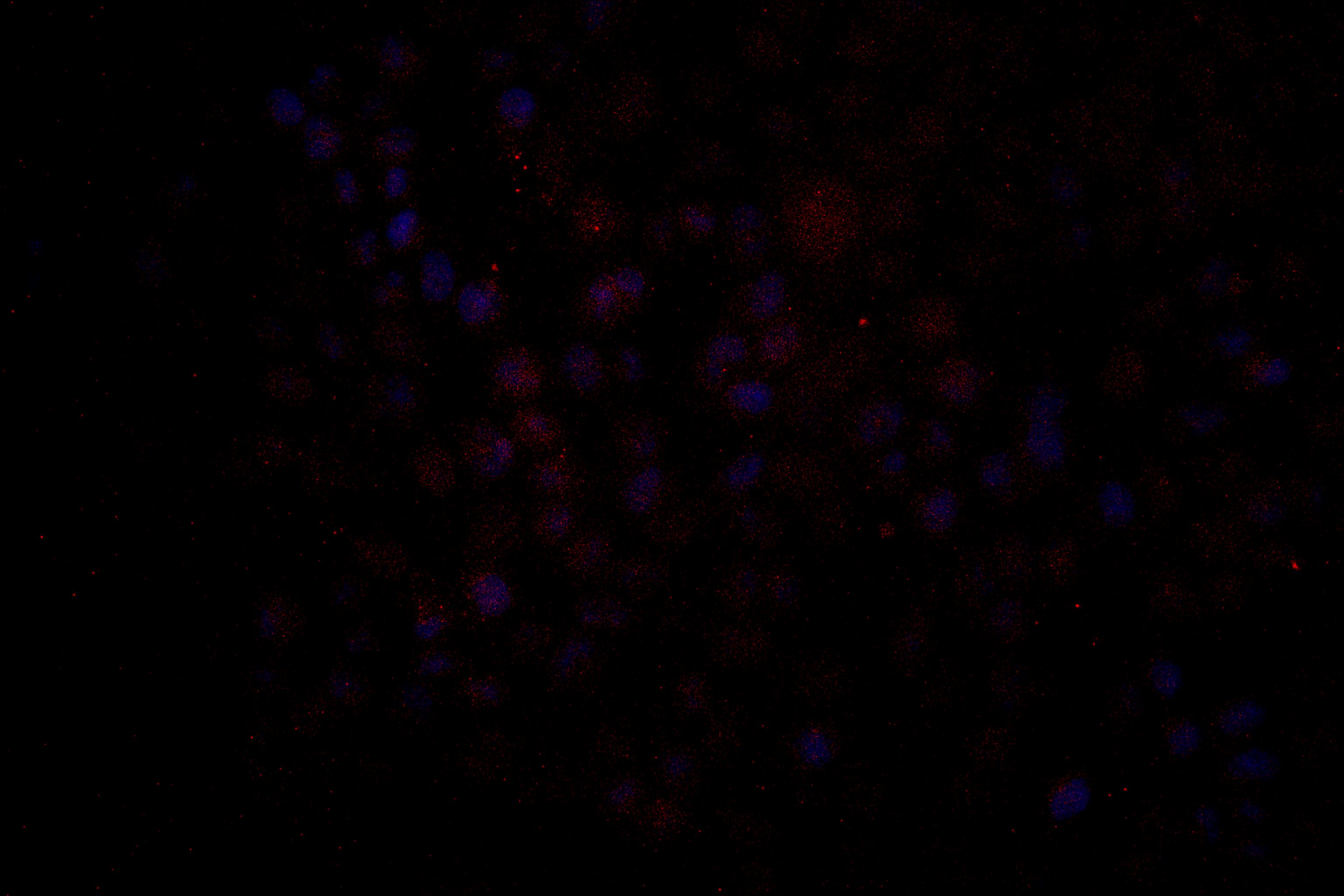

Supplement: Supplemental Information 4 [file peerj-11-14608-s004.zip › micrograph Figure1 CD1632/MO-NC/2-2-2.jpg]

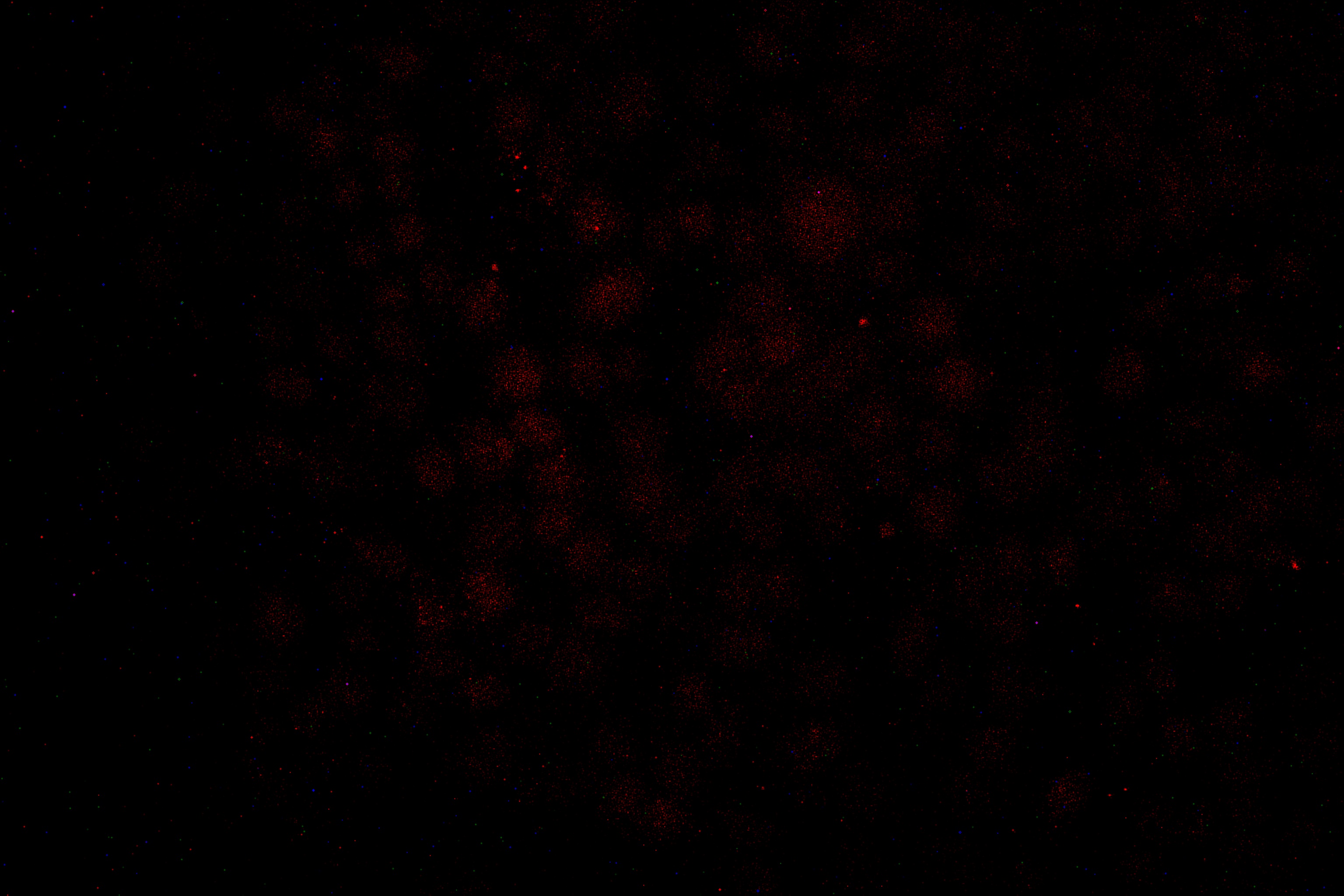

Supplement: Supplemental Information 4 [file peerj-11-14608-s004.zip › micrograph Figure1 CD1632/MO-NC/2-2.jpg]

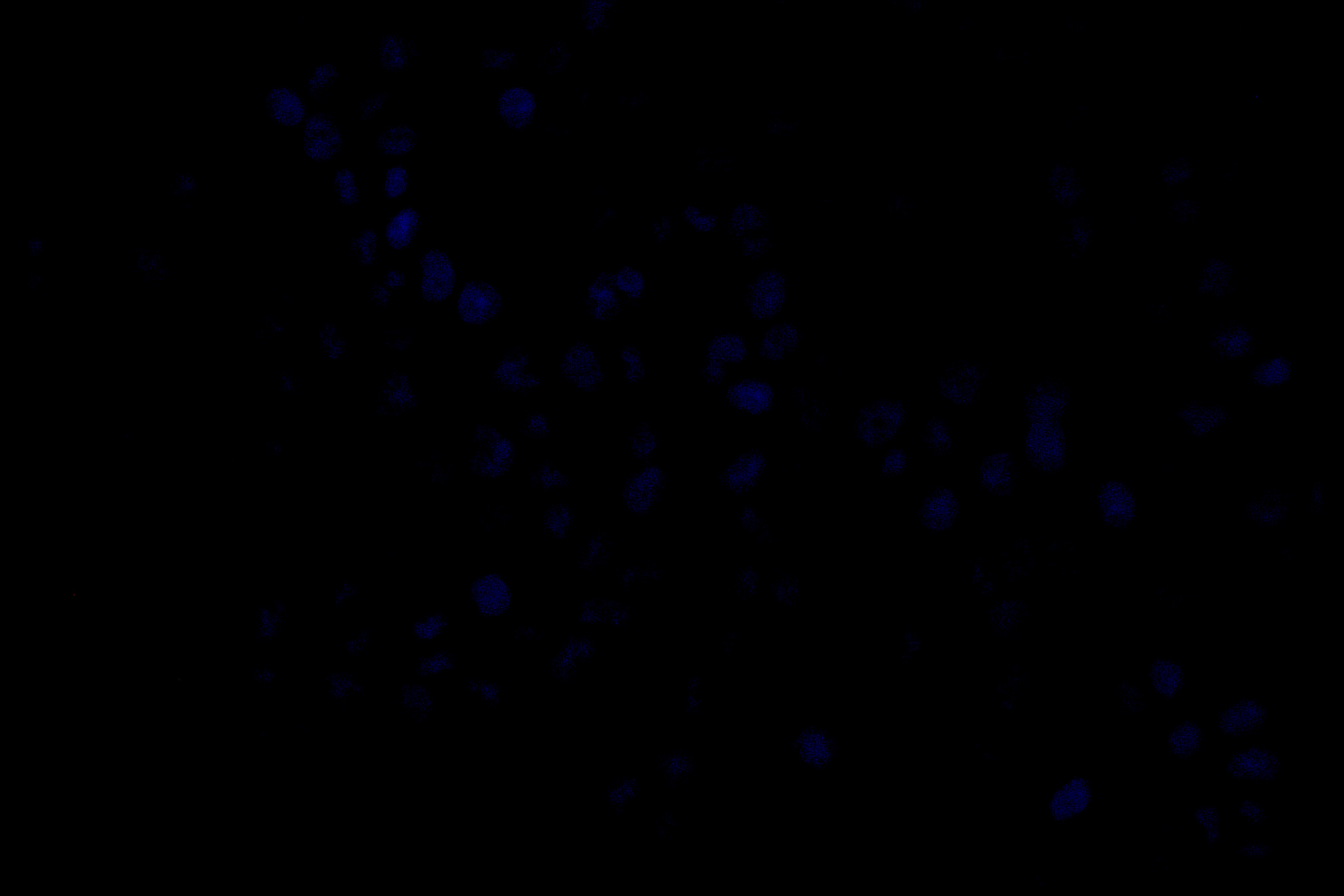

Supplement: Supplemental Information 4 [file peerj-11-14608-s004.zip › micrograph Figure1 CD1632/MO-NC/2.jpg]

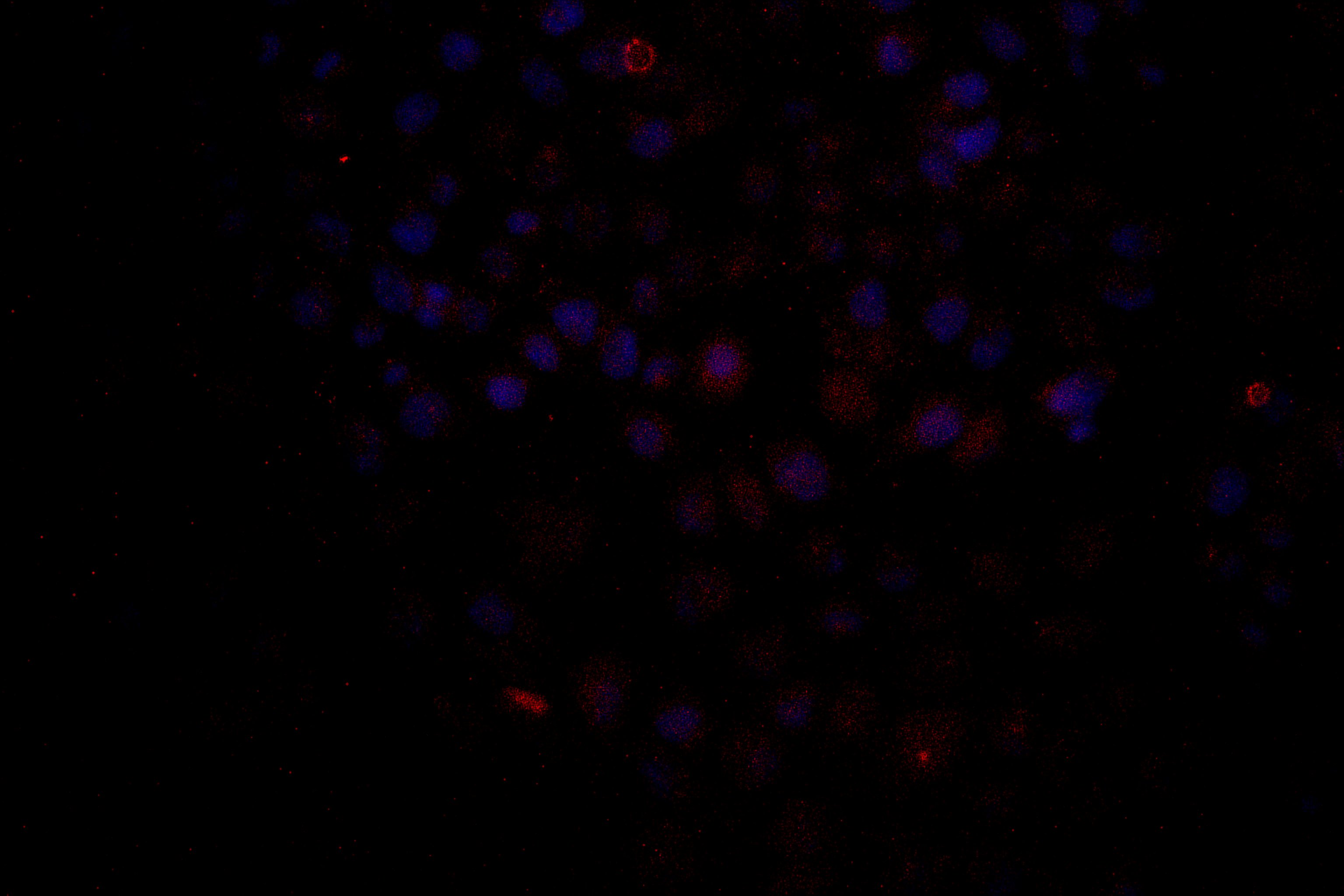

Supplement: Supplemental Information 4 [file peerj-11-14608-s004.zip › micrograph Figure1 CD1632/MO-NC/3-3-3.jpg]

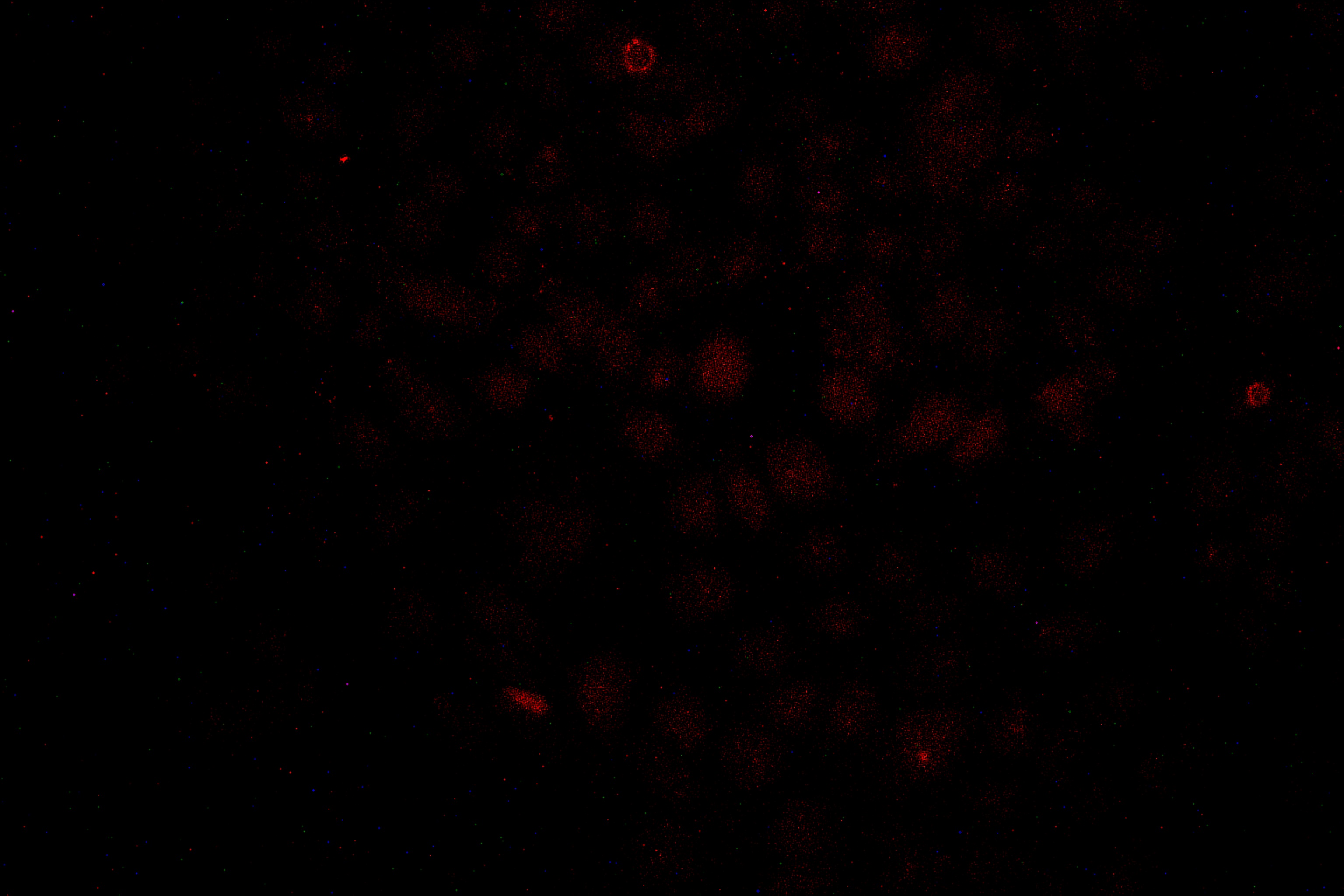

Supplement: Supplemental Information 4 [file peerj-11-14608-s004.zip › micrograph Figure1 CD1632/MO-NC/3-3.jpg]

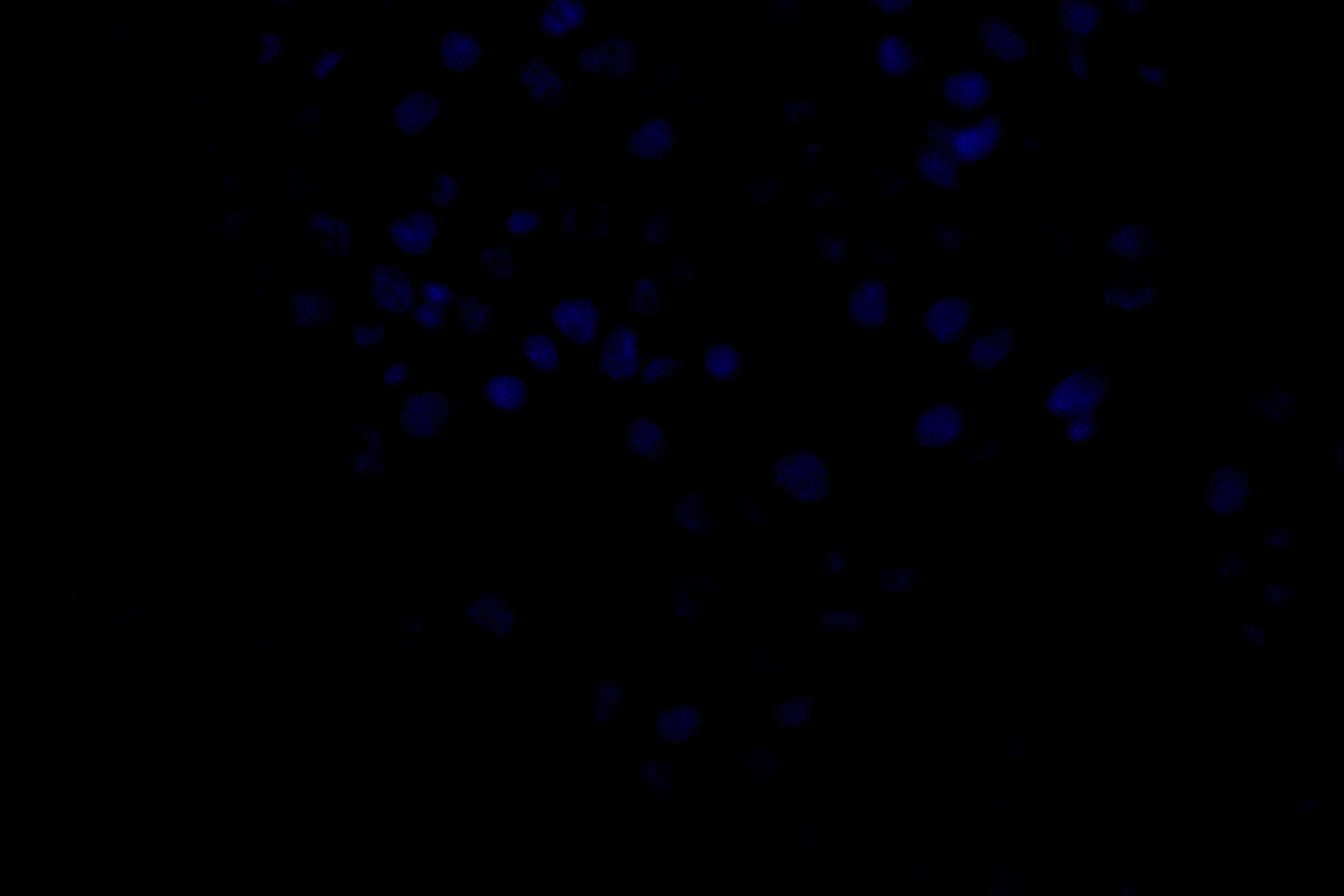

Supplement: Supplemental Information 4 [file peerj-11-14608-s004.zip › micrograph Figure1 CD1632/MO-NC/3.jpg]

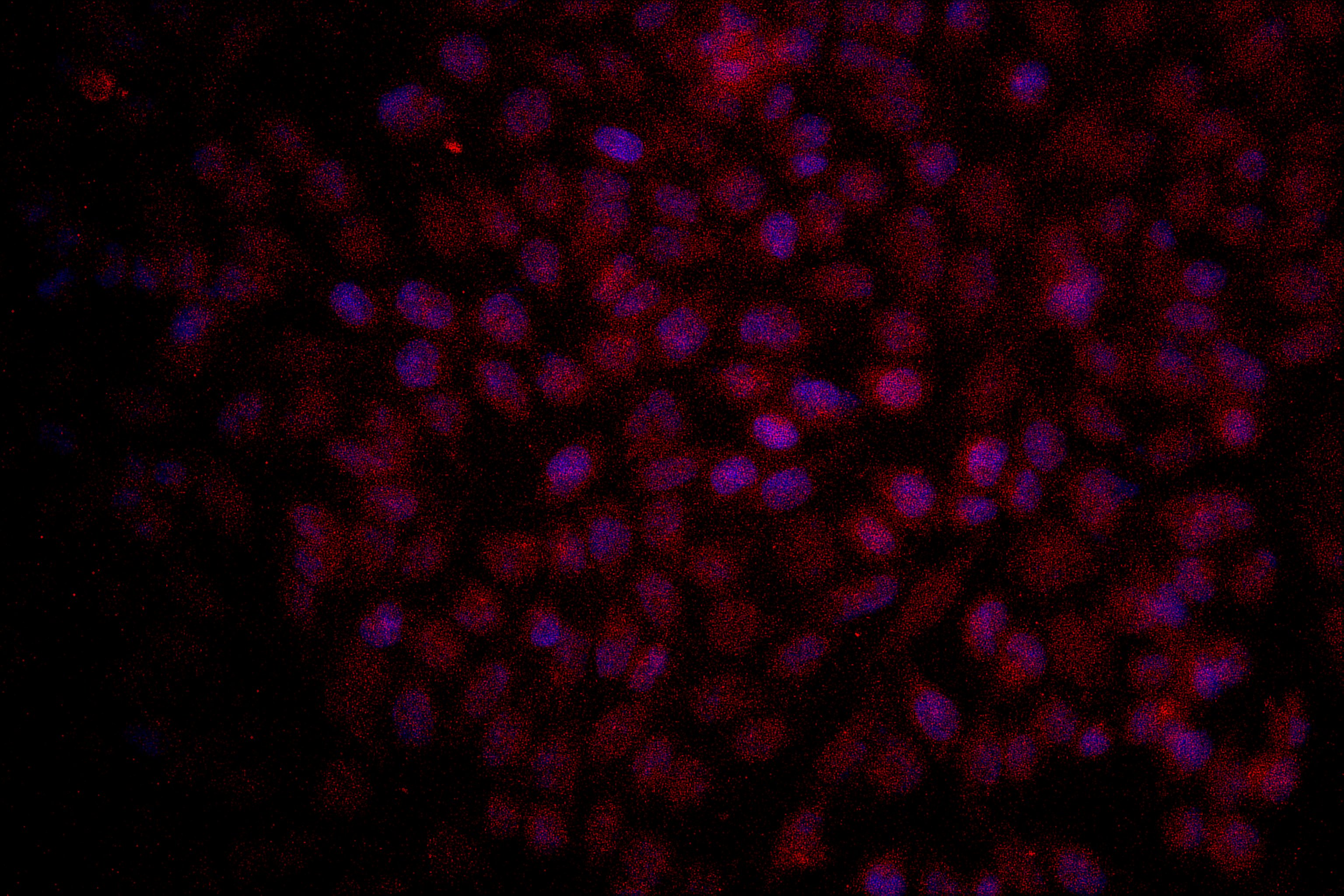

Supplement: Supplemental Information 4 [file peerj-11-14608-s004.zip › micrograph Figure1 CD1632/MO-vitexin/1-1-1.jpg]

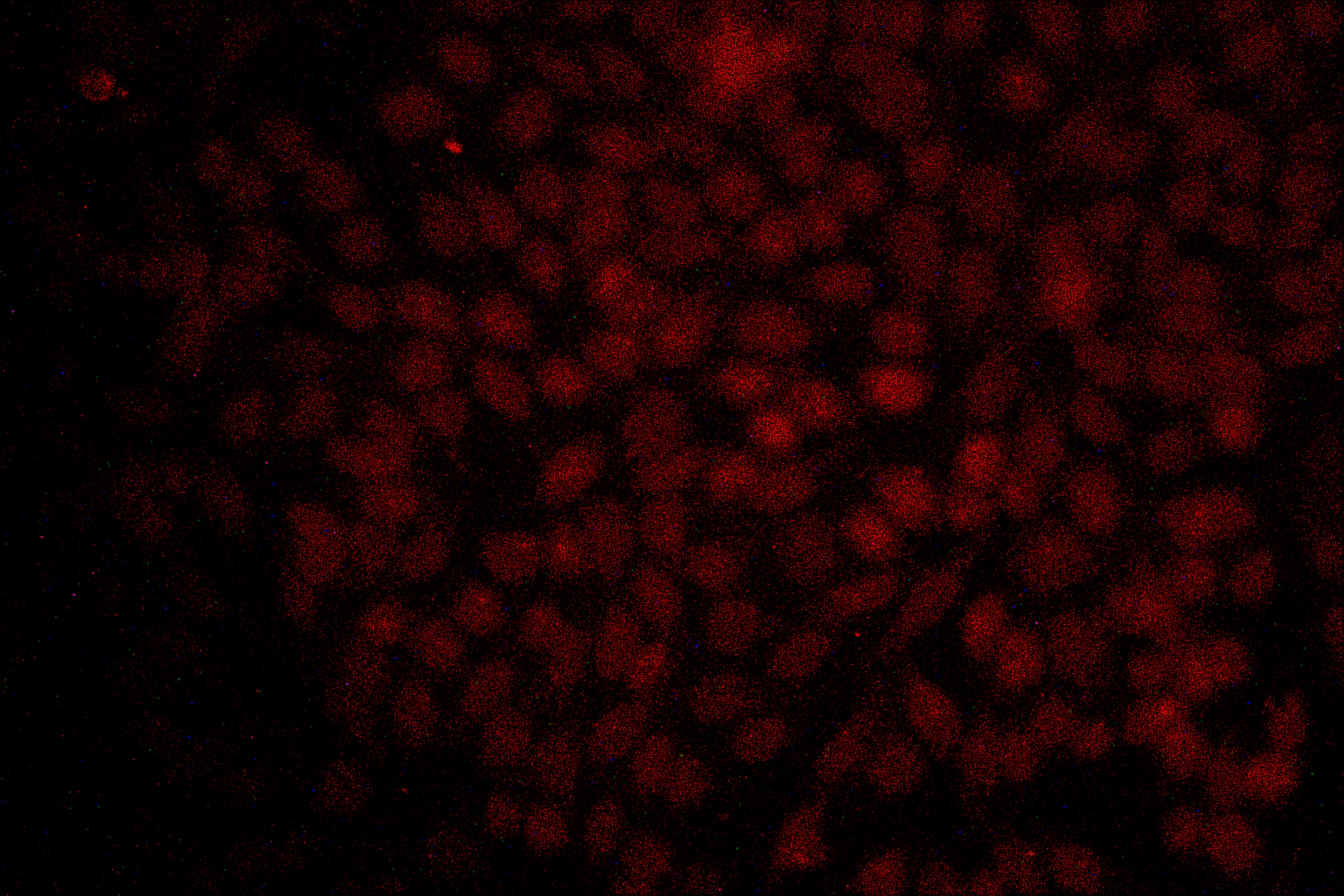

Supplement: Supplemental Information 4 [file peerj-11-14608-s004.zip › micrograph Figure1 CD1632/MO-vitexin/1-1.jpg]

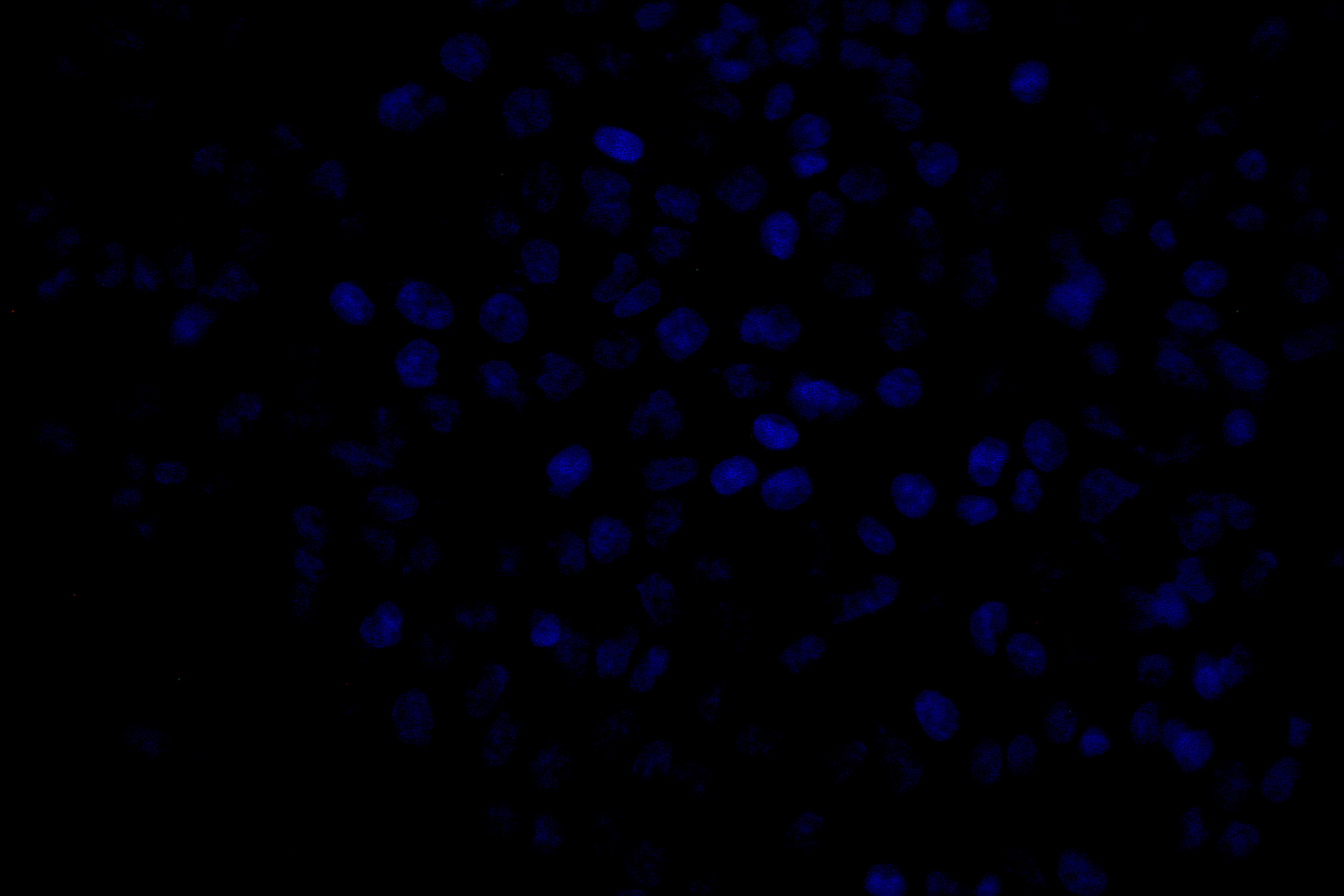

Supplement: Supplemental Information 4 [file peerj-11-14608-s004.zip › micrograph Figure1 CD1632/MO-vitexin/1.jpg]

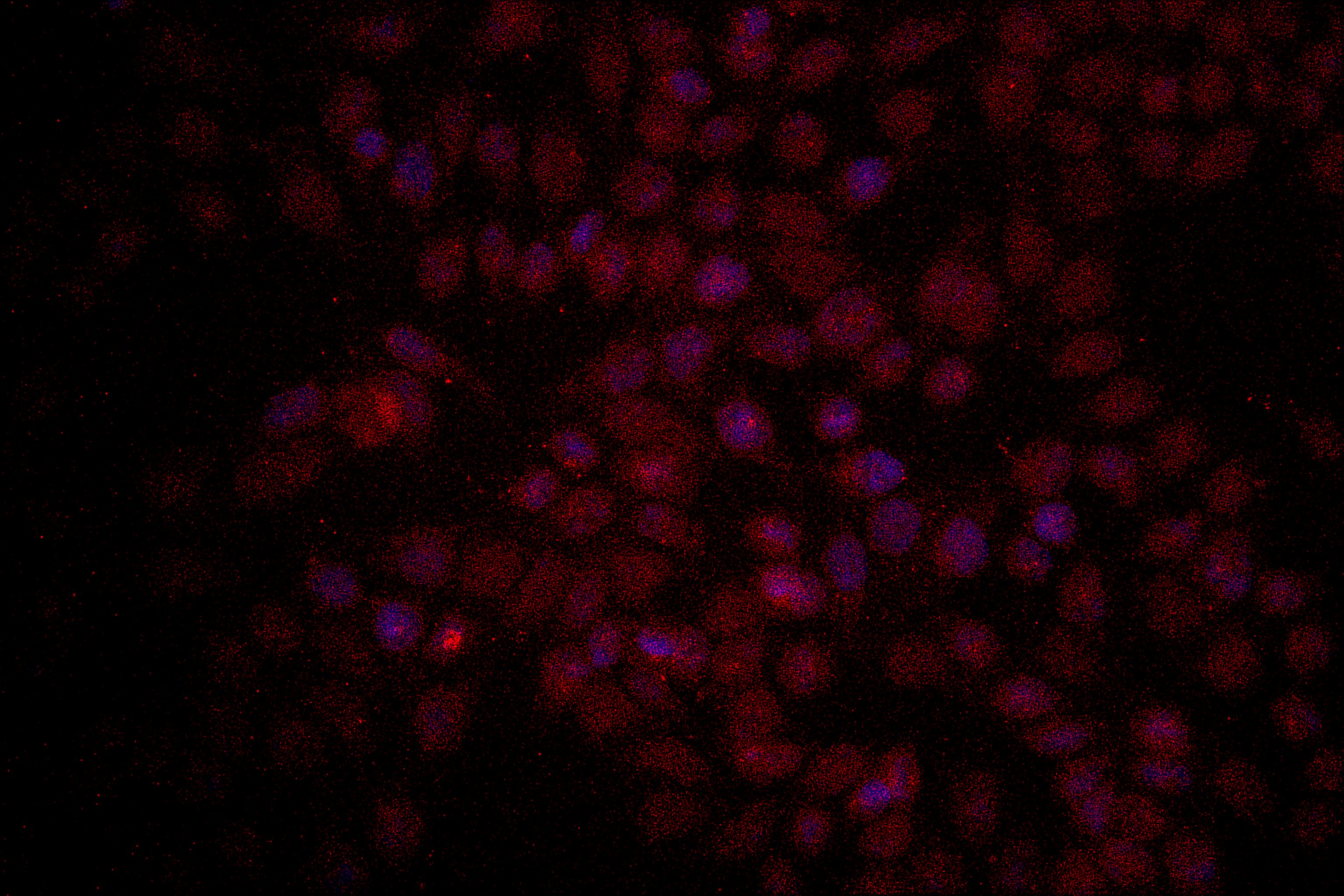

Supplement: Supplemental Information 4 [file peerj-11-14608-s004.zip › micrograph Figure1 CD1632/MO-vitexin/2-2-2.jpg]

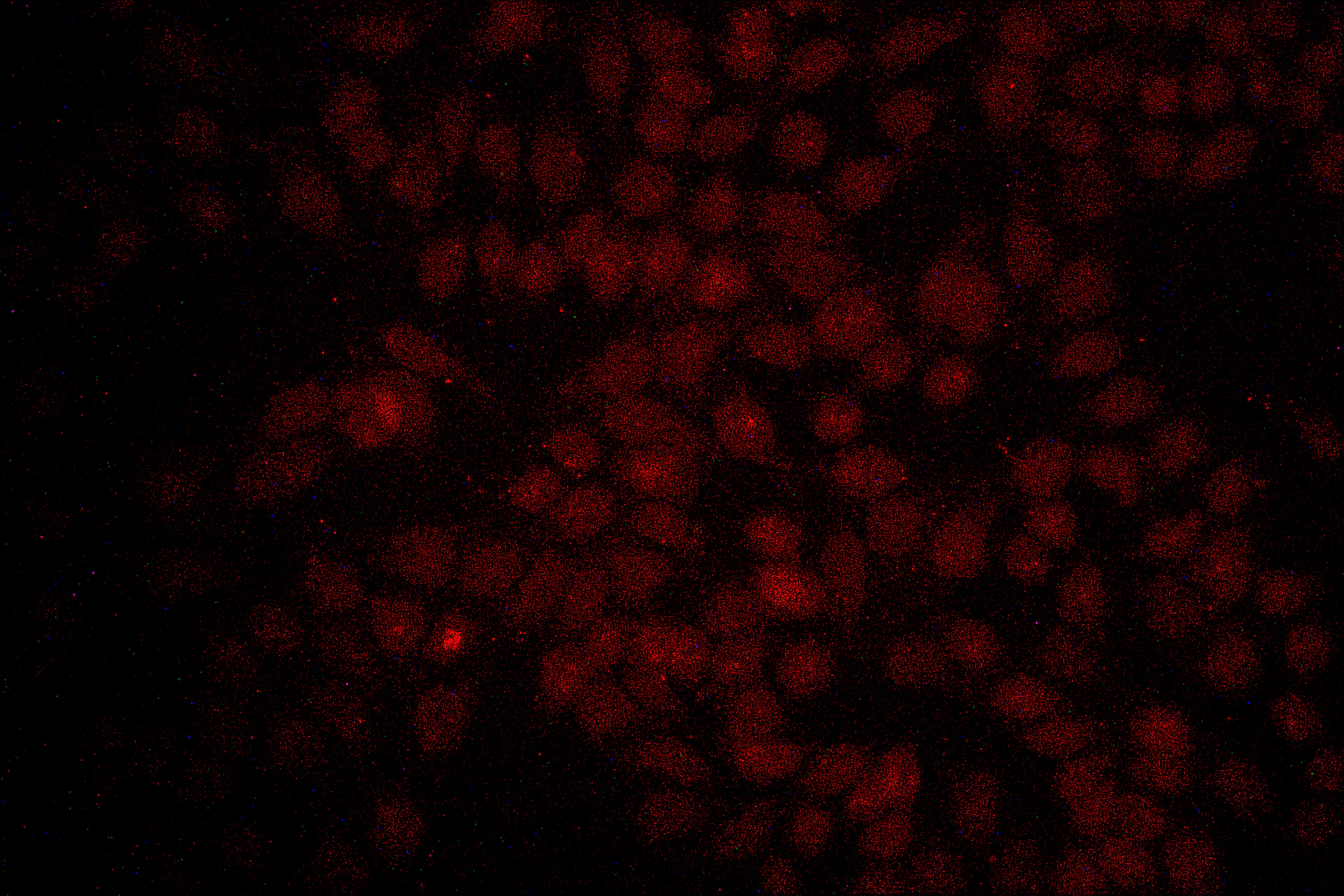

Supplement: Supplemental Information 4 [file peerj-11-14608-s004.zip › micrograph Figure1 CD1632/MO-vitexin/2-2.jpg]

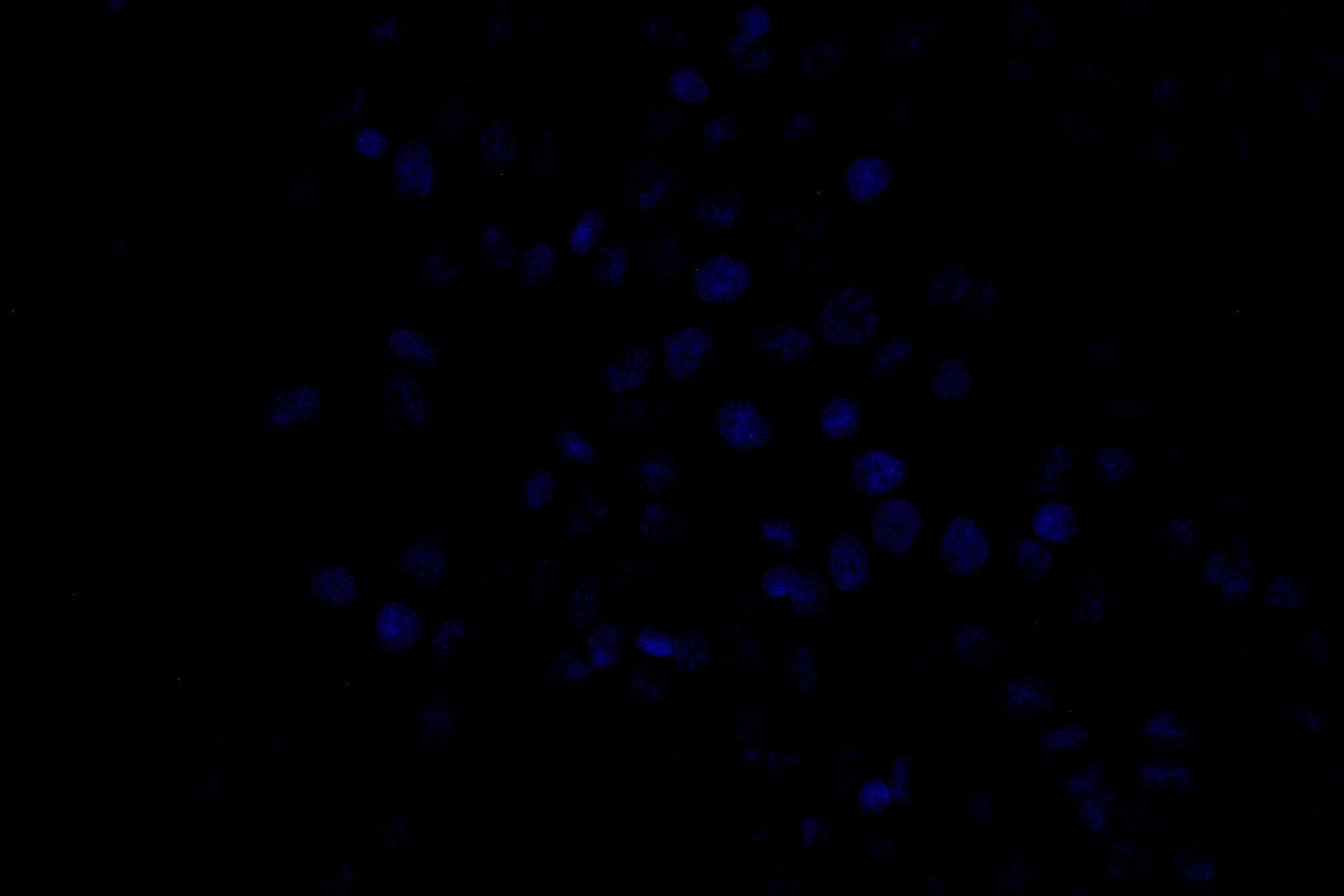

Supplement: Supplemental Information 4 [file peerj-11-14608-s004.zip › micrograph Figure1 CD1632/MO-vitexin/2.jpg]

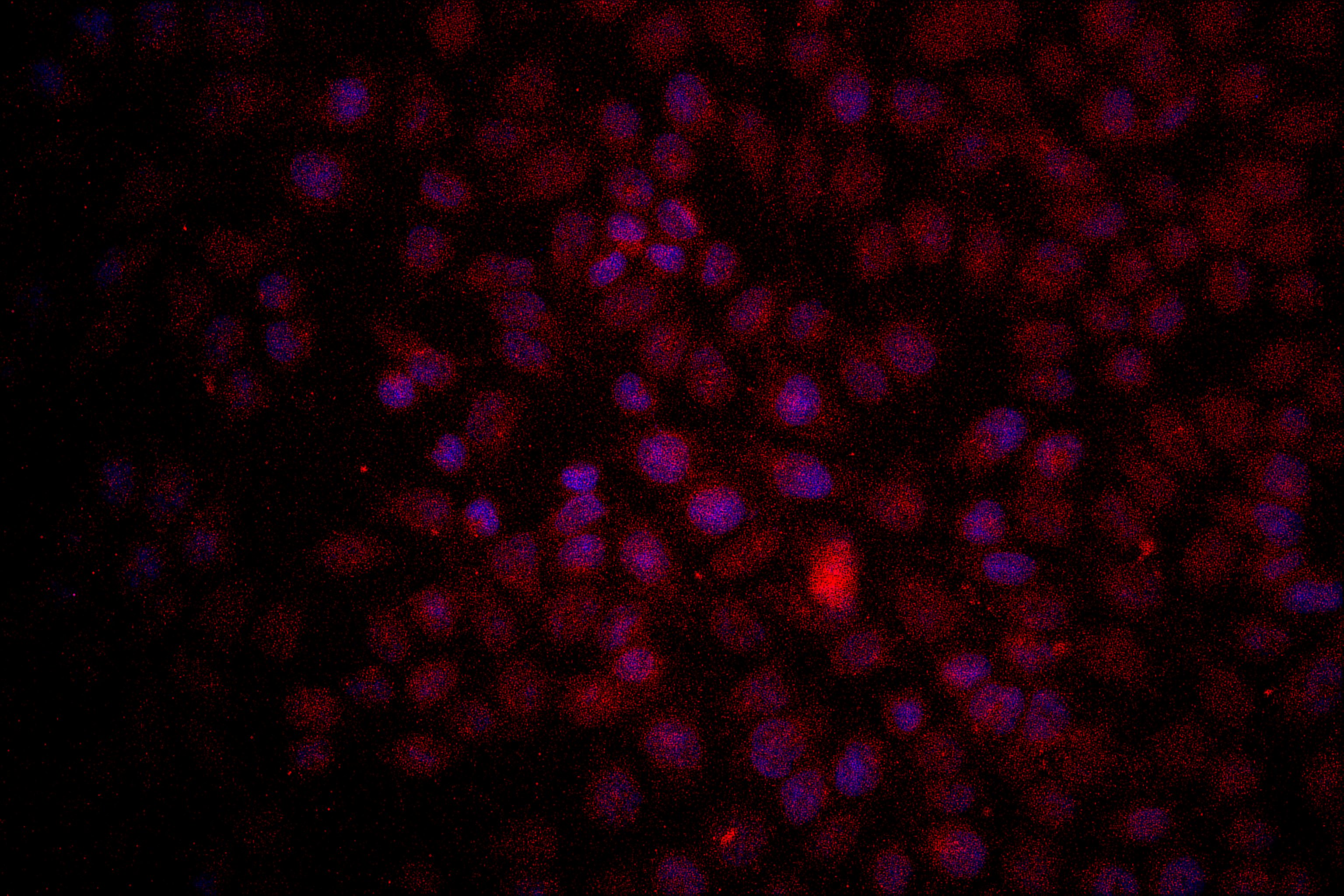

Supplement: Supplemental Information 4 [file peerj-11-14608-s004.zip › micrograph Figure1 CD1632/MO-vitexin/3-3-3.jpg]

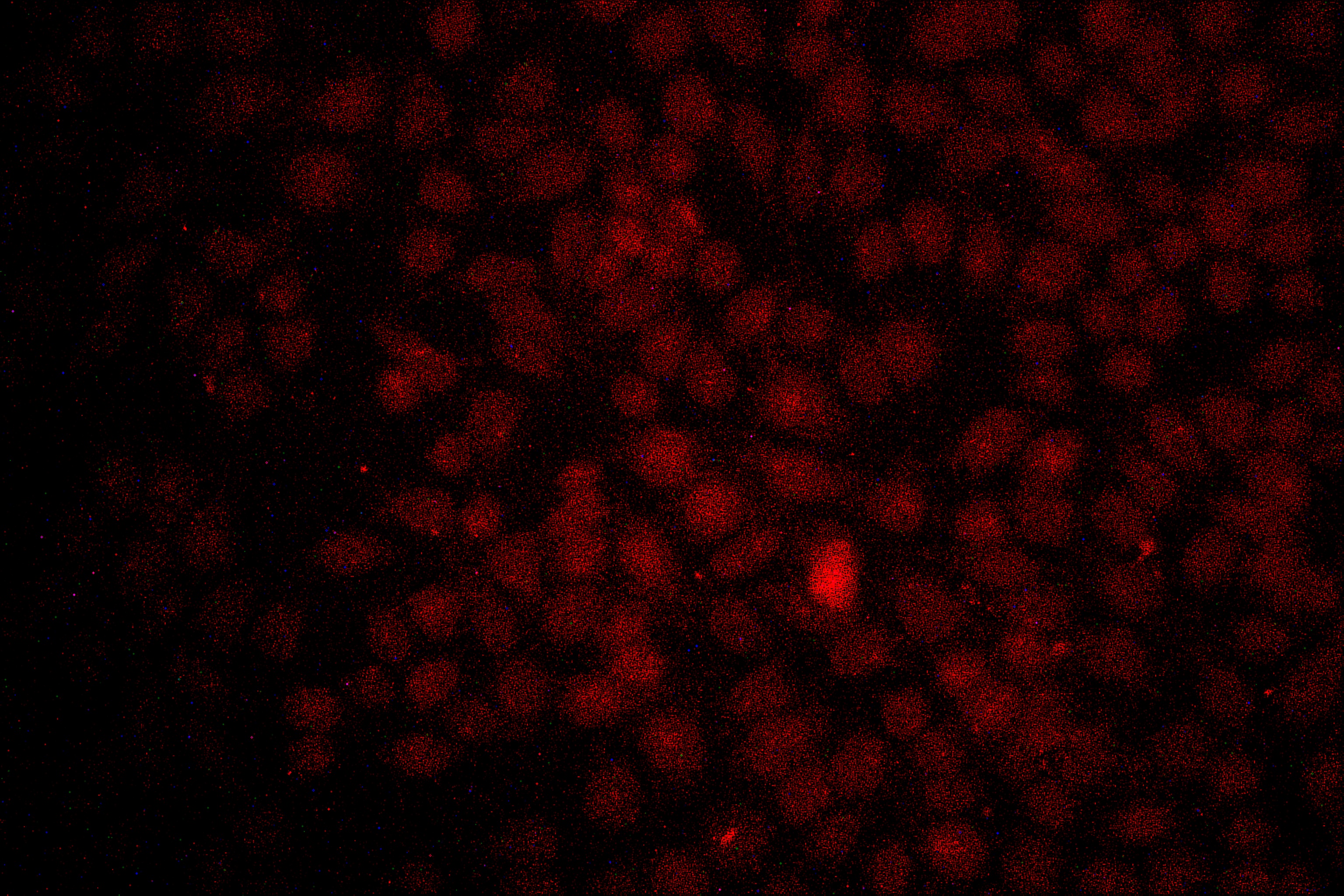

Supplement: Supplemental Information 4 [file peerj-11-14608-s004.zip › micrograph Figure1 CD1632/MO-vitexin/3-3.jpg]

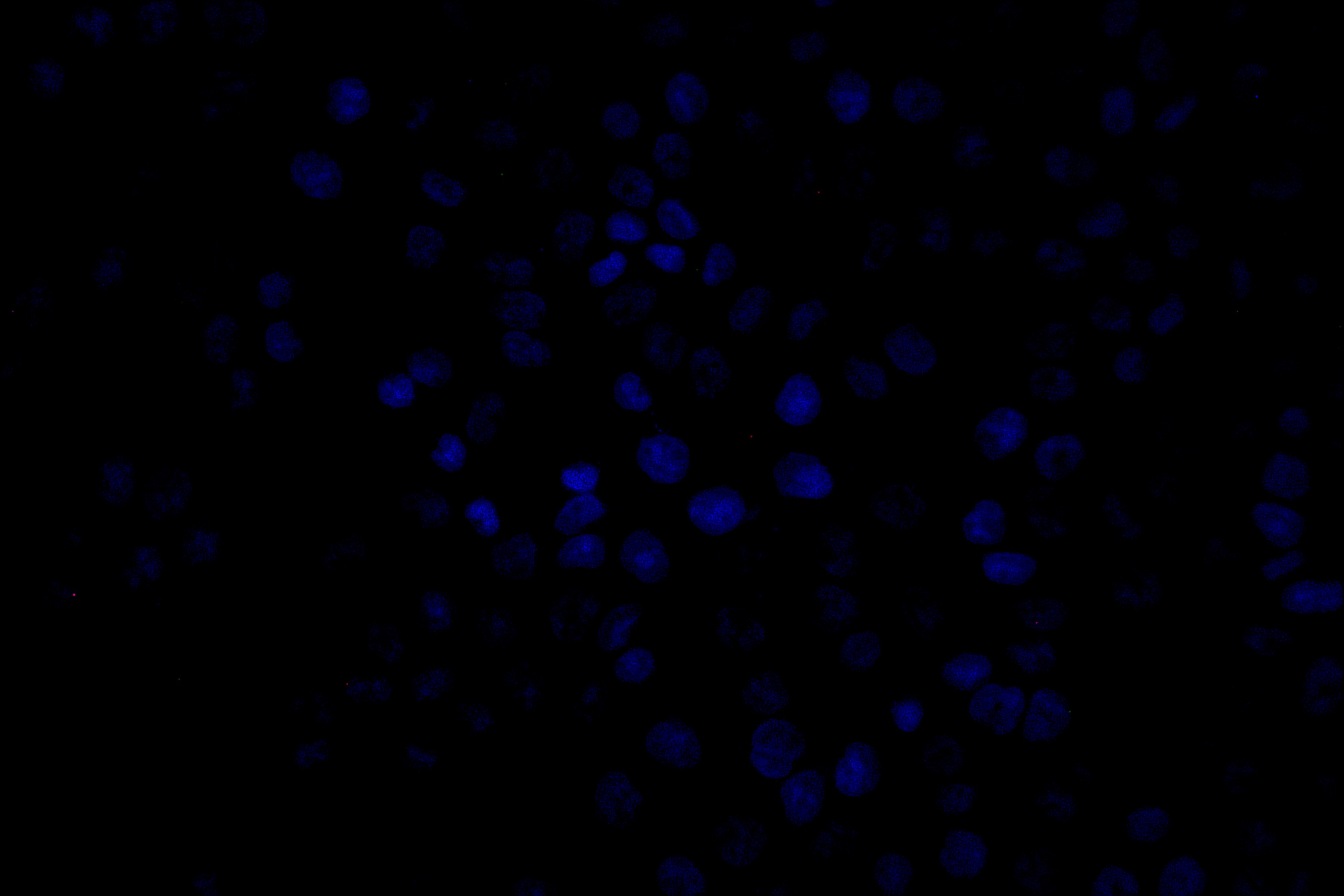

Supplement: Supplemental Information 4 [file peerj-11-14608-s004.zip › micrograph Figure1 CD1632/MO-vitexin/3.jpg]

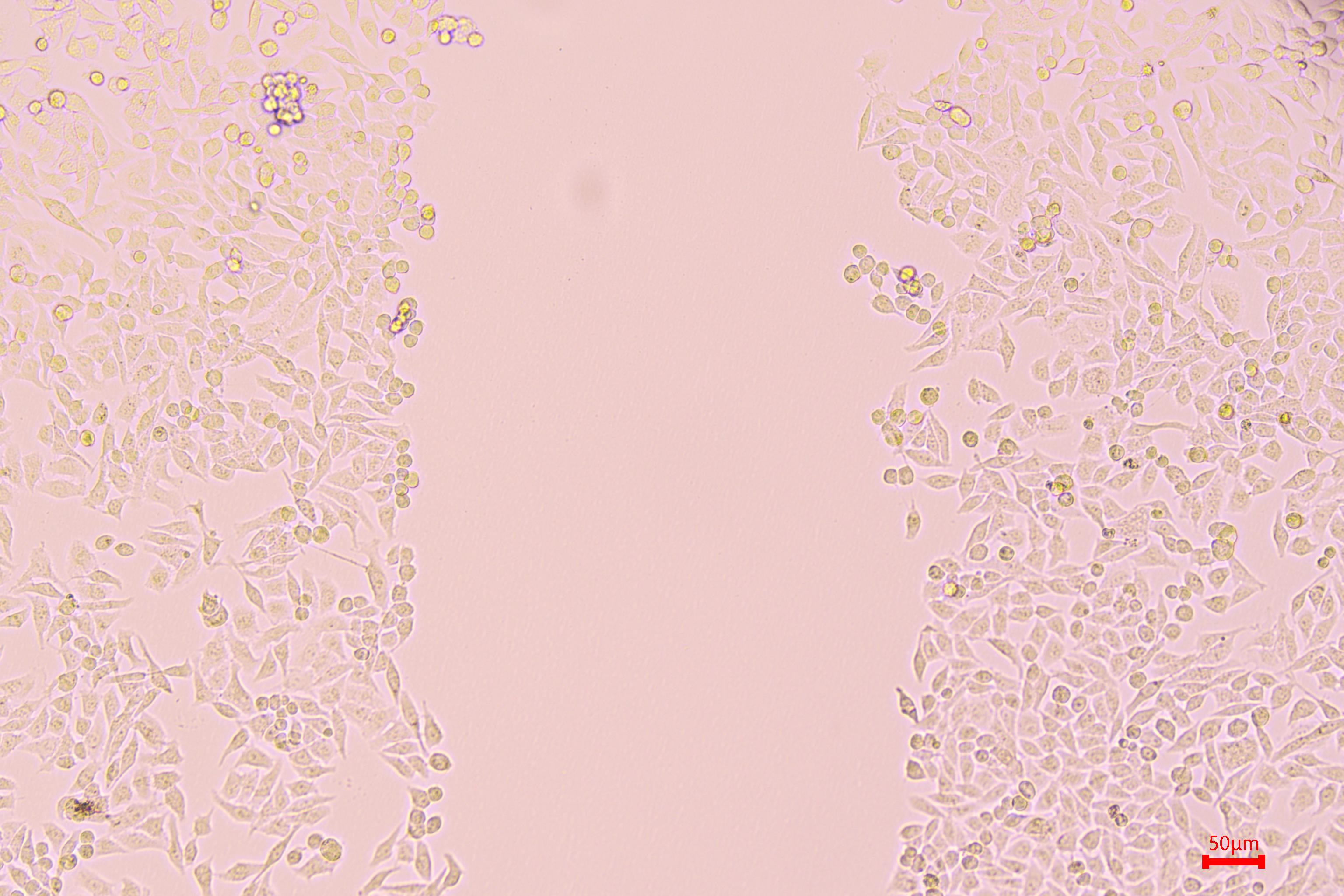

Supplement: Supplemental Information 5 [file peerj-11-14608-s005.zip › micrograph Figure2/B/A549/0h (1).jpg]

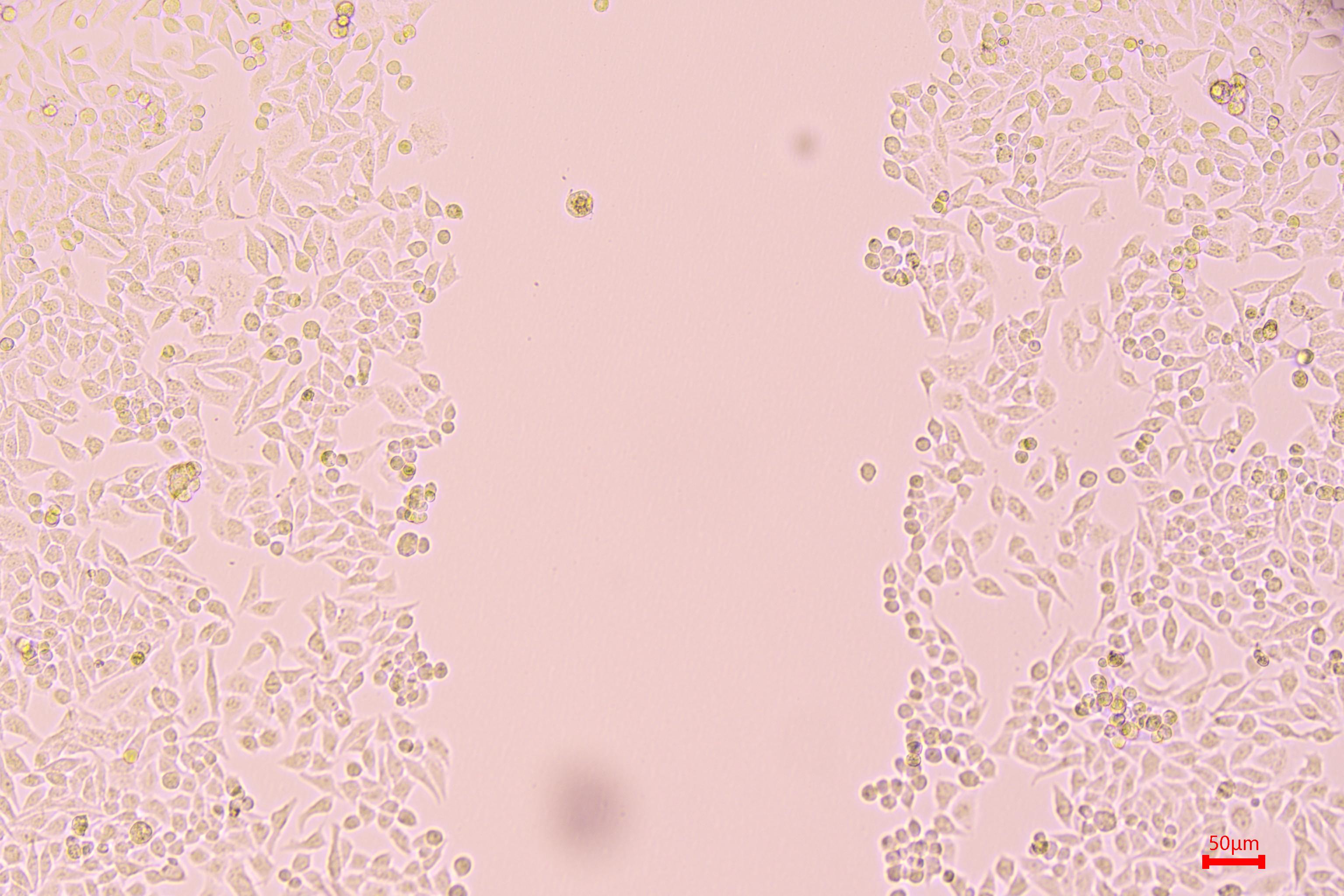

Supplement: Supplemental Information 5 [file peerj-11-14608-s005.zip › micrograph Figure2/B/A549/0h (2).jpg]

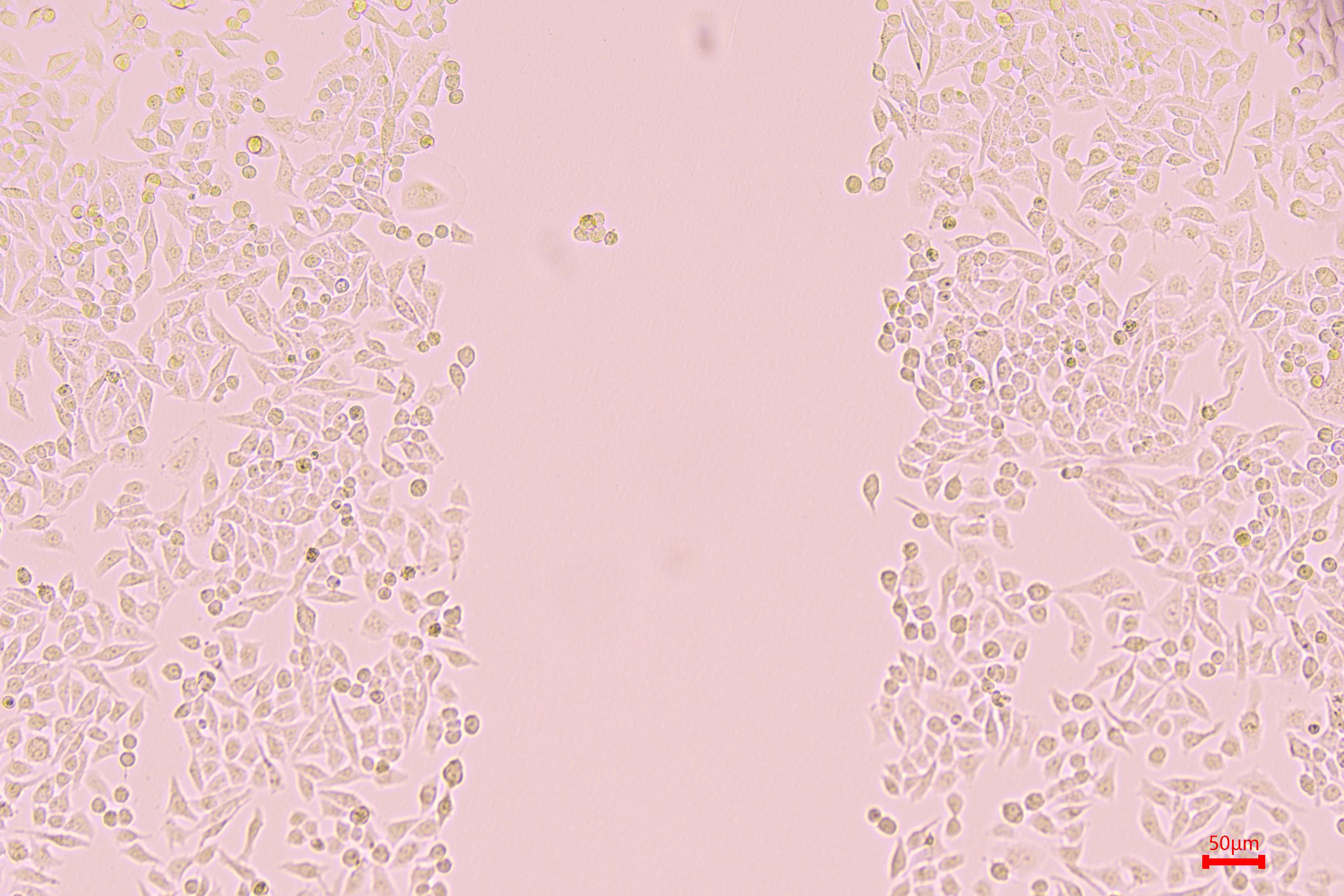

Supplement: Supplemental Information 5 [file peerj-11-14608-s005.zip › micrograph Figure2/B/A549/0h (3).jpg]

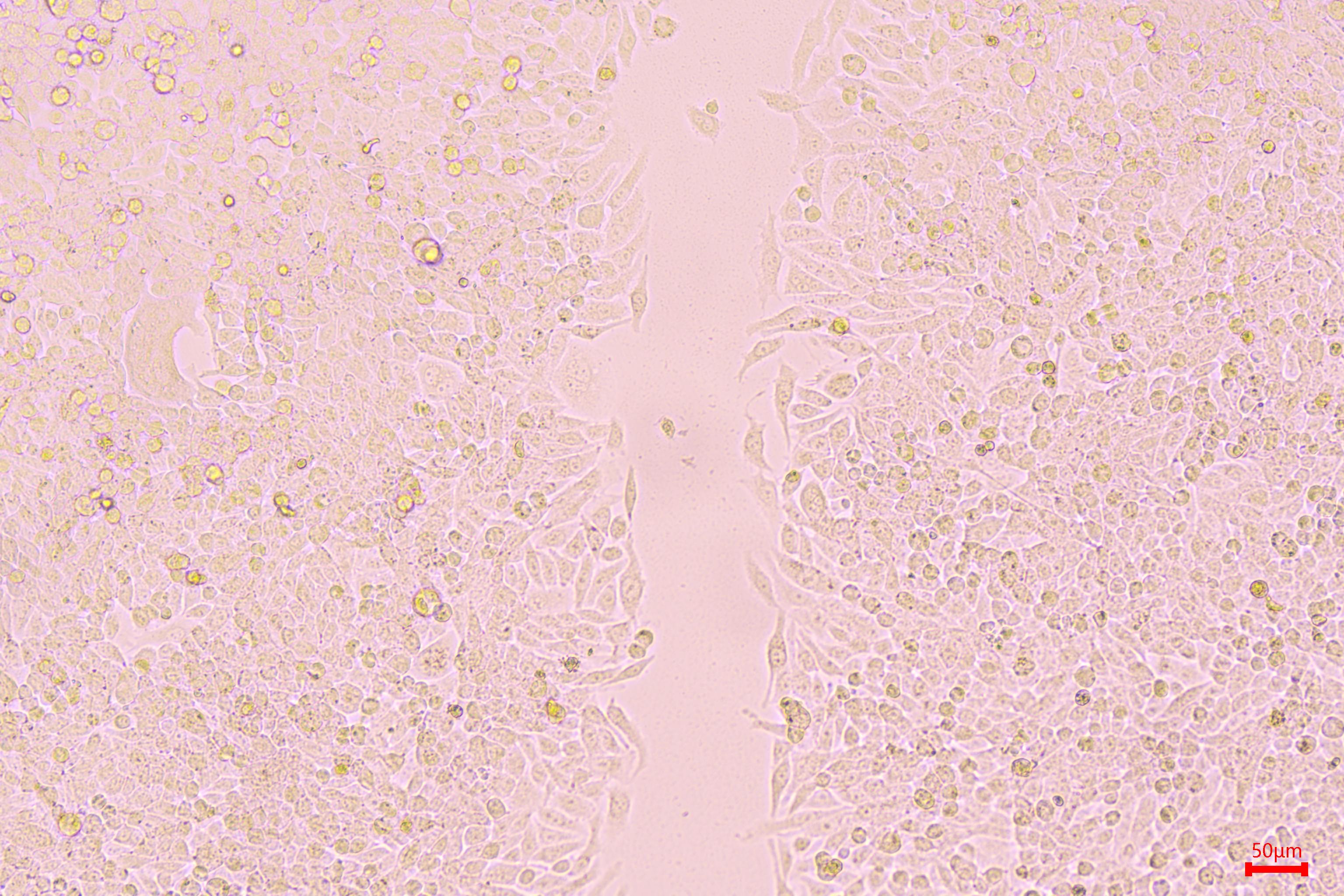

Supplement: Supplemental Information 5 [file peerj-11-14608-s005.zip › micrograph Figure2/B/A549/24h (1).jpg]

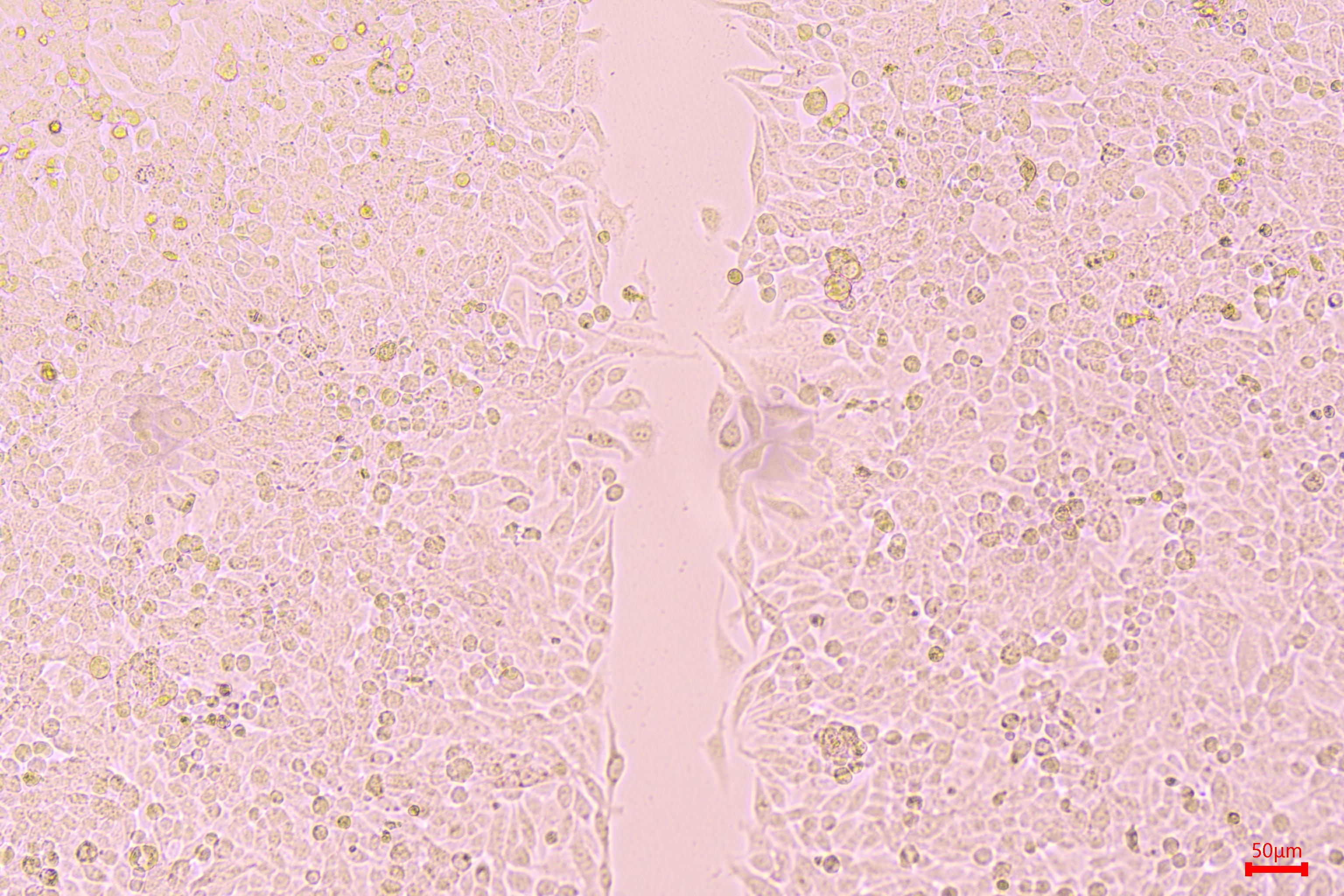

Supplement: Supplemental Information 5 [file peerj-11-14608-s005.zip › micrograph Figure2/B/A549/24h (2).jpg]

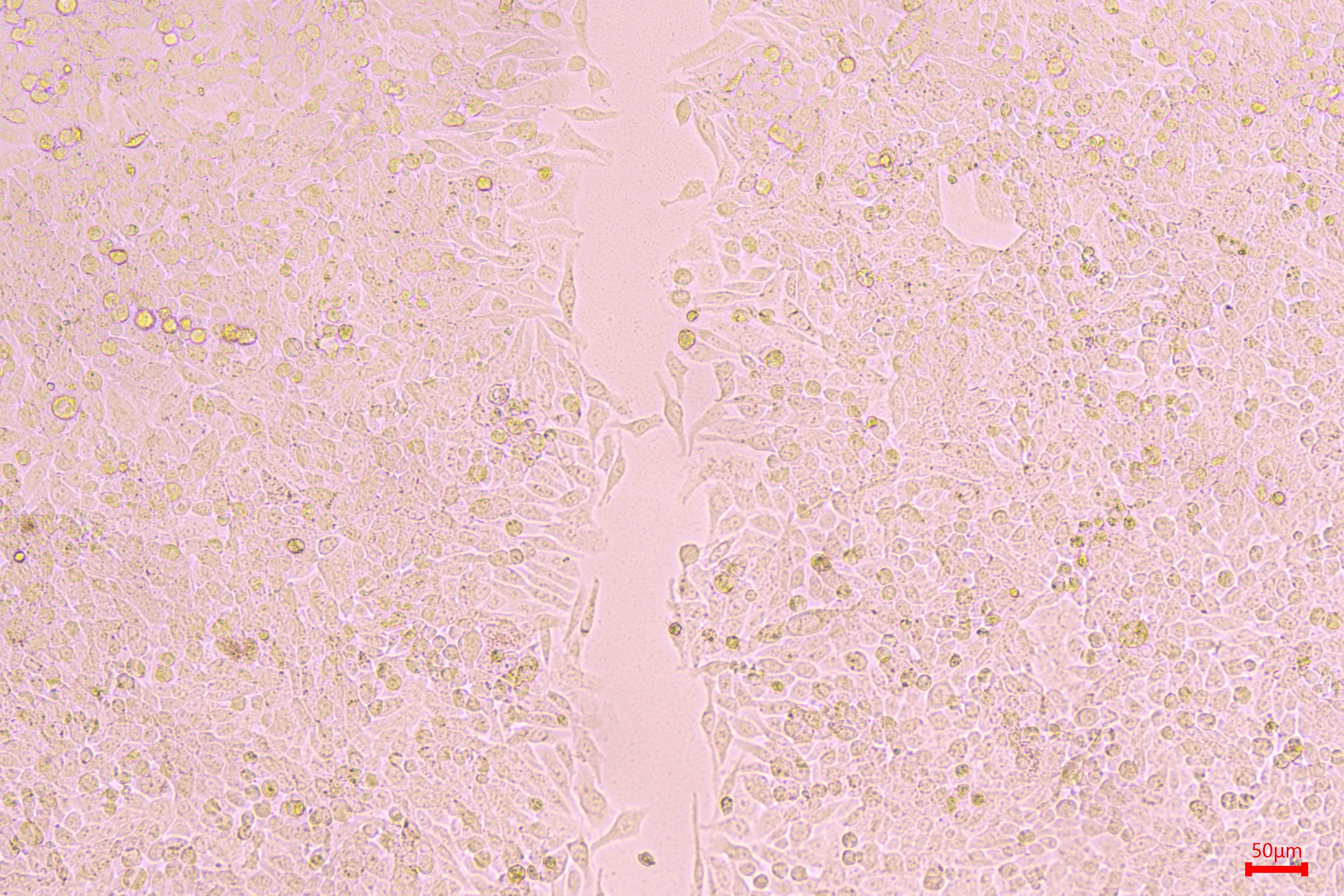

Supplement: Supplemental Information 5 [file peerj-11-14608-s005.zip › micrograph Figure2/B/A549/24h (3).jpg]

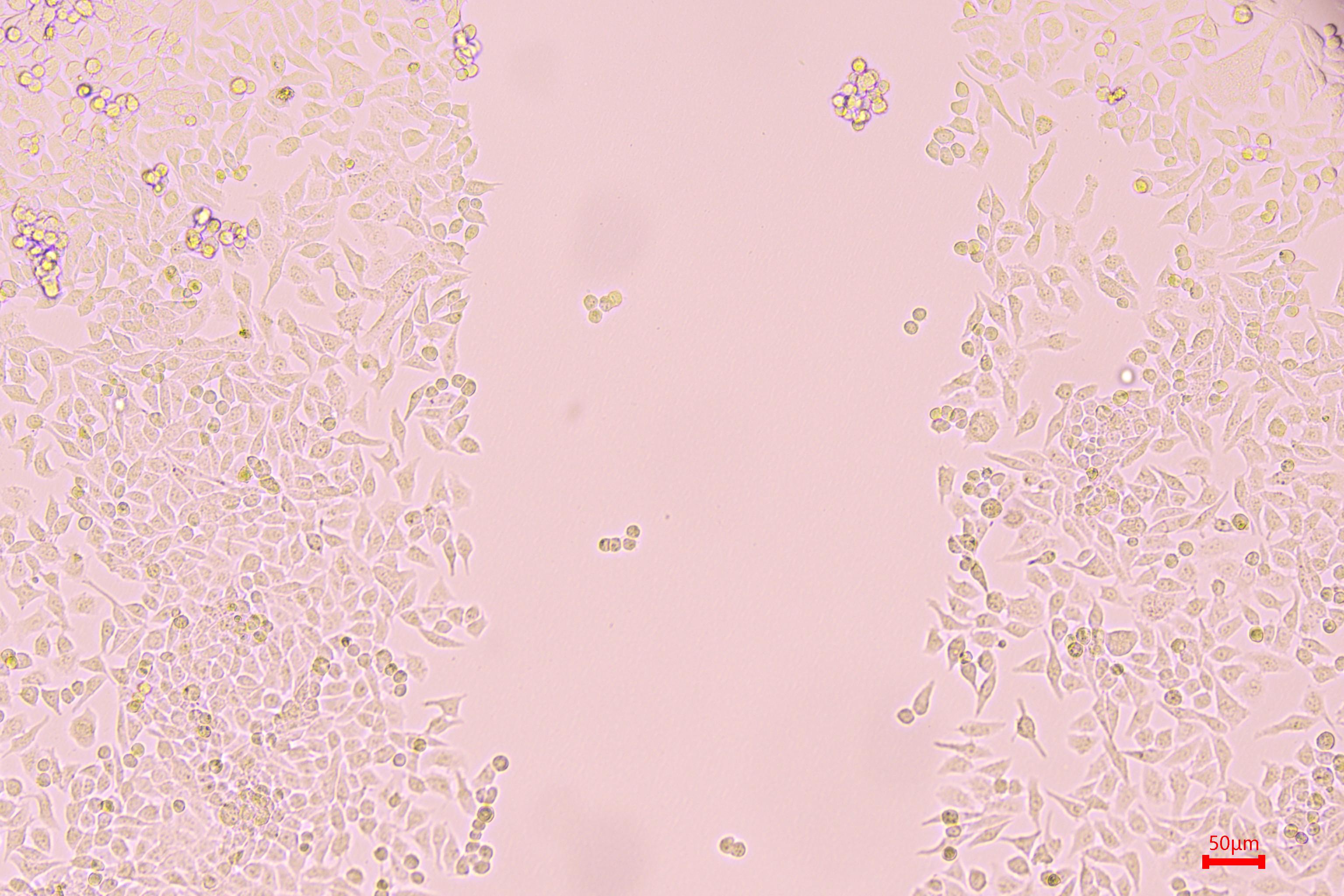

Supplement: Supplemental Information 5 [file peerj-11-14608-s005.zip › micrograph Figure2/B/A549+M1/0h (1).jpg]

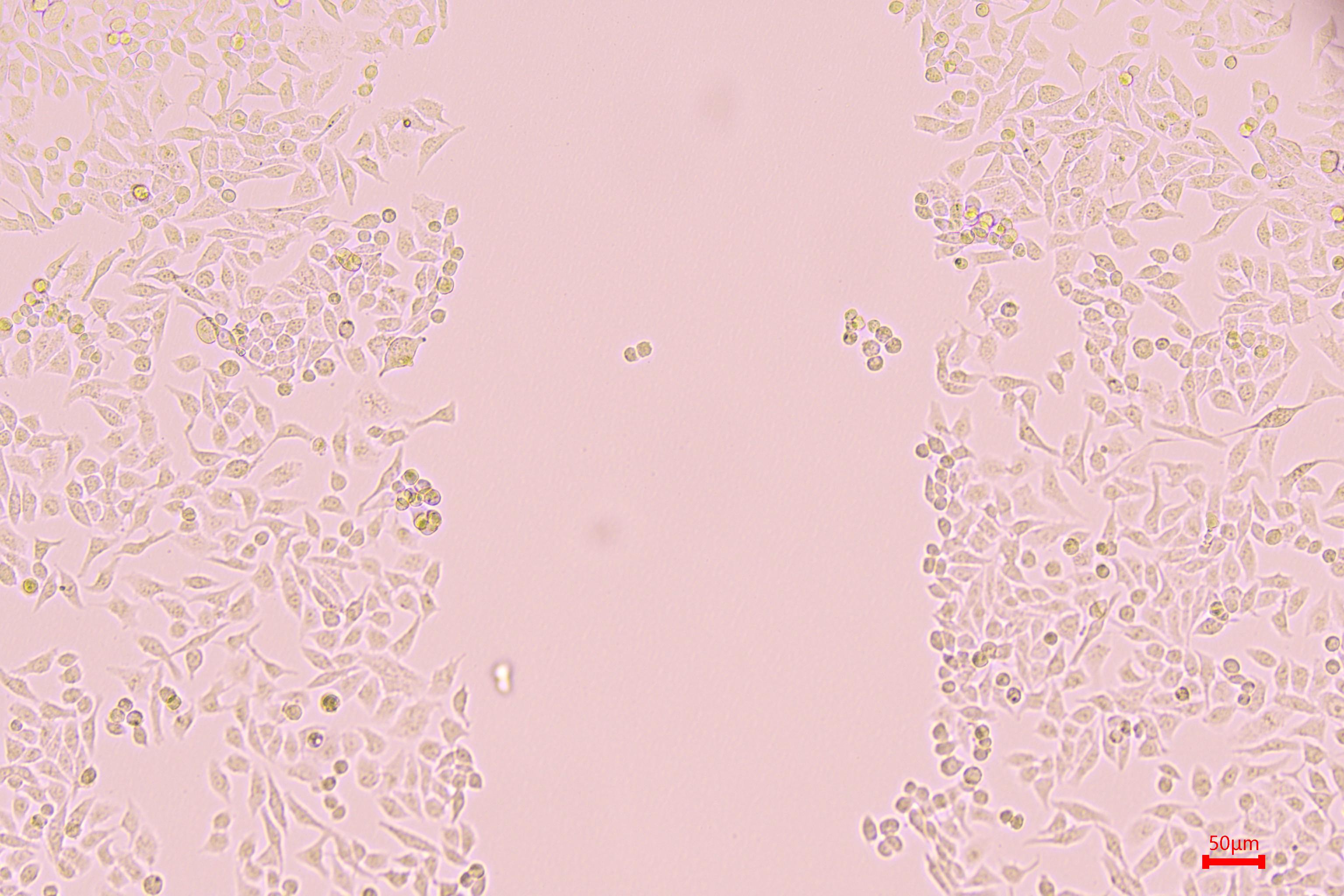

Supplement: Supplemental Information 5 [file peerj-11-14608-s005.zip › micrograph Figure2/B/A549+M1/0h (2).jpg]

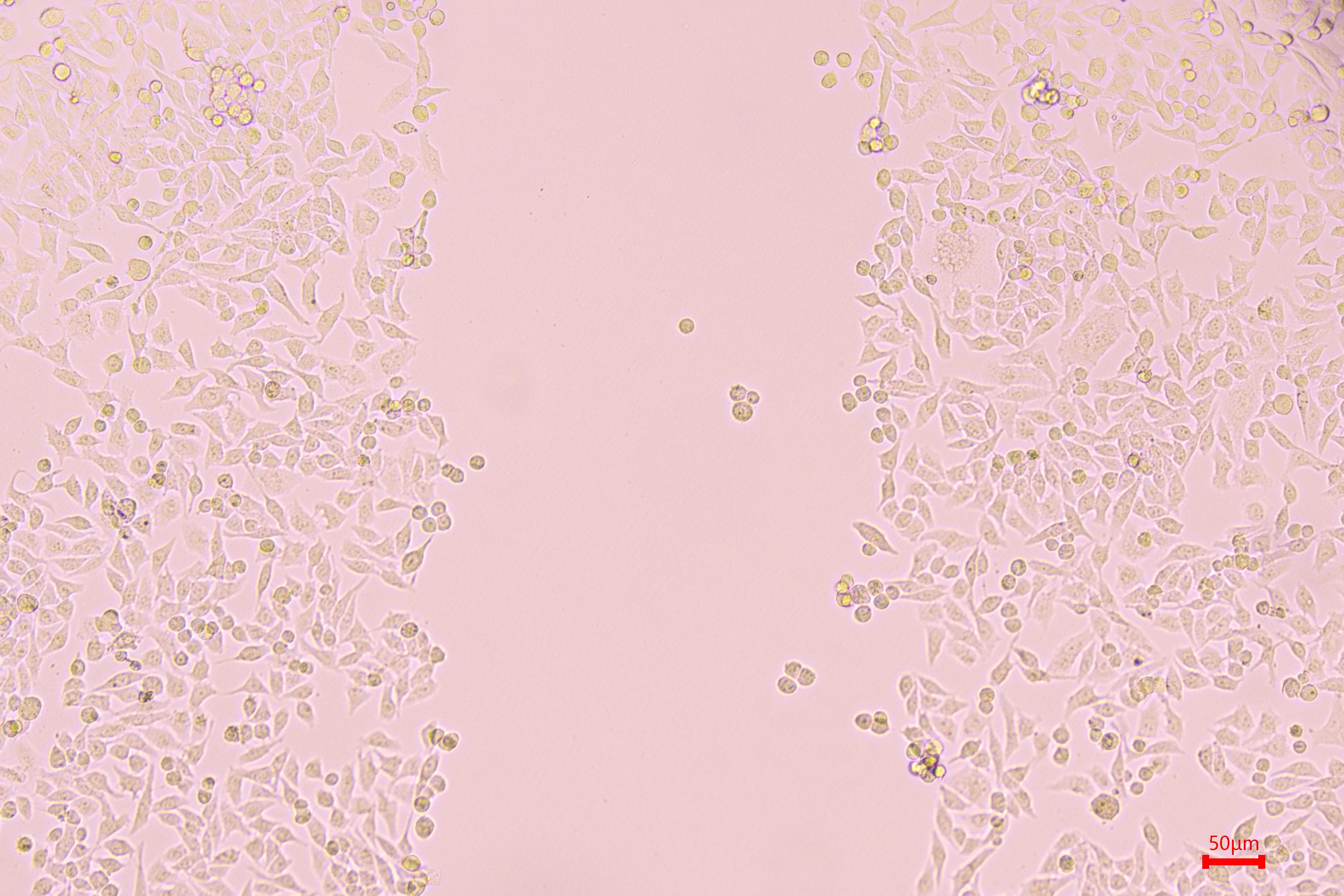

Supplement: Supplemental Information 5 [file peerj-11-14608-s005.zip › micrograph Figure2/B/A549+M1/0h (3).jpg]

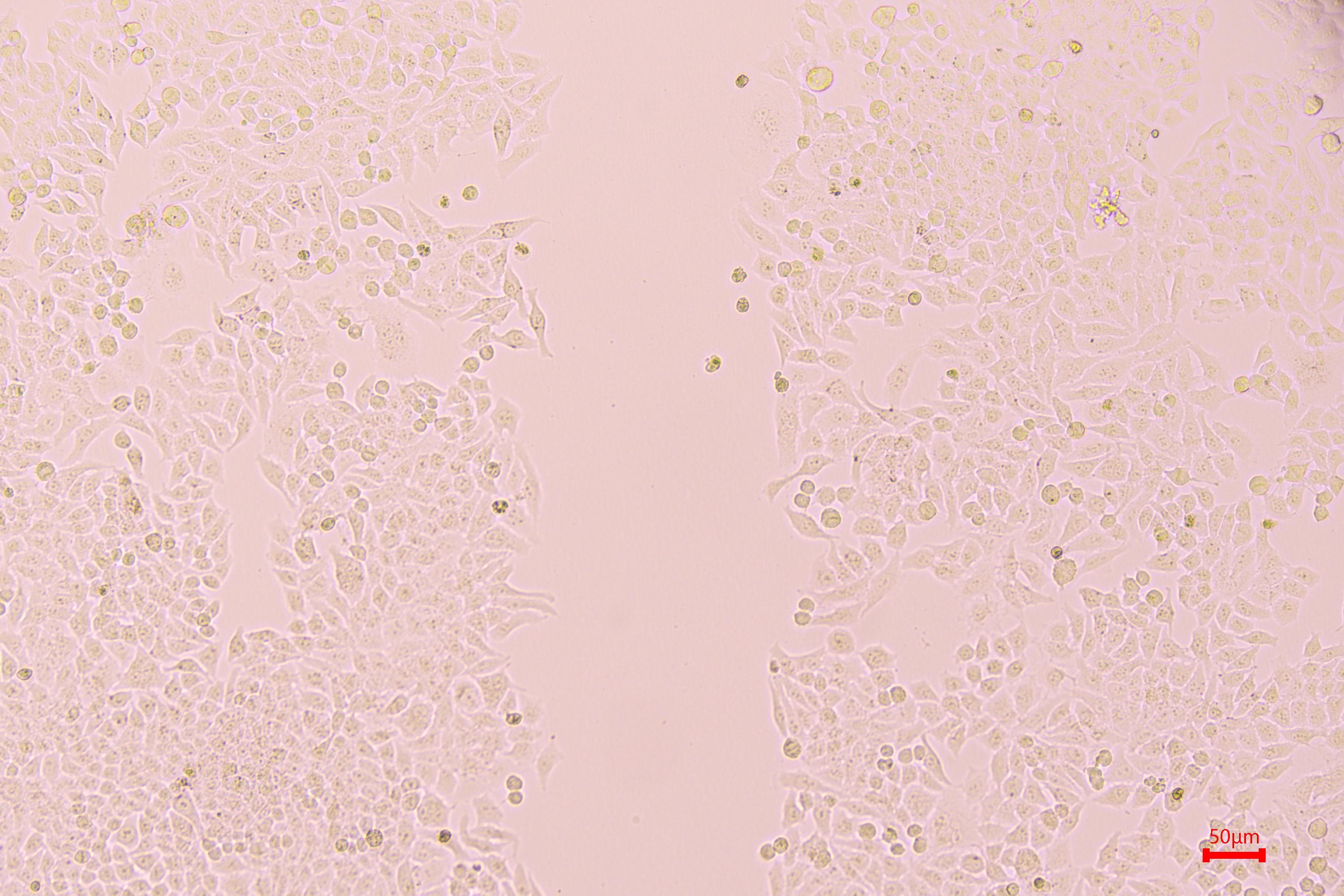

Supplement: Supplemental Information 5 [file peerj-11-14608-s005.zip › micrograph Figure2/B/A549+M1/24h (1).jpg]

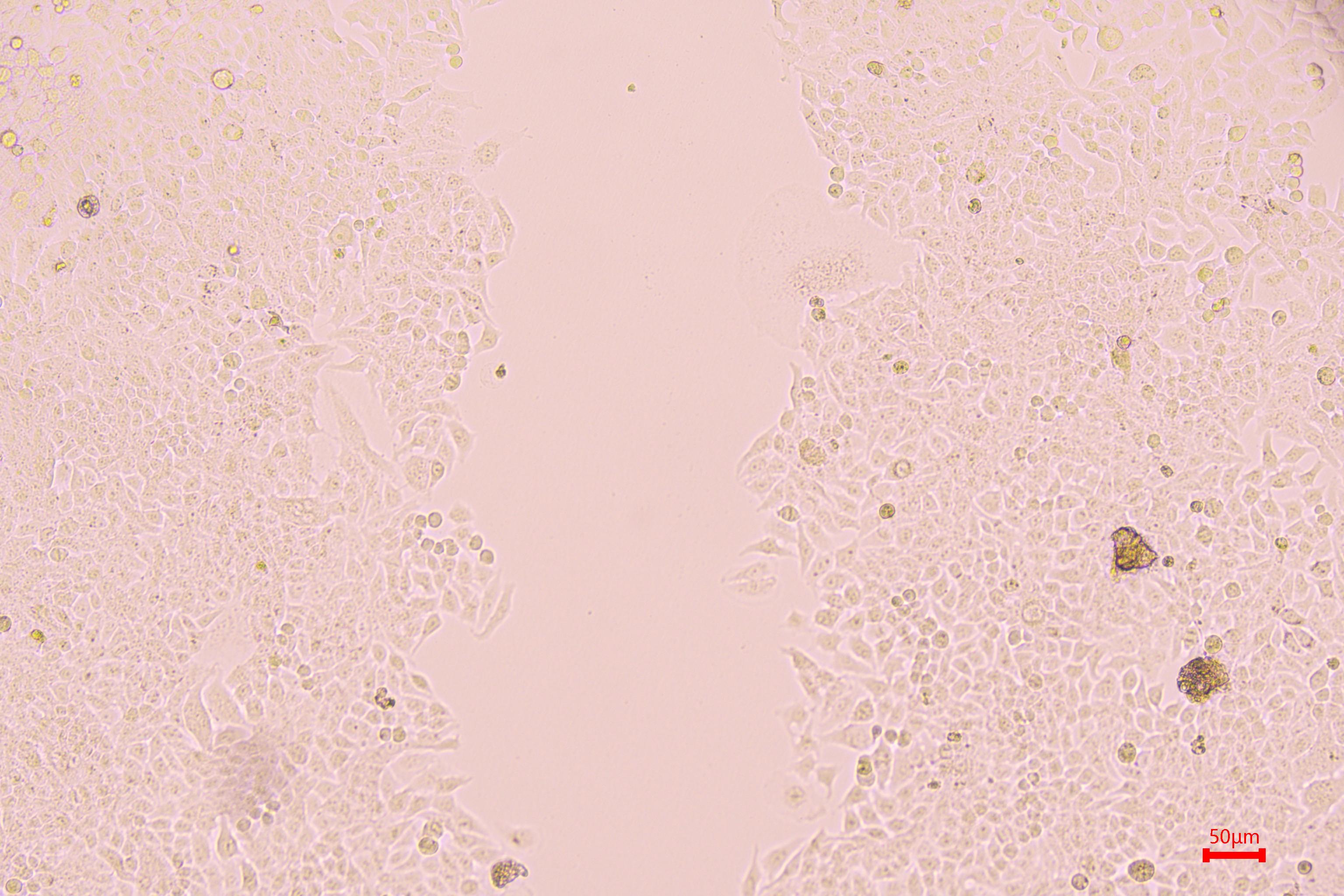

Supplement: Supplemental Information 5 [file peerj-11-14608-s005.zip › micrograph Figure2/B/A549+M1/24h (2).jpg]

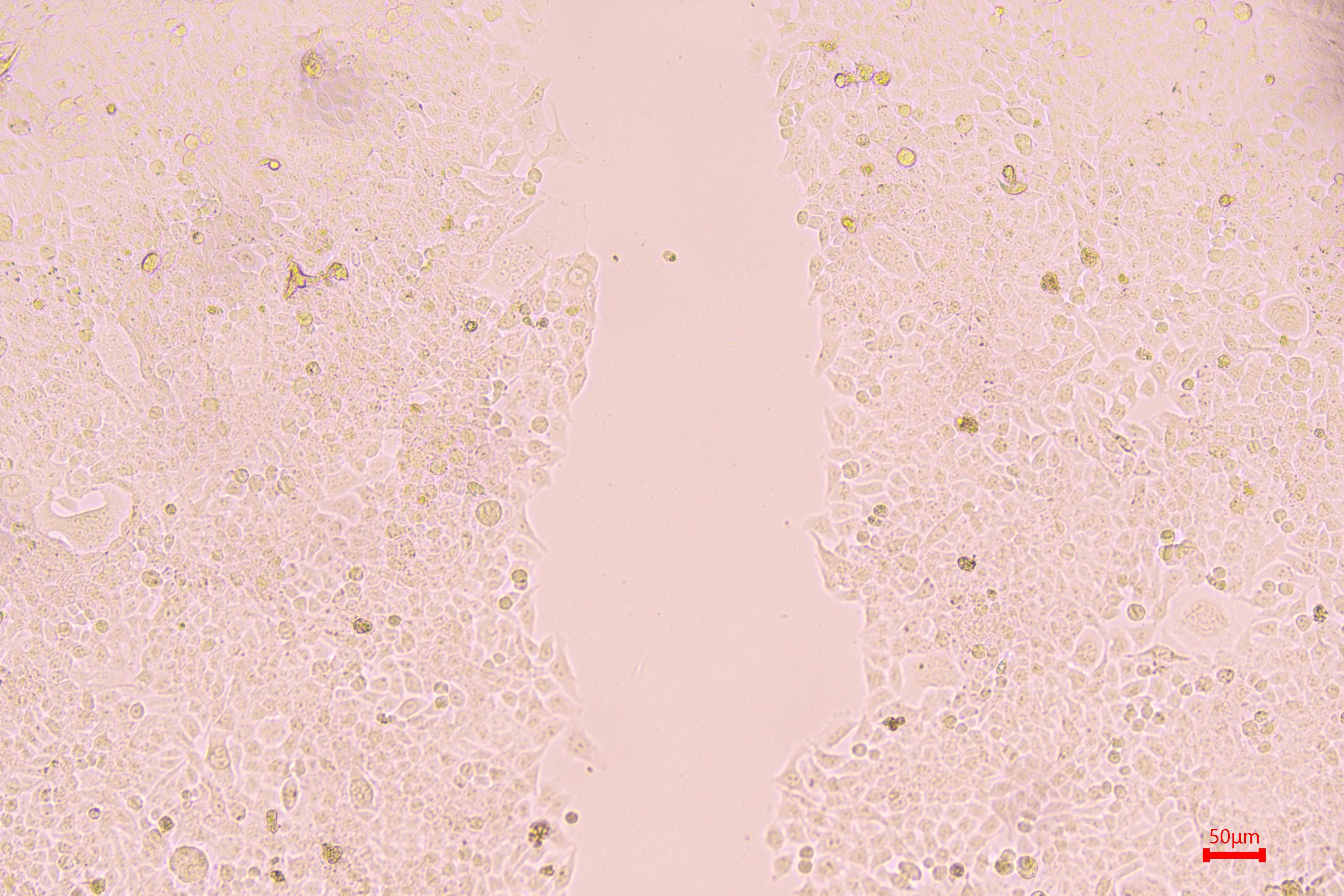

Supplement: Supplemental Information 5 [file peerj-11-14608-s005.zip › micrograph Figure2/B/A549+M1/24h (3).jpg]

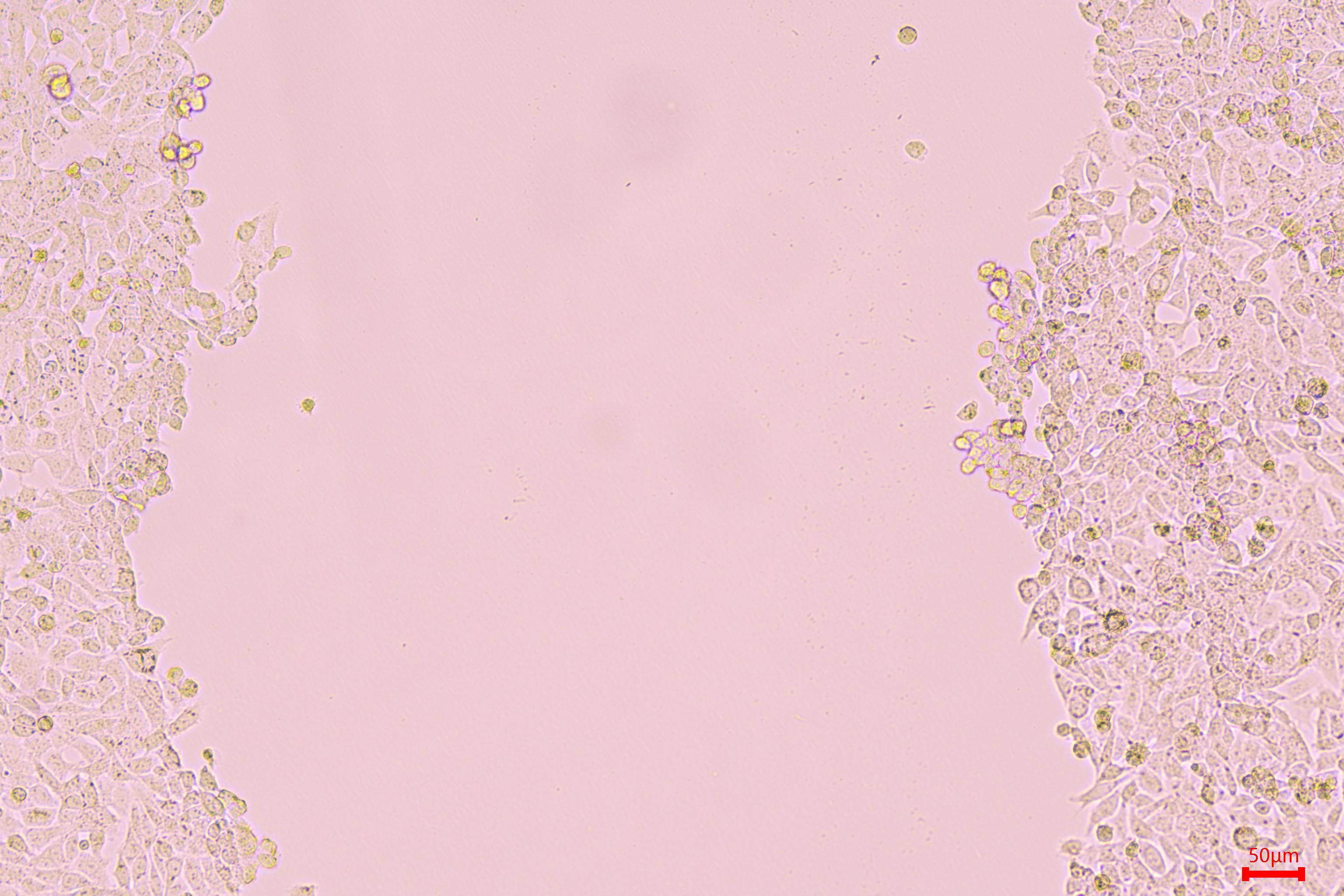

Supplement: Supplemental Information 5 [file peerj-11-14608-s005.zip › micrograph Figure2/B/HLF-A/0h (1).jpg]

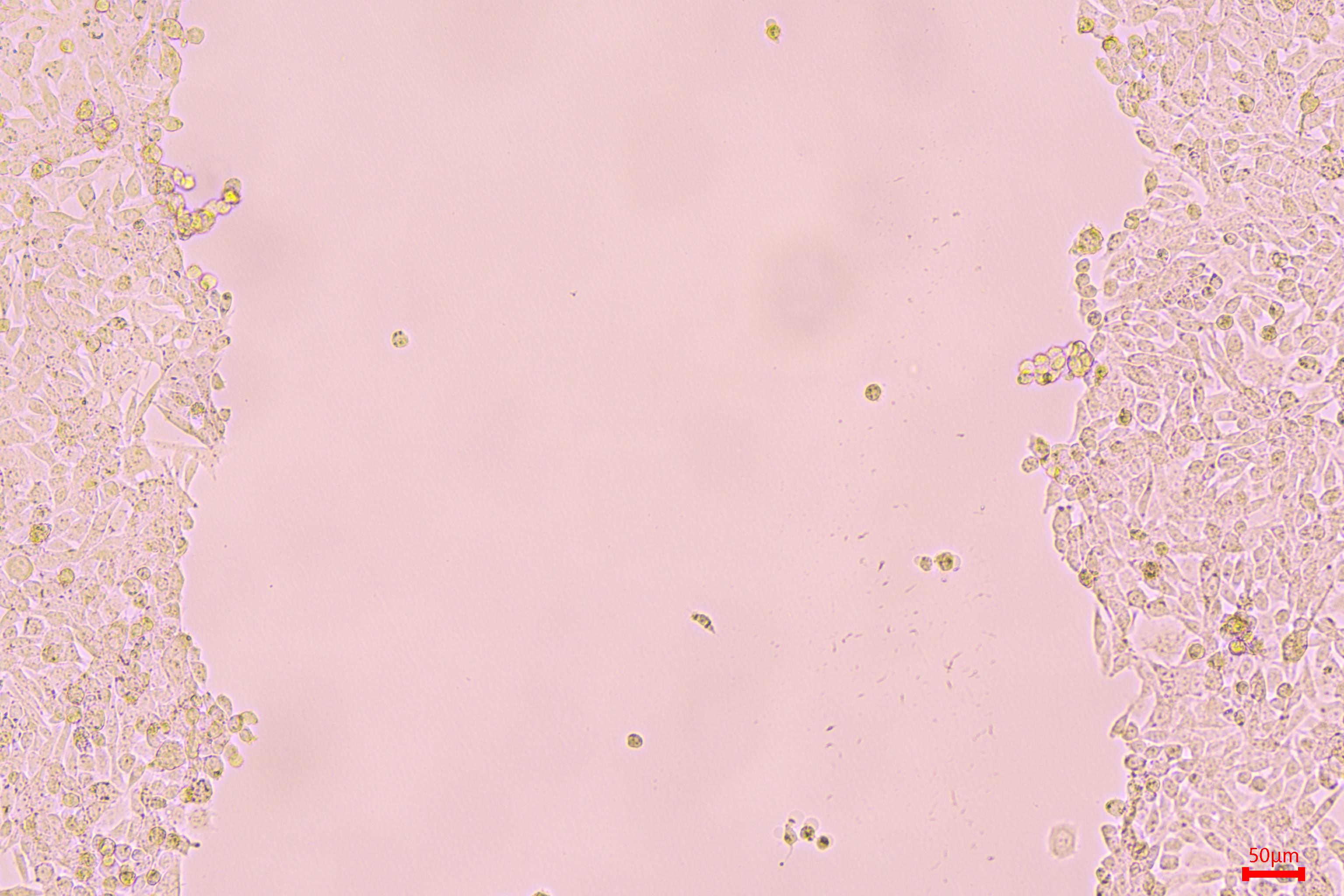

Supplement: Supplemental Information 5 [file peerj-11-14608-s005.zip › micrograph Figure2/B/HLF-A/0h (2).jpg]

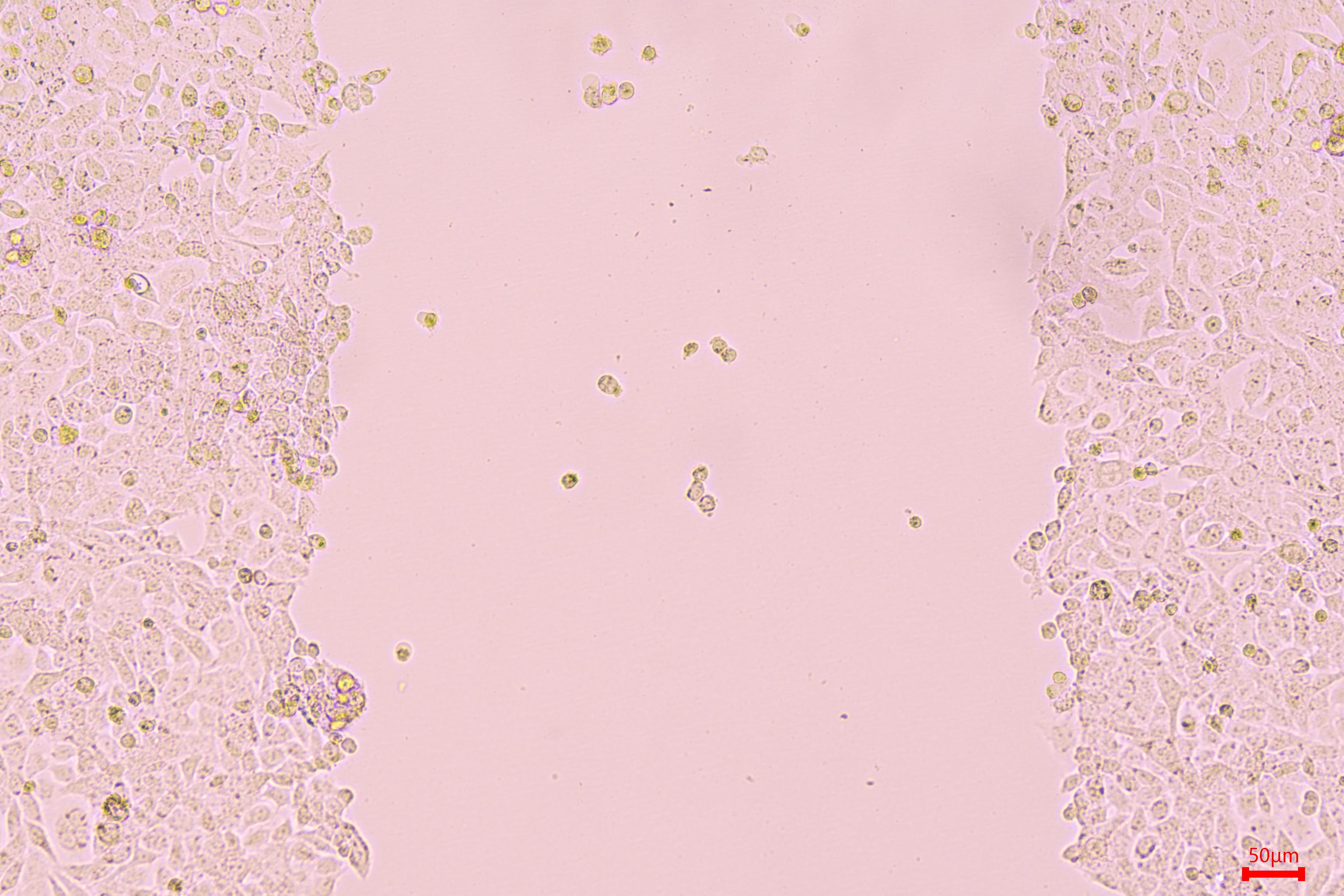

Supplement: Supplemental Information 5 [file peerj-11-14608-s005.zip › micrograph Figure2/B/HLF-A/0h (3).jpg]

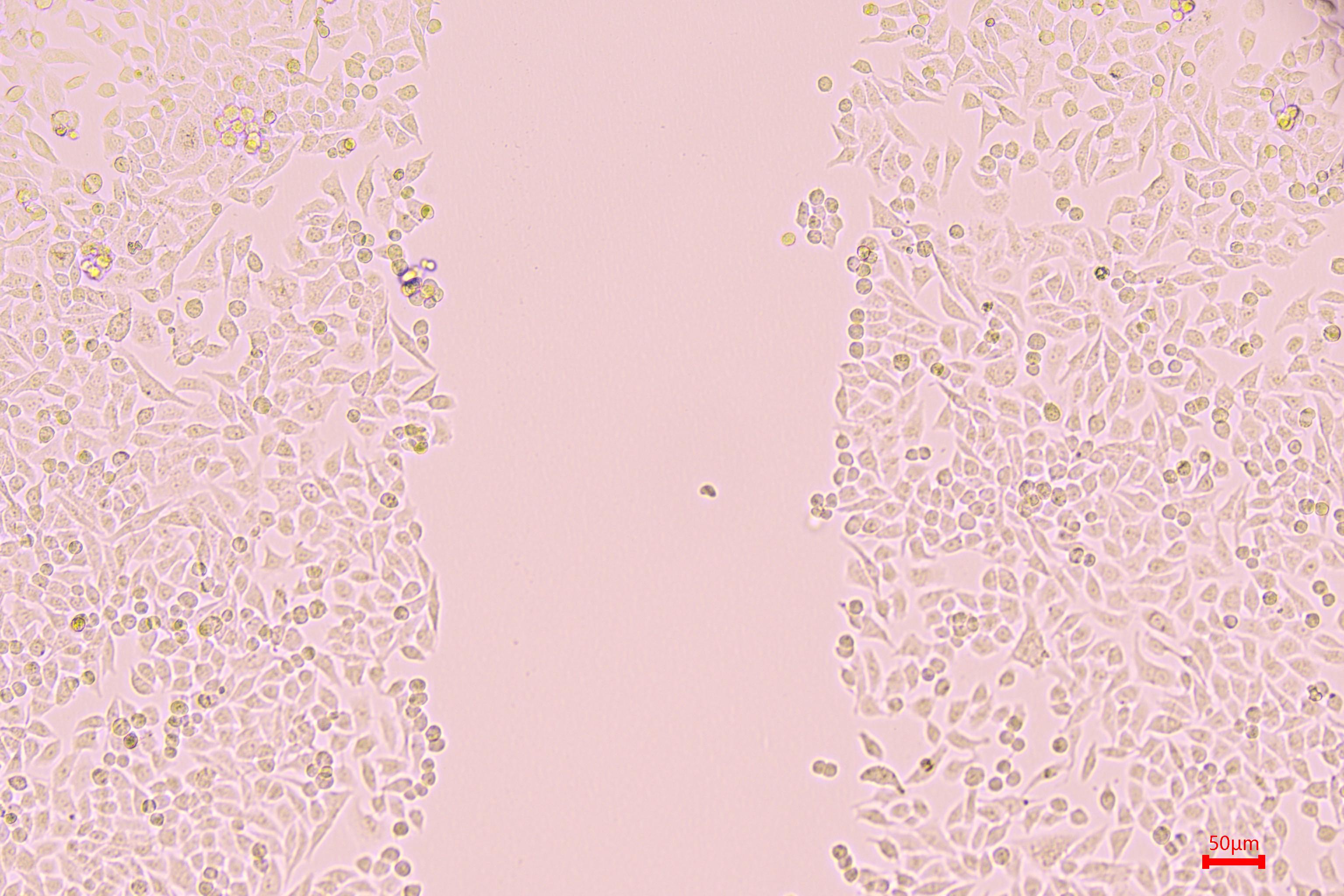

Supplement: Supplemental Information 5 [file peerj-11-14608-s005.zip › micrograph Figure2/B/HLF-A/24h (1).jpg]

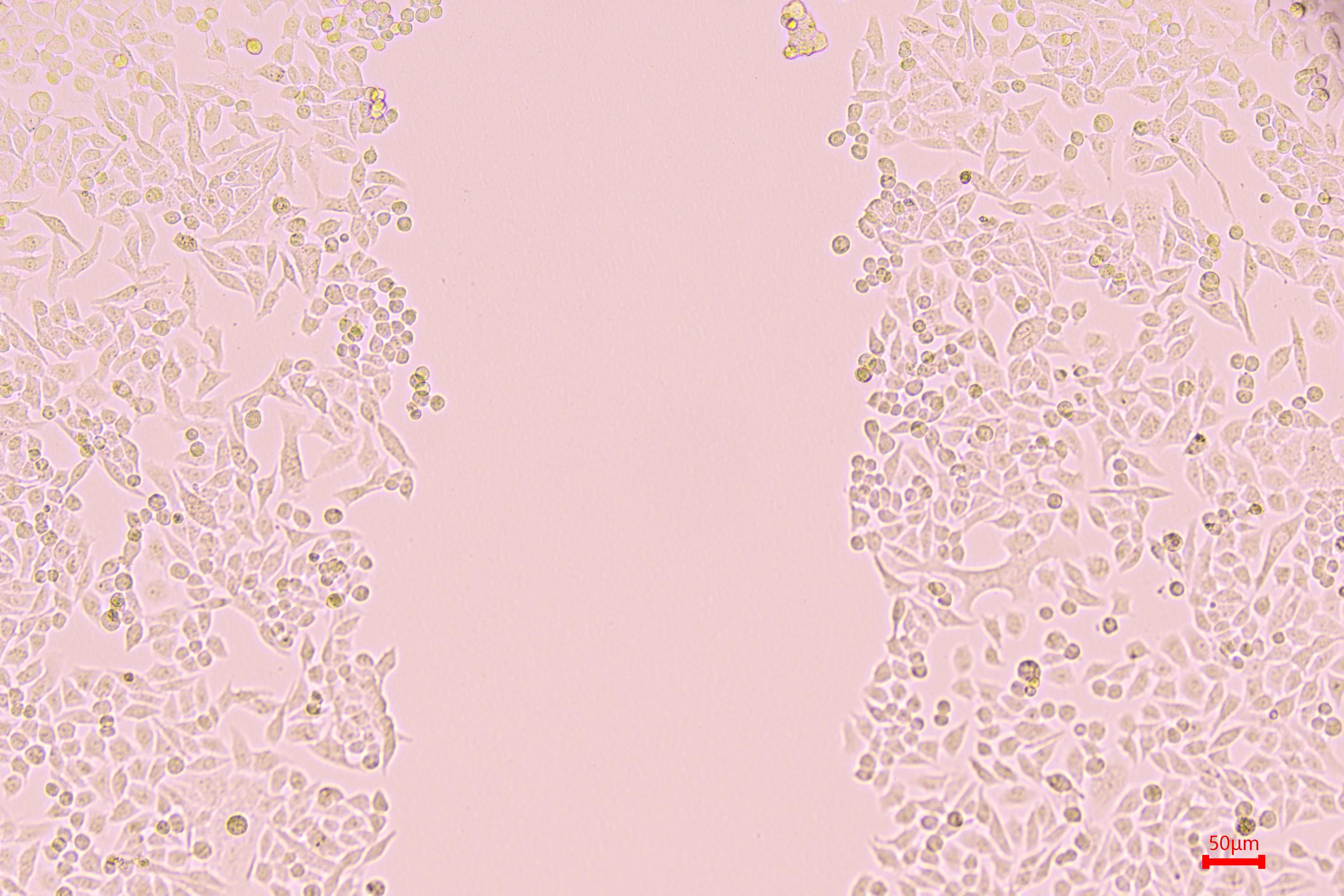

Supplement: Supplemental Information 5 [file peerj-11-14608-s005.zip › micrograph Figure2/B/HLF-A/24h (2).jpg]

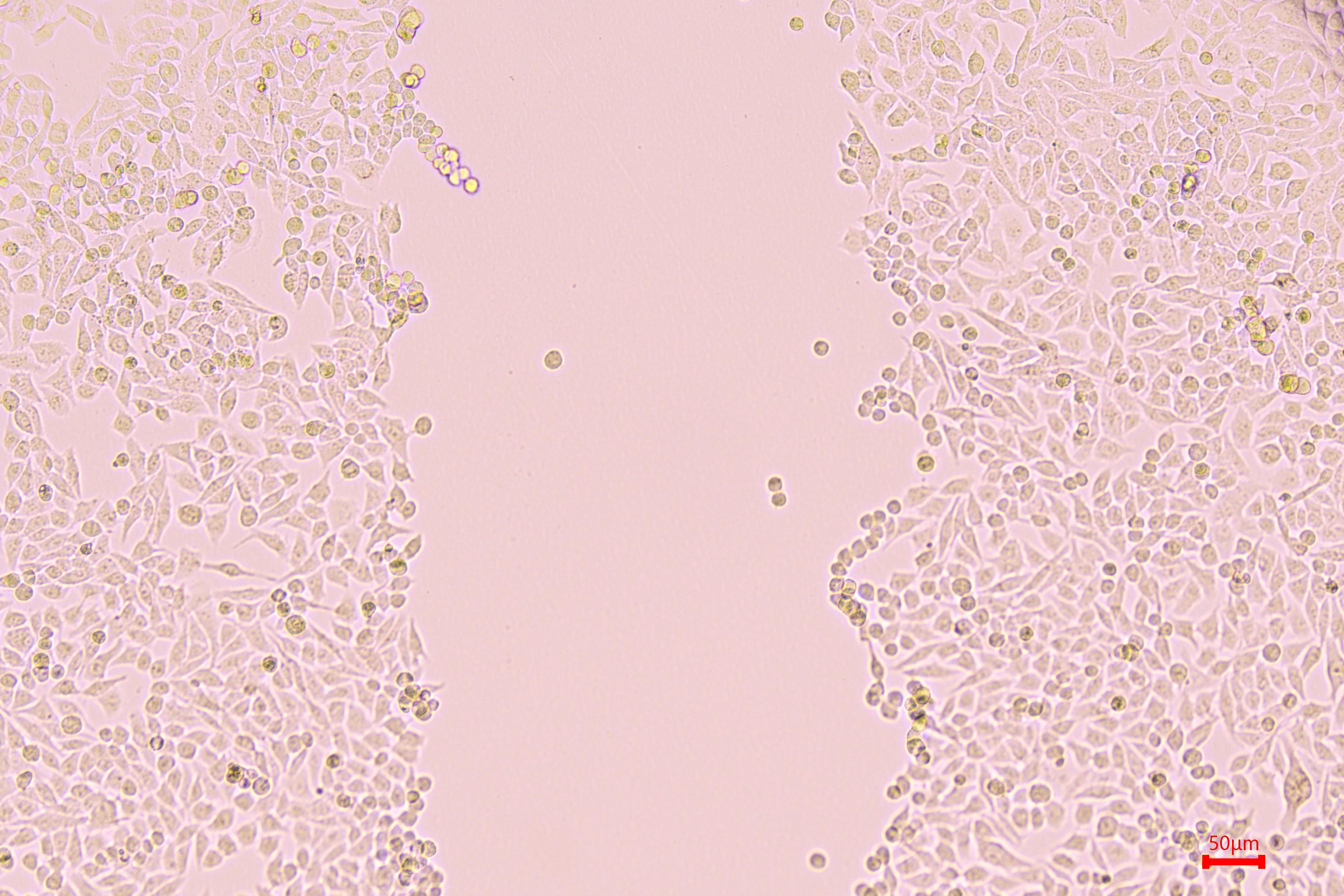

Supplement: Supplemental Information 5 [file peerj-11-14608-s005.zip › micrograph Figure2/B/HLF-A/24h (3).jpg]

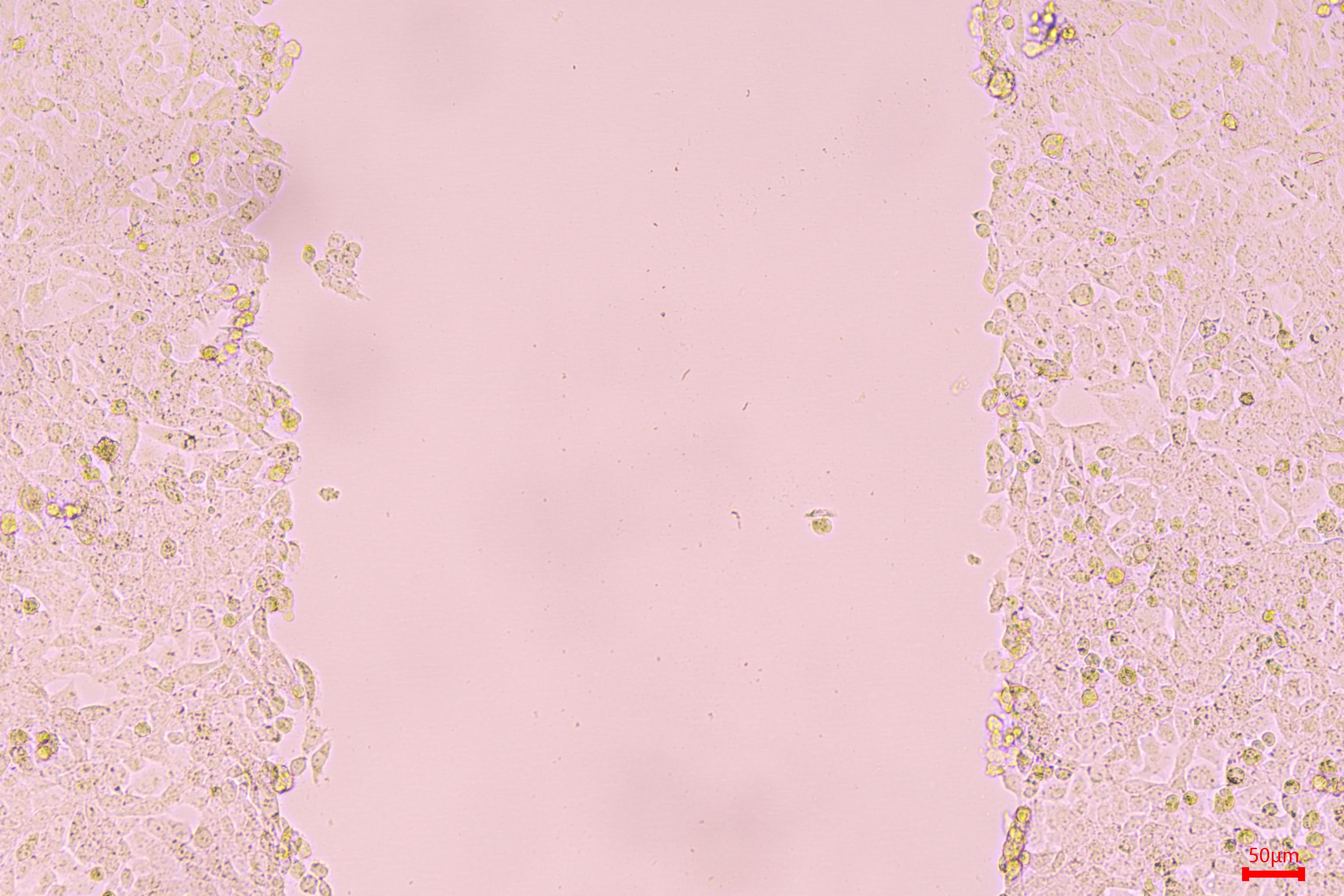

Supplement: Supplemental Information 5 [file peerj-11-14608-s005.zip › micrograph Figure2/B/HLF-A+M1/0h (1).jpg]

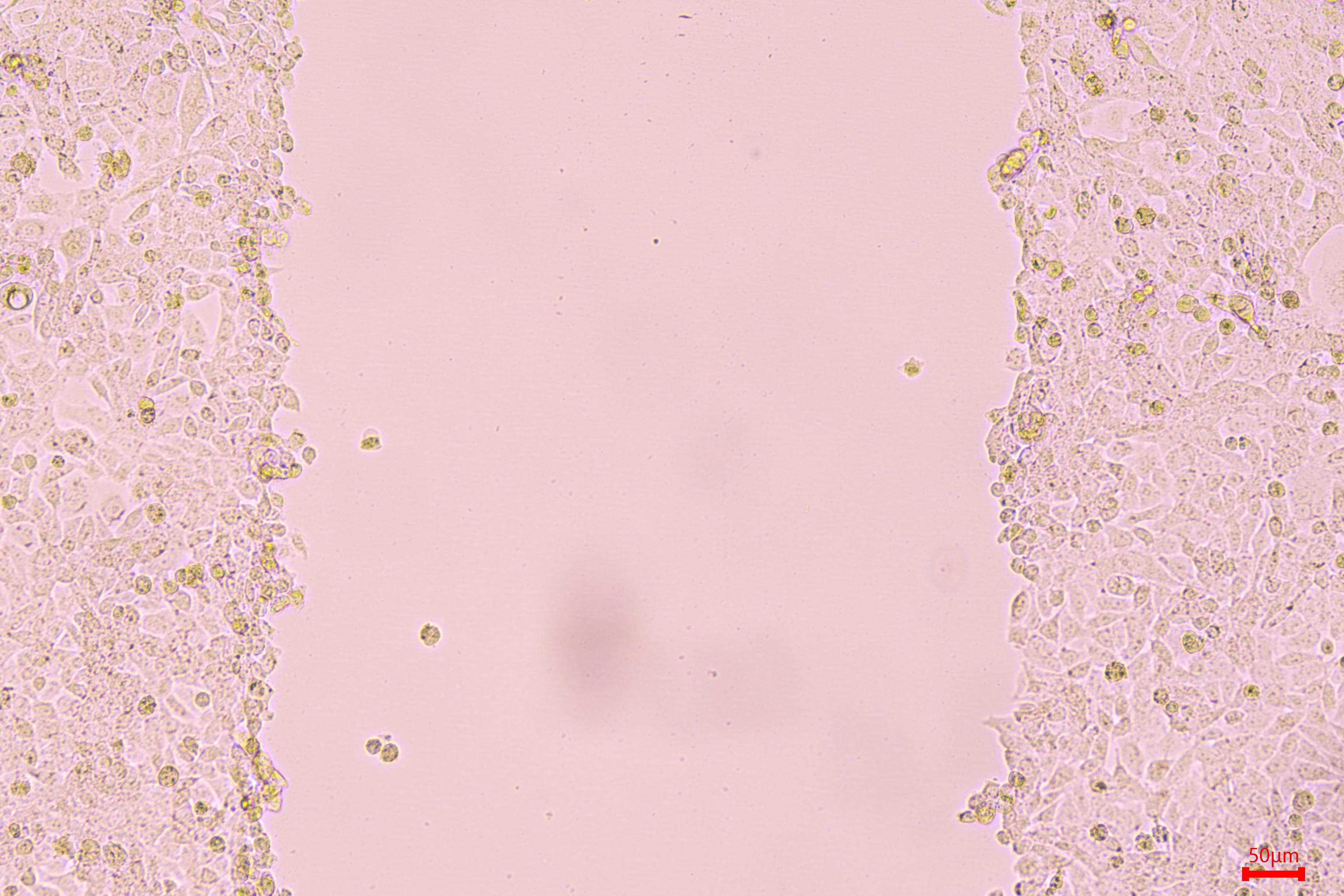

Supplement: Supplemental Information 5 [file peerj-11-14608-s005.zip › micrograph Figure2/B/HLF-A+M1/0h (2).jpg]

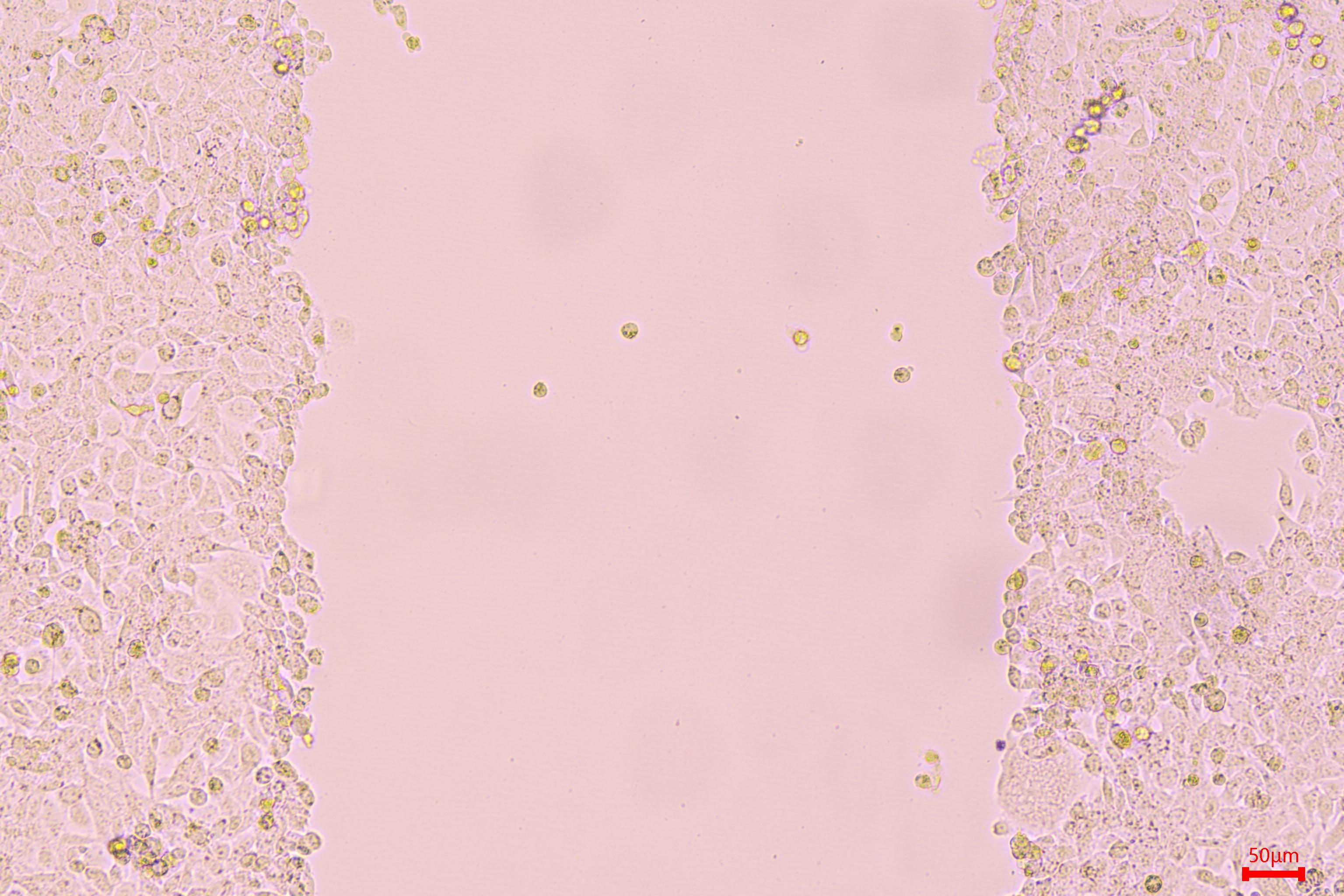

Supplement: Supplemental Information 5 [file peerj-11-14608-s005.zip › micrograph Figure2/B/HLF-A+M1/0h (3).jpg]

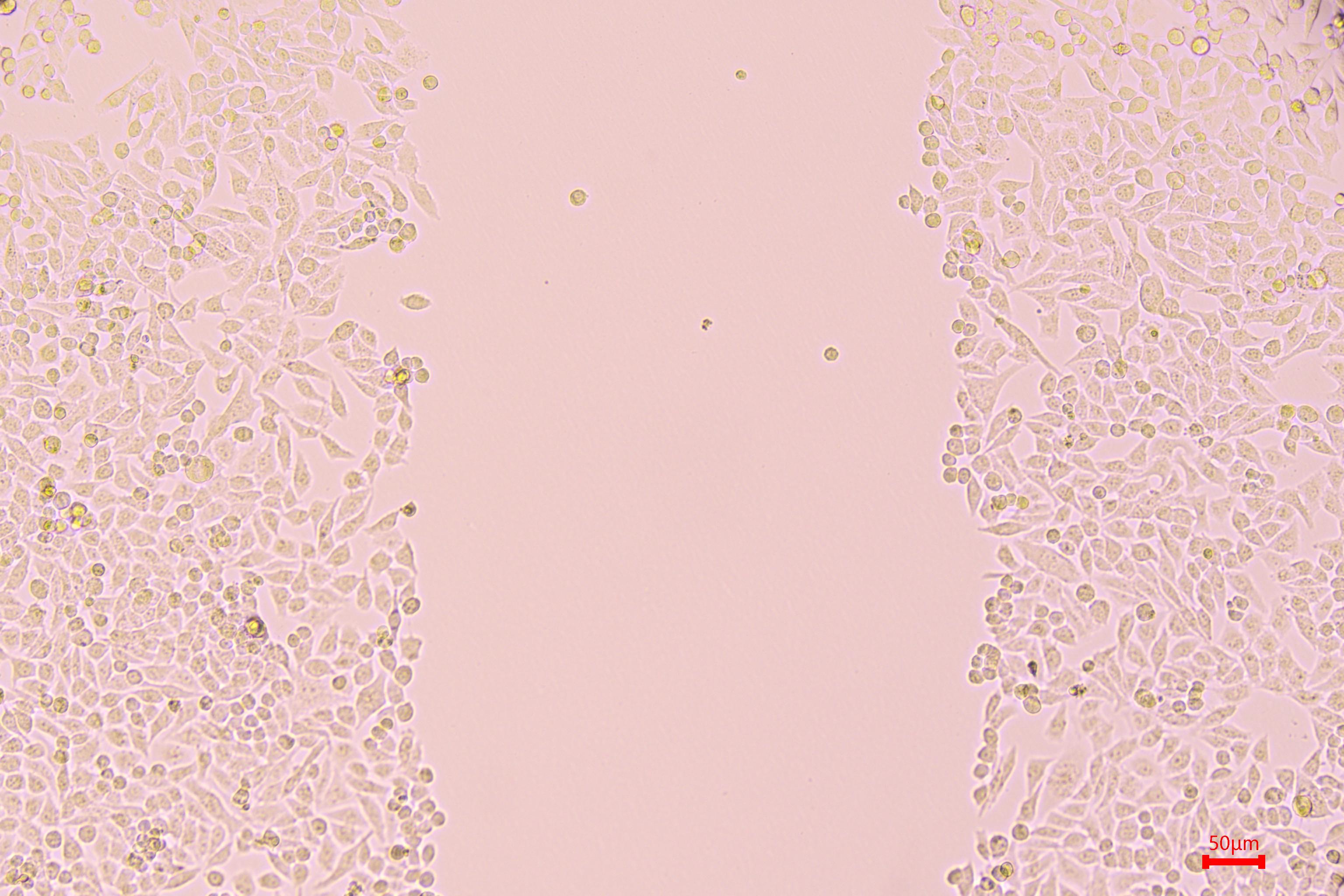

Supplement: Supplemental Information 5 [file peerj-11-14608-s005.zip › micrograph Figure2/B/HLF-A+M1/24h (1).jpg]

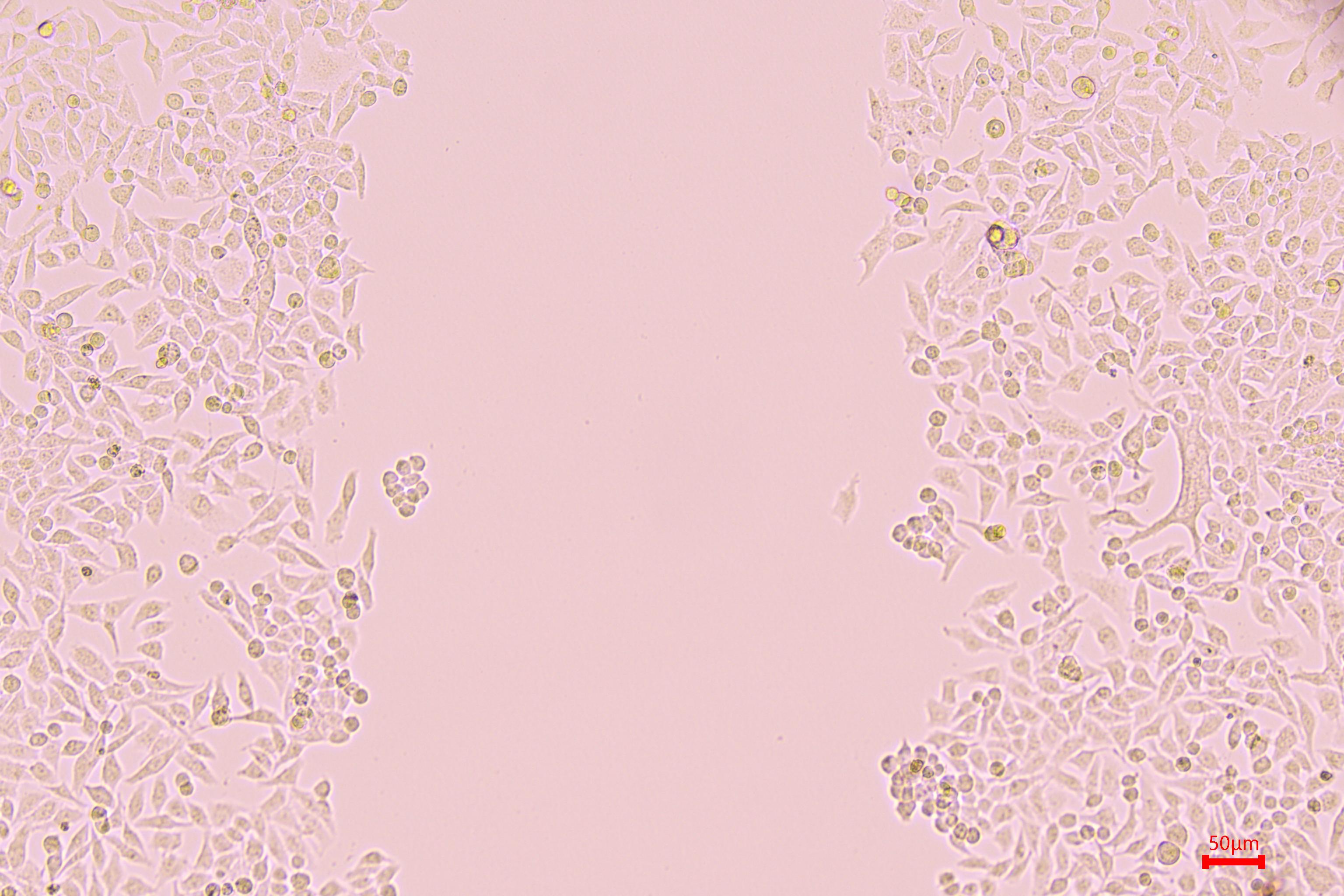

Supplement: Supplemental Information 5 [file peerj-11-14608-s005.zip › micrograph Figure2/B/HLF-A+M1/24h (2).jpg]

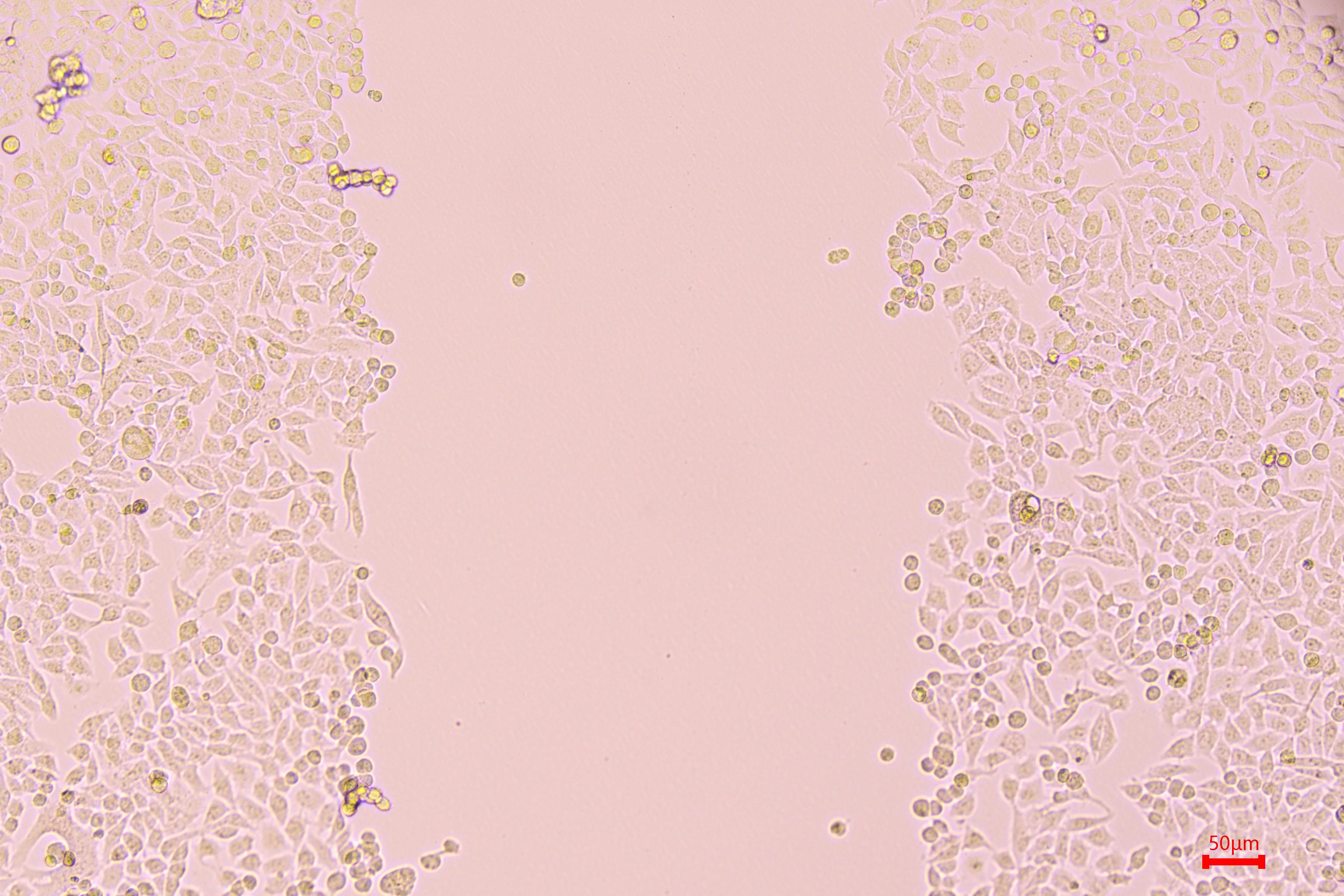

Supplement: Supplemental Information 5 [file peerj-11-14608-s005.zip › micrograph Figure2/B/HLF-A+M1/24h (3).jpg]

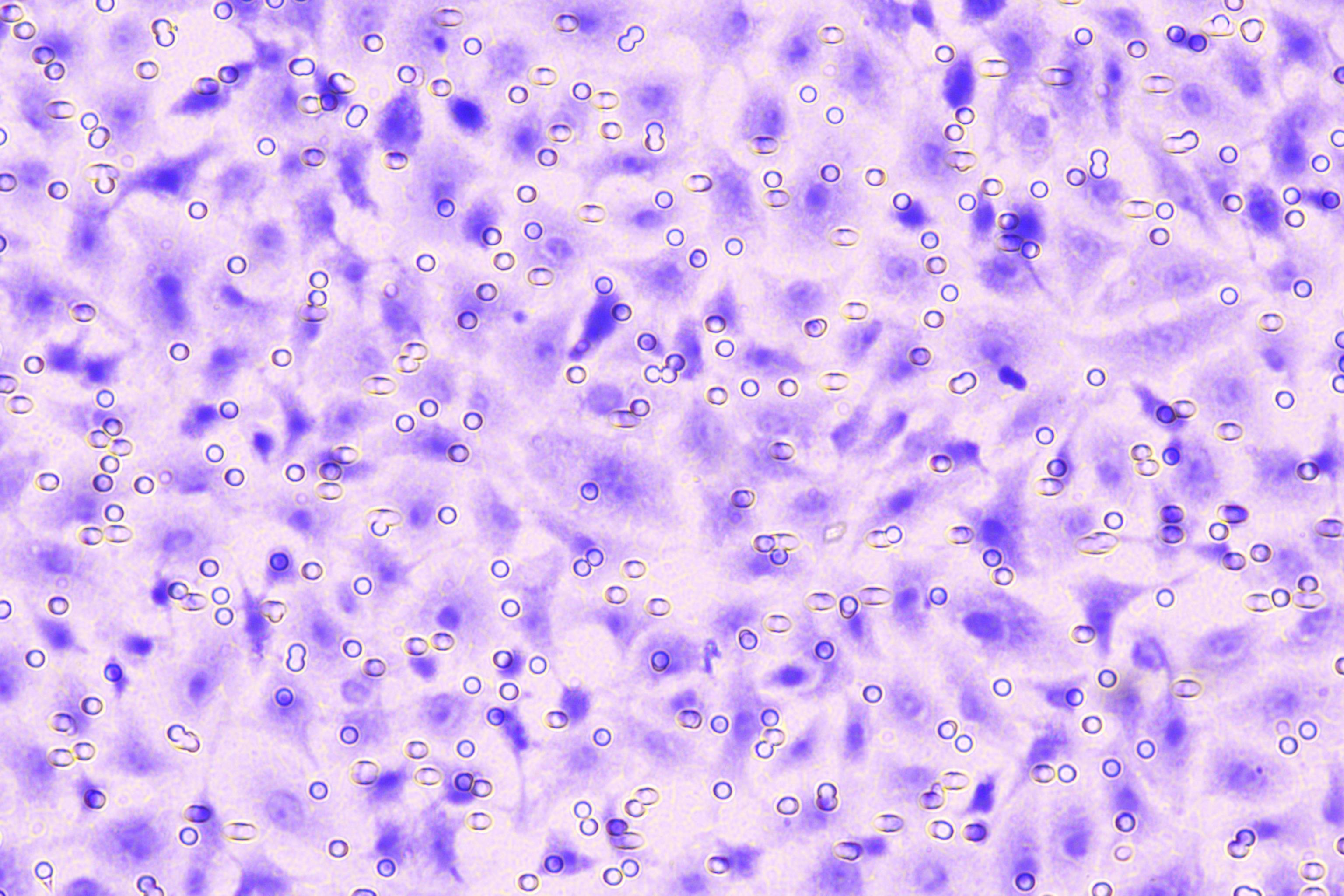

Supplement: Supplemental Information 5 [file peerj-11-14608-s005.zip › micrograph Figure2/C/A549/1.jpg]

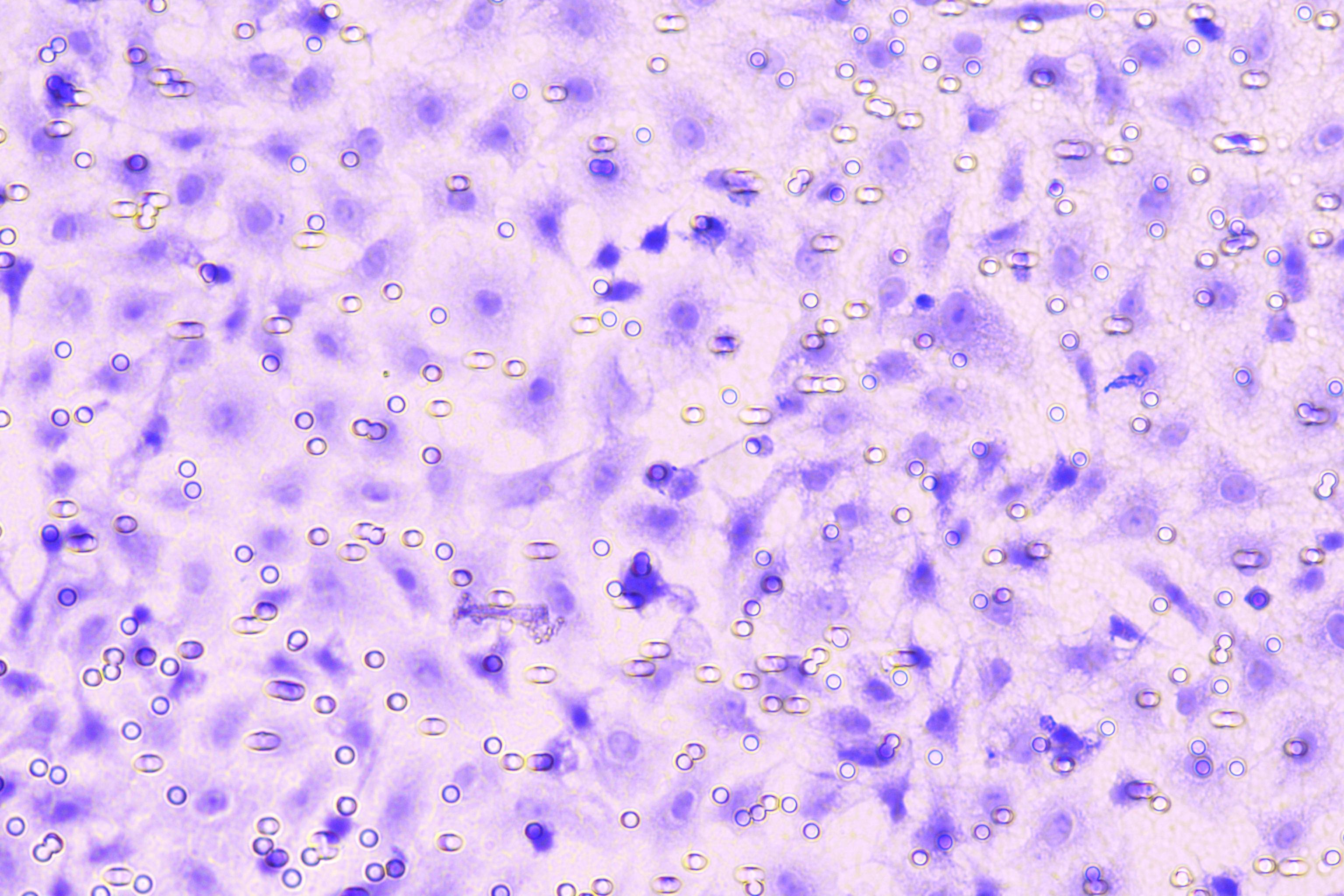

Supplement: Supplemental Information 5 [file peerj-11-14608-s005.zip › micrograph Figure2/C/A549/2.jpg]

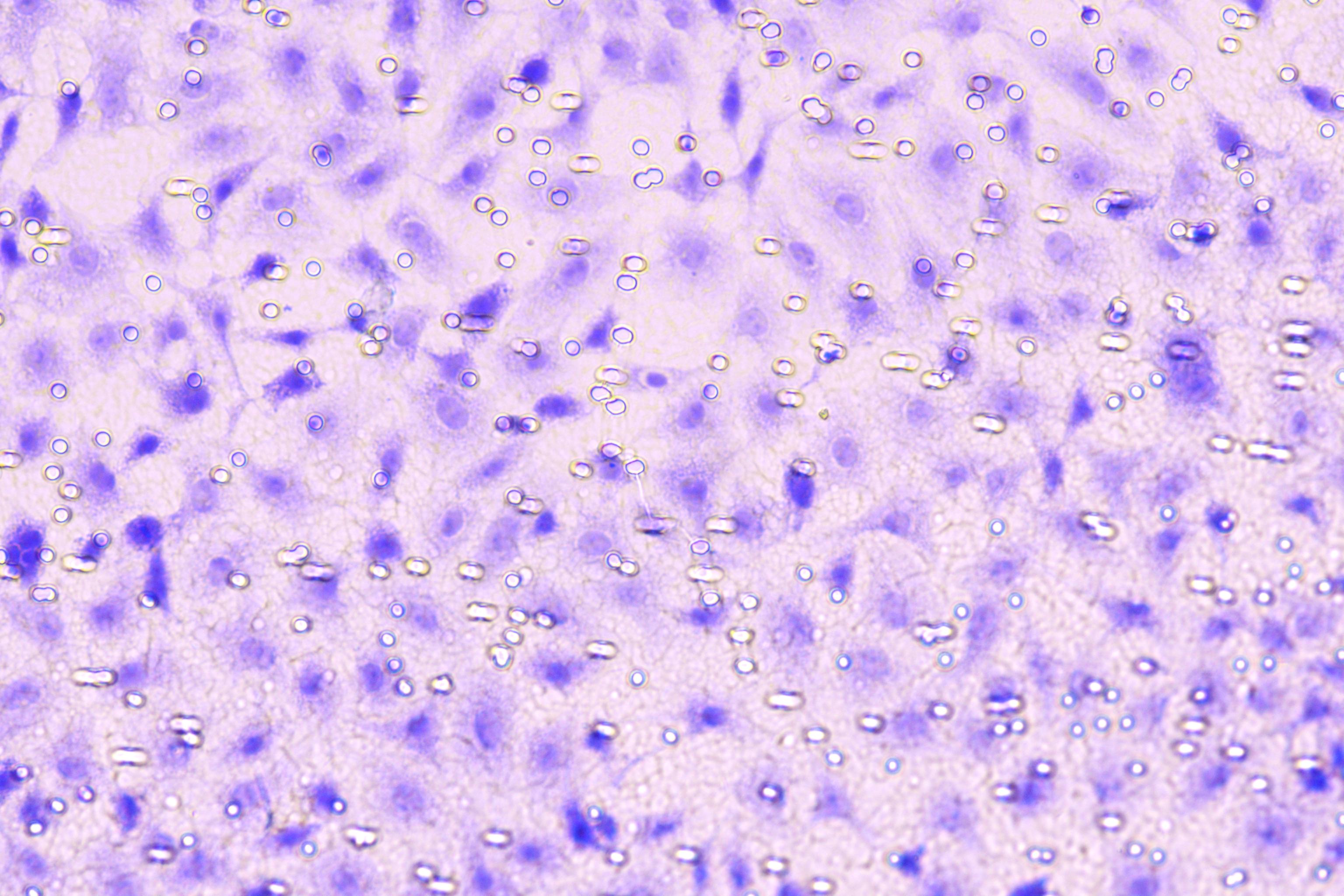

Supplement: Supplemental Information 5 [file peerj-11-14608-s005.zip › micrograph Figure2/C/A549/3.jpg]

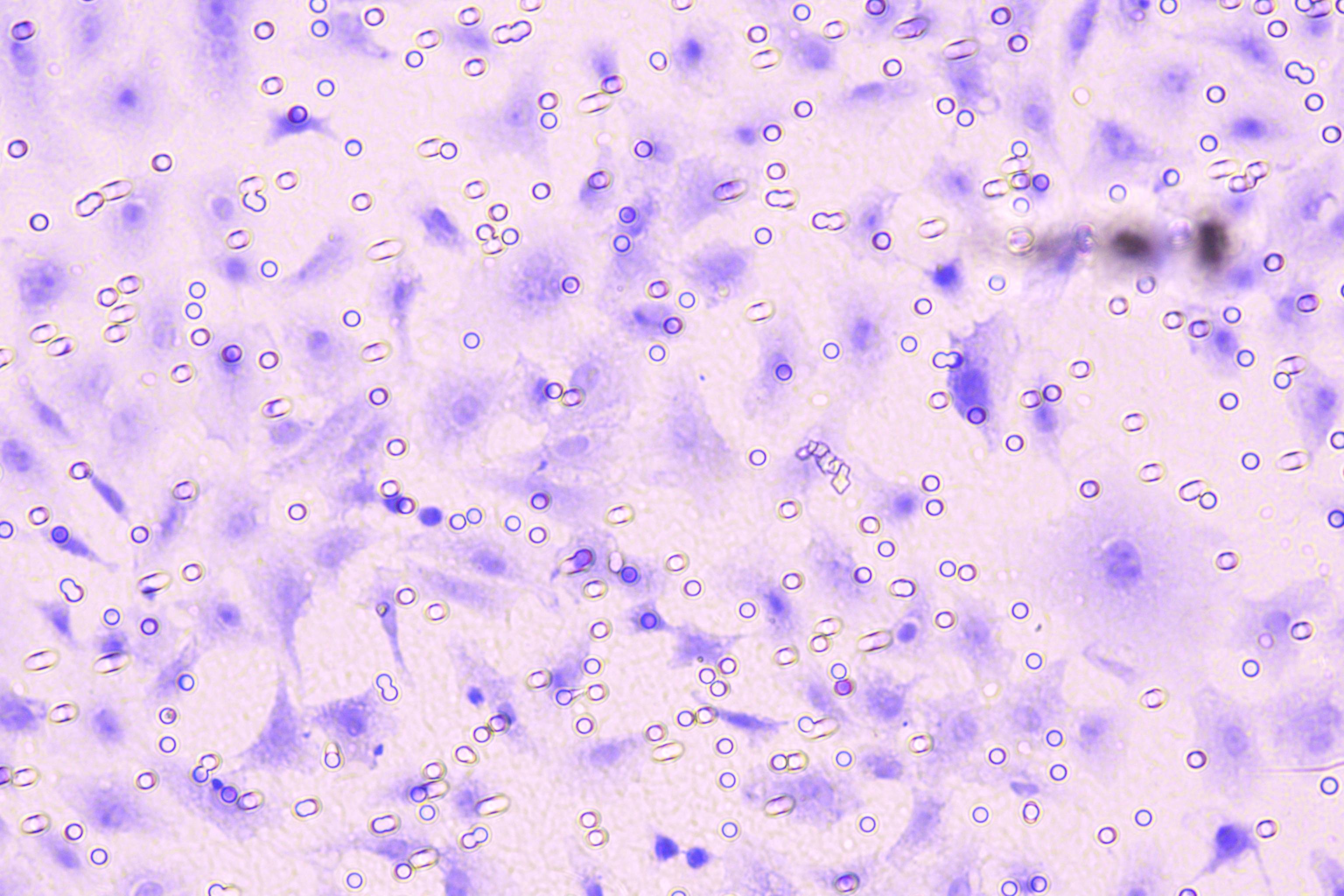

Supplement: Supplemental Information 5 [file peerj-11-14608-s005.zip › micrograph Figure2/C/A549+M1/1.jpg]

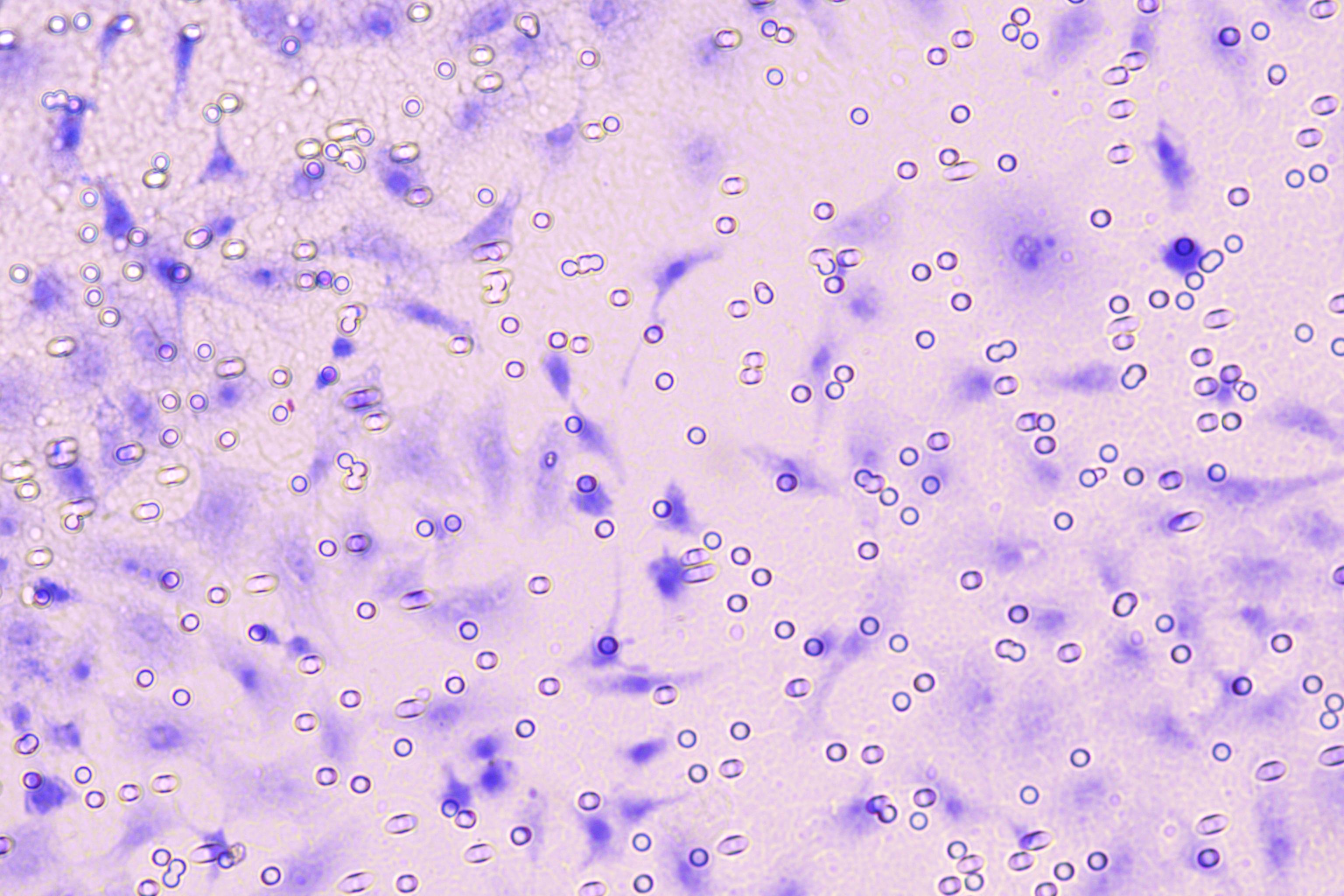

Supplement: Supplemental Information 5 [file peerj-11-14608-s005.zip › micrograph Figure2/C/A549+M1/2.jpg]

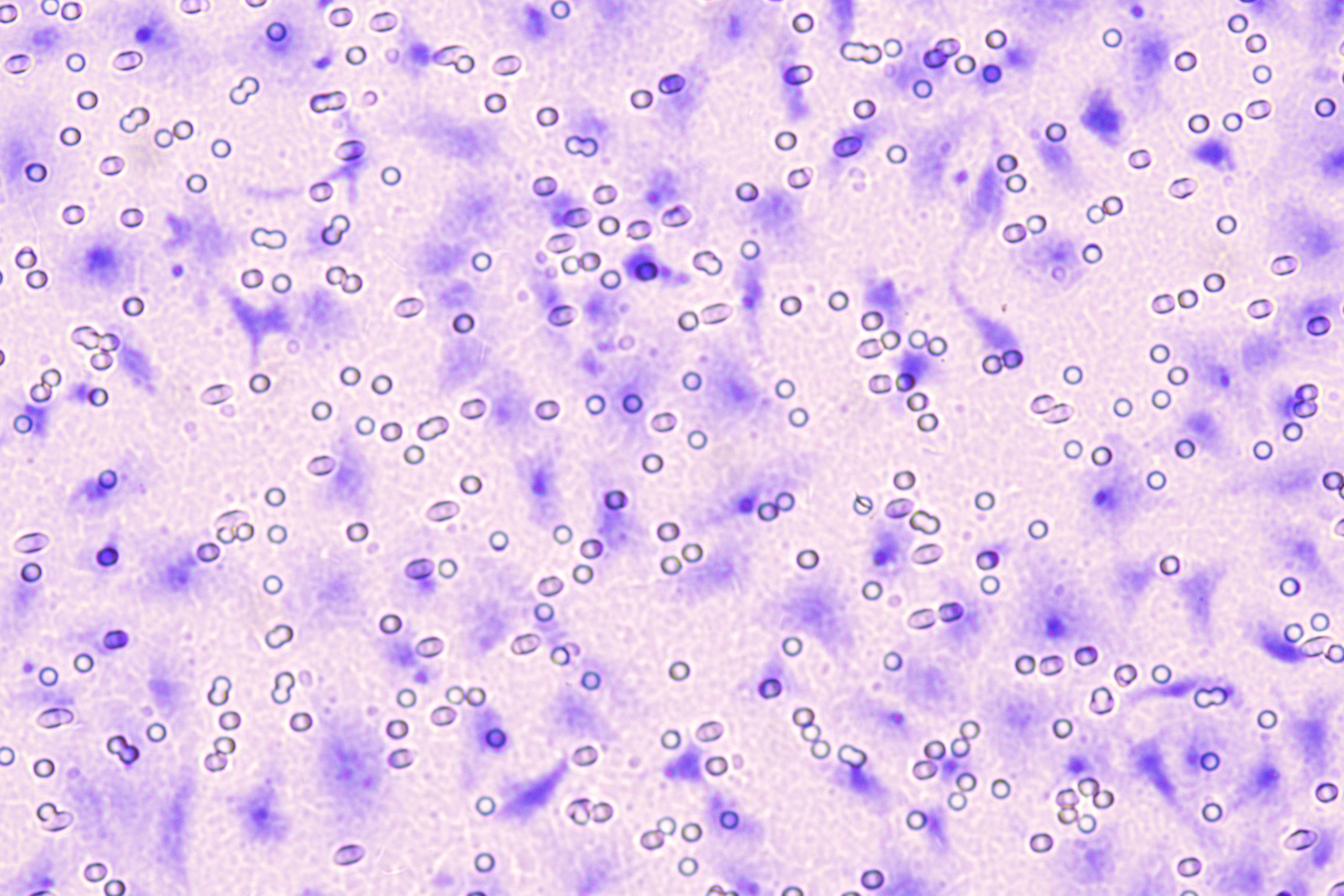

Supplement: Supplemental Information 5 [file peerj-11-14608-s005.zip › micrograph Figure2/C/A549+M1/3.jpg]

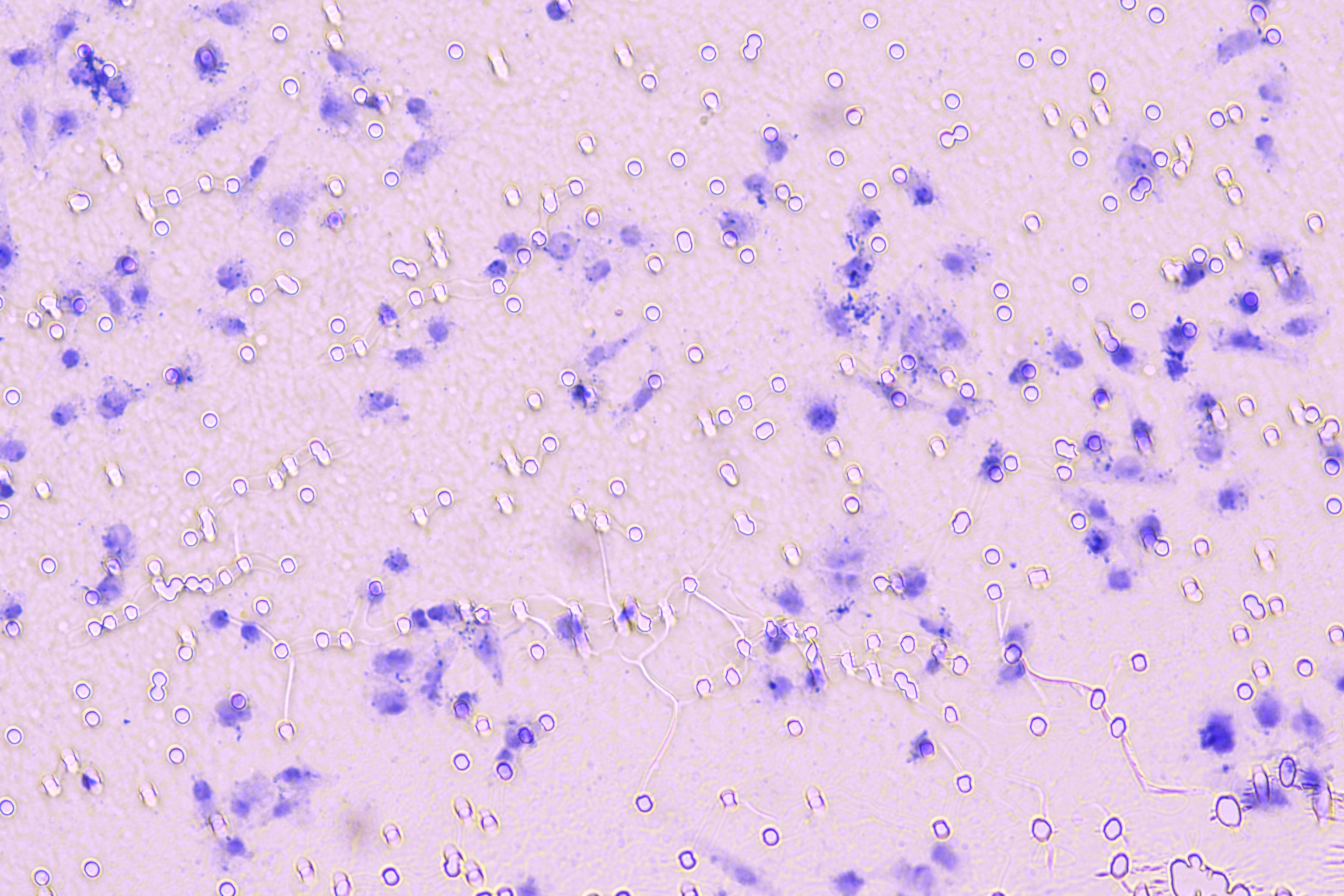

Supplement: Supplemental Information 5 [file peerj-11-14608-s005.zip › micrograph Figure2/C/HLF-A/1.jpg]

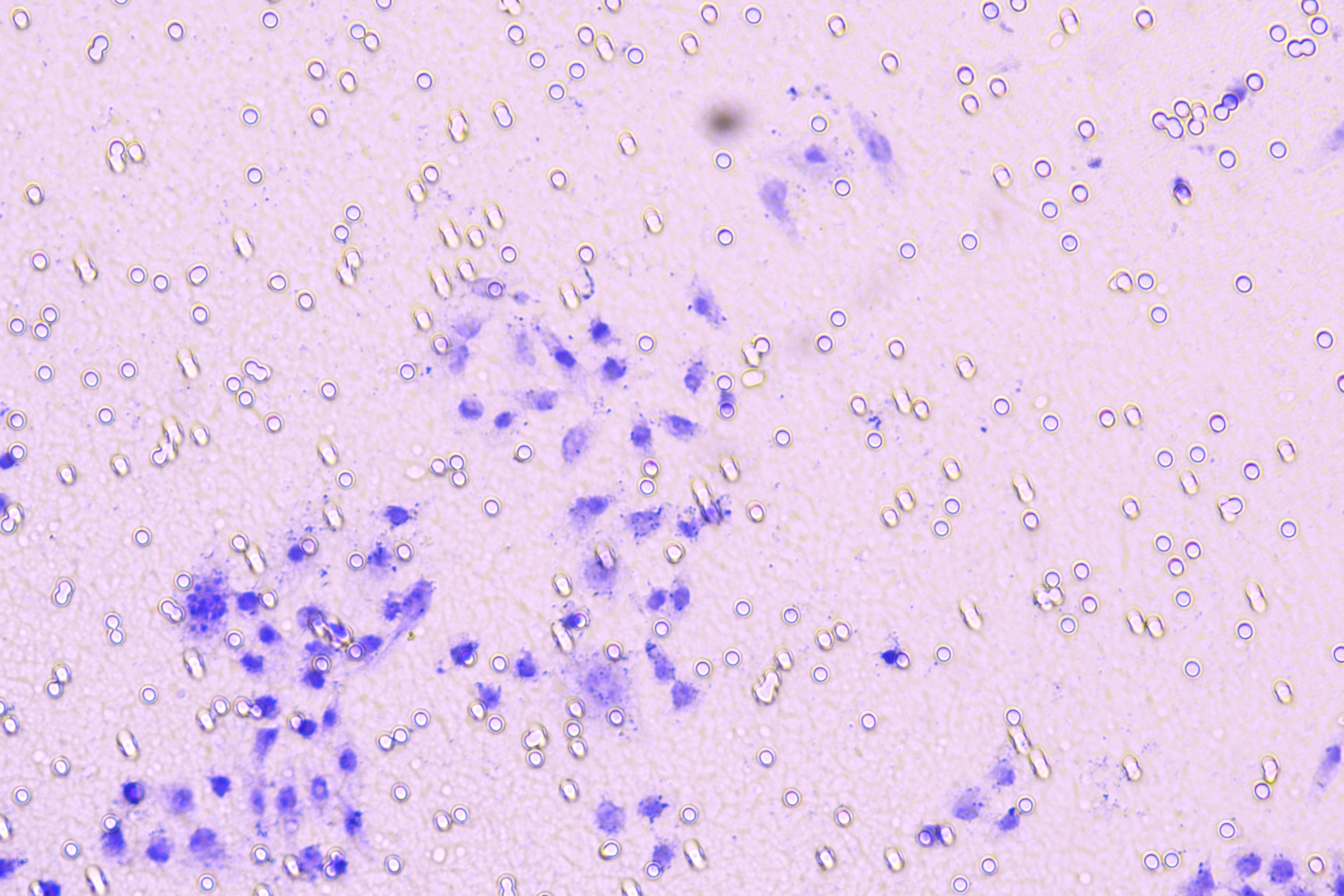

Supplement: Supplemental Information 5 [file peerj-11-14608-s005.zip › micrograph Figure2/C/HLF-A/2.jpg]

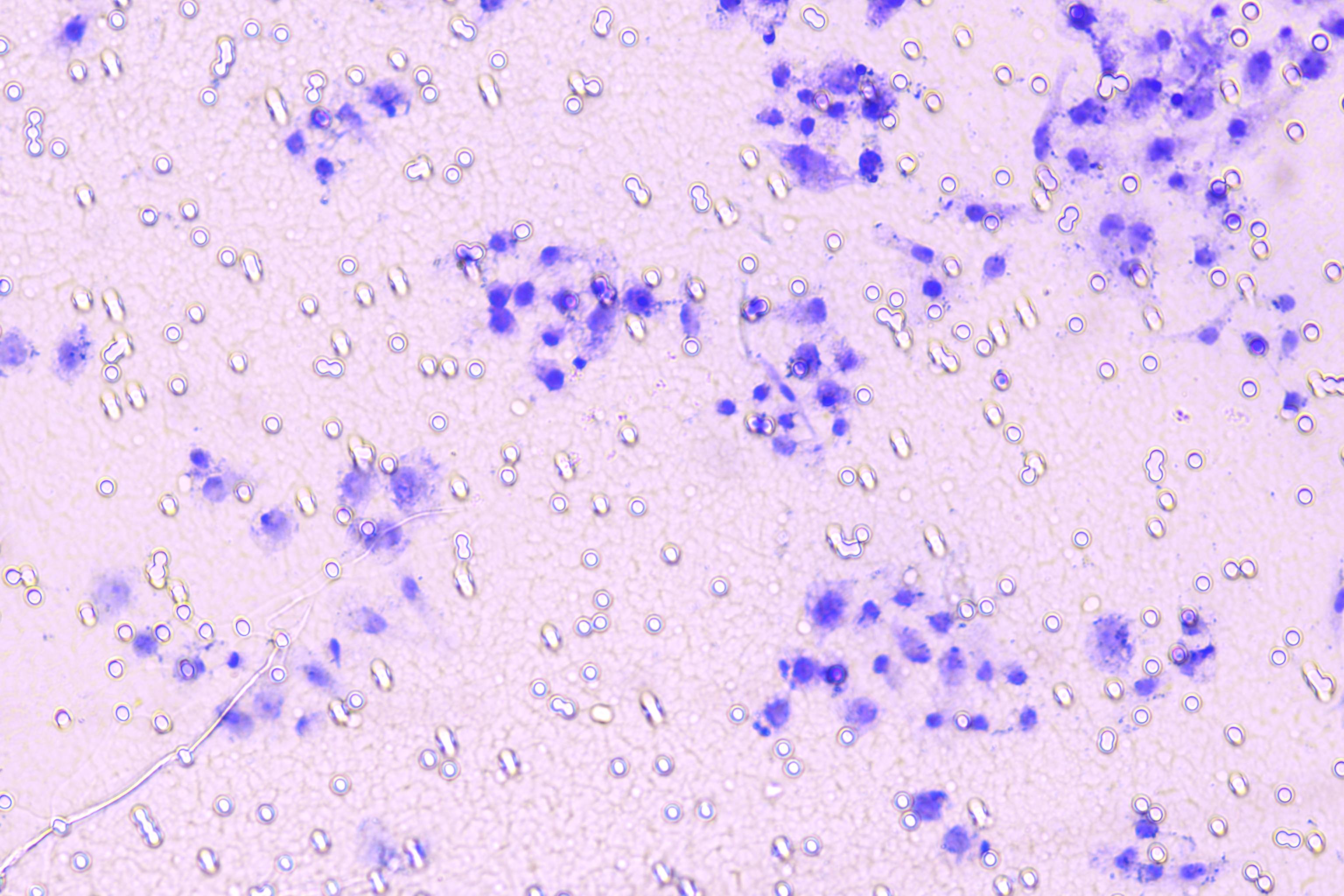

Supplement: Supplemental Information 5 [file peerj-11-14608-s005.zip › micrograph Figure2/C/HLF-A/3.jpg]

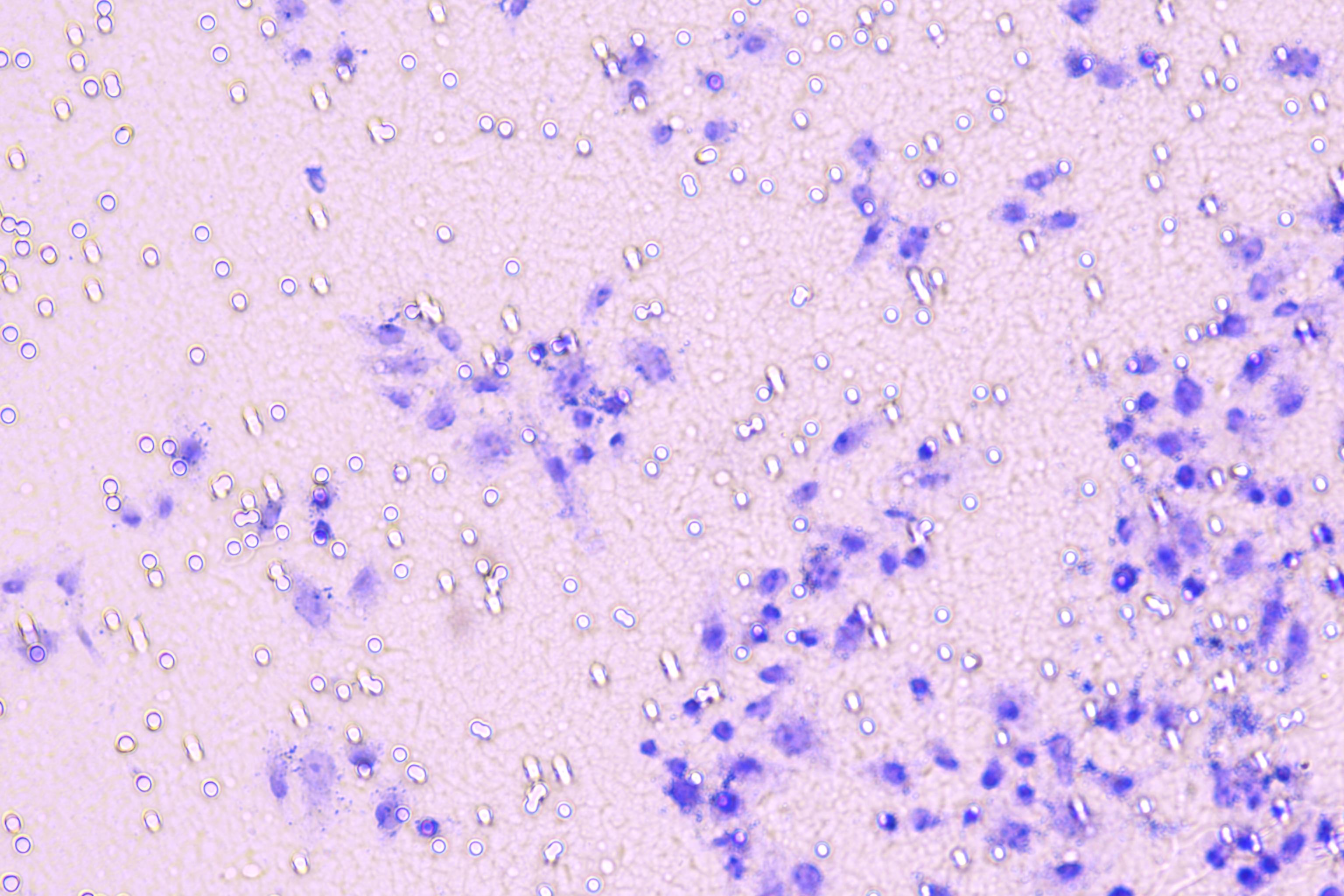

Supplement: Supplemental Information 5 [file peerj-11-14608-s005.zip › micrograph Figure2/C/HLF-A+M1/1.jpg]

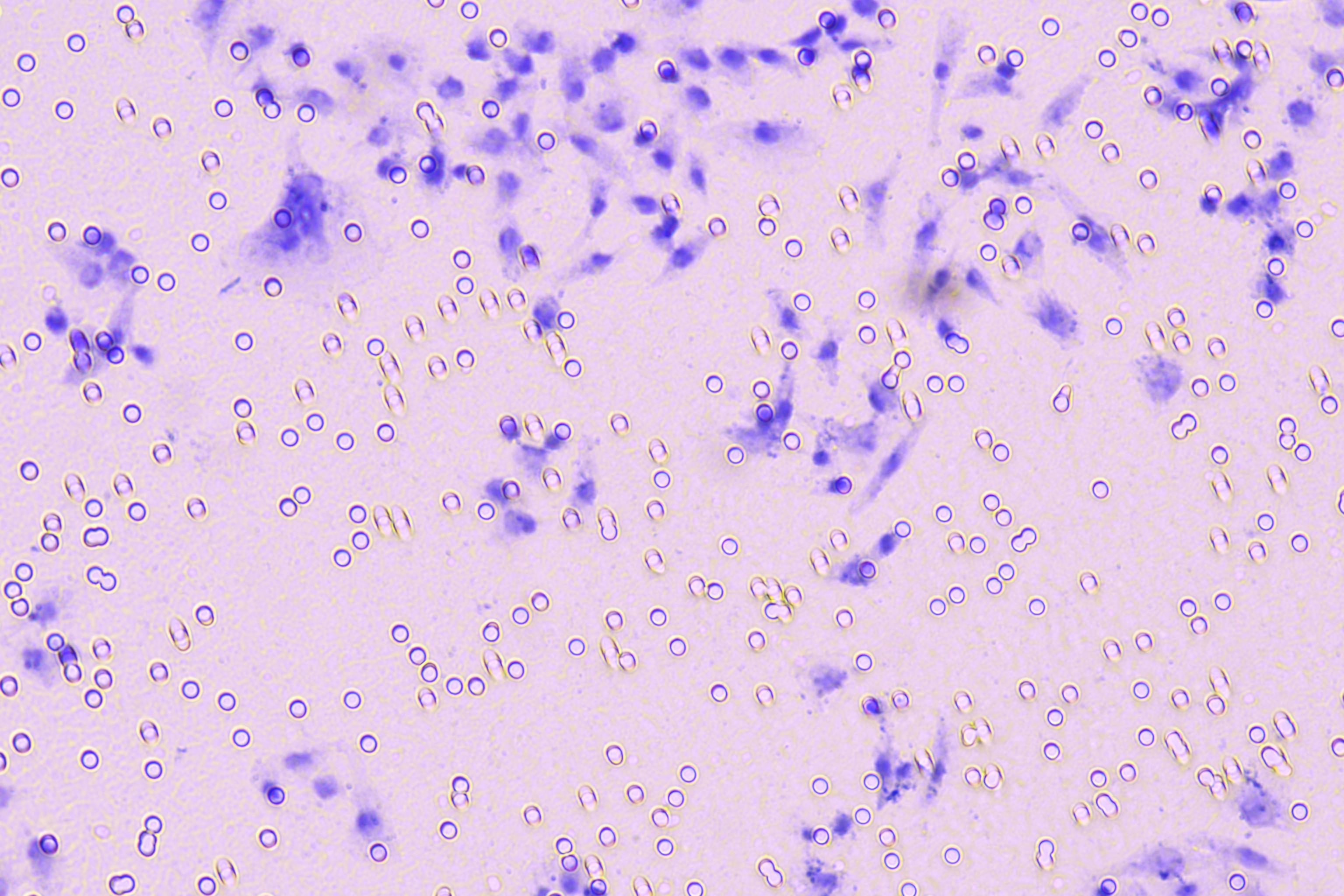

Supplement: Supplemental Information 5 [file peerj-11-14608-s005.zip › micrograph Figure2/C/HLF-A+M1/2.jpg]

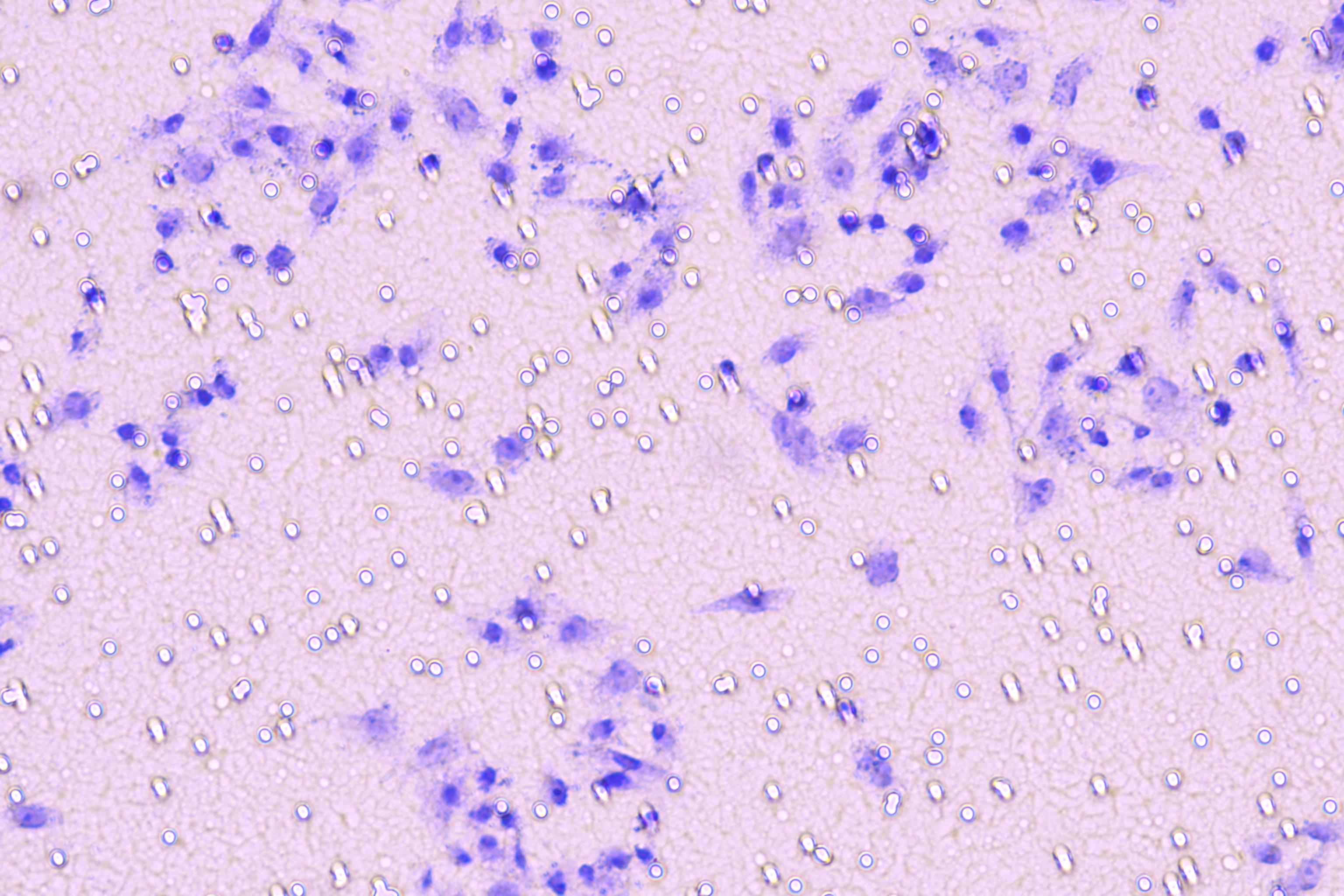

Supplement: Supplemental Information 5 [file peerj-11-14608-s005.zip › micrograph Figure2/C/HLF-A+M1/3.jpg]

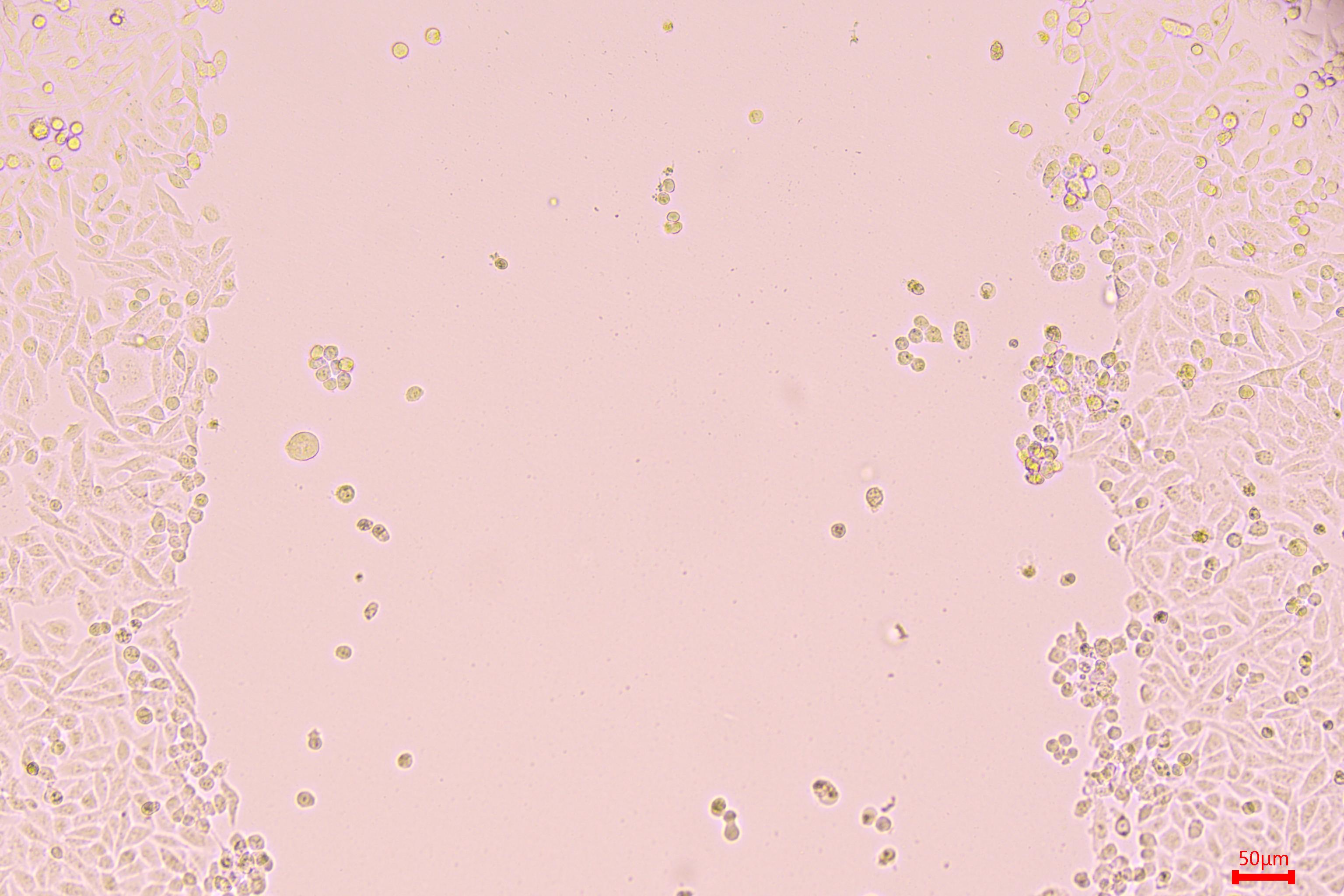

Supplement: Supplemental Information 6 [file peerj-11-14608-s006.zip › micrograph Figure3/B/A549/0h (1).jpg]

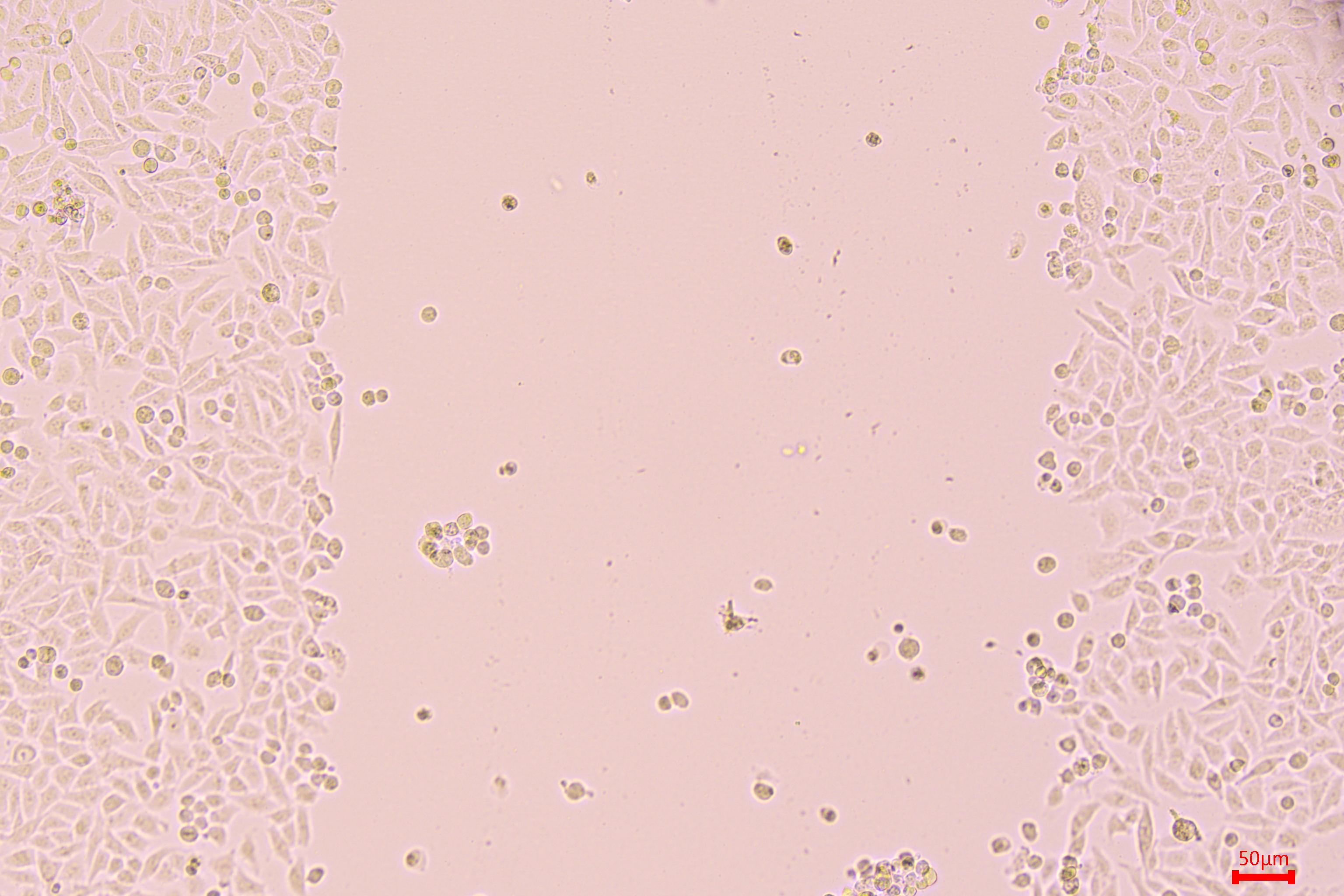

Supplement: Supplemental Information 6 [file peerj-11-14608-s006.zip › micrograph Figure3/B/A549/0h (2).jpg]

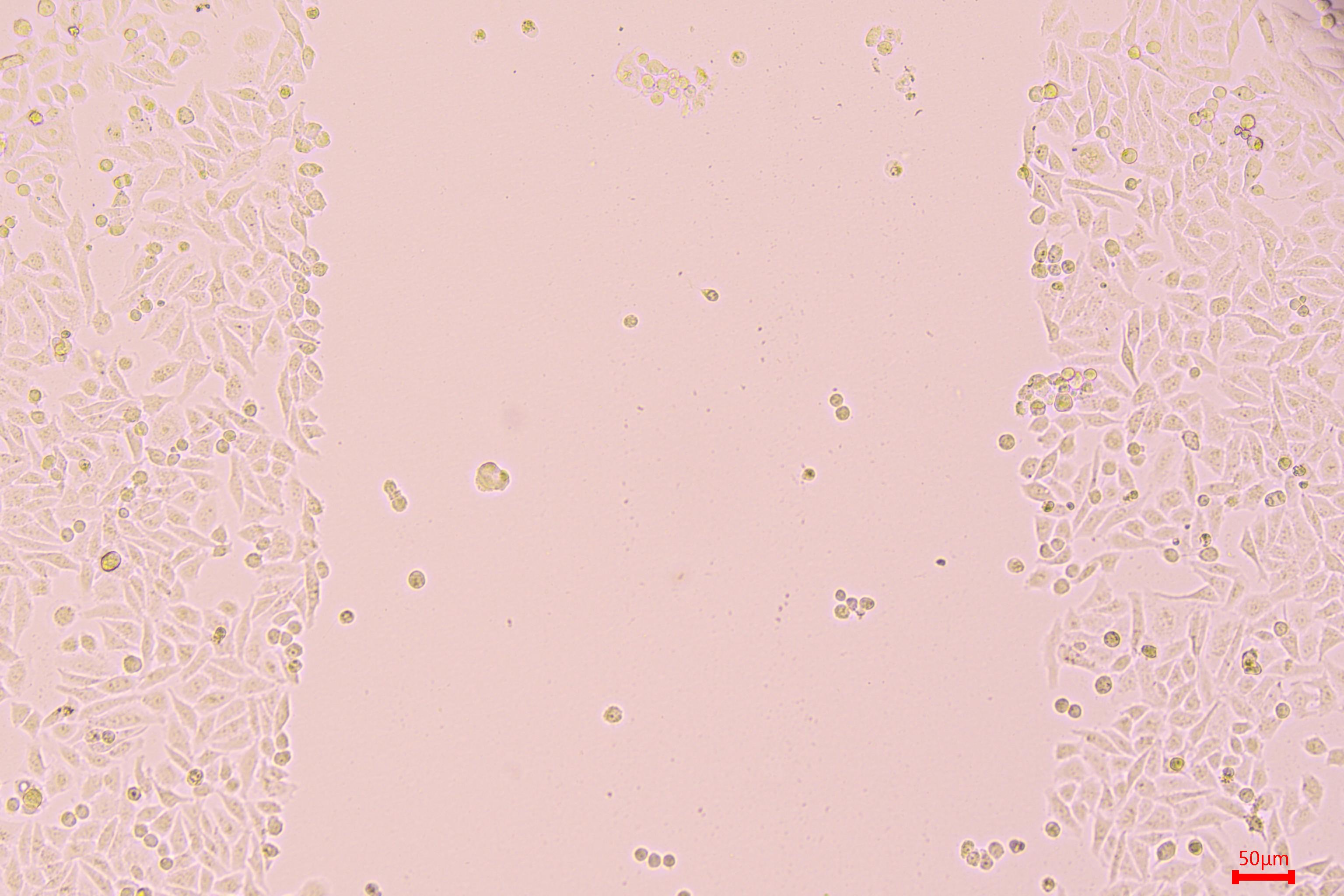

Supplement: Supplemental Information 6 [file peerj-11-14608-s006.zip › micrograph Figure3/B/A549/0h (3).jpg]

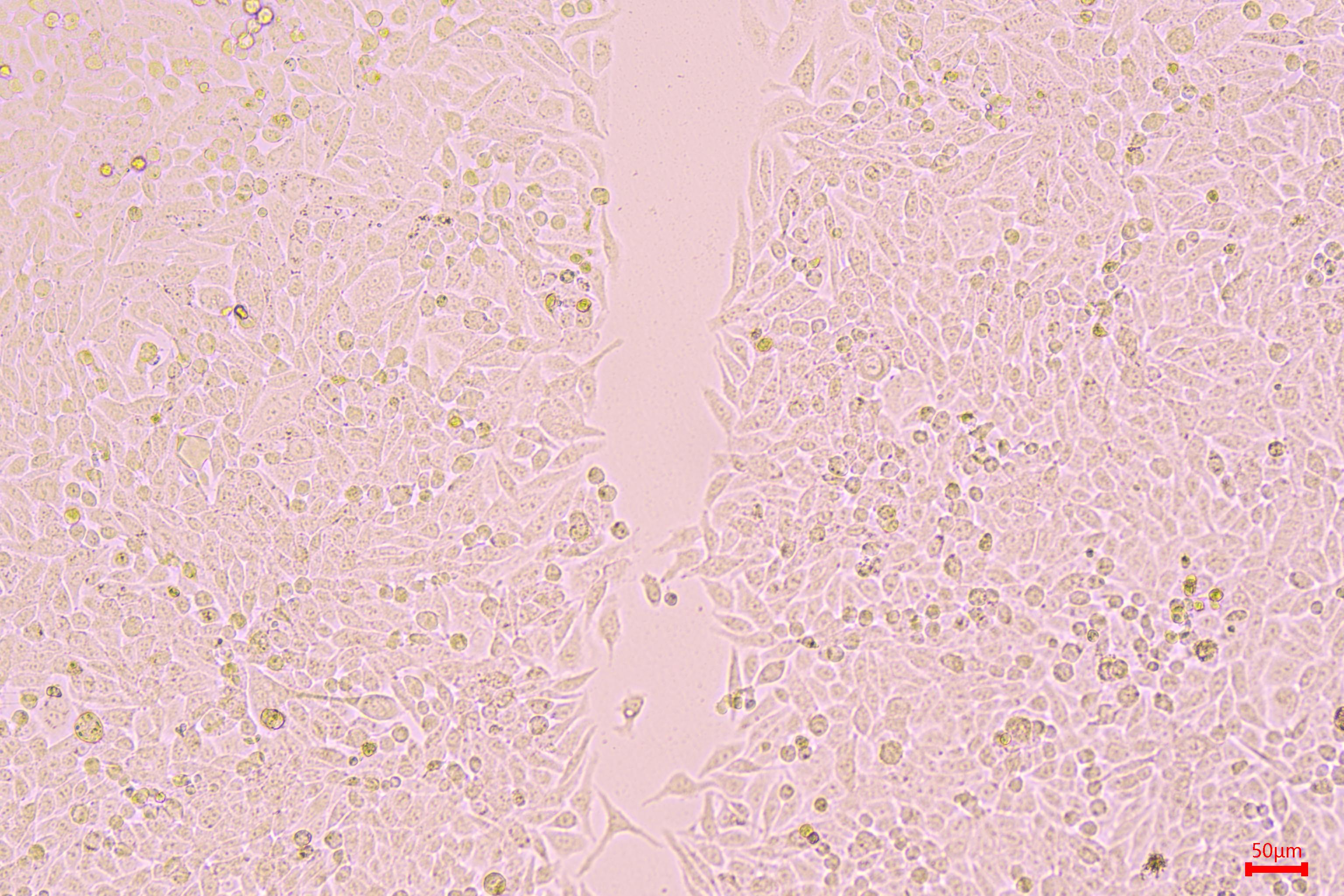

Supplement: Supplemental Information 6 [file peerj-11-14608-s006.zip › micrograph Figure3/B/A549/24h (1).jpg]

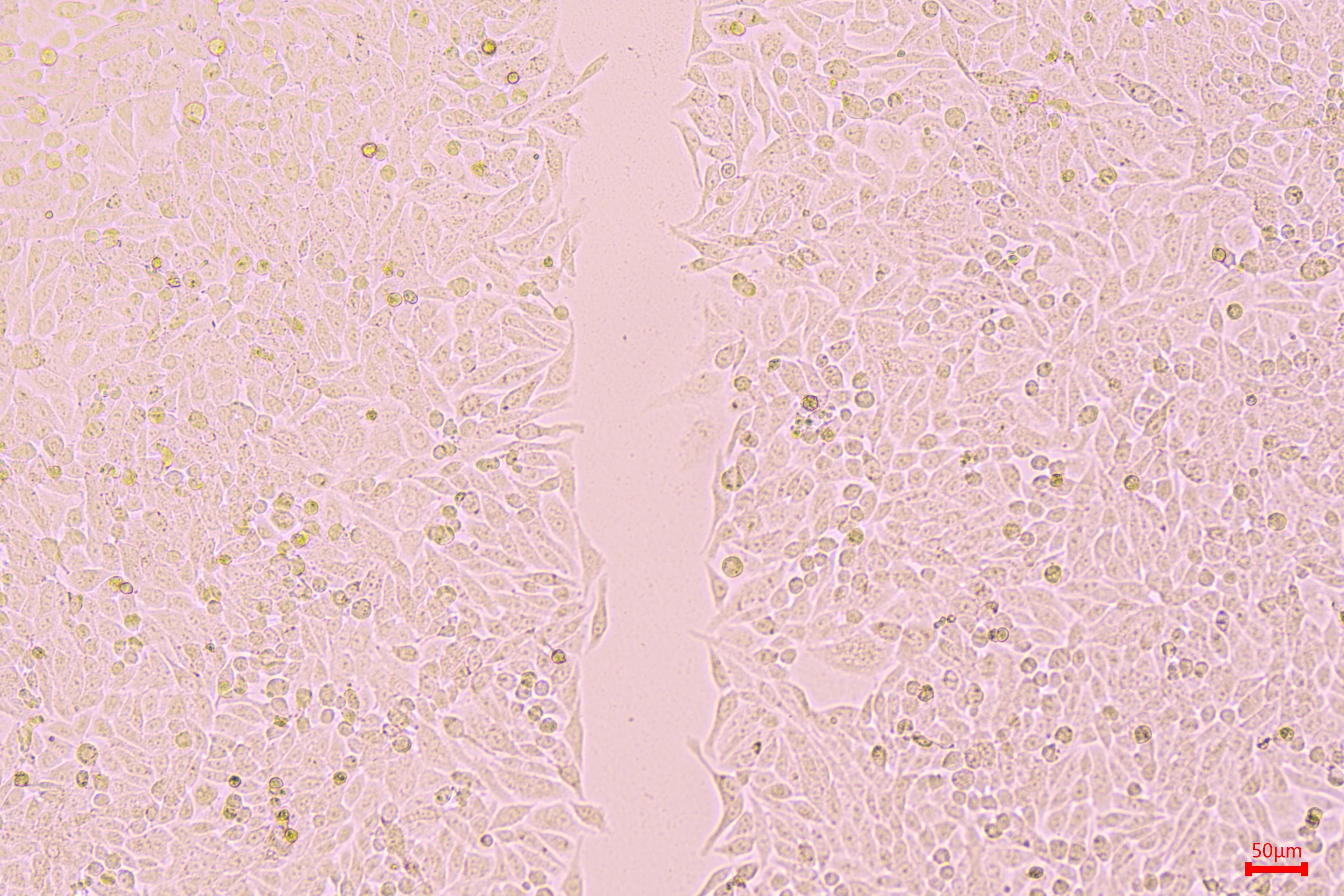

Supplement: Supplemental Information 6 [file peerj-11-14608-s006.zip › micrograph Figure3/B/A549/24h (2).jpg]

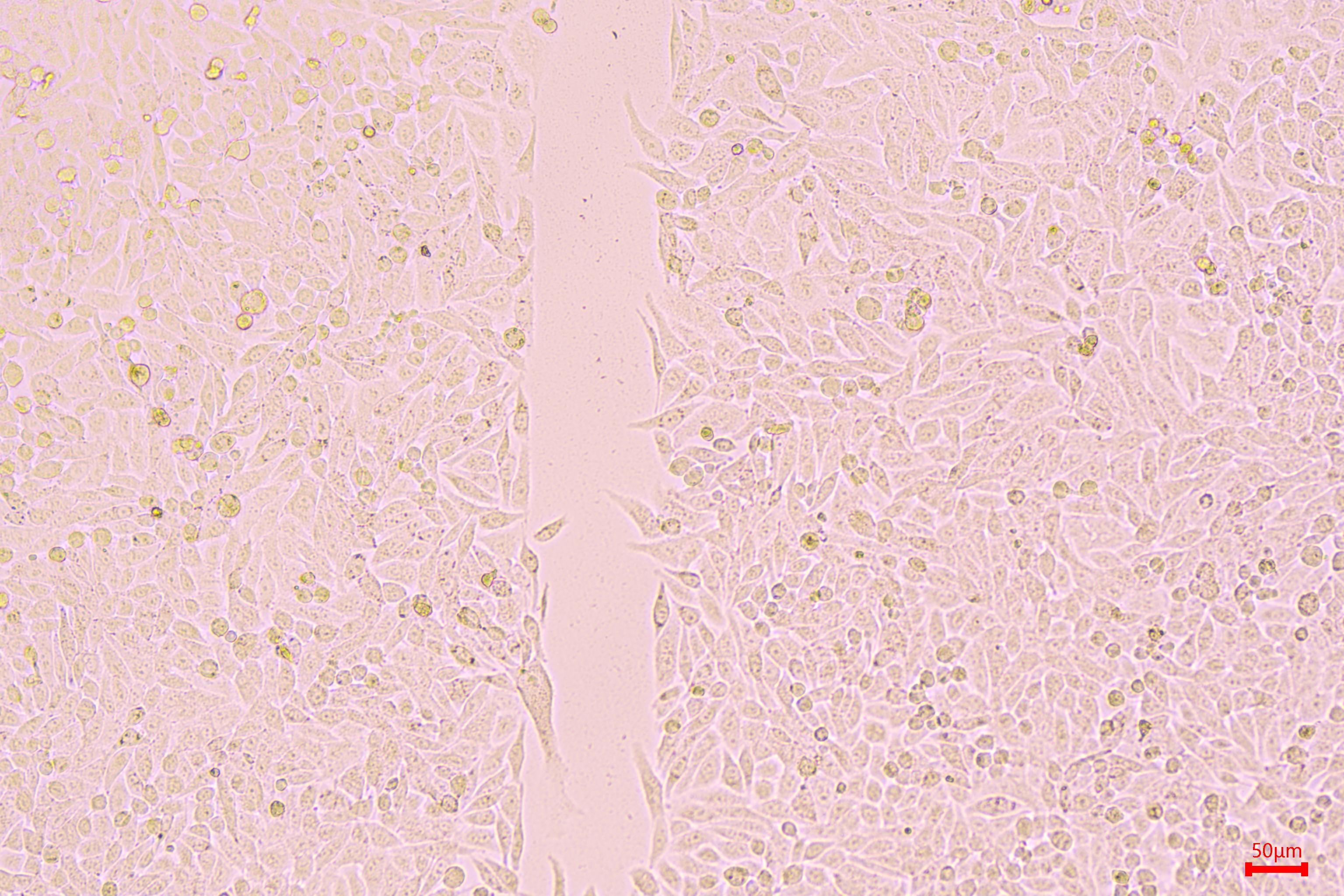

Supplement: Supplemental Information 6 [file peerj-11-14608-s006.zip › micrograph Figure3/B/A549/24h (3).jpg]

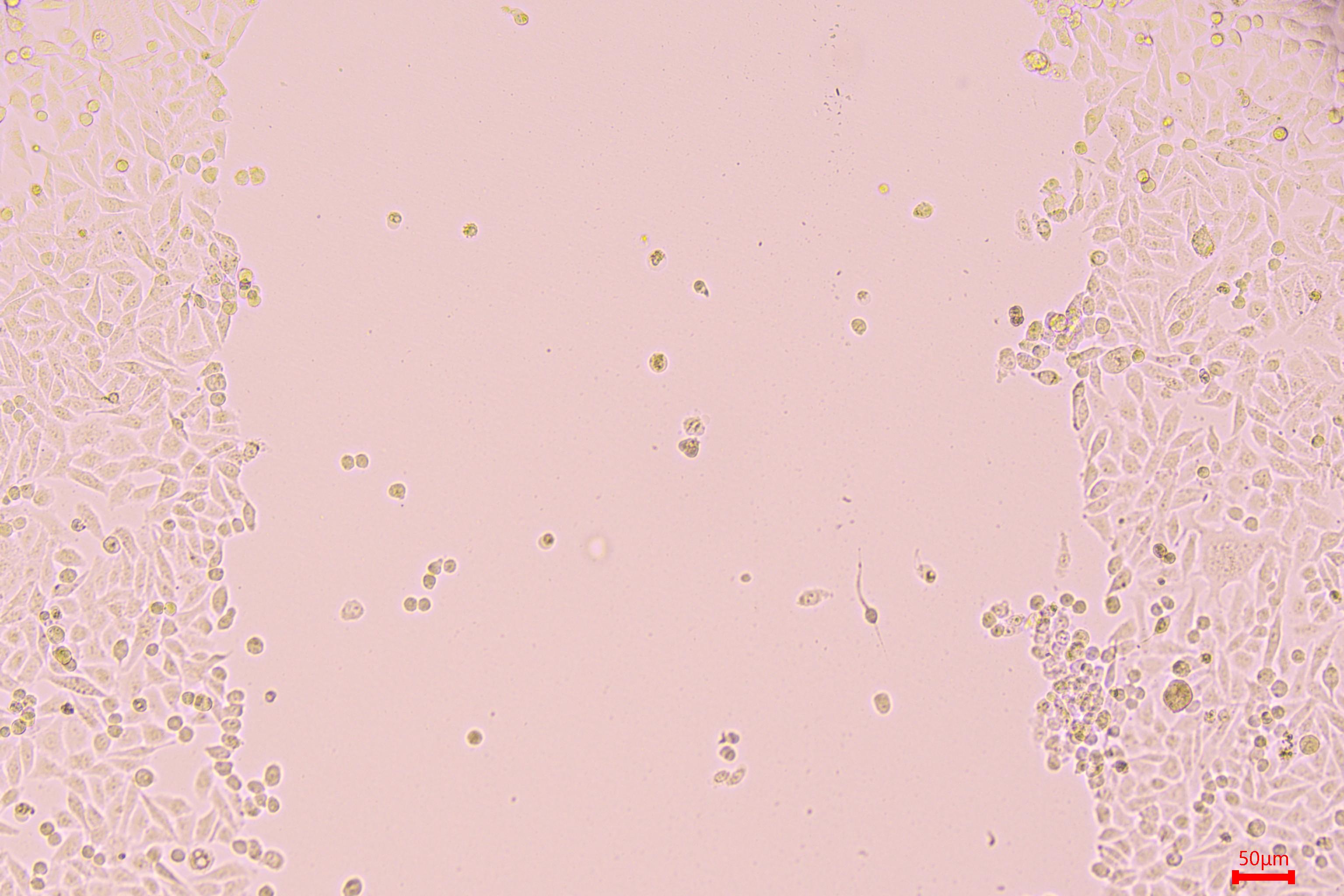

Supplement: Supplemental Information 6 [file peerj-11-14608-s006.zip › micrograph Figure3/B/A549+EXO/0h (1).jpg]

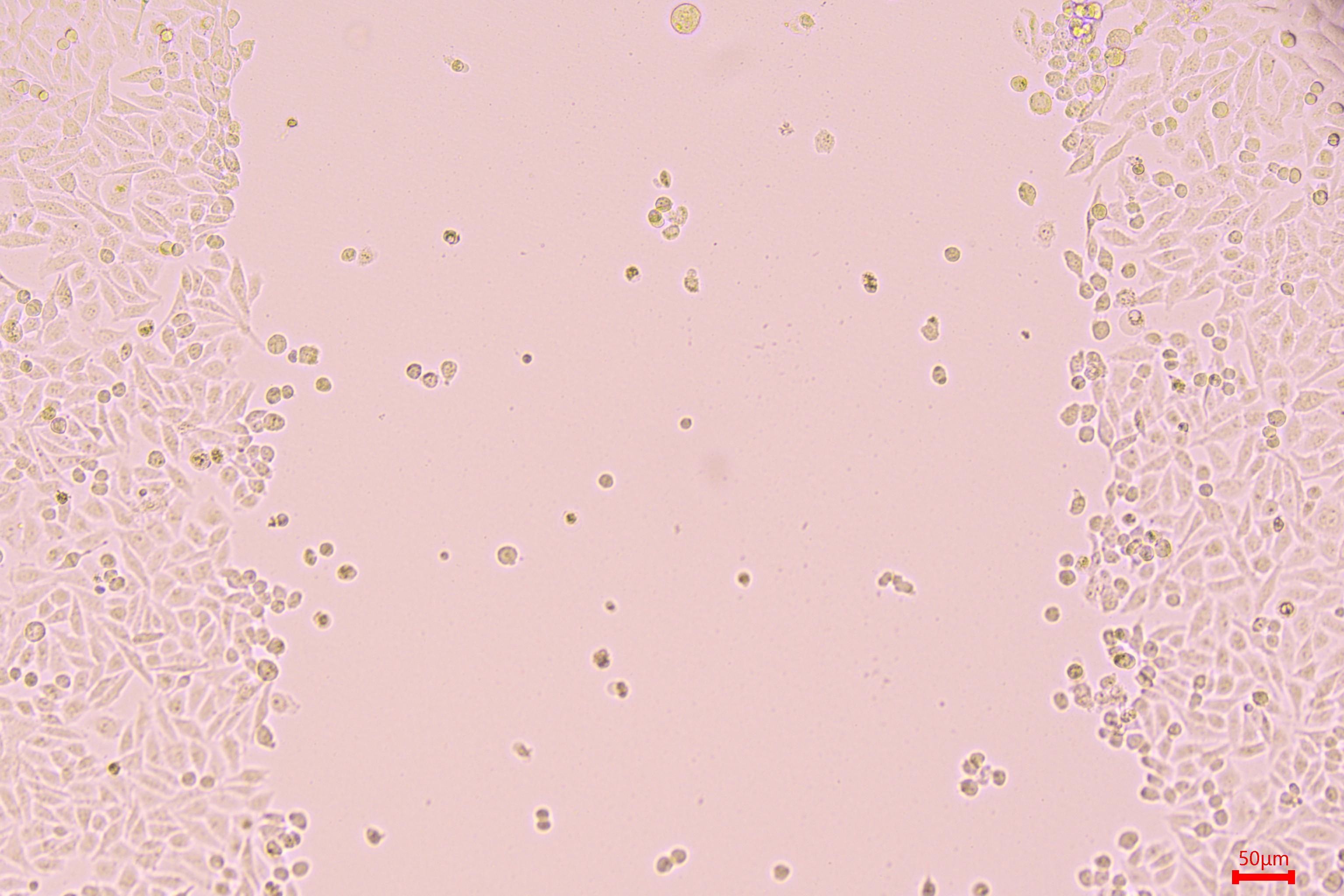

Supplement: Supplemental Information 6 [file peerj-11-14608-s006.zip › micrograph Figure3/B/A549+EXO/0h (2).jpg]

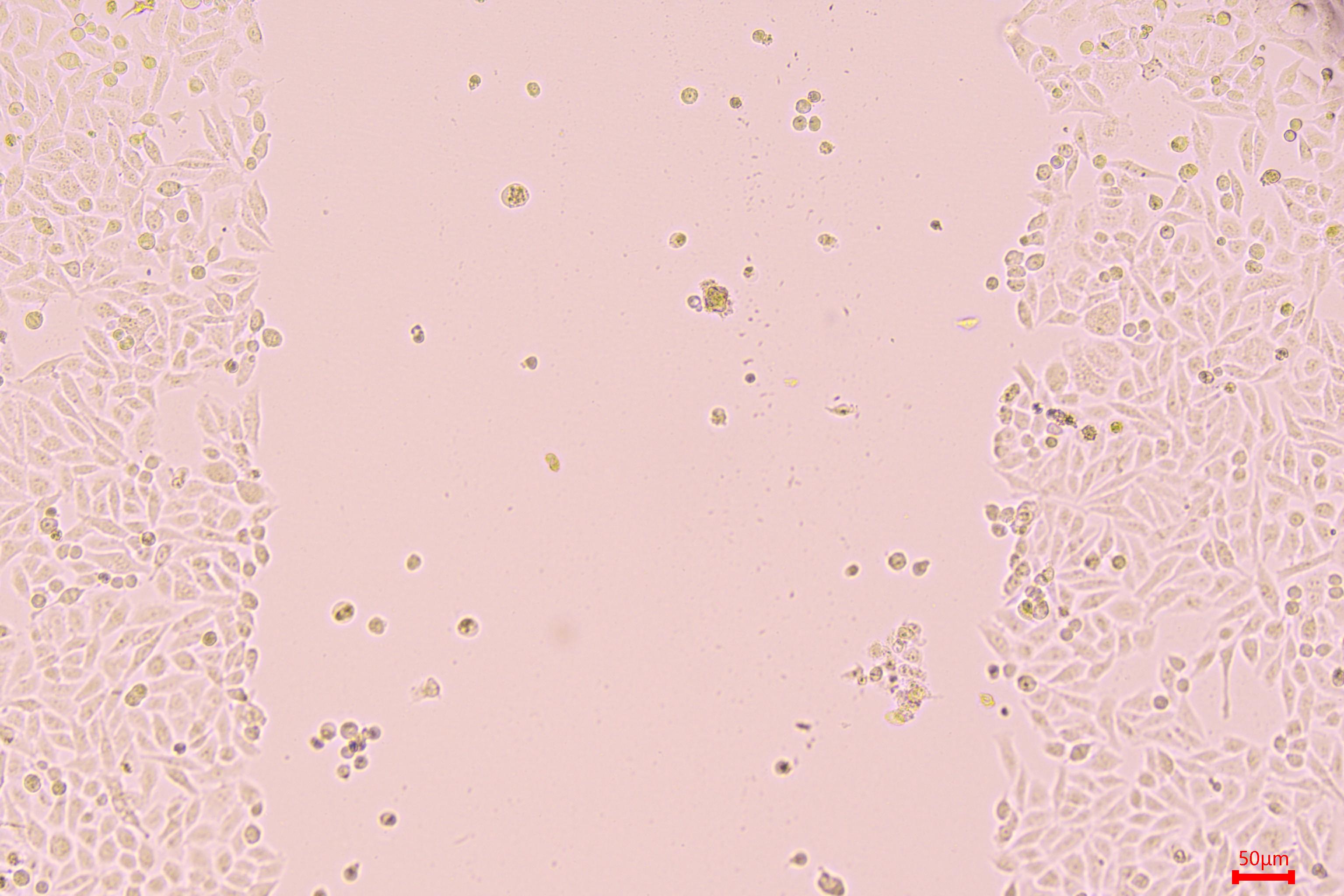

Supplement: Supplemental Information 6 [file peerj-11-14608-s006.zip › micrograph Figure3/B/A549+EXO/0h (3).jpg]

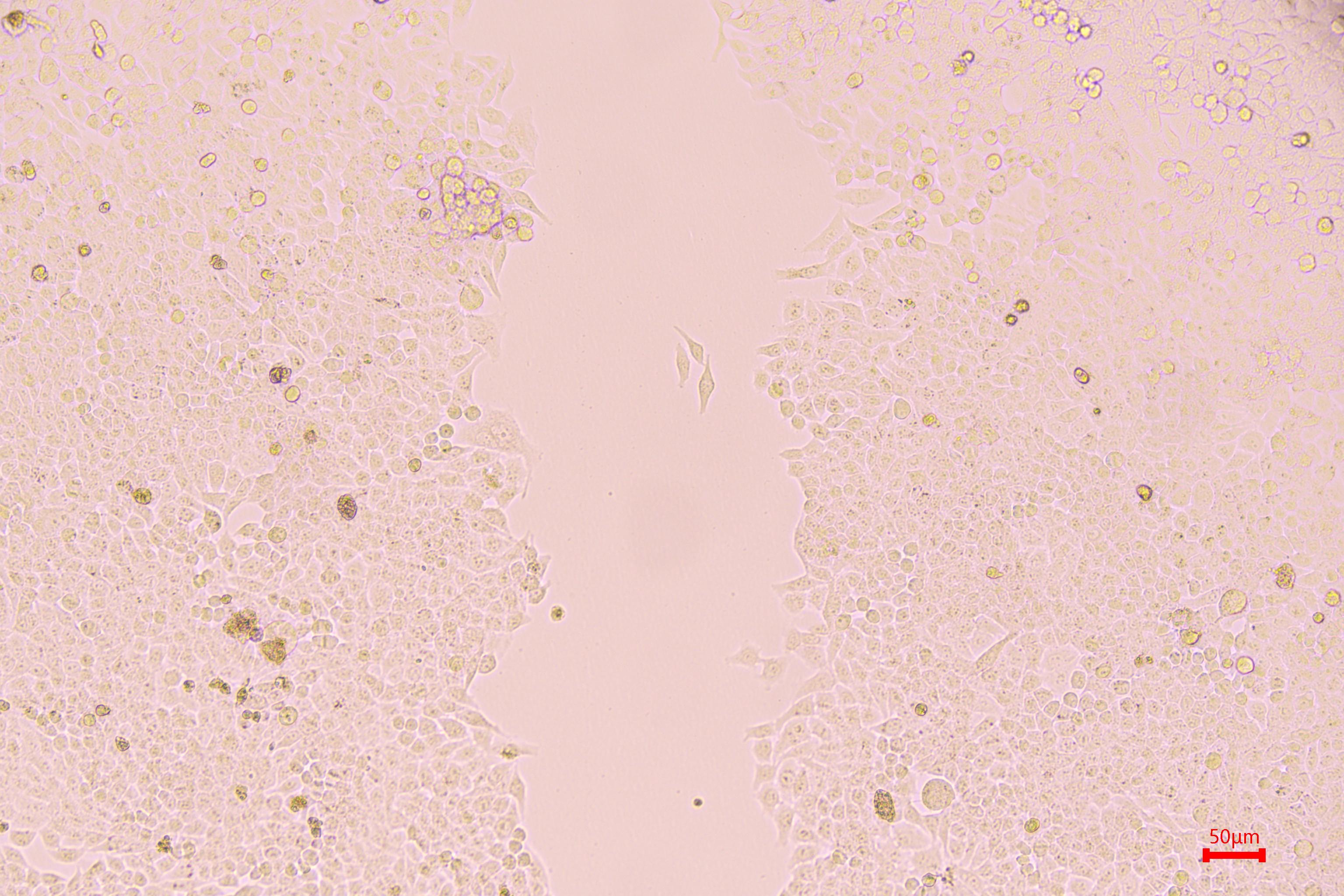

Supplement: Supplemental Information 6 [file peerj-11-14608-s006.zip › micrograph Figure3/B/A549+EXO/24h (1).jpg]

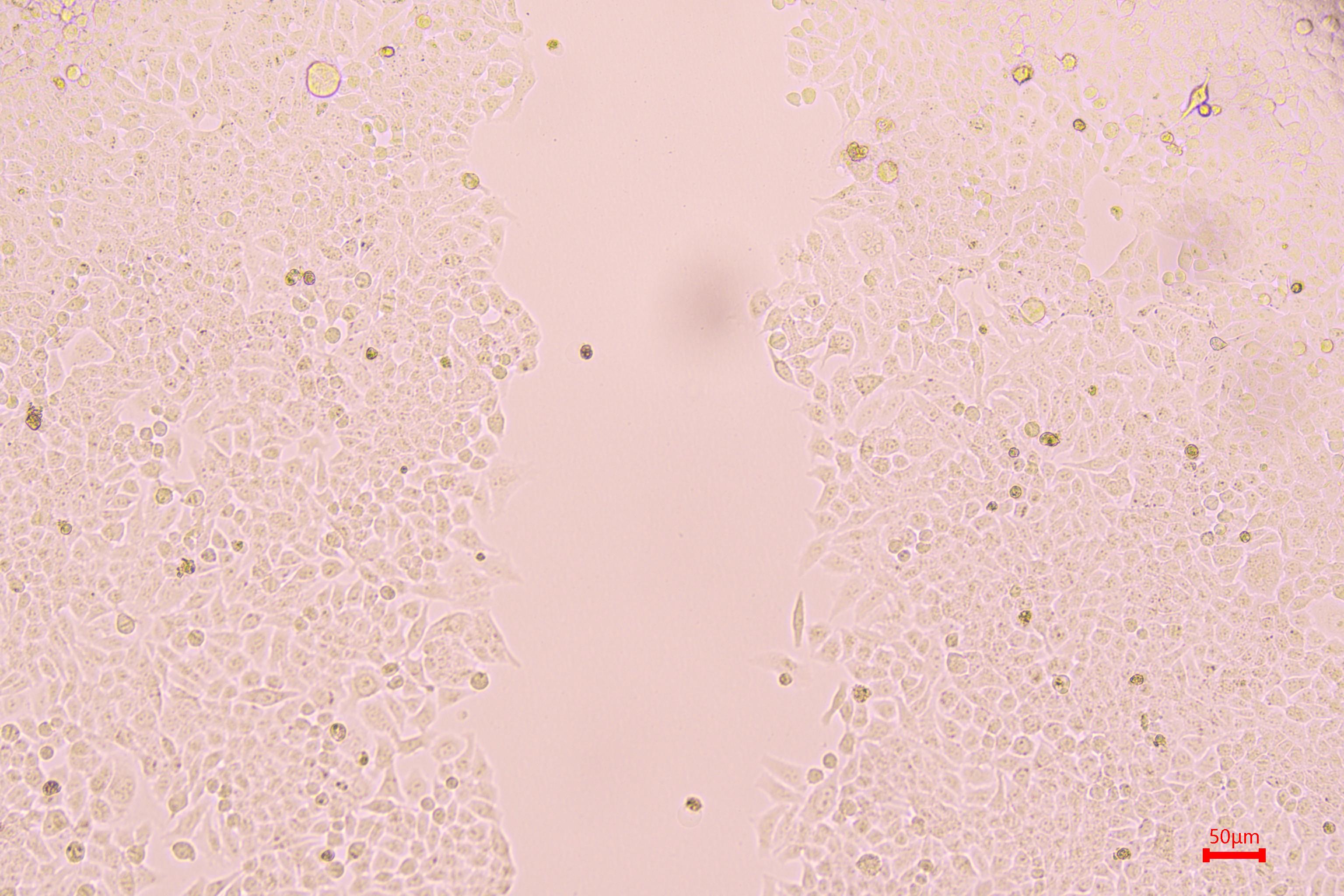

Supplement: Supplemental Information 6 [file peerj-11-14608-s006.zip › micrograph Figure3/B/A549+EXO/24h (2).jpg]

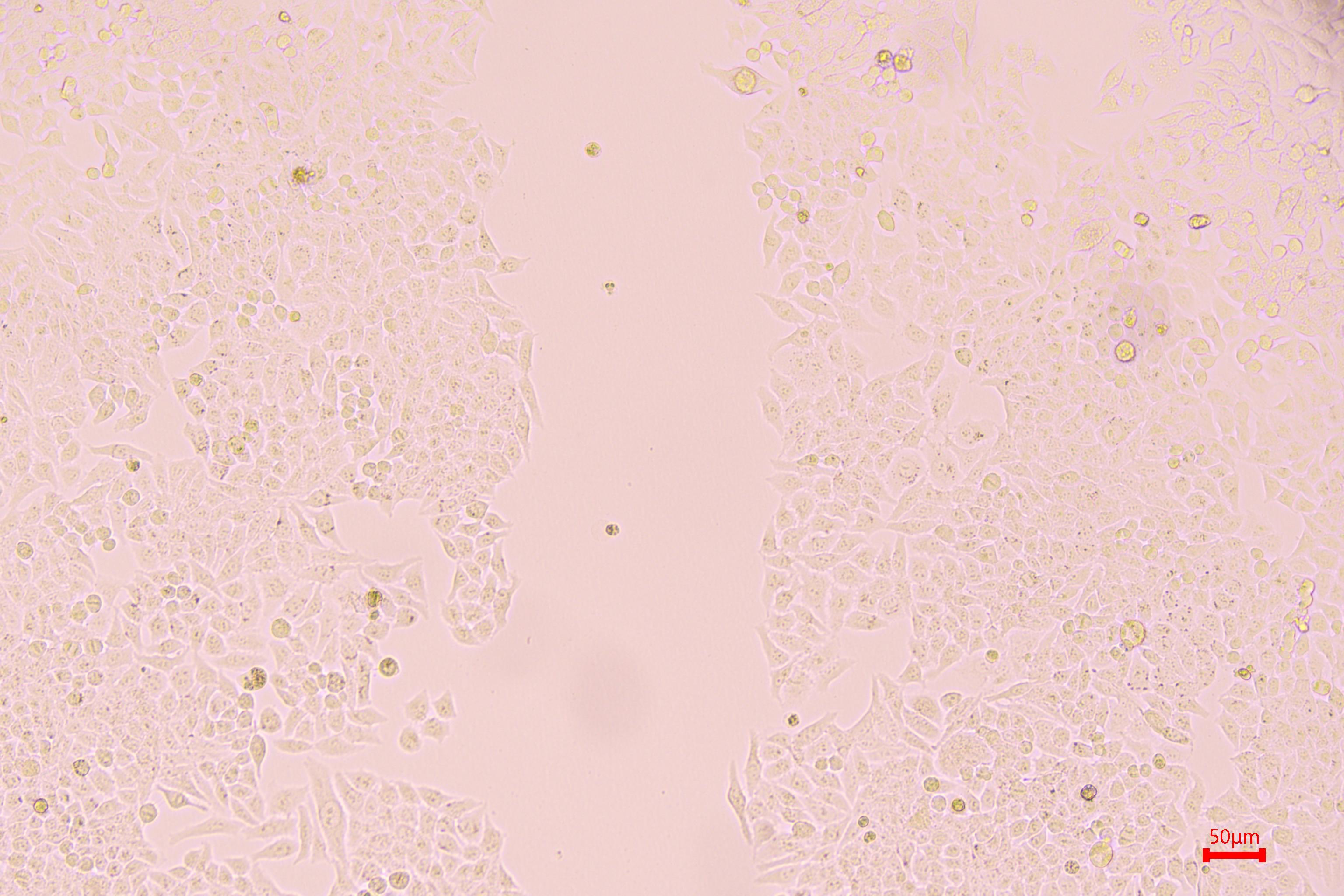

Supplement: Supplemental Information 6 [file peerj-11-14608-s006.zip › micrograph Figure3/B/A549+EXO/24h (3).jpg]

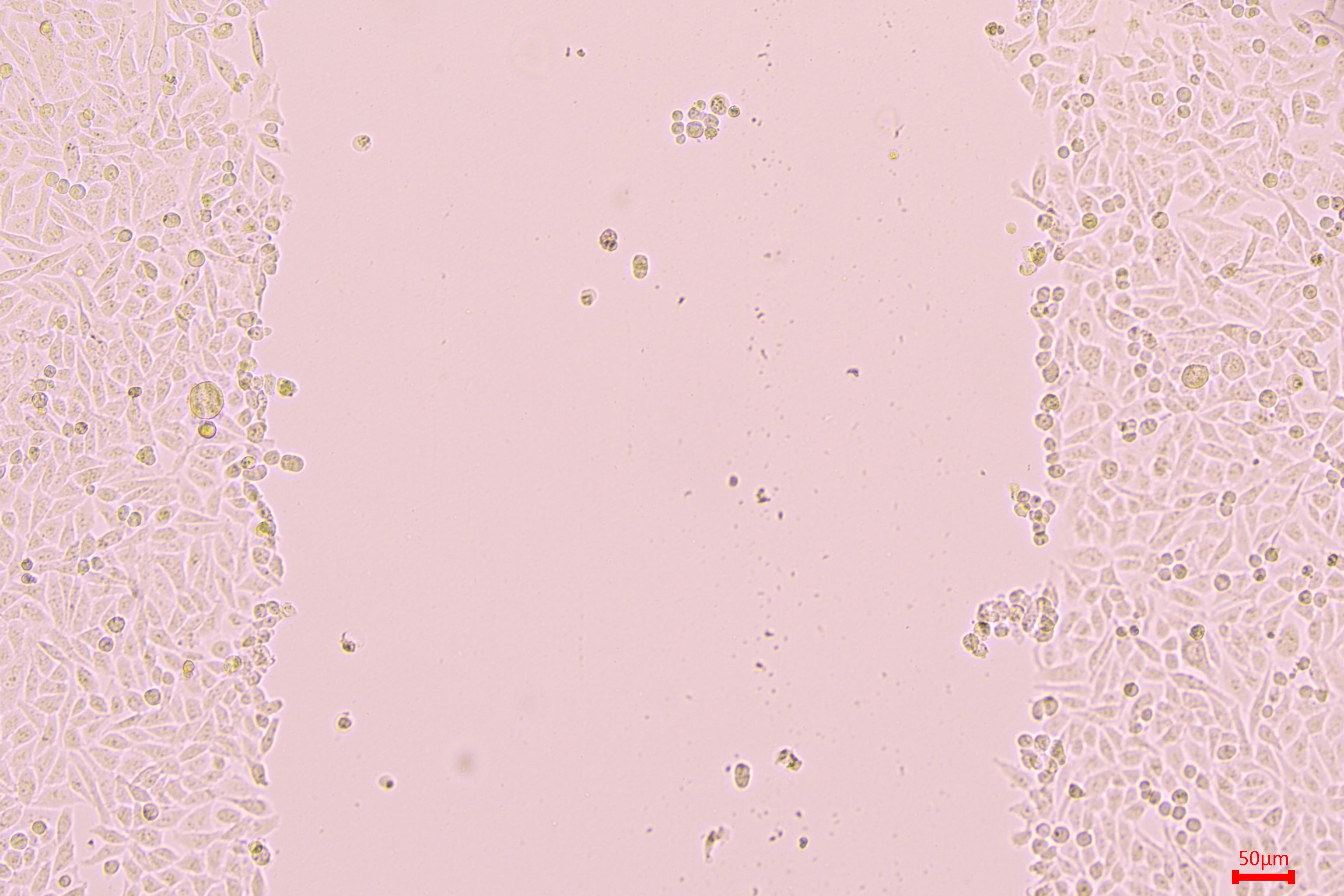

Supplement: Supplemental Information 6 [file peerj-11-14608-s006.zip › micrograph Figure3/B/HLF-A/0h (1).jpg]

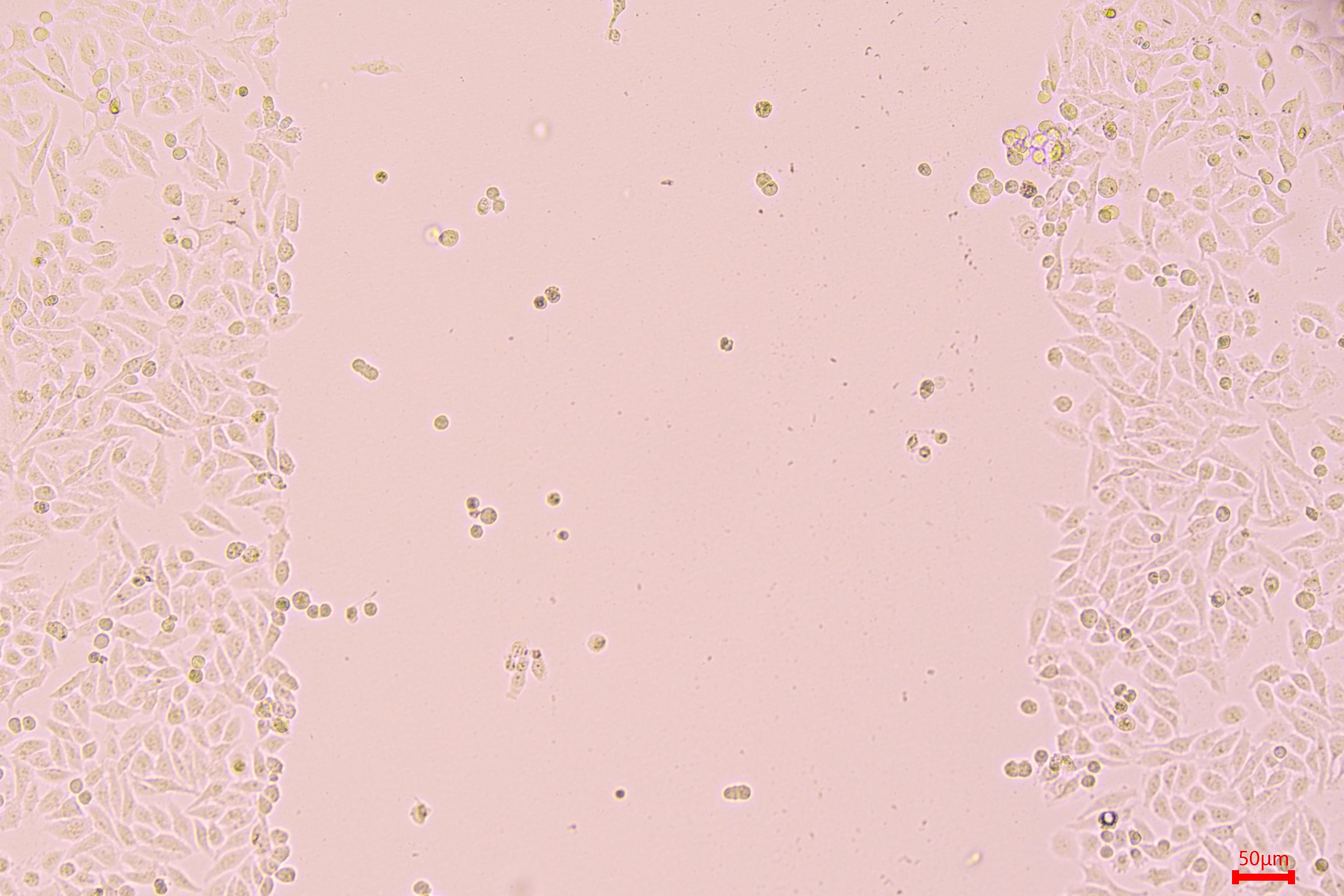

Supplement: Supplemental Information 6 [file peerj-11-14608-s006.zip › micrograph Figure3/B/HLF-A/0h (2).jpg]

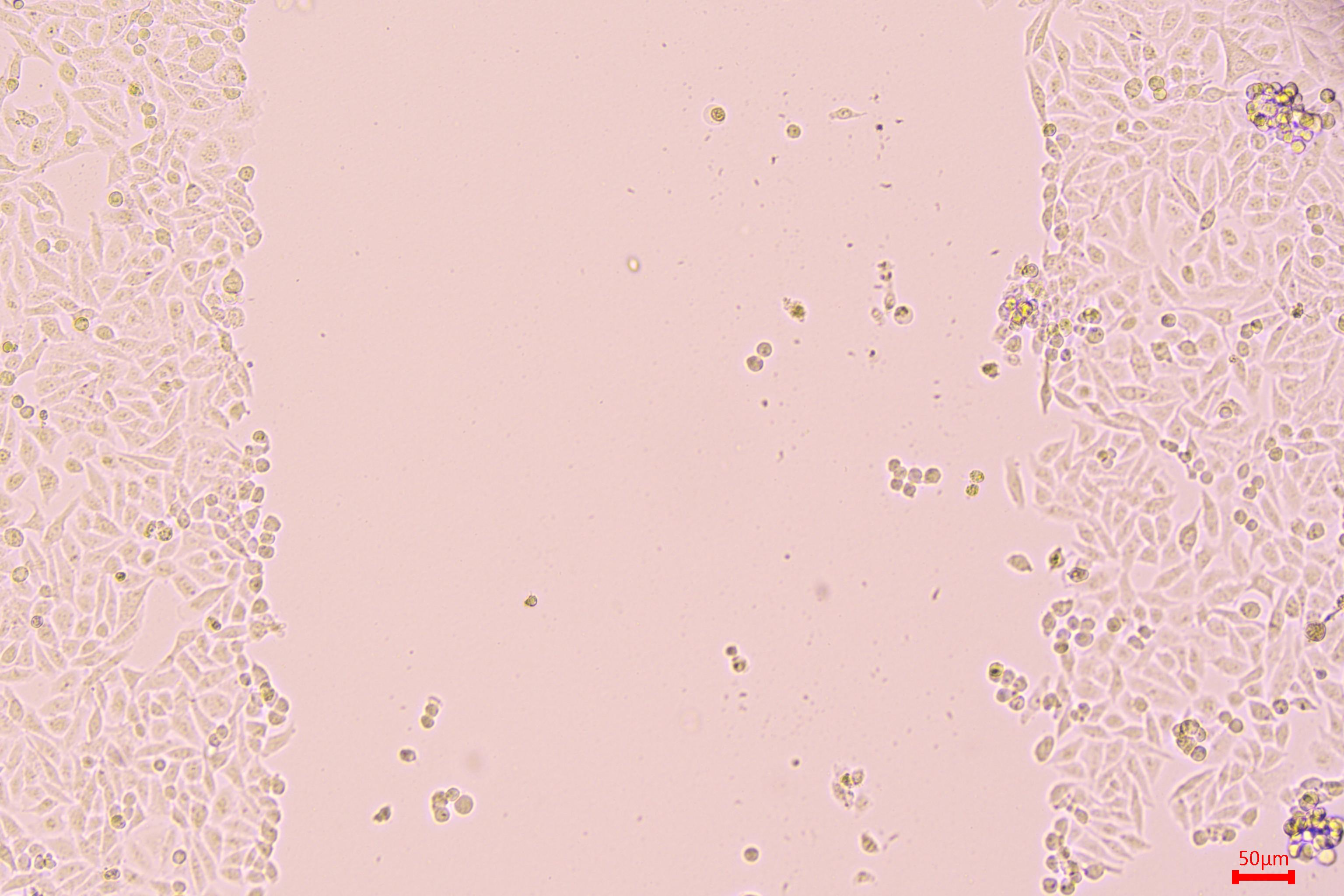

Supplement: Supplemental Information 6 [file peerj-11-14608-s006.zip › micrograph Figure3/B/HLF-A/0h (3).jpg]

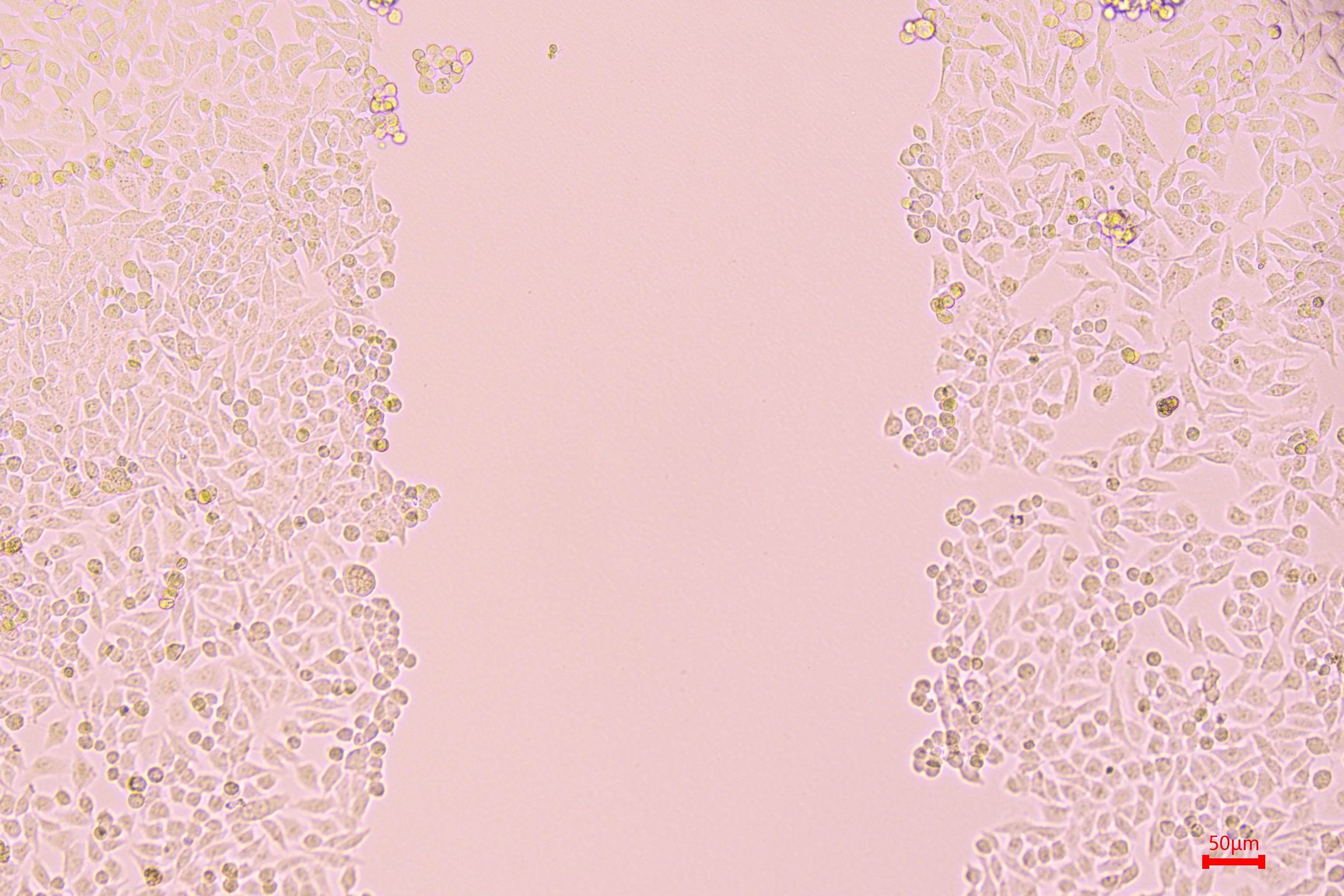

Supplement: Supplemental Information 6 [file peerj-11-14608-s006.zip › micrograph Figure3/B/HLF-A/24h (1).jpg]

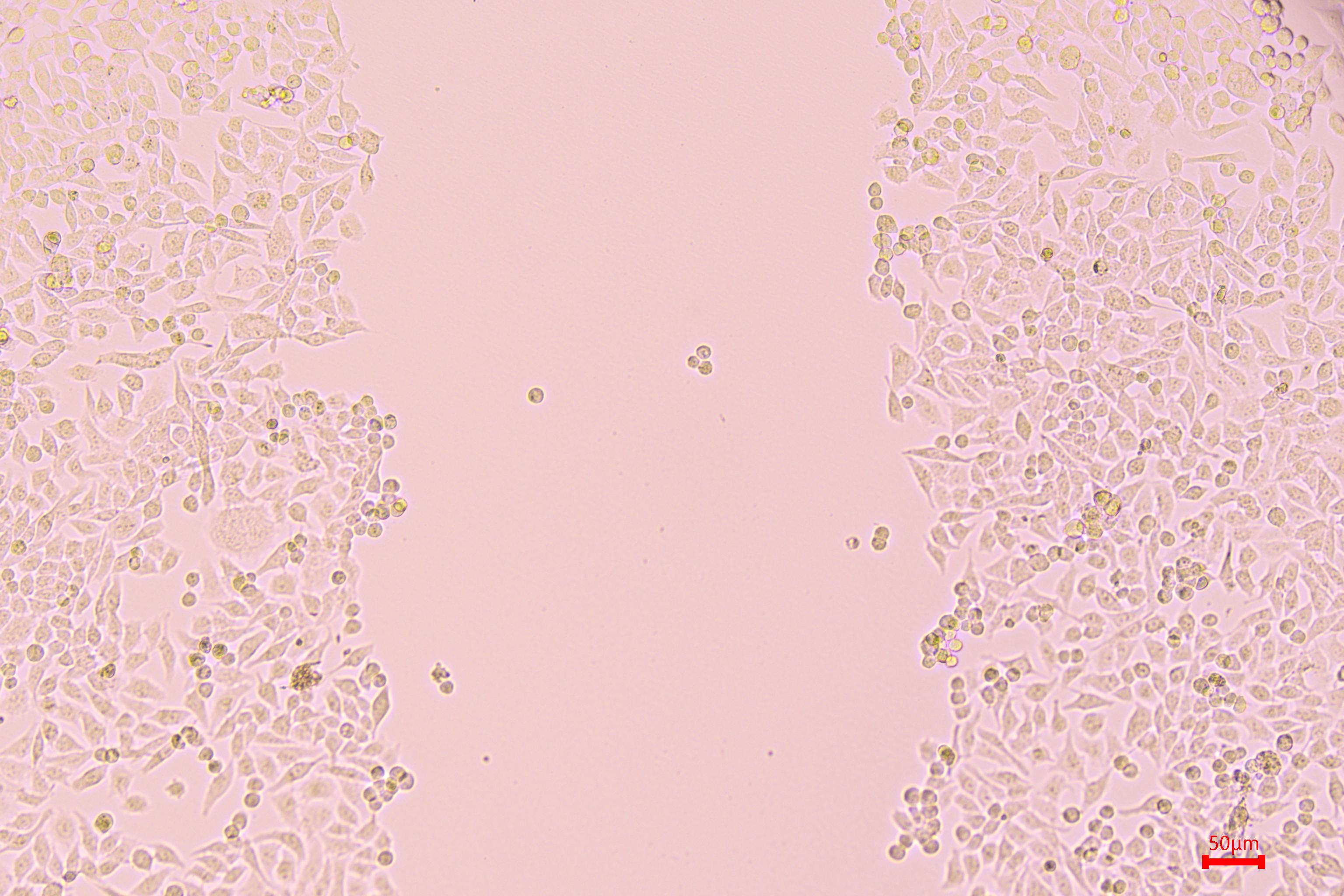

Supplement: Supplemental Information 6 [file peerj-11-14608-s006.zip › micrograph Figure3/B/HLF-A/24h (2).jpg]

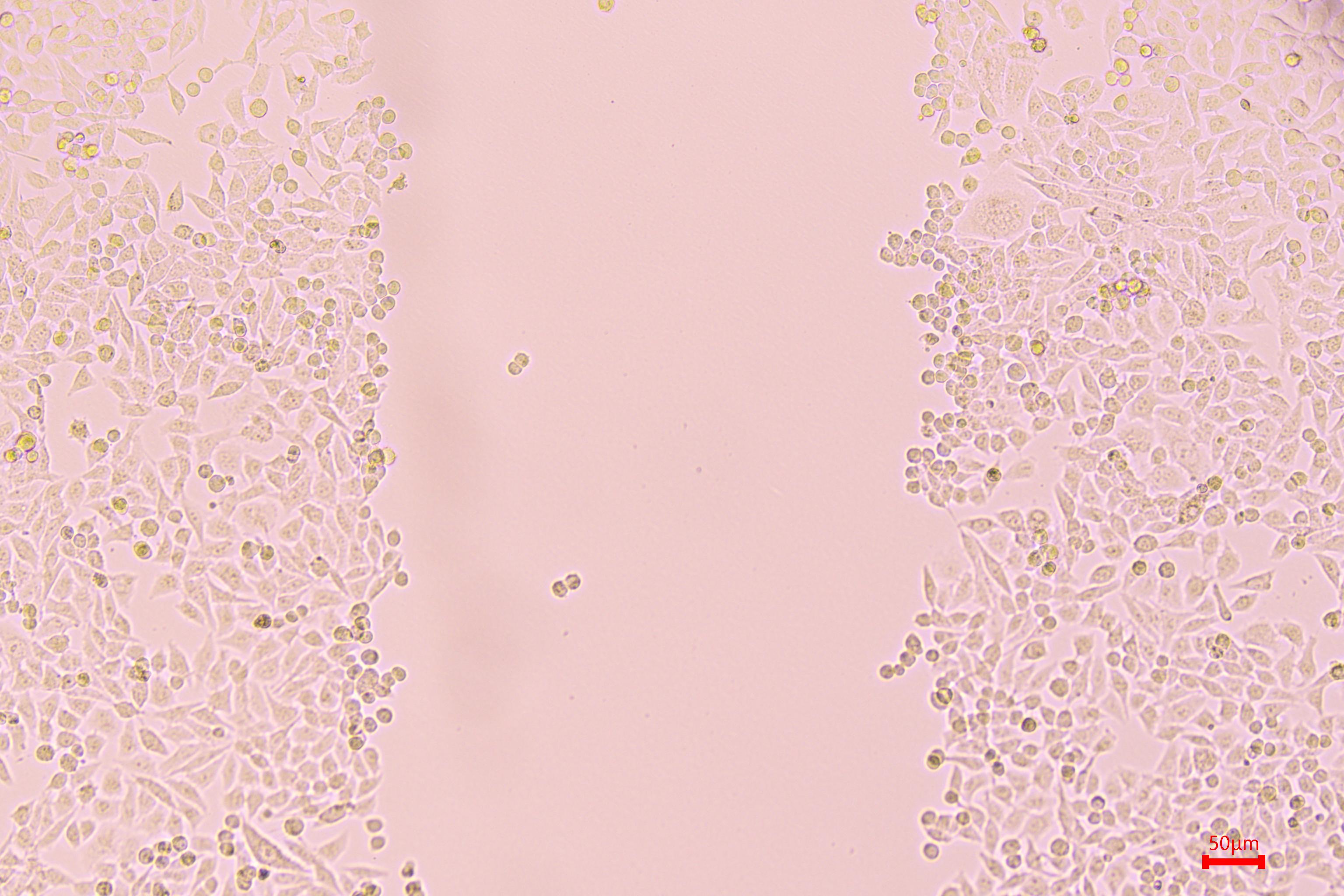

Supplement: Supplemental Information 6 [file peerj-11-14608-s006.zip › micrograph Figure3/B/HLF-A/24h (3).jpg]
