# Supplementary figures and images for: Exosomes derived from M1 macrophages inhibit the proliferation of the A549 and H1299 lung cancer cell lines via the miRNA-let-7b-5p-GNG5 axis (part 3 of 4)
Source: PeerJ. 2023 Jan 9;11:e14608. doi: 10.7717/peerj.14608 (PMC9835688; doi:10.7717/peerj.14608)

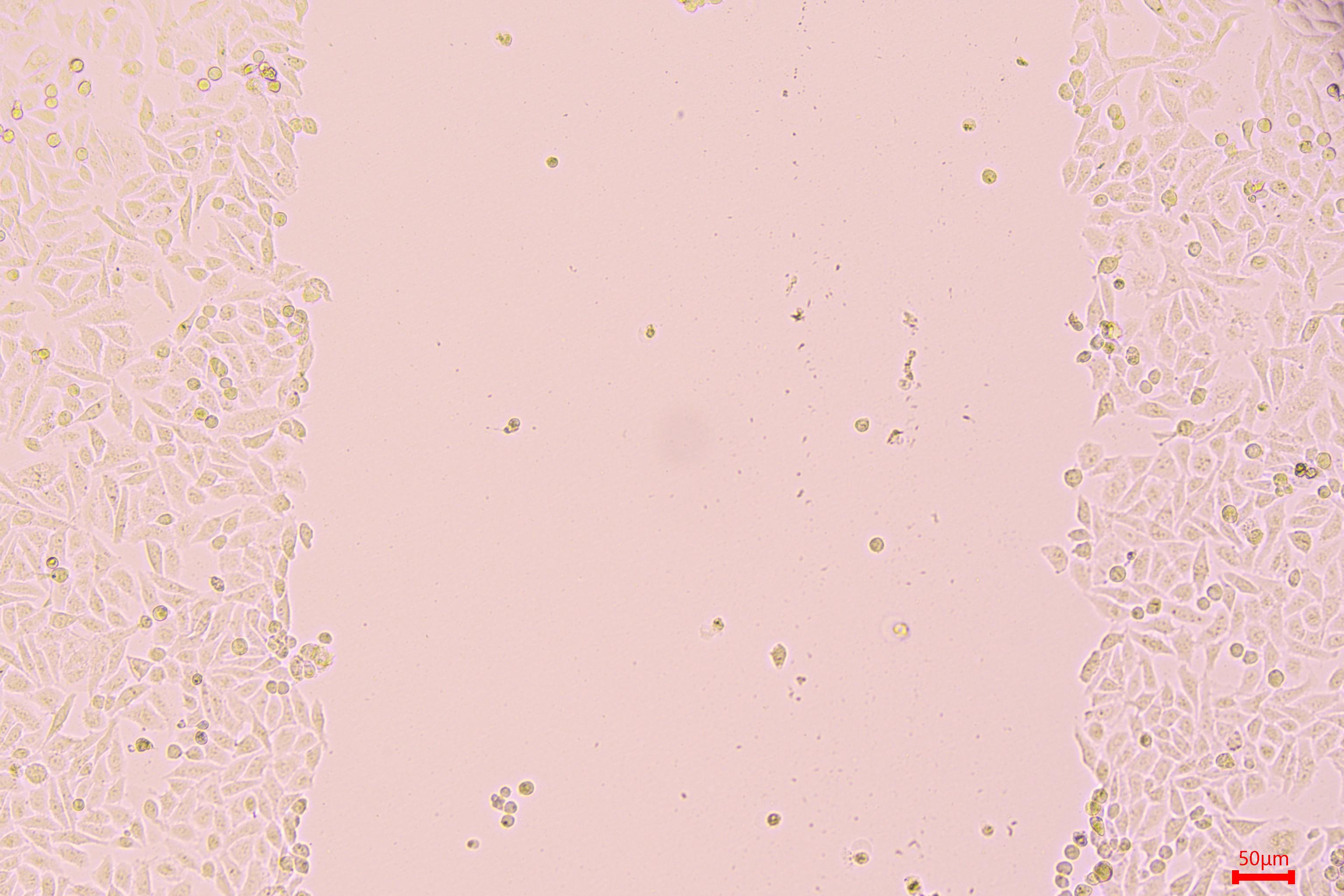

Supplement: Supplemental Information 6 [file peerj-11-14608-s006.zip › micrograph Figure3/B/HLF-A+EXO/0h (1).jpg]

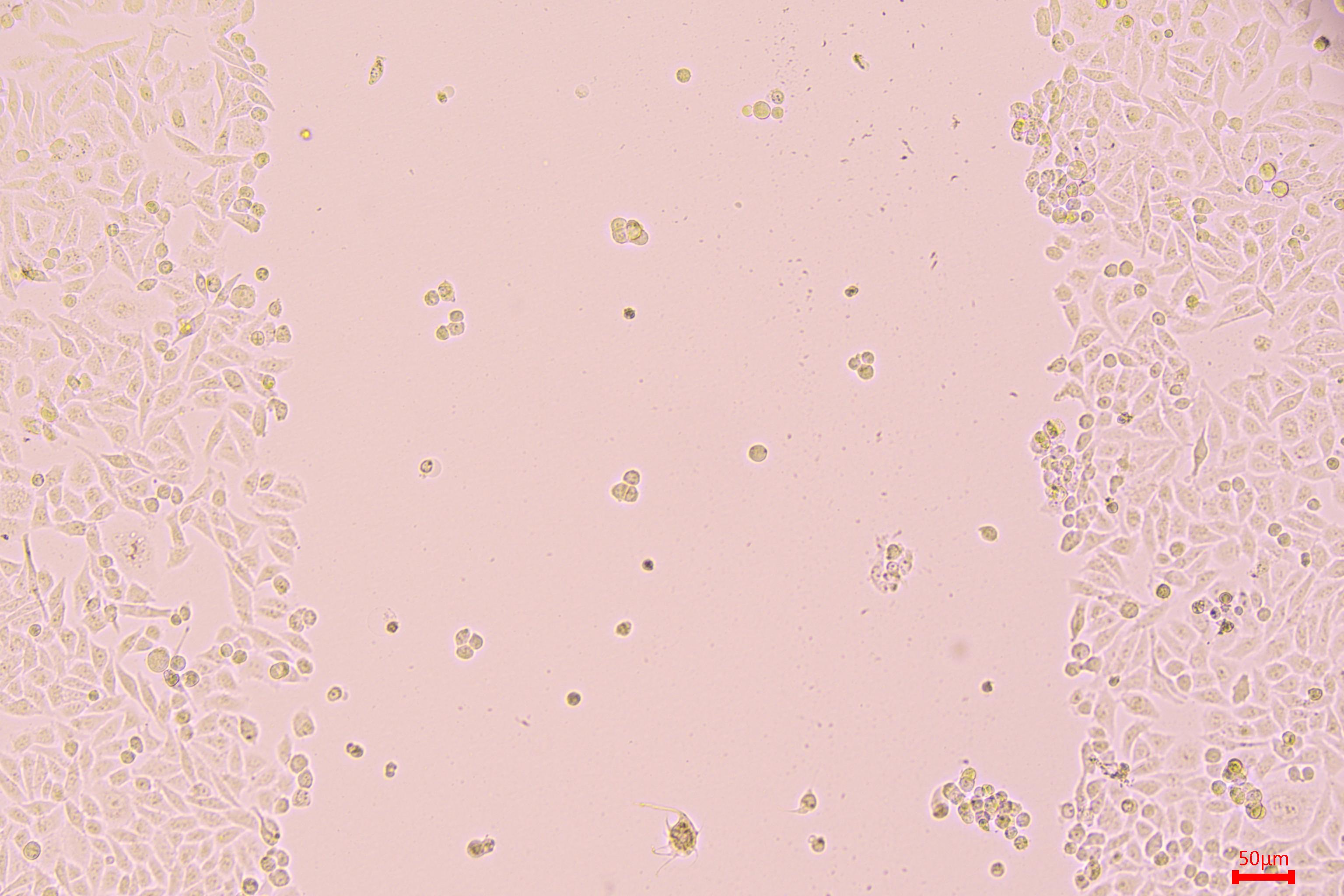

Supplement: Supplemental Information 6 [file peerj-11-14608-s006.zip › micrograph Figure3/B/HLF-A+EXO/0h (2).jpg]

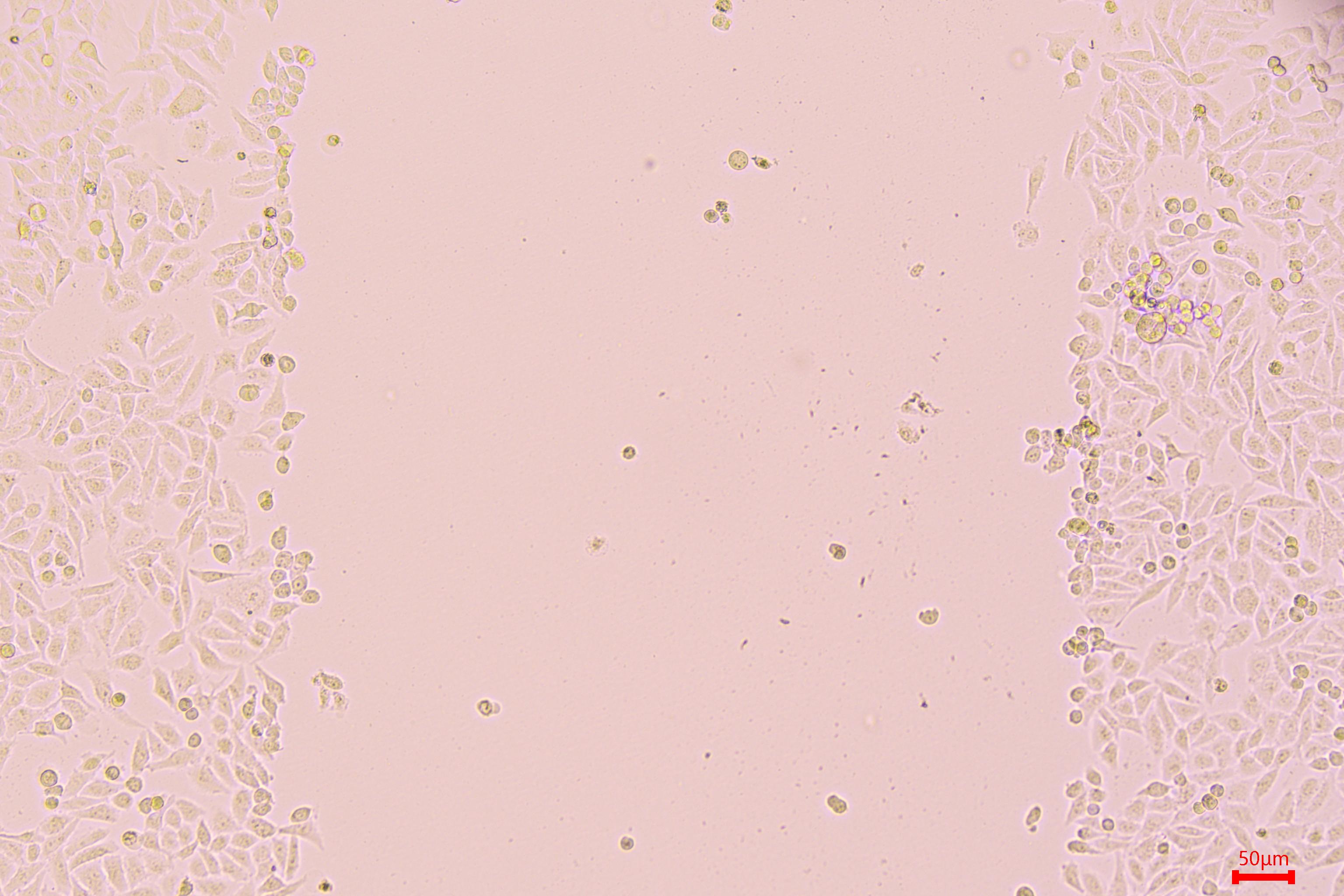

Supplement: Supplemental Information 6 [file peerj-11-14608-s006.zip › micrograph Figure3/B/HLF-A+EXO/0h (3).jpg]

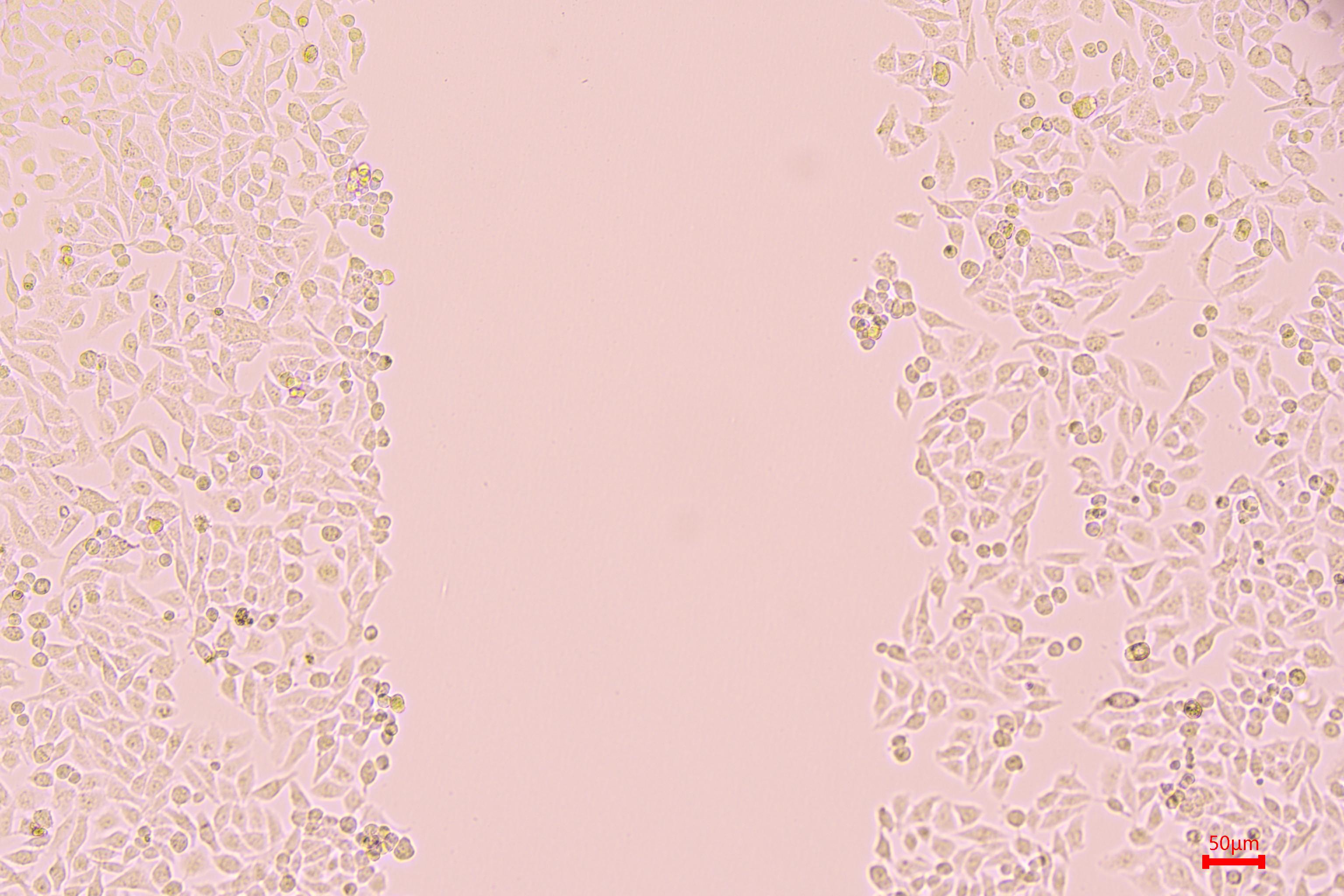

Supplement: Supplemental Information 6 [file peerj-11-14608-s006.zip › micrograph Figure3/B/HLF-A+EXO/24h (1).jpg]

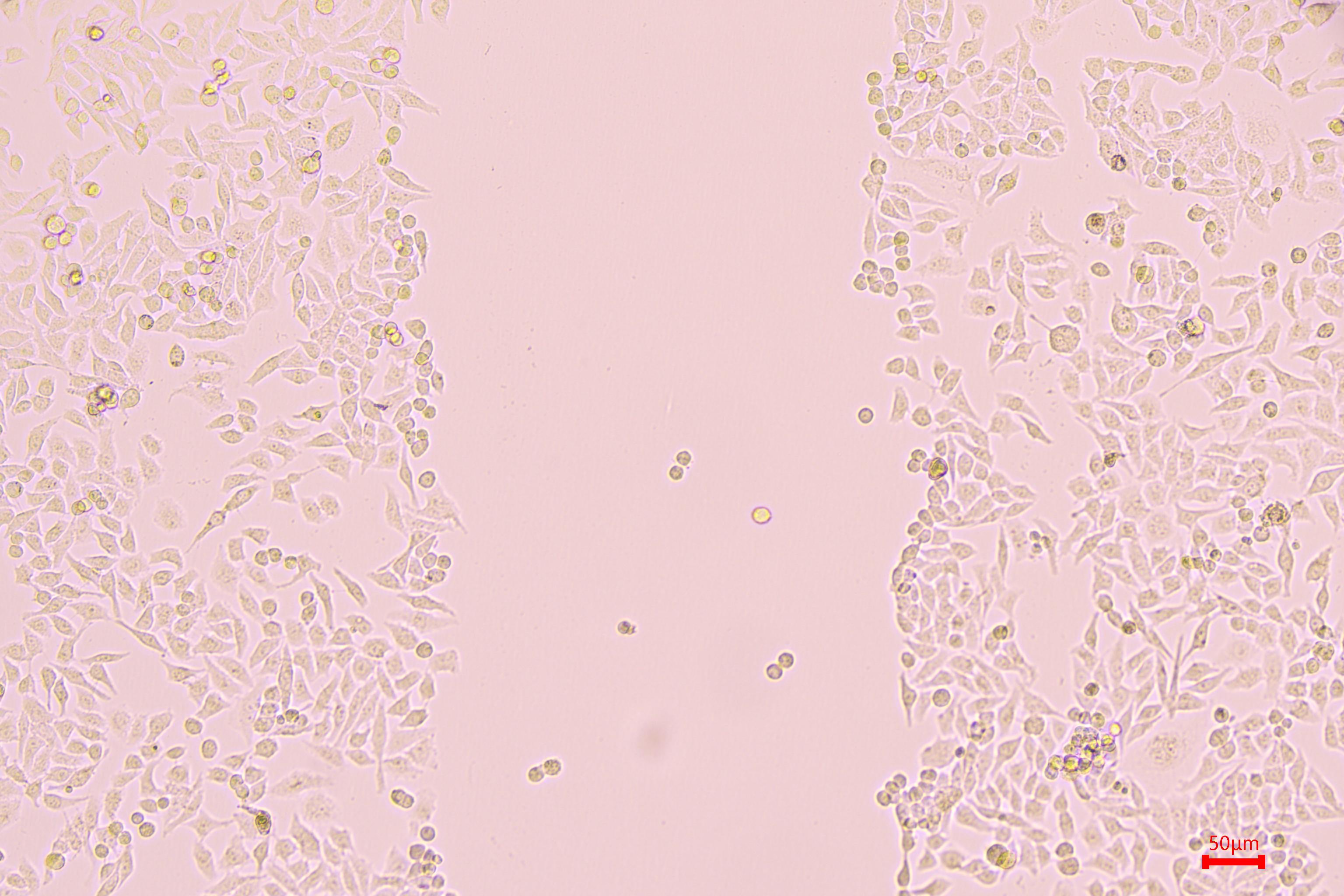

Supplement: Supplemental Information 6 [file peerj-11-14608-s006.zip › micrograph Figure3/B/HLF-A+EXO/24h (2).jpg]

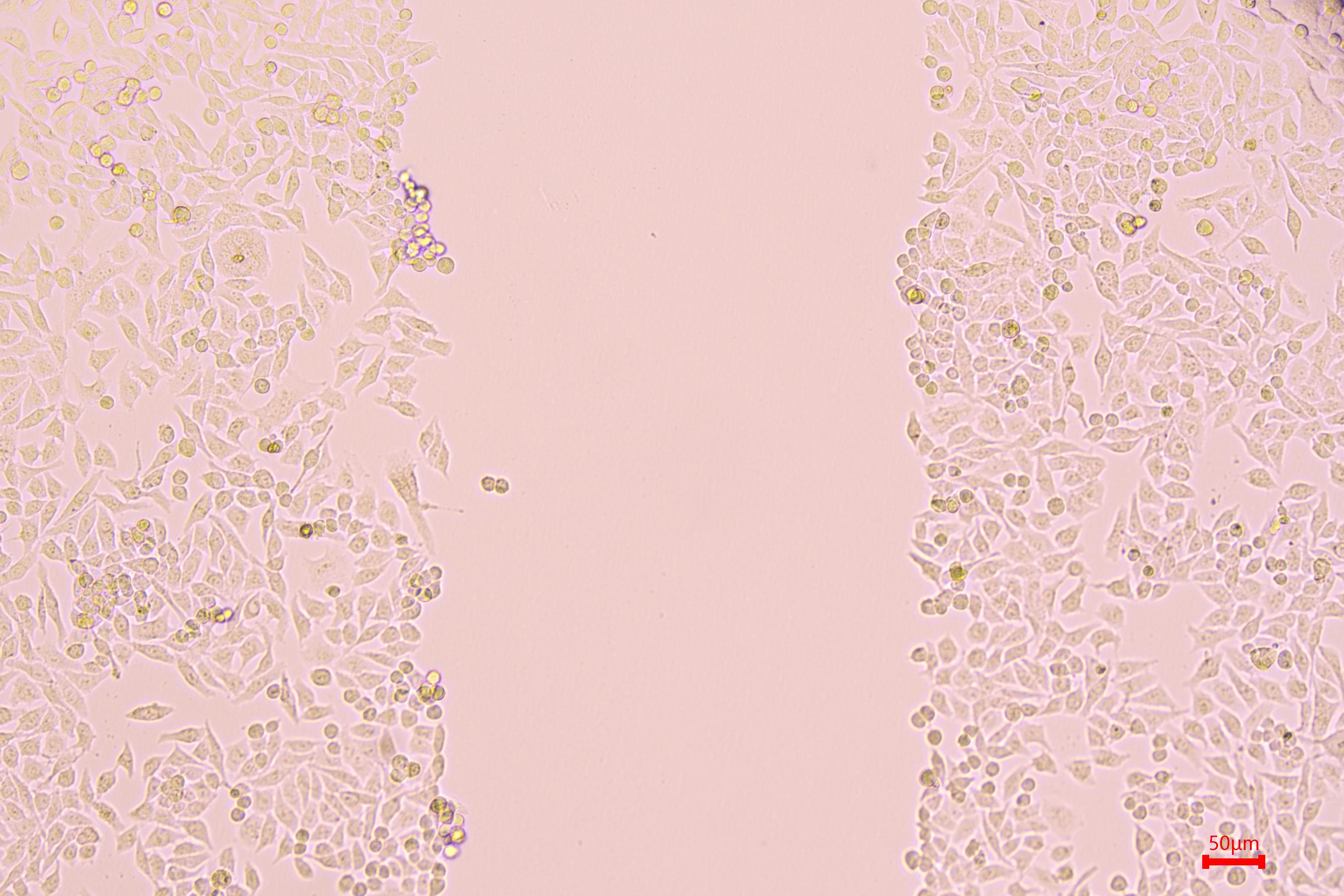

Supplement: Supplemental Information 6 [file peerj-11-14608-s006.zip › micrograph Figure3/B/HLF-A+EXO/24h (3).jpg]

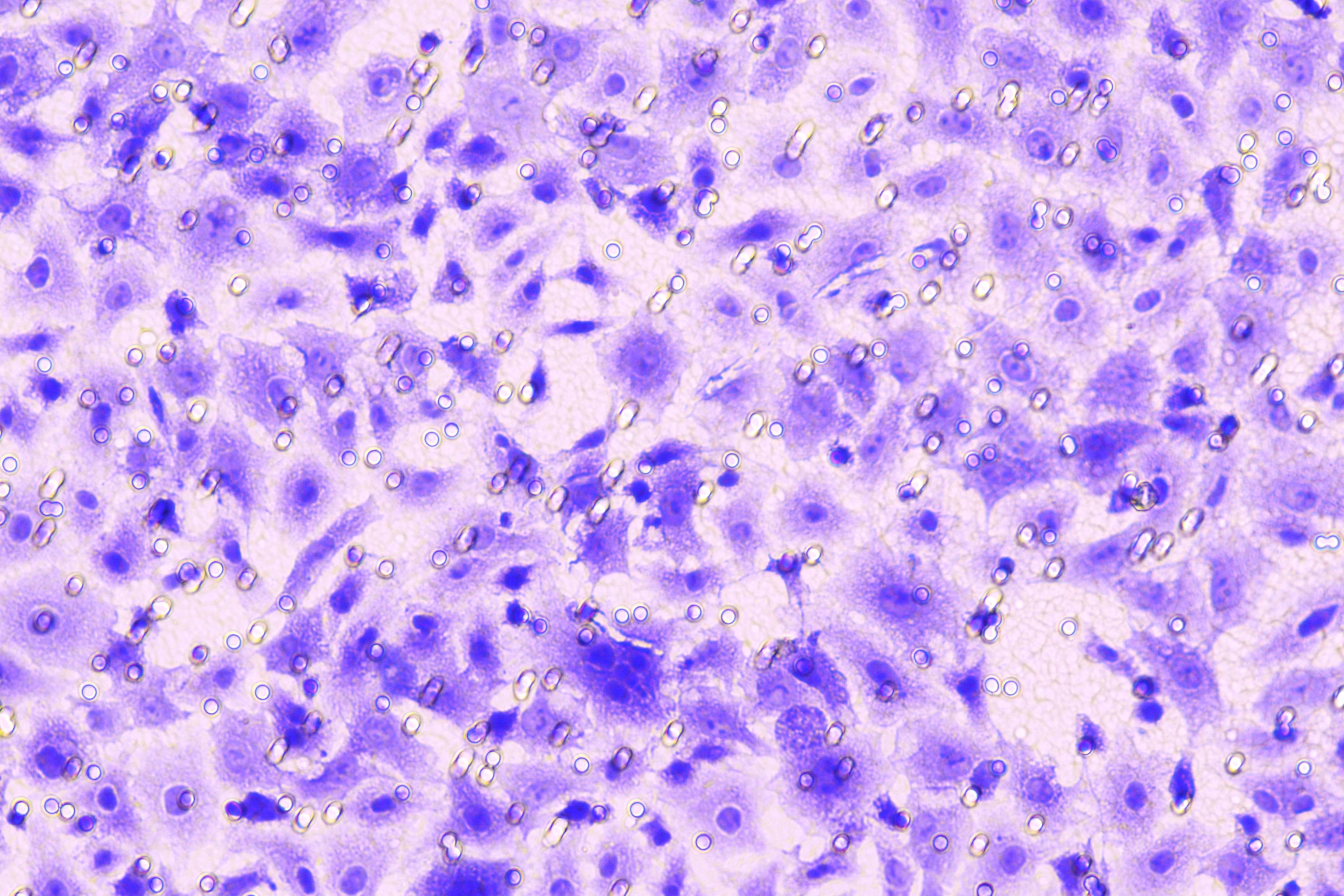

Supplement: Supplemental Information 6 [file peerj-11-14608-s006.zip › micrograph Figure3/C/A549/1.jpg]

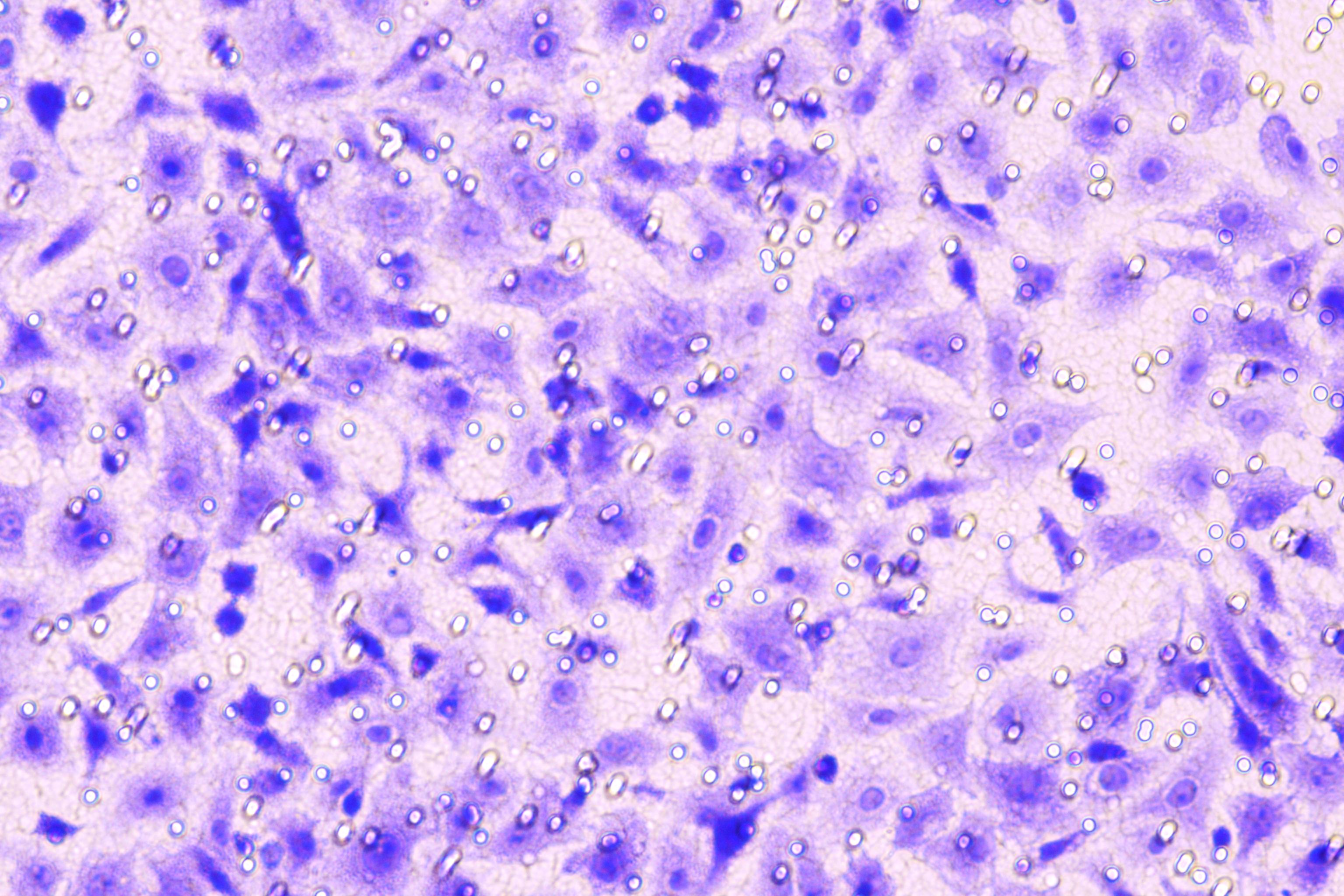

Supplement: Supplemental Information 6 [file peerj-11-14608-s006.zip › micrograph Figure3/C/A549/2.jpg]

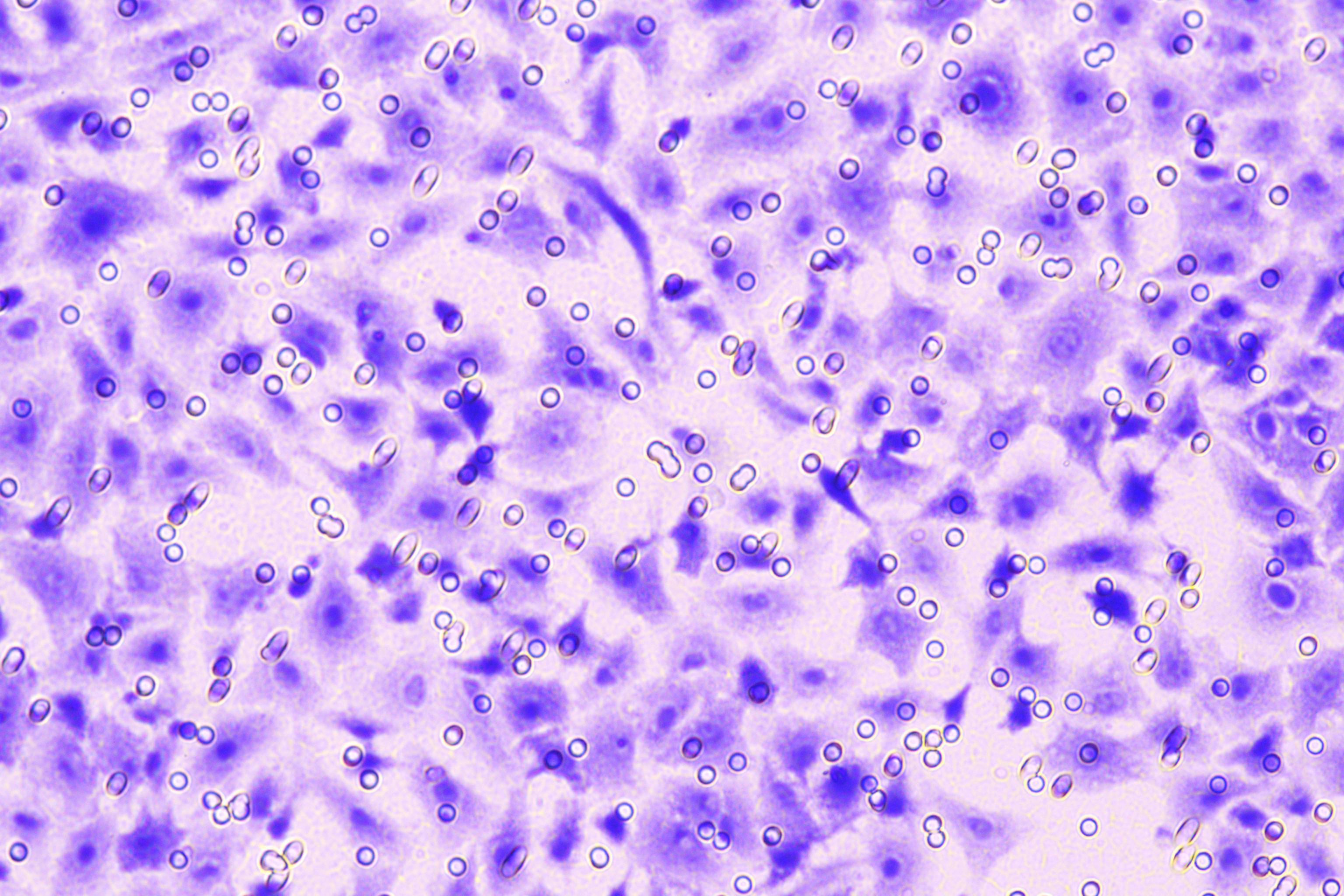

Supplement: Supplemental Information 6 [file peerj-11-14608-s006.zip › micrograph Figure3/C/A549/3.jpg]

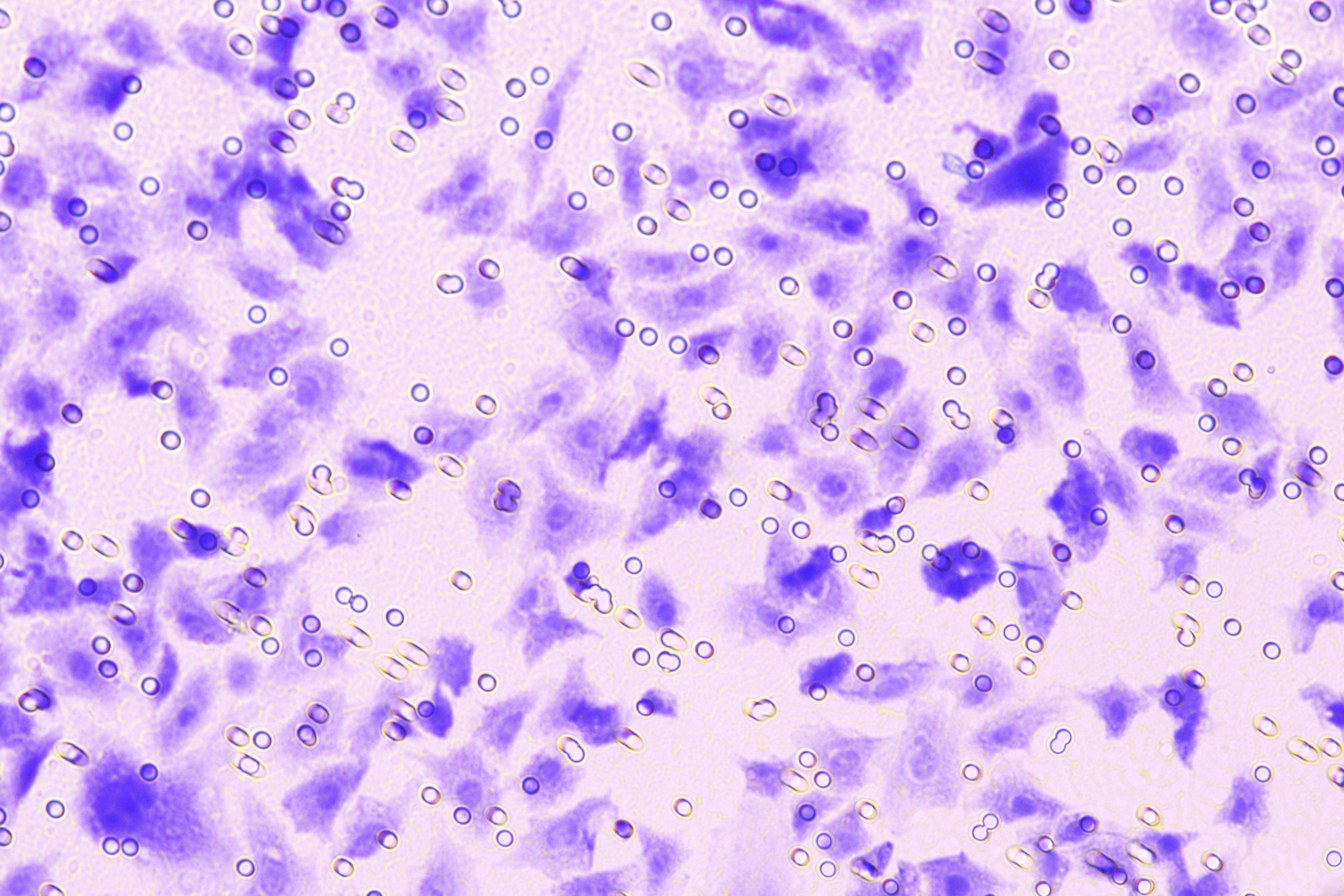

Supplement: Supplemental Information 6 [file peerj-11-14608-s006.zip › micrograph Figure3/C/A549+EXO/1.jpg]

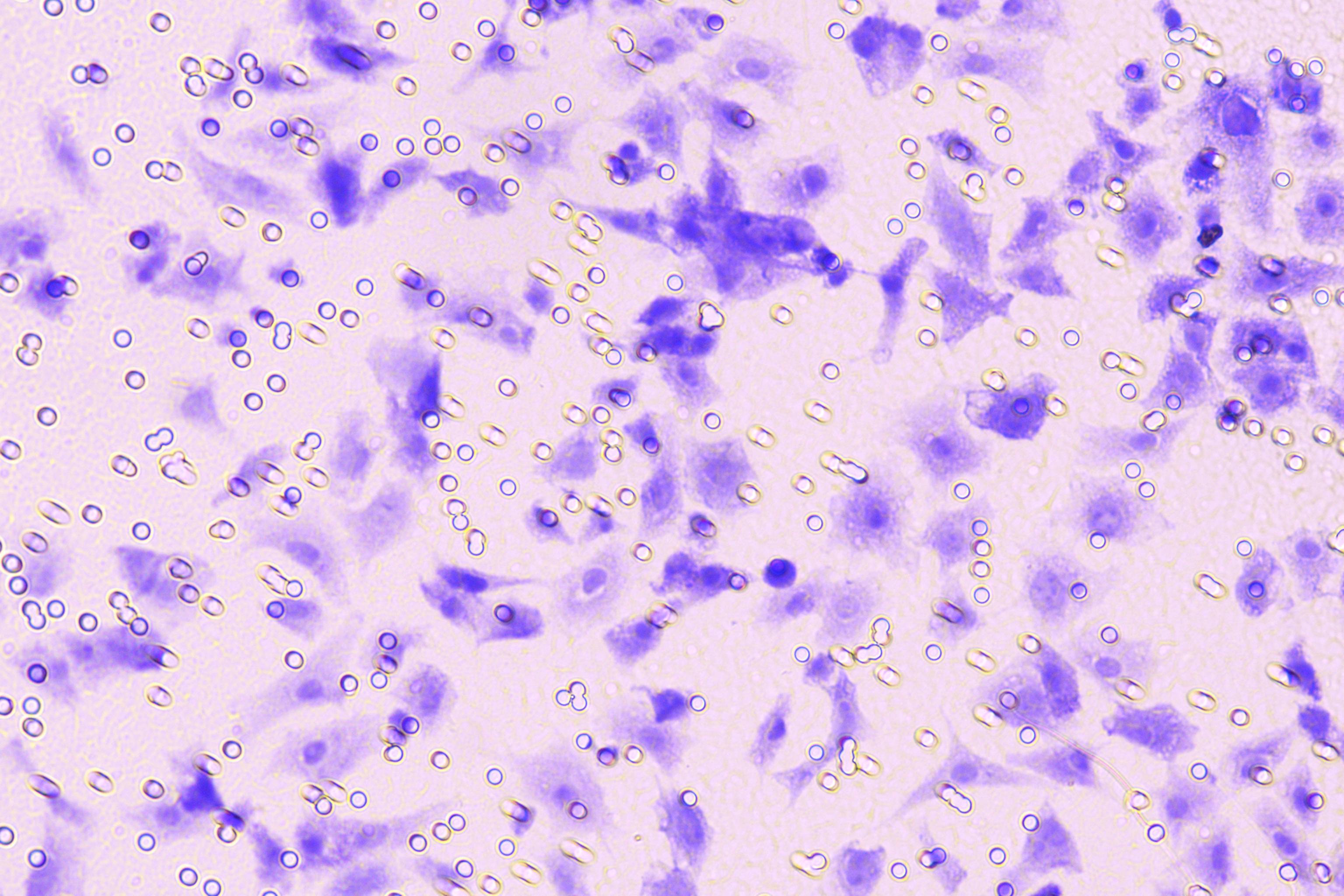

Supplement: Supplemental Information 6 [file peerj-11-14608-s006.zip › micrograph Figure3/C/A549+EXO/2.jpg]

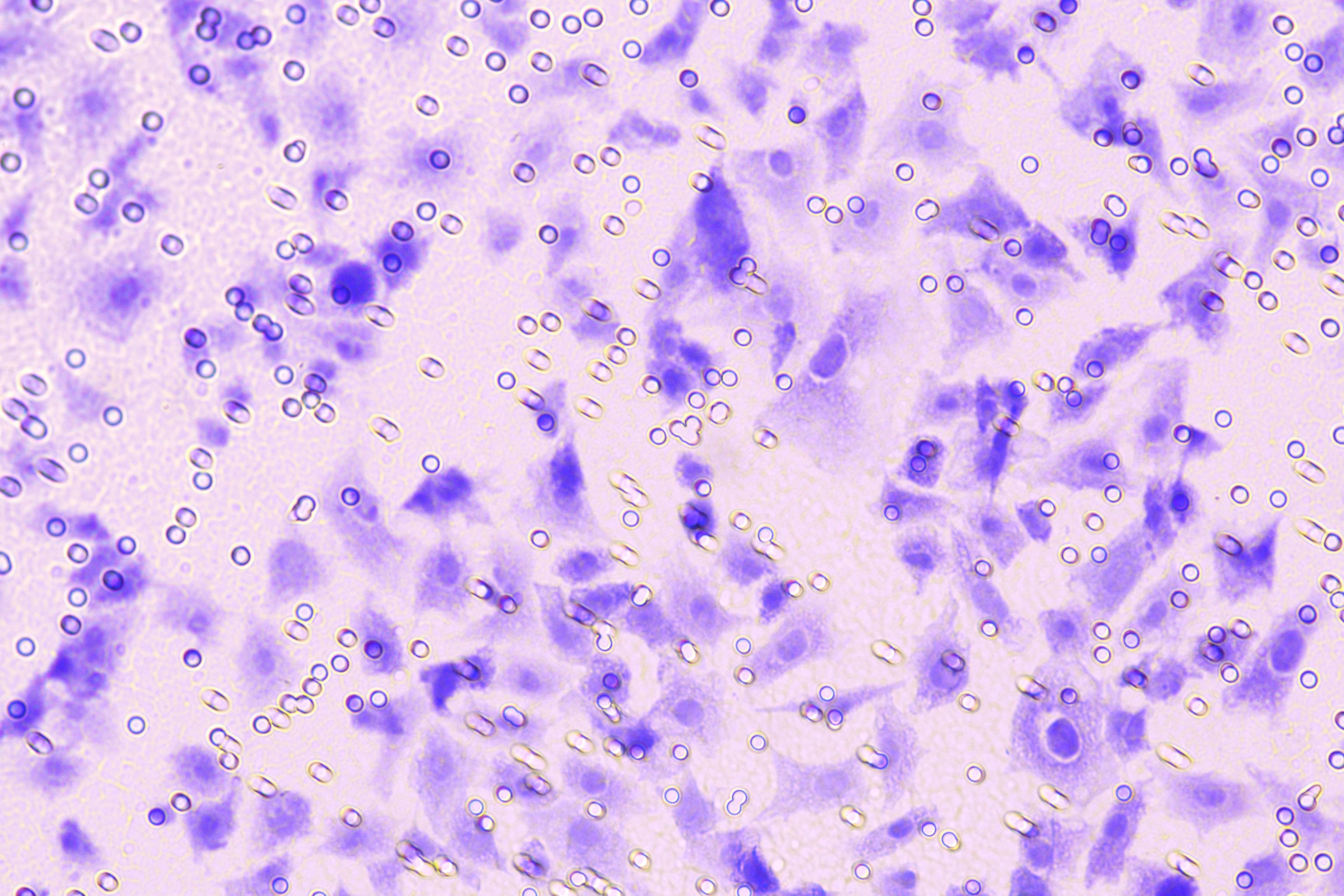

Supplement: Supplemental Information 6 [file peerj-11-14608-s006.zip › micrograph Figure3/C/A549+EXO/3.jpg]

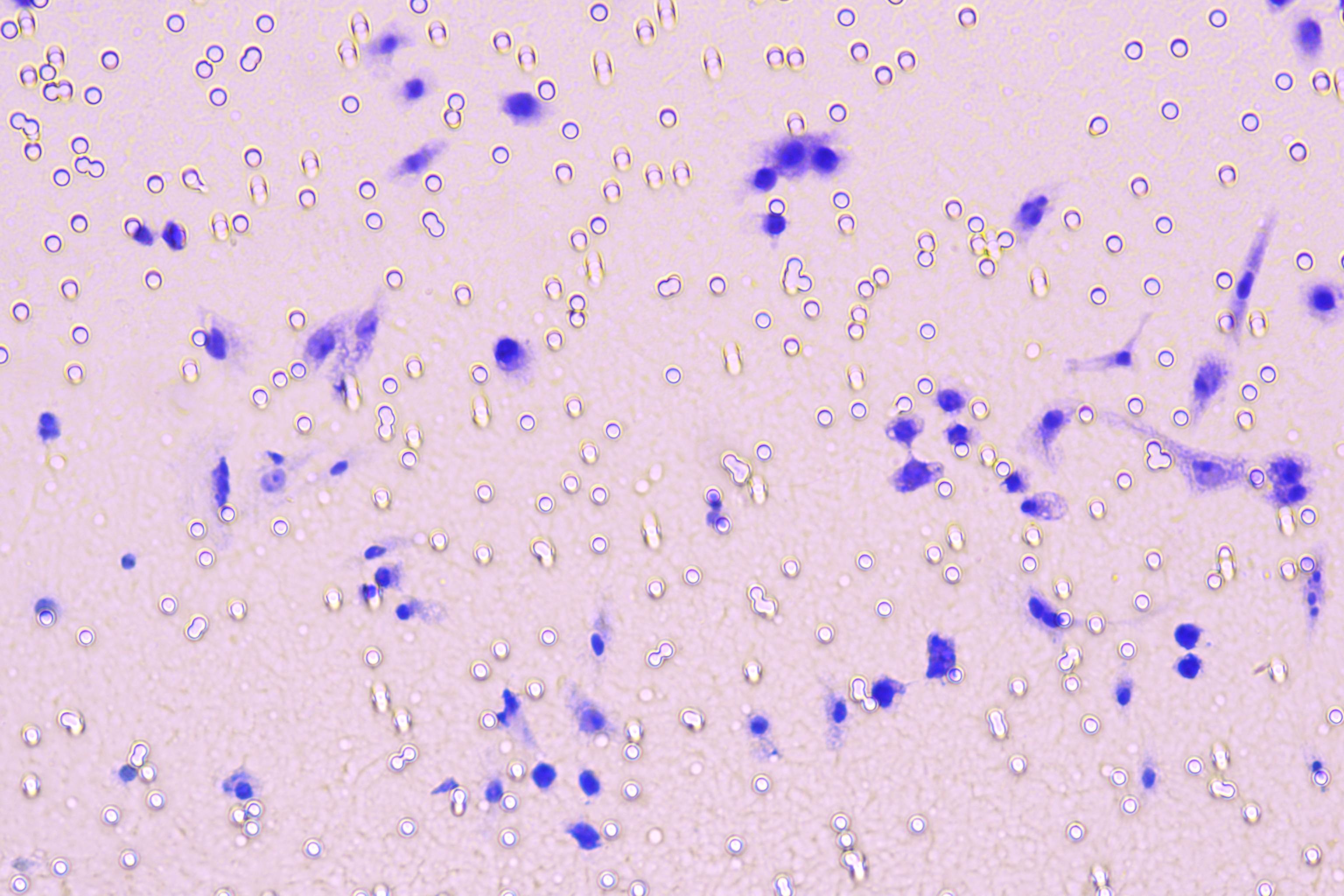

Supplement: Supplemental Information 6 [file peerj-11-14608-s006.zip › micrograph Figure3/C/HLF-A/1.jpg]

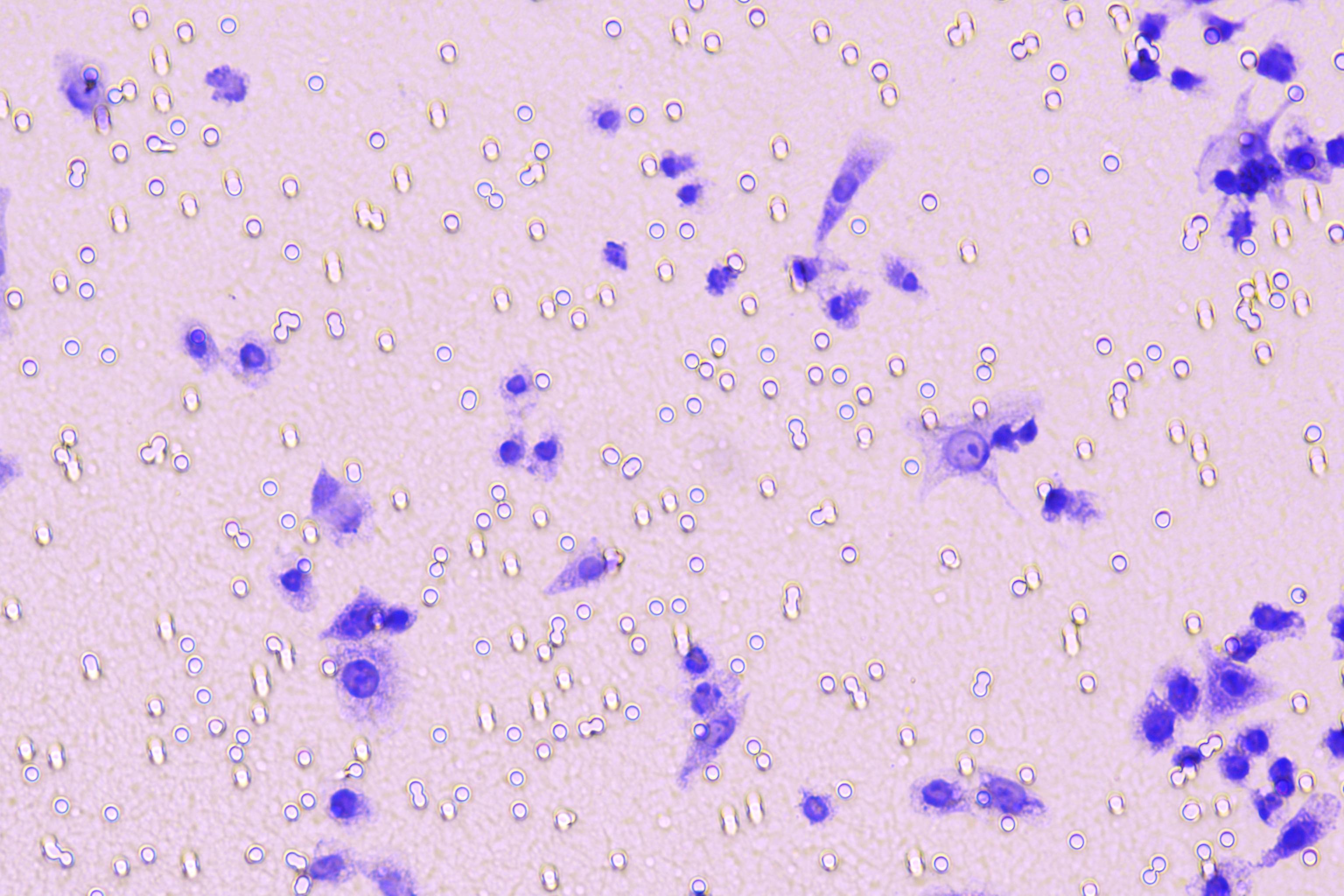

Supplement: Supplemental Information 6 [file peerj-11-14608-s006.zip › micrograph Figure3/C/HLF-A/2.jpg]

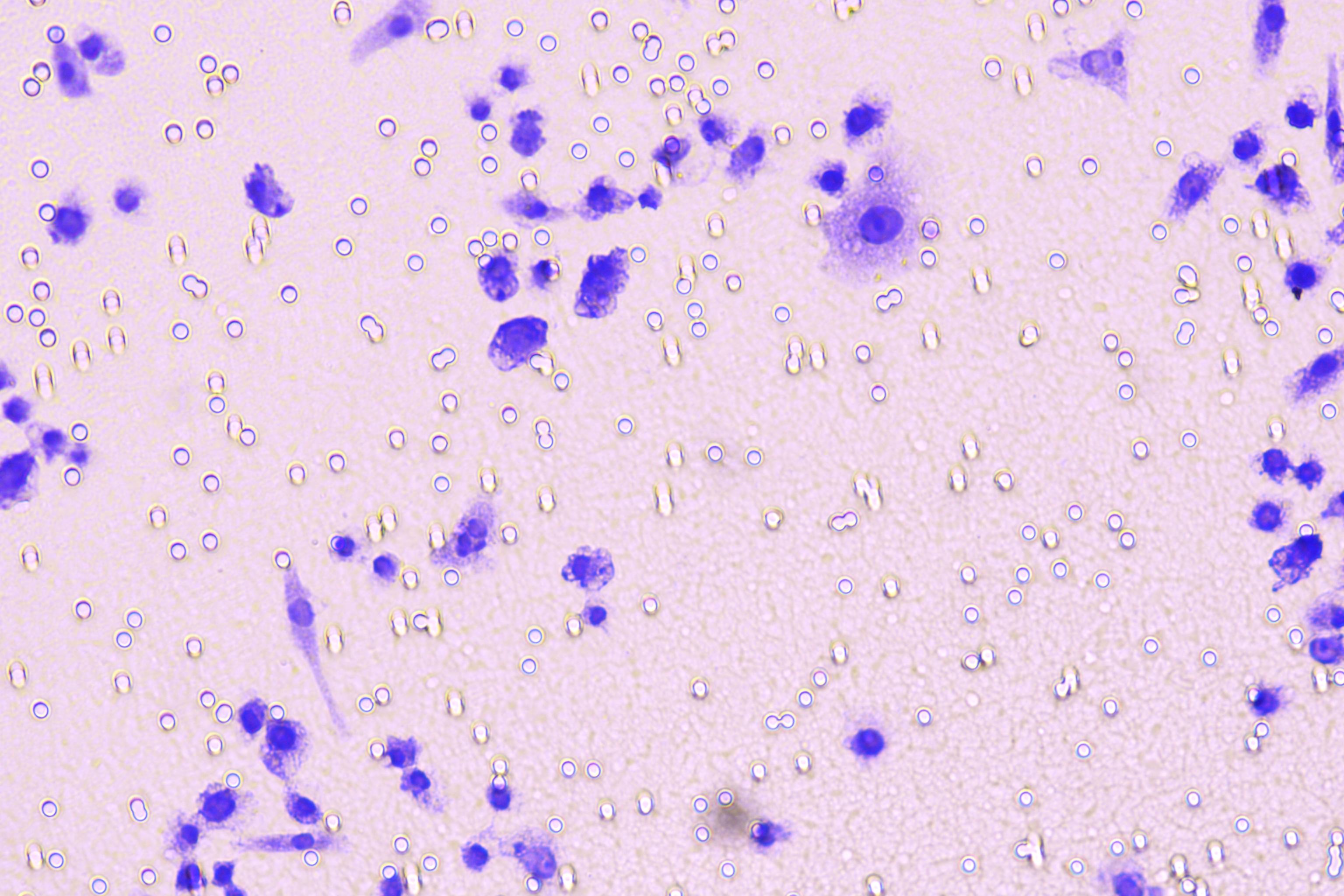

Supplement: Supplemental Information 6 [file peerj-11-14608-s006.zip › micrograph Figure3/C/HLF-A/3.jpg]

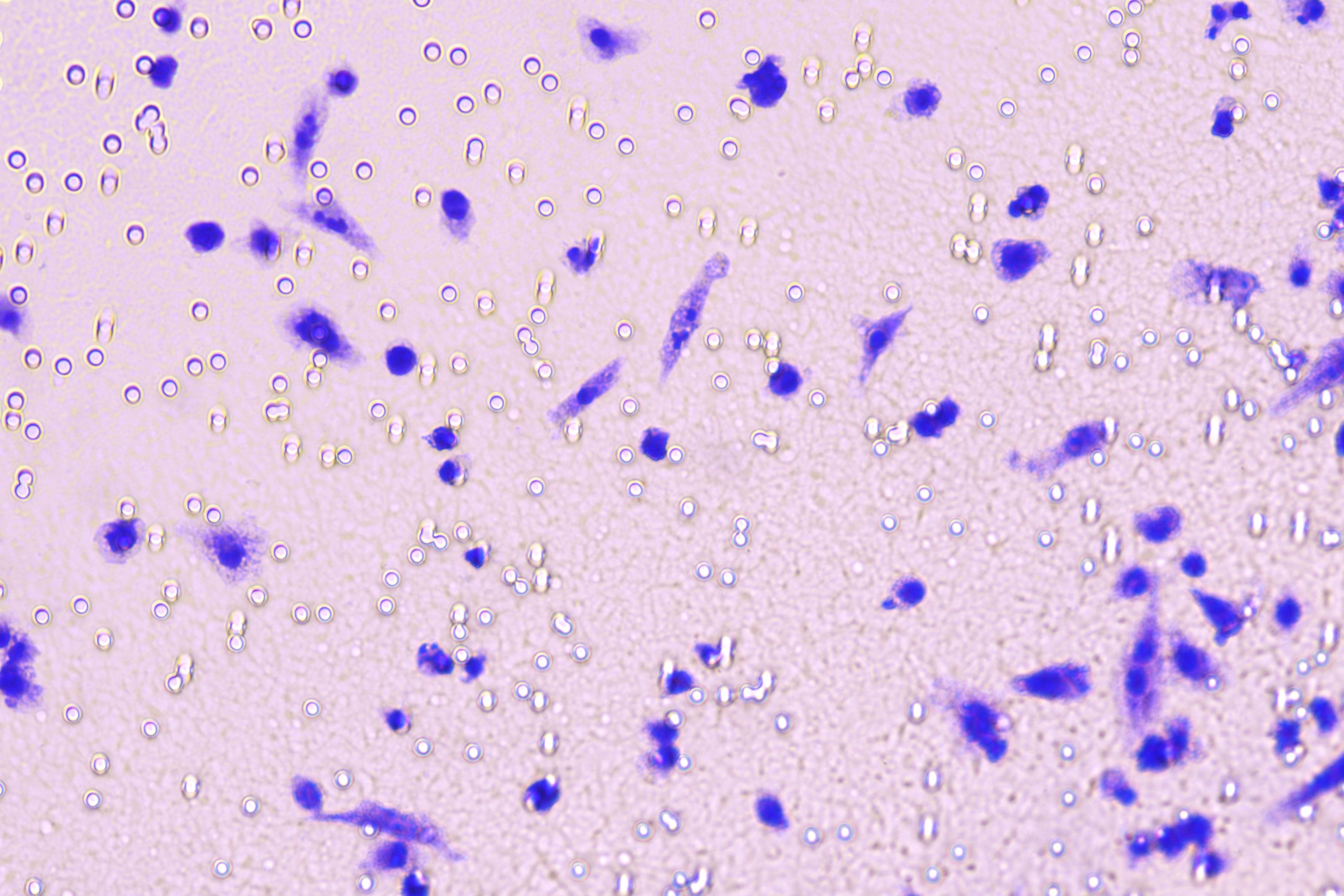

Supplement: Supplemental Information 6 [file peerj-11-14608-s006.zip › micrograph Figure3/C/HLF-A+EXO/1.jpg]

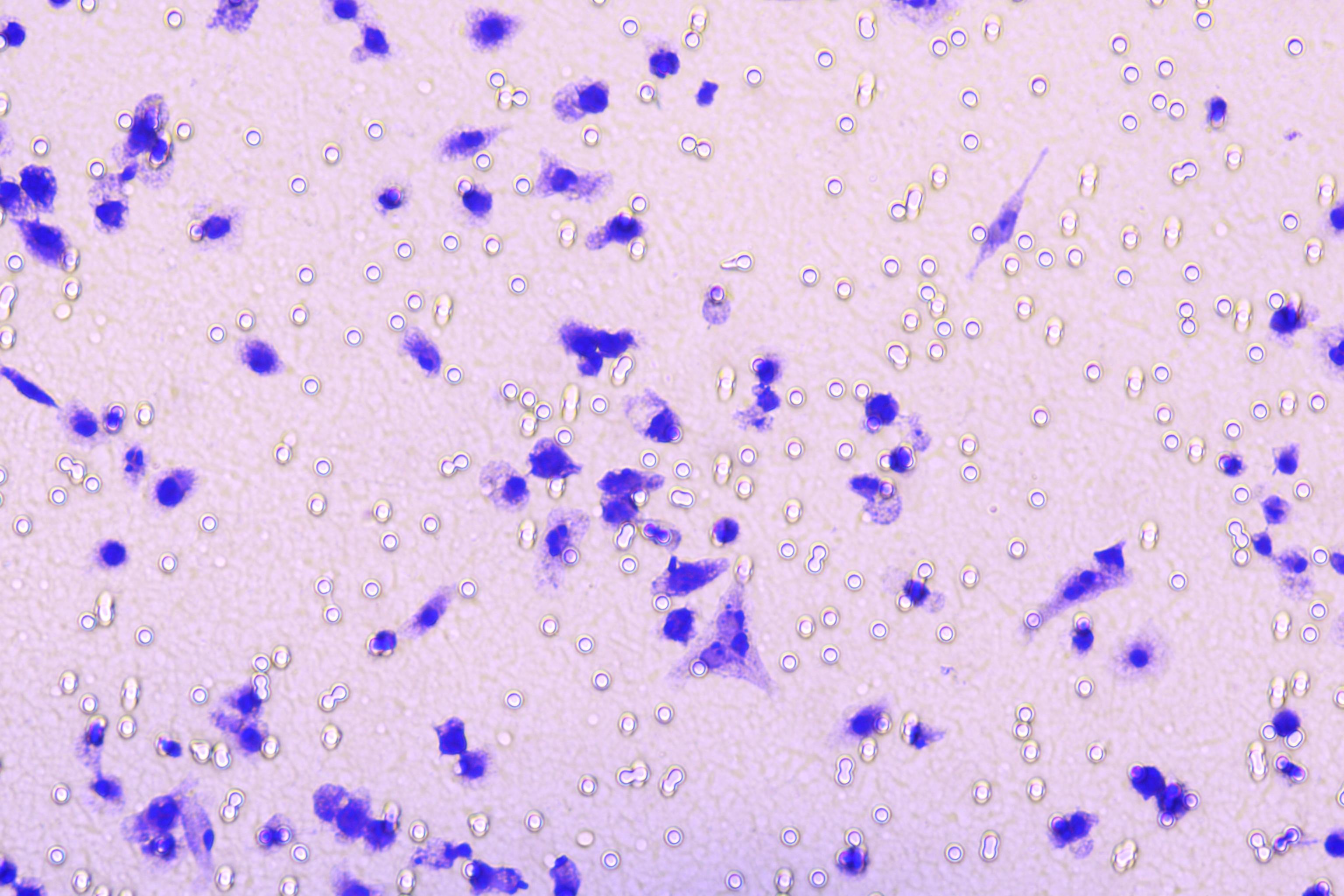

Supplement: Supplemental Information 6 [file peerj-11-14608-s006.zip › micrograph Figure3/C/HLF-A+EXO/2.jpg]

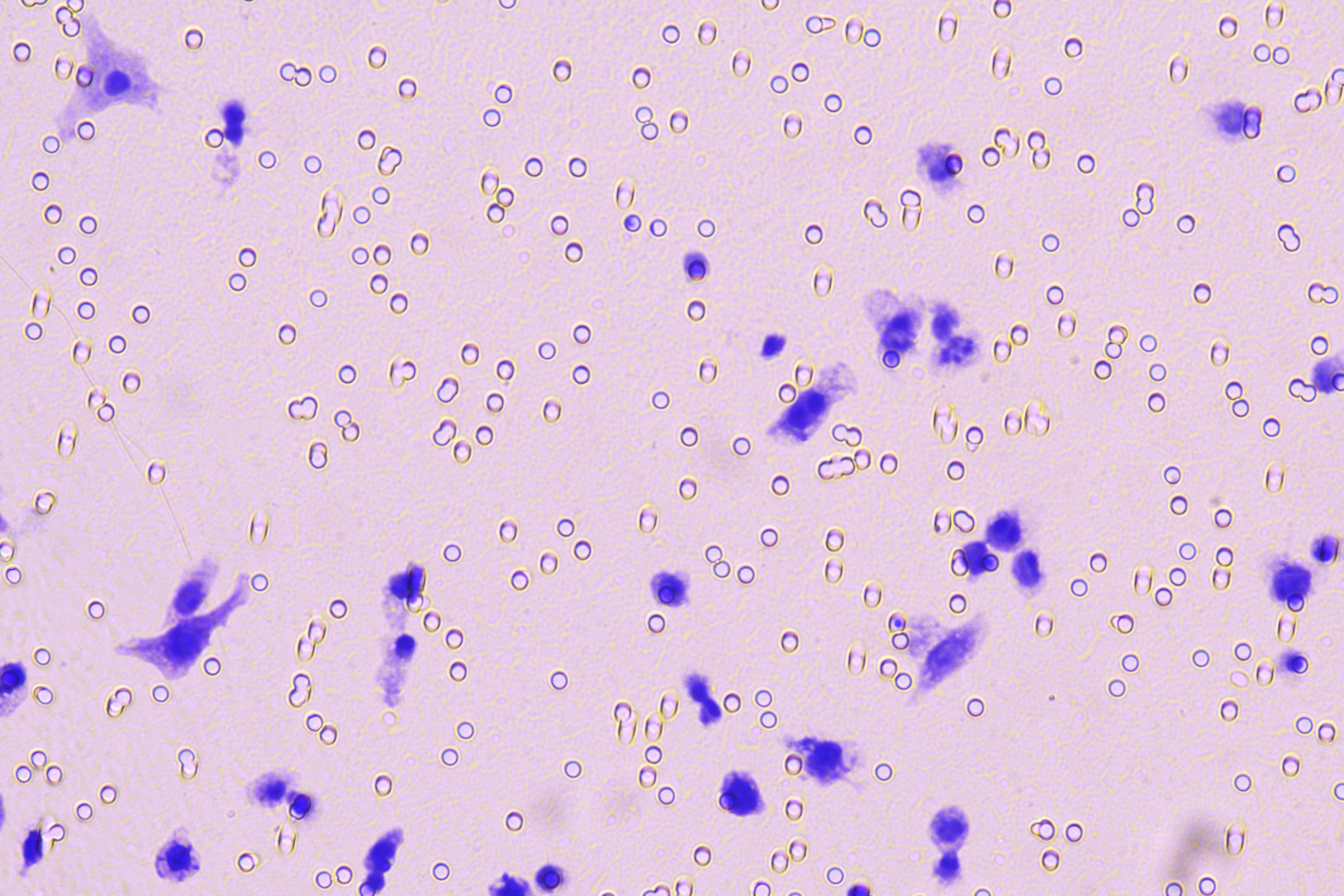

Supplement: Supplemental Information 6 [file peerj-11-14608-s006.zip › micrograph Figure3/C/HLF-A+EXO/3.jpg]

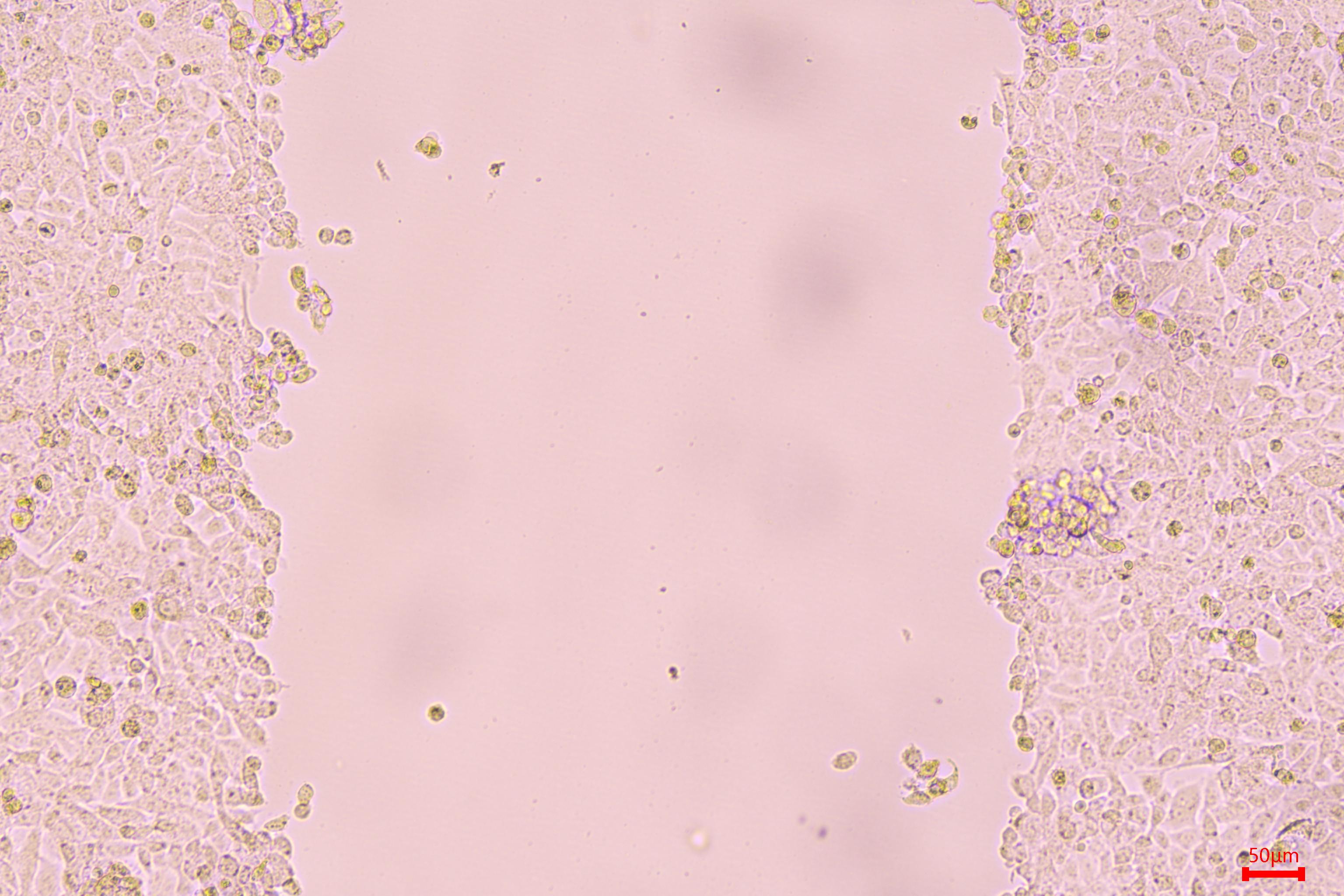

Supplement: Supplemental Information 8 [file peerj-11-14608-s008.zip › Figure 6 image/B/ASO-GNG5/0h(1).jpg]

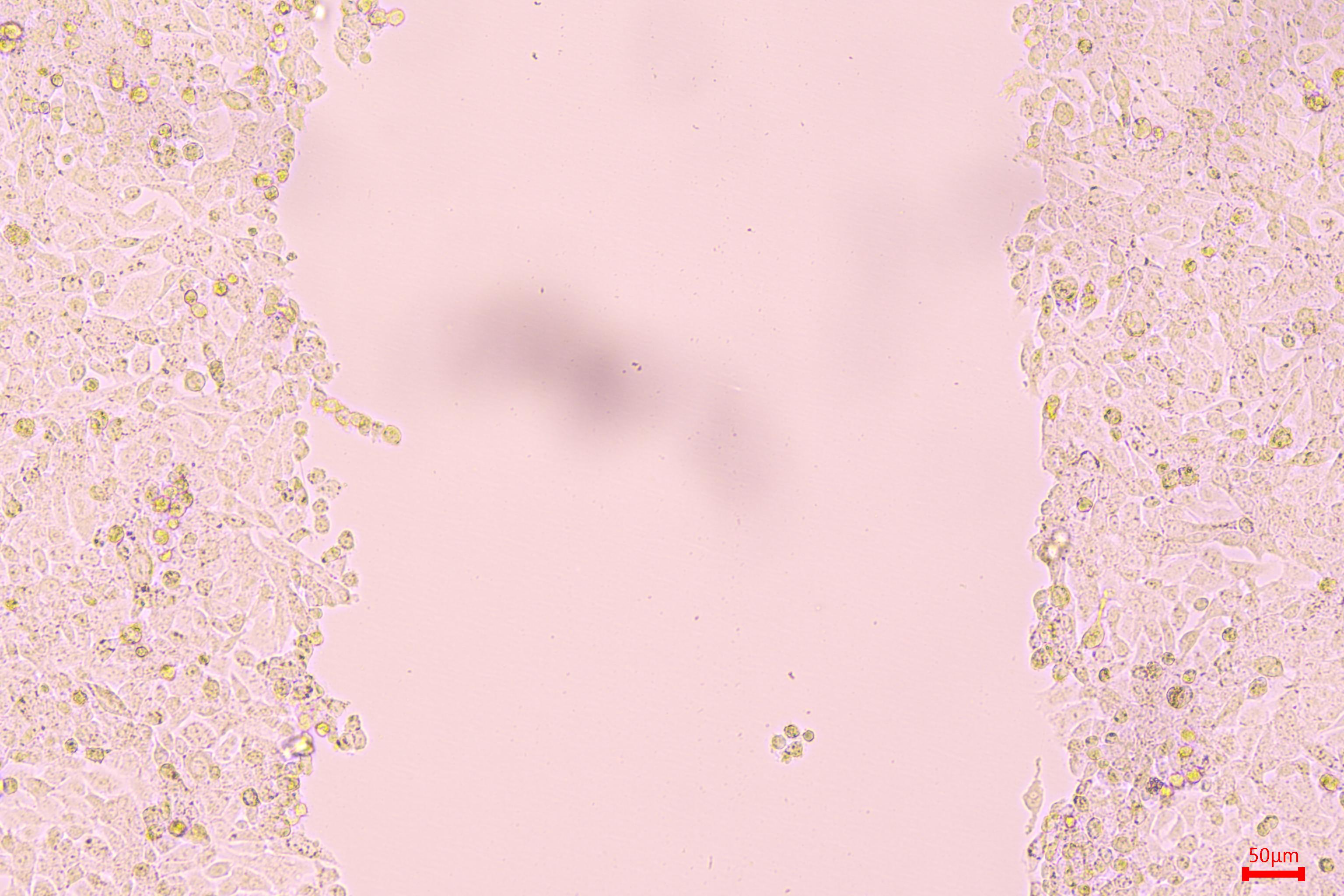

Supplement: Supplemental Information 8 [file peerj-11-14608-s008.zip › Figure 6 image/B/ASO-GNG5/0h(2).jpg]

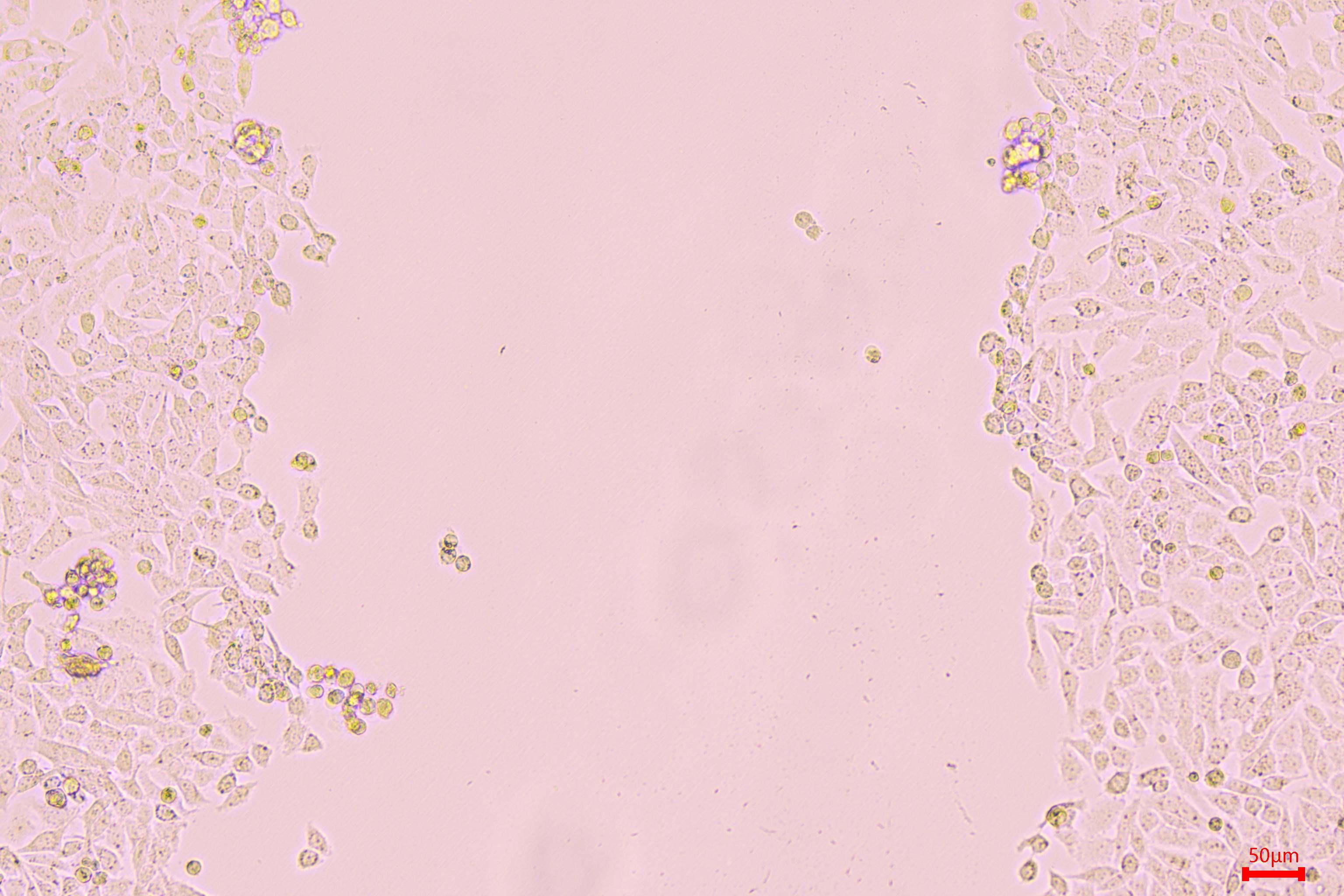

Supplement: Supplemental Information 8 [file peerj-11-14608-s008.zip › Figure 6 image/B/ASO-GNG5/0h(3).jpg]

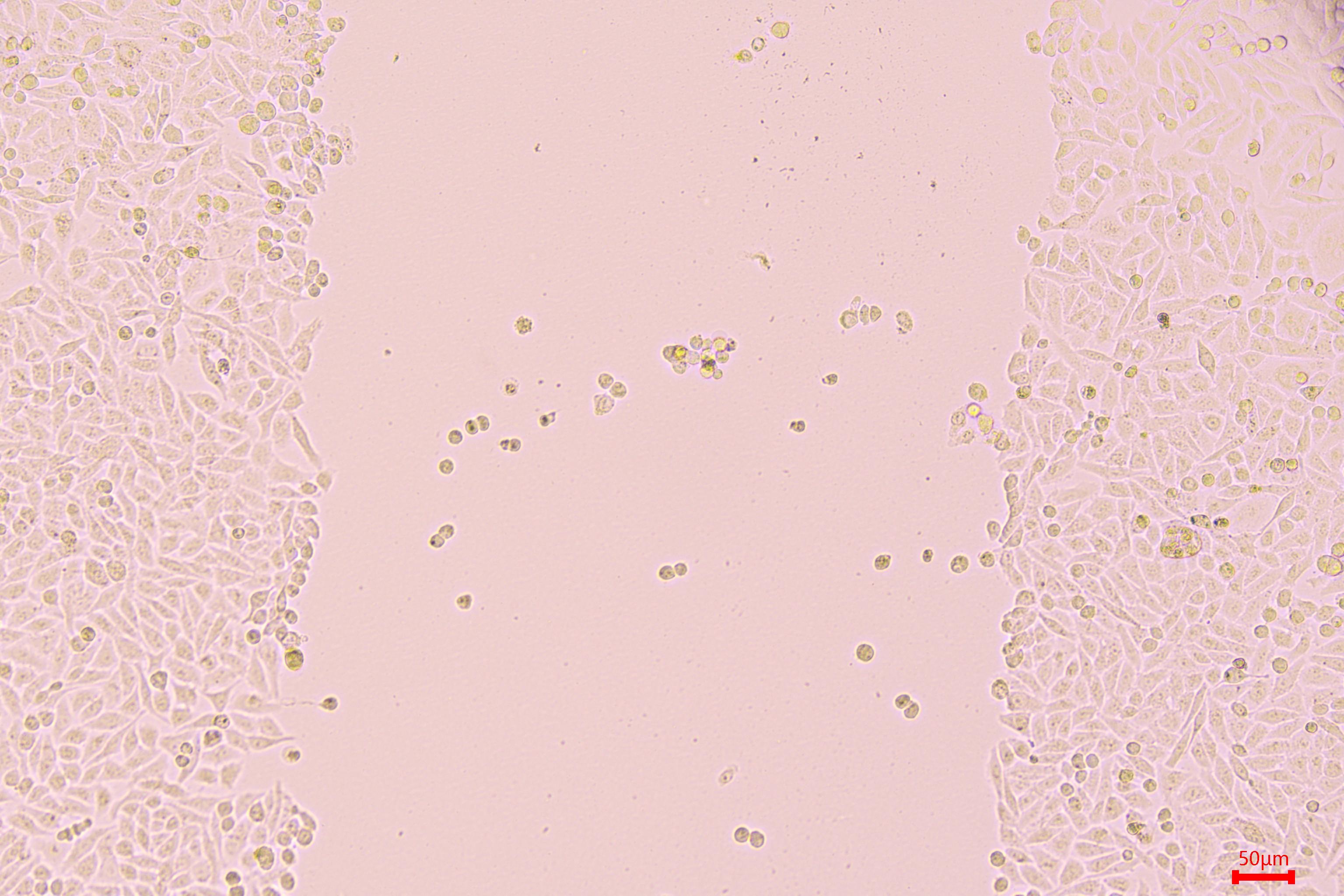

Supplement: Supplemental Information 8 [file peerj-11-14608-s008.zip › Figure 6 image/B/ASO-GNG5/48h (1).jpg]

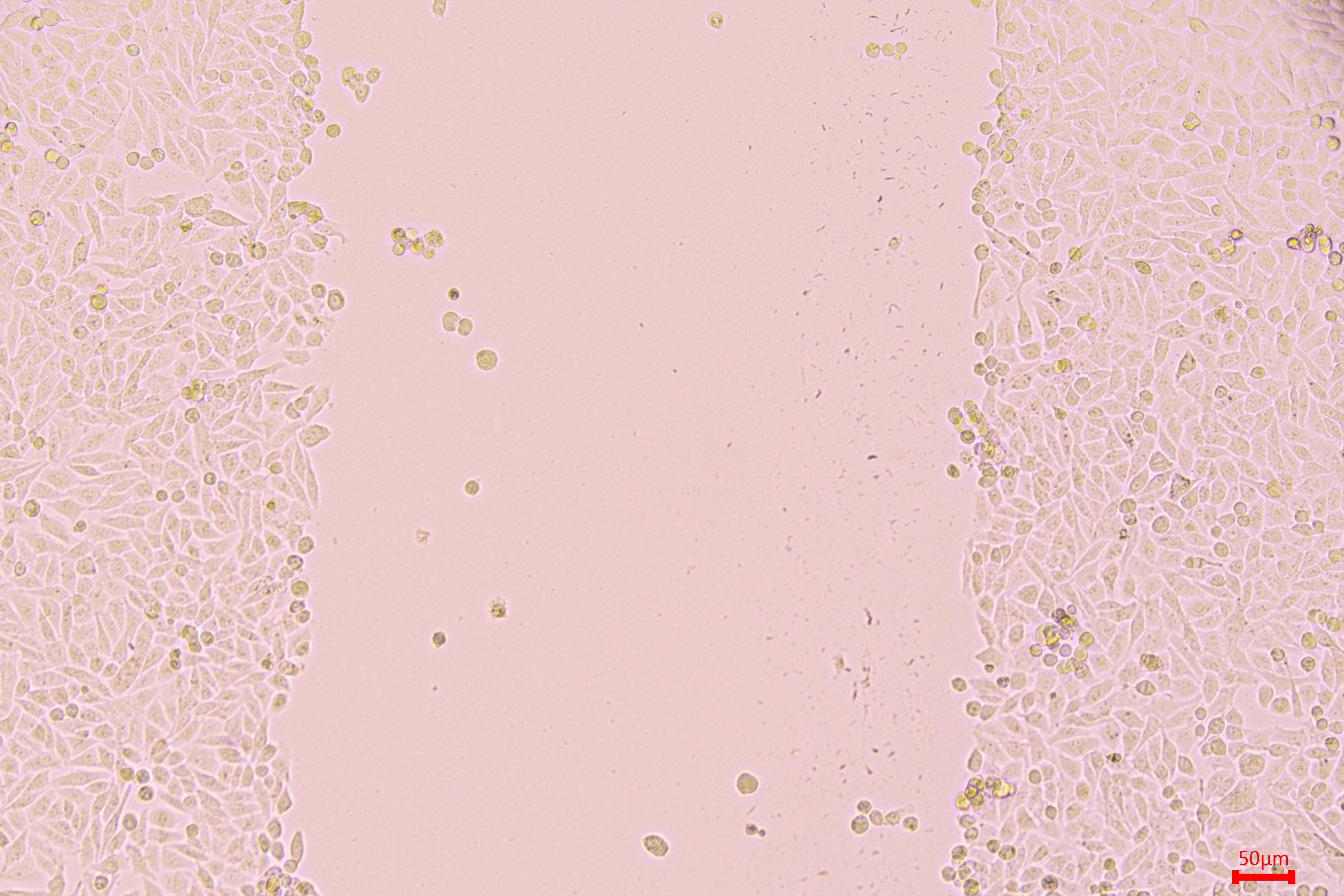

Supplement: Supplemental Information 8 [file peerj-11-14608-s008.zip › Figure 6 image/B/ASO-GNG5/48h (2).jpg]

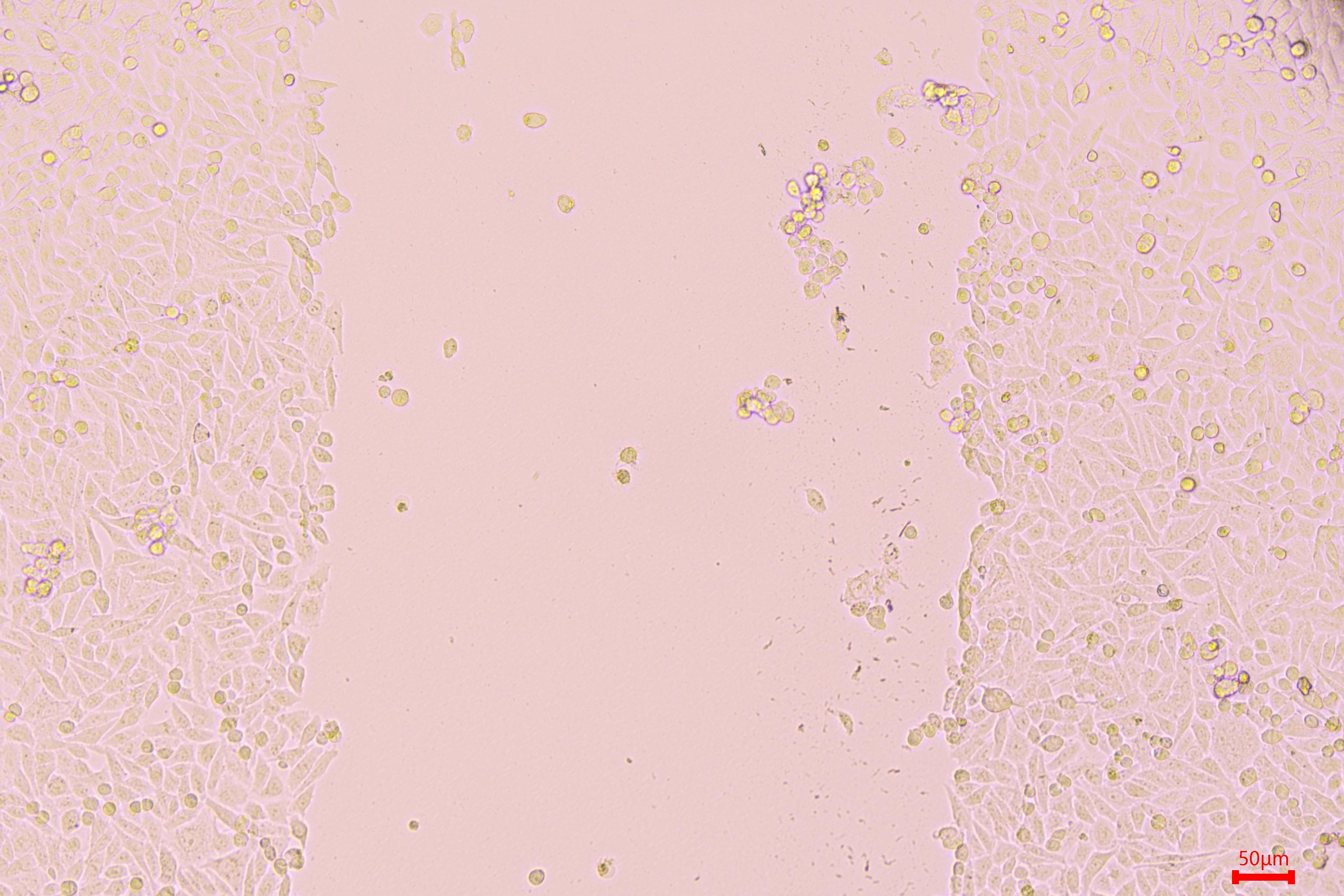

Supplement: Supplemental Information 8 [file peerj-11-14608-s008.zip › Figure 6 image/B/ASO-GNG5/48h (3).jpg]

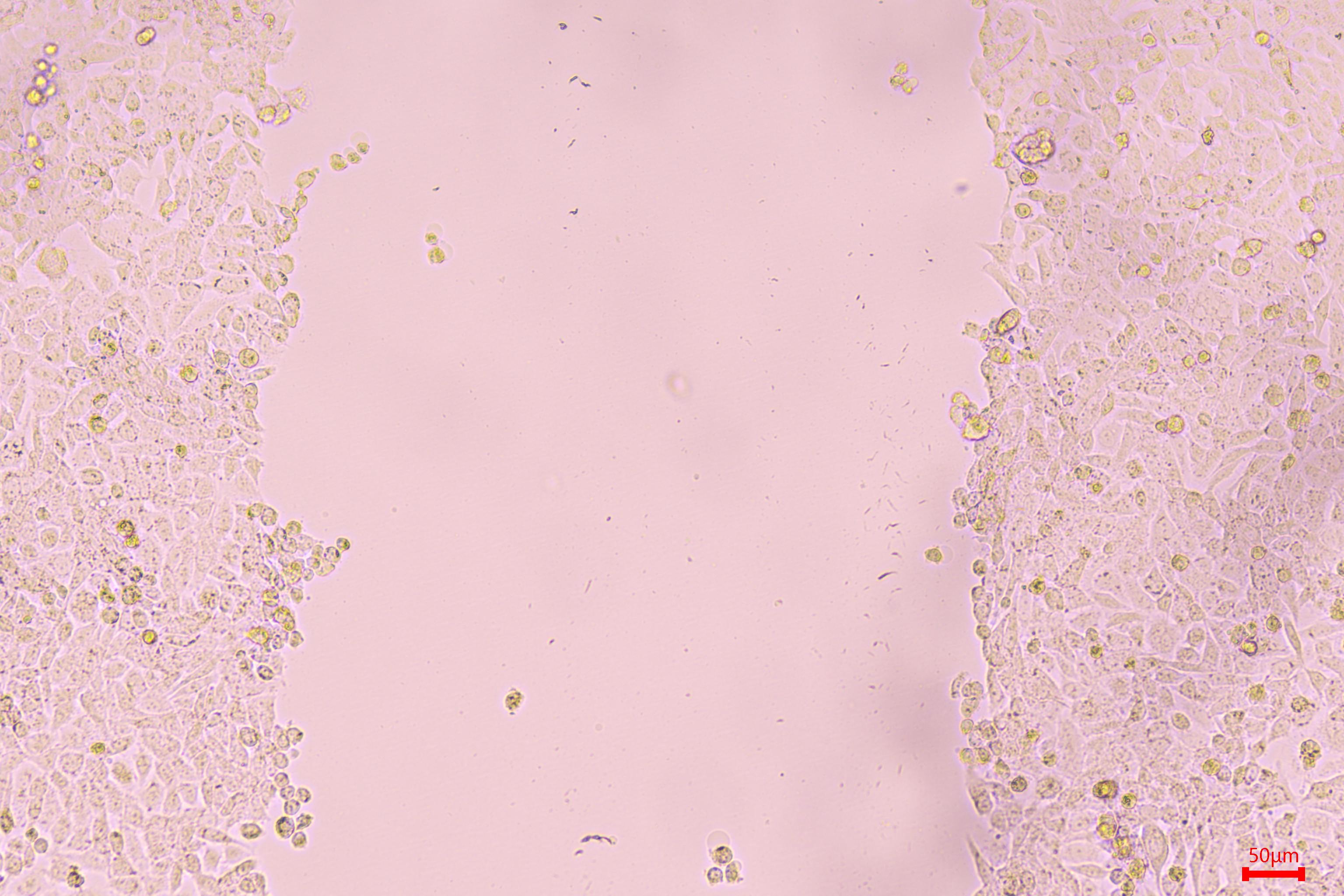

Supplement: Supplemental Information 8 [file peerj-11-14608-s008.zip › Figure 6 image/B/ASO-NC/0h(1).jpg]

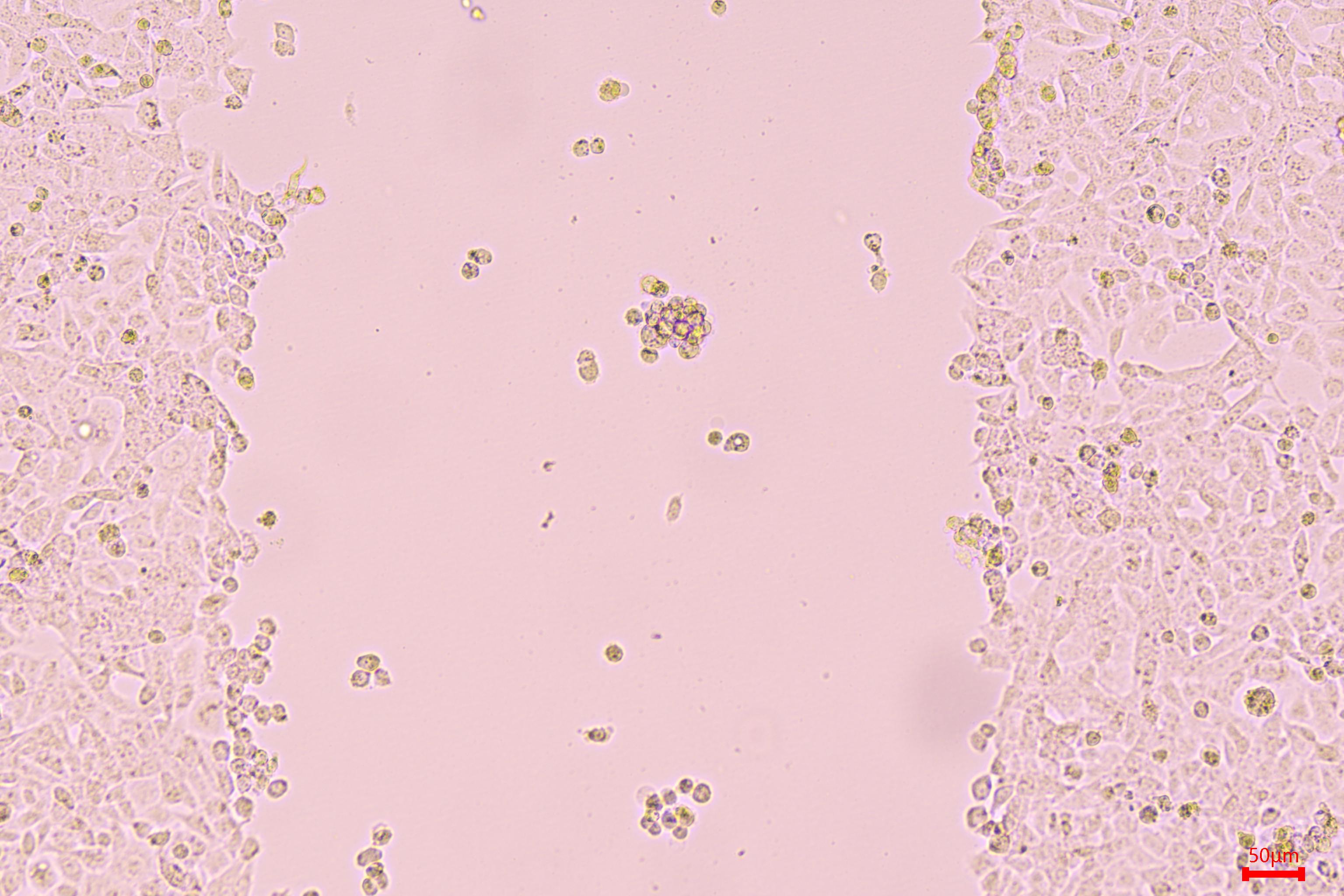

Supplement: Supplemental Information 8 [file peerj-11-14608-s008.zip › Figure 6 image/B/ASO-NC/0h(2).jpg]

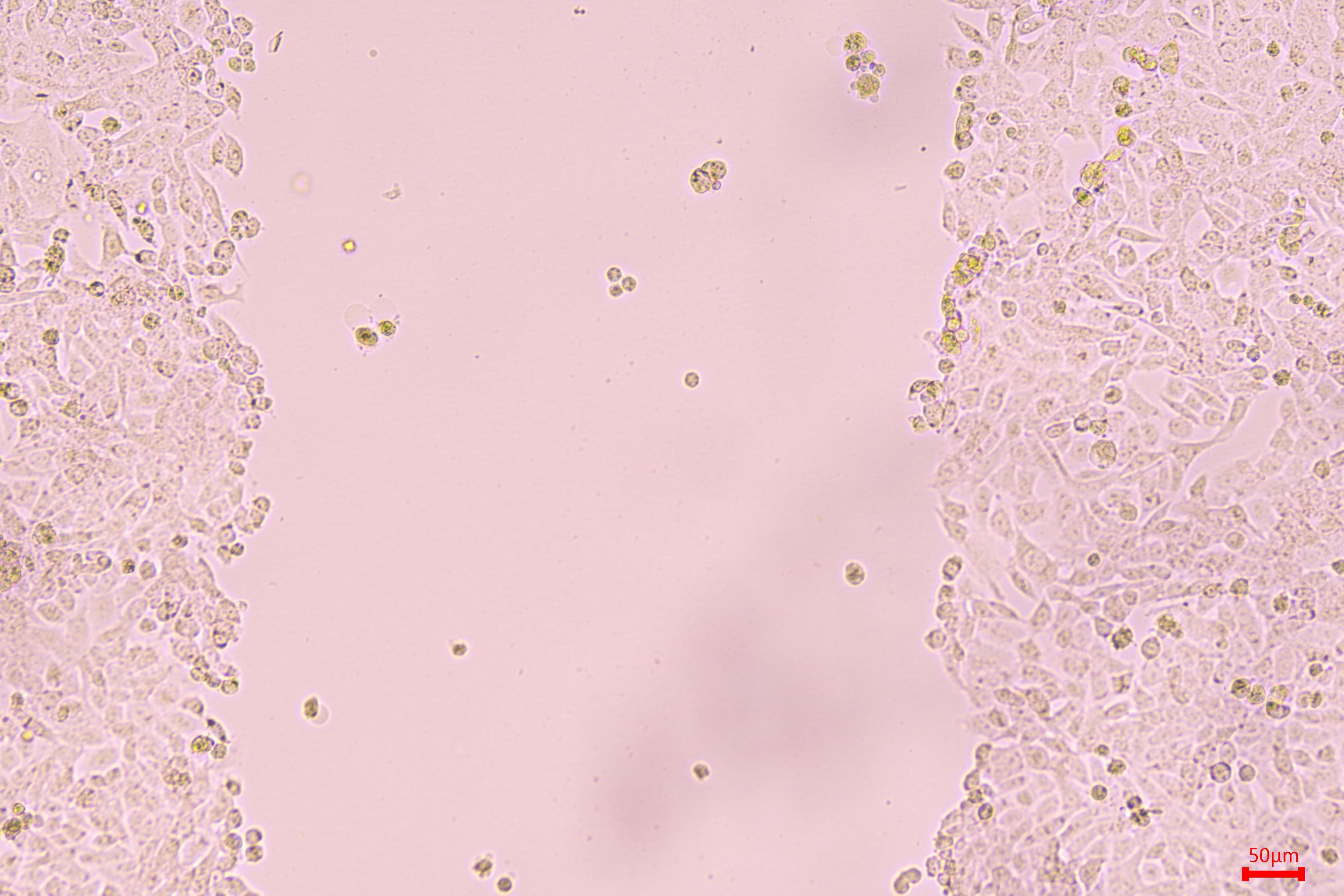

Supplement: Supplemental Information 8 [file peerj-11-14608-s008.zip › Figure 6 image/B/ASO-NC/0h(3).jpg]

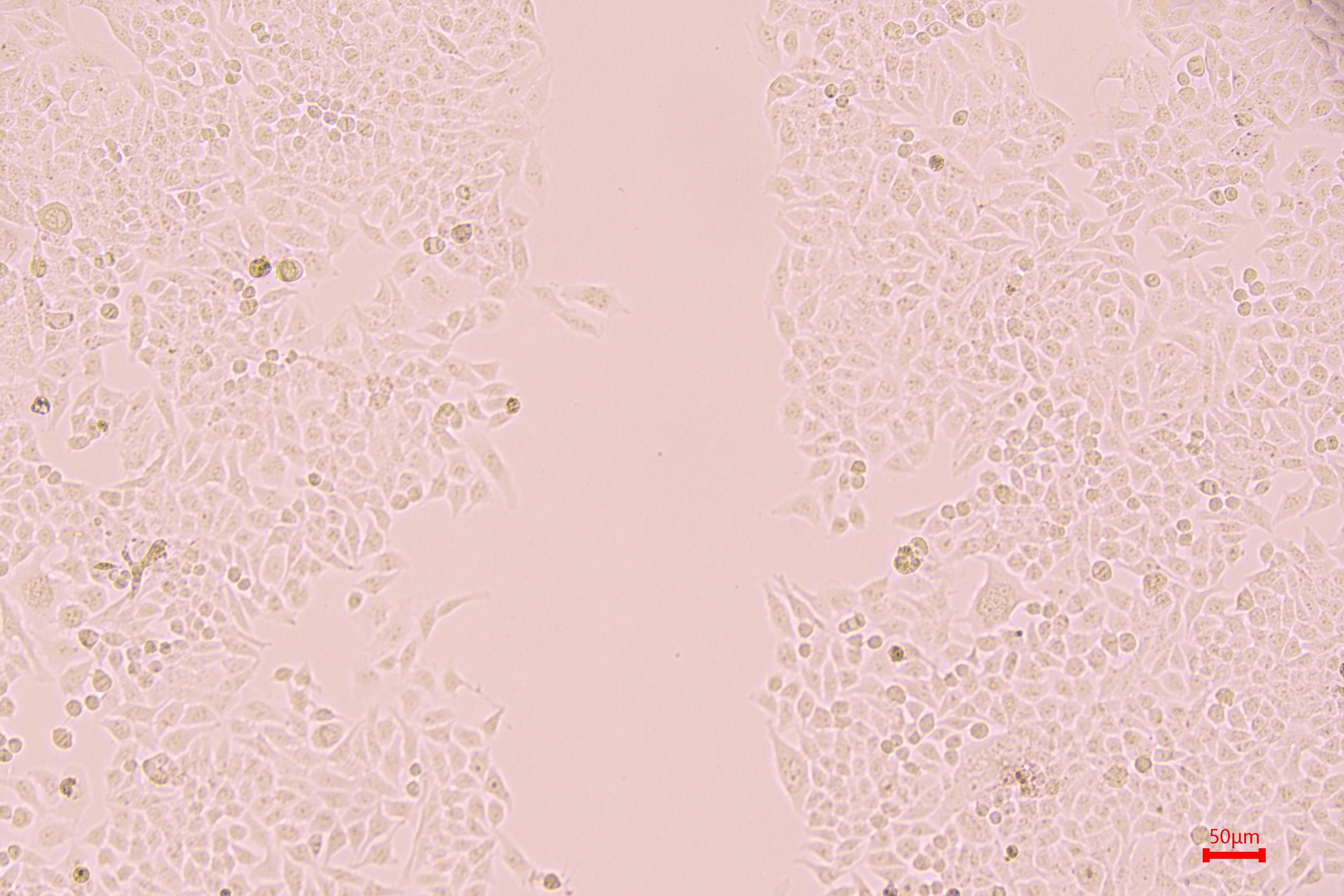

Supplement: Supplemental Information 8 [file peerj-11-14608-s008.zip › Figure 6 image/B/ASO-NC/48h (1).jpg]

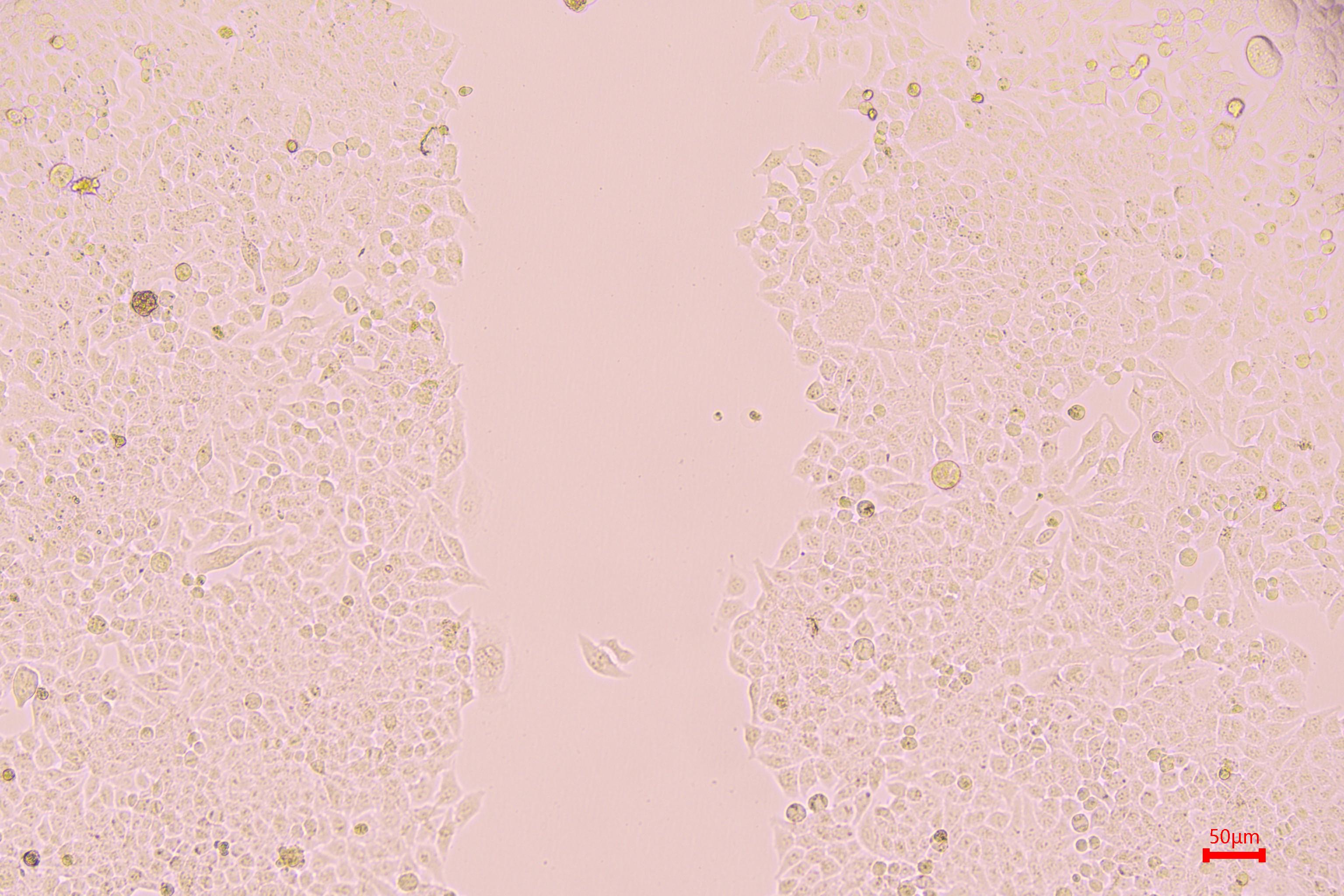

Supplement: Supplemental Information 8 [file peerj-11-14608-s008.zip › Figure 6 image/B/ASO-NC/48h (2).jpg]

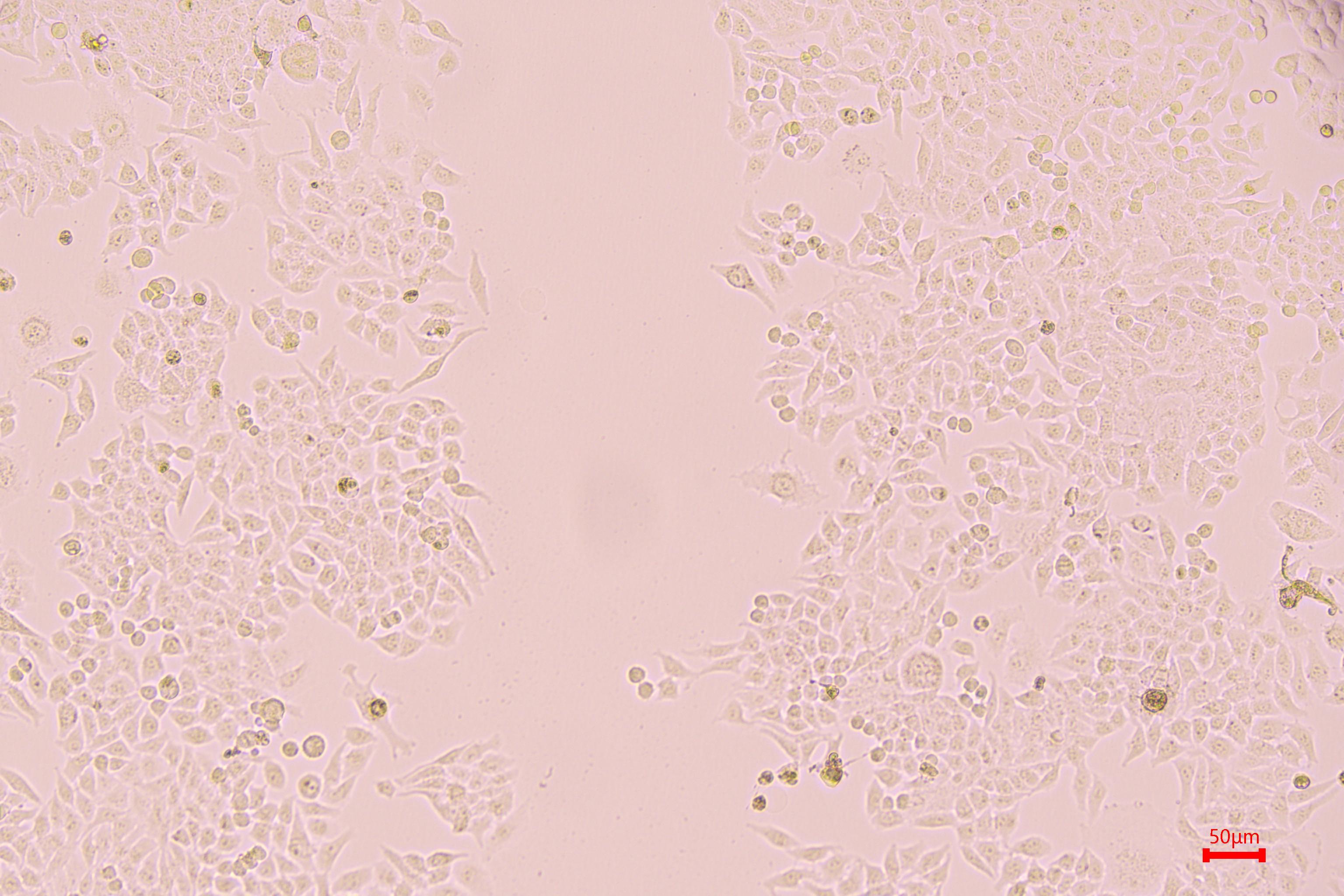

Supplement: Supplemental Information 8 [file peerj-11-14608-s008.zip › Figure 6 image/B/ASO-NC/48h (3).jpg]

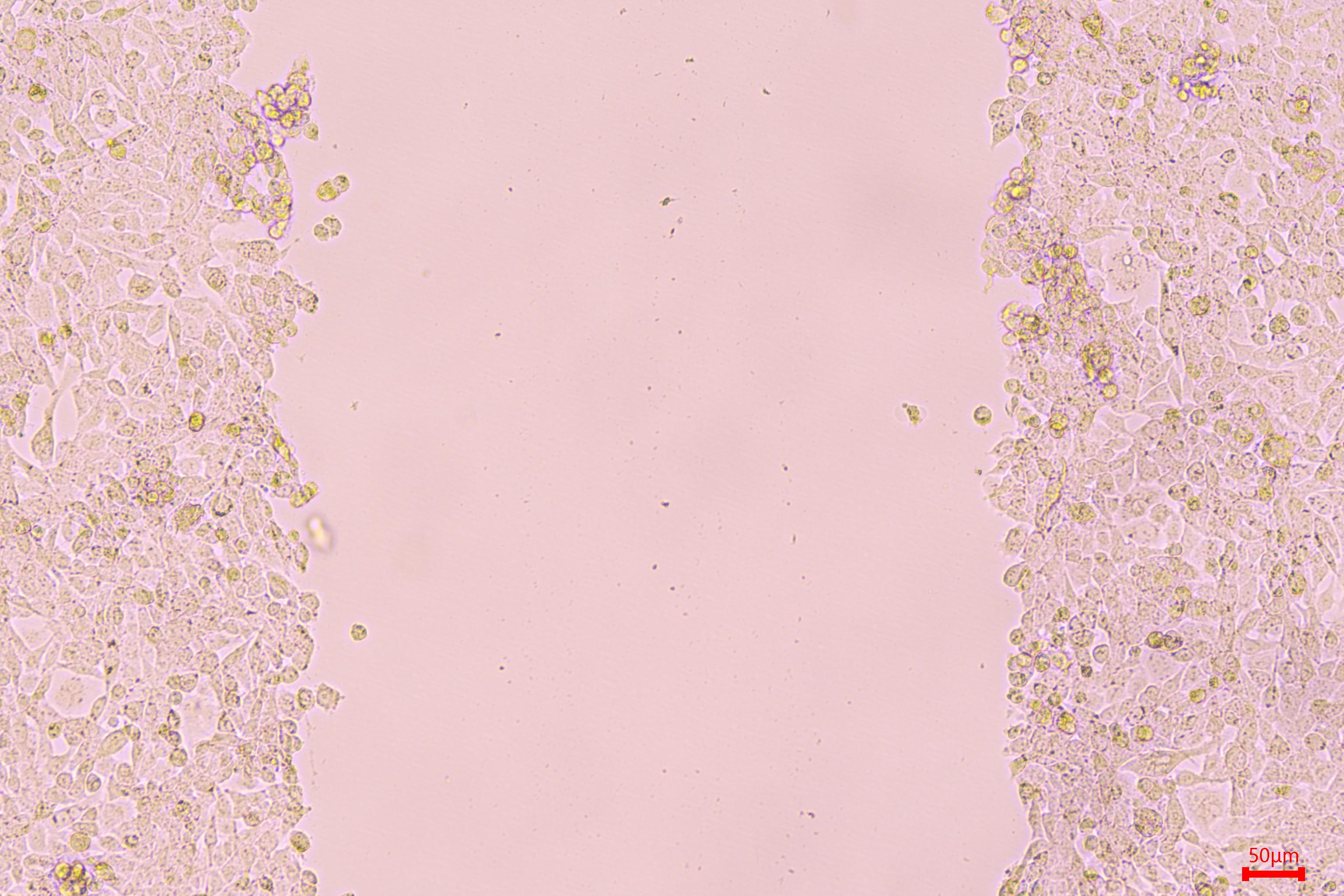

Supplement: Supplemental Information 8 [file peerj-11-14608-s008.zip › Figure 6 image/B/pcDNA3.1/0h (1).jpg]

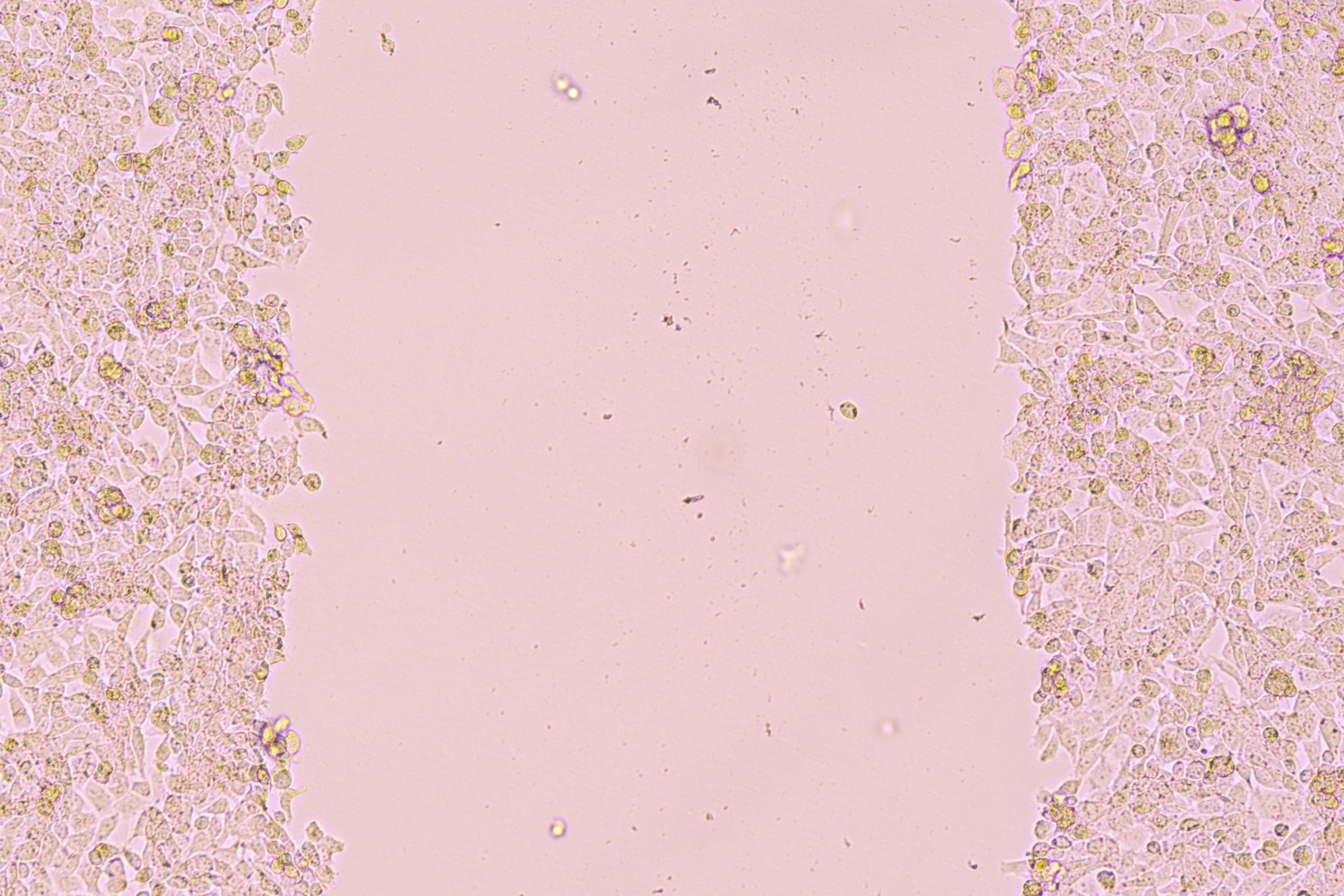

Supplement: Supplemental Information 8 [file peerj-11-14608-s008.zip › Figure 6 image/B/pcDNA3.1/0h (2).jpg]

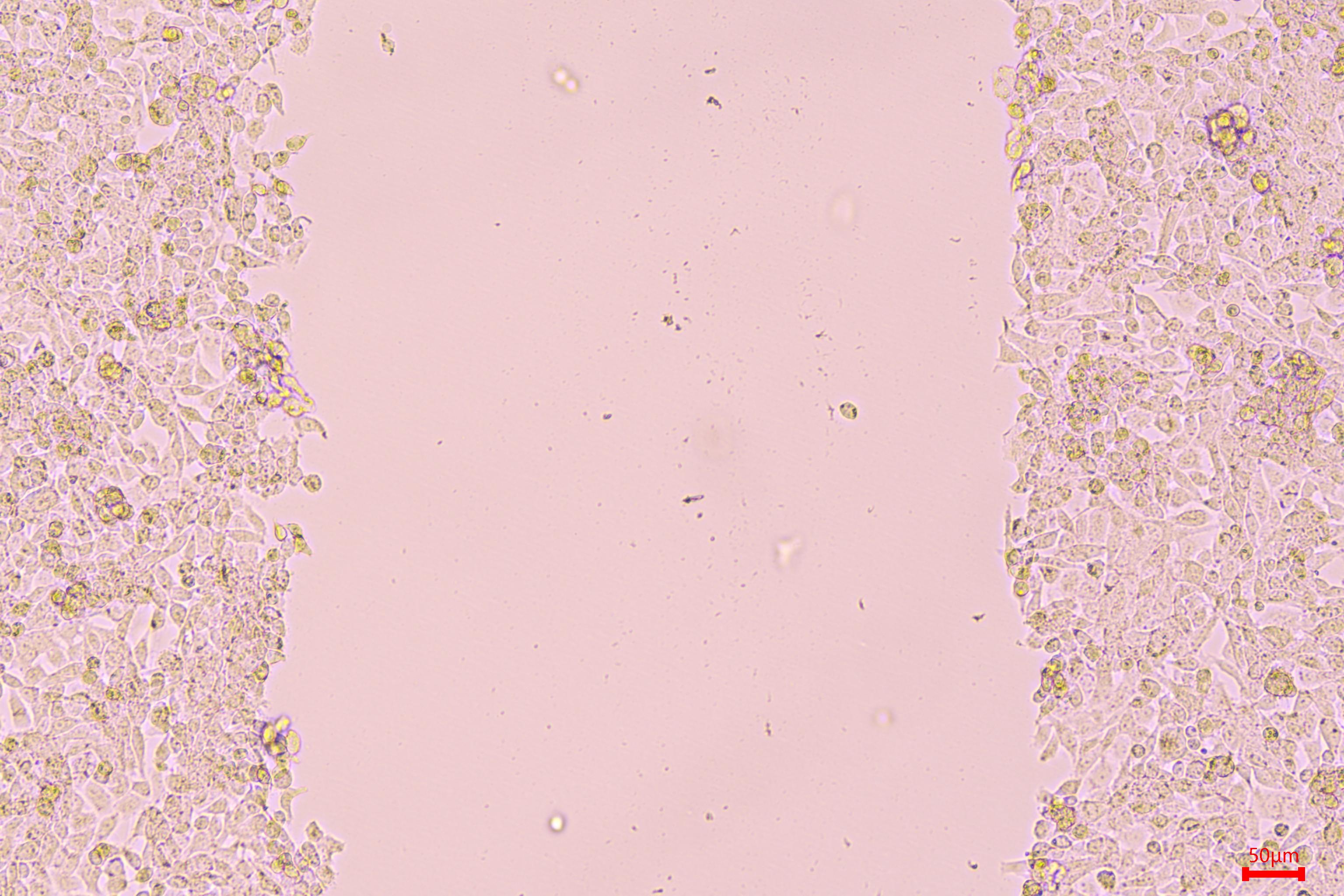

Supplement: Supplemental Information 8 [file peerj-11-14608-s008.zip › Figure 6 image/B/pcDNA3.1/0h (3).jpg]

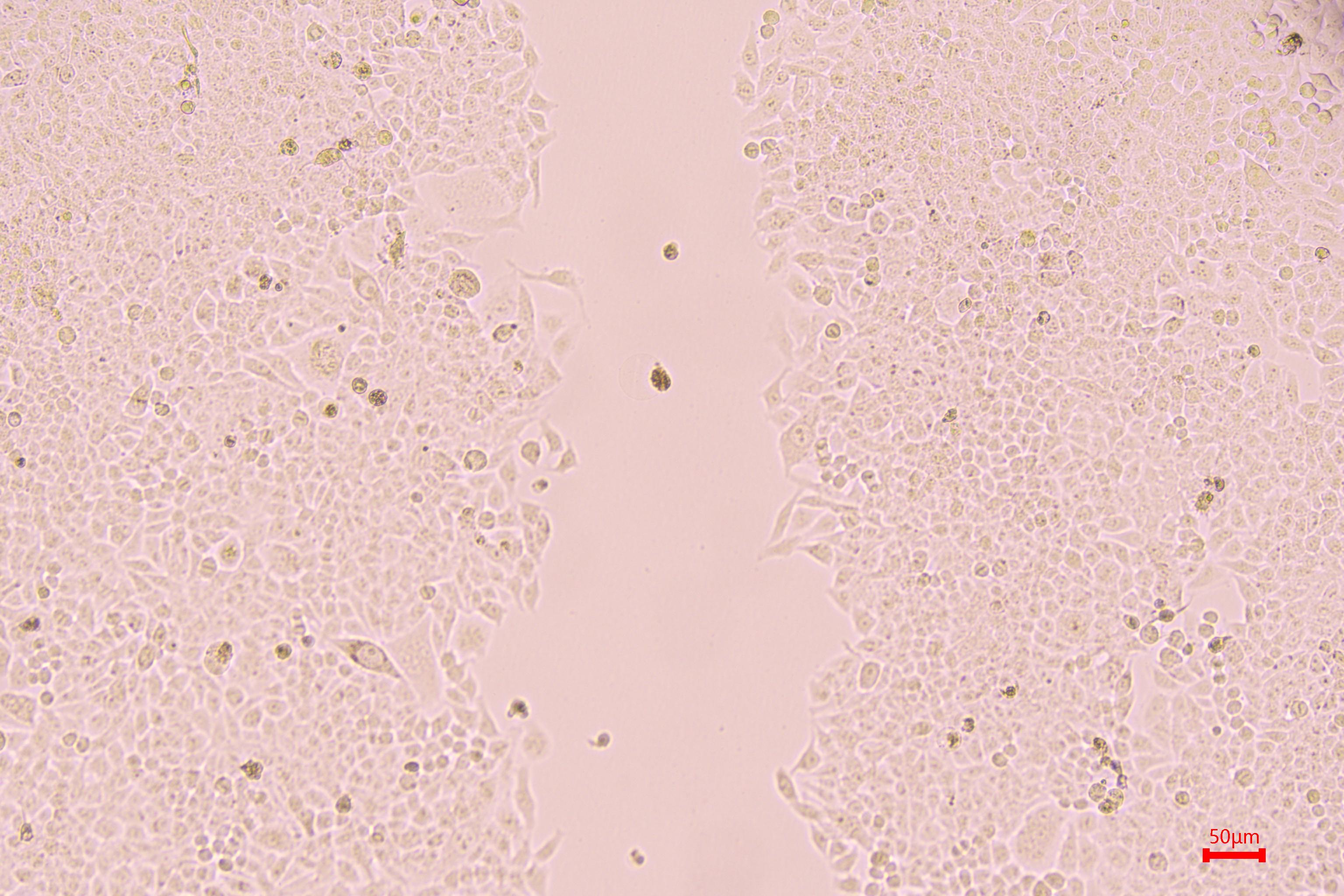

Supplement: Supplemental Information 8 [file peerj-11-14608-s008.zip › Figure 6 image/B/pcDNA3.1/48h (1).jpg]

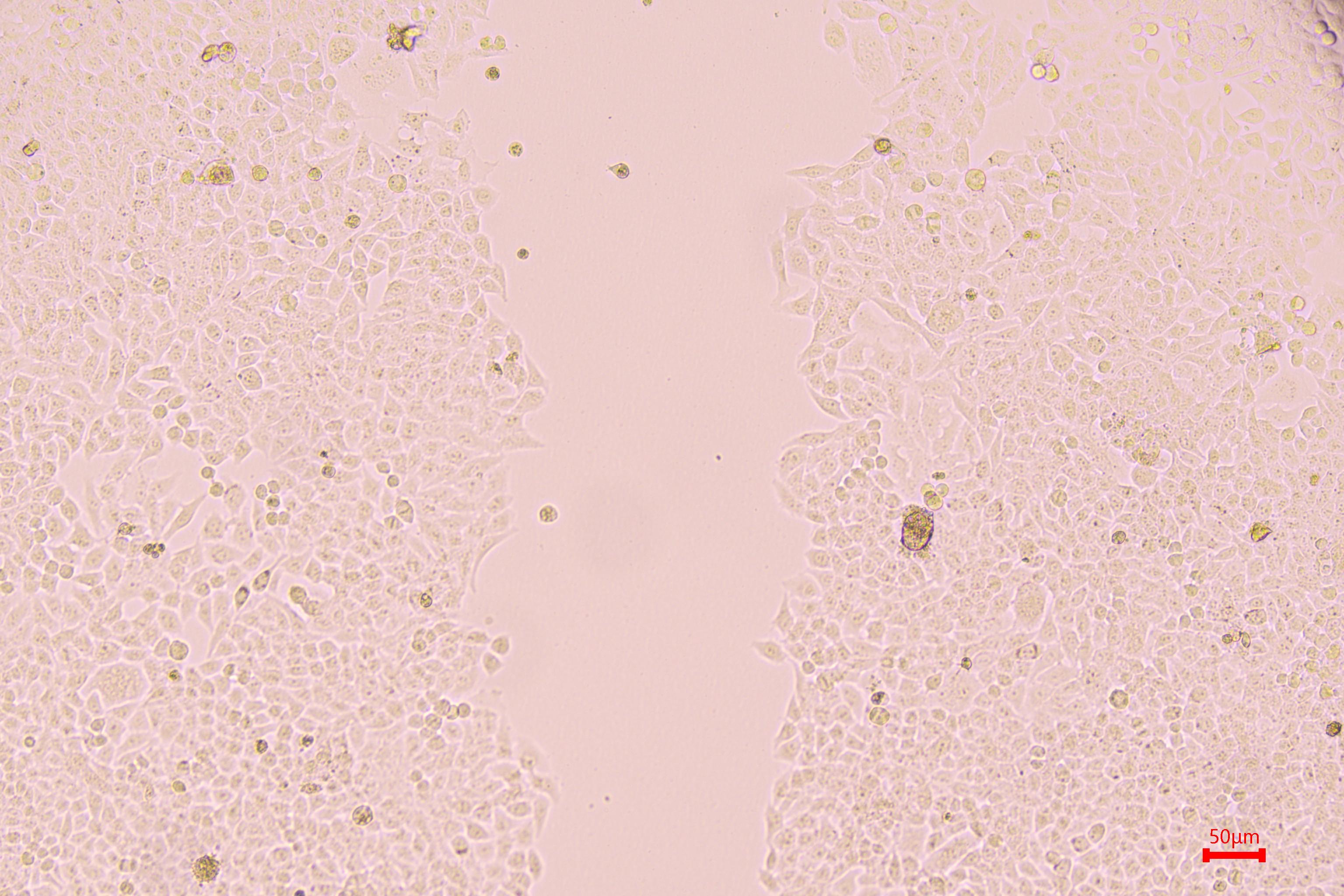

Supplement: Supplemental Information 8 [file peerj-11-14608-s008.zip › Figure 6 image/B/pcDNA3.1/48h (2).jpg]

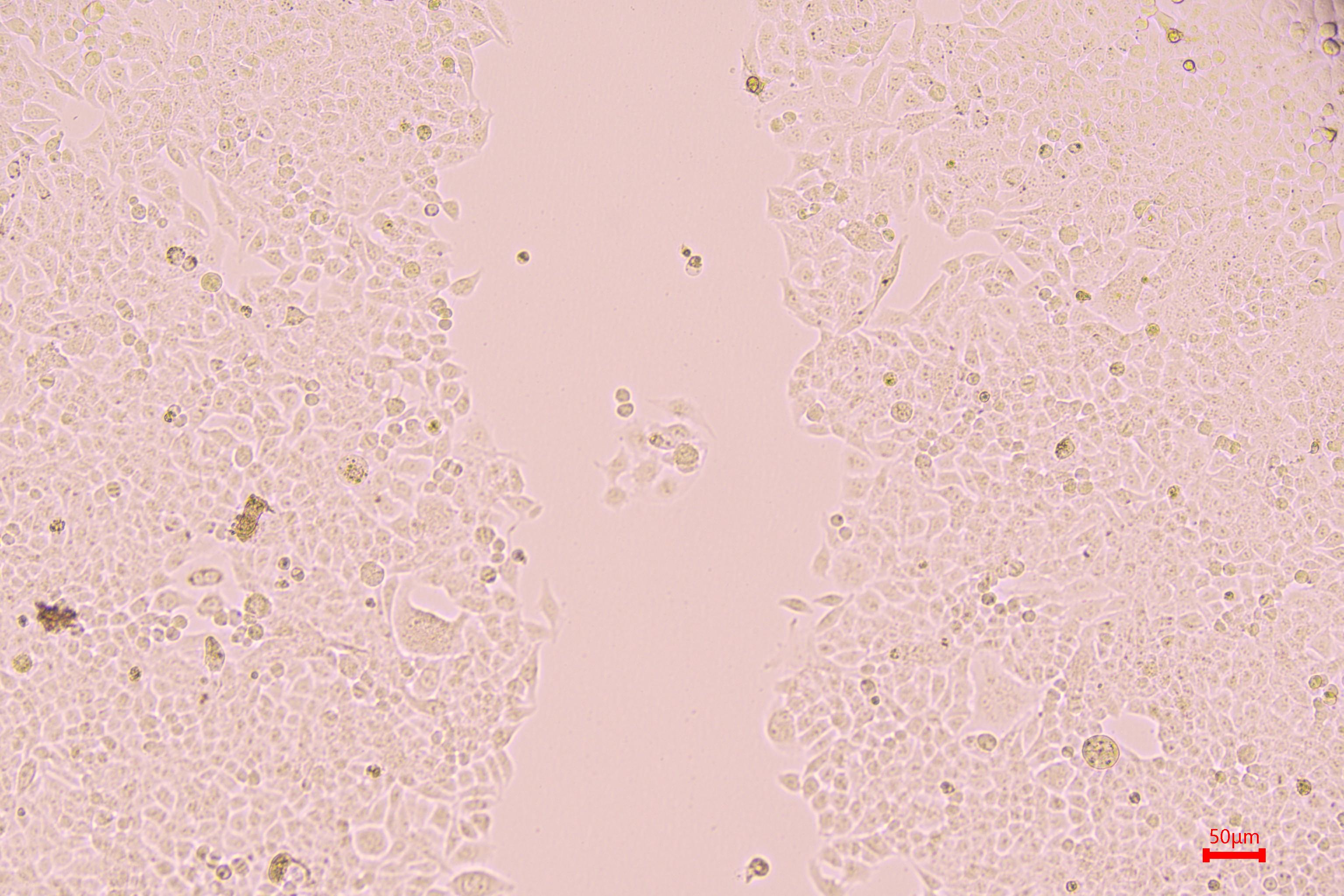

Supplement: Supplemental Information 8 [file peerj-11-14608-s008.zip › Figure 6 image/B/pcDNA3.1/48h (3).jpg]

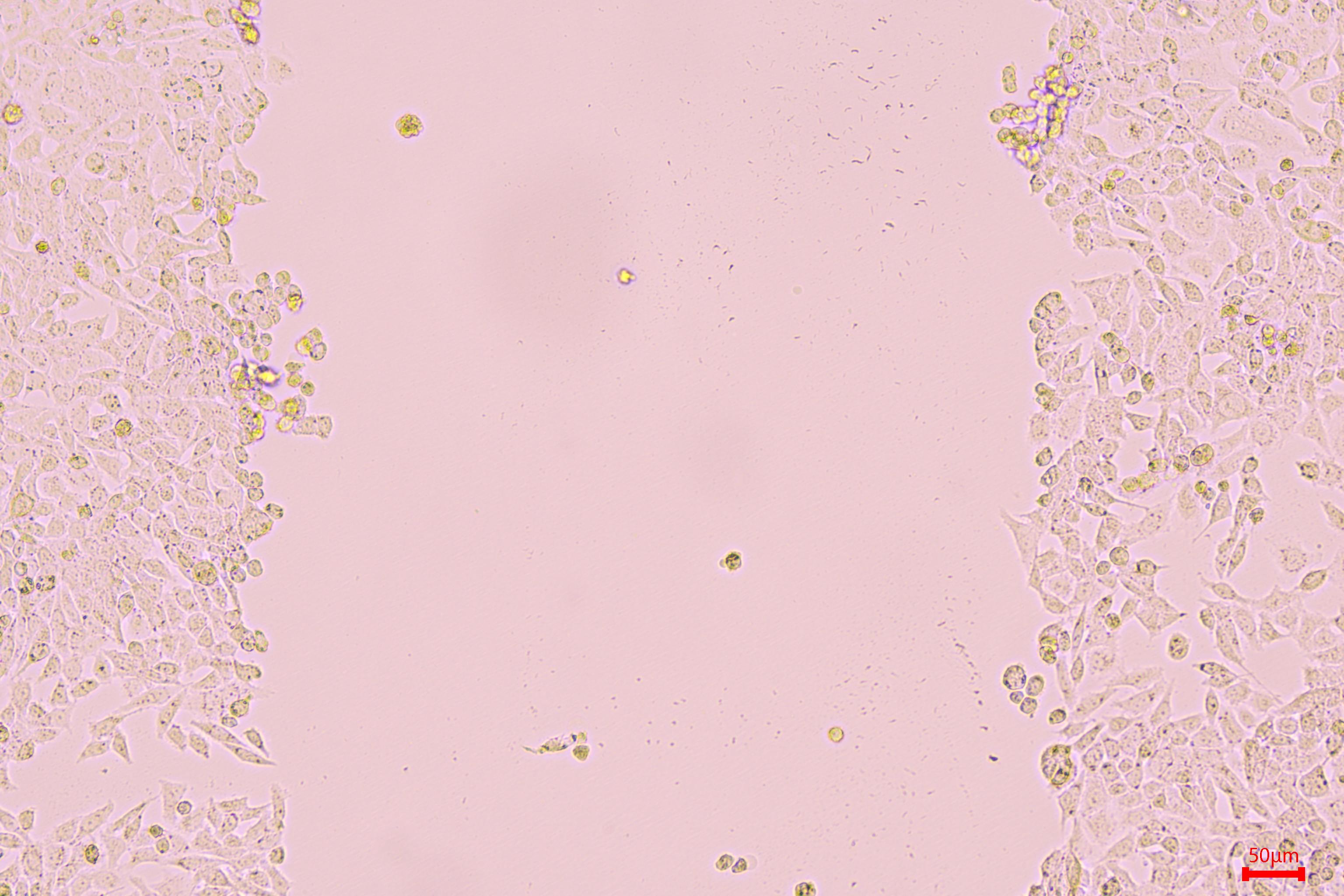

Supplement: Supplemental Information 8 [file peerj-11-14608-s008.zip › Figure 6 image/B/pcDNA3.1-GNG5/0h (1).jpg]

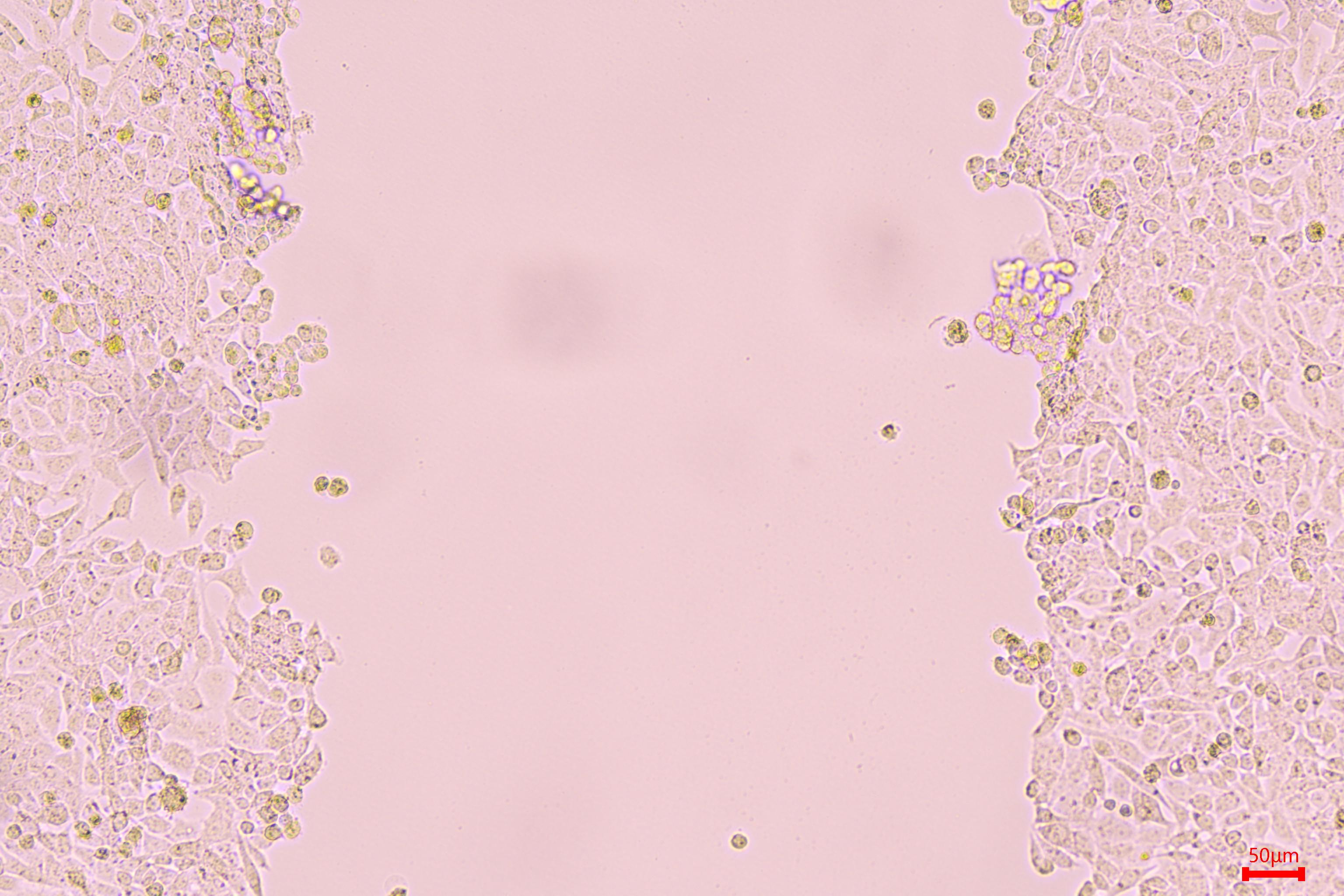

Supplement: Supplemental Information 8 [file peerj-11-14608-s008.zip › Figure 6 image/B/pcDNA3.1-GNG5/0h (2).jpg]

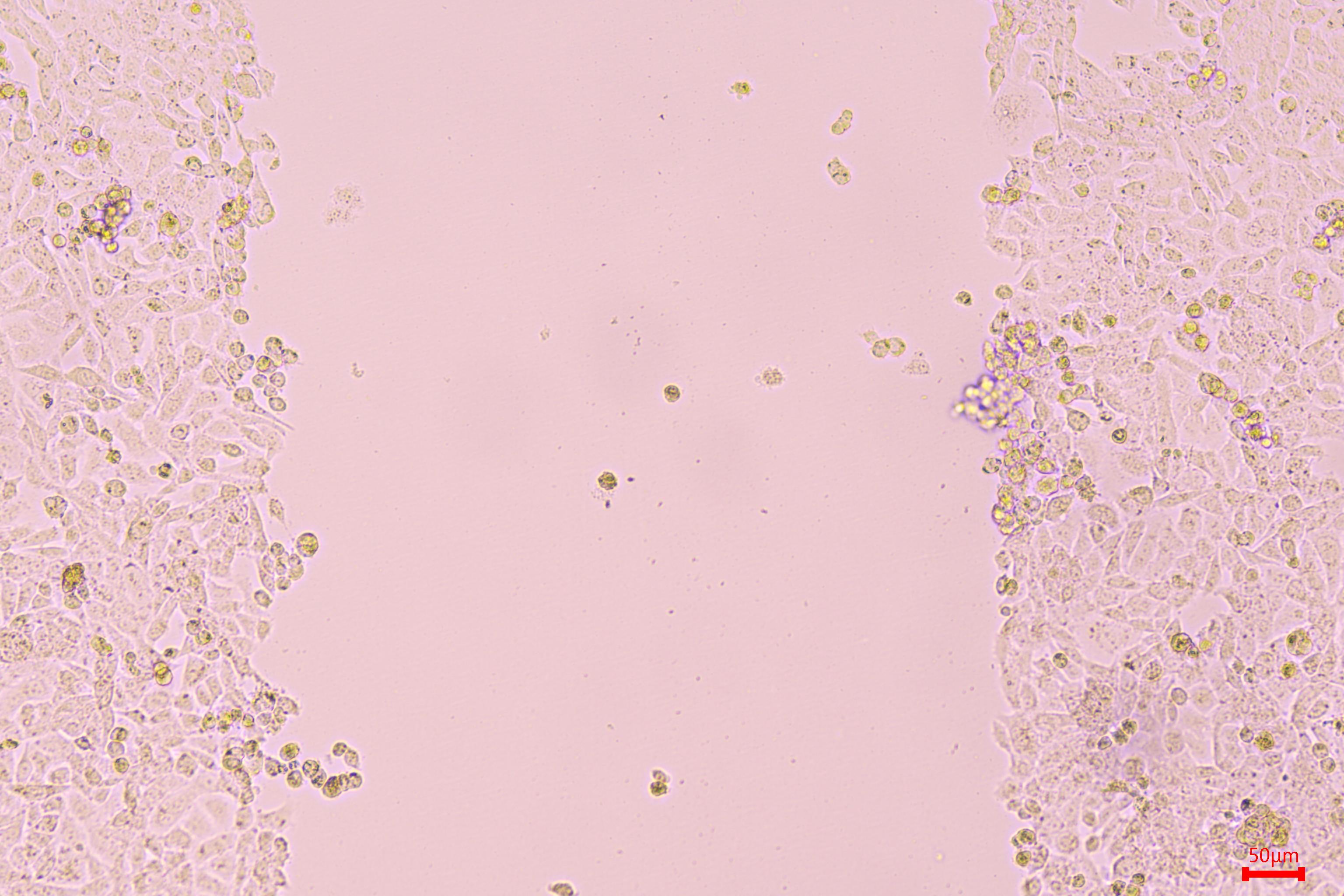

Supplement: Supplemental Information 8 [file peerj-11-14608-s008.zip › Figure 6 image/B/pcDNA3.1-GNG5/0h (3).jpg]

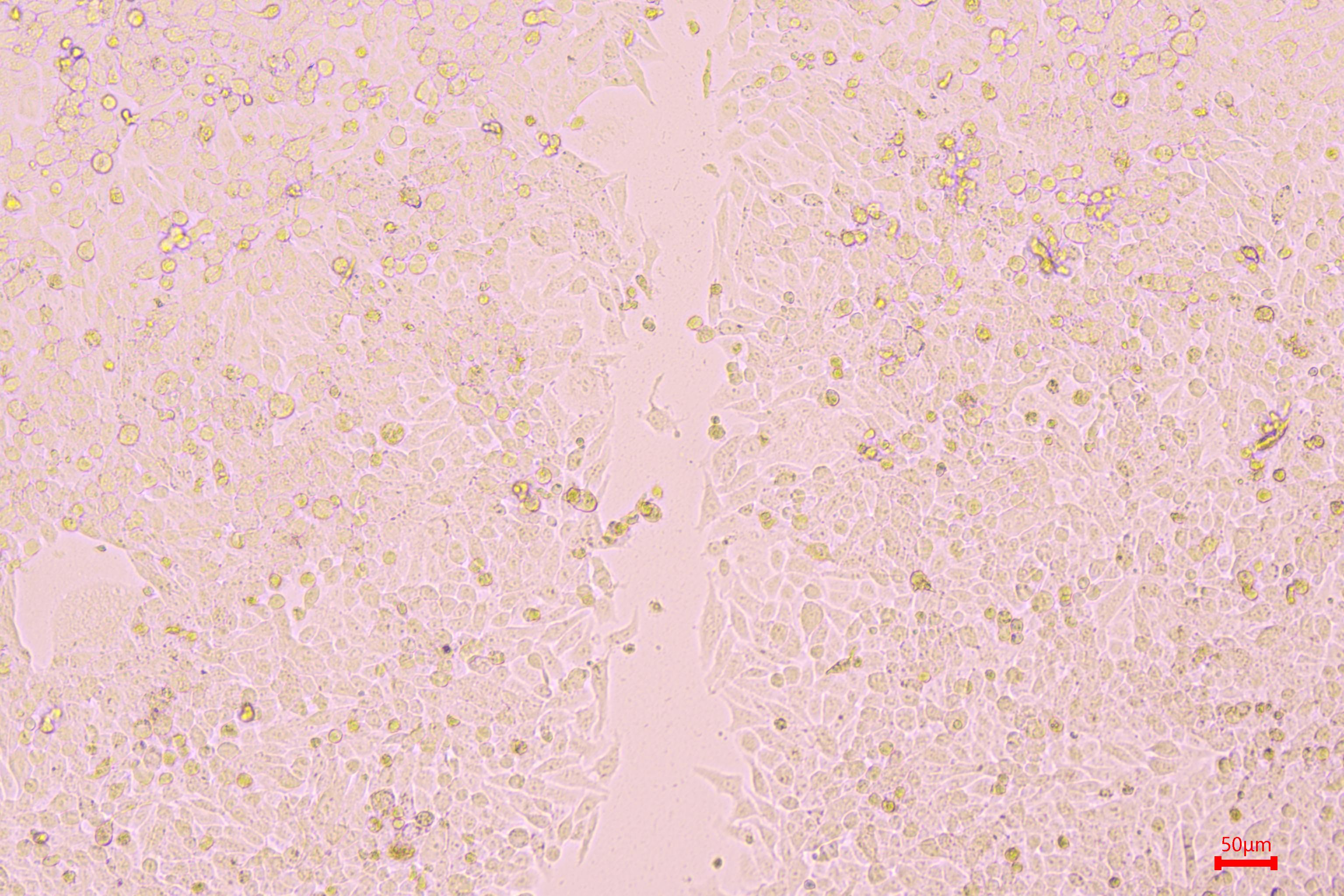

Supplement: Supplemental Information 8 [file peerj-11-14608-s008.zip › Figure 6 image/B/pcDNA3.1-GNG5/48h (1).jpg]

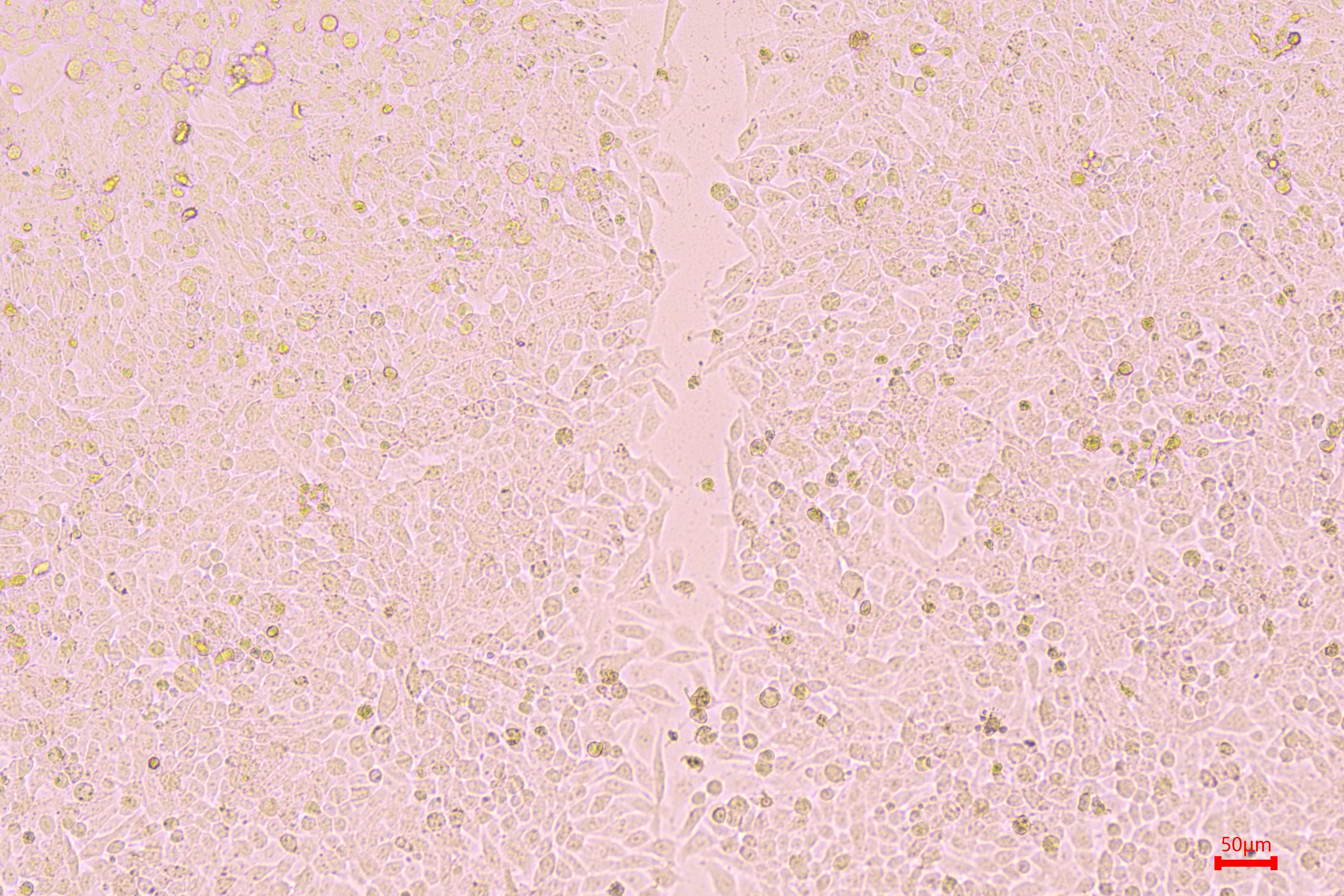

Supplement: Supplemental Information 8 [file peerj-11-14608-s008.zip › Figure 6 image/B/pcDNA3.1-GNG5/48h (2).jpg]

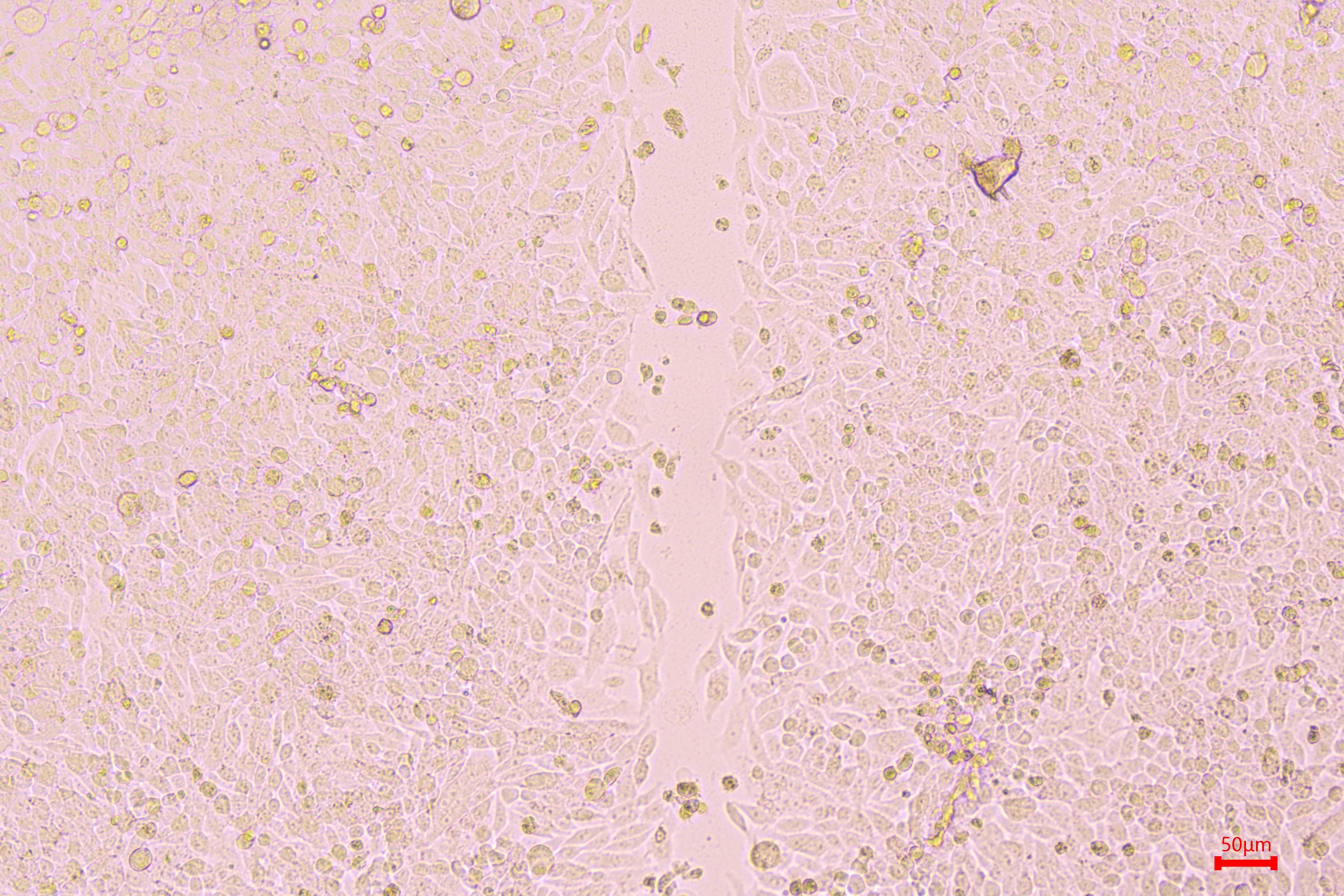

Supplement: Supplemental Information 8 [file peerj-11-14608-s008.zip › Figure 6 image/B/pcDNA3.1-GNG5/48h (3).jpg]

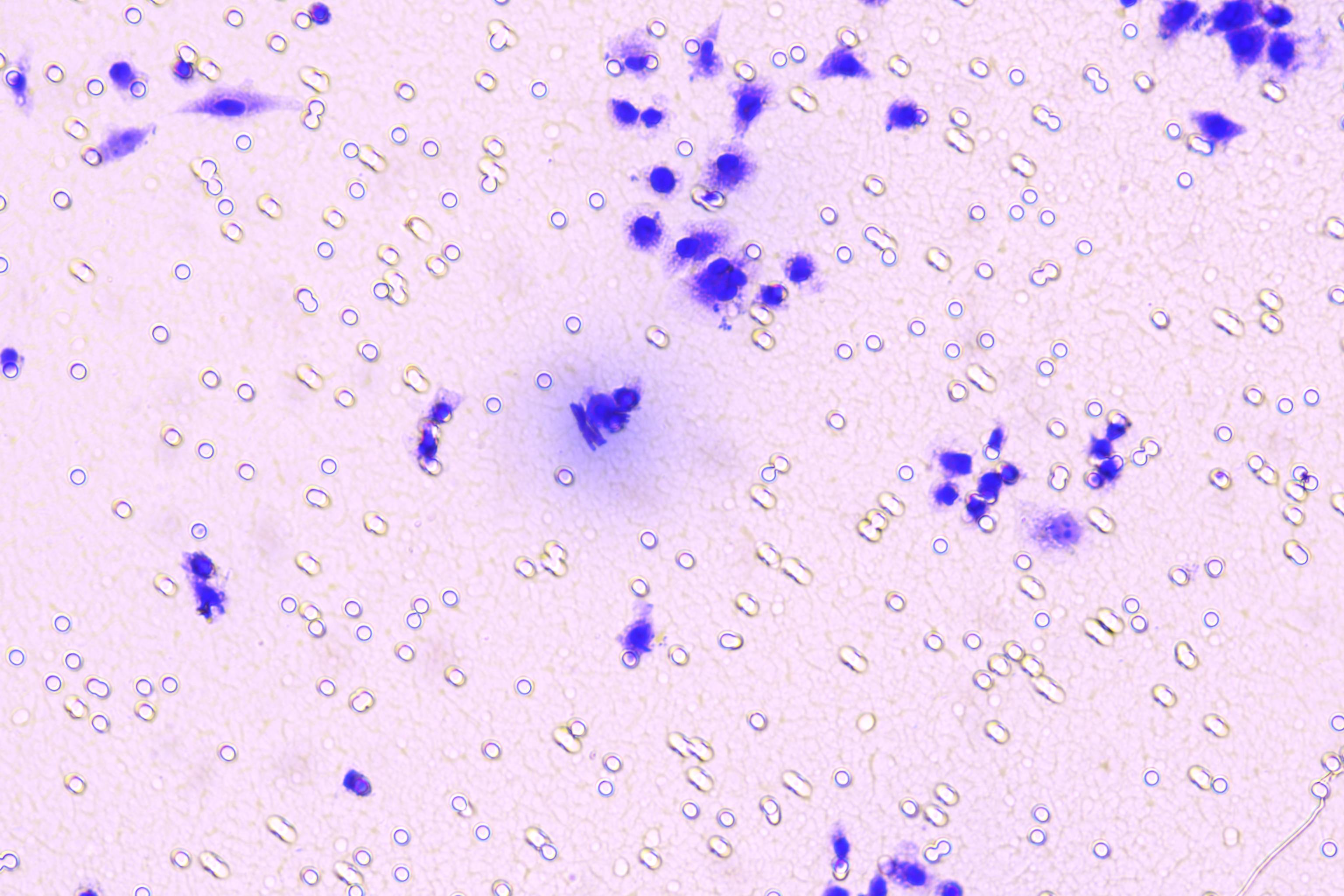

Supplement: Supplemental Information 8 [file peerj-11-14608-s008.zip › Figure 6 image/C/ASO-GNG5/1.jpg]

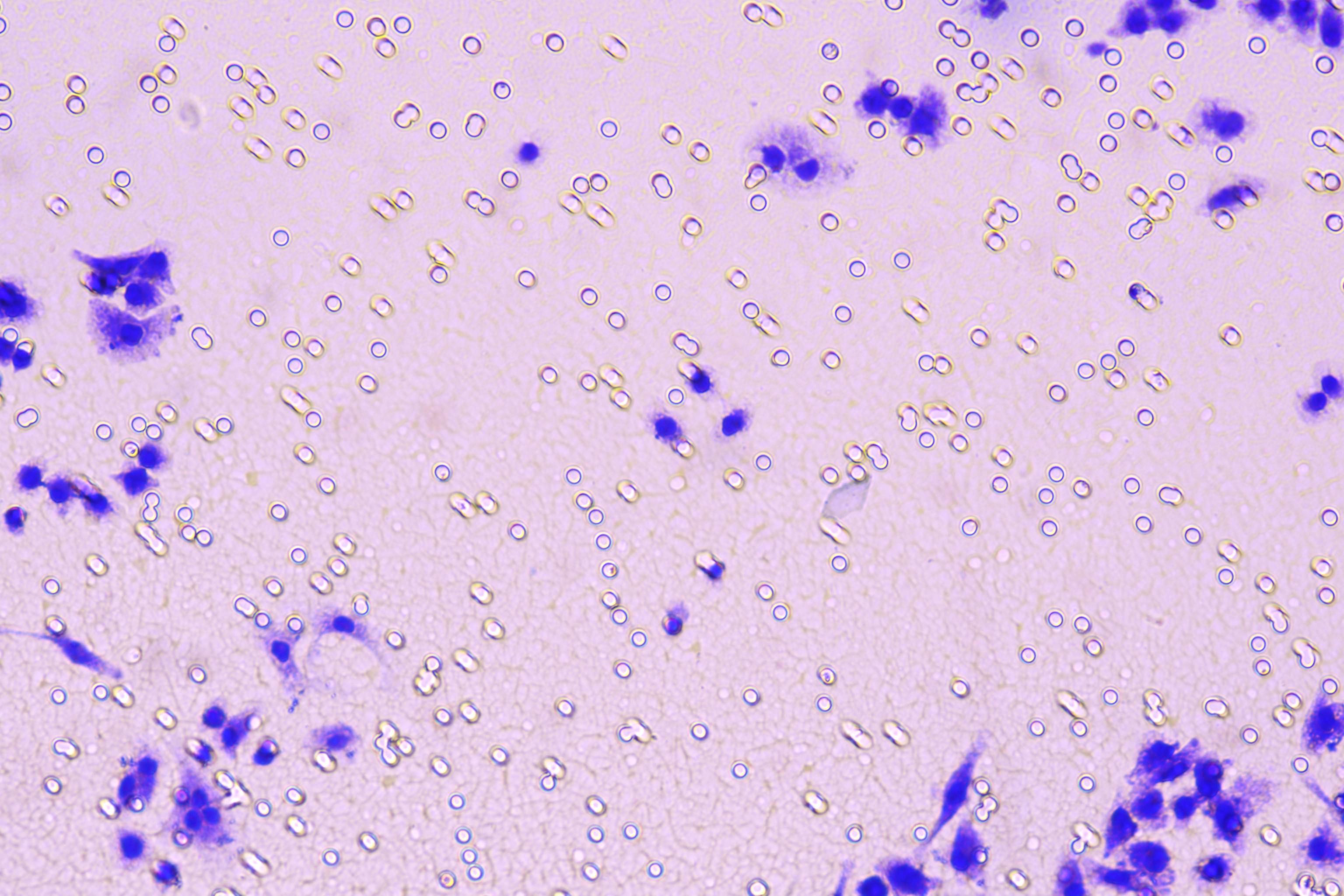

Supplement: Supplemental Information 8 [file peerj-11-14608-s008.zip › Figure 6 image/C/ASO-GNG5/2.jpg]

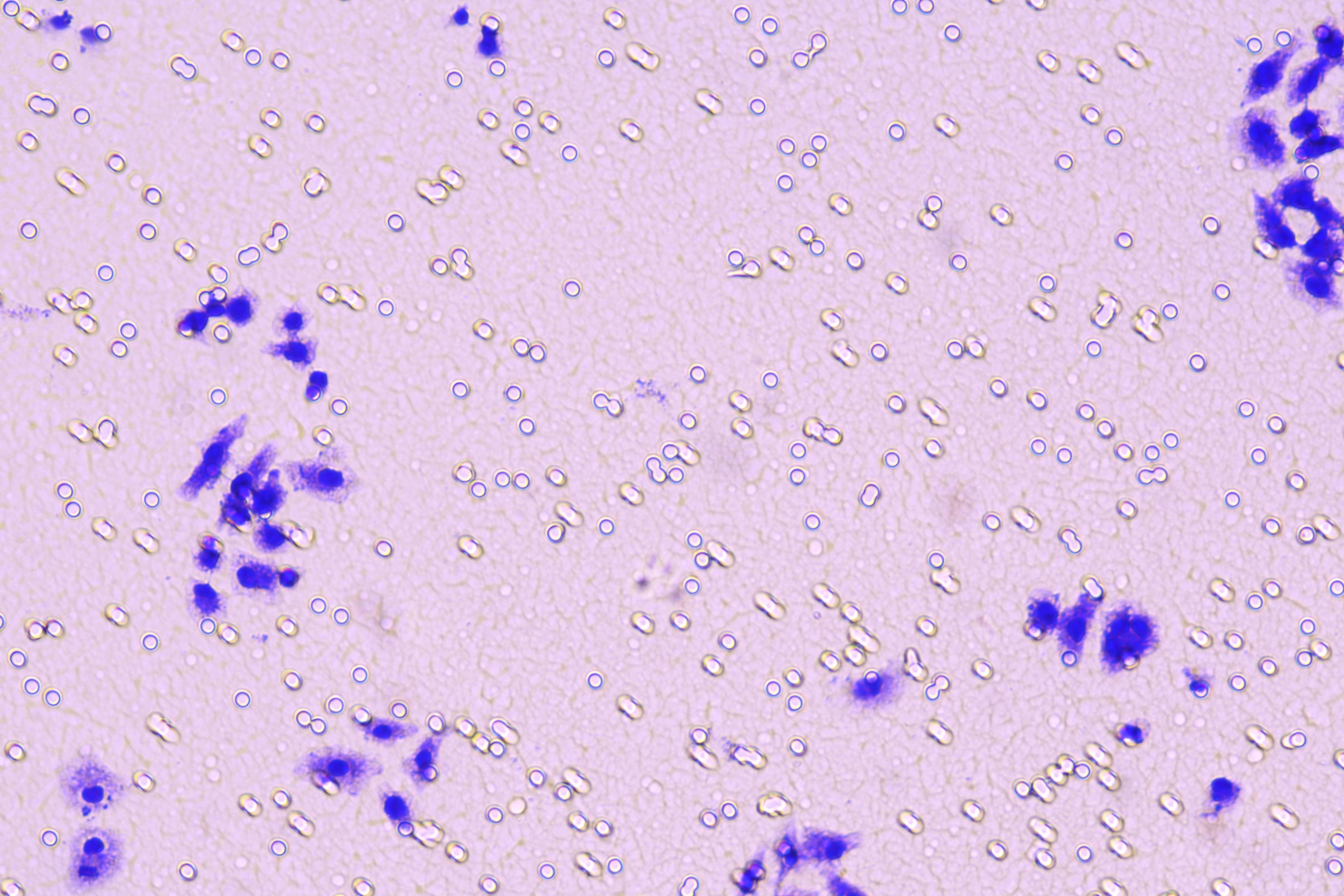

Supplement: Supplemental Information 8 [file peerj-11-14608-s008.zip › Figure 6 image/C/ASO-GNG5/3.jpg]

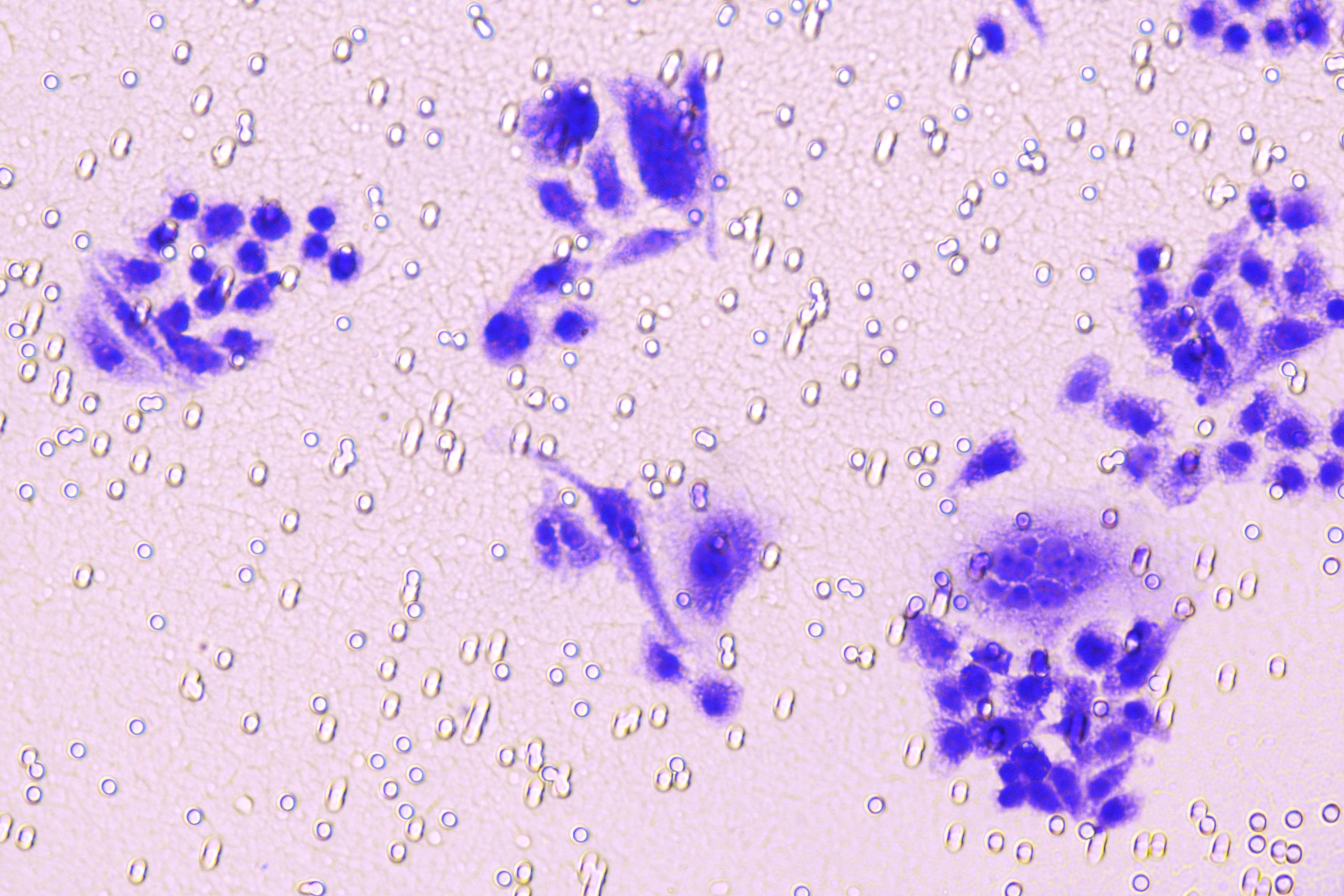

Supplement: Supplemental Information 8 [file peerj-11-14608-s008.zip › Figure 6 image/C/ASO-NC/1.jpg]

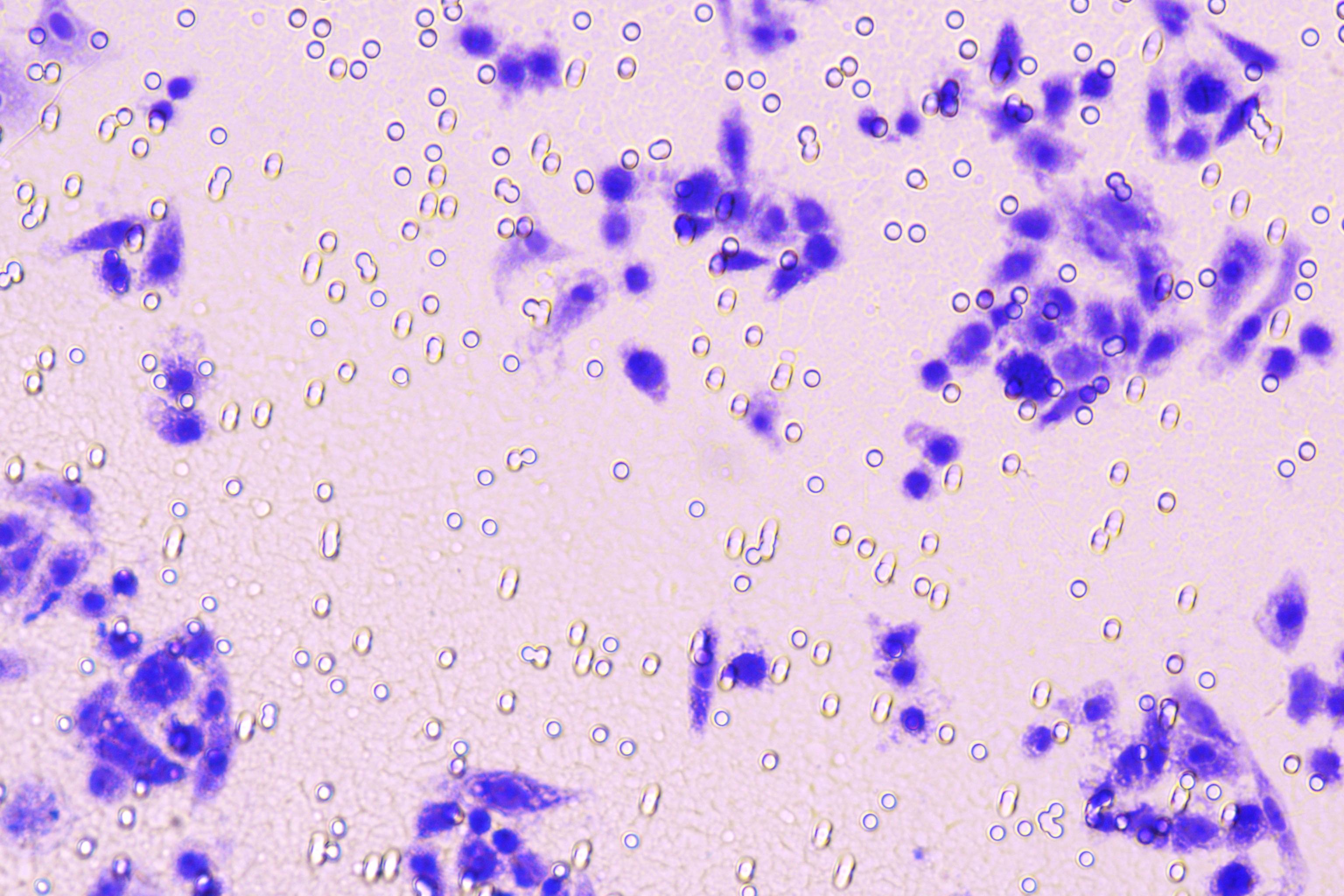

Supplement: Supplemental Information 8 [file peerj-11-14608-s008.zip › Figure 6 image/C/ASO-NC/2.jpg]

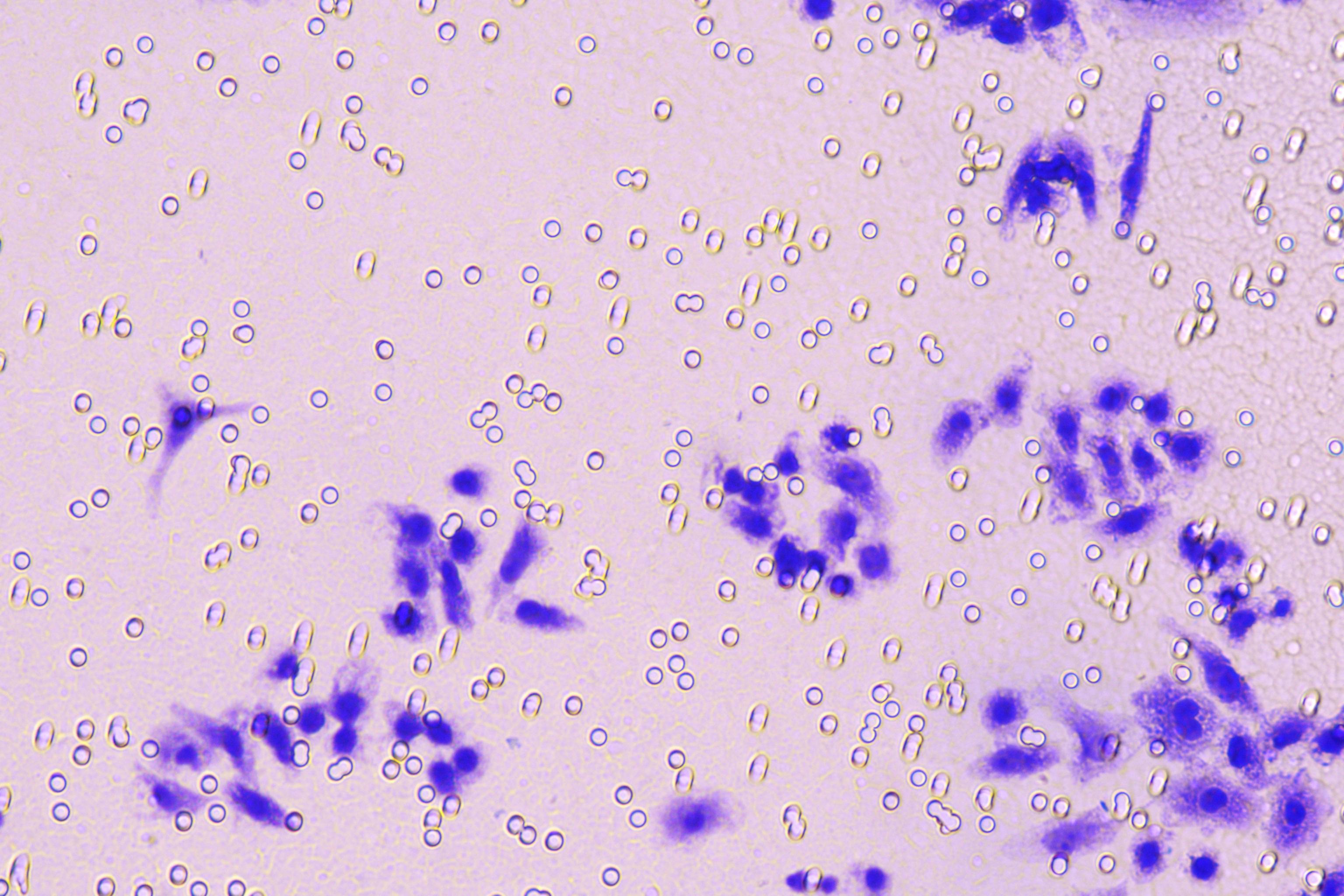

Supplement: Supplemental Information 8 [file peerj-11-14608-s008.zip › Figure 6 image/C/ASO-NC/3.jpg]

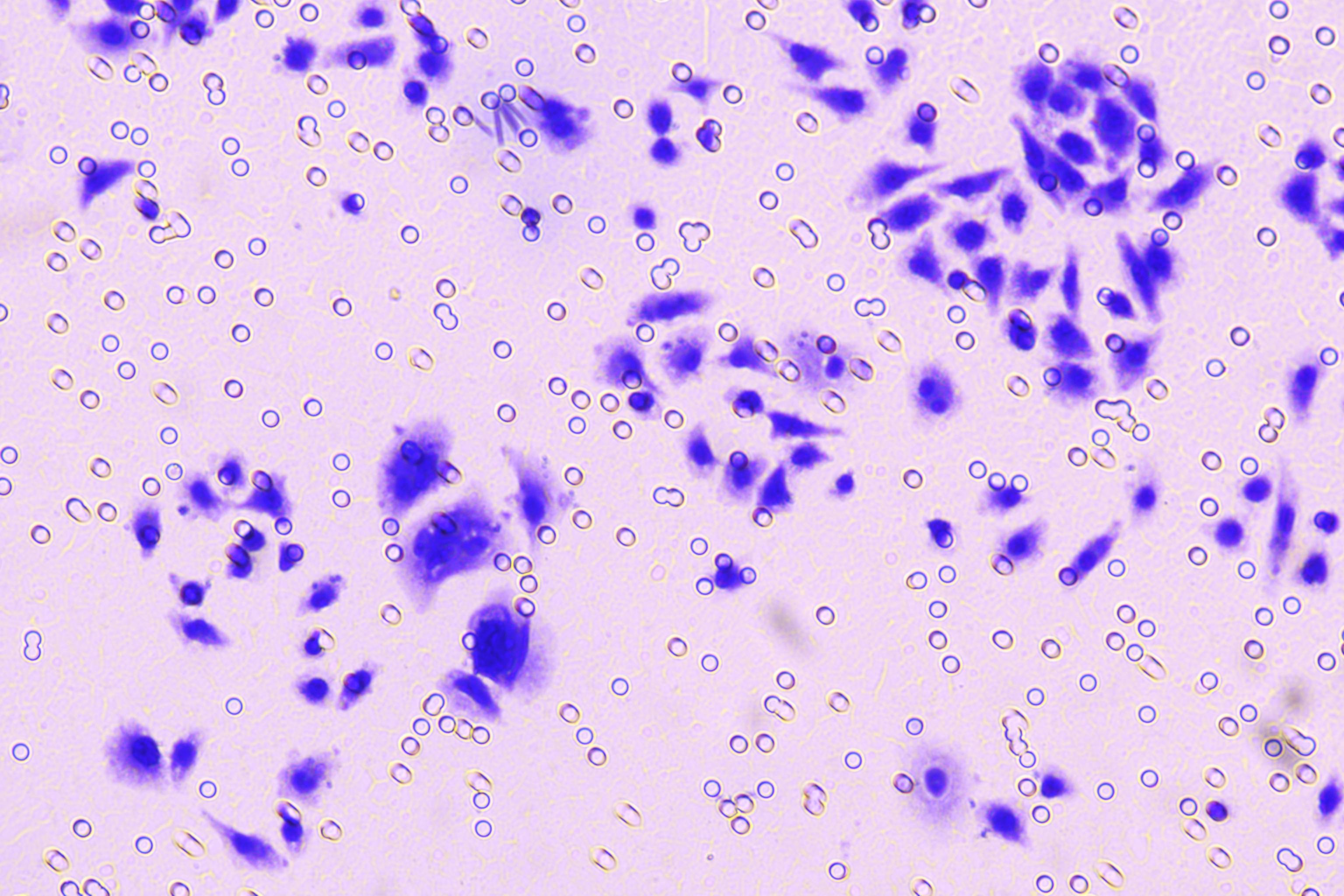

Supplement: Supplemental Information 8 [file peerj-11-14608-s008.zip › Figure 6 image/C/pcDNA3.1/1.jpg]

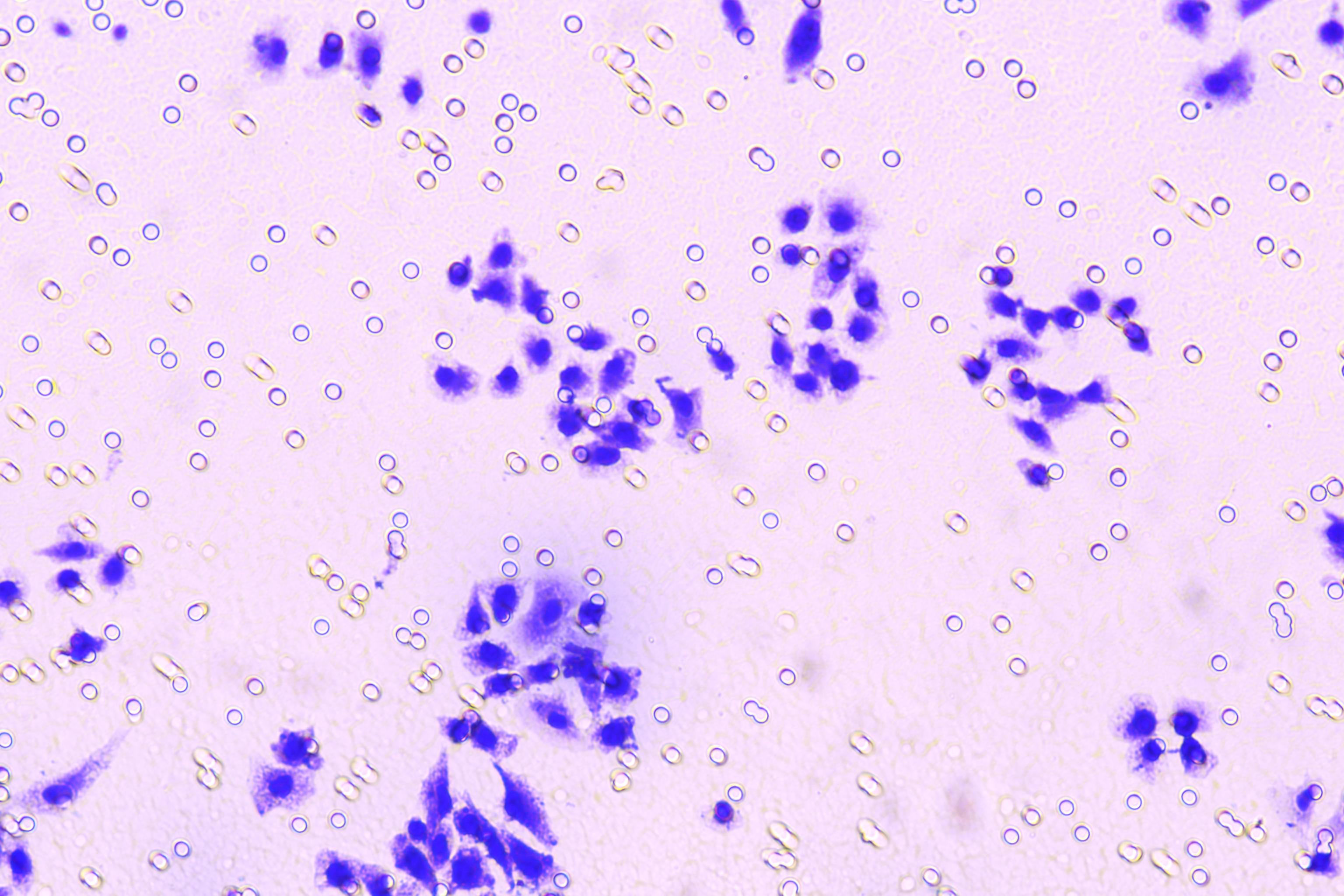

Supplement: Supplemental Information 8 [file peerj-11-14608-s008.zip › Figure 6 image/C/pcDNA3.1/2.jpg]

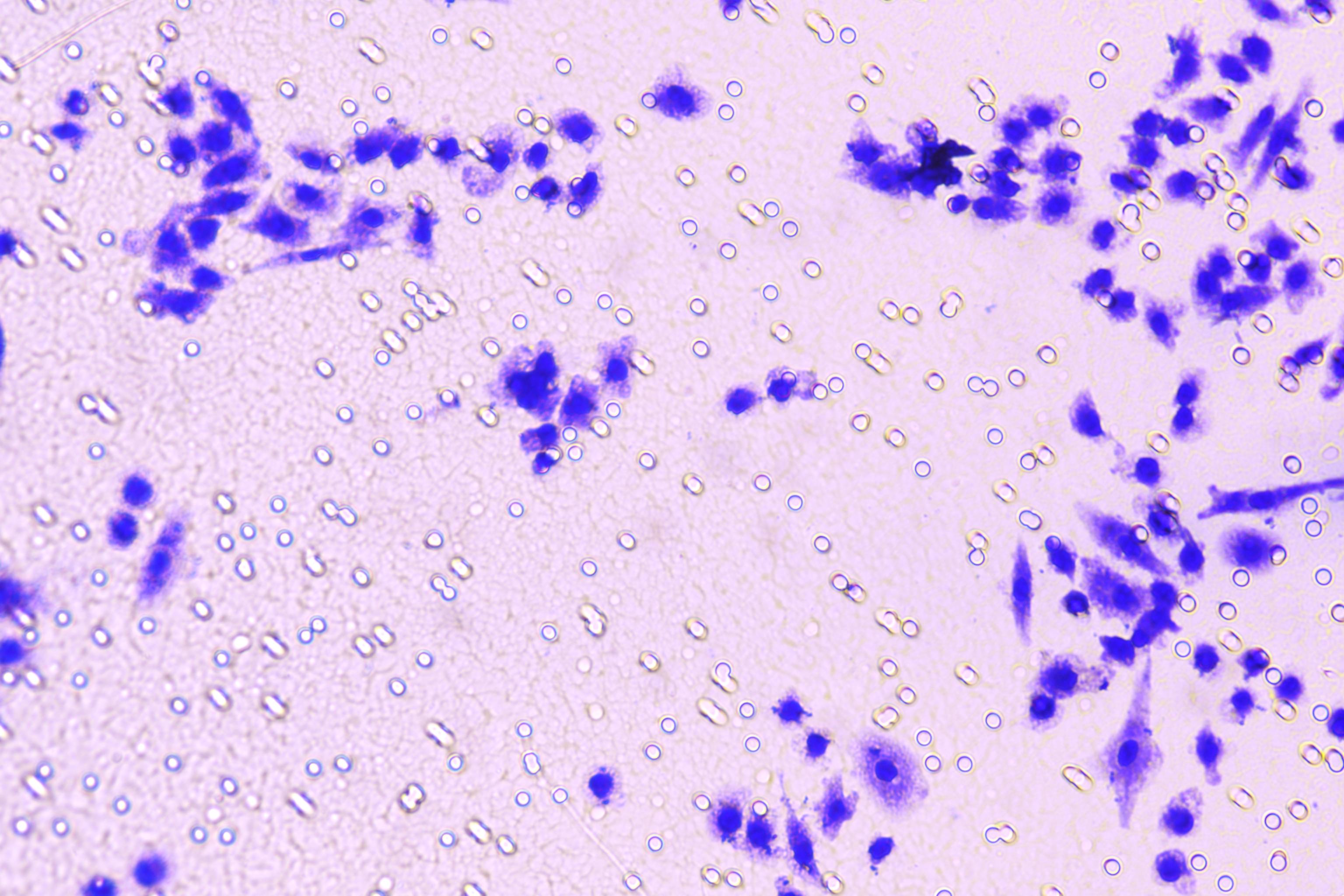

Supplement: Supplemental Information 8 [file peerj-11-14608-s008.zip › Figure 6 image/C/pcDNA3.1/3.jpg]

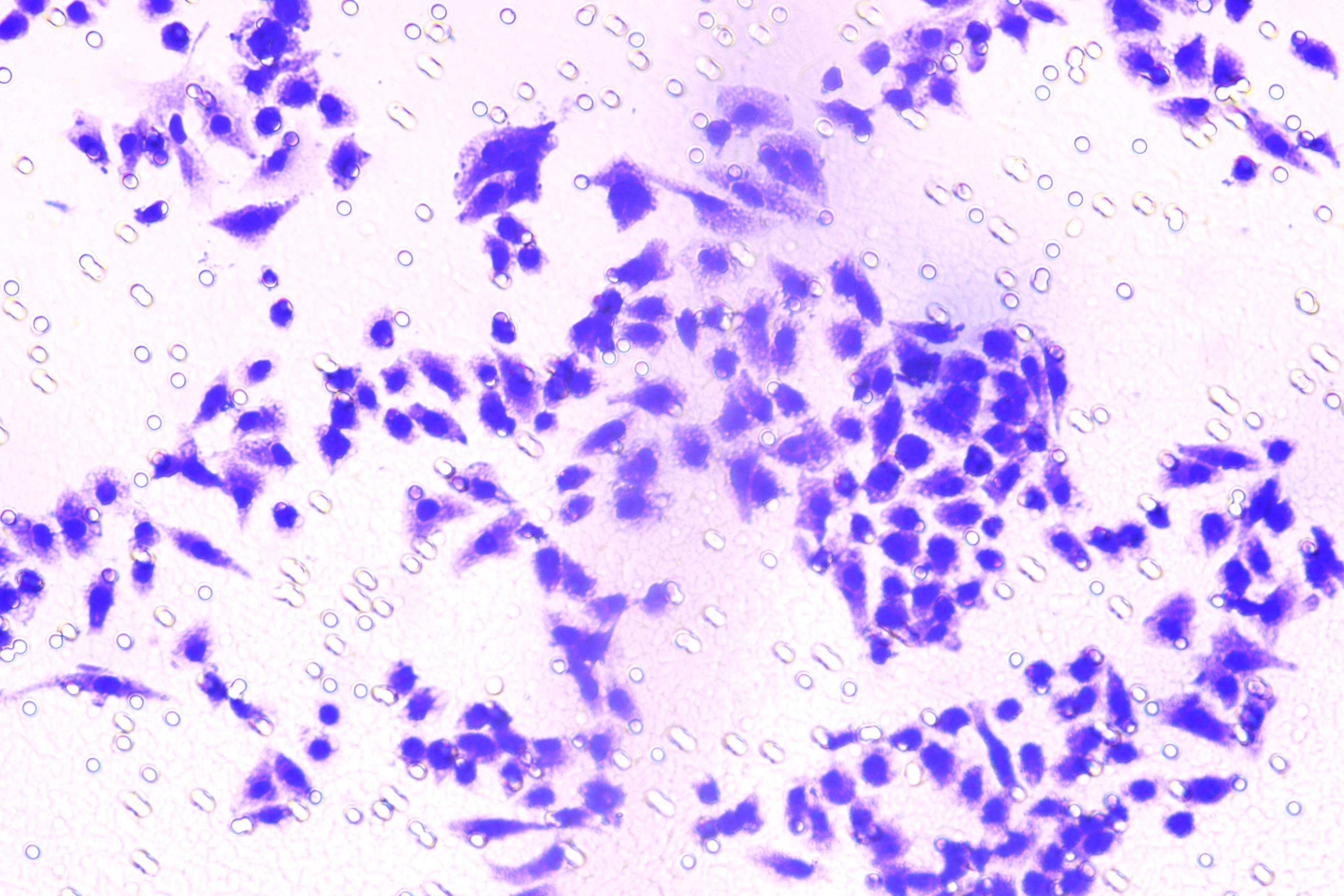

Supplement: Supplemental Information 8 [file peerj-11-14608-s008.zip › Figure 6 image/C/pcDNA3.1-GNG5/1.jpg]

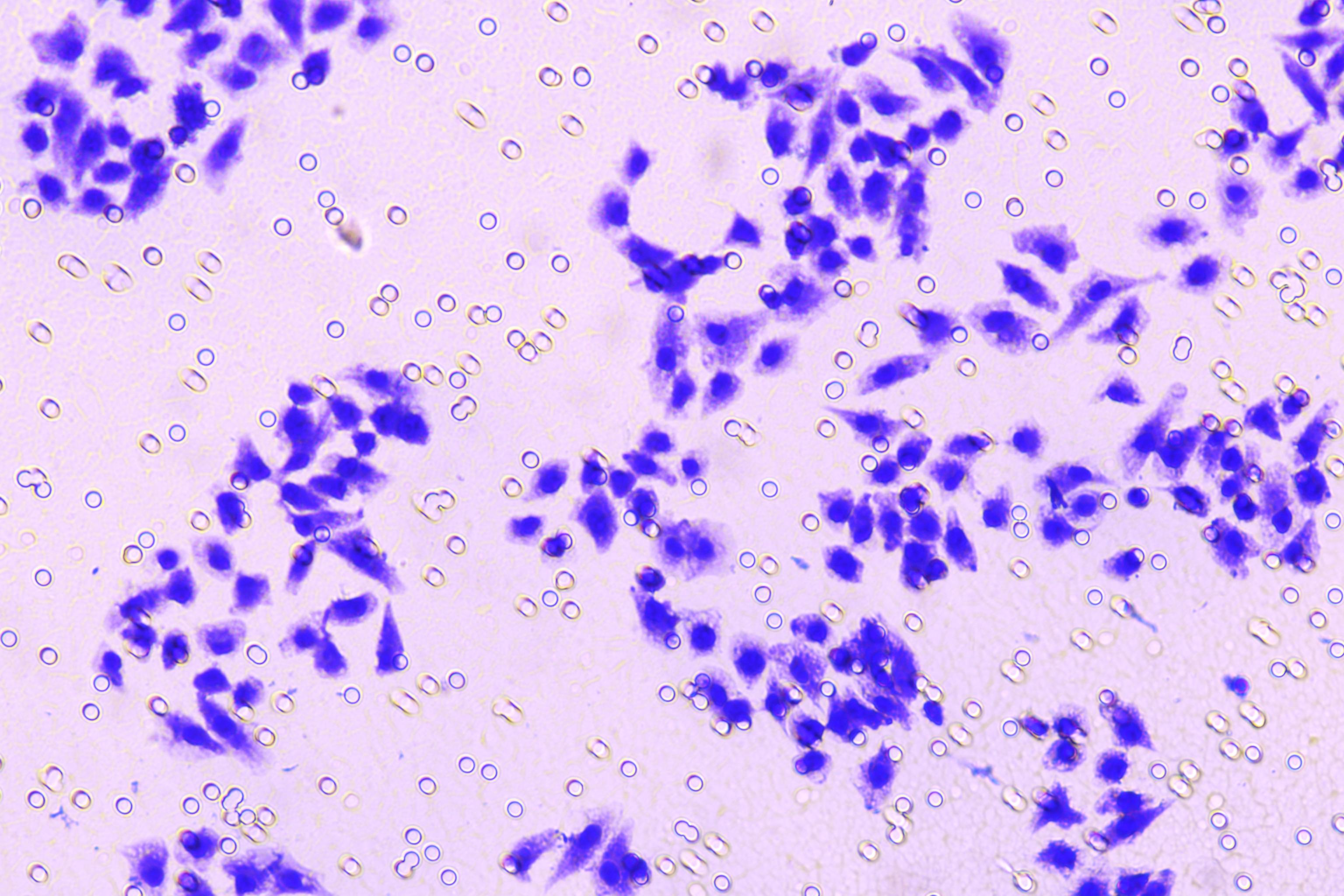

Supplement: Supplemental Information 8 [file peerj-11-14608-s008.zip › Figure 6 image/C/pcDNA3.1-GNG5/2.jpg]

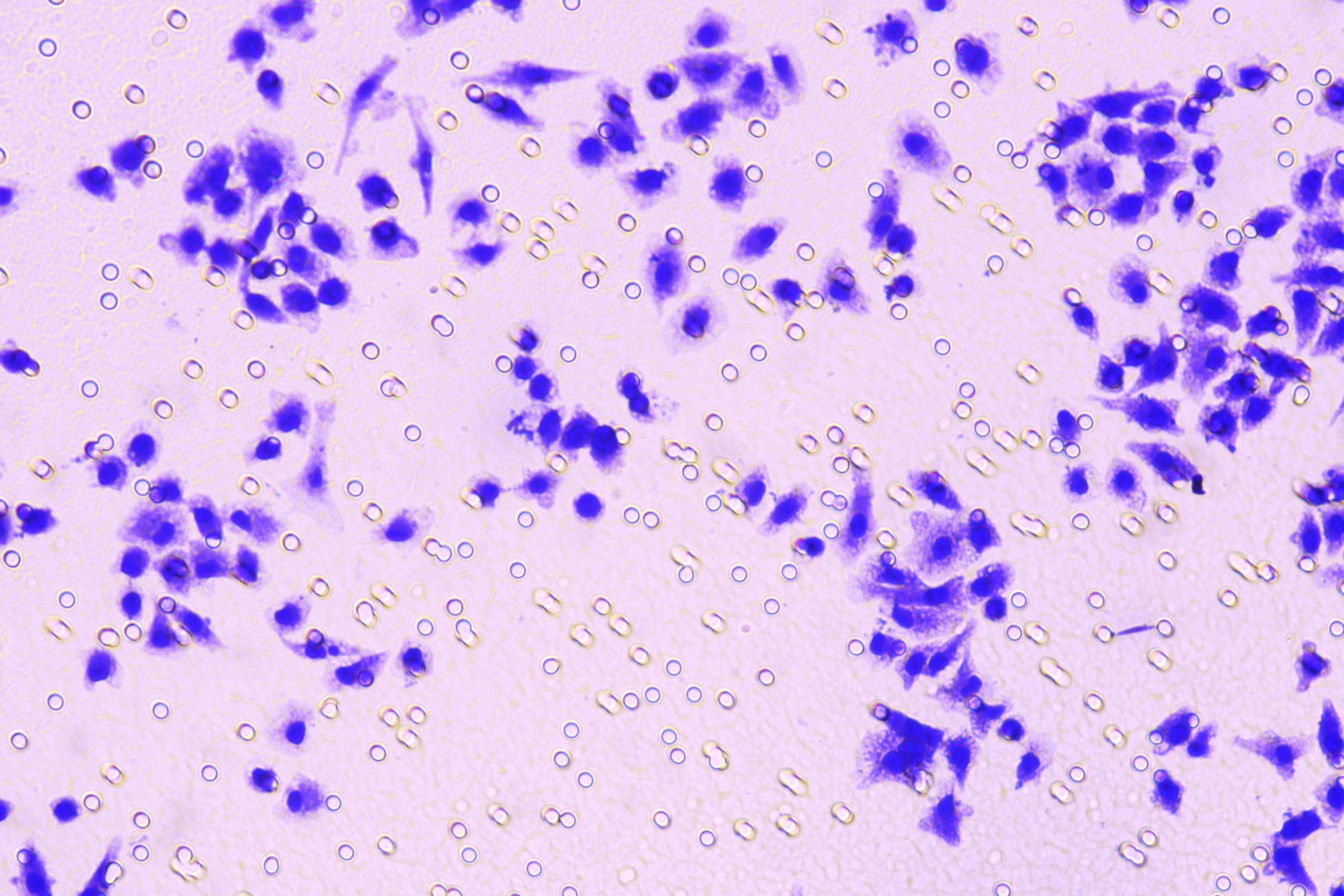

Supplement: Supplemental Information 8 [file peerj-11-14608-s008.zip › Figure 6 image/C/pcDNA3.1-GNG5/3.jpg]

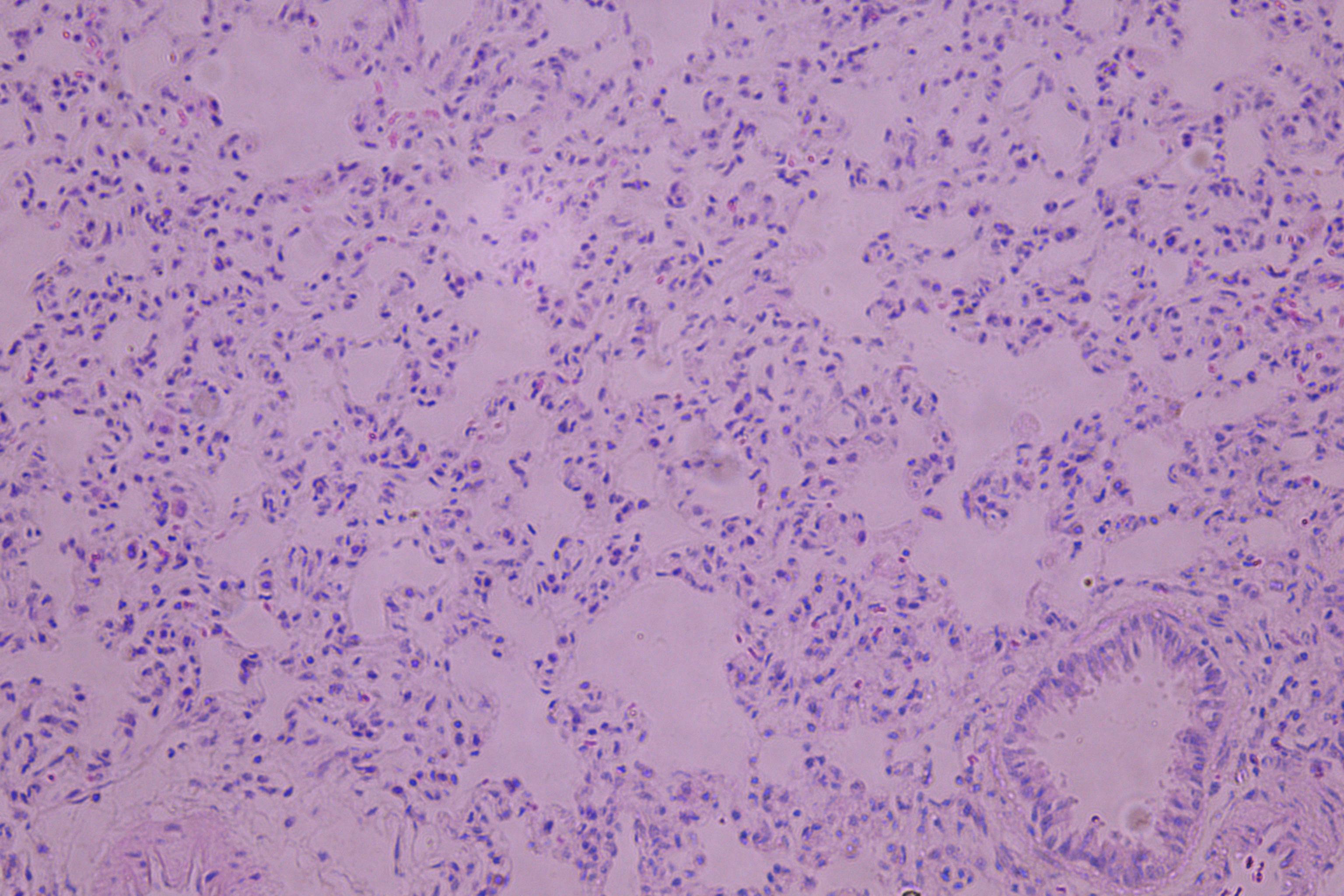

Supplement: Supplemental Information 9 [file peerj-11-14608-s009.zip › Figure 7 image/A/TUMOR/1.jpg]

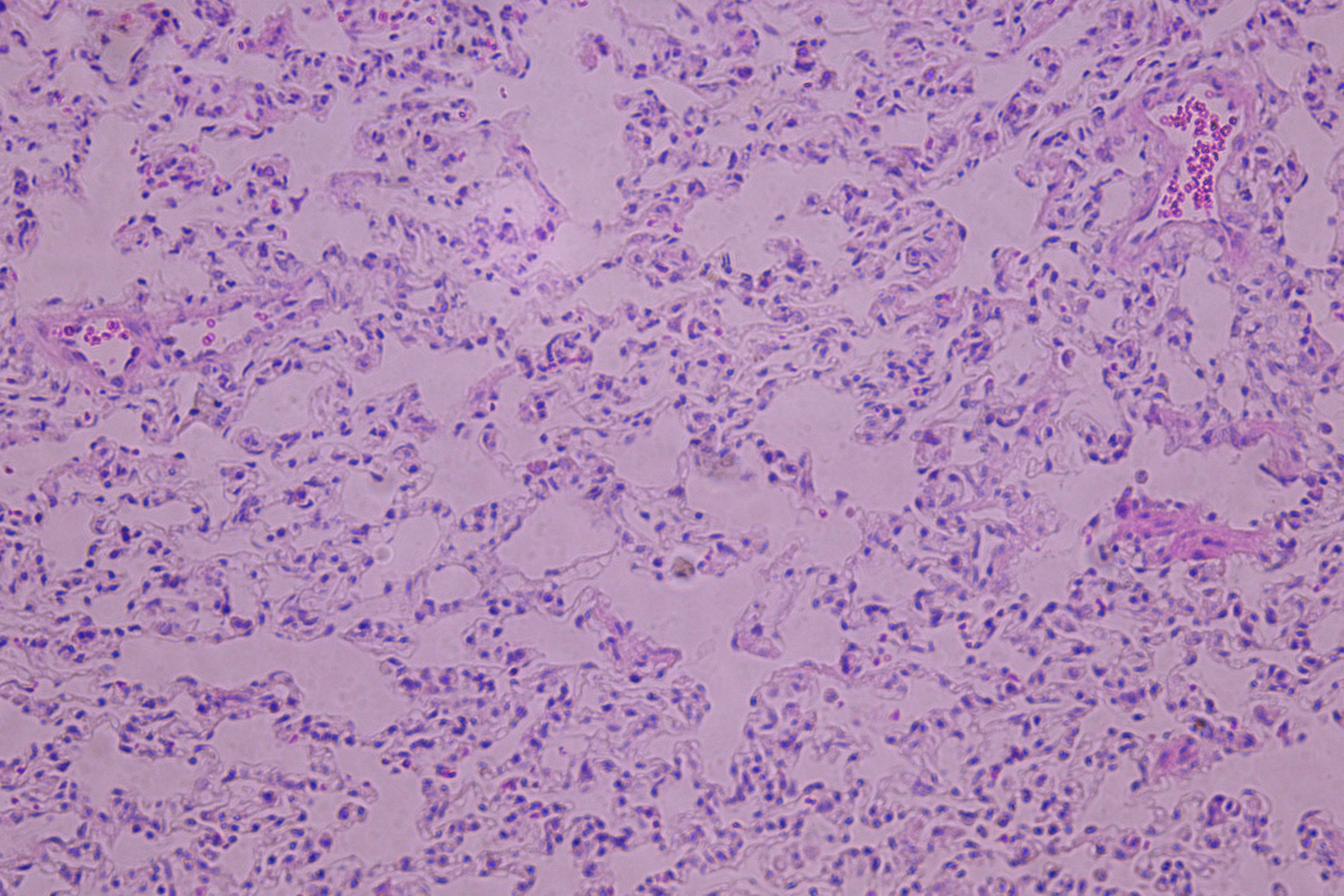

Supplement: Supplemental Information 9 [file peerj-11-14608-s009.zip › Figure 7 image/A/TUMOR/2.jpg]

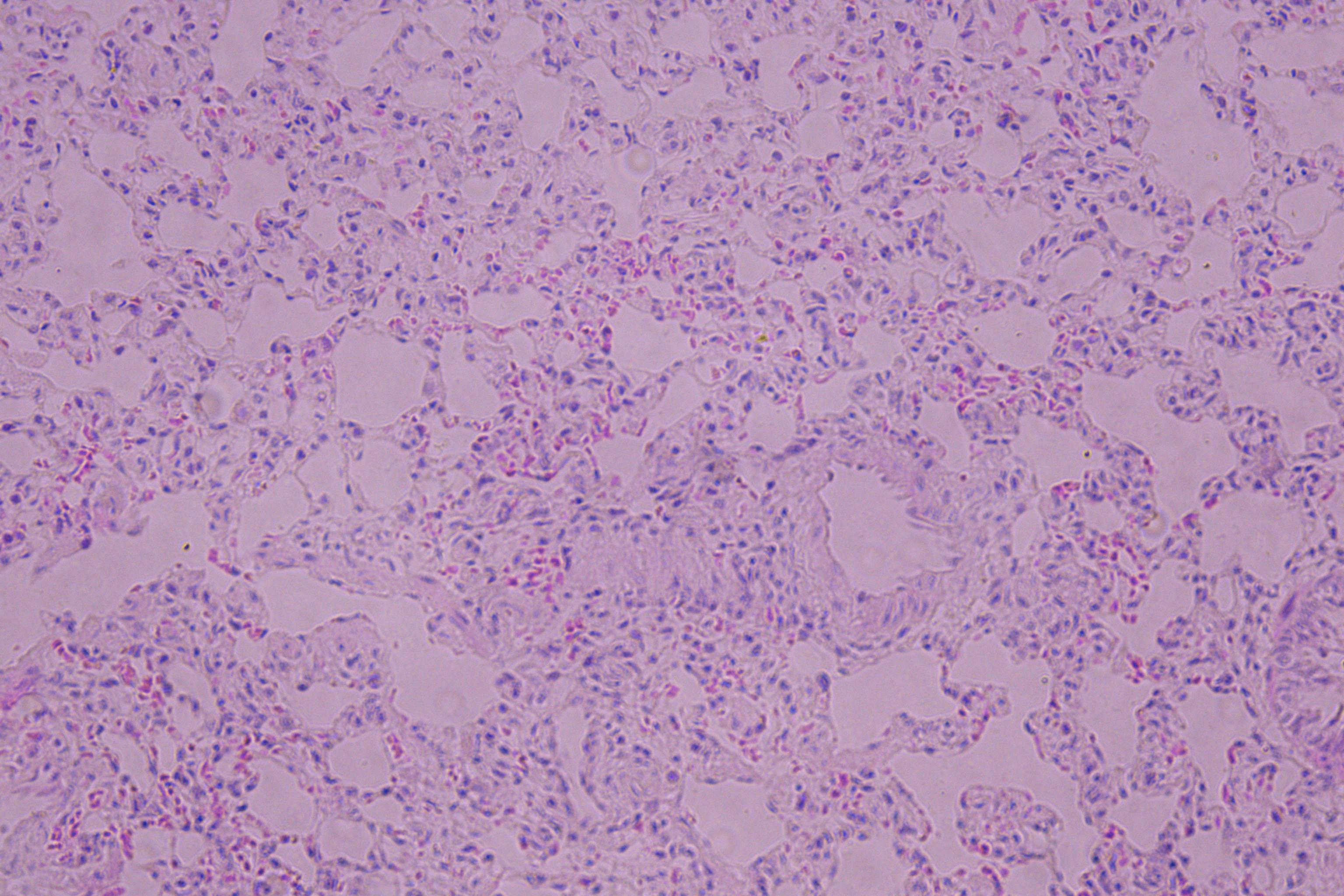

Supplement: Supplemental Information 9 [file peerj-11-14608-s009.zip › Figure 7 image/A/TUMOR/3.jpg]

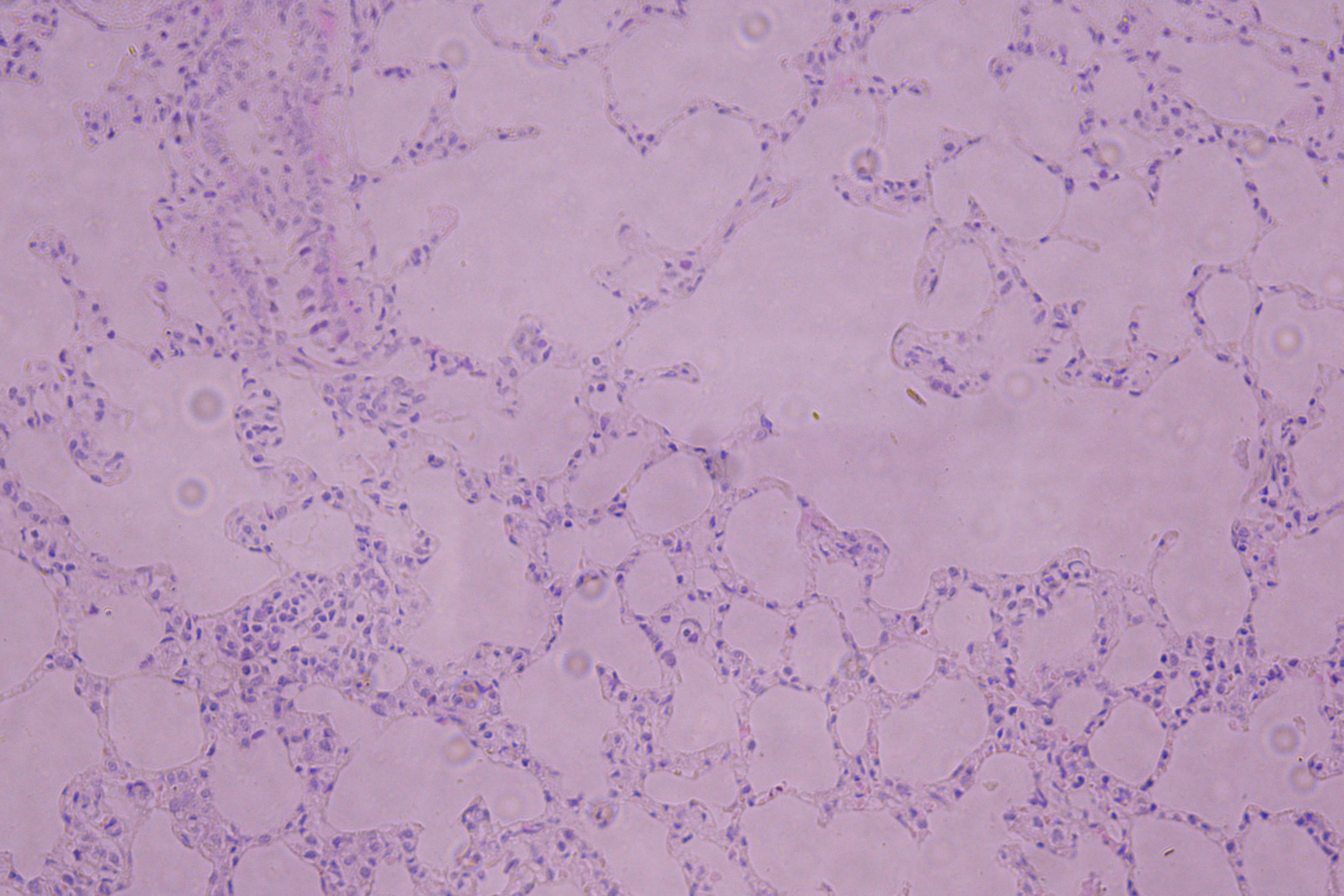

Supplement: Supplemental Information 9 [file peerj-11-14608-s009.zip › Figure 7 image/A/TUMOR+EXO/1.jpg]

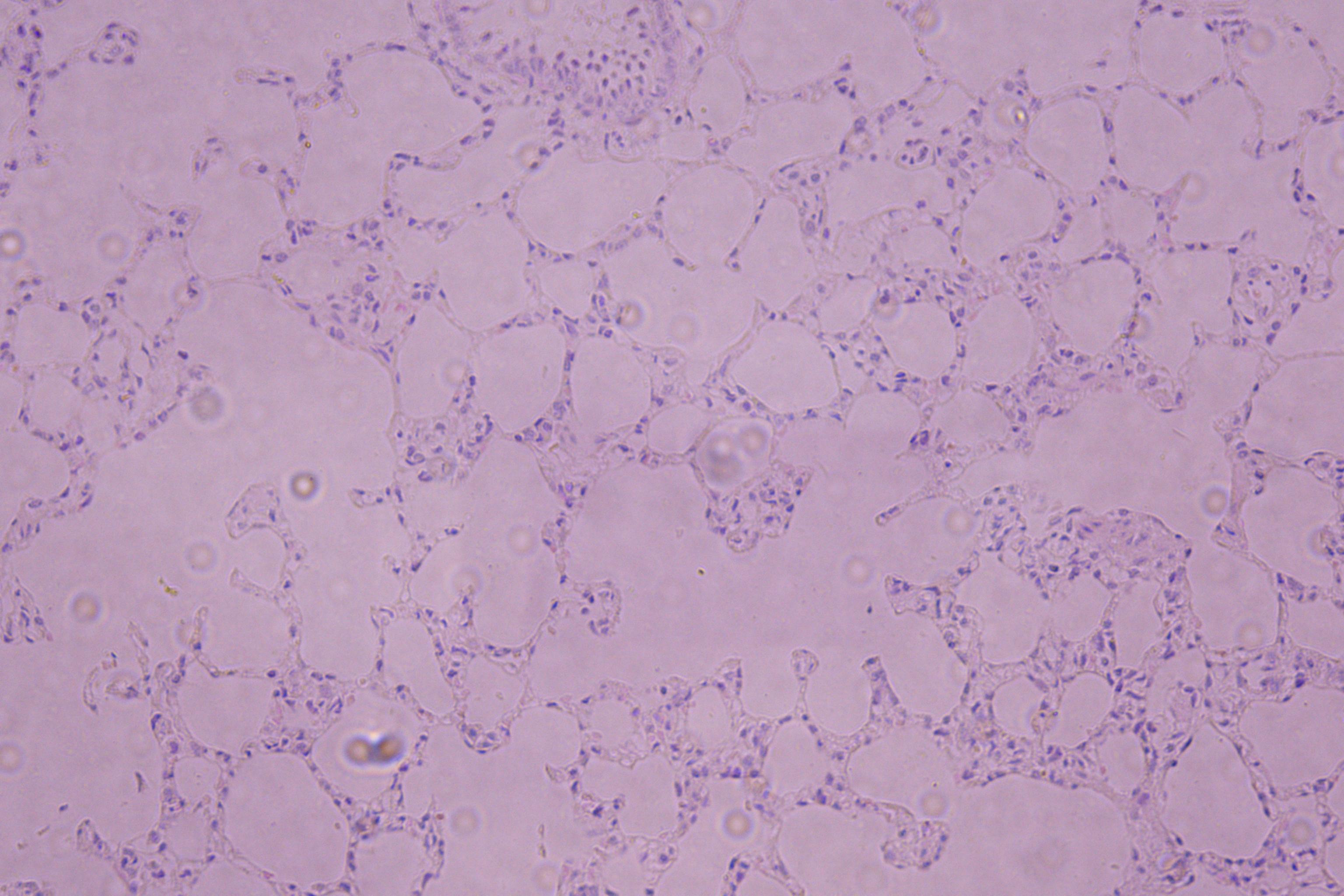

Supplement: Supplemental Information 9 [file peerj-11-14608-s009.zip › Figure 7 image/A/TUMOR+EXO/2.jpg]

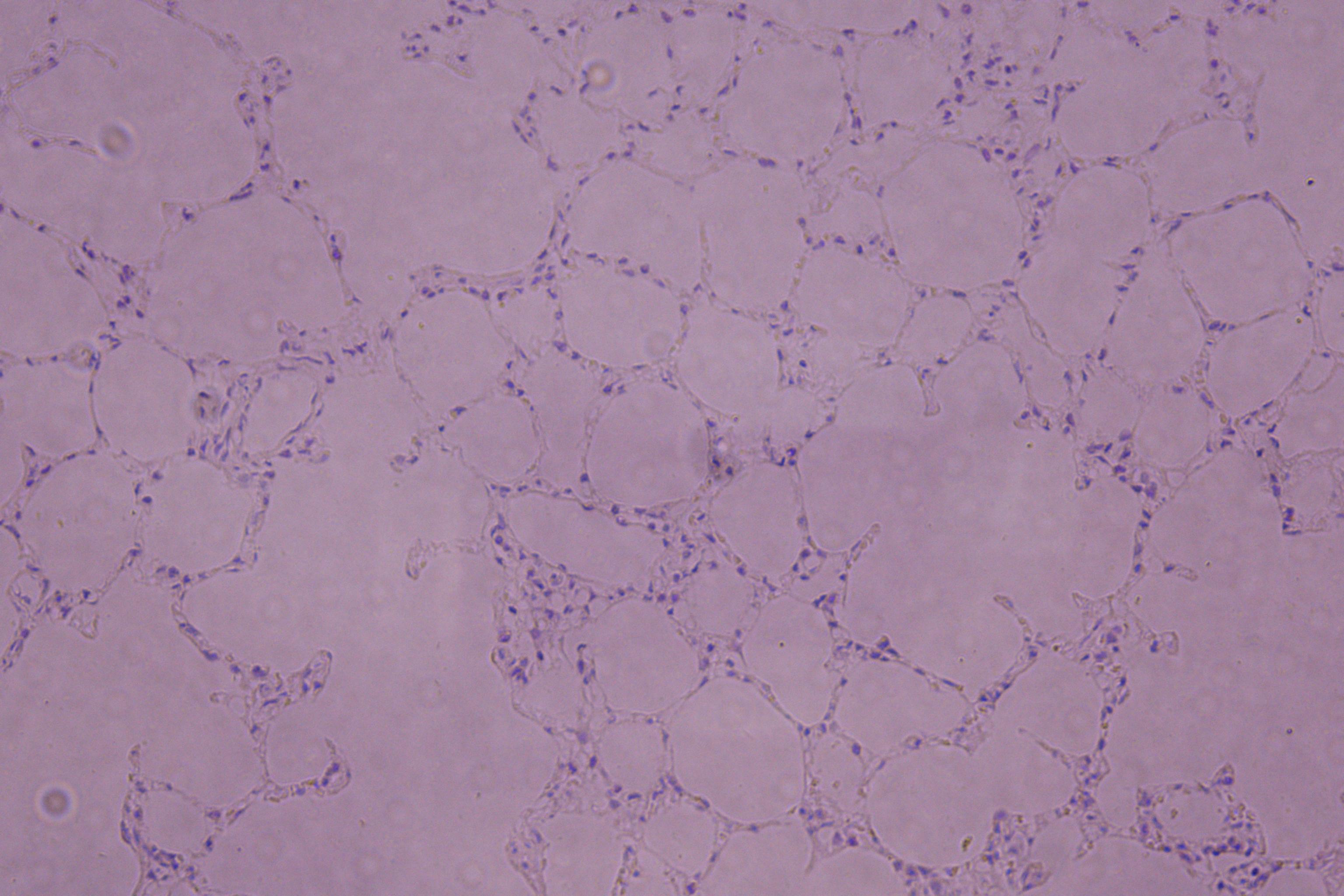

Supplement: Supplemental Information 9 [file peerj-11-14608-s009.zip › Figure 7 image/A/TUMOR+EXO/3.jpg]

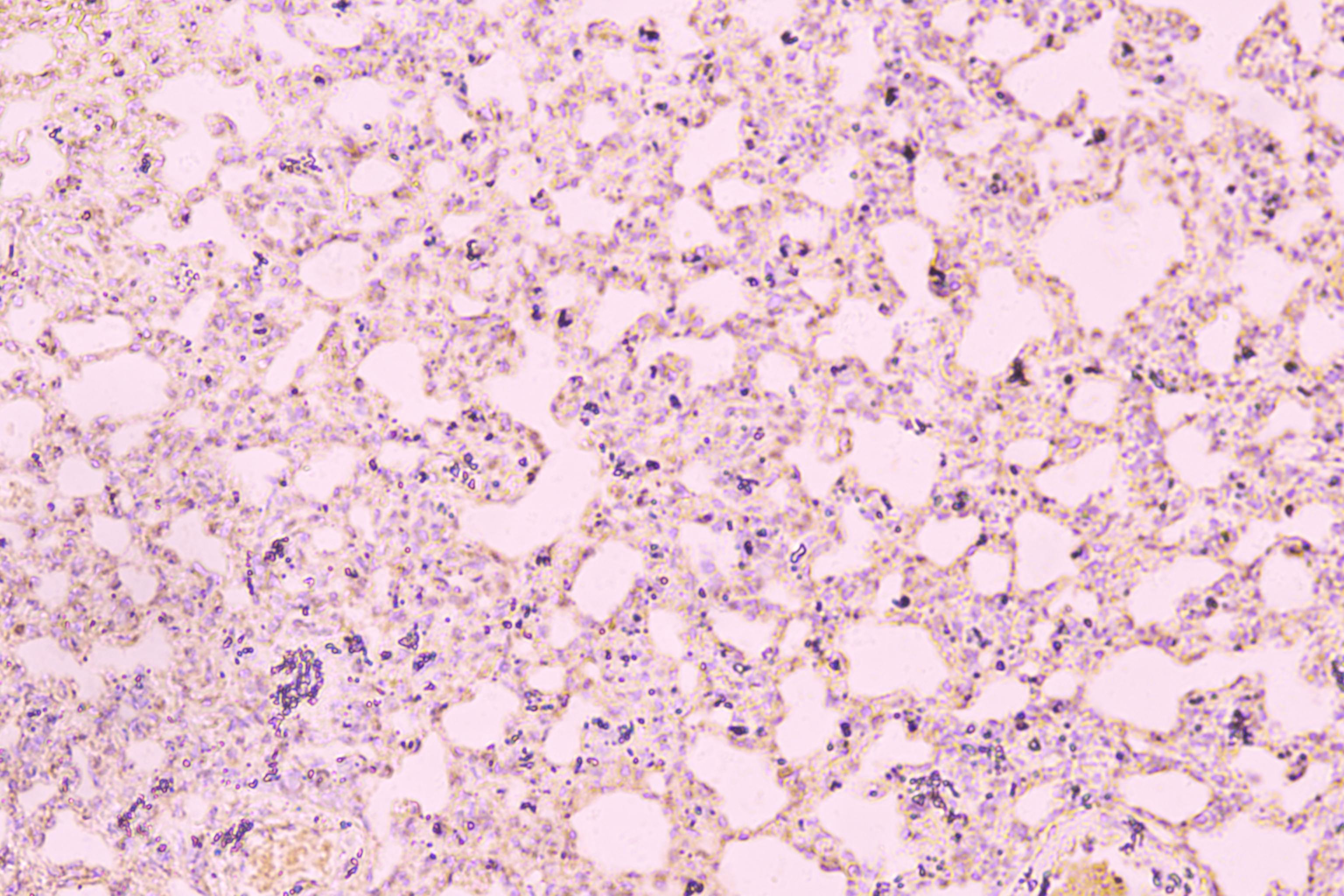

Supplement: Supplemental Information 9 [file peerj-11-14608-s009.zip › Figure 7 image/B/KI67/TUMOR/1 (1).jpg]

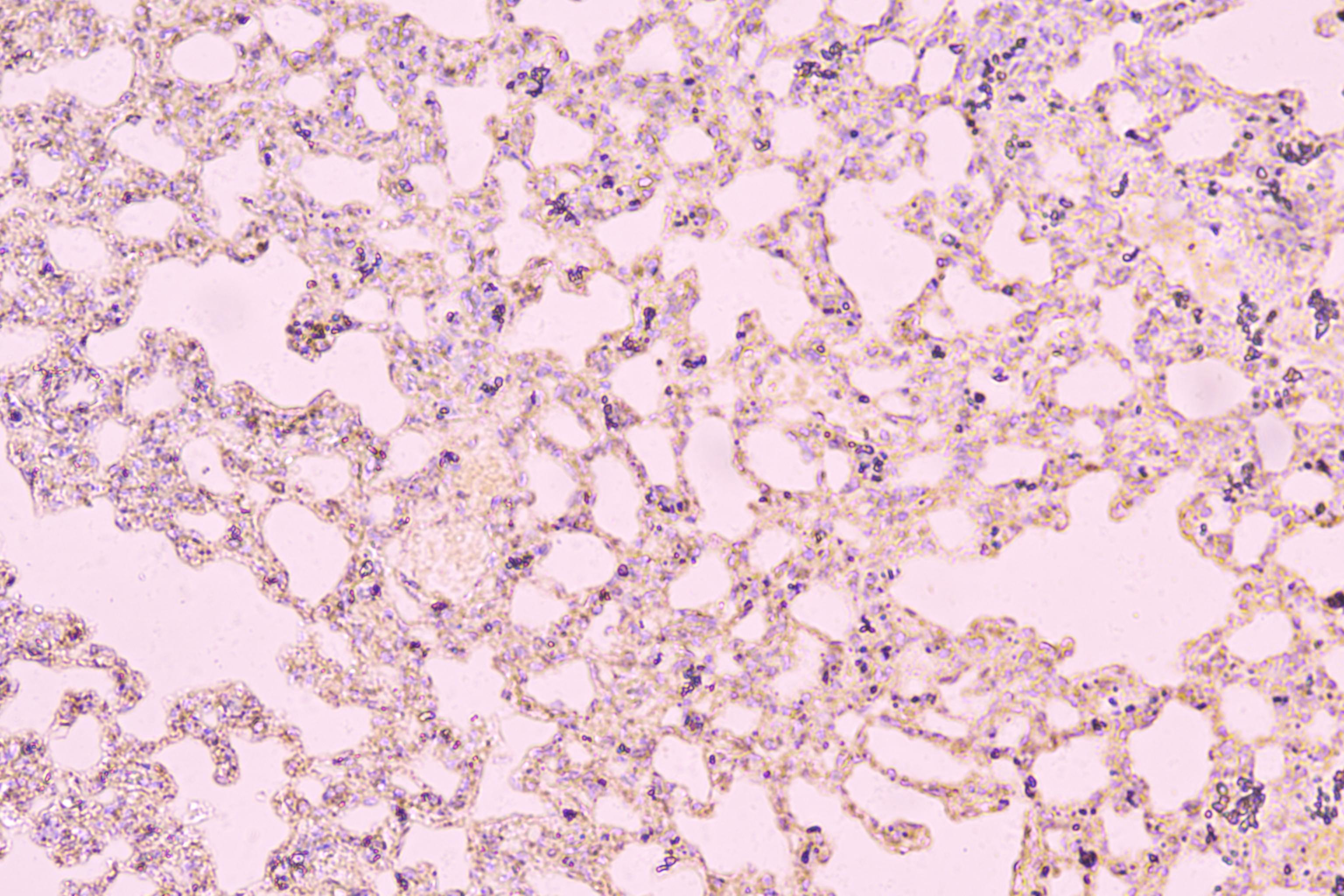

Supplement: Supplemental Information 9 [file peerj-11-14608-s009.zip › Figure 7 image/B/KI67/TUMOR/1 (2).jpg]

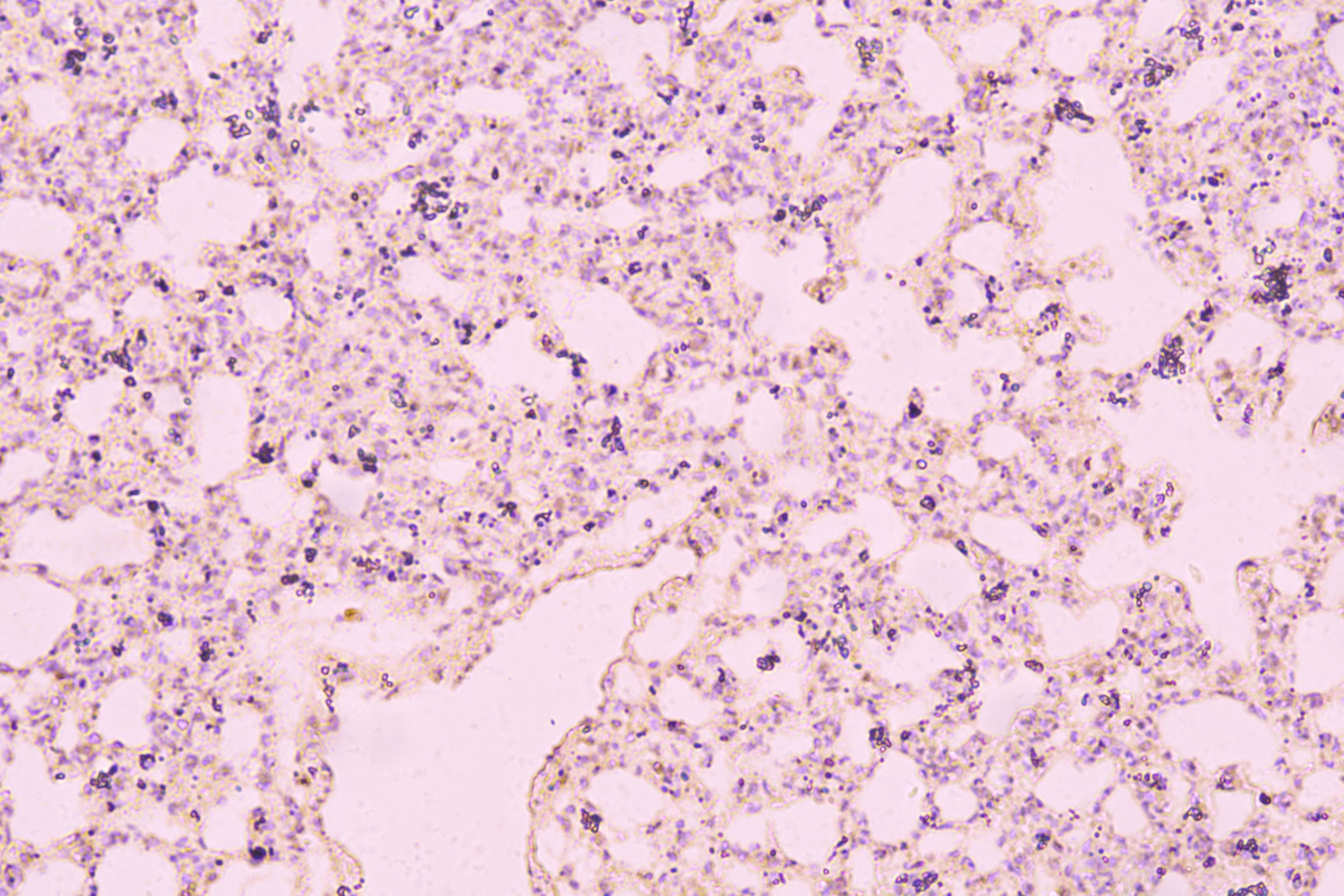

Supplement: Supplemental Information 9 [file peerj-11-14608-s009.zip › Figure 7 image/B/KI67/TUMOR/1 (3).jpg]

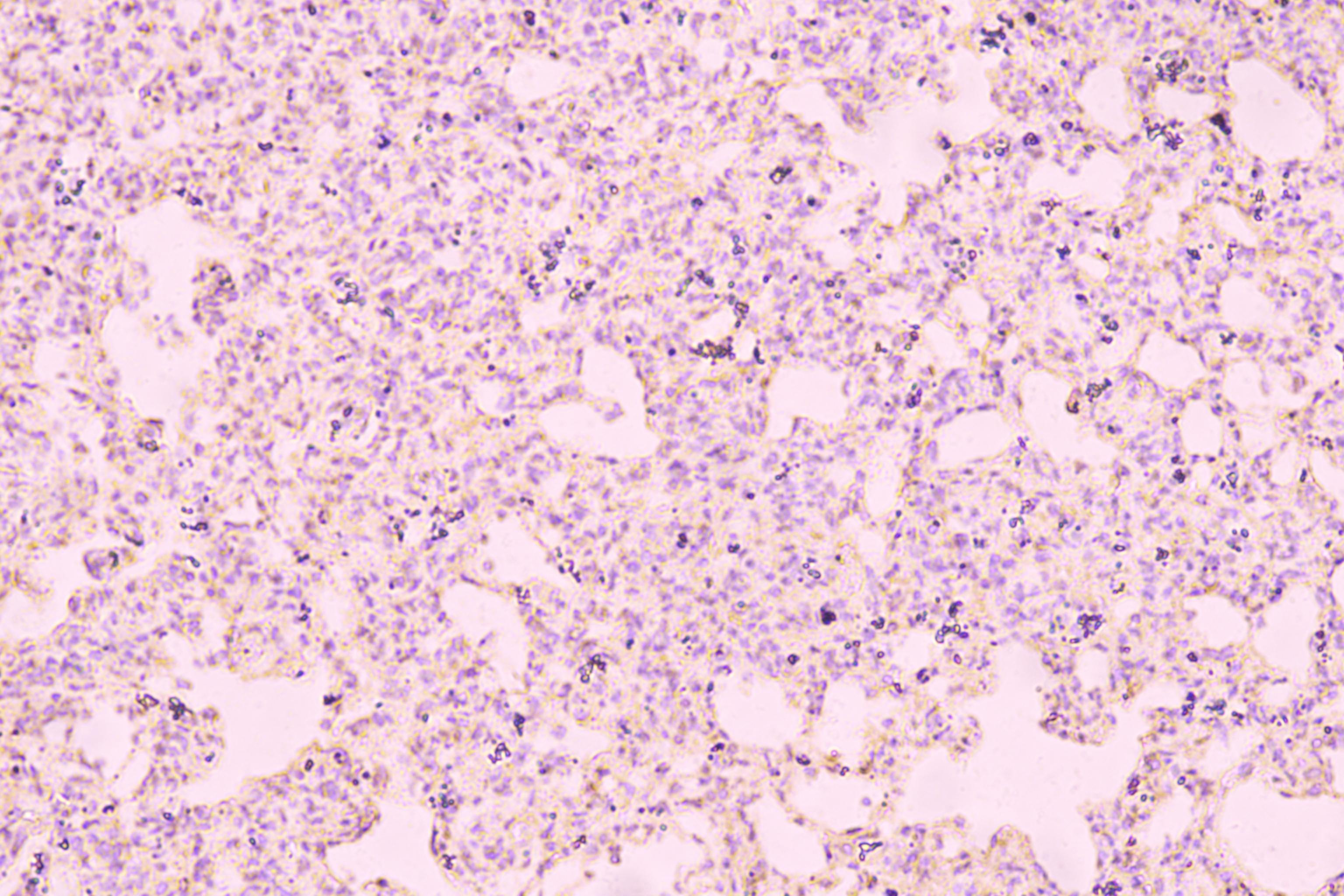

Supplement: Supplemental Information 9 [file peerj-11-14608-s009.zip › Figure 7 image/B/KI67/TUMOR+EXO/1 (1).jpg]

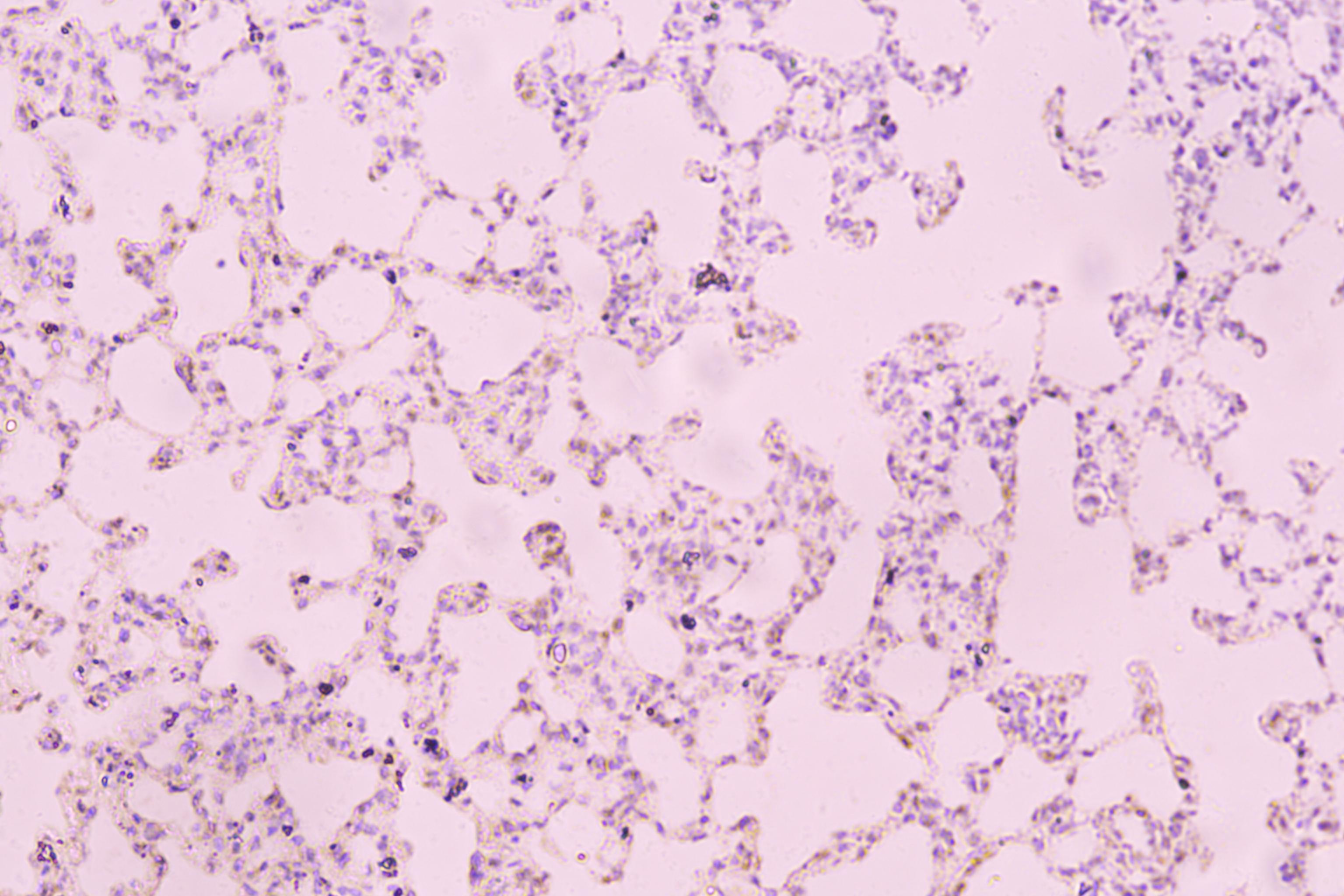

Supplement: Supplemental Information 9 [file peerj-11-14608-s009.zip › Figure 7 image/B/KI67/TUMOR+EXO/1 (2).jpg]

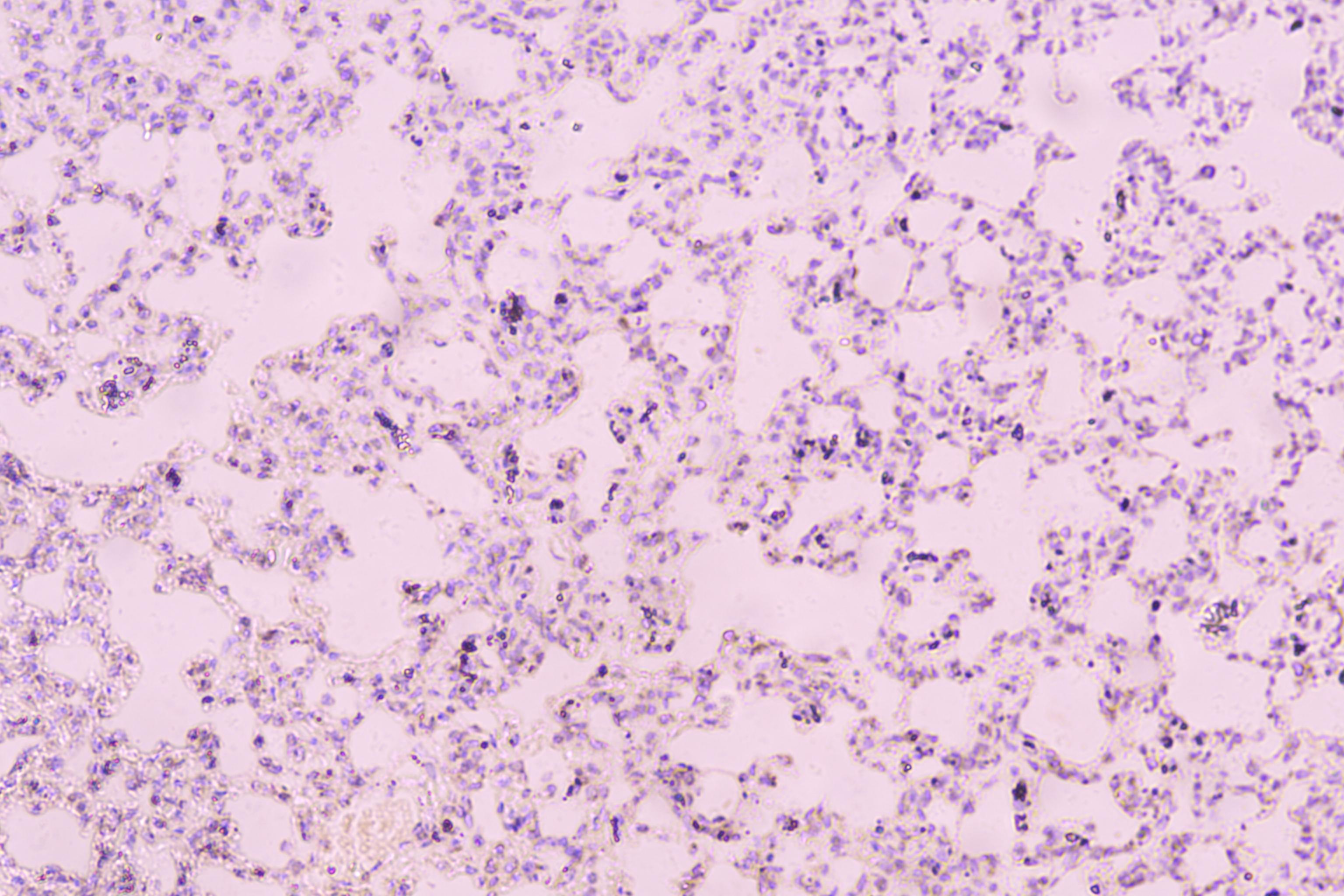

Supplement: Supplemental Information 9 [file peerj-11-14608-s009.zip › Figure 7 image/B/KI67/TUMOR+EXO/1 (3).jpg]

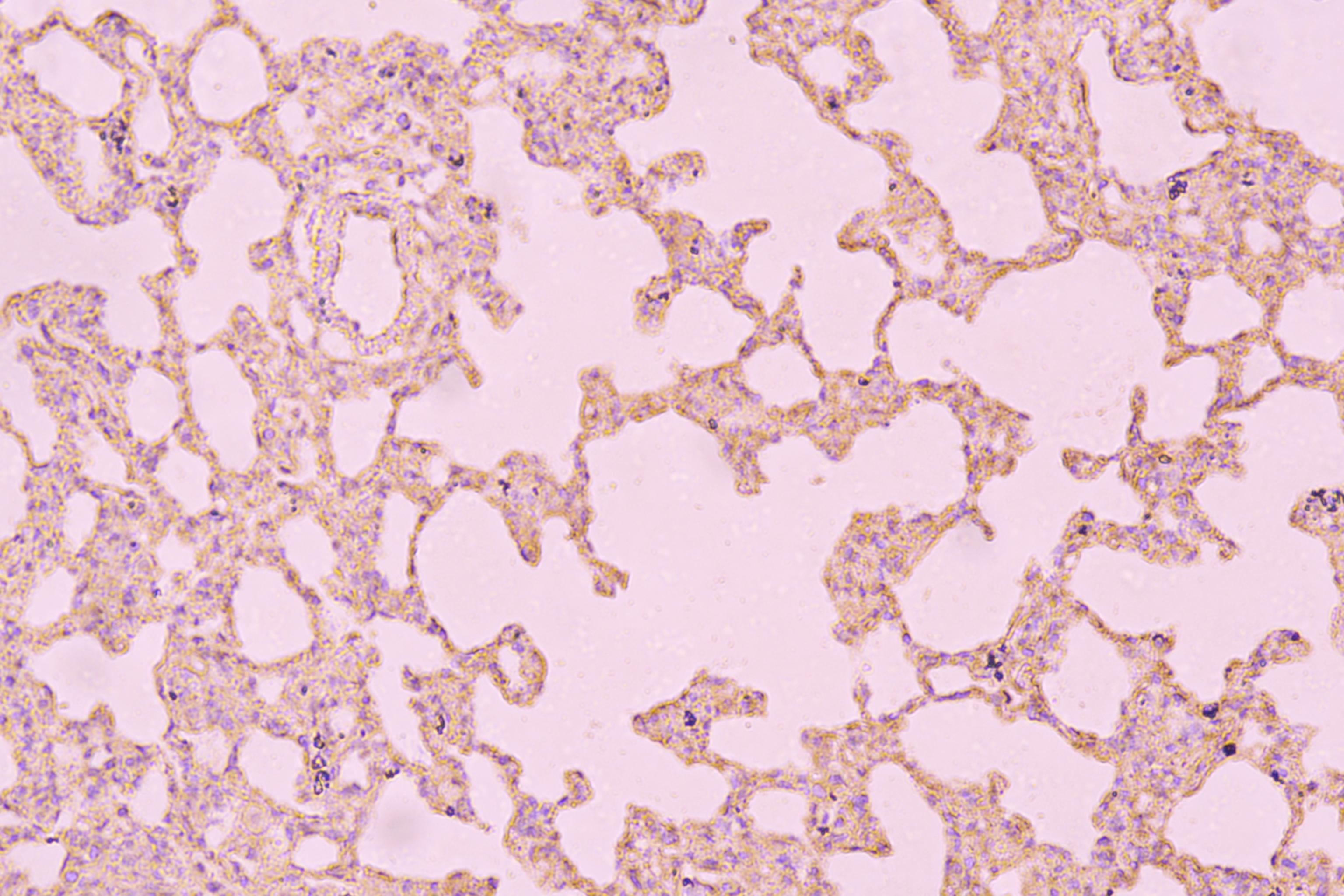

Supplement: Supplemental Information 9 [file peerj-11-14608-s009.zip › Figure 7 image/B/PCNA/TUMOR/1 (1).jpg]

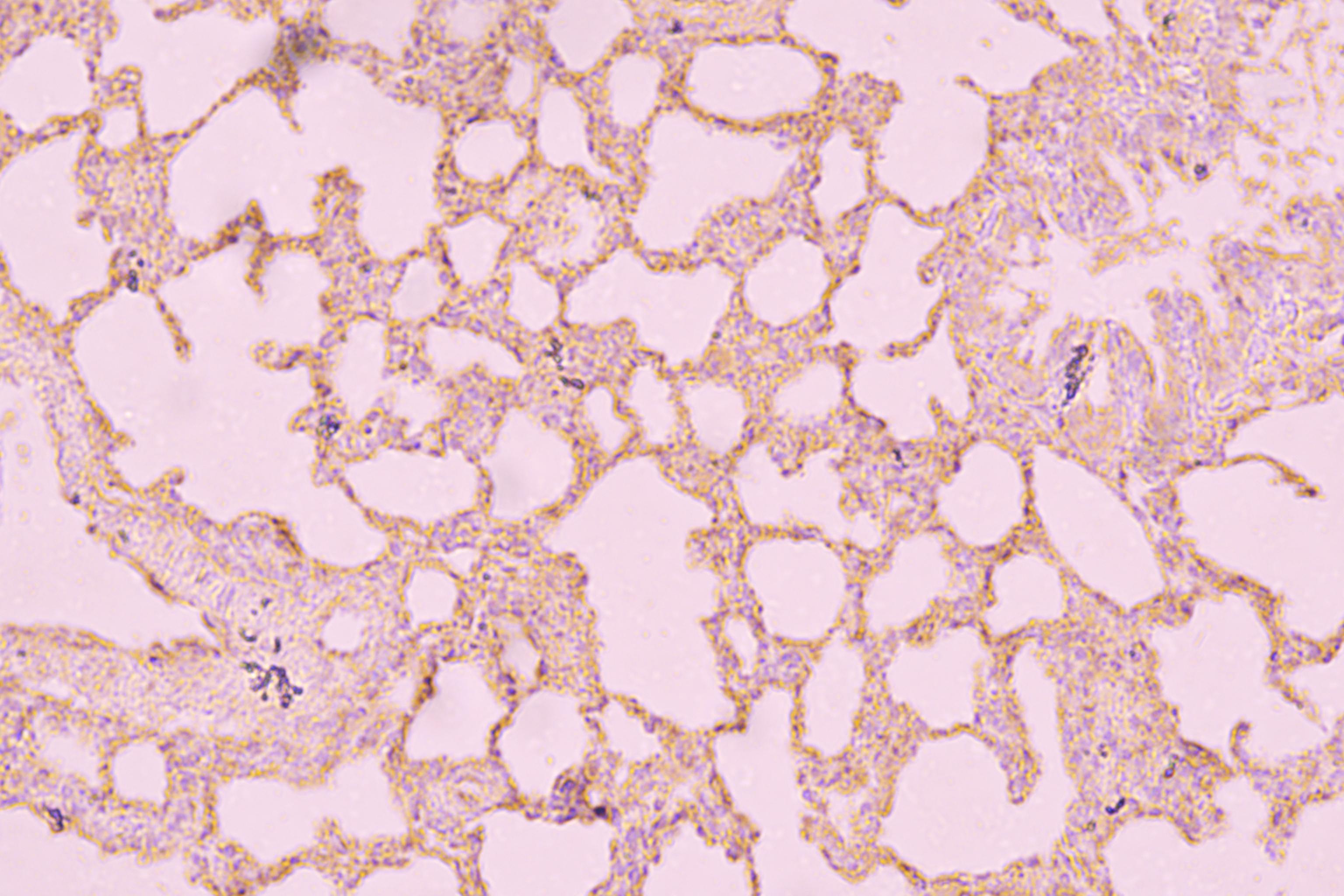

Supplement: Supplemental Information 9 [file peerj-11-14608-s009.zip › Figure 7 image/B/PCNA/TUMOR/1 (2).jpg]

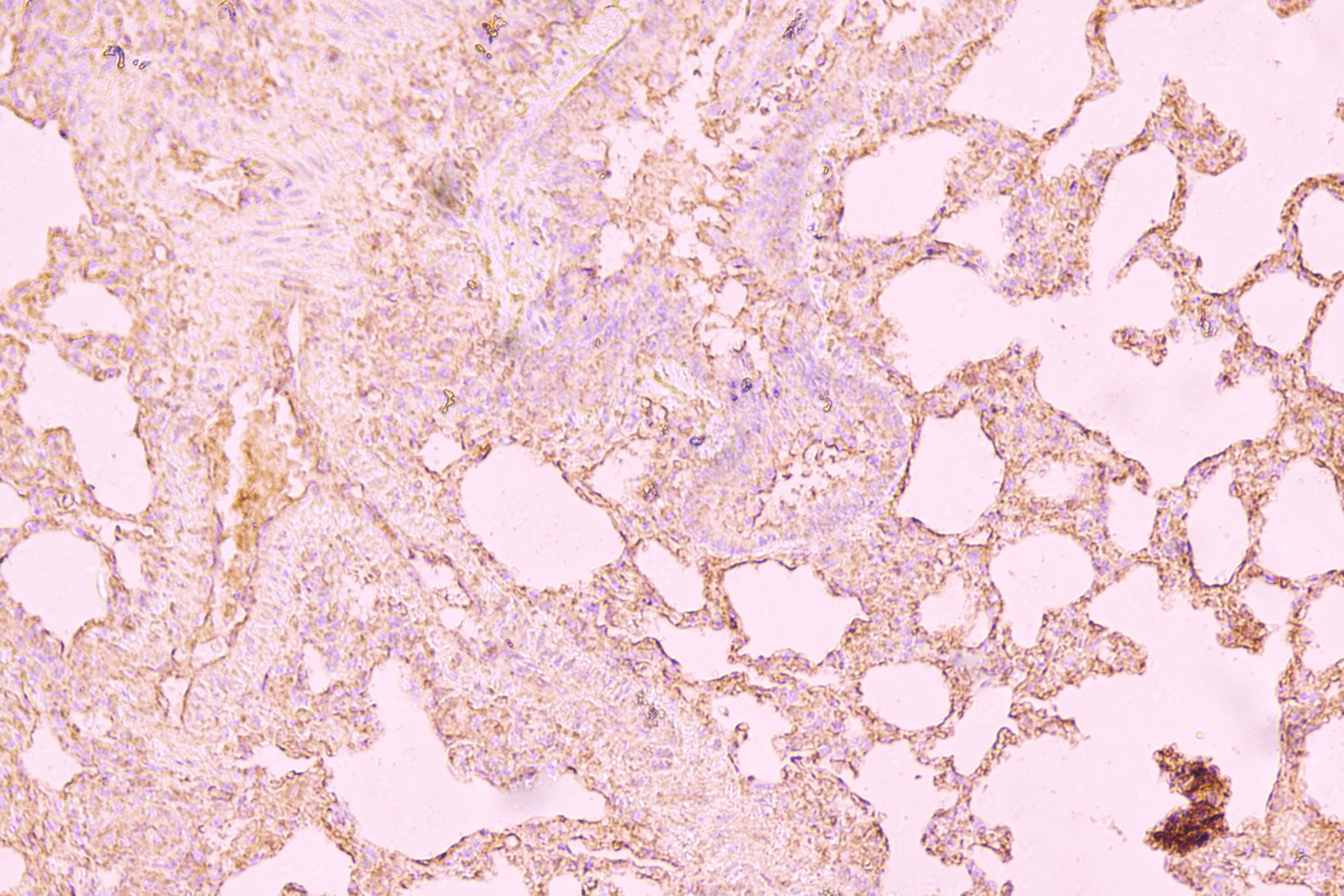

Supplement: Supplemental Information 9 [file peerj-11-14608-s009.zip › Figure 7 image/B/PCNA/TUMOR/1 (3).jpg]

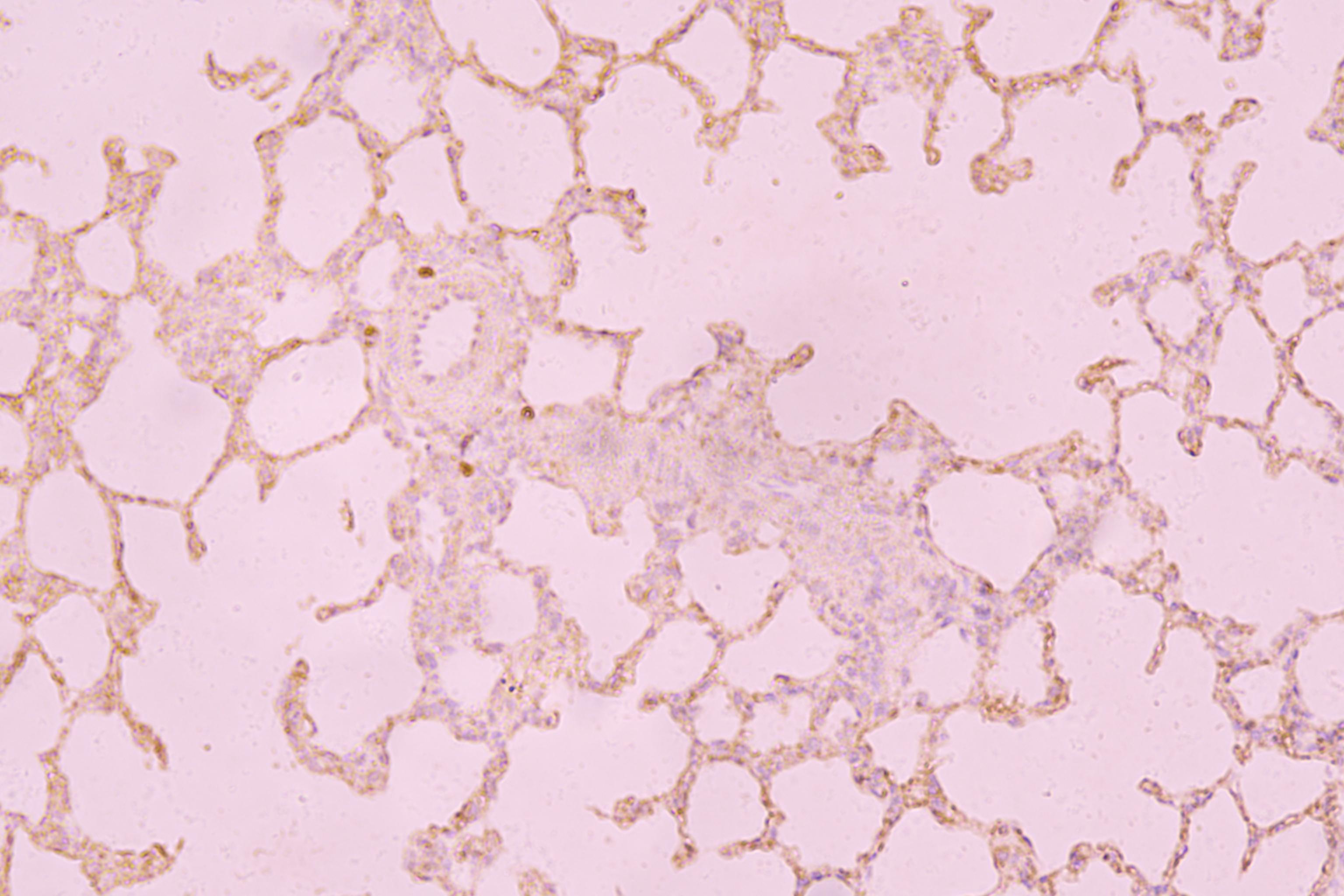

Supplement: Supplemental Information 9 [file peerj-11-14608-s009.zip › Figure 7 image/B/PCNA/TUMOR+EXO/1 (1).jpg]

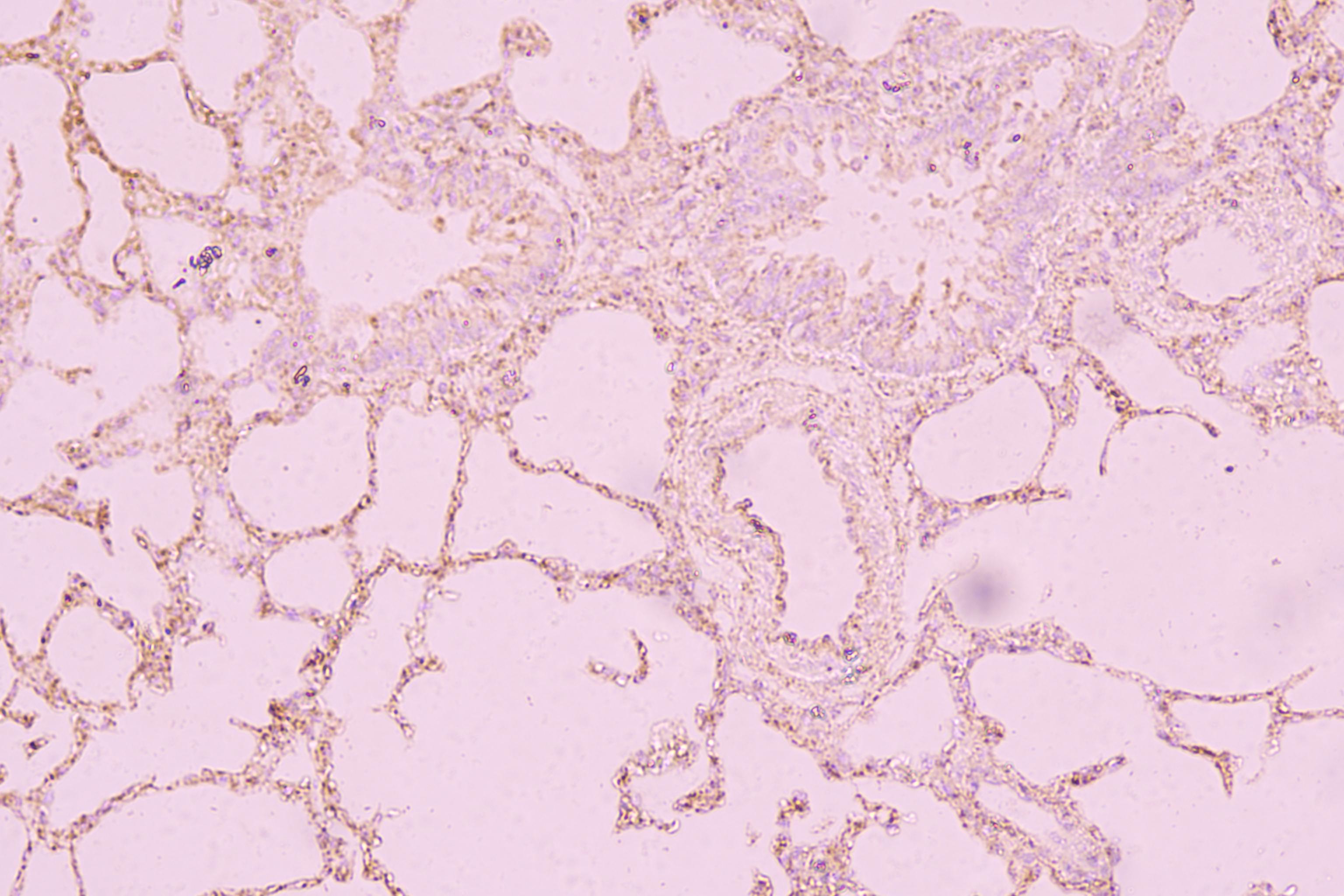

Supplement: Supplemental Information 9 [file peerj-11-14608-s009.zip › Figure 7 image/B/PCNA/TUMOR+EXO/1 (2).jpg]

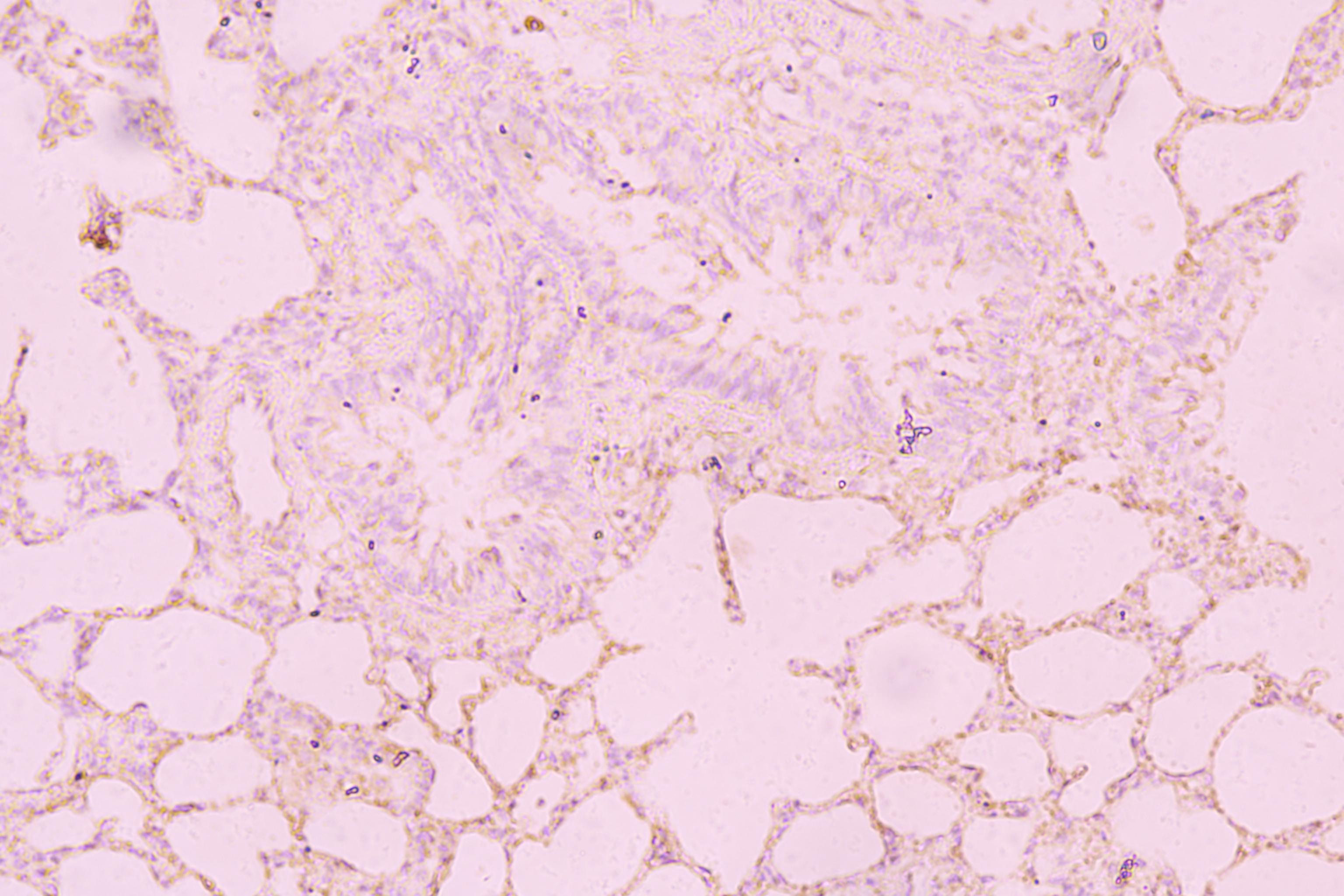

Supplement: Supplemental Information 9 [file peerj-11-14608-s009.zip › Figure 7 image/B/PCNA/TUMOR+EXO/1 (3).jpg]

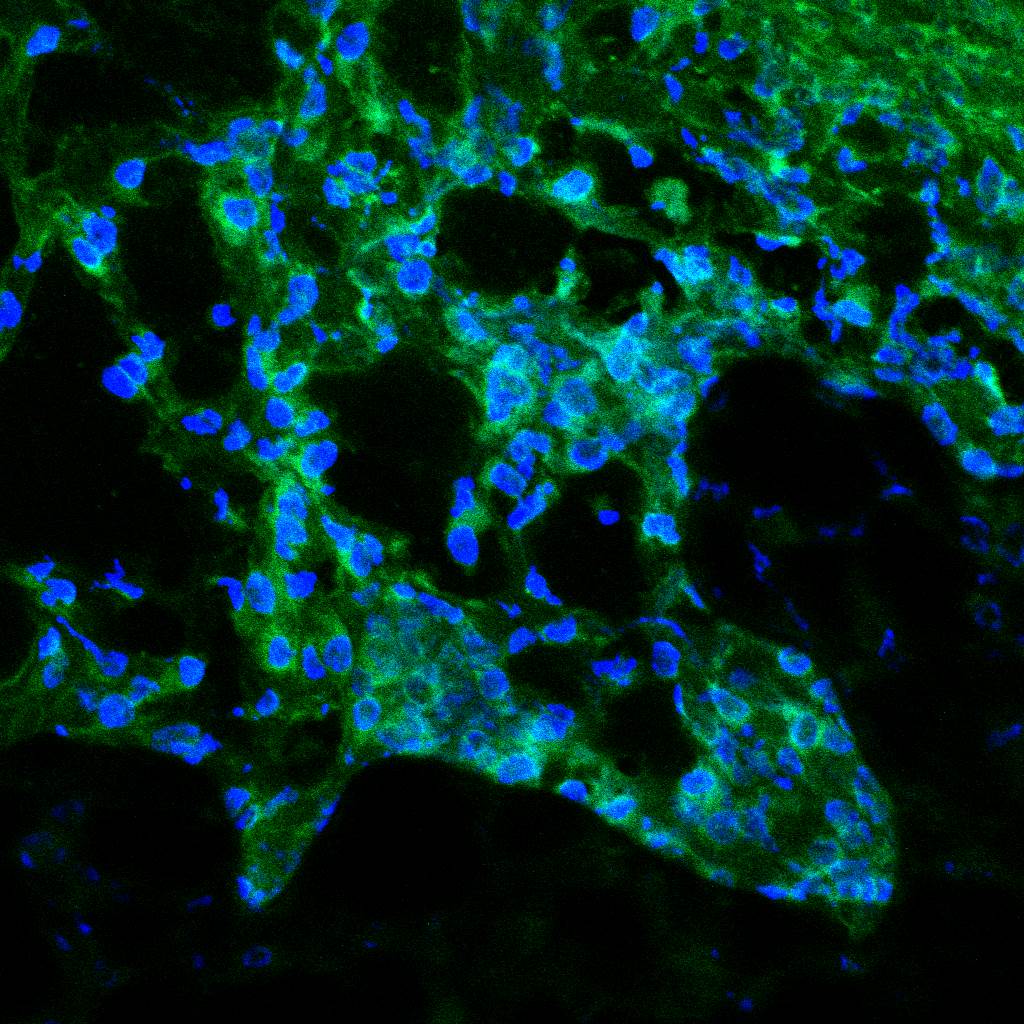

Supplement: Supplemental Information 9 [file peerj-11-14608-s009.zip › Figure 7 image/E/TUMOR/39_.jpg]

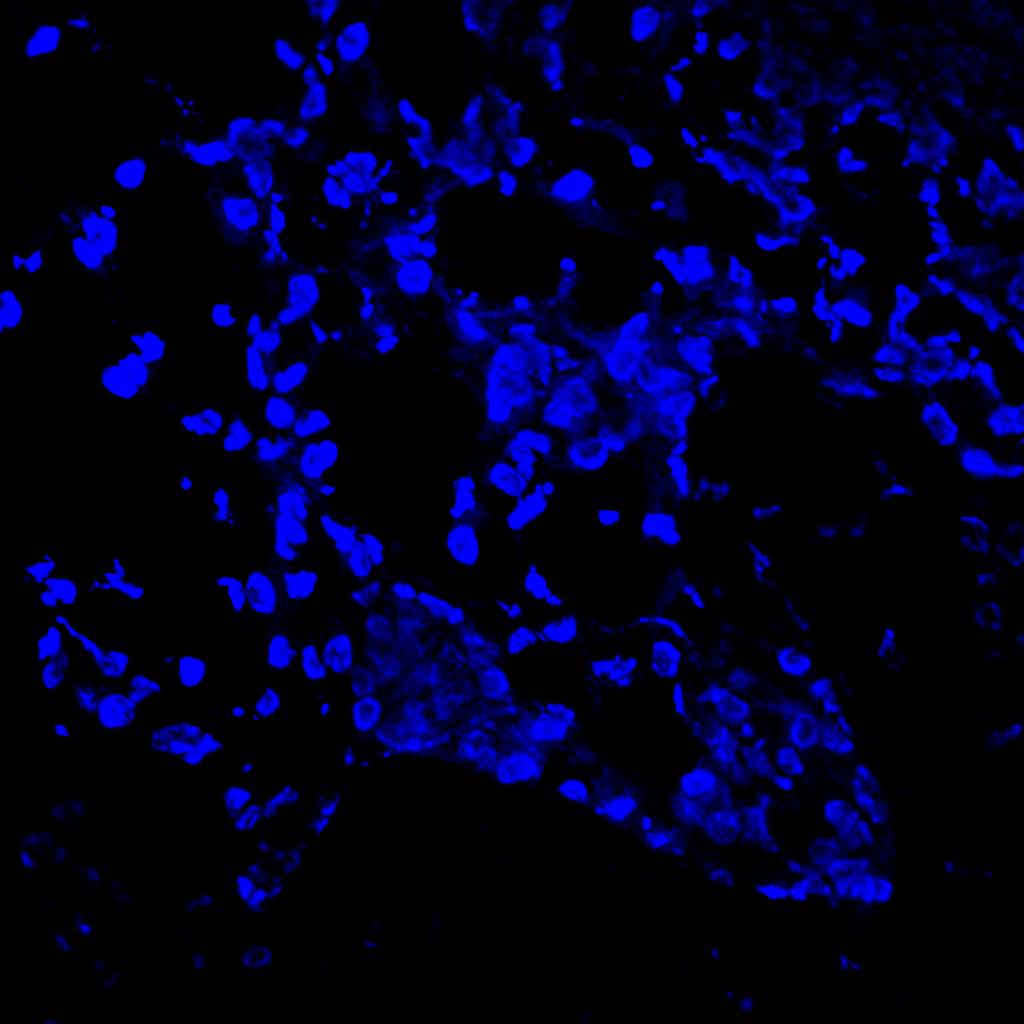

Supplement: Supplemental Information 9 [file peerj-11-14608-s009.zip › Figure 7 image/E/TUMOR/39_C001.jpg]

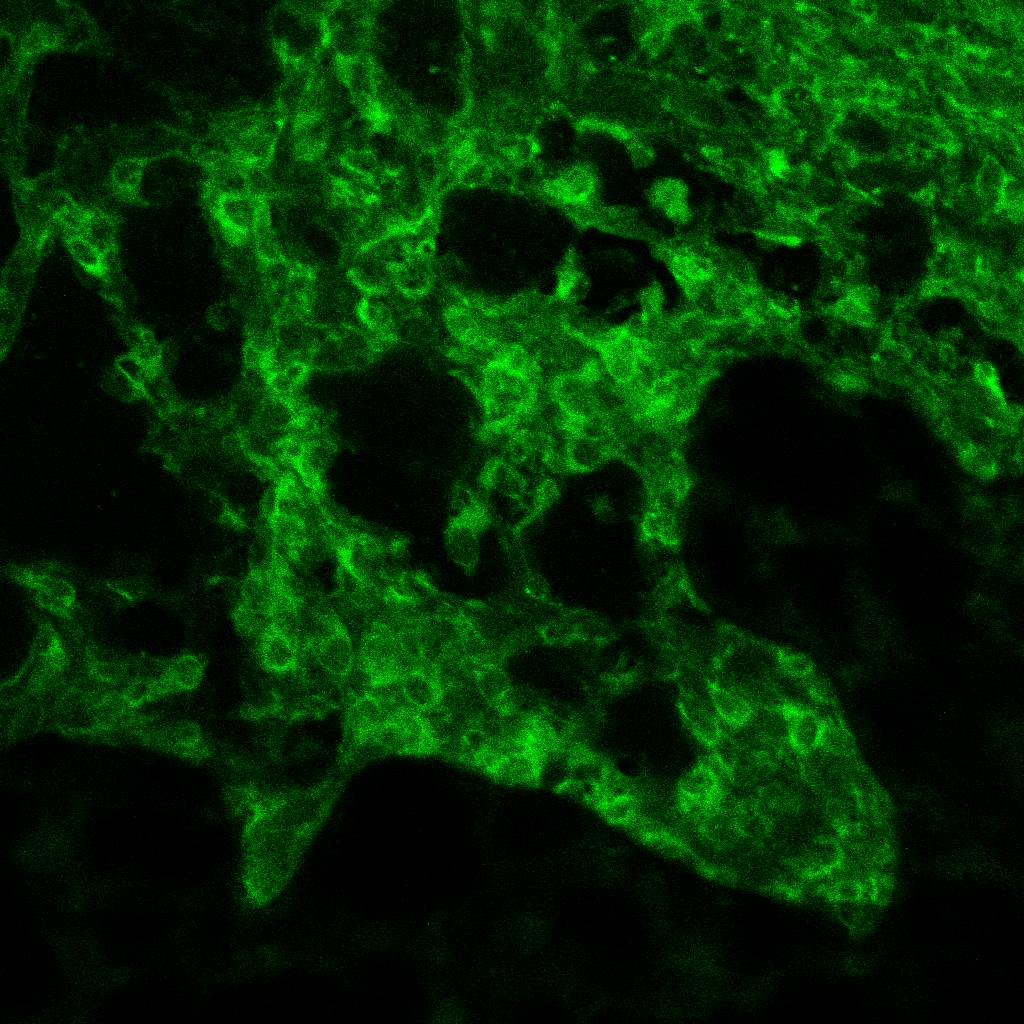

Supplement: Supplemental Information 9 [file peerj-11-14608-s009.zip › Figure 7 image/E/TUMOR/39_C002.jpg]

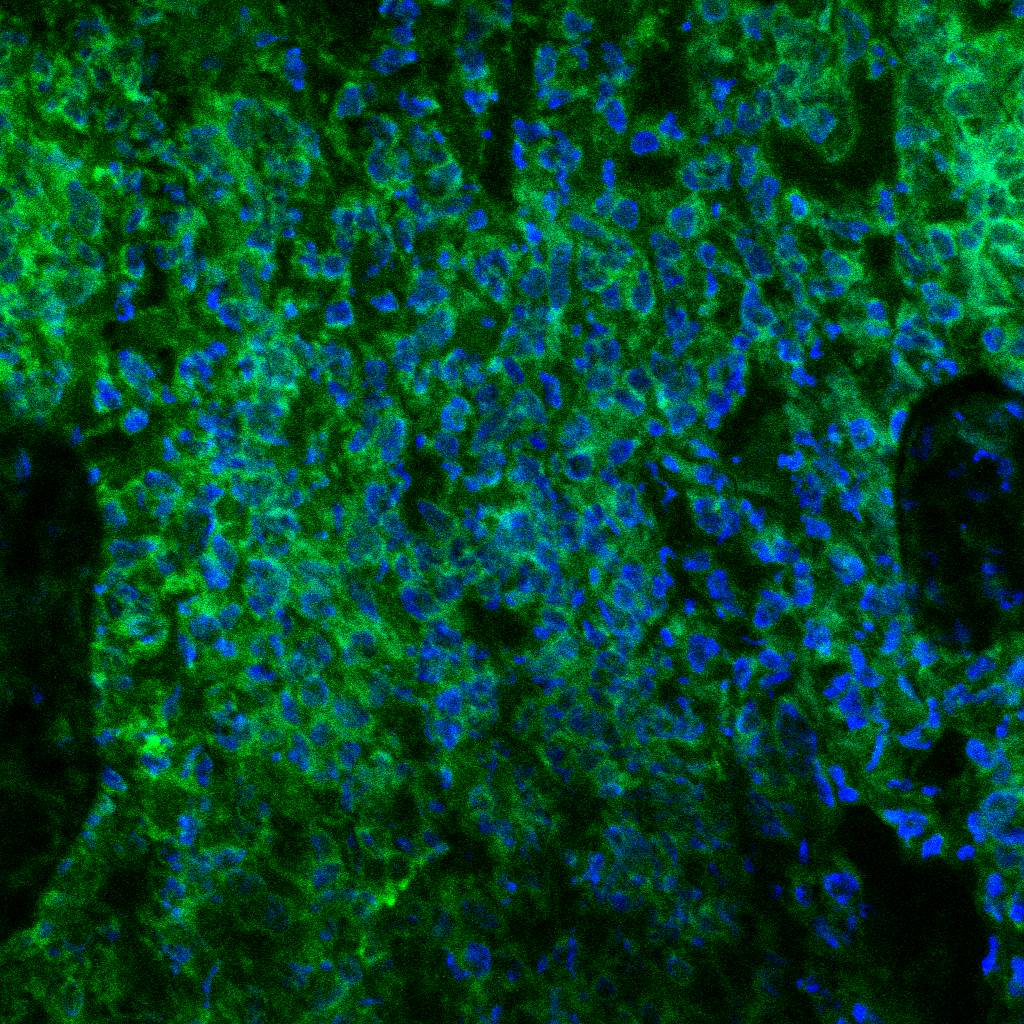

Supplement: Supplemental Information 9 [file peerj-11-14608-s009.zip › Figure 7 image/E/TUMOR+EXO/36_C002_.jpg]

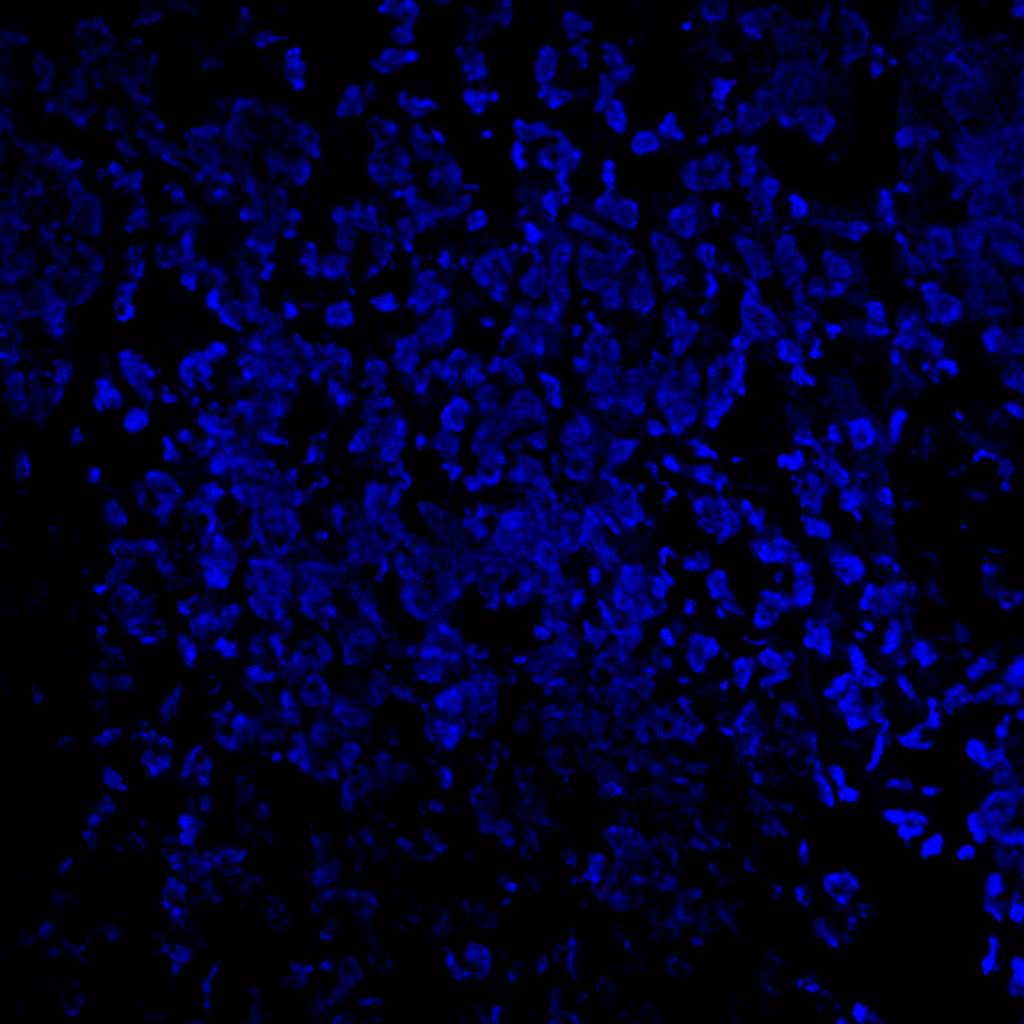

Supplement: Supplemental Information 9 [file peerj-11-14608-s009.zip › Figure 7 image/E/TUMOR+EXO/36_C002_C001.jpg]

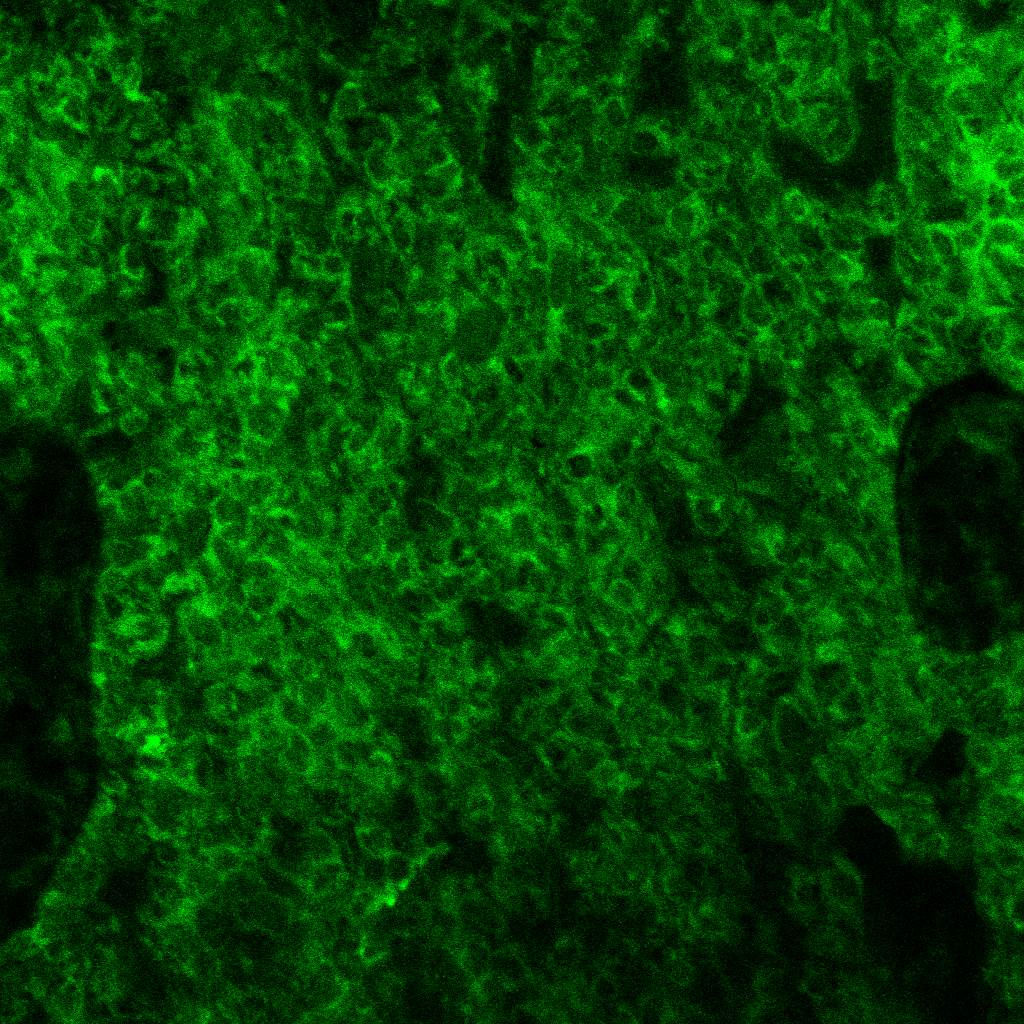

Supplement: Supplemental Information 9 [file peerj-11-14608-s009.zip › Figure 7 image/E/TUMOR+EXO/36_C002_C002.jpg]

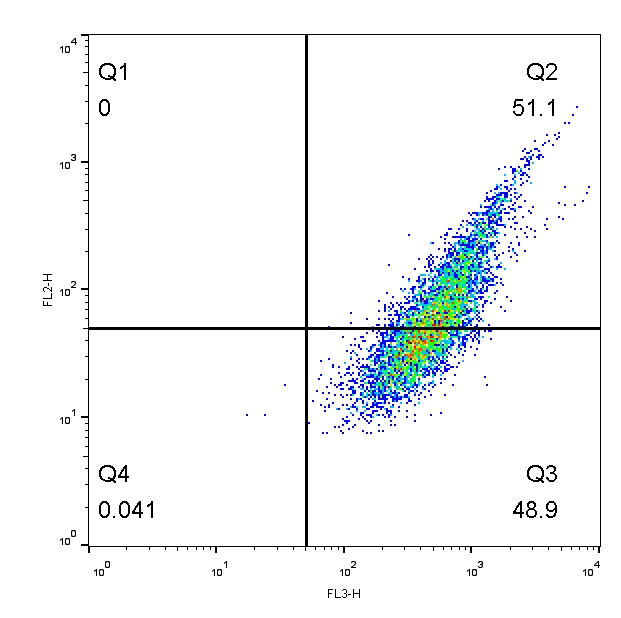

Supplement: Supplemental Information 11 [file peerj-11-14608-s011.zip › other raw data/Figure 1 FCM/M0/MO-NC/Data.1.jpg]

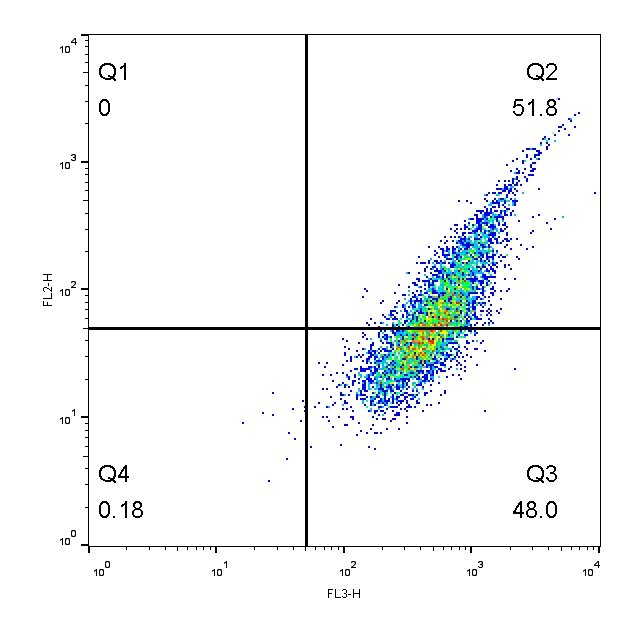

Supplement: Supplemental Information 11 [file peerj-11-14608-s011.zip › other raw data/Figure 1 FCM/M0/MO-NC/Data.2.jpg]

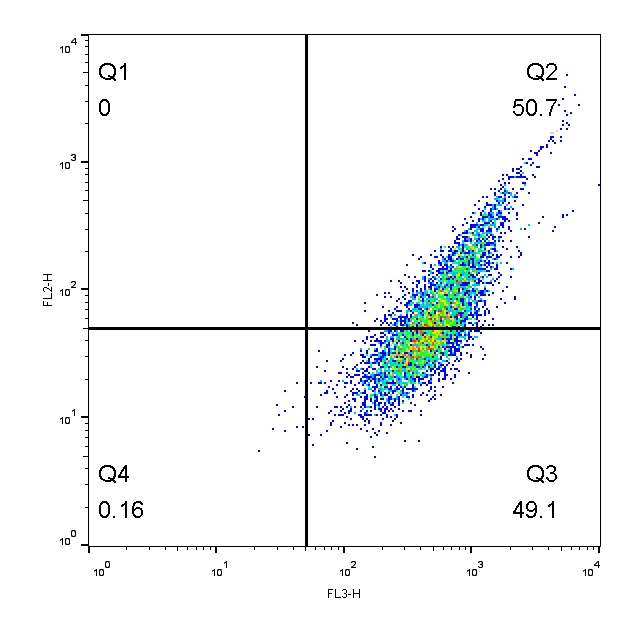

Supplement: Supplemental Information 11 [file peerj-11-14608-s011.zip › other raw data/Figure 1 FCM/M0/MO-NC/Data.3.jpg]

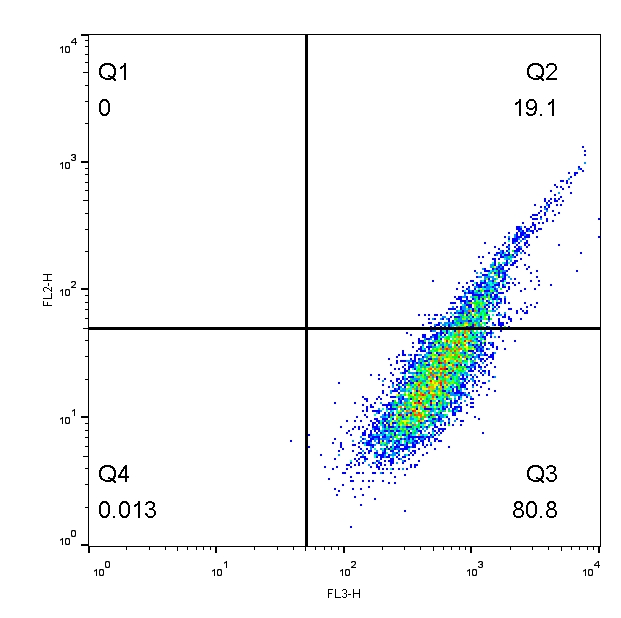

Supplement: Supplemental Information 11 [file peerj-11-14608-s011.zip › other raw data/Figure 1 FCM/M0/MO-VITEXIN/Data.1.jpg]

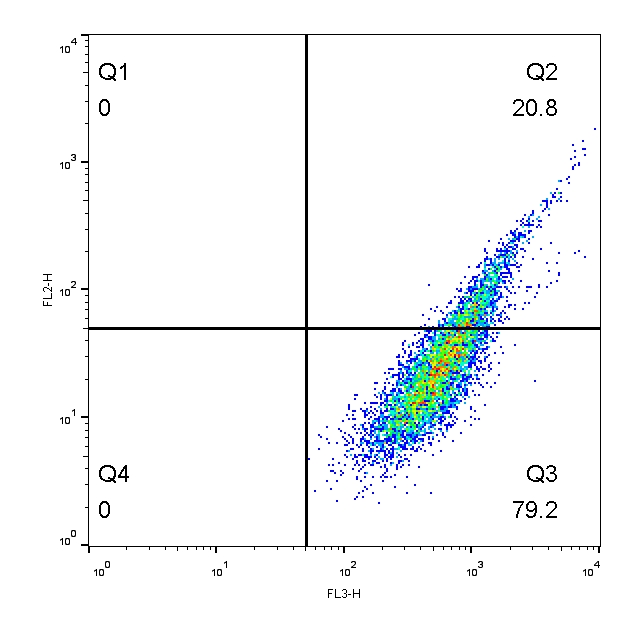

Supplement: Supplemental Information 11 [file peerj-11-14608-s011.zip › other raw data/Figure 1 FCM/M0/MO-VITEXIN/Data.2.jpg]

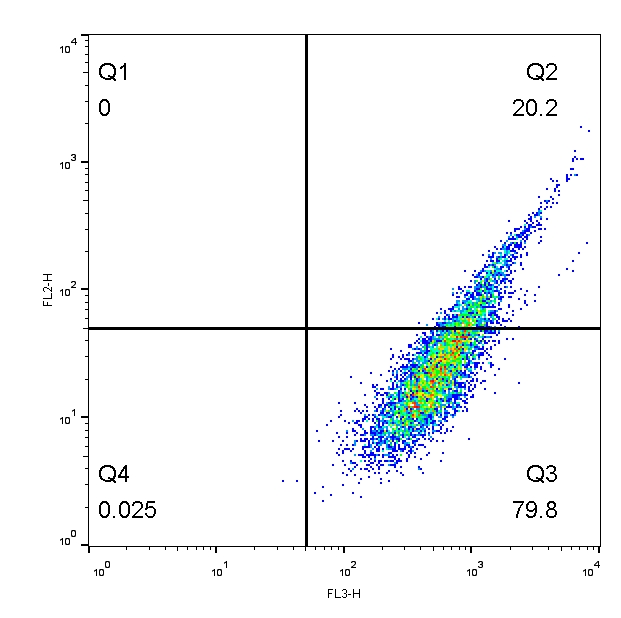

Supplement: Supplemental Information 11 [file peerj-11-14608-s011.zip › other raw data/Figure 1 FCM/M0/MO-VITEXIN/Data.3.jpg]

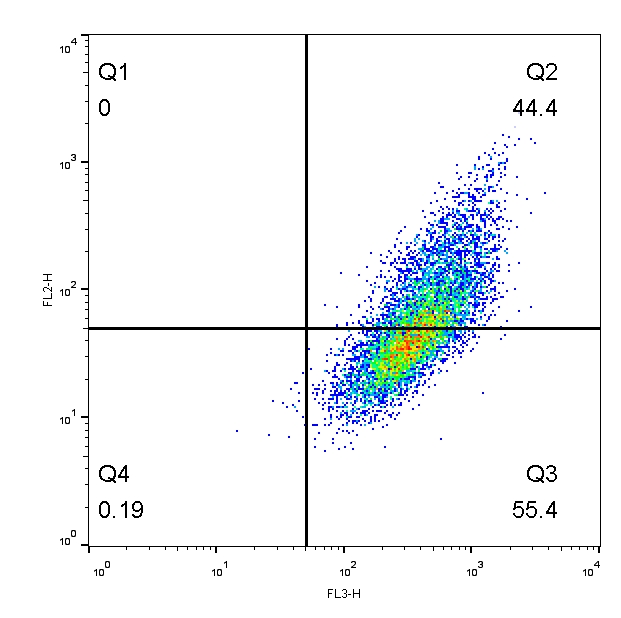

Supplement: Supplemental Information 11 [file peerj-11-14608-s011.zip › other raw data/Figure 1 FCM/M2/MO-NC/Data.1.jpg]

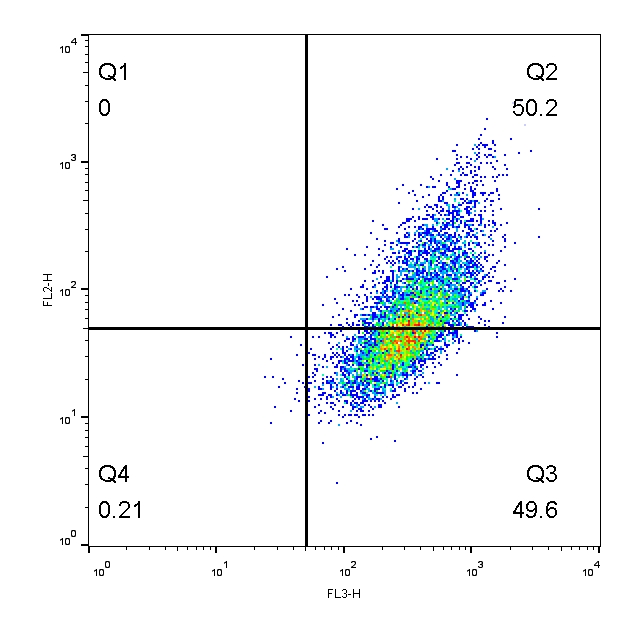

Supplement: Supplemental Information 11 [file peerj-11-14608-s011.zip › other raw data/Figure 1 FCM/M2/MO-NC/Data.2.jpg]

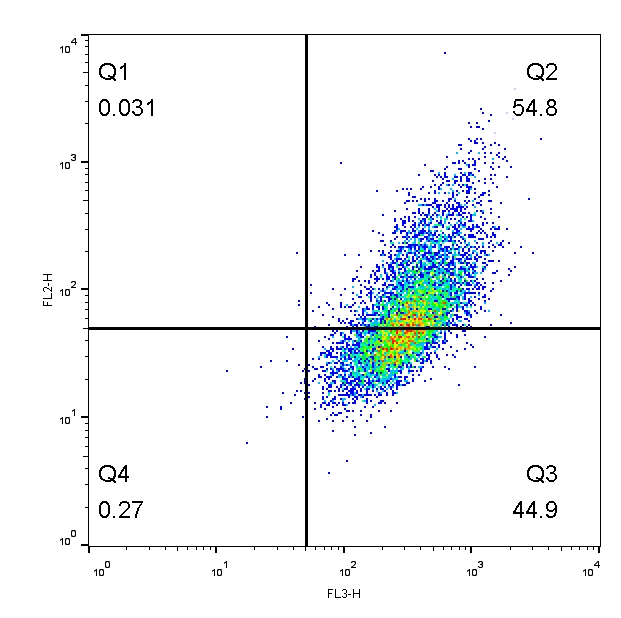

Supplement: Supplemental Information 11 [file peerj-11-14608-s011.zip › other raw data/Figure 1 FCM/M2/MO-NC/Data.3.jpg]

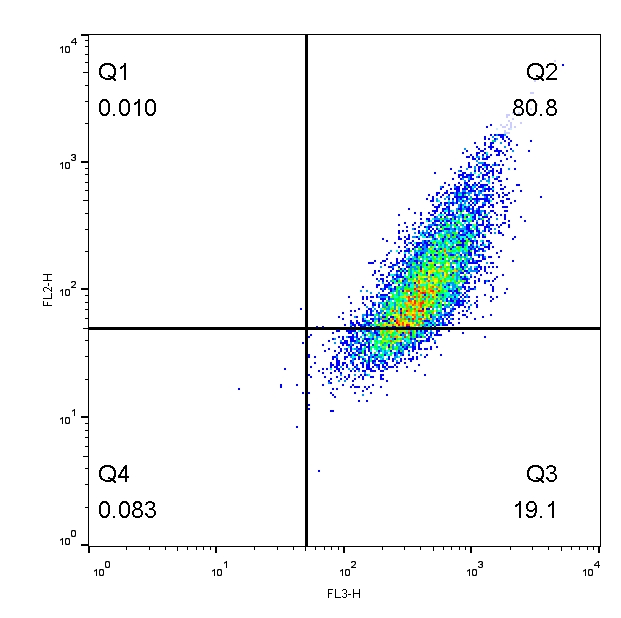

Supplement: Supplemental Information 11 [file peerj-11-14608-s011.zip › other raw data/Figure 1 FCM/M2/MO-VITEXIN/Data.1.jpg]

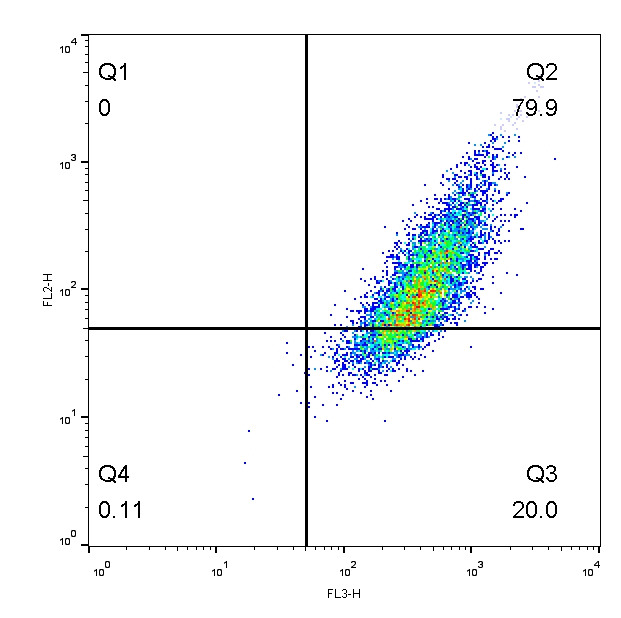

Supplement: Supplemental Information 11 [file peerj-11-14608-s011.zip › other raw data/Figure 1 FCM/M2/MO-VITEXIN/Data.2.jpg]

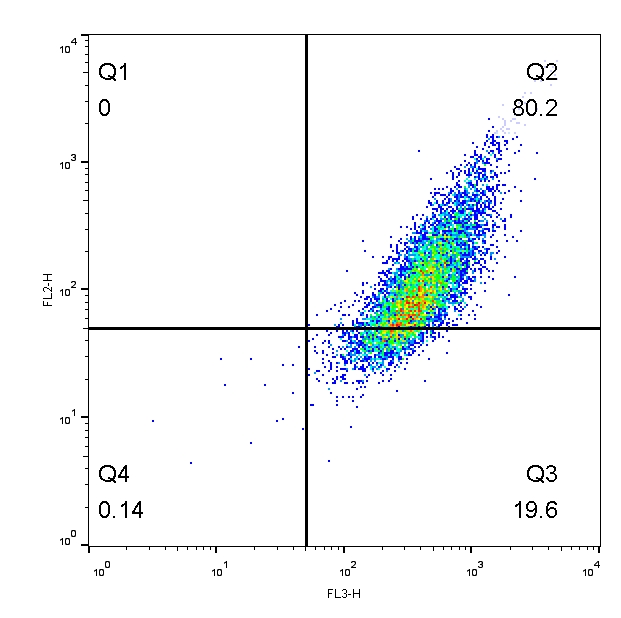

Supplement: Supplemental Information 11 [file peerj-11-14608-s011.zip › other raw data/Figure 1 FCM/M2/MO-VITEXIN/Data.3.jpg]

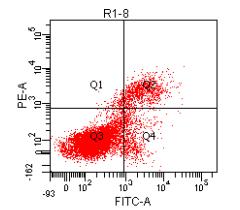

Supplement: Supplemental Information 11 [file peerj-11-14608-s011.zip › other raw data/Figure2/D FITC/A549+M1.jpg]

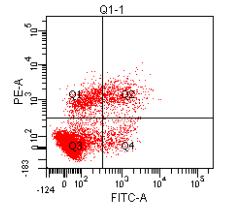

Supplement: Supplemental Information 11 [file peerj-11-14608-s011.zip › other raw data/Figure2/D FITC/A549.jpg]

# FACSDiva Version 6.1.3

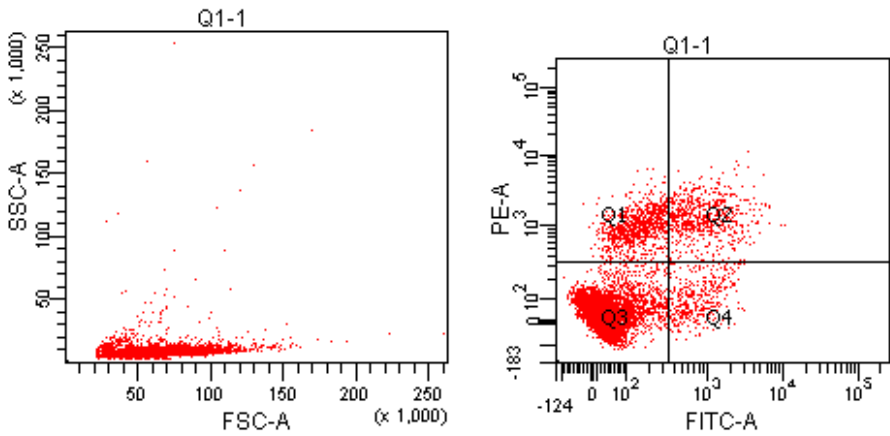

| Tube: Q1-1   |         |         |        |
|--------------|---------|---------|--------|
| Population   | #Events | %Parent | %Total |
| ■ All Events | 10,000  | ###     | 100.0  |
| ▣ P1         | 348     | 3.5     | 3.5    |
| ▣ Q1         | 822     | 8.2     | 8.2    |
| ▣ Q2         | 626     | 6.3     | 6.3    |
| ▣ Q3         | 8,177   | 81.8    | 81.8   |
| ▣ Q4         | 375     | 3.8     | 3.8    |

Supplement: Supplemental Information 11 [file peerj-11-14608-s011.zip › other raw data/Figure2/D FITC/A549.pdf]

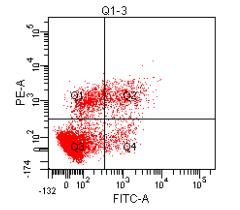

Supplement: Supplemental Information 11 [file peerj-11-14608-s011.zip › other raw data/Figure2/D FITC/HLF-A+M1.jpg]

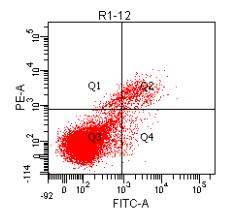

Supplement: Supplemental Information 11 [file peerj-11-14608-s011.zip › other raw data/Figure2/D FITC/HLF-A.jpg]

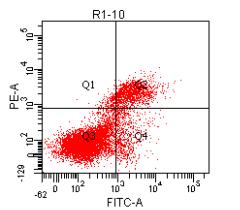

Supplement: Supplemental Information 11 [file peerj-11-14608-s011.zip › other raw data/Figure3/D FITC/A549+EXO.jpg]

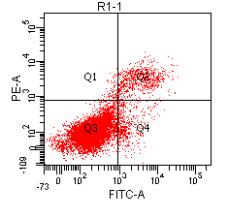

Supplement: Supplemental Information 11 [file peerj-11-14608-s011.zip › other raw data/Figure3/D FITC/A549.jpg]

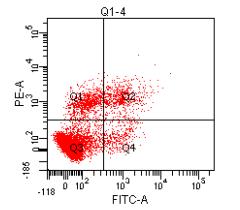

Supplement: Supplemental Information 11 [file peerj-11-14608-s011.zip › other raw data/Figure3/D FITC/HLF-A+EXO.jpg]

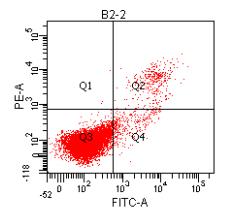

Supplement: Supplemental Information 11 [file peerj-11-14608-s011.zip › other raw data/Figure3/D FITC/HLF-A.jpg]

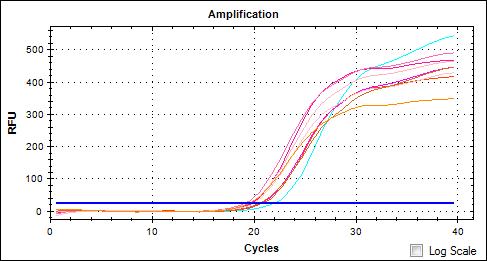

Supplement: Supplemental Information 11 [file peerj-11-14608-s011.zip › other raw data/Figure4/A/miRNA-23a-3P Amplification curve.jpg]
