# Supplementary material for: Exosomes derived from M1 macrophages inhibit the proliferation of the A549 and H1299 lung cancer cell lines via the miRNA-let-7b-5p-GNG5 axis
Source: PeerJ. 2023 Jan 9;11:e14608. doi: 10.7717/peerj.14608 (PMC9835688; doi:10.7717/peerj.14608)
Supplement: Supplemental Information 11 [file peerj-11-14608-s011.zip › other raw data/Figure2/D FITC/A549+M1.pdf]

FACSDiva Version 6.1.3

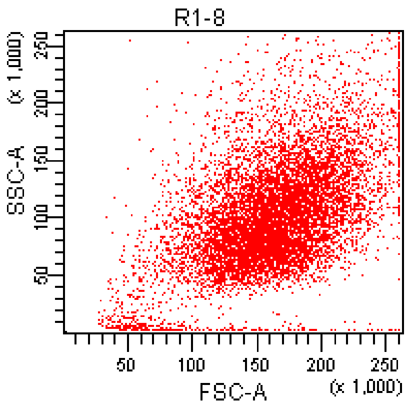

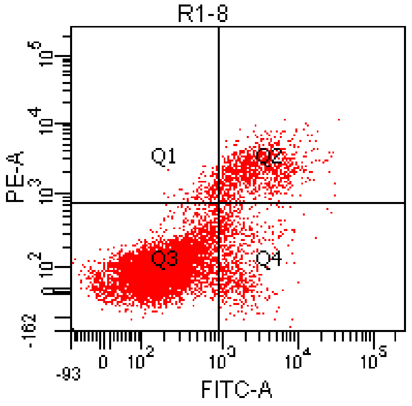

Tube: R1-8

Population	#Events	%Parent	%Total
All Events	10,000	####	100.0
Q1	107	1.1	1.1
Q2	1,063	10.6	10.6
Q3	8,082	80.8	80.8
Q4	748	7.5	7.5
