# Supplementary material for: Exosomes derived from M1 macrophages inhibit the proliferation of the A549 and H1299 lung cancer cell lines via the miRNA-let-7b-5p-GNG5 axis
Source: PeerJ. 2023 Jan 9;11:e14608. doi: 10.7717/peerj.14608 (PMC9835688; doi:10.7717/peerj.14608)
Supplement: Supplemental Information 11 [file peerj-11-14608-s011.zip › other raw data/Figure3/D FITC/HLF-A+EXO.pdf]

FACSDiva Version 6.1.3

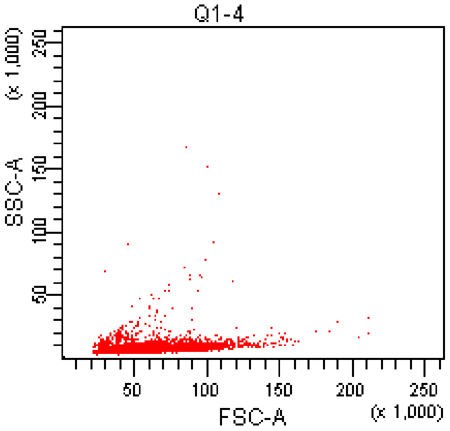

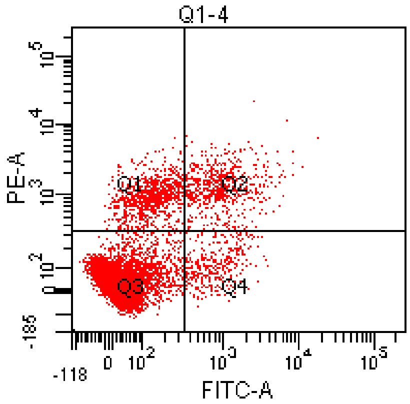

Tube: Q1-4

Population	#Events	%Parent	%Total
All Events	10,000	####	100.0
P1	320	3.2	3.2
Q1	832	8.3	8.3
Q2	650	6.5	6.5
Q3	8,166	81.7	81.7
Q4	352	3.5	3.5
