# Supplementary material for: Exosomes derived from M1 macrophages inhibit the proliferation of the A549 and H1299 lung cancer cell lines via the miRNA-let-7b-5p-GNG5 axis
Source: PeerJ. 2023 Jan 9;11:e14608. doi: 10.7717/peerj.14608 (PMC9835688; doi:10.7717/peerj.14608)
Supplement: Supplemental Information 11 [file peerj-11-14608-s011.zip › other raw data/Figure4/F FITC/ASO-NC.pdf]

FACSDiva Version 6.1.3

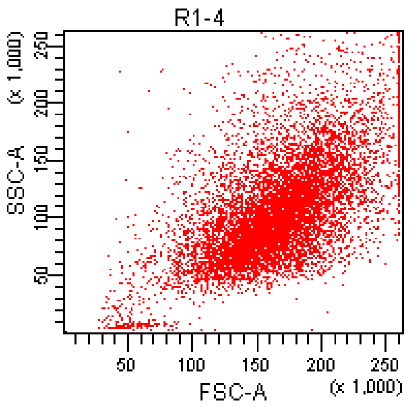

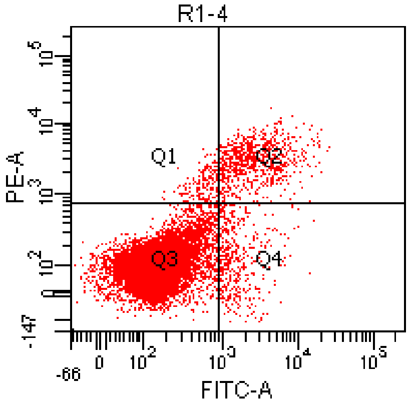

Tube: R1-4

Population	#Events	%Parent	%Total
All Events	10,000	####	100.0
Q1	157	1.6	1.6
Q2	800	8.0	8.0
Q3	8,555	85.6	85.6
Q4	488	4.9	4.9
