# Supplementary material for: Exosomes derived from M1 macrophages inhibit the proliferation of the A549 and H1299 lung cancer cell lines via the miRNA-let-7b-5p-GNG5 axis
Source: PeerJ. 2023 Jan 9;11:e14608. doi: 10.7717/peerj.14608 (PMC9835688; doi:10.7717/peerj.14608)
Supplement: Supplemental Information 11 [file peerj-11-14608-s011.zip › other raw data/Figure6/D FITC/ASO-GNG5.pdf]

FACSDiva Version 6.1.3

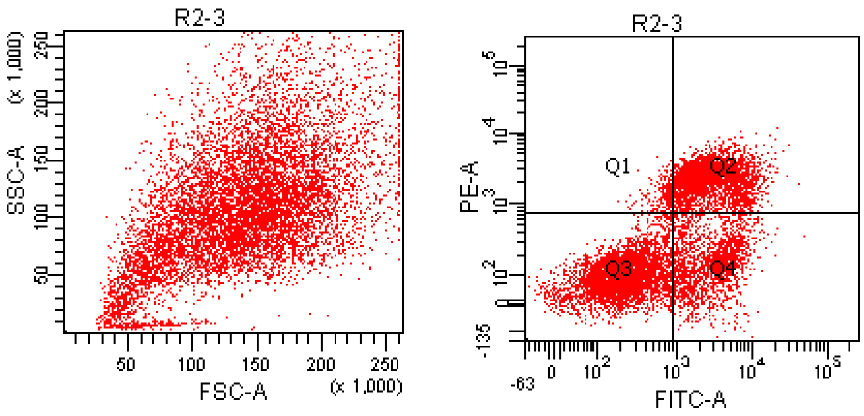

Tube: R2-3

Population	#Events	%Parent	%Total
All Events	10,000	####	100.0
Q1	183	1.8	1.8
Q2	3,189	31.9	31.9
Q3	4,603	46.0	46.0
Q4	2,025	20.2	20.2
