# Supplementary figures and images for: Exosomes derived from M1 macrophages inhibit the proliferation of the A549 and H1299 lung cancer cell lines via the miRNA-let-7b-5p-GNG5 axis (part 4 of 4)
Source: PeerJ. 2023 Jan 9;11:e14608. doi: 10.7717/peerj.14608 (PMC9835688; doi:10.7717/peerj.14608)

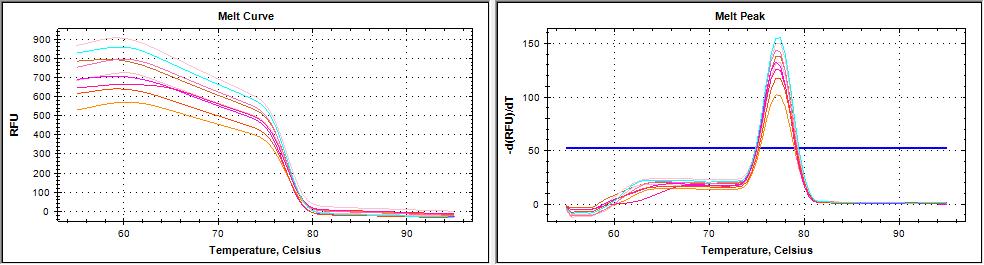

Supplement: Supplemental Information 11 [file peerj-11-14608-s011.zip › other raw data/Figure4/A/miRNA-23a-3P Melting curve.jpg]

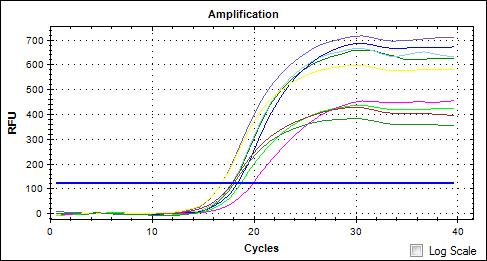

Supplement: Supplemental Information 11 [file peerj-11-14608-s011.zip › other raw data/Figure4/A/miRNA-30a-3p Amplification curve.jpg]

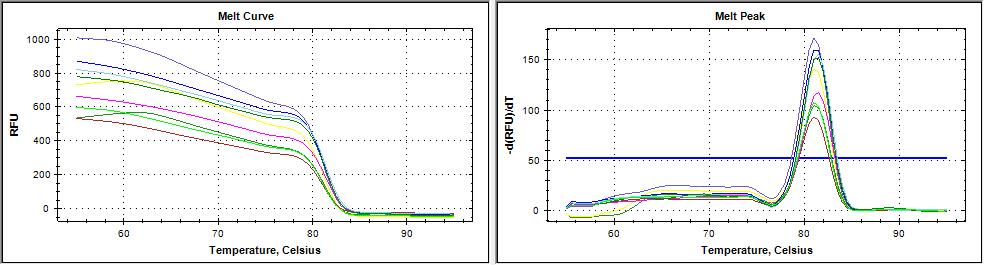

Supplement: Supplemental Information 11 [file peerj-11-14608-s011.zip › other raw data/Figure4/A/miRNA-30a-3p Melting curve.jpg]

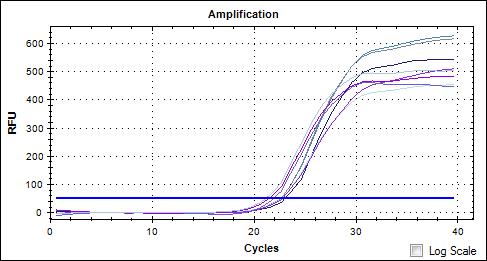

Supplement: Supplemental Information 11 [file peerj-11-14608-s011.zip › other raw data/Figure4/A/miRNA-486-5p Amplification curve.jpg]

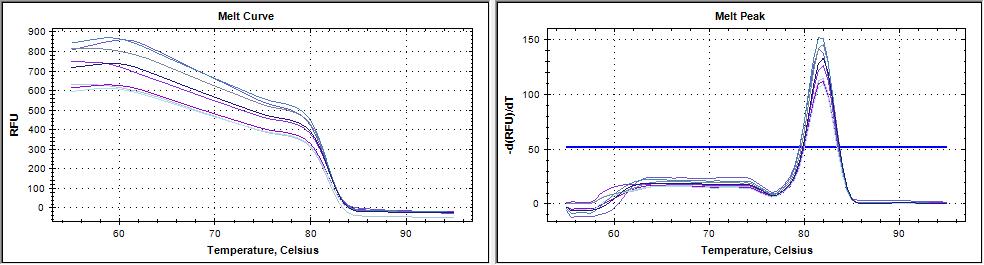

Supplement: Supplemental Information 11 [file peerj-11-14608-s011.zip › other raw data/Figure4/A/miRNA-486-5p Melting curve.jpg]

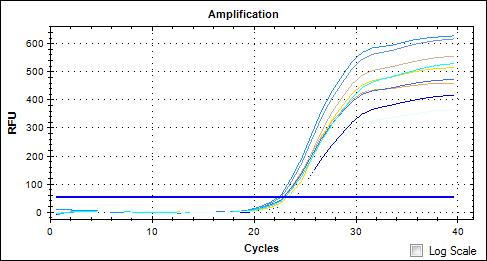

Supplement: Supplemental Information 11 [file peerj-11-14608-s011.zip › other raw data/Figure4/A/miRNA-let-7b-5p Amplification curve.jpg]

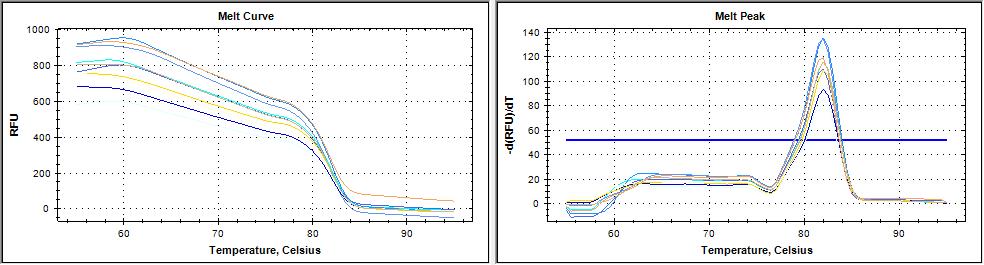

Supplement: Supplemental Information 11 [file peerj-11-14608-s011.zip › other raw data/Figure4/A/miRNA-let-7b-5p Melting curve.jpg]

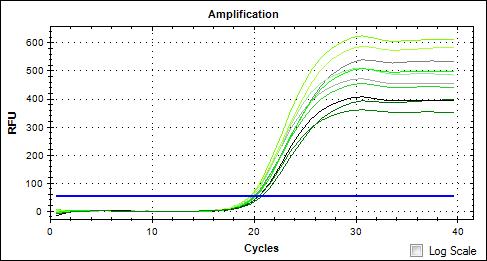

Supplement: Supplemental Information 11 [file peerj-11-14608-s011.zip › other raw data/Figure4/A/u6 Amplification curve.jpg]

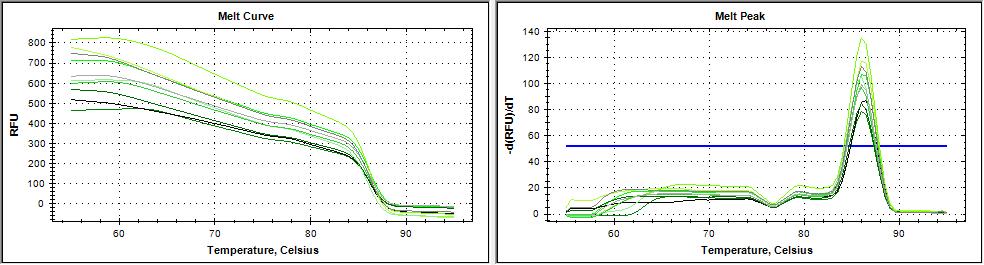

Supplement: Supplemental Information 11 [file peerj-11-14608-s011.zip › other raw data/Figure4/A/u6 Melting curve.jpg]

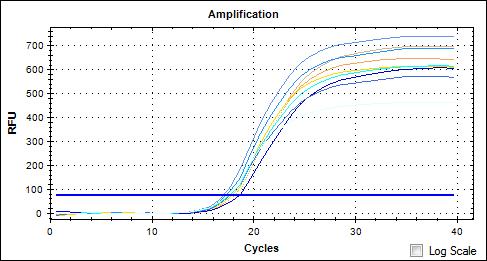

Supplement: Supplemental Information 11 [file peerj-11-14608-s011.zip › other raw data/Figure4/B/mi-RNA-let-7b-5p Amplification curve.jpg]

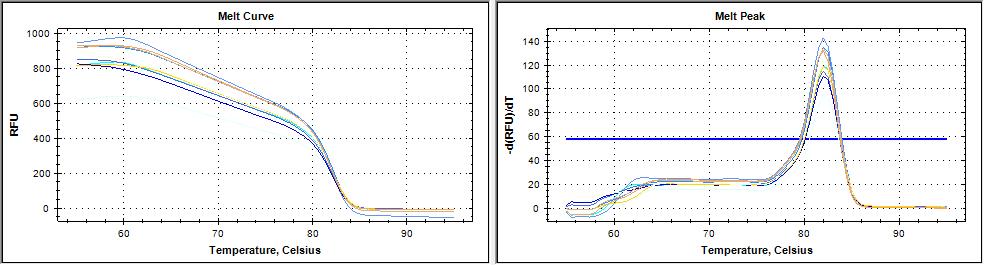

Supplement: Supplemental Information 11 [file peerj-11-14608-s011.zip › other raw data/Figure4/B/mi-RNA-let-7b-5p Melting curve.jpg]

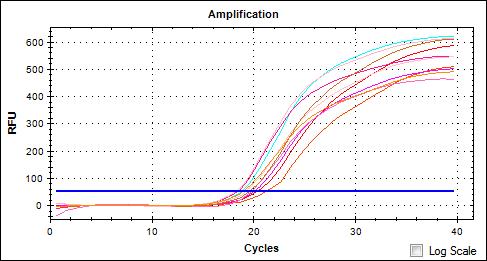

Supplement: Supplemental Information 11 [file peerj-11-14608-s011.zip › other raw data/Figure4/B/miRNA-23a-3p Amplification curve.jpg]

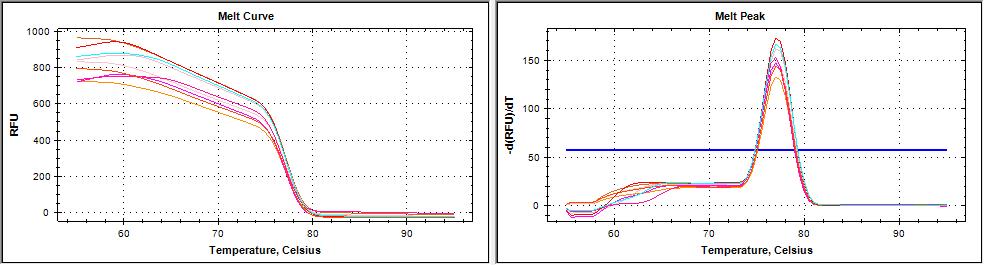

Supplement: Supplemental Information 11 [file peerj-11-14608-s011.zip › other raw data/Figure4/B/miRNA-23a-3p Melting curve.jpg]

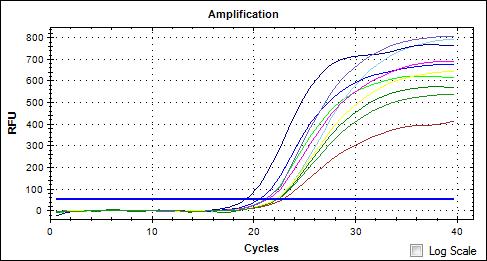

Supplement: Supplemental Information 11 [file peerj-11-14608-s011.zip › other raw data/Figure4/B/miRNA-30a-3p Amplification curve.jpg]

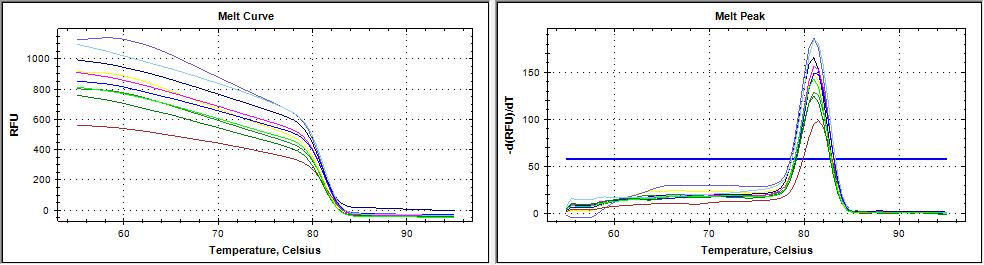

Supplement: Supplemental Information 11 [file peerj-11-14608-s011.zip › other raw data/Figure4/B/miRNA-30a-3p Melting curve.jpg]

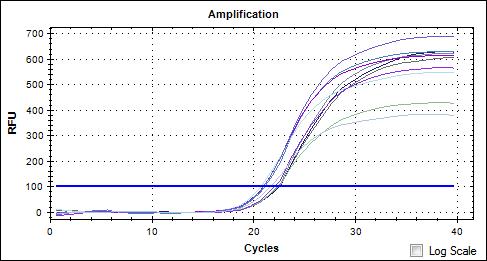

Supplement: Supplemental Information 11 [file peerj-11-14608-s011.zip › other raw data/Figure4/B/miRNA-486-5p Amplification curve.jpg]

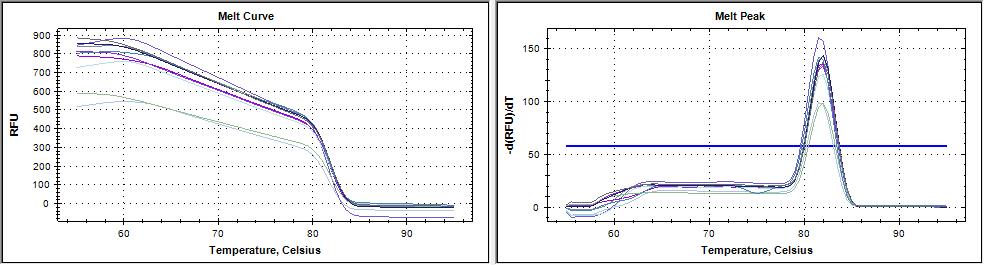

Supplement: Supplemental Information 11 [file peerj-11-14608-s011.zip › other raw data/Figure4/B/miRNA-486-5p Melting curve.jpg]

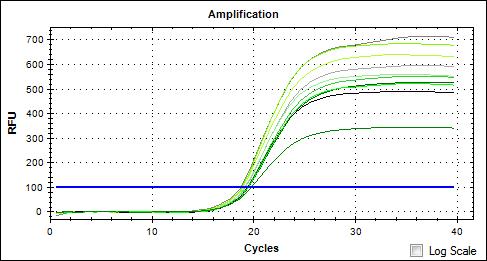

Supplement: Supplemental Information 11 [file peerj-11-14608-s011.zip › other raw data/Figure4/B/U6 Amplification curve.jpg]

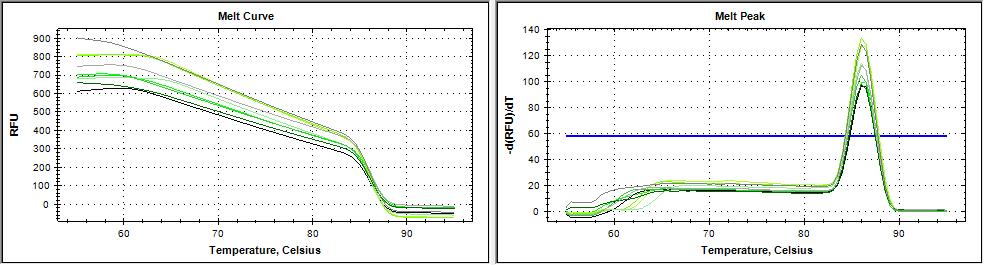

Supplement: Supplemental Information 11 [file peerj-11-14608-s011.zip › other raw data/Figure4/B/U6 Melting curve.jpg]

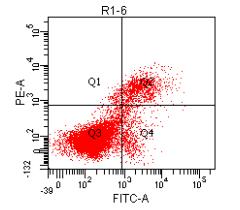

Supplement: Supplemental Information 11 [file peerj-11-14608-s011.zip › other raw data/Figure4/F FITC/ASO-mmiRNA-let-7b-5p.jpg]

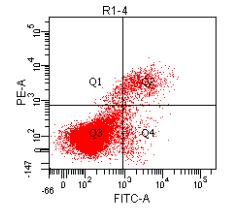

Supplement: Supplemental Information 11 [file peerj-11-14608-s011.zip › other raw data/Figure4/F FITC/ASO-NC.jpg]

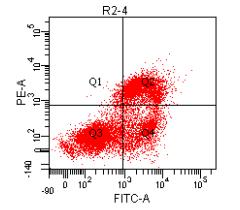

Supplement: Supplemental Information 11 [file peerj-11-14608-s011.zip › other raw data/Figure4/F FITC/miRNA-let-7b-5p mimics.jpg]

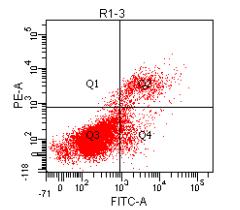

Supplement: Supplemental Information 11 [file peerj-11-14608-s011.zip › other raw data/Figure4/F FITC/NC mimics.jpg]

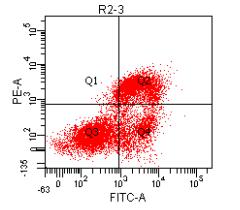

Supplement: Supplemental Information 11 [file peerj-11-14608-s011.zip › other raw data/Figure6/D FITC/ASO-GNG5.jpg]

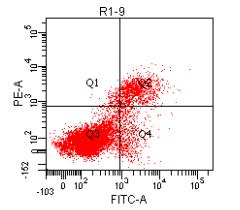

Supplement: Supplemental Information 11 [file peerj-11-14608-s011.zip › other raw data/Figure6/D FITC/ASO-NC.jpg]

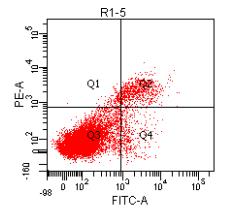

Supplement: Supplemental Information 11 [file peerj-11-14608-s011.zip › other raw data/Figure6/D FITC/pcDNA3.1-GNG5.jpg]

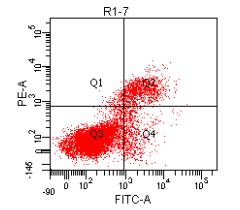

Supplement: Supplemental Information 11 [file peerj-11-14608-s011.zip › other raw data/Figure6/D FITC/pcDNA3.1.jpg]

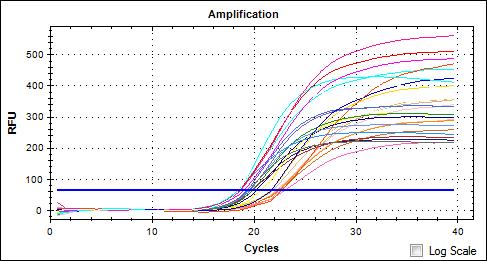

Supplement: Supplemental Information 11 [file peerj-11-14608-s011.zip › other raw data/Figure7/C/Amplification curve.jpg]

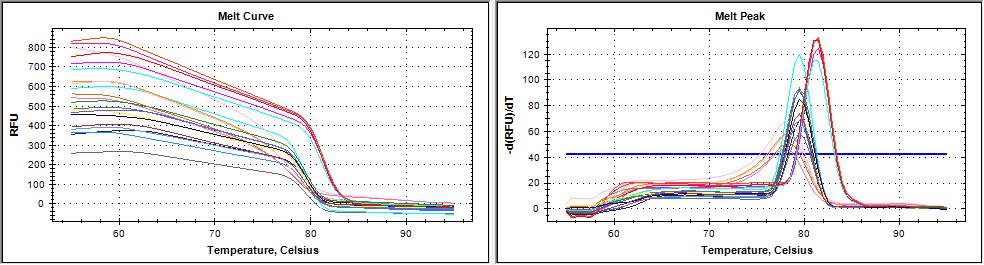

Supplement: Supplemental Information 11 [file peerj-11-14608-s011.zip › other raw data/Figure7/C/Melting curve.jpg]
